# A genome-wide association study for survival from a multi-centre European study identified variants associated with COVID-19 risk of death

**DOI:** 10.1038/s41598-024-53310-x

**Published:** 2024-02-06

**Authors:** Francesca Minnai, Filippo Biscarini, Martina Esposito, Tommaso A. Dragani, Luis Bujanda, Souad Rahmouni, Marta E. Alarcón-Riquelme, David Bernardo, Elena Carnero-Montoro, Maria Buti, Hugo Zeberg, Rosanna Asselta, Manuel Romero-Gómez, GEN-COVID Multicenter Study, Francesca Mari, Sergio Daga, Ilaria Meloni, Giulia Brunelli, Mirjam Lista, Debora Maffeo, Elena Pasquinelli, Enrica Antolini, Simona Letizia Basso, Samantha Minetto, Giulia Rollo, Angela Rina, Martina Rozza, Rossella Tita, Maria Antonietta Mencarelli, Caterina Lo Rizzo, Anna Maria Pinto, Francesca Ariani, Francesca Montagnani, Mario Tumbarello, Ilaria Rancan, Massimiliano Fabbiani, Paolo Cameli, David Bennett, Federico Anedda, Simona Marcantonio, Sabino Scolletta, Federico Franchi, Maria Antonietta Mazzei, Susanna Guerrini, Edoardo Conticini, Luca Cantarini, Bruno Frediani, Danilo Tacconi, Chiara Spertilli Raffaelli, Arianna Emiliozzi, Marco Feri, Alice Donati, Raffaele Scala, Luca Guidelli, Genni Spargi, Marta Corridi, Cesira Nencioni, Leonardo Croci, Gian Piero Caldarelli, Davide Romani, Paolo Piacentini, Maria Bandini, Elena Desanctis, Silvia Cappelli, Anna Canaccini, Agnese Verzuri, Valentina Anemoli, Agostino Ognibene, Maria Lorubbio, Alessandro Pancrazzi, Massimo Vaghi, Antonella D.’Arminio Monforte, Federica Gaia Miraglia, Mario U. Mondelli, Stefania Mantovani, Raffaele Bruno, Marco Vecchia, Marcello Maffezzoni, Enrico Martinelli, Massimo Girardis, Stefano Busani, Sophie Venturelli, Andrea Cossarizza, Andrea Antinori, Alessandra Vergori, Stefano Rusconi, Matteo Siano, Arianna Gabrieli, Agostino Riva, Daniela Francisci, Elisabetta Schiaroli, Carlo Pallotto, Saverio Giuseppe Parisi, Monica Basso, Sandro Panese, Stefano Baratti, Pier Giorgio Scotton, Francesca Andretta, Mario Giobbia, Renzo Scaggiante, Francesca Gatti, Francesco Castelli, Eugenia Quiros-Roldan, Melania Degli Antoni, Isabella Zanella, Matteo della Monica, Carmelo Piscopo, Mario Capasso, Roberta Russo, Immacolata Andolfo, Achille Iolascon, Giuseppe Fiorentino, Massimo Carella, Marco Castori, Giuseppe Merla, Gabriella Maria Squeo, Filippo Aucella, Pamela Raggi, Rita Perna, Matteo Bassetti, Antonio Di Biagio, Maurizio Sanguinetti, Luca Masucci, Alessandra Guarnaccia, Serafina Valente, Alex Di Florio, Marco Mandalà, Alessia Giorli, Lorenzo Salerni, Patrizia Zucchi, Pierpaolo Parravicini, Elisabetta Menatti, Tullio Trotta, Ferdinando Giannattasio, Gabriella Coiro, Gianluca Lacerenza, Cristina Mussini, Luisa Tavecchia, Lia Crotti, Gianfranco Parati, Roberto Menè, Maurizio Sanarico, Marco Gori, Francesco Raimondi, Alessandra Stella, Filippo Biscarini, Tiziana Bachetti, Maria Teresa La Rovere, Maurizio Bussotti, Serena Ludovisi, Katia Capitani, Simona Dei, Sabrina Ravaglia, Annarita Giliberti, Giulia Gori, Rosangela Artuso, Elena Andreucci, Antonio Perrella, Francesco Bianchi, Paola Bergomi, Emanuele Catena, Riccardo Colombo, Sauro Luchi, Giovanna Morelli, Paola Petrocelli, Sarah Iacopini, Sara Modica, Silvia Baroni, Giulia Micheli, Marco Falcone, Donato Urso, Giusy Tiseo, Tommaso Matucci, Alice Pulcinelli, Davide Grassi, Claudio Ferri, Franco Marinangeli, Francesco Brancati, Antonella Vincenti, Valentina Borgo, Stefania Lombardi, Mirco Lenzi, Massimo Antonio Di Pietro, Letizia Attala, Cecilia Costa, Andrea Gabbuti, Alessio Bellucci, Marta Colaneri, Patrizia Casprini, Cristoforo Pomara, Massimiliano Esposito, Roberto Leoncini, Michele Cirianni, Lucrezia Galasso, Marco Antonio Bellini, Chiara Gabbi, Nicola Picchiotti, Simone Furini, Elisabetta Pelo, Barbara Minuti, Francesca Gerundino, Chiara Lazzeri, Arianna Vecchi, Leila Bianchi, Elisabetta Venturini, Carlotta Montagnani, Elena Chiappini, Cristina Beltrami, Luisa Galli, Israel Fernandez-Cadenas, Chiara Fallerini, Kristina Zguro, Susanna Croci, Margherita Baldassarri, Mirella Bruttini, Simone Furini, Alessandra Renieri, Francesca Colombo

**Affiliations:** 1 Institute of Biomedical Technologies, National Research Council, Via F.lli Cervi, 93, 20054 Segrate, MI Italy; 2 Department of Medical Biotechnology and Translational Medicine (BioMeTra), Università degli Studi di Milano, Milan, Italy; 3 Institute of Agricultural Biology and Biotechnology, National Research Council, Milan, Italy; 4 Aspidia S.R.L., Milan, Italy; 5 Centro de Investigación Biomédica en Red de Enfermedades Hepáticas y Digestivas (CIBERehd), Biodonostia Health Research Institute, Universidad del País Vasco (UPV/EHU), San Sebastián, Spain; 6 GIGA-Medical Genomics Unit, Uliege, Liege, Belgium; 7 GENYO, University of Granada, Andalusian Regional Government, Granada, Spain; 8 Institute for Environmental Medicine, Karolinska Institute, Solna, Sweden; 9 Centro de Investigación Biomédica en Red en Enfermedades Hepáticas y Digestivas (CIBEREHD), Instituto de Salud Carlos III (ISCIII), Madrid, Spain; 10 Mucosal Immunology Lab, Unit of Excellence, Institute of Biomedicine and Molecular Genetics (IBGM), University of Valladolid-CSIC, Valladolid, Spain; 11 University of Granada, Granada, Spain; 12 Vall D’Hebron Institut de Recerca, Barcelona, Spain; 13 Department of Physiology and Pharmacology, Karolinska Institutet, Stockholm, Sweden; 14 Department of Biomedical Sciences, Humanitas University, Pieve Emanuele, MI Italy; 15 IRCCS Humanitas Research Hospital, Rozzano, MI Italy; 16 Digestive Diseases Unit and CiberehdVirgen del Rocío University HospitalInstitute of Biomedicine of Seville (HUVR/CSIC/US), University of Seville, Seville, Spain; 17 Stroke Pharmacogenomics and Genetics Group, Sant Pau Hospital Research Institute, Barcelona, Spain; 18 Medical Genetics, University of Siena, 53100 Siena, Italy; 19 Department of Medical Biotechnologies, Med Biotech Hub and Competence Center, University of Siena, 53100 Siena, Italy; 20 Genetica Medica, Azienda Ospedaliero-Universitaria Senese, 53100 Siena, Italy; 21 Dipartimento di Ingegneria dell’Energia Elettrica e dell’Informazione “Guglielmo Marconi”, Alma Mater Studiorum - Università di Bologna, Bologna, Italy; 22 Infectious and Tropical Diseases Unit, Department of Medical Sciences, Azienda Ospedaliera Universitaria Senese, 53100 Siena, Italy; 23 Unit of Respiratory Diseases and Lung Transplantation, Department of Internal and Specialist Medicine, University of Siena, 53100 Siena, Italy; 24 Unit of Intensive Care Medicine, Department of Emergency and Urgency, Medicine, Surgery and Neurosciences, Siena University Hospital, 53100 Siena, Italy; 25 Unit of Diagnostic Imaging, Department of Medical, Surgical and Neuro Sciences and Radiological Sciences, University of Siena, 53100 Siena, Italy; 26 Rheumatology Unit, Department of Medicine, Surgery and Neurosciences, University of Siena, Policlinico Le Scotte, 53100 Siena, Italy; 27 Infectious Diseases Unit, Department of Specialized and Internal Medicine, San Donato Hospital, 52100 Arezzo, Italy; 28 Anesthesia Unit, Department of Emergency, San Donato Hospital, Arezzo, Italy; 29 Pneumology Unit and UTIP, Department of Specialized and Internal Medicine, San Donato Hospital, 52100 Arezzo, Italy; 30 Anesthesia Unit, Department of Emergency, Misericordia Hospital, 58100 Grosseto, Italy; 31 Infectious Diseases Unit, Department of Specialized and Internal Medicine, Misericordia Hospital, 58100 Grosseto, Italy; 32 Clinical Chemical Analysis Laboratory, Misericordia Hospital, 58100 Grosseto, Italy; 33 Dipartimento di Prevenzione, Azienda USL Toscana Sud Est, 53100 Arezzo, Italy; 34 Dipartimento Tecnico-Scientifico Territoriale, Azienda USL Toscana Sud Est, 53100 Arezzo, Italy; 35 UOC Laboratorio Analisi Chimico Cliniche, 52100 Arezzo, Italy; 36 Chirurgia Vascolare, Ospedale Maggiore di Crema, 26013 Crema, Italy; 37 Department of Health Sciences, Clinic of Infectious Diseases, ASST Santi Paolo e Carlo, University of Milan, 20142 Milan, Italy; 38 Division of Clinical Immunology - Infectious Diseases, Department of Medicine, Fondazione IRCCS Policlinico San Matteo, 27100 Pavia, Italy; 39 Department of Internal Medicine and Therapeutics, University of Pavia, 27100 Pavia, Italy; 40 University of Pavia, 27100 Pavia, Italy; 41 Department of Respiratory Diseases, Azienda Ospedaliera di Cremona, 26100 Cremona, Italy; 42 Department of Anesthesia and Intensive Care, University of Modena and Reggio Emilia, 41121 Modena, Italy; 43 Department of Medical and Surgical Sciences for Children and Adults, University of Modena and Reggio Emilia, 41121 Modena, Italy; 44 HIV/AIDS Department, National Institute for Infectious Diseases, IRCCS, Lazzaro Spallanzani, 00161 Rome, Italy; 45 III Infectious Diseases Unit, ASST-FBF-Sacco, 20146 Milan, Italy; 46 Department of Biomedical and Clinical Sciences Luigi Sacco, University of Milan, 20146 Milan, Italy; 47 Infectious Diseases Clinic, “Santa Maria Della Misericordia” Hospital, University of Perugia, 06100 Perugia, Italy; 48 Department of Molecular Medicine, University of Padova, Padua, Italy; 49 Clinical Infectious Diseases, Mestre Hospital, Venezia, Italy; 50 Department of Infectious Diseases, Treviso Hospital, Local Health Unit 2 Marca Trevigiana, Treviso, Italy; 51 Infectious Diseases Clinic, ULSS1, Belluno, Italy; 52 Department of Infectious and Tropical Diseases, University of Brescia and ASST Spedali Civili Hospital, Brescia, Italy; 53 Department of Molecular and Translational Medicine, University of Brescia, Brescia, Italy; 54 Clinical Chemistry Laboratory, Cytogenetics and Molecular Genetics Section, Diagnostic Department, ASST Spedali Civili di Brescia, Brescia, Italy; 55 Medical Genetics and Laboratory of Medical Genetics Unit, A.O.R.N. “Antonio Cardarelli”, Naples, Italy; 56 Department of Molecular Medicine and Medical Biotechnology, University of Naples Federico II, Naples, Italy; 57 CEINGE Biotecnologie Avanzate, Naples, Italy; 58 Unit of Respiratory Physiopathology, AORN dei Colli, Monaldi Hospital, Naples, Italy; 59 Division of Medical Genetics, Fondazione IRCCS Casa Sollievo della Sofferenza Hospital, San Giovanni Rotondo, Italy; 60 Laboratory of Regulatory and Functional Genomics, Fondazione IRCCS Casa Sollievo della Sofferenza, Foggia, Italy; 61 Department of Medical Sciences, Fondazione IRCCS Casa Sollievo della Sofferenza Hospital, San Giovanni Rotondo, Italy; 62 Clinical Trial Office, Fondazione IRCCS Casa Sollievo della Sofferenza Hospital, San Giovanni Rotondo, Italy; 63 Department of Health Sciences, University of Genova, Genoa, Italy; 64 Infectious Diseases Clinic, Policlinico San Martino Hospital, IRCCS for Cancer Research Genova, Genoa, Italy; 65 Microbiology, Fondazione Policlinico Universitario Agostino Gemelli IRCCS, Catholic University of Medicine, Rome, Italy; 66 Department of Laboratory Sciences and Infectious Diseases, Fondazione Policlinico Universitario A. Gemelli IRCCS, Rome, Italy; 67 Department of Cardiovascular Diseases, University of Siena, Siena, Italy; 68 Otolaryngology Unit, University of Siena, Siena, Italy; 69 Department of Internal Medicine, ASST Valtellina e Alto Lario, Sondrio, Italy; 70 Study Coordinator Oncologia Medica e Ufficio Flussi Sondrio, Sondrio, Italy; 71 First Aid Department, Luigi Curto Hospital, Polla, Salerno, Italy; 72 Department of Pharmaceutical Medicine, Misericordia Hospital, Grosseto, Italy; 73 Infectious Diseases Clinics, University of Modena and Reggio Emilia, Modena, Italy; 74 U.O.C. Medicina, ASST Nord Milano, Ospedale Bassini, Cinisello Balsamo, MI Italy; 75 Department of Cardiovascular, Neural and Metabolic Sciences, San Luca Hospital, Istituto Auxologico Italiano, IRCCS, Milan, Italy; 76 Department of Medicine and Surgery, University of Milano-Bicocca, Milan, Italy; 77 Center for Cardiac Arrhythmias of Genetic Origin, Istituto Auxologico Italiano, IRCCS, Milan, Italy; 78 Laboratory of Cardiovascular Genetics, Istituto Auxologico Italiano, IRCCS, Milan, Italy; 79 Member of the European Reference Network for Rare, Low Prevalence and Complex Diseases of the Heart-ERN GUARD-Heart, Amsterdam, The Netherlands; 80 Milan, Italy; 81 DIISM-SAILAB, University of Siena, Siena, Italy; 82 Maasai, I3S CNRS, Université Côte d’Azur, Nice, France; 83 Laboratorio di Biologia Bio@SNS, Scuola Normale Superiore, Pisa, Italy; 84 CNR-Consiglio Nazionale delle Ricerche, Istituto di Biologia e Biotecnologia Agraria (IBBA), Milan, Italy; 85 Direzione Scientifica, Istituti Clinici Scientifici Maugeri IRCCS, Pavia, Italy; 86 Department of Cardiology, Institute of Montescano, Istituti Clinici Scientifici Maugeri IRCCS, Pavia, Italy; 87 Department of Cardiology, Institute of Milan, Istituti Clinici Scientifici Maugeri IRCCS, Pavia, Italy; 88 Fondazione IRCCS Ca’ Granda Ospedale Maggiore Policlinico, Milan, Italy; 89 Core Research Laboratory, ISPRO, Florence, Italy; 90 Health Management, Azienda USL Toscana Sud Est, Tuscany, Italy; 91 IRCCS C. Mondino Foundation, Pavia, Italy; 92 Medical Genetics Unit, Meyer Children’s University Hospital, Florence, Italy; 93 Pneumology Unit, Department of Medicine, Misericordia Hospital, Grosseto, Italy; 94 Department of Anesthesia and Intensive Care Unit, ASST Fatebenefratelli Sacco, Luigi Sacco Hospital, Polo Universitario, University of Milan, Milan, Italy; 95 Infectious Disease Unit, Hospital of Lucca, Lucca, Italy; 96 Department of Diagnostic and Laboratory Medicine, Institute of Biochemistry and Clinical Biochemistry, Fondazione Policlinico Universitario A. Gemelli IRCCS, Catholic University of the Sacred Heart, Rome, Italy; 97 Clinic of Infectious Diseases, Catholic University of the Sacred Heart, Rome, Italy; 98 Infectious Diseases Unit, Department of Clinical and Experimental Medicine, University of Pisa, Pisa, Italy; 99 Department of Clinical Medicine, Public Health, Life and Environment Sciences, University of L’Aquila, L’Aquila, Italy; 100 Anesthesiology and Intensive Care, University of L’Aquila, L’Aquila, Italy; 101 Department of Life, Health and Environmental Sciences, University of L’Aquila, 67100 L’Aquila, Italy; 102 Human Functional Genomics Laboratory, IRCCS San Raffaele Roma, 00167 Rome, Italy; 103 Infectious Disease Unit, Hospital of Massa, Massa, Italy; 104 Infectious Diseases Unit, USL Centro, Santa Maria Annunziata Hospital, Florence, Italy; 105 Laboratory of Clinical Pathology and Immunoallergy, Florence-Prato, Italy; 106 Department of Medical, Surgical and Advanced Technologies “G.F. Ingrassia”, University of Catania, Catania, Italy; 107 Laboratorio Patologia Clinica, Azienda Ospedaliero-Universitaria Senese, Siena, Italy; 108 Ambulatory Chronic Polipathology of Siena, Department of Medicine, Surgery and Neurosciences, University of Siena, Siena, Italy; 109 Department of Biosciences and Nutrition, Karolinska Institutet, Stockholm, Sweden; 110 Bioinformatics, University of Bologna, Bologna, Italy; 111 SOD Diagnostica Genetica, Azienda Ospedaliero Universitaria Careggi, Florence, Italy; 112 Infectious Diseases Unit, Department of Pediatrics, Meyer Children’s Hospital, Florence, Italy; 113 Department of Health Sciences, University of Florence, Florence, Italy

**Keywords:** Genome-wide association studies, SARS-CoV-2, Genetics research

## Abstract

The clinical manifestations of SARS-CoV-2 infection vary widely among patients, from asymptomatic to life-threatening. Host genetics is one of the factors that contributes to this variability as previously reported by the COVID-19 Host Genetics Initiative (HGI), which identified sixteen loci associated with COVID-19 severity. Herein, we investigated the genetic determinants of COVID-19 mortality, by performing a case-only genome-wide survival analysis, 60 days after infection, of 3904 COVID-19 patients from the GEN-COVID and other European series (EGAS00001005304 study of the COVID-19 HGI). Using imputed genotype data, we carried out a survival analysis using the Cox model adjusted for age, age2, sex, series, time of infection, and the first ten principal components. We observed a genome-wide significant (*P*-value < 5.0 × 10^−8^) association of the rs117011822 variant, on chromosome 11, of rs7208524 on chromosome 17, approaching the genome-wide threshold (*P*-value = 5.19 × 10^−8^). A total of 113 variants were associated with survival at *P*-value < 1.0 × 10^−5^ and most of them regulated the expression of genes involved in immune response (e.g., CD300 and KLR genes), or in lung repair and function (e.g., FGF19 and CDH13). Overall, our results suggest that germline variants may modulate COVID-19 risk of death, possibly through the regulation of gene expression in immune response and lung function pathways.

## Introduction

The clinical manifestations of COVID-19, the disease caused by the SARS-CoV-2 virus, vary widely from mild respiratory symptoms to severe organ failure and death^1–3^. The mortality rate of COVID-19 also shows remarkable temporal and spatial heterogeneity across the world^4,5^. Several risk factors have been associated with increased mortality, such as older age, male sex, and presence of comorbidities^6,7^.

The role of genetics in modulating the severity and outcome of COVID-19 has been a subject of intense research and growing evidence supports the existence of individual genetic factors predisposing to a severe outcome^8^. For example, the COVID-19 Host Genetics Initiative (HGI) consortium performed large-scale meta-analyses of genome-wide data from over nine thousand critically ill cases (defined as patients who required respiratory support or died from COVID-19) and over 25 thousand hospitalized cases with moderate or severe disease, compared with up to five million controls^9^. These studies identified several genetic loci associated with either critical illness or hospitalization due to COVID-19. However, the consortium did not address the survival probability of SARS-CoV-2 infected patients, which is a relevant time-to-event phenotype that has received limited attention so far. Indeed, most studies have focused on COVID-19 severity (reviewed in^10^), some on mortality^11–13^ and very few on survival^14^, mainly investigating candidate gene polymorphisms rather than performing genome-wide analyses.

In this study, we conducted a genome-wide survival analysis to identify variants affecting the risk of death from acute SARS-CoV-2 infection. We used genotyping and clinical follow-up data (at 60 days post-infection) from about four thousand COVID-19 patients from five European cohorts. We adjusted the analyses for known non-genetic independent prognostic factors, like age, sex, and pandemic wave, which were available for all patients.

## Materials and methods

### Case series

The case series investigated in this study comprised 3904 COVID-19 patients molecularly tested for SARS-CoV-2 infection and enrolled for host genetics studies at several recruiting centres, in the context of the international COVID-19 HGI. Patients from GEN-COVID Multicenter Study and from the series included in the European Genome-Phenome Archive (EGA) study number EGAS00001005304 (except for BRACOVID and INMUNGEN-CoV series), with 60-days follow-up information and full data about sex, age, and infection date were analysed. BRACOVID patients were not included in the analysis since their data were not shared with us. The INMUNGEN-CoV series was not included in our study, since 90% of patients did not have survival data and, in addition, they were genotyped with a different SNP-array. Patients provided written informed consent to the use of their biological samples and data for research purposes. Personal data treatment was GDPR compliant. The research was approved by the Committees for Ethics of the recruiting centres.

### Survival analysis

A multivariable Cox proportional hazard model with demographic-clinical features (i.e., age, sex, series, and time of infection) was used for survival analysis^15^ (age was considered as both a linear and non-linear term, the latter defined as age squared and hereafter named age^2^). The R *survival* package^16^ was used to draw Kaplan–Meier (KM) curves and run the log-rank test in R (v. 3.6.0) environment. The hazard proportionality assumptions were verified through the function “*cox.zph()*” of the *survival* package. The variables that were found to impact on survival from the log-rank test (with *P*-value < 0.05) were analysed both in multivariable Cox regression and in a weighted multivariable analysis to account for non-proportional hazards^17^, using the *survival* R package and the *coxphw* R package^18^ (applying the Average Hazards Ratio method, by setting the parameter template = “AHR”), respectively. Cox and log-rank test *P* values < 0.05 (two-sided) indicated sufficient statistical significance.

### Genotyping data quality check, principal component analysis, and imputation

Genome-wide genotyping data were available at EGA (study number EGAS00001005304) and University of Siena. The LiftOver tool (https://liftover.broadinstitute.org/) was used to convert genomic coordinates and bring all the datasets to the same genomic build (GRCh38). PLINK v.2 software^19^ was used to carry out genotype quality control (QC) steps (Supplementary Figure S1). In detail, for each patient series, per-sample and per-variant QC steps were performed, excluding samples with call rate < 99% and excess of heterozygosity (F >  ± 0.2), removing insertions/deletions, duplicated and non-informative variants, and filtering out single nucleotide polymorphisms (SNPs) with genotyping call rate < 99% and Hardy–Weinberg equilibrium test *P*-value < 1.0 × 10^−10^. Then, all the datasets were merged, and an additional round of QC was carried out to remove duplicated and related individuals and patients for whom no survival data were available. Additionally, SNPs with minor allele frequency (MAF) < 1% were filtered out, together with SNPs mapping in regions of extended linkage disequilibrium (LD)^20^. PLINK v.2 software was also used to carry out principal components analysis (PCA): we plotted PC1 versus PC2 visualizing samples according to our five patient series (i.e., BelCovid, GENCOVID, Hostage, SPGRX, and SweCovid; Supplementary Figure S2A). Additionally, to better visualize the ancestry of our patients, we projected the first four principal components of our patients together with those of 2504 individuals from five different populations, selected from 1000 Genomes Project^21^: Africans, Americans, South-East Asians, East Asians, and Europeans (Supplementary Figure S2B and S2C). We defined as Europeans those patients that clustered together with 1000 Genomes Project European individuals. Genotype imputation to whole-genome sequence was carried out on the TopMed imputation server^22^ using Eagle v.2.4 for phasing^23^, minimac4 algorithm^24^, and TopMed r2 as reference panel^25^. Finally, SNPs with a low-quality imputation (R^2^ ≤ 0.3)^26^ and with a MAF < 0.02 were filtered out. This second MAF filter was applied after imputation to remove variants with very low frequency alleles, thus reducing the risk of the Cox model not-converging and of obtaining spurious association results when, in patients carrying the minor allele, few or no events (i.e., death) were observed.

### Genome-wide survival analysis

The associations between SNPs (additive model) and patient overall survival were assessed using multivariable Cox proportional hazard model, with the first 10 PCs, sex, age, age^2^, patient series, and time of infection (before or after the first pandemic wave, that we considered finished by the end of June 2020), as covariates, using the GenAbel package in R environment^27^. Correction for multiple testing was performed with the Benjamini–Hochberg method of false discovery rate (FDR)^28^. Top significant polymorphisms were also tested under a dominant model using the same covariates as above. This was done for the following two reasons: first, we hypothesized that the minor allele was increasing the risk of death by acting as a dominant allele or at least codominant; second, in this way we reduced the risk of artifacts in the Kaplan–Meier curves by comparing the probability of survival of patients homozygous for the major allele with that of patients with at least one minor allele in their genotype (due to the low number of patients homozygous for the minor allele). COVID-19 severity top-significantly associated variants (https://app.covid19hg.org/variants; analysis B2: Hospitalized COVID19 + vs. population controls)^9^ were also investigated in our study, by comparing *P*-values of association between these variants and survival with those reported by COVID-19 HGI.

A logistic regression between a binary status phenotype (live vs. dead during the 60-days follow-up) and genome-wide imputed SNPs was performed using PLINK v.2, with the first 10 PCs, sex, age, age^2^, time of infection, and patient series as covariates.

### Functional analyses

To investigate the functional role of the identified variants associated with survival to COVID-19, we used multiple databases to obtain more reliable and robust results, by pooling together partial results from each platform.

First, we looked for the variants associated with COVID-19 survival at *P*-value < 1.0 × 10^−5^, in the GTEx (Analysis V8 release, GTEx_Analysis_v8_eQTL_EUR.tar) and eQTLGen^29^ (https://www.eqtlgen.org/cis-eqtls.html) databases (accessed on 08/05/2023), to test whether they have already been reported as *cis* expression quantitative trait loci (eQTLs). For some variants, we also looked for eQTL SNPs in LD with them, using the LDexpress tool of LDlink^30^. We searched all tissue eQTLs reported in the European population and in LD with query variants at D’ > 0.8. An over-representation analysis of the gene list including all the target genes of the found eQTLs was done, using the WEB-based Gene SeT AnaLysis Toolkit^31^, to search for enriched Reactome and KEGG pathways.

Additionally, genes that included or are close to the variants associated with COVID-19 survival (*P*-value < 1.0 × 10^−5^) were retrieved. The ENSEMBL gene database was used with the R Bioconductor package “biomaRt”^32^ to find genes that lie at a maximum distance of ± 50 kb from detected SNP variants. The resulting list of genes was then used as input for functional analysis tools. We used the Database for Annotation, Visualization and Integrated Discovery (DAVID)^33^ with default functional categories and applying the high classification stringency parameters; Benjamini–Hochberg adjusted *P*-values (FDR) < 0.05 was considered as significance threshold of enrichment.

The GENE2FUNC functionality of the Functional Mapping and Annotation (FUMA) for GWAS platform^34^ was selected to retrieve gene ontologies (biological processes, molecular functions, cell compartments), differentially expressed genes associated with COVID-19, target tissues and metabolic pathways, with the following parameters: all background genes; Ensembl version v102; GTEx v8 (54 tissue types) and GTEx v8 (30 general tissue types) gene expression datasets; Benjamini-Hochberg (FDR) correction when testing for gene-set enrichment (threshold: FDR < 0.05); minimum overlapping genes with gene set: ≥ 2.

### Ethical approval and informed consent

The research was performed in accordance with the Declaration of Helsinki and was approved by the committees for Ethics of the recruiting centres, namely, the University Hospital (Azienda ospedaliero-universitaria Senese) ethical review board, Siena, Italy (Prot n. 16917, dated March 16th, 2020); Euskadi Ethics Committee, Donostia-San Sebastian, Spain, on April 6, 2020 (approval number PI2020064); Vall d’Hebron Ethical Committee, Barcelona, Spain; ethics committee of the Junta de Andalucia, Spain (ethics id: 0886-N-20 and 1954-N-20); the ethics committee of Humanitas Clinical and Research Center, Rozzano (MI), Italy (reference number, 316/20); Valladolid Ethics Committee (PI-201716) and the Granada Ethics Committee, Spain, on March 24, 2020, and April 13, 2020, respectively; Erasme Ethics committee, Bruxelles, Belgium (protocol P2020_209); the National Ethical Review Agency, Sweden (EPM; 2020-01623). Patients provided written informed consent to the use of their biological samples and data for research purposes. Personal data treatment was GDPR compliant.

## Results

### Age, sex, and period of infection are associated with COVID-19 patient survival

In this study, we included a total of 3904 European COVID-19 patients recruited in Italy (GEN-COVID and, in part, Hostage series), Spain (Hostage and SPGRX), Sweden (SWECOVID) and Belgium (BelCovid) (Table 1). The median age at infection was 63 years and male patients were slightly prevalent (58%). More than two thirds of patients included in this study were infected during the first COVID-19 wave (before June 30th, 2020). Most of enrolled patients were hospitalised (86%), but 78% of the whole series did not need to be admitted to the intensive care unit (ICU). Among the 3175 patients for whom we have information about the respiratory support, just a small fraction of patients included in the study (15%) did not need any oxygen support, whereas most of them (40%) received oxygen by mask or cannula, 11% received non-invasive ventilation, and 14% needed intubation. Information about comorbidities was available for 2887 patients and about a quarter of patients had a comorbidity (hypertension, diabetes, cancer, asthma, and heart failure, in order from the most to the least frequent) and 12% had at least two comorbidities. Considering a follow-up time of 60 days after the infection, approximately 11% of COVID-19 patients of this study have died.

**Table 1:** Clinical characteristics of patients included in the survival genome-wide association study.

Characteristic	BelCovid (n = 381)	GENCOVID (n = 1727)	Hostage (n = 1303)	SPGRX (n = 364)	SweCovid (n = 129)	Total (n = 3904)
Age at diagnosis, years, median (range)	59 (23–104)	60 (18–99)	65 (1–96)	71 (23–103)	61 (24–86)	63 (1–104)
Age at diagnosis, years, n (%)						
< 55	151 (39.6)	615 (35.6)	318 (24.4)	66 (18.1)	42 (32.6)	1192 (30.5)
55–64	89 (23.4)	416 (24.1)	321 (24.6)	59 (16.2)	31 (24.0)	916 (23.5)
65–74	83 (21.8)	345 (20.0)	363 (27.9)	88 (24.2)	30 (23.3)	909 (23.3)
75–84	43 (11.3)	227 (13.1)	202 (15.5)	88 (24.2)	24 (18.6)	584 (15.0)
> 85	15 (3.94)	124 (7.18)	99 (7.60)	63 (17.3)	2 (1.55)	303 (7.76)
Sex, n (%)						
Male	199 (52.2)	1032 (59.8)	773 (59.3)	188 (51.6)	91 (70.5)	2283 (58.5)
Female	182 (47.8)	695 (40.2)	530 (40.7)	176 (48.4)	38 (29.5)	1621 (41.5)
Infection before June 30th, 2020, n (%)						
No	41 (10.8)	1075 (62.2)	118 (9.06)	0	17 (13.2)	1251 (32.0)
Yes	340 (89.2)	652 (37.8)	1185 (90.9)	364 (100)	112 (86.8)	2653 (68.0)
Hospitalization, n (%)						
No	125 (32.8)	357 (20.7)	9 (0.691)	52 (14.3)	0	543 (13.9)
Yes	256 (67.2)	1370 (79.3)	1294 (99.3)	312 (85.7)	129 (100)	3361 (86.1)
ICU admission, n (%)						
No	238 (62.5)	1566 (90.7)	961 (73.8)	263 (72.2)	0	3028 (77.6)
Yes	143 (37.5)	161 (9.32)	342 (26.2)	101 (27.7)	129 (100)	876 (22.4)
Highest respiratory support, n (%)						
None	0	605 (35.0)	0	0	0	605 (15.5)
Oxygen (mask or cannula)	124 (32.5)	592 (34.3)	854 (65.5)	0	0	1570 (40.2)
Non-invasive ventilation *	6 (1.57)	369 (21.4)	63 (4.83)	0	0	438 (11.2)
Intubation	103 (27.0)	161 (9.32)	298 (22.9)	0	0	562 (14.4)
Not available	148 (38.8)	0	88 (6.75)	364 (100)	129 (100)	729 (18.7)
Number of comorbidities, n (%)						
None	64 (16.8)	865 (50.1)	323 (24.8)	0	36 (27.9)	1322 (33.9)
1	97 (25.5)	522 (30.2)	305 (23.4)	0	48 (37.2)	964 (24.7)
2	72 (18.9)	205 (11.9)	174 (13.4)	0	36 (27.9)	469 (12.0)
3 or more	22 (5.77)	55 (3.18)	54 (4.14)	0	9 (6.98)	132 (3.38)
Not available	126 (33.1)	80 (4.63)	447 (34.3)	364 (100)	0	1013 (26.1)
Comorbidity, n (%)						
Asthma	34 (8.92)	148 (8.57)	91 (6.98)		22 (17.1)	295 (7.56)
Cancer	51 (13.4)	126 (7.30)	154 (11.8)		15 (11.6)	346 (8.86)
Diabetes	89 (23.4)	207 (12.0)	266 (20.4)		35 (27.1)	597 (15.3)
Heart failure	39 (10.2)	160 (9.26)	87 (6.68)		6 (4.65)	292 (7.48)
Hypertension	156 (40.9)	484 (28.0)	554 (42.5)		70 (54.3)	1264 (32.4)
Alive status (at 60 days of follow-up), n (%)						
Alive	335 (87.9)	1622 (93.9)	1147 (88.0)	286 (78.6)	95 (73.6)	3485 (89.3)
Deceased	46 (12.1)	105 (6.08)	156 (12.0)	78 (21.4)	34 (26.4)	419 (10.7)
Series, n (%)						
BelCovid						381 (9.76)
GEN-COVID						1727 (44.2)
Hostage						1303 (33.4)
SPRGX						364 (9.32)
SweCovid						129 (3.33)

*CPAP, BiPAP, or high-flow cannula.

To investigate the factors affecting mortality after SARS-CoV-2 infection, we carried out a survival analysis in a period of 60 days post-infection. Since the hazard proportionality assumption was not verified in our series (global Schoenfeld residuals test *P*-value = 6.5 × 10^−6^) we carried out both a weighted multivariable Cox analysis, to deal with non-proportional hazards, and a multivariable proportional hazard Cox regression. In both models we used the following variables, for which we had full data availability: age, age^2^, sex, and date of infection (Table 2A). The results obtained were similar. Indeed, we observed that the major mortality risk factor for COVID-19 patients was the age at infection (as expected, in both models it was a risk factor for poor prognosis, with HR > 1, with increasing age). We also explored a non-linear effect of age (i.e., age^2^) on survival and, although statistically significant, it was quite irrelevant (HR = 1 in both models). Female sex was associated with a slightly higher probability of survival (HR = 0.73 in both models), compared to males. Additionally, we observed that patients infected in the first pandemic wave (i.e., in the first half of 2020) had lower probabilities of survival than patients infected later (HR = 1.6 and 1.5 in Cox and weighted Cox models, respectively).

**Table 2:** Clinical and personal prognostic factors for COVID-19 patients, as resulted from Cox’s and weighted Cox’s multivariable tests.

	Multivariable Cox’s test		Multivariable weighted Cox’s test	
	HR (95% CI)	*P*-value	HR (95% CI)	*P*-value
A				
Age at diagnosis (linear)	1.20 (1.10–1.30)	2.59 × 10^−5^	1.20 (1.09–1.32)	1.54 × 10^−4^
Age at diagnosis (quadratic)	1.00 (1.00–1.00)	0.0135	1.00 (1.00–1.00)	0.0235
Sex				
Male	1		1	
Female	0.730 (0.596–0.894)	2.38 × 10^−3^	0.733 (0.597–0.902)	3.23 × 10^−3^
Infection before June 30th, 2020				
No	1		1	
Yes	1.55 (1.21–1.99)	6.13 × 10^−4^	1.53 (1.19–1.96)	9.35 × 10^−4^
B				
Age at diagnosis (linear)	1.20 (1.10–1.30)	2.68 × 10^−5^	1.20 (1.10–1.31)	1.32 × 10^−4^
Age at diagnosis (quadratic)	1.00 (1.00–1.00)	0.0159	1.00 (1.00–1.00)	0.025
Sex				
Male	1		1	
Female	0.730 (0.595–0.895)	2.43 × 10^−3^	0.733 (0.595–0.903)	3.56 × 10^−3^
Series				
BelCovid	1		1	
GEN-COVID	0.736 (0.508–1.07)	0.104	0.739 (0.503–1.09)	0.124
Hostage	0.760 (0.547–1.06)	0.103	0.745 (0.529–1.05)	0.0931
SPRGX	1.05 (0.727–1.52)	0.795	1.03 (0.702–1.51)	0.884
SweCovid	2.57 (1.64–4.01)	3.52 × 10^−5^	2.54 (1.62–3.99)	4.79 × 10^−5^
Infection before June 30th, 2020				
No	1		1	
Yes	1.36 (1.01–1.83)	0.0405	1.36 (1.01–1.82)	0.0424

HR, hazard ratio; CI, confidential interval.

We also tested Cox and weighted Cox models with an additional covariate, i.e., the series in which patients were recruited. Indeed, we were aware that some differences in the recruitment might have confounding effects on patient survival. For instance, we knew that SweCovid patients were all admitted in intensive care unit, they probably were affected by a severe form of COVID-19 and, therefore, their probability of survival was lower than other patient series. As expected, SweCOVID patients had the highest risk of death among all patients included in the present study (Table 2B). These results prompted us to include the explored variables as covariates in the genome-wide survival analysis, in order to identify genetic variants that were independent prognostic factors.

We also drew KM curves testing the effects of age, sex, pandemic wave, and patient series, on the risk of death 60 days after infection. Regarding the age, it was discretized in five age groups, starting from patients < 55 years old, then by decades till the age of 84, and the last group comprising patients ≥ 85 years old. Highly significant associations were observed for age, patient series, and pandemic wave (Supplementary Figure S3), whereas the association with sex was weaker, but still significant (log-rank test, *P*-value = 0.03). As already observed with the Cox models, the probability of survival decreased with increasing age and was lower for males than females, for patients infected at the beginning of 2020, and as expected, for SweCOVID patients.

### Germline variants are associated with COVID-19 overall survival 60 days after infection

Genotype data of patients from GEN-COVID Multicenter Study and from the series included in the European Genome-Phenome Archive (EGA) study number EGAS00001005304 (except for BRACOVID and INMUNGEN-CoV, that were unavailable and with missing data, respectively, as explained above) were used for the genome-wide survival analysis. After quality controls and imputation, the dataset comprised 7,151,809 variants and 3904 patients, 91% of which were of European ancestry (Supplementary Figure S2B and C).

**Figure 1:**
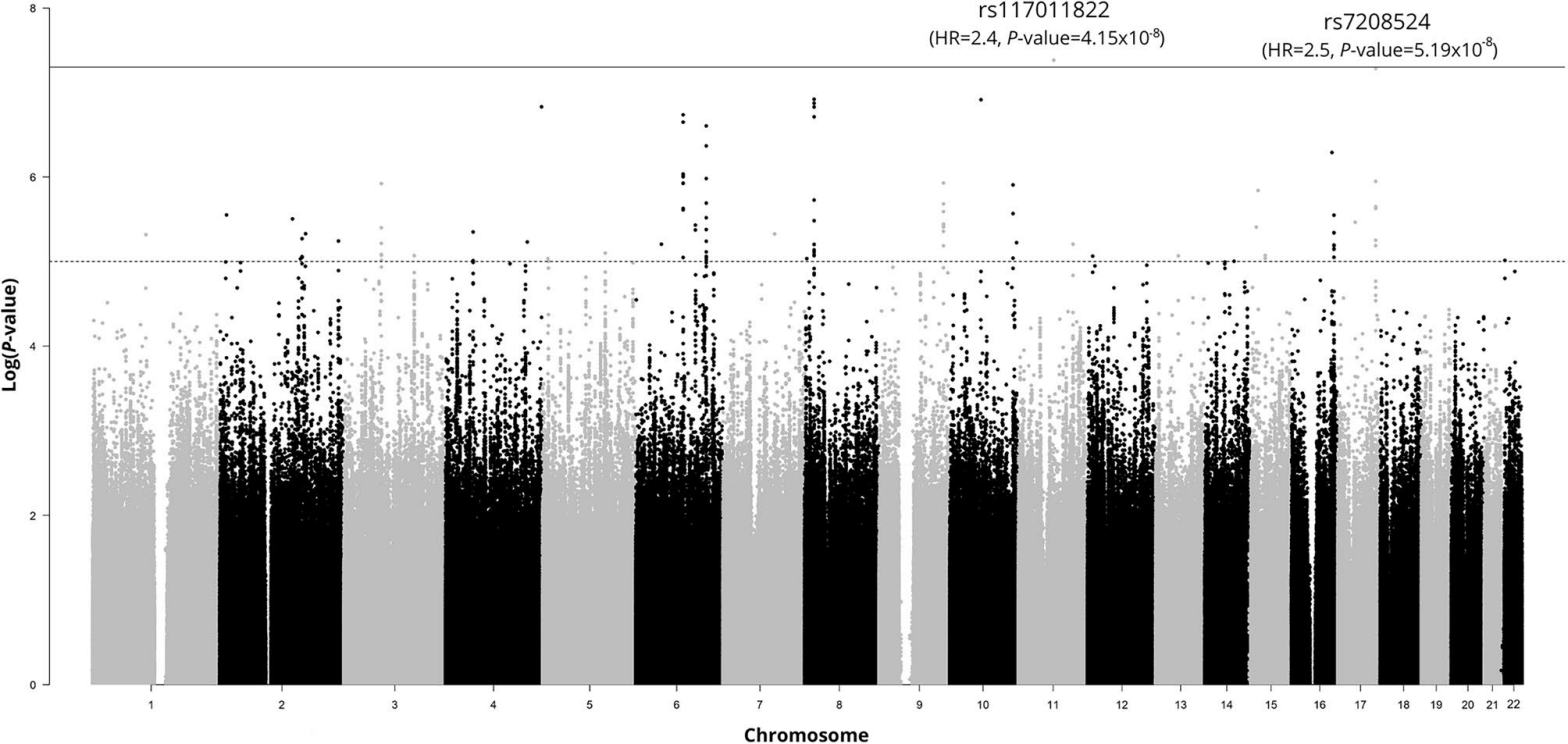
Manhattan plot of the results of the GWAS for survival of COVID-19 patients. SNPs are plotted on the x-axis according to their genomic position (GChr 38, hg38 release), and *P*-values (− log10(*P*)) for their association with survival probability are plotted on the y-axis. The horizontal solid line represents the threshold of significance (*P*-value < 5.0 × 10^−8^), whereas the dashed one represents a suggestive threshold (*P*-value < 1.0 × 10^5^). Names, hazard ratios and *P*-values of the two most significant SNPs are shown on top.

We run a GWAS Cox model, with the first 10 PCs, age, age^2^, sex, period of infection and patient series as covariates and we found one variant (rs117011822) associated with survival at *P*-value < 5.0 × 10^−8^ (genome-wide significance level) and another one (rs7208524) nearly significant (*P*-value = 5.19 × 10^−8^). The results for all the variants tested in the multivariable Cox model are reported in a Manhattan plot (Fig. 1). The minor alleles of both these variants were risk factors for poor prognosis, showing hazard ratios > 1. Both are low frequency variants, in our dataset (MAF < 5%). The top one is a 2 kb upstream variant of *FGF19* gene on chromosome 11, and the other one maps in an intron of the *GPRC5C* gene on chromosome 17.

**Figure 2:**
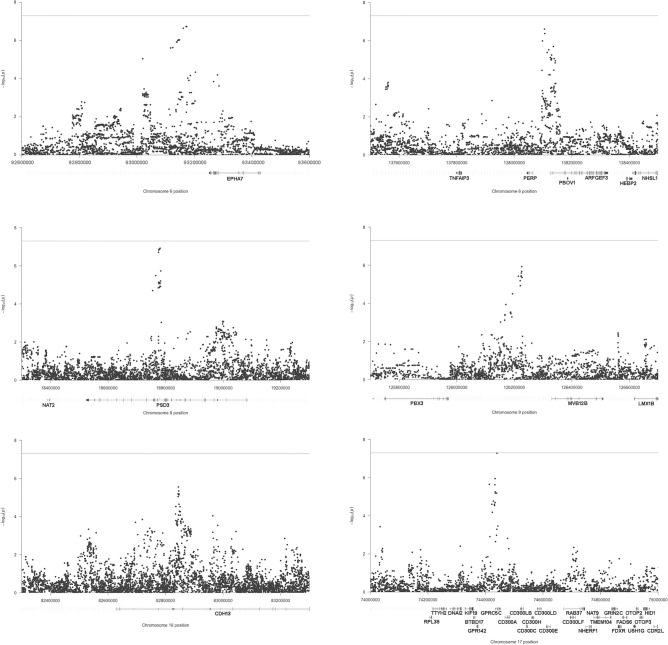
Zoomed plots of six loci associated with patient survival. SNPs are plotted on the x-axis according to their chromosome position (GChr 38, hg38 release), and *P*-values (− log10(*P*)) for their association with survival probability are plotted on the y-axis. The horizontal line represents the threshold of significance (*P*-value < 5.0 × 10^−8^). Below the x axis, the mapped genes are plotted (according to University of California Santa Cruz Genome Browser notation).

Looking at all the variants with a *P*-value < 1.0 × 10^−5^ (n = 113; Supplementary Table S1), we found that 7 variants mapped in the *GPRC5C* gene locus, 16 polymorphisms were on chromosome 8, in the *PSD3* gene locus, 9 SNPs on chromosome 9, in an intergenic region of 359 kb between *PBX3* and *MVB12B* genes, and 9 variants in the *CDH13* locus, on chromosome 16. Additionally, other 27 mapped on chromosome 6, 12 of which were in an intergenic region near the *EPHA7* gene, in proximity of an enhancer region (ENSR00000798782), and 13 mapped in the locus of the *PERP* gene, mostly in its 5’ regulatory region. A zoomed plot for each of these loci is reported in Fig. 2.

**Figure 3:**
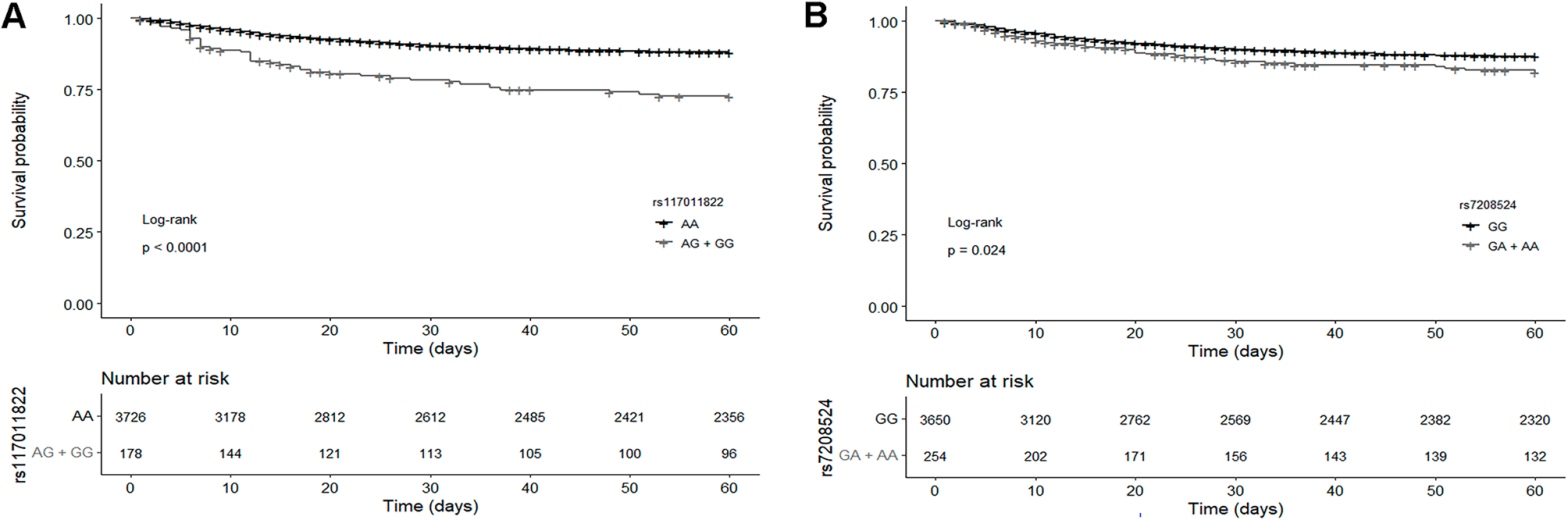
Kaplan–Meier survival curves for COVID-19 patients according to the genotype of the top significant variants (**A**) rs117011822 and (**B**) rs7208524. Black line represents patients homozygous for the major allele and grey line represents patients with at least one copy of the minor allele (according to the dominant model, patients with heterozygous genotype and homozygous for the minor allele were grouped together). Crosses denote censored samples. Numbers of patients at risk are shown below the plot. Log–rank test *P*‐value is shown.

Beside the additive model, we tested the two top-significant variants in a dominant model (using the same covariates), where the survival of patients heterozygous and homozygous for the alternative allele was compared with that of patients homozygous for the common allele. Also this analysis indicated that having at least one alternative allele of these variants conferred a higher risk of poor COVID-19 prognosis than having a wild-type genotype (rs117011822: HR = 2.47, *P*-value = 3.43 × 10^−8^; rs7208524: HR = 2.70, *P*-value = 1.10 × 10^−7^). We plotted the KM curves to visualize the probability of survival according to the genotypes of both variants (Fig. 3).

We also looked for the top-significant variants previously identified by the meta-analysis, reported by the COVID-19 HGI, as associated with COVID-19 severity^9^, in our results. Of note, the phenotypes under investigation in that study were quite different, since the COVID-19 HGI defined the severity phenotype as a binary variable representing COVID-19 patients’ risk of hospitalisation. As shown in Supplementary Table S2, none of the COVID-19 HGI top variants were significant, neither at nominal *P*-value < 0.01, in our survival model.

Finally, we tested the risk of mortality in the 60 days after infection, using a logistic regression model, with the same covariates used in the Cox analysis. In this GWAS, no SNPs reached the genome-wide significance threshold. However, 30% of the 113 top significant SNPs associated with survival probability were confirmed also in this GWAS, as associated with mortality risk, although at a higher *P*-value (< 1.0 × 10^−5^). These results are reported in Supplementary Table S1, for comparison with Cox analysis results.

### Variants associated with COVID-19 patient survival are involved in immune or lung functions

We queried two different eQTL databases, namely GTEX and eQTLGen, to test whether our most significant variants (*P*-value < 1.0 × 10^−5^) were previously reported as eQTLs. In GTEx, 43 out of 113 SNPs were eQTLSs of 33 target genes, in several different tissues (e.g., brain, lung, muscle, heart, spleen, whole blood) and in eQTLGen 54 out of 113 SNPs acted as *cis* eQTLs for 30 genes, in the whole blood (Supplementary Table S3 and S4). Eight target genes (i.e., *TSPYL1*, *EPHB4*, *MOSPD3*, *UFSP1*, *GIGYF1*, *SLC12A9*, *MVB12B*, and *KLRC1*) were found in both databases. The list of the 55 unique eQTL target genes was enriched for the Reactome pathway R-HSA-198933: Immunoregulatory interactions between a Lymphoid and a non-Lymphoid cell (enrichment ratio = 16.7; FDR = 0.017), including the CD300 genes (A, C, and LB) and two other genes (*KLRC1* and *KLRF1*).

In addition, we explored the possibility that the quite numerous SNPs identified on chromosome 6 and 8, next to *EPHA7* and *PSD3* genes, respectively, might be in LD with some near variants reported as eQTLs of these two genes. Indeed, none of these SNPs were reported as eQTLs in the two searched databases. Looking at data available in the European population (using LDlink), we found several variants in strong LD (D’ > 0.8) with our SNPs and acting as *EPHA7* and *PSD3* eQTLs, in many tissues (Supplementary Table S5).

Considering the list of 38 genes (Supplementary Table S6) mapping within 50 kbps of the 113 top significant variants, functional annotation analysis with DAVID identified 19 significant terms (FDR < 0.05, Supplementary Table S7). Among these, the top significant ones are two Gene Ontology (GO) biological processes, namely, “positive regulation of natural killer (NK) cell mediated cytotoxicity” and “stimulatory C-type lectin receptor signalling pathway”, both involving the same five genes (*KLRK1*, *KLRC3*, *KLRC4*, *KLRC4-KLRK1*, and *KLRD1*). Three of these genes (*KLRC3*, *KLRC4*, and *KLRD1*) were also annotated in the Biocarta “h_nkcellsPathway: Ras-Independent pathway in NK cell-mediated cytotoxicity”, the only pathway that reached the statistical significance threshold.

Partially overlapping results were obtained in the overrepresentation analysis carried out with the FUMA platform (Supplementary Table S8), that identified 36 significantly enriched functional gene sets. Among them, we observed GO biological processes, KEGG and Biocarta pathways related to NK cell regulation and other immune functions, in which approximately the same genes as above are involved. In addition, looking at tissue specificity analysis by FUMA, we observed that the 38-gene list was enriched of genes over-expressed in the lung and in the brain’s putamen basal ganglia (Supplementary Figure S4).

## Discussion

In this study we investigated the effects of host germline variants on the overall survival of COVID-19 patients, 60 days after the infection. With a case-only approach and using a multivariable Cox model to look for variants associated with the probability of survival after infection, we aimed to dissect the genetics bases to develop a severe COVID-19 from a different point of view, as compared to several other genetic studies on COVID-19 severity (reviewed in^10^). The analysis considered, as covariate of the multivariable model, the most widely known prognostic factors for COVID-19 survival, i.e., patient age at infection, sex, and period of infection (at the very beginning of the pandemic or after).

We identified a genome-wide level significant association between survival and the SNP rs117011822, on chromosome 11. We observed that individuals with an increasing number of minor alleles of this variant in their genotype (both under additive and dominant model) had a worse prognosis than patients homozygous for the major allele, 60 days after SARS-CoV-2 infection. This variant maps in a regulatory region (ENSR00000958007), specifically a CTCF binding site, upstream the *FGF19* gene. However, we did not find evidence for a regulation of *FGF19* expression by this variant in GTEx or eQTLGen, but it might be interesting to investigate this aspect further. Indeed, it was reported that serum levels of the FGF19 protein were lower in asymptomatic than symptomatic COVID-19 patients^35^. In that study, the authors discussed a possible role of FGF19 (together with other proteins) in lung tissue repair, with differences between asymptomatic and symptomatic patients. Therefore, it might be interesting to further investigate the role of the minor allele of rs117011822 in the regulation on *FGF19* levels with the aim to understand the functional mechanism underlying the statistical association observed in our study.

Additional loci on chromosomes 17, 8, 6, 9, and 16 were suggestively associated with COVID-19 patient overall survival. On chromosome 17 we identified a locus of seven variants that were reported to act as regulators of the expression of CD300 genes (*CD300A*, *CD300C*, and *CD300LB*). Of note, CD300 is a family of leukocyte surface proteins involved in immune response signalling pathways and it has been observed that shifts in the expression pattern of CD300 molecules in T-cells of COVID-19 patients correlated with COVID-19 severity^36^.

The 16 variants on chromosome 8 mapped in intronic regions of the *PSD3* gene, also known as *EFA6R*, a member of the family of guanine nucleotide exchange factors, that activate ADP-ribosylation factor 6 (ARF6)^37^. This protein is involved in endocytosis, and it has been reported to play a role in SARS-CoV-2 cell entry^38,39^. So far, it has not yet been reported any role of *PSD3* variants in the regulation of ARF6 activation. Our variants showed a strong LD with eQTL SNPs of *PSD3*.

The six variants we found associated with COVID-19 patient survival, mapping in an intergenic region of chromosome 9, were reported to be eQTLs of the near the *MVB12B* gene. No evidence for a role of this gene in COVID-19 is available, but it is interesting to underline that it codes for a subunit of the ESCRT-I complex that mediates HIV virus budding^40^.

The 12 intergenic variants on chromosome 6 (at position 93 Mb) were near the *EPHA7* gene, which was suggested to be a downstream mediator of cytokine production, induced by the N-terminal domain of the SARS-CoV-2 spike protein^41^. These variants mapped in an enhancer region and might affect the expression of the *EPHA7* gene, although we did not find them among the eQTLs of this gene. However, these variants are in strong LD with eQTL SNPs of *EPHA7*. The other 11 variants on chromosome 6, instead, mapped in the *PERP* gene locus and some of them were already annotated as eQTLs of this gene. Although no functions related to COVID-19 have been reported for the *PERP* gene, so far, it encodes a protein that is a p53 apoptosis effector, and, recently, Wang and colleagues reviewed the possible roles of p53 in mediating host-virus interactions in infections caused by Coronaviruses^42^.

Finally, the nine SNPs on chromosome 16, intronic to the *CDH13* gene, were eQTLs of this gene, which encodes the T-cadherin protein, a regulator of vascular permeability, but also receptor of adiponectin and LDL. It is involved in lung function, and reportedly associated with several metabolic disorders as atherosclerosis, dyslipidemia, obesity, and also diabetes (as reviewed in^43^).

Interestingly, the list of genes, whose expression was reported to be regulated by our top-significant variants, was enriched for genes with immunoregulatory functions. These included the already mentioned CD300 genes, but also *KLRC1* and *KLRF1* genes. *KLRC1* (alias *NKG2A*) expression, in SARS-CoV-2 infected patients was suggested to correlate with functional exhaustion of cytotoxic lymphocytes and with a severe COVID-19 outcome^44^. In addition, functional annotation of the genes near or where top-significant variants mapped resulted in an enrichment of immune-related terms and pathways, in particular those involved in regulation of NK cell-mediated cytotoxicity. This finding is interesting in the light of previous results by Maucorant et al.^45^ showing that distinct NK-immunotypes were related to COVID-19 severity. Among the genes in this pathway, there is *KLRK1*, also known as *NKG2D*, coding for a NK cell activating receptor. It has been previously reported that SARS-CoV-2 non-structural protein 1 can downregulate ligands of the NKG2D receptor, thus escaping NK cells cytotoxicity^46^.

Regarding the findings from FUMA tissue specificity analysis, the observed enrichment of our gene list in genes whose expression is altered in lung is not unexpected: as the major clinical manifestation of SARS-CoV-2 infection and severity is at respiratory level, we expected that variants associated with COVID-19 survival were in genes expressed in the lungs. On the other hand, it is more difficult to speculate on the finding of an enrichment of genes upregulated in brain putamen basal ganglia. In a recent paper^47^, Balsak et al. reported that basal ganglia can be damaged after COVID-19, due to microstructural alterations caused by hypoxia. However, further studies are needed to understand if the variants/genes identified in our study might be involved in the hypoxia induced brain alterations after SARS-CoV-2 infection and, also, if this kind of damage might affect COVID-19 patient survival.

Despite the above cited evidence found in the literature, without appropriate functional studies, we cannot assert that the variants we identified as associated with survival play a role in predisposing patients to a worse COVID-19 outcome. However, we believe that our findings are worthy of further investigation and are of interest, since we explored the genetics of COVID-19 severity with an unconventional approach that might have led to new results. Indeed, not surprisingly, none of the previously reported variants associated with severity in^9^ were significant in our study. Indeed, both the phenotype (severity vs. overall survival 60 days after the infection) and the analysis model (regression vs. Cox) were completely different.

We are aware of some limitations in our study. First, validation in an independent, wider, but possibly genetically homogeneous patient series should be needed. Indeed, we were able to detect only one variant associated with survival at a genome-wide significance level. In addition, our series, is mainly composed of patients of European ancestry and our results, although controlled for population stratification, would not be directly generalizable to patients from different ethnicities. Additionally, since we did not adjust for patients’ comorbidities (as no full data were available), we cannot exclude that some of the identified associations might result from such potential confounding factors. For instance, the variants in the *CDH13* gene, involved in metabolic disorders, might be this case. Although we were aware of this limitation, we preferred not to further reduce the already relatively small sample size of our analysis, by excluding patients with unavailable comorbidity data, to avoid losing statistical power.

Overall, our results shed new light on the genetics of COVID-19 severity, having identified some loci associated with patient survival, at 60 days after infection. Although our findings suggest that genetics plays a limited role in affecting mortality probability after SARS-CoV-2 infection, the identified variants are worthy to be further investigated as possible prognostic factors for COVID-19.

### Supplementary Information

Supplementary Information 1.

Supplementary Figures.

Supplementary Table S1.

Supplementary Table S2.

Supplementary Table S3.

Supplementary Table S4.

Supplementary Table S5.

Supplementary Table S6.

Supplementary Table S7.

Supplementary Table S8.

## Data Availability

Genotype data that support the findings of this study are available at The European Genome-phenome Archive (EGA) (study number: EGAS00001005304) and upon request to Prof. Renieri at University of Siena.

## Code availability

No custom software or algorithm were used. Packages and tools used for the analyses described in the manuscript are all publicly available. Anyway, used scripts are available from the corresponding author upon request.

## References

[1] Long QX, Tang XJ, Shi QL (2020). Clinical and immunological assessment of asymptomatic SARS-CoV-2 infections. Nat. Med..

[2] Guan W, Ni Z, Hu Y (2020). Clinical Characteristics of Coronavirus Disease 2019 in China. N. Engl. J. Med..

[3] White-Dzuro G, Gibson LE, Zazzeron L (2020). Multisystem effects of COVID-19: A concise review for practitioners. Postgrad. Med..

[4] Michelozzi P, De’Donato F, Scortichini M (2020). Temporal dynamics in total excess mortality and COVID-19 deaths in Italian cities. BMC Public Health.

[5] Rostami A, Sepidarkish M, Leeflang MMG (2021). SARS-CoV-2 seroprevalence worldwide: A systematic review and meta-analysis. Clin. Microbiol. Infect..

[6] Elliott J, Bodinier B, Whitaker M (2021). COVID-19 mortality in the UK Biobank cohort: Revisiting and evaluating risk factors. Eur. J. Epidemiol..

[7] Minnai F, De Bellis G, Dragani TA, Colombo F (2022). COVID-19 mortality in Italy varies by patient age, sex and pandemic wave. Sci. Rep..

[8] Onoja A, Picchiotti N, Fallerini C (2022). An explainable model of host genetic interactions linked to COVID-19 severity. Commun. Biol..

[9] Pathak GA, Karjalainen J, Stevens C (2022). A first update on mapping the human genetic architecture of COVID-19. Nature.

[10] Cappadona C, Rimoldi V, Paraboschi EM, Asselta R (2023). Genetic susceptibility to severe COVID-19. Infect. Genet. Evol..

[11] Lehrer S, Rheinstein PH (2021). ABO blood groups, COVID-19 infection and mortality. Blood Cells Mol. Dis..

[12] Fricke-Galindo I, Martínez-Morales A, Chávez-Galán L (2022). IFNAR2 relevance in the clinical outcome of individuals with severe COVID-19. Front. Immunol..

[13] Hu J, Li C, Wang S, Li T, Zhang H (2021). Genetic variants are identified to increase risk of COVID-19 related mortality from UK Biobank data. Hum. Genom..

[14] de Andrade CC, Silva ATP, Vasconcelos LRS (2022). A polymorphism in the TMPRSS2 gene increases the risk of death in older patients hospitalized with COVID-19. Viruses.

[15] Clark TG, Bradburn MJ, Love SB, Altman DG (2003). Survival analysis part I: Basic concepts and first analyses. Br. J. Cancer.

[16] Therneau TM, Grambsch PM (2000). Modeling Survival Data: Extending the Cox Model.

[17] Schemper M (1992). Cox analysis of survival data with non-proportional hazard functions. The Statistician.

[18] Dunkler D, Ploner M, Schemper M, Heinze G (2018). Weighted cox regression using the R package coxphw. J. Stat. Softw..

[19] Chang CC, Chow CC, Tellier LCAM, Vattikuti S, Purcell SM, Lee JJ (2015). Second-generation PLINK: Rising to the challenge of larger and richer datasets. Gigascience.

[20] Price AL, Weale ME, Patterson N (2008). Long-range LD can confound genome scans in admixed populations. Am. J. Hum. Genet..

[21] Delaneau O, Marchini J, McVean GA (2014). Integrating sequence and array data to create an improved 1000 Genomes Project haplotype reference panel. Nat. Commun..

[22] Das S, Forer L, Schönherr S (2016). Next-generation genotype imputation service and methods. Nat. Genet..

[23] Loh PR, Danecek P, Palamara PF (2016). Reference-based phasing using the haplotype reference consortium panel. Nat. Genet..

[24] Fuchsberger C, Abecasis GR, Hinds DA (2015). minimac2: Faster genotype imputation. Bioinformatics.

[25] Taliun D, Harris DN, Kessler MD (2021). Sequencing of 53,831 diverse genomes from the NHLBI TOPMed program. Nature.

[26] Verlouw JAM, Clemens E, de Vries JH (2021). A comparison of genotyping arrays. Eur. J. Hum. Genet..

[27] Aulchenko YS, Ripke S, Isaacs A, van Duijn CM (2007). GenABEL: An R library for genome-wide association analysis. Bioinformatics.

[28] Benjamini Y, Hochberg Y (1995). Controlling the false discovery rate: A practical and powerful approach to multiple testing. J. R. Stat. Soc. Ser. B (Methodol.).

[29] Võsa U, Claringbould A, Westra H-J (2021). Large-scale cis- and trans-eQTL analyses identify thousands of genetic loci and polygenic scores that regulate blood gene expression. Nat. Genet..

[30] Machiela MJ, Chanock SJ (2015). LDlink: A web-based application for exploring population-specific haplotype structure and linking correlated alleles of possible functional variants. Bioinformatics.

[31] Wang J, Vasaikar S, Shi Z, Greer M, Zhang B (2017). WebGestalt 2017: A more comprehensive, powerful, flexible and interactive gene set enrichment analysis toolkit. Nucleic Acids Res..

[32] Durinck S, Spellman PT, Birney E, Huber W (2009). Mapping identifiers for the integration of genomic datasets with the R/Bioconductor package biomaRt. Nat. Protoc..

[33] Huang DW, Sherman BT, Tan Q (2007). DAVID bioinformatics resources: Expanded annotation database and novel algorithms to better extract biology from large gene lists. Nucleic Acids Res..

[34] Watanabe K, Taskesen E, van Bochoven A, Posthuma D (2017). Functional mapping and annotation of genetic associations with FUMA. Nat. Commun..

[35] Soares-Schanoski A, Sauerwald N, Goforth CW (2022). Asymptomatic SARS-CoV-2 infection is associated with higher levels of serum IL-17C, matrix metalloproteinase 10 and fibroblast growth factors than mild symptomatic COVID-19. Front. Immunol..

[36] Zenarruzabeitia O, Astarloa-Pando G, Terrén I (2021). T cell activation, highly armed cytotoxic cells and a shift in monocytes CD300 receptors expression is characteristic of patients with severe COVID-19. Front. Immunol..

[37] Kanamarlapudi V (2014). Exchange factor EFA6R requires C-terminal targeting to the plasma membrane to promote cytoskeletal rearrangement through the activation of ADP-ribosylation factor 6 (ARF6). J. Biol. Chem..

[38] Zhou Y-Q, Wang K, Wang X-Y (2022). SARS-CoV-2 pseudovirus enters the host cells through spike protein-CD147 in an Arf6-dependent manner. Emerg. Microbes Infect..

[39] Mirabelli C, Bragazzi Cunha J, Wotring JW (2023). ARF6 is a host factor for SARS-CoV-2 infection in vitro. J. Gener. Virol..

[40] Morita E, Sandrin V, Alam SL, Eckert DM, Gygi SP, Sundquist WI (2007). Identification of human MVB12 proteins as ESCRT-I subunits that function in HIV budding. Cell Host. Microbe.

[41] Chan M, Vijay S, McNevin J, McElrath MJ, Holland EC, Gujral TS (2021). Machine learning identifies molecular regulators and therapeutics for targeting SARS-CoV2-induced cytokine release. Mol. Syst. Biol..

[42] Wang X, Liu Y, Li K, Hao Z (2023). Roles of p53-mediated host-virus interaction in coronavirus infection. Int. J. Mol. Sci..

[43] Rubina KA, Semina EV, Kalinina NI, Sysoeva VYu, Balatskiy AV, Tkachuk VA (2021). Revisiting the multiple roles of T-cadherin in health and disease. Eur. J. Cell Biol..

[44] Zheng M, Gao Y, Wang G (2020). Functional exhaustion of antiviral lymphocytes in COVID-19 patients. Cell Mol. Immunol..

[45] Maucourant C, Filipovic I, Ponzetta A (2020). Natural killer cell immunotypes related to COVID-19 disease severity. Sci. Immunol..

[46] Lee MJ, Leong MW, Rustagi A (2022). SARS-CoV-2 escapes direct NK cell killing through Nsp1-mediated downregulation of ligands for NKG2D. Cell Rep..

[47] Balsak S, Atasoy B, Donmez Z (2023). Microstructural alterations in hypoxia-related BRAIN centers after COVID-19 by using DTI: A preliminary study. J. Clin. Ultrasound.

