# Supplementary material for: A genome-wide association study for survival from a multi-centre European study identified variants associated with COVID-19 risk of death
Source: Sci Rep. 2024 Feb 6;14:3000. doi: 10.1038/s41598-024-53310-x (PMC10847137; doi:10.1038/s41598-024-53310-x)
Supplement: Supplementary file 2 — Supplementary Figures. [file 41598_2024_53310_MOESM2_ESM.pdf]

Supplementary Figures

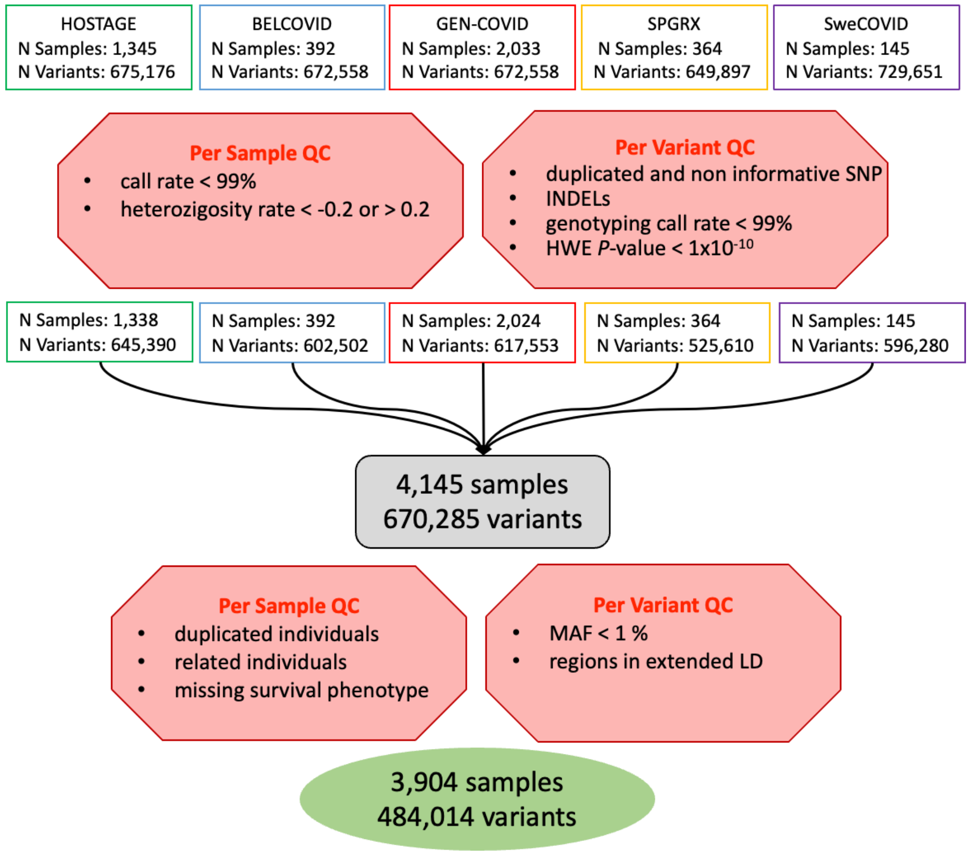

Supplementary Figure S1. Pre-imputation QC steps of genotyping data.

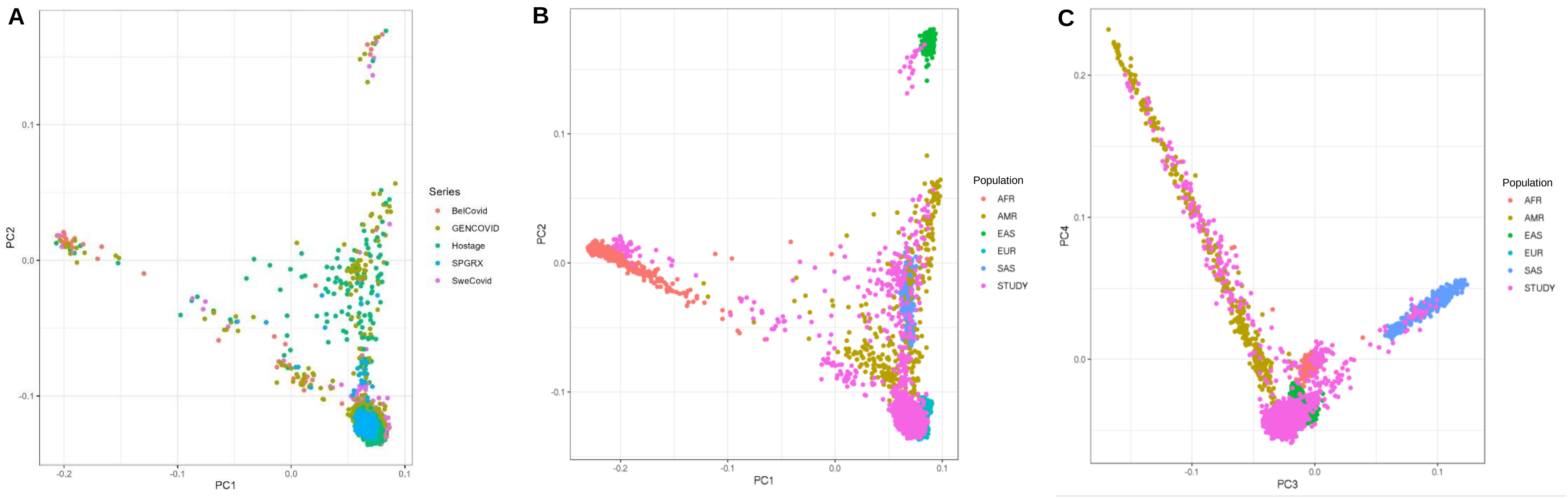

Supplementary Figure S2. (**A**) PC plot of the first two PCs of our dataset (n=3,904). Dots are coloured according to the five patient series (BelCovid, GENCOVID, Hostage, SPGRX, and SweCovid). PC1 and PC2 (**B**) and PC3 and PC4 (**C**) of our dataset (STUDY, pink dots, n=3,904) were plotted along with the same PCs of 2,504 individuals from five populations from the 1000 Genome Project (AFR, Africans; AMR, Americans; EAS, East Asians; EUR, Europeans; SAS, South Asians).

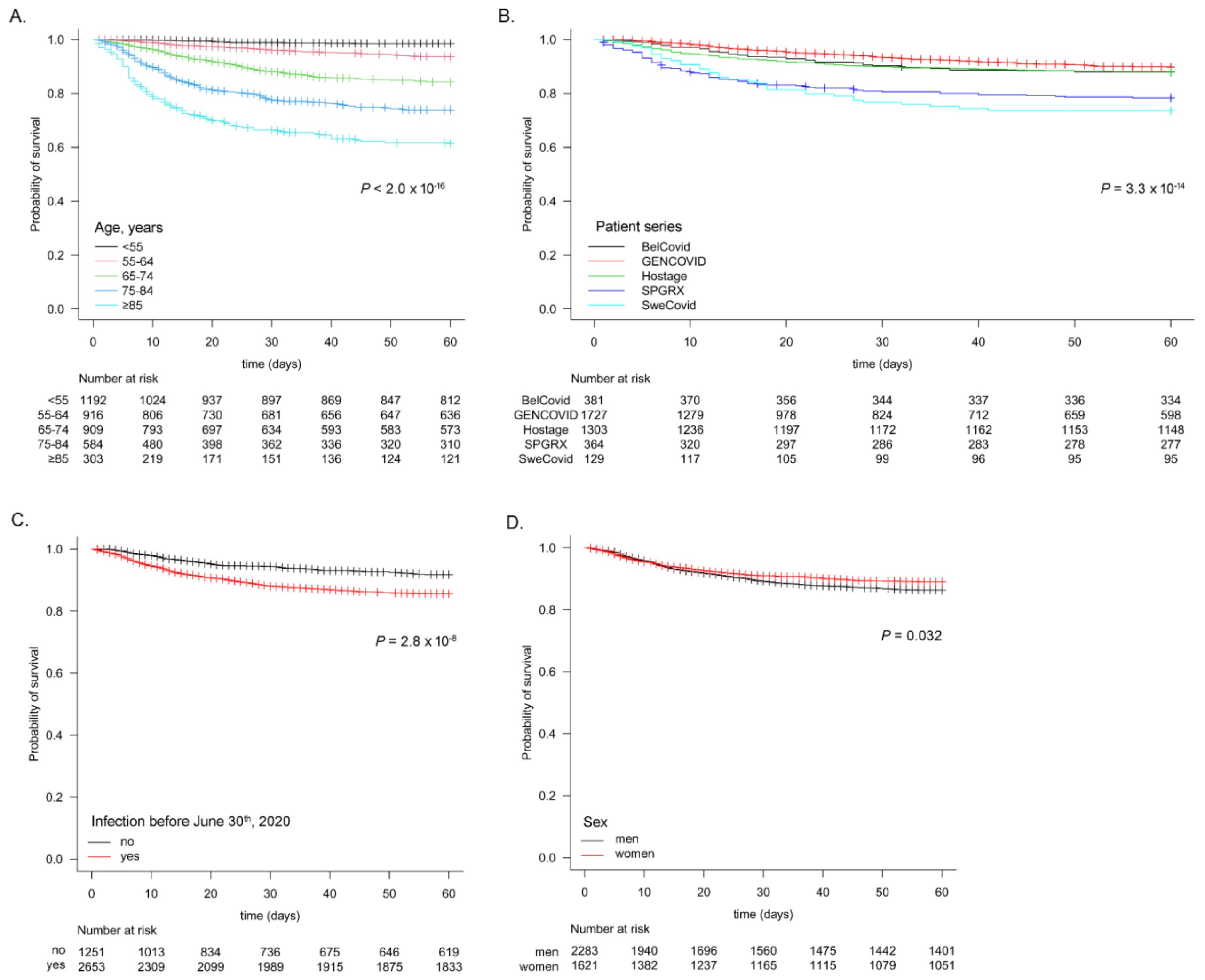

Supplementary Figure S3. KM curves of patient survival, according to age (A), patient series (B), date of infection (C), and sex (D). Crosses denote censored samples. Below each plot are indicated the number of patients at risk in the groups. Log rank *P*-values are shown.

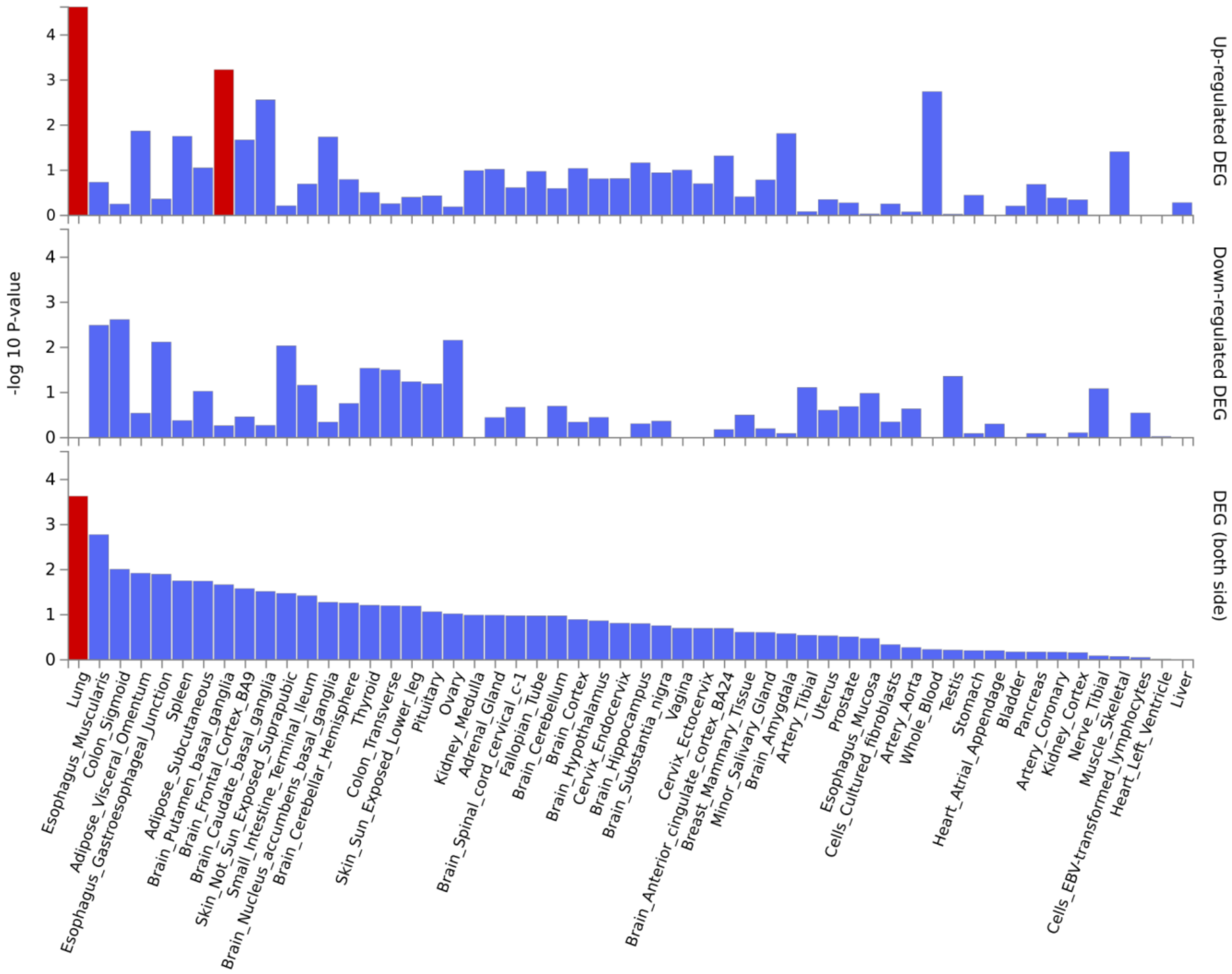

Supplementary Figure S4. Differentially expressed gene sets from FUMA analysis. Significant enrichments at Bonferroni corrected *P*-value ≤ 0.05 are coloured in red.
