# Supplementary material for: A genome-wide association study for survival from a multi-centre European study identified variants associated with COVID-19 risk of death
Source: Sci Rep. 2024 Feb 6;14:3000. doi: 10.1038/s41598-024-53310-x (PMC10847137; doi:10.1038/s41598-024-53310-x)
Supplement: Supplementary file 3 — Supplementary Table S1. [file 41598_2024_53310_MOESM3_ESM.pdf]

Supplementary Table S1. Results of the multivariable Cox model for the 113 top-significant variants associated with COVID-19 survival (*P*- value < 1.0×10^{-5}) and of the logistic regression.

					Cox model			logistic regression			
**Variant**	**rsID**	**Chr**	**Position (bp) ***	**MAF**	**HR**	***P*-value**	**FDR**	**OR**	***P*-value**	**Variant type**	**Gene symbol (distance) §**
chr11:69705448:A:G	rs117011822	11	69,705,448	0.02	2.4	4.15×10^{-8}	0.1	2.7	1.31×10^{-6}	upstream	FGF19 (1426)
chr17:74439635:G:A	rs7208524	17	74,439,635	0.04	2.5	5.19×10^{-8}	0.1	2.6	4.14×10^{-6}	intronic	GPRC5C
chr8:18781150:T:C	rs12677280	8	18,781,150	0.04	2.2	1.20×10^{-7}	0.1	2.6	3.01×10^{-7}	intronic	PSD3
chr10:61229281:C:T	rs7092588	10	61,229,281	0.05	1.9	1.22×10^{-7}	0.1	2.2	8.27×10^{-7}	intergenic	TMEM26 (177362)
chr8:18777746:G:C	rs7836892	8	18,777,746	0.04	2.2	1.33×10^{-7}	0.1	2.6	3.40×10^{-7}	intronic	PSD3
chr8:18778068:A:G	rs4921959	8	18,778,068	0.04	2.2	1.33×10^{-7}	0.1	2.6	3.40×10^{-7}	intronic	PSD3
chr8:18778934:T:C	rs7830088	8	18,778,934	0.04	2.2	1.33×10^{-7}	0.1	2.6	3.40×10^{-7}	intronic	PSD3
chr4:187830740:A:G	rs114906209	4	187,830,740	0.02	2.6	1.47×10^{-7}	0.1	2.8	6.42×10^{-6}	intergenic	ZFP42 (165031)
chr8:18776372:G:A	rs112738876	8	18,776,372	0.04	2.2	1.47×10^{-7}	0.1	2.6	2.43×10^{-7}	intronic	PSD3
chr6:93171196:C:A	rs16870964	6	93,171,196	0.06	1.9	1.83×10^{-7}	0.1	1.9	5.88×10^{-6}	intergenic	EPHA7 (68824)
chr6:93173205:G:A	rs76462658	6	93,173,205	0.06	1.9	1.83×10^{-7}	0.1	1.9	5.88×10^{-6}	intergenic	EPHA7 (66815)
chr8:18775266:T:C	rs7814472	8	18,775,266	0.04	2.2	1.94×10^{-7}	0.1	2.6	3.29×10^{-7}	intronic	PSD3
chr6:93161760:A:G	rs76037606	6	93,161,760	0.06	1.9	2.24×10^{-7}	0.1	1.9	8.08×10^{-6}	intergenic	EPHA7 (78260)
chr6:138105000:G:A	rs72984647	6	138,105,000	0.15	1.6	2.48×10^{-7}	0.1	1.6	3.61×10^{-6}	intronic	PERP
chr6:138106052:A:G	rs72984652	6	138,106,052	0.14	1.6	4.28×10^{-7}	0.2	1.6	4.28×10^{-6}	intronic	PERP
chr16:78966872:C:G	rs74464049	16	78,966,872	0.08	1.7	5.12×10^{-7}	0.2	1.9	4.84×10^{-6}	intronic	WWOX
chr6:93148458:C:T	rs75841618	6	93,148,458	0.14	1.6	9.22×10^{-7}	0.3	1.6	2.36×10^{-5}	intergenic	EPHA7 (91562)
chr6:93143986:G:C	rs16870897	6	93,143,986	0.14	1.6	9.25×10^{-7}	0.3	1.6	2.54×10^{-5}	intergenic	EPHA7 (96034)
chr6:93146602:T:C	rs6927004	6	93,146,602	0.14	1.6	9.36×10^{-7}	0.3	1.6	2.56×10^{-5}	intergenic	EPHA7 (93418)
chr6:93142560:G:A	rs16880170	6	93,142,560	0.35	1.5	9.89×10^{-7}	0.3	1.6	3.04×10^{-5}	intergenic	EPHA7 (97460)
chr6:138098070:A:G	rs2327871	6	138,098,070	0.25	1.4	1.04×10^{-6}	0.3	1.5	2.15×10^{-6}	intronic	PERP
chr17:74433282:A:C	rs60879525	17	74,433,282	0.04	2.3	1.12×10^{-6}	0.3	2.4	3.72×10^{-5}	intronic	GPRC5C
chr9:126226220:T:C	rs944221	9	126,226,220	0.15	1.5	1.17×10^{-6}	0.3	1.6	6.40×10^{-6}	intergenic	MVB12B (100609)
chr6:93138225:A:G	rs16870893	6	93,138,225	0.15	1.5	1.18×10^{-6}	0.3	1.5	3.59×10^{-5}	intergenic	EPHA7 (101795)
chr6:93139667:A:G	rs17540607	6	93,139,667	0.15	1.5	1.19×10^{-6}	0.3	1.6	3.32×10^{-5}	regulatory region	EPHA7 (100353)
chr3:73184232:C:T	rs112192337	3	73,184,232	0.12	1.6	1.19×10^{-6}	0.3	1.6	2.22×10^{-5}	intergenic	PPP4R2 (115034)
chr10:123527896:A:G	rs705173	10	123,527,896	0.08	1.7	1.24×10^{-6}	0.3	1.8	6.15×10^{-6}	intronic	GPR26 (138459)
chr15:36958633:T:C	rs62002421	15	36,958,633	0.17	1.5	1.45×10^{-6}	0.4	1.5	4.78×10^{-5}	intronic	MEIS2
chr8:18783281:G:A	rs7839578	8	18,783,281	0.05	2.1	1.86×10^{-6}	0.5	2.4	2.38×10^{-6}	intronic	PSD3

chr6:138135822:A:G	rs55808177	6	138,135,822	0.15	1.5	2.04×10^{-6}	0.5	1.6	2.85×10^{-5}	intergenic	ARFGEF3 (26117)
chr9:126225879:C:A	rs1888160	9	126,225,879	0.16	1.5	2.07×10^{-6}	0.5	1.5	1.13×10^{-5}	intergenic	MVB12B (100950)
chr17:74413200:C:T	rs73348353	17	74,413,200	0.03	2.5	2.24×10^{-6}	0.5	2.5	6.77×10^{-5}	intergenic	GPRC5C (11651)
chr6:93125192:T:A	rs1575539	6	93,125,192	0.15	1.5	2.35×10^{-6}	0.5	1.5	6.98×10^{-5}	intergenic	EPHA7 (114828)
chr17:74432607:A:G	rs57063902	17	74,432,607	0.04	2.2	2.36×10^{-6}	0.5	2.3	7.46×10^{-5}	intronic	GPRC5C
chr6:93117543:C:G	rs72928570	6	93,117,543	0.15	1.5	2.44×10^{-6}	0.5	1.5	8.80×10^{-5}	Regulatory region	EPHA7 (122477)
chr9:126224480:C:T	rs2248934	9	126,224,480	0.17	1.5	2.55×10^{-6}	0.5	1.5	1.36×10^{-5}	intergenic	MVB12B (102349)
chr10:123525316:T:C	rs841018	10	123,525,316	0.11	1.6	2.70×10^{-6}	0.5	1.7	1.66×10^{-5}	intronic	GPR26 (141039)
chr2:13260969:T:C	rs34888710	2	13,260,969	0.04	1.9	2.80×10^{-6}	0.5	2.3	9.19×10^{-7}	intronic non-coding transcript	TRIB2 (518235)
chr16:82844550:G:A	rs62035176	16	82,844,550	0.42	1.4	2.82×10^{-6}	0.5	1.5	5.86×10^{-6}	intronic	CDH13
chr6:138119910:G:A	rs12528432	6	138,119,910	0.15	1.5	3.03×10^{-6}	0.6	1.6	2.50×10^{-5}	intergenic	PERP (12491)
chr2:142185171:G:A	rs115036660	2	142,185,171	0.03	2.1	3.13×10^{-6}	0.6	2.2	4.01×10^{-5}	Regulatory region	LRP1B (54155)
chr8:18765697:T:C	rs111241636	8	18,765,697	0.02	2.3	3.27×10^{-6}	0.6	2.9	4.26×10^{-6}	intronic	PSD3
chr17:34598270:G:A	rs1029742	17	34,598,270	0.27	1.4	3.41×10^{-6}	0.6	1.6	3.60×10^{-7}	intronic	TMEM132E
chr9:126223762:G:A	rs2809426	9	126,223,762	0.18	1.5	3.58×10^{-6}	0.6	1.5	2.21×10^{-5}	intergenic	MVB12B (103067)
chr6:116786541:G:C	rs76096820	6	116,786,541	0.03	2.1	3.70×10^{-6}	0.6	2.4	9.34×10^{-6}	intergenic	GPRC6A (5544)
chr9:126223597:G:T	rs2809425	9	126,223,597	0.18	1.5	3.71×10^{-6}	0.6	1.5	2.29×10^{-5}	intergenic	MVB12B (103232)
chr9:126214010:G:A	rs2026810	9	126,214,010	0.16	1.5	3.75×10^{-6}	0.6	1.5	2.52×10^{-5}	intergenic	MVB12B (112819)
chr15:33242123:C:T	rs16953757	15	33,242,123	0.15	1.5	3.90×10^{-6}	0.6	1.6	4.15×10^{-6}	intronic	FMN1 (47390)
chr9:126224050:C:G	rs2773391	9	126,224,050	0.18	1.5	3.91×10^{-6}	0.6	1.5	2.39×10^{-5}	intergenic	MVB12B (102779)
chr3:73180132:C:G	rs111805058	3	73,180,132	0.12	1.6	3.98×10^{-6}	0.6	1.6	5.40×10^{-5}	Regulatory region	PPP4R2 (110934)
chr6:138114271:C:G	rs12663855	6	138,114,271	0.15	1.5	4.19×10^{-6}	0.6	1.6	3.03×10^{-5}	intergenic	PERP (6852)
chr6:116851088:G:T	rs139799434	6	116,851,088	0.02	2.1	4.23×10^{-6}	0.6	2.5	9.98×10^{-6}	intergenic	GPRC6A (22051)
chr9:126227214:G:A	rs881355	9	126,227,214	0.16	1.5	4.38×10^{-6}	0.6	1.5	1.99×10^{-5}	intergenic	MVB12B (99615)
chr4:54478531:T:A	rs13121274	4	54,478,531	0.12	1.5	4.47×10^{-6}	0.6	1.7	6.31×10^{-6}	intergenic	KIT (179426)
chr16:82845653:T:A	rs4783291	16	82,845,653	0.41	1.4	4.54×10^{-6}	0.6	1.4	9.54×10^{-6}	intronic	CDH13
chr16:82845739:A:T	rs4782734	16	82,845,739	0.41	1.4	4.58×10^{-6}	0.6	1.4	9.73×10^{-6}	intronic	CDH13
chr2:167468987:T:C	rs78286703	2	167,468,987	0.03	2.1	4.69×10^{-6}	0.6	2.4	1.89×10^{-5}	intronic	B3GALT1
chr7:100772252:T:C	rs314296	7	100,772,252	0.37	0.7	4.70×10^{-6}	0.6	0.7	5.33×10^{-6}	Intronic	ZAN
chr1:104696748:A:C	rs116030389	1	104,696,748	0.03	2	4.79×10^{-6}	0.6	2.2	1.69×10^{-5}	intergenic	AMY1C (938058)
chr1:104735774:T:C	rs76709326	1	104,735,774	0.03	2	4.79×10^{-6}	0.6	2.2	1.69×10^{-5}	intergenic	AMY1C (977084)
chr2:160740820:T:C	rs270931	2	160,740,820	0.04	1.9	5.34×10^{-6}	0.6	2.1	1.69×10^{-5}	intergenic	RBMS1 (247013)
chr17:74433821:G:T	rs1320039	17	74,433,821	0.03	2.3	5.59×10^{-6}	0.6	2.4	1.34×10^{-4}	intronic	GPRC5C
chr2:231524502:C:T	rs12694894	2	231,524,502	0.22	1.4	5.72×10^{-6}	0.6	1.5	1.14×10^{-5}	intronic	NMUR1

chr6:138129769:T:C	rs55819060	6	138,129,769	0.15	1.5	$5.75x10^{-6}$	0.6	1.5	$4.33x10^{-5}$	intergenic	PERP (22350)
chr4:160105317:A:G	rs17327389	4	160,105,317	0.02	2.1	$5.85x10^{-6}$	0.6	2.3	$6.87x10^{-5}$	intergenic	RAPGEF2 (745144)
chr10:130567926:G:A	rs11017335	10	130,567,926	0.22	1.4	$5.97x10^{-6}$	0.6	1.4	$5.43x10^{-5}$	intergenic	GLRX3 (387549)
chr3:73160451:T:C	rs79039859	3	73,160,451	0.12	1.5	$6.07x10^{-6}$	0.6	1.6	$1.24x10^{-5}$	intergenic	PPP4R2 (91253)
chr6:50405460:T:C	rs79235865	6	50,405,460	0.03	1.9	$6.22x10^{-6}$	0.6	2.2	$1.11x10^{-5}$	intergenic	TFAP2D (308066)
chr11:106890513:G:C	rs949003	11	106,890,513	0.07	1.7	$6.22x10^{-6}$	0.6	1.9	$2.57x10^{-6}$	intergenic	GUCY1A2
chr8:18782343:T:C	rs73666715	8	18,782,343	0.03	2.1	$6.25x10^{-6}$	0.6	2.6	$9.17x10^{-6}$	intergenic	PSD3
chr16:82843437:A:G	rs11641036	16	82,843,437	0.39	1.4	$6.36x10^{-6}$	0.6	1.4	$1.43x10^{-5}$	intronic	CDH13
chr17:74433788:G:A	rs1320038	17	74,433,788	0.03	2.3	$6.45x10^{-6}$	0.6	2.4	$1.70x10^{-4}$	intronic	GPRC5C
chr16:82846736:T:G	rs1462051	16	82,846,736	0.4	1.4	$6.48x10^{-6}$	0.6	1.4	$1.30x10^{-5}$	intronic	CDH13
chr9:126220371:C:T	rs2150010	9	126,220,371	0.17	1.5	$6.50x10^{-6}$	0.6	1.5	$4.53x10^{-5}$	intergenic	MVB12B (106458)
chr17:74438470:G:A	rs4351086	17	74,438,470	0.05	2	$6.50x10^{-6}$	0.6	2.1	$5.75x10^{-5}$	intronic	GPRC5C
chr16:82844989:C:G	rs1903618	16	82,844,989	0.4	1.4	$7.05x10^{-6}$	0.6	1.4	$1.49x10^{-5}$	intronic	CDH13
chr16:82846452:T:G	rs12149890	16	82,846,452	0.4	1.4	$7.15x10^{-6}$	0.6	1.4	$1.43x10^{-5}$	intronic	CDH13
chr16:82846465:A:C	rs12929586	16	82,846,465	0.4	1.4	$7.15x10^{-6}$	0.6	1.4	$1.43x10^{-5}$	intronic	CDH13
chr8:18775429:T:G	rs79441119	8	18,775,429	0.03	2.2	$7.28x10^{-6}$	0.6	2.6	$1.03x10^{-5}$	intronic	PSD3
chr8:18779975:G:C	rs56062960	8	18,779,975	0.03	2.2	$7.65x10^{-6}$	0.6	2.6	$1.14x10^{-5}$	intronic	PSD3
chr6:138125420:T:C	rs112021110	6	138,125,420	0.15	1.5	$7.70x10^{-6}$	0.6	1.5	$5.47x10^{-5}$	intergenic	PERP (18001)
chr8:18779698:T:C	rs12676642	8	18,779,698	0.03	2.2	$7.75x10^{-6}$	0.6	2.6	$1.16x10^{-5}$	intronic	PSD3
chr8:18779932:A:C	rs57557661	8	18,779,932	0.03	2.2	$7.75x10^{-6}$	0.6	2.6	$1.16x10^{-5}$	intronic	PSD3
chr5:122199809:A:T	rs1421882	5	122,199,809	0.21	0.7	$7.91x10^{-6}$	0.6	0.6	$7.47x10^{-6}$	intergenic	ZNF474 (46240)
chr3:73137775:C:G	rs17010556	3	73,137,775	0.12	1.5	$8.21x10^{-6}$	0.6	1.6	$1.64x10^{-5}$	intergenic	PPP4R2 (68577)
chr8:18780560:A:T	rs4921961	8	18,780,560	0.03	2.1	$8.36x10^{-6}$	0.6	2.6	$1.25x10^{-5}$	intronic	PSD3
chr3:73173278:G:A	rs75037888	3	73,173,278	0.11	1.5	$8.41x10^{-6}$	0.6	1.6	$3.49x10^{-5}$	intergenic	PPP4R2 (104080)
chr15:50803386:C:A	rs117063109	15	50,803,386	0.03	2.1	$8.49x10^{-6}$	0.6	2.4	$2.07x10^{-5}$	intergenic	SPPL2A (37680)
chr3:137349171:C:T	rs6780666	3	137,349,171	0.38	1.4	$8.49x10^{-6}$	0.6	1.4	$8.74x10^{-5}$	intergenic	IL20RB (338086)
chr8:18776586:C:A	rs4921958	8	18,776,586	0.03	2.1	$8.51x10^{-6}$	0.6	2.5	$1.29x10^{-5}$	intronic	PSD3
chr8:18779139:T:C	rs13439022	8	18,779,139	0.03	2.1	$8.54x10^{-6}$	0.6	2.5	$1.45x10^{-5}$	intronic	PSD3
chr13:62572801:T:C	rs2809621	13	62,572,801	0.02	2.2	$8.56x10^{-6}$	0.6	2.5	$4.47x10^{-5}$	intronic non-coding transcript	PCDH20 (1156952)
chr12:10372222:A:G	rs17549124	12	10,372,222	0.05	1.8	$8.63x10^{-6}$	0.6	1.8	$1.30x10^{-4}$	downstream	KLRK1 (131)
chr6:138129468:T:A	rs12661738	6	138,129,468	0.15	1.5	$8.69x10^{-6}$	0.6	1.5	$5.55x10^{-5}$	TF binding site	PERP (22049)
chr6:138129535:G:A	rs12660248	6	138,129,535	0.15	1.5	$8.69x10^{-6}$	0.6	1.5	$5.55x10^{-5}$	regulatory region	PERP (22116)
chr2:160732035:G:C	rs4664363	2	160,732,035	0.04	1.9	$8.73x10^{-6}$	0.6	2.1	$2.91x10^{-5}$	regulatory region	RBMS1 (238228)
chr16:82843614:A:C	rs1947715	16	82,843,614	0.39	1.4	$8.90x10^{-6}$	0.6	1.4	$1.73x10^{-5}$	intronic	CDH13

chr6:138138672:G:A	rs12661760	6	138,138,672	0.16	1.5	$8.91x10^{-6}$	0.6	1.5	$9.51x10^{-5}$	intergenic	ARFGEF3 (23267)
chr6:93020601:G:A	rs146803692	6	93,020,601	0.02	2.2	$8.95x10^{-6}$	0.6	2.3	$3.01x10^{-4}$	intergenic	EPHA7 (219419)
chr2:160723228:A:G	rs2884325	2	160,723,228	0.04	1.9	$8.96x10^{-6}$	0.6	2.1	$2.95x10^{-5}$	intergenic	RBMS1 (229421)
chr10:123521619:G:A	rs841005	10	123,521,619	0.08	1.6	$9.12x10^{-6}$	0.6	1.7	$4.56x10^{-5}$	intronic non-coding transcript	GPR26 (144736)
chr15:50778834:C:T	rs147010615	15	50,778,834	0.03	2.1	$9.17x10^{-6}$	0.6	2.3	$2.19x10^{-5}$	intergenic	SPPL2A (13128)
chr5:10490293:A:T	rs1996254	5	10,490,293	0.49	0.7	$9.20x10^{-6}$	0.6	0.7	$3.82x10^{-5}$	regulatory region	MARCHF6 (49905)
chr8:4258903:G:T	rs73500607	8	4,258,903	0.08	1.6	$9.24x10^{-6}$	0.6	1.7	$7.68x10^{-5}$	intronic	CSMD1
chr2:157511744:T:A	rs72909677	2	157,511,744	0.02	2.2	$9.27x10^{-6}$	0.6	2.5	$6.16x10^{-5}$	intergenic	ACVR1C (15023)
chr6:138129016:G:A	rs72984688	6	138,129,016	0.15	1.5	$9.34x10^{-6}$	0.6	1.5	$6.14x10^{-5}$	Intergenic	PERP (21597)
chr22:17359371:C:G	rs35586716	22	17,359,371	0.04	1.9	$9.68x10^{-6}$	0.6	1.9	$3.21x10^{-4}$	intergenic	CECR2
chr3:73178392:T:A	rs61461427	3	73,178,392	0.12	1.5	$9.70x10^{-6}$	0.6	1.5	$1.14x10^{-4}$	intergenic	PPP4R2 (109194)
chr4:54446910:T:A	rs1553659	4	54,446,910	0.14	1.5	$9.72x10^{-6}$	0.6	1.6	$4.05x10^{-5}$	intergenic	PDGFRA (148665)
chr6:138127621:A:C	rs1334704	6	138,127,621	0.15	1.5	$9.77x10^{-6}$	0.6	1.5	$6.40x10^{-5}$	intergenic	PERP (20202)
chr14:76956523:G:A	rs6574342	14	76,956,523	0.13	1.5	$9.88x10^{-6}$	0.6	1.5	$7.27x10^{-5}$	TF binding protein	IRF2BPL (68020)
chr5:11132230:G:T	rs2158444	5	11,132,230	0.43	0.7	$9.92x10^{-6}$	0.6	0.7	$4.12x10^{-5}$	Intronic	CTNND2
chr5:11133041:C:G	rs6876115	5	11,133,041	0.43	0.7	$9.92x10^{-6}$	0.6	0.7	$4.12x10^{-5}$	Intronic	CTNND2

* According to GRCh38 human genome reference assembly; § Gene where the SNPs map or nearest gene (distance is indicated in brackets, in bp).
