# Supplementary material for: A genome-wide association study for survival from a multi-centre European study identified variants associated with COVID-19 risk of death
Source: Sci Rep. 2024 Feb 6;14:3000. doi: 10.1038/s41598-024-53310-x (PMC10847137; doi:10.1038/s41598-024-53310-x)
Supplement: Supplementary file 5 — Supplementary Table S3. [file 41598_2024_53310_MOESM5_ESM.pdf]

Supplementary Table S3. eQTLs found in GTEx database, among the top 113 SNPs from the survival GWAS

rsID	variant_id	phenotype_id	gene symbol	tss_distance	pval_nominal	slope	slope_se	tissue
rs17549124	chr12_10372222_A_G_b38	ENSG00000134545.13	KLRC1	-82463	0.000169229	-0.756309	0.198678	Esophagus_Muscularis
	chr12_10372222_A_G_b38	ENSG00000150045.11	KLRF1	544741	5.15E-05	-0.423828	0.103276	Adipose_Visceral_Omentum
rs6574342	chr14_76956523_G_A_b38	ENSG00000243225.2	RP11-7F17.1	-12160	3.47E-09	-0.618065	0.101042	Heart_Atrial_Appendage
	chr14_76956523_G_A_b38	ENSG00000243225.2	RP11-7F17.1	-12160	1.48E-12	-0.693011	0.0933691	Heart_Left_Ventricle
	chr14_76956523_G_A_b38	ENSG00000243225.2	RP11-7F17.1	-12160	1.69E-06	-0.56859	0.115561	Esophagus_Gastroesophageal_Junction
	chr14_76956523_G_A_b38	ENSG00000243225.2	RP11-7F17.1	-12160	4.80E-05	-0.417791	0.101138	Artery_Aorta
	chr14_76956523_G_A_b38	ENSG00000243225.2	RP11-7F17.1	-12160	3.17E-10	-0.614473	0.0945543	Esophagus_Muscularis
	chr14_76956523_G_A_b38	ENSG00000243225.2	RP11-7F17.1	-12160	1.57E-06	-0.537371	0.108717	Colon_Sigmoid
	chr14_76956523_G_A_b38	ENSG00000243225.2	RP11-7F17.1	-12160	1.95E-10	-0.573467	0.0878034	Artery_Tibial
	chr14_76956523_G_A_b38	ENSG00000243225.2	RP11-7F17.1	-12160	1.60E-07	-0.812442	0.147308	Prostate
	chr14_76956523_G_A_b38	ENSG00000258602.1	RP11-7F17.7	-3115	1.40E-11	-0.618321	0.0888747	Artery_Tibial
	chr14_76956523_G_A_b38	ENSG00000258602.1	RP11-7F17.7	-3115	3.46E-07	-0.496656	0.0947109	Colon_Transverse
	chr14_76956523_G_A_b38	ENSG00000258602.1	RP11-7F17.7	-3115	6.68E-08	-0.794491	0.139385	Prostate
	chr14_76956523_G_A_b38	ENSG00000258602.1	RP11-7F17.7	-3115	4.60E-08	-0.55227	0.0985694	Esophagus_Muscularis
	chr14_76956523_G_A_b38	ENSG00000258602.1	RP11-7F17.7	-3115	4.17E-10	-0.599482	0.092441	Heart_Left_Ventricle
	chr14_76956523_G_A_b38	ENSG00000258602.1	RP11-7F17.7	-3115	8.37E-09	-0.622665	0.104564	Heart_Atrial_Appendage
	chr14_76956523_G_A_b38	ENSG00000258602.1	RP11-7F17.7	-3115	3.14E-05	-0.438953	0.103676	Artery_Aorta
	chr14_76956523_G_A_b38	ENSG00000266553.2	RN7SL356P	-11242	6.74E-07	-0.506812	0.0995009	Heart_Atrial_Appendage
rs147010615	chr15_50778834_C_T_b38	ENSG00000186417.13	GLDN	-562795	2.10E-05	0.717239	0.166126	Adipose_Visceral_Omentum
rs117063109	chr15_50803386_C_A_b38	ENSG00000186417.13	GLDN	-538243	2.10E-05	0.717239	0.166126	Adipose_Visceral_Omentum
rs11641036	chr16_82843437_A_G_b38	ENSG00000140945.16	CDH13	216634	1.42E-05	0.192382	0.0435205	Artery_Aorta
rs1947715	chr16_82843614_A_C_b38	ENSG00000140945.16	CDH13	216811	2.11E-05	0.189161	0.0437083	Artery_Aorta
rs62035176	chr16_82844550_G_A_b38	ENSG00000140945.16	CDH13	217747	8.91E-07	0.214403	0.0426293	Artery_Aorta
rs1903618	chr16_82844989_C_G_b38	ENSG00000140945.16	CDH13	218186	2.62E-05	0.185699	0.0434222	Artery_Aorta
rs4783291	chr16_82845653_T_A_b38	ENSG00000140945.16	CDH13	218850	5.42E-05	0.125066	0.0306538	Artery_Tibial
	chr16_82845653_T_A_b38	ENSG00000140945.16	CDH13	218850	3.30E-06	0.202676	0.0426774	Artery_Aorta
rs4782734	chr16_82845739_A_T_b38	ENSG00000140945.16	CDH13	218936	5.42E-05	0.125066	0.0306538	Artery_Tibial
	chr16_82845739_A_T_b38	ENSG00000140945.16	CDH13	218936	3.30E-06	0.202676	0.0426774	Artery_Aorta
rs12149890	chr16_82846452_T_G_b38	ENSG00000140945.16	CDH13	219649	2.62E-05	0.185699	0.0434222	Artery_Aorta
rs12929586	chr16_82846465_A_C_b38	ENSG00000140945.16	CDH13	219662	2.62E-05	0.185699	0.0434222	Artery_Aorta
rs1462051	chr16_82846736_T_G_b38	ENSG00000140945.16	CDH13	219933	2.62E-05	0.185699	0.0434222	Artery_Aorta
rs1320038	chr17_74433788_G_A_b38	ENSG00000125450.10	NUP85	-771871	2.80E-05	0.710238	0.167403	Nerve_Tibial
	chr17_74433788_G_A_b38	ENSG00000125450.10	NUP85	-771871	2.48E-05	0.671642	0.157445	Artery_Tibial
	chr17_74433788_G_A_b38	ENSG00000170190.15	SLC16A5	-653939	9.26E-05	0.55589	0.140415	Adipose_Visceral_Omentum
rs1320039	chr17_74433821_G_T_b38	ENSG00000125450.10	NUP85	-771838	2.60E-05	0.669639	0.157397	Artery_Tibial
	chr17_74433821_G_T_b38	ENSG00000125450.10	NUP85	-771838	2.89E-05	0.708894	0.167384	Nerve_Tibial
	chr17_74433821_G_T_b38	ENSG00000170190.15	SLC16A5	-653906	9.26E-05	0.55589	0.140415	Adipose_Visceral_Omentum
rs12694894	chr2_231524502_C_T_b38	ENSG00000171596.6	NMUR1	-5993	5.13E-13	0.451464	0.059893	Esophagus_Muscularis
	chr2_231524502_C_T_b38	ENSG00000171596.6	NMUR1	-5993	4.59E-18	0.51017	0.0542677	Colon_Transverse
	chr2_231524502_C_T_b38	ENSG00000171596.6	NMUR1	-5993	2.49E-20	0.376478	0.0383813	Nerve_Tibial
	chr2_231524502_C_T_b38	ENSG00000171596.6	NMUR1	-5993	1.88E-08	0.25415	0.0443047	Adipose_Subcutaneous
	chr2_231524502_C_T_b38	ENSG00000171596.6	NMUR1	-5993	2.48E-20	0.733602	0.0714184	Colon_Sigmoid
	chr2_231524502_C_T_b38	ENSG00000171596.6	NMUR1	-5993	8.25E-09	0.248345	0.0421861	Artery_Tibial
	chr2_231524502_C_T_b38	ENSG00000171596.6	NMUR1	-5993	1.07E-08	0.743199	0.120672	Brain_Cerebellar_Hemisphere
	chr2_231524502_C_T_b38	ENSG00000171596.6	NMUR1	-5993	2.16E-08	0.296926	0.0520572	Skin_Sun_Exposed_Lower_leg

	chr2_231524502_C_T_b38	ENSG00000171596.6	NMUR1	-5993	6.14E-06	0.221686	0.0485209	Muscle_Skeletal
	chr2_231524502_C_T_b38	ENSG00000171596.6	NMUR1	-5993	3.05E-11	0.741271	0.103159	Brain_Cerebellum
	chr2_231524502_C_T_b38	ENSG00000171596.6	NMUR1	-5993	1.49E-13	0.501077	0.06441	Artery_Aorta
	chr2_231524502_C_T_b38	ENSG00000171596.6	NMUR1	-5993	1.45E-05	0.189706	0.0431554	Lung
	chr2_231524502_C_T_b38	ENSG00000171596.6	NMUR1	-5993	1.65E-08	0.381867	0.0649475	Stomach
	chr2_231524502_C_T_b38	ENSG00000171596.6	NMUR1	-5993	9.46E-06	0.227414	0.0505257	Adipose_Visceral_Omentum
	chr2_231524502_C_T_b38	ENSG00000171596.6	NMUR1	-5993	8.01E-08	0.587252	0.103725	Prostate
	chr2_231524502_C_T_b38	ENSG00000171596.6	NMUR1	-5993	6.76E-09	0.490373	0.0812879	Esophagus_Gastroesophageal_Junction
	chr2_231524502_C_T_b38	ENSG00000171596.6	NMUR1	-5993	3.45E-10	0.374359	0.0579728	Skin_Not_Sun_Exposed_Suprapubic
	chr2_231524502_C_T_b38	ENSG00000171596.6	NMUR1	-5993	9.58E-17	0.347413	0.040042	Thyroid
	chr2_231524502_C_T_b38	ENSG00000171596.6	NMUR1	-5993	7.68E-07	0.234482	0.0465506	Esophagus_Mucosa
	chr2_231524502_C_T_b38	ENSG00000181798.2	LINC00471	10163	9.51E-10	-0.339883	0.0545319	Muscle_Skeletal
	chr2_231524502_C_T_b38	ENSG00000181798.2	LINC00471	10163	2.00E-05	-0.370644	0.0853216	Heart_Atrial_Appendage
	chr2_231524502_C_T_b38	ENSG00000181798.2	LINC00471	10163	1.94E-07	-0.359297	0.0678608	Thyroid
	chr2_231524502_C_T_b38	ENSG00000181798.2	LINC00471	10163	1.60E-06	-0.352777	0.0722041	Cells_Cultured_fibroblasts
	chr2_231524502_C_T_b38	ENSG00000181798.2	LINC00471	10163	7.77E-05	-0.284886	0.0712833	Nerve_Tibial
	chr2_231524502_C_T_b38	ENSG00000181798.2	LINC00471	10163	4.67E-06	-0.527953	0.111034	Brain_Cerebellum
	chr2_231524502_C_T_b38	ENSG00000283312.1	GTF2I repeat domain containing 2(GTF2IRD2) pseudogene	29203	8.49E-06	-0.263718	0.0584721	Artery_Tibial
	chr2_231524502_C_T_b38	ENSG00000283312.1	GTF2I repeat domain containing 2(GTF2IRD2) pseudogene	29203	1.75E-05	-0.353213	0.0796554	Adrenal_Gland
	chr2_231524502_C_T_b38	ENSG00000283312.1	GTF2I repeat domain containing 2(GTF2IRD2) pseudogene	29203	9.80E-10	-0.298507	0.047701	Thyroid
	chr2_231524502_C_T_b38	ENSG00000283312.1	GTF2I repeat domain containing 2(GTF2IRD2) pseudogene	29203	1.27E-08	-0.506424	0.0837754	Brain_Nucleus_accumbens_basal_ganglia
	chr2_231524502_C_T_b38	ENSG00000283312.1	GTF2I repeat domain containing 2(GTF2IRD2) pseudogene	29203	3.54E-08	-0.418755	0.0737824	Heart_Left_Ventricle
	chr2_231524502_C_T_b38	ENSG00000283312.1	GTF2I repeat domain containing 2(GTF2IRD2) pseudogene	29203	2.07E-05	-0.258889	0.0597578	Breast_Mammary
	chr2_231524502_C_T_b38	ENSG00000283312.1	GTF2I repeat domain containing 2(GTF2IRD2) pseudogene	29203	9.20E-06	-0.422064	0.0914554	Brain_Caudate_basal_ganglia
	chr2_231524502_C_T_b38	ENSG00000283312.1	GTF2I repeat domain containing 2(GTF2IRD2) pseudogene	29203	1.60E-06	-0.526384	0.104102	Brain_Frontal_Cortex
	chr2_231524502_C_T_b38	ENSG00000283312.1	GTF2I repeat domain containing 2(GTF2IRD2) pseudogene	29203	1.27E-05	-0.312168	0.0700163	Colon_Transverse
	chr2_231524502_C_T_b38	ENSG00000283312.1	GTF2I repeat domain containing 2(GTF2IRD2) pseudogene	29203	3.20E-11	-0.47464	0.0691095	Cells_Cultured_fibroblasts
	chr2_231524502_C_T_b38	ENSG00000283312.1	GTF2I repeat domain containing 2(GTF2IRD2) pseudogene	29203	8.68E-05	-0.246278	0.0620469	Lung
	chr2_231524502_C_T_b38	ENSG00000283312.1	GTF2I repeat domain containing 2(GTF2IRD2) pseudogene	29203	5.36E-06	-0.53583	0.112329	Brain_Cerebellar_Hemisphere
	chr2_231524502_C_T_b38	ENSG00000283312.1	GTF2I repeat domain containing 2(GTF2IRD2) pseudogene	29203	7.99E-06	-0.350698	0.0769658	Heart_Atrial_Appendage
	chr2_231524502_C_T_b38	ENSG00000283312.1	GTF2I repeat domain containing 2(GTF2IRD2) pseudogene	29203	6.48E-13	-0.332485	0.0450716	Muscle_Skeletal
	chr2_231524502_C_T_b38	ENSG00000283312.1	GTF2I repeat domain containing 2(GTF2IRD2) pseudogene	29203	1.24E-07	-0.297916	0.0552208	Skin_Not_Sun_Exposed_Suprapubic
	chr2_231524502_C_T_b38	ENSG00000283312.1	GTF2I repeat domain containing 2(GTF2IRD2) pseudogene	29203	1.23E-05	-0.325797	0.0724289	Pituitary
	chr2_231524502_C_T_b38	ENSG00000283312.1	GTF2I repeat domain containing 2(GTF2IRD2) pseudogene	29203	4.13E-06	-0.219909	0.0471512	Skin_Sun_Exposed_Lower_leg
	chr2_231524502_C_T_b38	ENSG00000283312.1	GTF2I repeat domain containing 2(GTF2IRD2) pseudogene	29203	5.58E-05	-0.311976	0.0757743	Pancreas
	chr2_231524502_C_T_b38	ENSG00000283312.1	GTF2I repeat domain containing 2(GTF2IRD2) pseudogene	29203	6.21E-11	-0.327237	0.0485664	Nerve_Tibial
	chr2_231524502_C_T_b38	ENSG00000283312.1	GTF2I repeat domain containing 2(GTF2IRD2) pseudogene	29203	4.38E-06	-0.259428	0.0555757	Esophagus_Mucosa
	chr2_231524502_C_T_b38	ENSG00000283312.1	GTF2I repeat domain containing 2(GTF2IRD2) pseudogene	29203	3.71E-07	-0.486255	0.0911976	Brain_Cortex
	chr2_231524502_C_T_b38	ENSG00000283312.1	GTF2I repeat domain containing 2(GTF2IRD2) pseudogene	29203	3.87E-06	-0.265394	0.0566812	Adipose_Subcutaneous
	chr2_231524502_C_T_b38	ENSG00000283312.1	GTF2I repeat domain containing 2(GTF2IRD2) pseudogene	29203	4.43E-05	-0.295523	0.0709051	Testis
	chr2_231524502_C_T_b38	ENSG00000283312.1	GTF2I repeat domain containing 2(GTF2IRD2) pseudogene	29203	5.44E-09	-0.528109	0.0852256	Brain_Cerebellum
	chr2_231524502_C_T_b38	ENSG00000283491.1	GTF2I repeat domain containing 2(GTF2IRD2) pseudogene	17246	0.000131609	-0.267192	0.0692603	Skin_Sun_Exposed_Lower_leg
	chr2_231524502_C_T_b38	ENSG00000283491.1	GTF2I repeat domain containing 2(GTF2IRD2) pseudogene	17246	2.45E-05	-0.255536	0.0600072	Muscle_Skeletal
	chr2_231524502_C_T_b38	ENSG00000283491.1	GTF2I repeat domain containing 2(GTF2IRD2) pseudogene	17246	1.42E-07	-0.598805	0.108317	Brain_Cerebellum
	chr2_231524502_C_T_b38	ENSG00000283491.1	GTF2I repeat domain containing 2(GTF2IRD2) pseudogene	17246	1.12E-05	-0.308891	0.0694583	Thyroid
rs6780666	chr3_137349171_C_T_b38	ENSG00000174564.12	IL20RB	391306	0.000325767	-0.171473	0.0472143	Cells_Cultured_fibroblasts
rs61461427	chr3_73178392_T_A_b38	ENSG00000182921.9	CCDC75P1	-4899	2.25E-05	0.25052	0.0578243	Testis

rs111805058	chr3_73180132_C_G_b38	ENSG00000182921.9	CCDC75P1	-3159	6.65E-06	0.273908	0.0593261	Testis
rs112192337	chr3_73184232_C_T_b38	ENSG00000182921.9	CCDC75P1	941	6.65E-06	0.273908	0.0593261	Testis
rs1421882	chr5_122199809_A_T_b38	ENSG00000064692.18	SNCAIP	-111882	7.41E-07	-0.417888	0.0798519	Brain_Cerebellar_Hemisphere
	chr5_122199809_A_T_b38	ENSG00000064692.18	SNCAIP	-111882	7.78E-05	0.244195	0.0605898	Colon_Sigmoid
	chr5_122199809_A_T_b38	ENSG00000064692.18	SNCAIP	-111882	3.44E-06	-0.411618	0.0854302	Adrenal_Gland
	chr5_122199809_A_T_b38	ENSG00000250328.5	MGC32805	-279278	6.26E-05	-0.146204	0.0361011	Lung
rs76096820	chr6_116786541_G_C_b38	ENSG00000111834.12	RSPH4A	170062	0.000179698	-0.337119	0.0884465	Testis
	chr6_116786541_G_C_b38	ENSG00000153989.7	NUS1	-888961	0.00013946	-0.255943	0.0661956	Heart_Atrial_Appendage
	chr6_116786541_G_C_b38	ENSG00000189241.6	TSPYL1	506638	0.000289065	0.154684	0.0423816	Muscle_Skeletal
	chr6_116786541_G_C_b38	ENSG00000189241.6	TSPYL1	506638	5.42E-05	0.223916	0.0548802	Artery_Tibial
rs139799434	chr6_116851088_G_T_b38	ENSG00000153989.7	NUS1	-824414	4.28E-05	-0.278164	0.066827	Heart_Atrial_Appendage
	chr6_116851088_G_T_b38	ENSG00000189241.6	TSPYL1	571185	1.97E-05	0.242803	0.0562119	Artery_Tibial
	chr6_116851088_G_T_b38	ENSG00000189241.6	TSPYL1	571185	0.000389926	0.154184	0.0431864	Muscle_Skeletal
rs2327871	chr6_138098070_A_G_b38	ENSG00000112378.11	PERP	-9441	5.01E-06	-0.319279	0.0665053	Brain_Hippocampus
	chr6_138098070_A_G_b38	ENSG00000112378.11	PERP	-9441	1.30E-06	-0.394654	0.0773186	Brain_Frontal_Cortex
	chr6_138098070_A_G_b38	ENSG00000112378.11	PERP	-9441	3.41E-06	0.176888	0.0376708	Muscle_Skeletal
	chr6_138098070_A_G_b38	ENSG00000112378.11	PERP	-9441	2.09E-06	-0.139892	0.0290605	Artery_Tibial
	chr6_138098070_A_G_b38	ENSG00000112378.11	PERP	-9441	0.000147664	-0.128119	0.0333704	Cells_Cultured_fibroblasts
	chr6_138098070_A_G_b38	ENSG00000112378.11	PERP	-9441	1.62E-05	-0.365294	0.0801368	Brain_Spinal_cord_cervical
rs72984647	chr6_138105000_G_A_b38	ENSG00000112378.11	PERP	-2511	7.36E-06	-0.459036	0.0976431	Brain_Putamen_basal_ganglia
	chr6_138105000_G_A_b38	ENSG00000112378.11	PERP	-2511	2.59E-05	-0.407497	0.0927644	Brain_Hippocampus
	chr6_138105000_G_A_b38	ENSG00000112378.11	PERP	-2511	6.24E-06	-0.378751	0.0799852	Brain_Hypothalamus
	chr6_138105000_G_A_b38	ENSG00000112378.11	PERP	-2511	9.54E-12	-0.657453	0.0868808	Brain_Frontal_Cortex
	chr6_138105000_G_A_b38	ENSG00000112378.11	PERP	-2511	1.52E-05	-0.484786	0.105969	Brain_Spinal_cord_cervical
	chr6_138105000_G_A_b38	ENSG00000112378.11	PERP	-2511	6.66E-06	-0.395243	0.084497	Brain_Cortex
rs72984652	chr6_138106052_A_G_b38	ENSG00000112378.11	PERP	-1459	1.76E-05	-0.490642	0.10815	Brain_Spinal_cord_cervical
	chr6_138106052_A_G_b38	ENSG00000112378.11	PERP	-1459	7.36E-06	-0.459036	0.0976431	Brain_Putamen_basal_ganglia
	chr6_138106052_A_G_b38	ENSG00000112378.11	PERP	-1459	3.81E-12	-0.674318	0.0870689	Brain_Frontal_Cortex
	chr6_138106052_A_G_b38	ENSG00000112378.11	PERP	-1459	6.80E-06	-0.411794	0.0881295	Brain_Cortex
	chr6_138106052_A_G_b38	ENSG00000112378.11	PERP	-1459	5.55E-06	-0.385685	0.0809668	Brain_Hypothalamus
	chr6_138106052_A_G_b38	ENSG00000112378.11	PERP	-1459	2.59E-05	-0.407497	0.0927644	Brain_Hippocampus
rs12663855	chr6_138114271_C_G_b38	ENSG00000112378.11	PERP	6760	6.51E-06	-0.381101	0.0806553	Brain_Hypothalamus
	chr6_138114271_C_G_b38	ENSG00000112378.11	PERP	6760	1.66E-12	-0.678818	0.0858887	Brain_Frontal_Cortex
	chr6_138114271_C_G_b38	ENSG00000112378.11	PERP	6760	1.66E-05	-0.393545	0.0882921	Brain_Cortex
	chr6_138114271_C_G_b38	ENSG00000112378.11	PERP	6760	1.92E-05	-0.433187	0.0970524	Brain_Putamen_basal_ganglia
	chr6_138114271_C_G_b38	ENSG00000112378.11	PERP	6760	1.07E-05	-0.420272	0.0910472	Brain_Hippocampus
rs12528432	chr6_138119910_G_A_b38	ENSG00000112378.11	PERP	12399	1.66E-12	-0.678818	0.0858887	Brain_Frontal_Cortex
	chr6_138119910_G_A_b38	ENSG00000112378.11	PERP	12399	1.92E-05	-0.433187	0.0970524	Brain_Putamen_basal_ganglia
	chr6_138119910_G_A_b38	ENSG00000112378.11	PERP	12399	1.66E-05	-0.393545	0.0882921	Brain_Cortex
	chr6_138119910_G_A_b38	ENSG00000112378.11	PERP	12399	1.07E-05	-0.420272	0.0910472	Brain_Hippocampus
	chr6_138119910_G_A_b38	ENSG00000112378.11	PERP	12399	6.51E-06	-0.381101	0.0806553	Brain_Hypothalamus
rs112021110	chr6_138125420_T_C_b38	ENSG00000112378.11	PERP	17909	2.14E-05	-0.432602	0.0975123	Brain_Putamen_basal_ganglia
	chr6_138125420_T_C_b38	ENSG00000112378.11	PERP	17909	8.17E-12	-0.651448	0.0857465	Brain_Frontal_Cortex
	chr6_138125420_T_C_b38	ENSG00000112378.11	PERP	17909	2.60E-05	-0.379787	0.0873629	Brain_Cortex
	chr6_138125420_T_C_b38	ENSG00000112378.11	PERP	17909	2.77E-05	-0.353076	0.0808765	Brain_Hypothalamus
rs1334704	chr6_138127621_A_C_b38	ENSG00000112378.11	PERP	20110	1.08E-05	-0.367966	0.0799784	Brain_Hypothalamus
	chr6_138127621_A_C_b38	ENSG00000112378.11	PERP	20110	2.60E-05	-0.379787	0.0873629	Brain_Cortex

	chr6_138127621_A_C_b38	ENSG00000112378.11	PERP	20110	8.51E-13	-0.671376	0.0835969	Brain_Frontal_Cortex
	chr6_138127621_A_C_b38	ENSG00000112378.11	PERP	20110	2.35E-05	-0.429352	0.0973164	Brain_Putamen_basal_ganglia
rs72984688	chr6_138129016_G_A_b38	ENSG00000112378.11	PERP	21505	1.08E-05	-0.367966	0.0799784	Brain_Hypothalamus
	chr6_138129016_G_A_b38	ENSG00000112378.11	PERP	21505	8.51E-13	-0.671376	0.0835969	Brain_Frontal_Cortex
	chr6_138129016_G_A_b38	ENSG00000112378.11	PERP	21505	2.35E-05	-0.429352	0.0973164	Brain_Putamen_basal_ganglia
	chr6_138129016_G_A_b38	ENSG00000112378.11	PERP	21505	2.60E-05	-0.379787	0.0873629	Brain_Cortex
rs12661738	chr6_138129468_T_A_b38	ENSG00000112378.11	PERP	21957	2.60E-05	-0.379787	0.0873629	Brain_Cortex
	chr6_138129468_T_A_b38	ENSG00000112378.11	PERP	21957	1.08E-05	-0.367966	0.0799784	Brain_Hypothalamus
	chr6_138129468_T_A_b38	ENSG00000112378.11	PERP	21957	2.35E-05	-0.429352	0.0973164	Brain_Putamen_basal_ganglia
	chr6_138129468_T_A_b38	ENSG00000112378.11	PERP	21957	8.51E-13	-0.671376	0.0835969	Brain_Frontal_Cortex
rs12660248	chr6_138129535_G_A_b38	ENSG00000112378.11	PERP	22024	2.60E-05	-0.379787	0.0873629	Brain_Cortex
	chr6_138129535_G_A_b38	ENSG00000112378.11	PERP	22024	8.51E-13	-0.671376	0.0835969	Brain_Frontal_Cortex
	chr6_138129535_G_A_b38	ENSG00000112378.11	PERP	22024	1.08E-05	-0.367966	0.0799784	Brain_Hypothalamus
	chr6_138129535_G_A_b38	ENSG00000112378.11	PERP	22024	2.35E-05	-0.429352	0.0973164	Brain_Putamen_basal_ganglia
rs55819060	chr6_138129769_T_C_b38	ENSG00000112378.11	PERP	22258	2.77E-05	-0.353076	0.0808765	Brain_Hypothalamus
	chr6_138129769_T_C_b38	ENSG00000112378.11	PERP	22258	2.60E-05	-0.379787	0.0873629	Brain_Cortex
	chr6_138129769_T_C_b38	ENSG00000112378.11	PERP	22258	2.14E-05	-0.432602	0.0975123	Brain_Putamen_basal_ganglia
	chr6_138129769_T_C_b38	ENSG00000112378.11	PERP	22258	8.17E-12	-0.651448	0.0857465	Brain_Frontal_Cortex
rs55808177	chr6_138135822_A_G_b38	ENSG00000112378.11	PERP	28311	2.77E-05	-0.353076	0.0808765	Brain_Hypothalamus
	chr6_138135822_A_G_b38	ENSG00000112378.11	PERP	28311	8.17E-12	-0.651448	0.0857465	Brain_Frontal_Cortex
	chr6_138135822_A_G_b38	ENSG00000112378.11	PERP	28311	2.14E-05	-0.432602	0.0975123	Brain_Putamen_basal_ganglia
	chr6_138135822_A_G_b38	ENSG00000112378.11	PERP	28311	2.60E-05	-0.379787	0.0873629	Brain_Cortex
rs12661760	chr6_138138672_G_A_b38	ENSG00000112378.11	PERP	31161	3.15E-13	-0.675226	0.0821467	Brain_Frontal_Cortex
rs314296	chr7_100772252_T_C_b38	ENSG00000087085.13	ACHE	-124722	3.52E-07	0.254109	0.0490752	Thyroid
	chr7_100772252_T_C_b38	ENSG00000087085.13	ACHE	-124722	8.43E-07	0.280577	0.0559919	Nerve_Tibial
	chr7_100772252_T_C_b38	ENSG00000087085.13	ACHE	-124722	2.75E-06	0.220727	0.0464148	Adipose_Subcutaneous
	chr7_100772252_T_C_b38	ENSG00000087085.13	ACHE	-124722	6.69E-07	0.389118	0.0764626	Artery_Aorta
	chr7_100772252_T_C_b38	ENSG00000106330.11	MOSPD3	160150	2.70E-17	0.254222	0.028943	Whole_Blood
	chr7_100772252_T_C_b38	ENSG00000130427.2	EPO	51452	3.84E-06	0.47465	0.0978655	Brain_Cerebellar_Hemisphere
	chr7_100772252_T_C_b38	ENSG00000146828.17	SLC12A9	-54568	0.000186663	0.113564	0.0300853	Nerve_Tibial
	chr7_100772252_T_C_b38	ENSG00000146830.9	GIGYF1	82804	5.06E-07	0.117012	0.0228768	Nerve_Tibial
	chr7_100772252_T_C_b38	ENSG00000146830.9	GIGYF1	82804	1.96E-07	0.129725	0.024407	Cells_Cultured_fibroblasts
	chr7_100772252_T_C_b38	ENSG00000146830.9	GIGYF1	82804	5.63E-06	0.0760041	0.0164915	Skin_Not_Sun_Exposed_Suprapubic
	chr7_100772252_T_C_b38	ENSG00000146830.9	GIGYF1	82804	5.17E-08	0.163594	0.0293691	Esophagus_Mucosa
	chr7_100772252_T_C_b38	ENSG00000146830.9	GIGYF1	82804	9.84E-07	0.125842	0.0253208	Thyroid
	chr7_100772252_T_C_b38	ENSG00000146830.9	GIGYF1	82804	4.80E-10	0.121951	0.0191462	Skin_Sun_Exposed_Lower_leg
	chr7_100772252_T_C_b38	ENSG00000146830.9	GIGYF1	82804	2.10E-05	0.131055	0.030242	Heart_Atrial_Appendage
	chr7_100772252_T_C_b38	ENSG00000146830.9	GIGYF1	82804	1.17E-06	0.134739	0.0271795	Esophagus_Muscularis
	chr7_100772252_T_C_b38	ENSG00000146830.9	GIGYF1	82804	1.52E-06	0.134304	0.0274074	Adipose_Visceral_Omentum
	chr7_100772252_T_C_b38	ENSG00000146830.9	GIGYF1	82804	1.15E-07	0.109477	0.0202819	Artery_Tibial
	chr7_100772252_T_C_b38	ENSG00000146830.9	GIGYF1	82804	6.75E-05	0.219548	0.0535895	Adrenal_Gland
	chr7_100772252_T_C_b38	ENSG00000146830.9	GIGYF1	82804	2.58E-06	0.212428	0.0437277	Pituitary
	chr7_100772252_T_C_b38	ENSG00000146830.9	GIGYF1	82804	6.43E-07	0.114704	0.0226802	Adipose_Subcutaneous
	chr7_100772252_T_C_b38	ENSG00000146830.9	GIGYF1	82804	9.70E-08	0.123072	0.0227336	Whole_Blood
	chr7_100772252_T_C_b38	ENSG00000146830.9	GIGYF1	82804	5.87E-05	0.113046	0.0276953	Breast_Mammary
	chr7_100772252_T_C_b38	ENSG00000146830.9	GIGYF1	82804	4.04E-05	0.218097	0.0514269	Spleen
	chr7_100772252_T_C_b38	ENSG00000146839.18	ZAN	38626	5.82E-18	0.557005	0.0589895	Testis

	chr7_100772252_T_C_b38	ENSG00000146839.18	ZAN	38626	0.00015283	-0.17996	0.0470221	Skin_Not_Sun_Exposed_Suprapubic
	chr7_100772252_T_C_b38	ENSG00000166529.14	ZSCAN21	722478	0.000109349	-0.136768	0.0349279	Cells_Cultured_fibroblasts
	chr7_100772252_T_C_b38	ENSG00000172354.9	GNB2	98721	4.11E-06	-0.0894387	0.0190951	Cells_Cultured_fibroblasts
	chr7_100772252_T_C_b38	ENSG00000176125.4	UFSP1	-117466	2.29E-06	-0.170701	0.0354499	Esophagus_Muscularis
	chr7_100772252_T_C_b38	ENSG00000176125.4	UFSP1	-117466	9.92E-05	-0.264167	0.0663392	Pituitary
	chr7_100772252_T_C_b38	ENSG00000196411.9	EPHB4	-55269	1.18E-25	-0.352902	0.0318127	Whole_Blood
	chr7_100772252_T_C_b38	ENSG00000196411.9	EPHB4	-55269	2.81E-05	0.176706	0.0416195	Esophagus_Mucosa
	chr7_100772252_T_C_b38	ENSG00000196411.9	EPHB4	-55269	4.20E-05	0.136154	0.0327696	Esophagus_Muscularis
	chr7_100772252_T_C_b38	ENSG00000205307.11	SAP25	198432	0.000138685	-0.168021	0.043581	Cells_Cultured_fibroblasts
	chr7_100772252_T_C_b38	ENSG00000224729.5	PCOLCE-AS1	168294	0.000236766	0.173929	0.0468903	Artery_Tibial
rs2026810	chr9_126214010_G_A_b38	ENSG00000196814.14	MVB12B	-112839	1.15E-05	0.155581	0.035096	Whole_Blood
rs2150010	chr9_126220371_C_T_b38	ENSG00000196814.14	MVB12B	-106478	0.000165637	0.130103	0.0342747	Whole_Blood
rs2809425	chr9_126223597_G_T_b38	ENSG00000196814.14	MVB12B	-103252	0.000164077	0.131021	0.0344948	Whole_Blood
rs2809426	chr9_126223762_G_A_b38	ENSG00000196814.14	MVB12B	-103087	0.000166719	0.130976	0.0345198	Whole_Blood
rs1888160	chr9_126225879_C_A_b38	ENSG00000196814.14	MVB12B	-100970	0.000192651	0.133462	0.0355223	Whole_Blood
rs944221	chr9_126226220_T_C_b38	ENSG00000196814.14	MVB12B	-100629	1.71E-05	0.158891	0.0365882	Whole_Blood
