# Supplementary material for: A genome-wide association study for survival from a multi-centre European study identified variants associated with COVID-19 risk of death
Source: Sci Rep. 2024 Feb 6;14:3000. doi: 10.1038/s41598-024-53310-x (PMC10847137; doi:10.1038/s41598-024-53310-x)
Supplement: Supplementary file 6 — Supplementary Table S4. [file 41598_2024_53310_MOESM6_ESM.pdf]

Supplementary Table S4. eQTLs found in eQTLGen dataset, among the top 113 SNPs of the survival GWAS

SNP	Pvalue	Zscore	Gene	GeneSymbol	FDR	BonferroniP
rs72909677	2.28E-15	-7.9249	ENSG00000115165	CYTIP	0	2.91E-07
rs12694894	5.79E-07	4.9982	ENSG00000231494	AC104634.3	0.0017638	1
rs17010556	3.21E-17	8.4389	ENSG00000255423	EBLN2	0	4.08E-09
rs79039859	8.63E-17	8.3224	ENSG00000255423	EBLN2	0	1.10E-08
rs75037888	8.08E-14	7.4692	ENSG00000255423	EBLN2	0	1.03E-05
rs61461427	2.34E-18	8.7395	ENSG00000255423	EBLN2	0	2.98E-10
rs111805058	1.52E-17	8.5261	ENSG00000255423	EBLN2	0	1.93E-09
rs112192337	3.00E-17	8.4465	ENSG00000255423	EBLN2	0	3.83E-09
rs17327389	2.20E-06	4.7339	ENSG00000109756	RAPGEF2	0.00603721	1
rs139799434	2.42E-41	-13.4678	ENSG00000111817	DSE	0	3.08E-33
rs139799434	3.50E-06	4.6392	ENSG00000189241	TSPYL1	0.00936461	1
rs139799434	1.21E-18	8.8137	ENSG00000188820	FAM26F	0	1.54E-10
rs2327871	2.54E-11	-6.6709	ENSG00000051620	HEBP2	0	0.00323537
rs314296	4.18E-06	4.602	ENSG00000077454	LRCH4	0.01114596	1
rs314296	3.27E-310	60.6928	ENSG00000196411	EPHB4	0	4.17E-302
rs314296	2.43E-11	-6.6775	ENSG00000106327	TFR2	0	0.003093
rs314296	1.29E-61	-16.5631	ENSG00000106330	MOSPD3	0	1.64E-53
rs314296	1.51E-66	17.2327	ENSG00000176125	UFSP1	0	1.93E-58
rs314296	5.47E-07	5.0092	ENSG00000066923	STAG3	0.00164825	1
rs314296	2.40E-13	7.3243	ENSG00000146833	TRIM4	0	3.06E-05
rs314296	5.41E-06	-4.5482	ENSG00000146826	C7orf43	0.01449084	1
rs314296	3.34E-46	-14.2705	ENSG00000146830	GIGYF1	0	4.25E-38
rs314296	4.06E-18	8.6773	ENSG00000087077	TRIP6	0	5.17E-10
rs314296	9.25E-25	10.2739	ENSG00000146828	SLC12A9	0	1.18E-16
rs2026810	2.18E-07	5.183	ENSG00000196814	MVB12B	0.00066184	1
rs2150010	2.56E-07	5.1534	ENSG00000196814	MVB12B	0.00076612	1
rs2809425	5.97E-07	4.9922	ENSG00000196814	MVB12B	0.0018128	1
rs2809426	2.12E-07	5.1889	ENSG00000196814	MVB12B	0.00064959	1
rs2773391	5.71E-07	5.0009	ENSG00000196814	MVB12B	0.00175233	1
rs17549124	3.39E-26	-10.5876	ENSG00000205810	KLRC3	0	4.32E-18
rs17549124	3.01E-19	-8.968	ENSG00000134545	KLRC1	0	3.84E-11
rs6574342	1.20E-52	-15.2702	ENSG00000119669	IRF2BPL	0	1.53E-44
rs6574342	1.45E-07	-5.2581	ENSG00000071246	VASH1	0.00045141	1
rs147010615	4.59E-06	4.5827	ENSG00000138600	SPPL2A	0.01231399	1
rs117063109	1.30E-05	4.3601	ENSG00000138600	SPPL2A	0.03347375	1
rs73348353	5.58E-07	-5.0052	ENSG00000167850	CD300C	0.00168487	1
rs73348353	1.62E-07	5.2389	ENSG00000178789	CD300LB	0.00050113	1
rs57063902	6.11E-14	7.5058	ENSG00000178789	CD300LB	0	7.77E-06
rs57063902	4.87E-107	21.9762	ENSG00000170412	GPRC5C	0	6.20E-99
rs60879525	1.13E-05	4.3901	ENSG00000167851	CD300A	0.02936314	1
rs60879525	5.58E-07	5.0053	ENSG00000178789	CD300LB	0.00168487	1
rs60879525	1.50E-21	-9.5347	ENSG00000167850	CD300C	0	1.91E-13
rs1320038	5.96E-08	-5.42	ENSG00000167850	CD300C	0.00023401	1
rs1320038	3.57E-07	5.0906	ENSG00000178789	CD300LB	0.001073	1

rs1320039	3.54E-07	5.0925	ENSG00000178789	CD300LB	0.001073	1
rs1320039	2.75E-08	-5.5562	ENSG00000167850	CD300C	0.00012112	1
rs4351086	1.21E-07	-5.2915	ENSG00000170412	GPRC5C	0.00037058	1
rs4351086	5.42E-07	-5.0107	ENSG00000204347	BTBD17	0.00163611	1
rs4351086	9.15E-50	-14.8315	ENSG00000167850	CD300C	0	1.17E-41
rs4351086	3.31E-08	-5.5238	ENSG00000167851	CD300A	0.00013361	1
rs7208524	4.84E-07	5.0327	ENSG00000178789	CD300LB	0.0014775	1
rs7208524	1.87E-25	-10.4264	ENSG00000167850	CD300C	0	2.39E-17
rs7208524	1.16E-06	4.8619	ENSG00000167851	CD300A	0.00325641	1
