# Supplementary material for: A genome-wide association study for survival from a multi-centre European study identified variants associated with COVID-19 risk of death
Source: Sci Rep. 2024 Feb 6;14:3000. doi: 10.1038/s41598-024-53310-x (PMC10847137; doi:10.1038/s41598-024-53310-x)
Supplement: Supplementary file 7 — Supplementary Table S5. [file 41598_2024_53310_MOESM7_ESM.pdf]

Supplementary Table S5a. *EPHA7* eQTL SNPs in LD with SNPs on chromosome 6, associated with COVID-19 survival

Query	RS ID	Position	R2	D'	Gene Symbol	Gencode ID	Tissue	Non-effect Allele Freq	Effect Allele Freq	Effect Size	P-value
rs146803692	rs1535833	chr6:93769380	0.00742434	1	EPHA7	ENSG00000135333.13	Adipose - Subcutaneous	C=0.722	T=0.278	-0.770455	1.90E-63
rs146803692	rs58205228	chr6:93778613	0.00764663	1	EPHA7	ENSG00000135333.13	Adipose - Subcutaneous	A=0.716	G=0.284	-0.767837	2.77E-63
rs146803692	rs9345321	chr6:93789199	0.00764663	1	EPHA7	ENSG00000135333.13	Adipose - Subcutaneous	T=0.716	G=0.284	-0.767837	2.77E-63
rs146803692	rs9345322	chr6:93790565	0.00764663	1	EPHA7	ENSG00000135333.13	Adipose - Subcutaneous	A=0.716	G=0.284	-0.767837	2.77E-63
rs146803692	rs9345323	chr6:93797607	0.00764663	1	EPHA7	ENSG00000135333.13	Adipose - Subcutaneous	C=0.716	T=0.284	-0.767837	2.77E-63
rs146803692	rs9342350	chr6:93798264	0.00764663	1	EPHA7	ENSG00000135333.13	Adipose - Subcutaneous	A=0.716	G=0.284	-0.767837	2.77E-63
rs146803692	rs9363029	chr6:93798520	0.00764663	1	EPHA7	ENSG00000135333.13	Adipose - Subcutaneous	T=0.716	G=0.284	-0.767837	2.77E-63
rs146803692	rs9353982	chr6:93802038	0.00764663	1	EPHA7	ENSG00000135333.13	Adipose - Subcutaneous	G=0.716	A=0.284	-0.767837	2.77E-63
rs146803692	rs9345324	chr6:93803037	0.00764663	1	EPHA7	ENSG00000135333.13	Adipose - Subcutaneous	C=0.716	A=0.284	-0.767837	2.77E-63
rs146803692	rs1570631	chr6:93811876	0.00753502	1	EPHA7	ENSG00000135333.13	Adipose - Subcutaneous	T=0.719	C=0.281	-0.759574	1.45E-59
rs146803692	rs77898219	chr6:93824234	0.00742434	1	EPHA7	ENSG00000135333.13	Adipose - Subcutaneous	C=0.722	-=0.278	-0.752617	1.58E-59
rs146803692	rs9363030	chr6:93806040	0.00746113	1	EPHA7	ENSG00000135333.13	Adipose - Subcutaneous	G=0.721	A=0.279	-0.757344	1.59E-59
rs146803692	rs471850	chr6:93766281	0.00817999	1	EPHA7	ENSG00000135333.13	Adipose - Subcutaneous	C=0.298	A=0.702	0.722148	8.24E-57
rs146803692	rs508511	chr6:93759983	0.0081412	1	EPHA7	ENSG00000135333.13	Adipose - Subcutaneous	C=0.297	T=0.703	0.709751	8.86E-55
rs146803692	rs538373	chr6:93732427	0.00746113	1	EPHA7	ENSG00000135333.13	Adipose - Subcutaneous	G=0.279	A=0.721	0.685223	1.01E-48
rs146803692	rs9294560	chr6:93823182	0.01244493	1	EPHA7	ENSG00000135333.13	Adipose - Subcutaneous	G=0.393	A=0.607	0.507369	5.45E-35
rs146803692	rs5878286	chr6:93823490	0.01244493	1	EPHA7	ENSG00000135333.13	Adipose - Subcutaneous	-=0.393	T=0.607	0.507292	1.66E-34
rs146803692	rs5878287	chr6:93823492	0.01244493	1	EPHA7	ENSG00000135333.13	Adipose - Subcutaneous	A=0.393	-=0.607	0.507292	1.66E-34
rs146803692	rs553697	chr6:93730393	0.00372217	1	EPHA7	ENSG00000135333.13	Adipose - Subcutaneous	T=0.162	C=0.838	0.615676	3.17E-27
rs146803692	rs9445060	chr6:93754781	0.01813537	1	EPHA7	ENSG00000135333.13	Adipose - Subcutaneous	A=0.485	G=0.515	0.430075	5.99E-25
rs146803692	rs13196582	chr6:93774046	0.01514718	1	EPHA7	ENSG00000135333.13	Adipose - Subcutaneous	C=0.56	T=0.44	0.379239	2.46E-19
rs146803692	rs6454936	chr6:93768526	0.01508619	1	EPHA7	ENSG00000135333.13	Adipose - Subcutaneous	C=0.561	T=0.439	0.377436	3.44E-19
rs146803692	rs1359241	chr6:93779417	0.01514718	1	EPHA7	ENSG00000135333.13	Adipose - Subcutaneous	A=0.56	C=0.44	0.376242	4.80E-19
rs146803692	rs9452238	chr6:93788831	0.01551771	1	EPHA7	ENSG00000135333.13	Adipose - Subcutaneous	A=0.554	G=0.446	0.376242	4.80E-19
rs146803692	rs9363030	chr6:93806040	0.00746113	1	EPHA7	ENSG00000135333.13	Lung	G=0.721	A=0.279	-0.467048	1.04E-18
rs146803692	rs1570631	chr6:93811876	0.00753502	1	EPHA7	ENSG00000135333.13	Lung	T=0.719	C=0.281	-0.467048	1.04E-18
rs146803692	rs11969246	chr6:93823113	0.01551771	1	EPHA7	ENSG00000135333.13	Adipose - Subcutaneous	G=0.554	T=0.446	0.372771	1.26E-18
rs146803692	rs58205228	chr6:93778613	0.00764663	1	EPHA7	ENSG00000135333.13	Lung	A=0.716	G=0.284	-0.459589	1.54E-18
rs146803692	rs9345321	chr6:93789199	0.00764663	1	EPHA7	ENSG00000135333.13	Lung	T=0.716	G=0.284	-0.459589	1.54E-18
rs146803692	rs9345322	chr6:93790565	0.00764663	1	EPHA7	ENSG00000135333.13	Lung	A=0.716	G=0.284	-0.459589	1.54E-18
rs146803692	rs9345323	chr6:93797607	0.00764663	1	EPHA7	ENSG00000135333.13	Lung	C=0.716	T=0.284	-0.459589	1.54E-18
rs146803692	rs9342350	chr6:93798264	0.00764663	1	EPHA7	ENSG00000135333.13	Lung	A=0.716	G=0.284	-0.459589	1.54E-18
rs146803692	rs9363029	chr6:93798520	0.00764663	1	EPHA7	ENSG00000135333.13	Lung	T=0.716	G=0.284	-0.459589	1.54E-18
rs146803692	rs9353982	chr6:93802038	0.00764663	1	EPHA7	ENSG00000135333.13	Lung	G=0.716	A=0.284	-0.459589	1.54E-18
rs146803692	rs9345324	chr6:93803037	0.00764663	1	EPHA7	ENSG00000135333.13	Lung	C=0.716	A=0.284	-0.459589	1.54E-18
rs146803692	rs9452240	chr6:93806820	0.01551771	1	EPHA7	ENSG00000135333.13	Adipose - Subcutaneous	G=0.554	T=0.446	0.371135	1.76E-18
rs146803692	rs7739743	chr6:93813381	0.01551771	1	EPHA7	ENSG00000135333.13	Adipose - Subcutaneous	G=0.554	A=0.446	0.371135	1.76E-18
rs146803692	rs9445075	chr6:93822458	0.01551771	1	EPHA7	ENSG00000135333.13	Adipose - Subcutaneous	T=0.554	A=0.446	0.371135	1.76E-18
rs146803692	rs9452225	chr6:93766609	0.01402424	1	EPHA7	ENSG00000135333.13	Adipose - Subcutaneous	C=0.579	A=0.421	0.369594	2.11E-18
rs146803692	rs77898219	chr6:93824234	0.00742434	1	EPHA7	ENSG00000135333.13	Lung	C=0.722	-=0.278	-0.456134	5.88E-18
rs146803692	rs508511	chr6:93759983	0.0081412	1	EPHA7	ENSG00000135333.13	Skin - Sun Exposed (Lower leg)	C=0.297	T=0.703	0.274292	1.32E-17
rs146803692	rs1535833	chr6:93769380	0.00742434	1	EPHA7	ENSG00000135333.13	Lung	C=0.722	T=0.278	-0.44499	2.25E-17
rs146803692	rs9363030	chr6:93806040	0.00746113	1	EPHA7	ENSG00000135333.13	Skin - Sun Exposed (Lower leg)	G=0.721	A=0.279	-0.281476	2.71E-17
rs146803692	rs471850	chr6:93766281	0.00817999	1	EPHA7	ENSG00000135333.13	Skin - Sun Exposed (Lower leg)	C=0.298	A=0.702	0.273553	4.10E-17
rs146803692	rs9353982	chr6:93802038	0.00764663	1	EPHA7	ENSG00000135333.13	Skin - Sun Exposed (Lower leg)	G=0.716	A=0.284	-0.276454	4.84E-17
rs146803692	rs1570631	chr6:93811876	0.00753502	1	EPHA7	ENSG00000135333.13	Skin - Sun Exposed (Lower leg)	T=0.719	C=0.281	-0.279518	5.05E-17
rs146803692	rs58205228	chr6:93778613	0.00764663	1	EPHA7	ENSG00000135333.13	Skin - Sun Exposed (Lower leg)	A=0.716	G=0.284	-0.274662	6.63E-17
rs146803692	rs9345323	chr6:93797607	0.00764663	1	EPHA7	ENSG00000135333.13	Skin - Sun Exposed (Lower leg)	C=0.716	T=0.284	-0.274662	6.63E-17
rs146803692	rs9342350	chr6:93798264	0.00764663	1	EPHA7	ENSG00000135333.13	Skin - Sun Exposed (Lower leg)	A=0.716	G=0.284	-0.274662	6.63E-17

rs146803692	rs9363029	chr6:93798520	0.00764663	1	EPHA7	ENSG00000135333.13	Skin - Sun Exposed (Lower leg)	T=0.716	G=0.284	-0.274662	6.63E-17
rs146803692	rs9345324	chr6:93803037	0.00764663	1	EPHA7	ENSG00000135333.13	Skin - Sun Exposed (Lower leg)	C=0.716	A=0.284	-0.274662	6.63E-17
rs146803692	rs9345321	chr6:93789199	0.00764663	1	EPHA7	ENSG00000135333.13	Skin - Sun Exposed (Lower leg)	T=0.716	G=0.284	-0.273298	8.03E-17
rs146803692	rs9345322	chr6:93790565	0.00764663	1	EPHA7	ENSG00000135333.13	Skin - Sun Exposed (Lower leg)	A=0.716	G=0.284	-0.273298	8.03E-17
rs146803692	rs1535833	chr6:93769380	0.00742434	1	EPHA7	ENSG00000135333.13	Skin - Sun Exposed (Lower leg)	C=0.722	T=0.278	-0.274816	9.27E-17
rs146803692	rs77898219	chr6:93824234	0.00742434	1	EPHA7	ENSG00000135333.13	Skin - Sun Exposed (Lower leg)	C=0.722	-=0.278	-0.274384	1.06E-16
rs146803692	rs471850	chr6:93766281	0.00817999	1	EPHA7	ENSG00000135333.13	Lung	C=0.298	A=0.702	0.4279	1.24E-16
rs146803692	rs538373	chr6:93732427	0.00746113	1	EPHA7	ENSG00000135333.13	Skin - Sun Exposed (Lower leg)	G=0.279	A=0.721	0.266249	9.79E-16
rs146803692	rs508511	chr6:93759983	0.0081412	1	EPHA7	ENSG00000135333.13	Lung	C=0.297	T=0.703	0.405027	3.04E-15
rs146803692	rs9363030	chr6:93806040	0.00746113	1	EPHA7	ENSG00000135333.13	Skin - Not Sun Exposed (Suprapubic)	G=0.721	A=0.279	-0.26209	8.68E-15
rs146803692	rs77898219	chr6:93824234	0.00742434	1	EPHA7	ENSG00000135333.13	Skin - Not Sun Exposed (Suprapubic)	C=0.722	-=0.278	-0.258658	1.10E-14
rs146803692	rs1570631	chr6:93811876	0.00753502	1	EPHA7	ENSG00000135333.13	Skin - Not Sun Exposed (Suprapubic)	T=0.719	C=0.281	-0.259435	1.84E-14
rs146803692	rs1535833	chr6:93769380	0.00742434	1	EPHA7	ENSG00000135333.13	Skin - Not Sun Exposed (Suprapubic)	C=0.722	T=0.278	-0.258257	2.03E-14
rs146803692	rs9342350	chr6:93798264	0.00764663	1	EPHA7	ENSG00000135333.13	Skin - Not Sun Exposed (Suprapubic)	A=0.716	G=0.284	-0.256364	2.36E-14
rs146803692	rs9363029	chr6:93798520	0.00764663	1	EPHA7	ENSG00000135333.13	Skin - Not Sun Exposed (Suprapubic)	T=0.716	G=0.284	-0.256364	2.36E-14
rs146803692	rs9353982	chr6:93802038	0.00764663	1	EPHA7	ENSG00000135333.13	Skin - Not Sun Exposed (Suprapubic)	G=0.716	A=0.284	-0.256364	2.36E-14
rs146803692	rs9345324	chr6:93803037	0.00764663	1	EPHA7	ENSG00000135333.13	Skin - Not Sun Exposed (Suprapubic)	C=0.716	A=0.284	-0.256364	2.36E-14
rs146803692	rs9345323	chr6:93797607	0.00764663	1	EPHA7	ENSG00000135333.13	Skin - Not Sun Exposed (Suprapubic)	C=0.716	T=0.284	-0.253851	3.29E-14
rs146803692	rs538373	chr6:93732427	0.00746113	1	EPHA7	ENSG00000135333.13	Lung	G=0.279	A=0.721	0.399654	3.68E-14
rs146803692	rs58205228	chr6:93778613	0.00764663	1	EPHA7	ENSG00000135333.13	Skin - Not Sun Exposed (Suprapubic)	A=0.716	G=0.284	-0.252522	5.58E-14
rs146803692	rs9345321	chr6:93789199	0.00764663	1	EPHA7	ENSG00000135333.13	Skin - Not Sun Exposed (Suprapubic)	T=0.716	G=0.284	-0.252522	5.58E-14
rs146803692	rs9345322	chr6:93790565	0.00764663	1	EPHA7	ENSG00000135333.13	Skin - Not Sun Exposed (Suprapubic)	A=0.716	G=0.284	-0.252522	5.58E-14
rs146803692	rs9353982	chr6:93802038	0.00764663	1	EPHA7	ENSG00000135333.13	Breast - Mammary Tissue	G=0.716	A=0.284	-0.435156	5.93E-13
rs146803692	rs58205228	chr6:93778613	0.00764663	1	EPHA7	ENSG00000135333.13	Breast - Mammary Tissue	A=0.716	G=0.284	-0.431238	6.79E-13
rs146803692	rs9345323	chr6:93797607	0.00764663	1	EPHA7	ENSG00000135333.13	Breast - Mammary Tissue	C=0.716	T=0.284	-0.431238	6.79E-13
rs146803692	rs9342350	chr6:93798264	0.00764663	1	EPHA7	ENSG00000135333.13	Breast - Mammary Tissue	A=0.716	G=0.284	-0.431238	6.79E-13
rs146803692	rs9363029	chr6:93798520	0.00764663	1	EPHA7	ENSG00000135333.13	Breast - Mammary Tissue	T=0.716	G=0.284	-0.431238	6.79E-13
rs146803692	rs9345324	chr6:93803037	0.00764663	1	EPHA7	ENSG00000135333.13	Breast - Mammary Tissue	C=0.716	A=0.284	-0.431238	6.79E-13
rs146803692	rs9363030	chr6:93806040	0.00746113	1	EPHA7	ENSG00000135333.13	Breast - Mammary Tissue	G=0.721	A=0.279	-0.42573	1.72E-12
rs146803692	rs1570631	chr6:93811876	0.00753502	1	EPHA7	ENSG00000135333.13	Breast - Mammary Tissue	T=0.719	C=0.281	-0.42573	1.72E-12
rs146803692	rs508511	chr6:93759983	0.0081412	1	EPHA7	ENSG00000135333.13	Breast - Mammary Tissue	C=0.297	T=0.703	0.411804	1.87E-12
rs146803692	rs1535833	chr6:93769380	0.00742434	1	EPHA7	ENSG00000135333.13	Breast - Mammary Tissue	C=0.722	T=0.278	-0.427079	1.88E-12
rs146803692	rs9345321	chr6:93789199	0.00764663	1	EPHA7	ENSG00000135333.13	Breast - Mammary Tissue	T=0.716	G=0.284	-0.422298	1.97E-12
rs146803692	rs9345322	chr6:93790565	0.00764663	1	EPHA7	ENSG00000135333.13	Breast - Mammary Tissue	A=0.716	G=0.284	-0.422298	1.97E-12
rs146803692	rs471850	chr6:93766281	0.00817999	1	EPHA7	ENSG00000135333.13	Skin - Not Sun Exposed (Suprapubic)	C=0.298	A=0.702	0.231641	2.54E-12
rs146803692	rs9363030	chr6:93806040	0.00746113	1	EPHA7	ENSG00000135333.13	Nerve - Tibial	G=0.721	A=0.279	-0.360092	2.81E-12
rs146803692	rs9363029	chr6:93798520	0.00764663	1	EPHA7	ENSG00000135333.13	Nerve - Tibial	T=0.716	G=0.284	-0.358549	2.86E-12
rs146803692	rs471850	chr6:93766281	0.00817999	1	EPHA7	ENSG00000135333.13	Breast - Mammary Tissue	C=0.298	A=0.702	0.415152	3.01E-12
rs146803692	rs9342350	chr6:93798264	0.00764663	1	EPHA7	ENSG00000135333.13	Nerve - Tibial	A=0.716	G=0.284	-0.358336	3.08E-12
rs146803692	rs9345324	chr6:93803037	0.00764663	1	EPHA7	ENSG00000135333.13	Nerve - Tibial	C=0.716	A=0.284	-0.358336	3.08E-12
rs146803692	rs9345323	chr6:93797607	0.00764663	1	EPHA7	ENSG00000135333.13	Nerve - Tibial	C=0.716	T=0.284	-0.352327	5.27E-12
rs146803692	rs9452184	chr6:93728200	0.0179201	1	EPHA7	ENSG00000135333.13	Adipose - Subcutaneous	T=0.482	G=0.518	0.313312	5.64E-12
rs146803692	rs77898219	chr6:93824234	0.00742434	1	EPHA7	ENSG00000135333.13	Breast - Mammary Tissue	C=0.722	-=0.278	-0.410352	5.75E-12
rs146803692	rs58205228	chr6:93778613	0.00764663	1	EPHA7	ENSG00000135333.13	Nerve - Tibial	A=0.716	G=0.284	-0.353209	6.01E-12
rs146803692	rs9353982	chr6:93802038	0.00764663	1	EPHA7	ENSG00000135333.13	Nerve - Tibial	G=0.716	A=0.284	-0.352973	7.65E-12
rs146803692	rs1570631	chr6:93811876	0.00753502	1	EPHA7	ENSG00000135333.13	Nerve - Tibial	T=0.719	C=0.281	-0.354962	8.15E-12
rs146803692	rs77898219	chr6:93824234	0.00742434	1	EPHA7	ENSG00000135333.13	Nerve - Tibial	C=0.722	-=0.278	-0.348397	9.13E-12
rs146803692	rs508511	chr6:93759983	0.0081412	1	EPHA7	ENSG00000135333.13	Skin - Not Sun Exposed (Suprapubic)	C=0.297	T=0.703	0.221947	9.53E-12
rs146803692	rs1535833	chr6:93769380	0.00742434	1	EPHA7	ENSG00000135333.13	Nerve - Tibial	C=0.722	T=0.278	-0.34822	1.38E-11
rs146803692	rs9345321	chr6:93789199	0.00764663	1	EPHA7	ENSG00000135333.13	Nerve - Tibial	T=0.716	G=0.284	-0.347757	1.48E-11
rs146803692	rs9345322	chr6:93790565	0.00764663	1	EPHA7	ENSG00000135333.13	Nerve - Tibial	A=0.716	G=0.284	-0.347757	1.48E-11

rs146803692	rs471850	chr6:93766281	0.00817999	1	EPHA7	ENSG00000135333.13	Nerve - Tibial	C=0.298	A=0.702	0.336681	1.73E-11
rs146803692	rs9445062	chr6:93755554	0.03639847	1	EPHA7	ENSG00000135333.13	Adipose - Subcutaneous	G=0.654	A=0.346	0.29883	1.89E-11
rs146803692	rs1014573	chr6:93756934	0.03655884	1	EPHA7	ENSG00000135333.13	Adipose - Subcutaneous	C=0.655	T=0.345	0.29883	1.89E-11
rs146803692	rs1986173	chr6:93756502	0.03639847	1	EPHA7	ENSG00000135333.13	Adipose - Subcutaneous	A=0.654	G=0.346	0.296067	3.01E-11
rs146803692	rs508511	chr6:93759983	0.0081412	1	EPHA7	ENSG00000135333.13	Nerve - Tibial	C=0.297	T=0.703	0.326769	4.40E-11
rs146803692	rs9294560	chr6:93823182	0.01244493	1	EPHA7	ENSG00000135333.13	Skin - Sun Exposed (Lower leg)	G=0.393	A=0.607	0.191768	6.27E-11
rs146803692	rs5878286	chr6:93823490	0.01244493	1	EPHA7	ENSG00000135333.13	Skin - Sun Exposed (Lower leg)	-=0.393	T=0.607	0.18936	1.11E-10
rs146803692	rs5878287	chr6:93823492	0.01244493	1	EPHA7	ENSG00000135333.13	Skin - Sun Exposed (Lower leg)	A=0.393	-=0.607	0.18936	1.11E-10
rs146803692	rs538373	chr6:93732427	0.00746113	1	EPHA7	ENSG00000135333.13	Breast - Mammary Tissue	G=0.279	A=0.721	0.394003	1.17E-10
rs146803692	rs9294560	chr6:93823182	0.01244493	1	EPHA7	ENSG00000135333.13	Lung	G=0.393	A=0.607	0.295686	2.00E-10
rs146803692	rs9445060	chr6:93754781	0.01813537	1	EPHA7	ENSG00000135333.13	Skin - Sun Exposed (Lower leg)	A=0.485	G=0.515	0.177558	6.96E-10
rs146803692	rs5878286	chr6:93823490	0.01244493	1	EPHA7	ENSG00000135333.13	Lung	-=0.393	T=0.607	0.287595	7.18E-10
rs146803692	rs5878287	chr6:93823492	0.01244493	1	EPHA7	ENSG00000135333.13	Lung	A=0.393	-=0.607	0.287595	7.18E-10
rs146803692	rs117365486	chr6:93820610	0.00063245	1	EPHA7	ENSG00000135333.13	Adipose - Subcutaneous	C=0.968	T=0.032	-0.679264	8.33E-10
rs146803692	rs148086058	chr6:93820887	0.00063245	1	EPHA7	ENSG00000135333.13	Adipose - Subcutaneous	C=0.968	A=0.032	-0.679264	8.33E-10
rs146803692	rs2476884	chr6:93737618	0.04846218	1	EPHA7	ENSG00000135333.13	Adipose - Subcutaneous	T=0.716	A=0.284	0.289164	9.73E-10
rs146803692	rs377586702	chr6:93736162	0.03925656	1	EPHA7	ENSG00000135333.13	Adipose - Subcutaneous	-=0.671	AAG=0.329	0.275498	1.51E-09
rs146803692	rs1590392	chr6:93735074	0.03873105	1	EPHA7	ENSG00000135333.13	Adipose - Subcutaneous	T=0.668	G=0.332	0.274016	1.85E-09
rs146803692	rs538373	chr6:93732427	0.00746113	1	EPHA7	ENSG00000135333.13	Skin - Not Sun Exposed (Suprapubic)	G=0.279	A=0.721	0.202336	2.00E-09
rs146803692	rs62414117	chr6:93747826	0.00688437	1	EPHA7	ENSG00000135333.13	Adipose - Subcutaneous	A=0.737	G=0.263	0.30945	2.14E-09
rs146803692	rs140630813	chr6:93748742	0.00688437	1	EPHA7	ENSG00000135333.13	Adipose - Subcutaneous	AAAG=0.737	-=0.263	0.30945	2.14E-09
rs146803692	rs1590390	chr6:93734963	0.04846218	1	EPHA7	ENSG00000135333.13	Adipose - Subcutaneous	G=0.716	A=0.284	0.281628	2.39E-09
rs146803692	rs2476891	chr6:93739608	0.03873105	1	EPHA7	ENSG00000135333.13	Adipose - Subcutaneous	G=0.668	A=0.332	0.271217	2.74E-09
rs146803692	rs2506923	chr6:93742094	0.03873105	1	EPHA7	ENSG00000135333.13	Adipose - Subcutaneous	A=0.668	G=0.332	0.271217	2.74E-09
rs146803692	rs62414114	chr6:93740390	0.00688437	1	EPHA7	ENSG00000135333.13	Adipose - Subcutaneous	C=0.737	G=0.263	0.307742	2.75E-09
rs146803692	rs145106301	chr6:93728868	0.04775928	1	EPHA7	ENSG00000135333.13	Adipose - Subcutaneous	AG=0.713	-=0.287	0.276396	2.81E-09
rs146803692	rs62415679	chr6:93794348	0.0048665	1	EPHA7	ENSG00000135333.13	Adipose - Subcutaneous	C=0.798	T=0.202	0.360505	2.86E-09
rs146803692	rs727998	chr6:93745434	0.03873105	1	EPHA7	ENSG00000135333.13	Adipose - Subcutaneous	T=0.668	A=0.332	0.269736	3.34E-09
rs146803692	rs1590388	chr6:93747226	0.03873105	1	EPHA7	ENSG00000135333.13	Adipose - Subcutaneous	T=0.668	C=0.332	0.269736	3.34E-09
rs146803692	rs2476886	chr6:93737850	0.04846218	1	EPHA7	ENSG00000135333.13	Adipose - Subcutaneous	G=0.716	A=0.284	0.275568	3.86E-09
rs146803692	rs2506910	chr6:93730374	0.04846218	1	EPHA7	ENSG00000135333.13	Adipose - Subcutaneous	A=0.716	G=0.284	0.273986	5.54E-09
rs146803692	rs2485806	chr6:93731257	0.04846218	1	EPHA7	ENSG00000135333.13	Adipose - Subcutaneous	G=0.716	A=0.284	0.273986	5.54E-09
rs146803692	rs2506911	chr6:93731302	0.04846218	1	EPHA7	ENSG00000135333.13	Adipose - Subcutaneous	A=0.716	G=0.284	0.273986	5.54E-09
rs146803692	rs1324138	chr6:93732110	0.04846218	1	EPHA7	ENSG00000135333.13	Adipose - Subcutaneous	A=0.716	T=0.284	0.273986	5.54E-09
rs146803692	rs1324139	chr6:93732300	0.04846218	1	EPHA7	ENSG00000135333.13	Adipose - Subcutaneous	C=0.716	A=0.284	0.273986	5.54E-09
rs146803692	rs1324140	chr6:93732437	0.04846218	1	EPHA7	ENSG00000135333.13	Adipose - Subcutaneous	T=0.716	C=0.284	0.273986	5.54E-09
rs146803692	rs2476873	chr6:93732800	0.04846218	1	EPHA7	ENSG00000135333.13	Adipose - Subcutaneous	T=0.716	G=0.284	0.273986	5.54E-09
rs146803692	rs2485807	chr6:93732805	0.04846218	1	EPHA7	ENSG00000135333.13	Adipose - Subcutaneous	T=0.716	A=0.284	0.273986	5.54E-09
rs146803692	rs2485808	chr6:93733384	0.04846218	1	EPHA7	ENSG00000135333.13	Adipose - Subcutaneous	T=0.716	C=0.284	0.273986	5.54E-09
rs146803692	rs2506913	chr6:93733402	0.04846218	1	EPHA7	ENSG00000135333.13	Adipose - Subcutaneous	C=0.716	G=0.284	0.273986	5.54E-09
rs146803692	rs2506914	chr6:93733665	0.04846218	1	EPHA7	ENSG00000135333.13	Adipose - Subcutaneous	C=0.716	T=0.284	0.273986	5.54E-09
rs146803692	rs2506915	chr6:93733815	0.04846218	1	EPHA7	ENSG00000135333.13	Adipose - Subcutaneous	C=0.716	T=0.284	0.273986	5.54E-09
rs146803692	rs2506916	chr6:93733967	0.04846218	1	EPHA7	ENSG00000135333.13	Adipose - Subcutaneous	A=0.716	G=0.284	0.273986	5.54E-09
rs146803692	rs2476874	chr6:93734085	0.04869976	1	EPHA7	ENSG00000135333.13	Adipose - Subcutaneous	G=0.717	T=0.283	0.273986	5.54E-09
rs146803692	rs1590391	chr6:93735034	0.04846218	1	EPHA7	ENSG00000135333.13	Adipose - Subcutaneous	T=0.716	C=0.284	0.273986	5.54E-09
rs146803692	rs2476879	chr6:93735793	0.04846218	1	EPHA7	ENSG00000135333.13	Adipose - Subcutaneous	C=0.716	G=0.284	0.273986	5.54E-09
rs146803692	rs2476880	chr6:93735832	0.04846218	1	EPHA7	ENSG00000135333.13	Adipose - Subcutaneous	T=0.716	C=0.284	0.273986	5.54E-09
rs146803692	rs2476881	chr6:93736107	0.04846218	1	EPHA7	ENSG00000135333.13	Adipose - Subcutaneous	A=0.716	G=0.284	0.273986	5.54E-09
rs146803692	rs78019787	chr6:93736390	0.04846218	1	EPHA7	ENSG00000135333.13	Adipose - Subcutaneous	G=0.716	C=0.284	0.273986	5.54E-09
rs146803692	rs2476882	chr6:93736881	0.04846218	1	EPHA7	ENSG00000135333.13	Adipose - Subcutaneous	A=0.716	G=0.284	0.273986	5.54E-09
rs146803692	rs2485809	chr6:93736977	0.04822624	1	EPHA7	ENSG00000135333.13	Adipose - Subcutaneous	T=0.715	C=0.285	0.273986	5.54E-09

rs146803692	rs6454927	chr6:93737184	0.04846218	1	EPHA7	ENSG00000135333.13	Adipose - Subcutaneous	T=0.716	C=0.284	0.273986	5.54E-09
rs146803692	rs6454928	chr6:93737185	0.04846218	1	EPHA7	ENSG00000135333.13	Adipose - Subcutaneous	G=0.716	C=0.284	0.273986	5.54E-09
rs146803692	rs7757592	chr6:93737361	0.04846218	1	EPHA7	ENSG00000135333.13	Adipose - Subcutaneous	T=0.716	G=0.284	0.273986	5.54E-09
rs146803692	rs7775409	chr6:93737401	0.04846218	1	EPHA7	ENSG00000135333.13	Adipose - Subcutaneous	A=0.716	G=0.284	0.273986	5.54E-09
rs146803692	rs2476885	chr6:93737694	0.04846218	1	EPHA7	ENSG00000135333.13	Adipose - Subcutaneous	T=0.716	C=0.284	0.273986	5.54E-09
rs146803692	rs2506919	chr6:93737780	0.04846218	1	EPHA7	ENSG00000135333.13	Adipose - Subcutaneous	C=0.716	T=0.284	0.273986	5.54E-09
rs146803692	rs553697	chr6:93730393	0.00372217	1	EPHA7	ENSG00000135333.13	Skin - Sun Exposed (Lower leg)	T=0.162	C=0.838	0.224058	5.68E-09
rs146803692	rs9452184	chr6:93728200	0.0179201	1	EPHA7	ENSG00000135333.13	Skin - Sun Exposed (Lower leg)	T=0.482	G=0.518	0.176788	6.01E-09
rs146803692	rs60369137	chr6:93735967	0.00691969	1	EPHA7	ENSG00000135333.13	Adipose - Subcutaneous	T=0.736	C=0.264	0.301202	6.31E-09
rs146803692	rs56144268	chr6:93736540	0.0069551	1	EPHA7	ENSG00000135333.13	Adipose - Subcutaneous	A=0.735	G=0.265	0.301202	6.31E-09
rs146803692	rs538373	chr6:93732427	0.00746113	1	EPHA7	ENSG00000135333.13	Nerve - Tibial	G=0.279	A=0.721	0.299304	8.08E-09
rs146803692	rs72928535	chr6:93804189	0.0036678	1	EPHA7	ENSG00000135333.13	Adipose - Subcutaneous	T=0.84	G=0.16	0.373604	9.66E-09
rs146803692	rs3123791	chr6:93742835	0.04846218	1	EPHA7	ENSG00000135333.13	Adipose - Subcutaneous	C=0.716	T=0.284	0.269761	1.00E-08
rs146803692	rs62414138	chr6:93771000	0.00468764	1	EPHA7	ENSG00000135333.13	Adipose - Subcutaneous	G=0.804	A=0.196	0.353497	1.02E-08
rs146803692	rs2506920	chr6:93738058	0.04846218	1	EPHA7	ENSG00000135333.13	Adipose - Subcutaneous	C=0.716	T=0.284	0.269004	1.03E-08
rs146803692	rs2476887	chr6:93738084	0.04846218	1	EPHA7	ENSG00000135333.13	Adipose - Subcutaneous	A=0.716	G=0.284	0.269004	1.03E-08
rs146803692	rs4053544	chr6:93738700	0.04846218	1	EPHA7	ENSG00000135333.13	Adipose - Subcutaneous	TG=0.716	-=0.284	0.269004	1.03E-08
rs146803692	rs2066011	chr6:93738725	0.04846218	1	EPHA7	ENSG00000135333.13	Adipose - Subcutaneous	A=0.716	G=0.284	0.269004	1.03E-08
rs146803692	rs2476889	chr6:93739308	0.04846218	1	EPHA7	ENSG00000135333.13	Adipose - Subcutaneous	C=0.716	T=0.284	0.269004	1.03E-08
rs146803692	rs2476890	chr6:93739465	0.04846218	1	EPHA7	ENSG00000135333.13	Adipose - Subcutaneous	A=0.716	G=0.284	0.269004	1.03E-08
rs146803692	rs2476892	chr6:93739722	0.04846218	1	EPHA7	ENSG00000135333.13	Adipose - Subcutaneous	T=0.716	A=0.284	0.269004	1.03E-08
rs146803692	rs1324142	chr6:93741039	0.04846218	1	EPHA7	ENSG00000135333.13	Adipose - Subcutaneous	T=0.716	G=0.284	0.269004	1.03E-08
rs146803692	rs1324143	chr6:93741213	0.04846218	1	EPHA7	ENSG00000135333.13	Adipose - Subcutaneous	T=0.716	A=0.284	0.269004	1.03E-08
rs146803692	rs2476893	chr6:93741420	0.04846218	1	EPHA7	ENSG00000135333.13	Adipose - Subcutaneous	G=0.716	A=0.284	0.269004	1.03E-08
rs146803692	rs2506921	chr6:93741899	0.04846218	1	EPHA7	ENSG00000135333.13	Adipose - Subcutaneous	A=0.716	G=0.284	0.269004	1.03E-08
rs146803692	rs2485810	chr6:93741903	0.04846218	1	EPHA7	ENSG00000135333.13	Adipose - Subcutaneous	G=0.716	A=0.284	0.269004	1.03E-08
rs146803692	rs2506922	chr6:93741970	0.04846218	1	EPHA7	ENSG00000135333.13	Adipose - Subcutaneous	G=0.716	T=0.284	0.269004	1.03E-08
rs146803692	rs2476850	chr6:93742041	0.04846218	1	EPHA7	ENSG00000135333.13	Adipose - Subcutaneous	G=0.716	A=0.284	0.269004	1.03E-08
rs146803692	rs2506924	chr6:93742108	0.04846218	1	EPHA7	ENSG00000135333.13	Adipose - Subcutaneous	C=0.716	T=0.284	0.269004	1.03E-08
rs146803692	rs2476851	chr6:93742248	0.04822624	1	EPHA7	ENSG00000135333.13	Adipose - Subcutaneous	C=0.715	A=0.285	0.269004	1.03E-08
rs146803692	rs2476852	chr6:93742310	0.04822624	1	EPHA7	ENSG00000135333.13	Adipose - Subcutaneous	G=0.715	A=0.285	0.269004	1.03E-08
rs146803692	rs9445050	chr6:93742909	0.04846218	1	EPHA7	ENSG00000135333.13	Adipose - Subcutaneous	C=0.716	A=0.284	0.269004	1.03E-08
rs146803692	rs9452196	chr6:93743149	0.04846218	1	EPHA7	ENSG00000135333.13	Adipose - Subcutaneous	T=0.716	G=0.284	0.269004	1.03E-08
rs146803692	rs9452197	chr6:93743169	0.04846218	1	EPHA7	ENSG00000135333.13	Adipose - Subcutaneous	A=0.716	G=0.284	0.269004	1.03E-08
rs146803692	rs6454929	chr6:93743651	0.04846218	1	EPHA7	ENSG00000135333.13	Adipose - Subcutaneous	C=0.716	G=0.284	0.269004	1.03E-08
rs146803692	rs6908179	chr6:93744034	0.04846218	1	EPHA7	ENSG00000135333.13	Adipose - Subcutaneous	C=0.716	T=0.284	0.269004	1.03E-08
rs146803692	rs9452204	chr6:93744272	0.04846218	1	EPHA7	ENSG00000135333.13	Adipose - Subcutaneous	A=0.716	G=0.284	0.269004	1.03E-08
rs146803692	rs9452205	chr6:93744365	0.04846218	1	EPHA7	ENSG00000135333.13	Adipose - Subcutaneous	A=0.716	C=0.284	0.269004	1.03E-08
rs146803692	rs9452208	chr6:93745044	0.04846218	1	EPHA7	ENSG00000135333.13	Adipose - Subcutaneous	T=0.716	C=0.284	0.269004	1.03E-08
rs146803692	rs1577970	chr6:93745608	0.04846218	1	EPHA7	ENSG00000135333.13	Adipose - Subcutaneous	T=0.716	A=0.284	0.269004	1.03E-08
rs146803692	rs1924472	chr6:93745615	0.04846218	1	EPHA7	ENSG00000135333.13	Adipose - Subcutaneous	C=0.716	T=0.284	0.269004	1.03E-08
rs146803692	rs1853600	chr6:93746420	0.04846218	1	EPHA7	ENSG00000135333.13	Adipose - Subcutaneous	T=0.716	G=0.284	0.269004	1.03E-08
rs146803692	rs1590386	chr6:93746885	0.04846218	1	EPHA7	ENSG00000135333.13	Adipose - Subcutaneous	G=0.716	T=0.284	0.269004	1.03E-08
rs146803692	rs1590387	chr6:93747142	0.04846218	1	EPHA7	ENSG00000135333.13	Adipose - Subcutaneous	C=0.716	T=0.284	0.269004	1.03E-08
rs146803692	rs1590389	chr6:93747266	0.04846218	1	EPHA7	ENSG00000135333.13	Adipose - Subcutaneous	G=0.716	A=0.284	0.269004	1.03E-08
rs146803692	rs2476859	chr6:93747271	0.04846218	1	EPHA7	ENSG00000135333.13	Adipose - Subcutaneous	G=0.716	A=0.284	0.269004	1.03E-08
rs146803692	rs1547632	chr6:93747562	0.04846218	1	EPHA7	ENSG00000135333.13	Adipose - Subcutaneous	G=0.716	A=0.284	0.269004	1.03E-08
rs146803692	rs1547633	chr6:93747735	0.04846218	1	EPHA7	ENSG00000135333.13	Adipose - Subcutaneous	G=0.716	T=0.284	0.269004	1.03E-08
rs146803692	rs1547634	chr6:93747743	0.04869976	1	EPHA7	ENSG00000135333.13	Adipose - Subcutaneous	A=0.717	T=0.283	0.269004	1.03E-08
rs146803692	rs5878282	chr6:93747748	0.04869976	1	EPHA7	ENSG00000135333.13	Adipose - Subcutaneous	T=0.717	-=0.283	0.269004	1.03E-08
rs146803692	rs2476861	chr6:93747876	0.04846218	1	EPHA7	ENSG00000135333.13	Adipose - Subcutaneous	G=0.716	A=0.284	0.269004	1.03E-08

rs146803692	rs1547739	chr6:93748079	0.04846218	1	EPHA7	ENSG00000135333.13	Adipose - Subcutaneous	C=0.716	T=0.284	0.269004	1.03E-08
rs146803692	rs1338244	chr6:93748547	0.04846218	1	EPHA7	ENSG00000135333.13	Adipose - Subcutaneous	T=0.716	C=0.284	0.269004	1.03E-08
rs146803692	rs1338245	chr6:93748601	0.04846218	1	EPHA7	ENSG00000135333.13	Adipose - Subcutaneous	A=0.716	G=0.284	0.269004	1.03E-08
rs146803692	rs1338246	chr6:93748605	0.04846218	1	EPHA7	ENSG00000135333.13	Adipose - Subcutaneous	C=0.716	A=0.284	0.269004	1.03E-08
rs146803692	rs1338247	chr6:93748638	0.04846218	1	EPHA7	ENSG00000135333.13	Adipose - Subcutaneous	C=0.716	T=0.284	0.269004	1.03E-08
rs146803692	rs2485812	chr6:93747334	0.04846218	1	EPHA7	ENSG00000135333.13	Adipose - Subcutaneous	A=0.716	G=0.284	0.266923	1.10E-08
rs146803692	rs2476862	chr6:93747972	0.04846218	1	EPHA7	ENSG00000135333.13	Adipose - Subcutaneous	T=0.716	C=0.284	0.267609	1.10E-08
rs146803692	rs1575542	chr6:93746652	0.04846218	1	EPHA7	ENSG00000135333.13	Adipose - Subcutaneous	G=0.716	A=0.284	0.267466	1.22E-08
rs146803692	rs189136709	chr6:93783798	9.62E-05	1	EPHA7	ENSG00000135333.13	Brain - Anterior cingulate cortex (BA24)	G=0.995	A=0.005	1.35876	1.60E-08
rs146803692	rs147704337	chr6:93743425	0.05264186	0.93069716	EPHA7	ENSG00000135333.13	Adipose - Subcutaneous	-=0.759	T=0.241	0.262969	2.11E-08
rs146803692	rs62414118	chr6:93748346	0.00492672	1	EPHA7	ENSG00000135333.13	Adipose - Subcutaneous	T=0.796	A=0.204	0.336344	2.22E-08
rs146803692	rs493340	chr6:93756884	0.01082079	1	EPHA7	ENSG00000135333.13	Adipose - Subcutaneous	T=0.64	C=0.36	0.262402	2.26E-08
rs146803692	rs6932844	chr6:93741143	0.00492672	1	EPHA7	ENSG00000135333.13	Adipose - Subcutaneous	A=0.796	G=0.204	0.335026	2.78E-08
rs146803692	rs370852231	chr6:93747736	0.04164835	1	EPHA7	ENSG00000135333.13	Adipose - Subcutaneous	A=0.684	-=0.316	0.255544	3.52E-08
rs146803692	rs72926538	chr6:93754775	0.0040539	1	EPHA7	ENSG00000135333.13	Adipose - Subcutaneous	C=0.826	T=0.174	0.342481	5.10E-08
rs146803692	rs55993443	chr6:93732587	0.00492672	1	EPHA7	ENSG00000135333.13	Adipose - Subcutaneous	A=0.796	C=0.204	0.325709	6.99E-08
rs146803692	rs493340	chr6:93756884	0.01082079	1	EPHA7	ENSG00000135333.13	Lung	T=0.64	C=0.36	0.269696	1.11E-07
rs146803692	rs1386276	chr6:94082749	0.00069388	1	EPHA7	ENSG00000135333.13	Heart - Left Ventricle	A=0.965	G=0.035	-0.463277	1.18E-07
rs146803692	rs79718749	chr6:94098832	0.00069388	1	EPHA7	ENSG00000135333.13	Heart - Left Ventricle	T=0.965	C=0.035	-0.463277	1.18E-07
rs146803692	rs12661215	chr6:94099119	0.00069388	1	EPHA7	ENSG00000135333.13	Heart - Left Ventricle	C=0.965	T=0.035	-0.463277	1.18E-07
rs146803692	rs16871305	chr6:94111173	0.00069388	1	EPHA7	ENSG00000135333.13	Heart - Left Ventricle	A=0.965	T=0.035	-0.463277	1.18E-07
rs146803692	rs80300952	chr6:94115209	0.00069388	1	EPHA7	ENSG00000135333.13	Heart - Left Ventricle	T=0.965	C=0.035	-0.463277	1.18E-07
rs146803692	rs553697	chr6:93730393	0.00372217	1	EPHA7	ENSG00000135333.13	Lung	T=0.162	C=0.838	0.331218	1.59E-07
rs146803692	rs11969676	chr6:93778241	0.19124708	1	EPHA7	ENSG00000135333.13	Skin - Sun Exposed (Lower leg)	G=0.909	A=0.091	0.236616	3.38E-07
rs146803692	rs503319	chr6:93750010	0.00551414	1	EPHA7	ENSG00000135333.13	Adipose - Subcutaneous	A=0.777	T=0.223	0.261009	3.49E-07
rs146803692	rs7769656	chr6:93757068	0.00599845	1	EPHA7	ENSG00000135333.13	Adipose - Subcutaneous	T=0.762	A=0.238	0.249857	6.17E-07
rs146803692	rs1324141	chr6:93736435	0.00471727	1	EPHA7	ENSG00000135333.13	Adipose - Subcutaneous	C=0.803	T=0.197	0.259125	7.88E-07
rs146803692	rs2476883	chr6:93737501	0.00471727	1	EPHA7	ENSG00000135333.13	Adipose - Subcutaneous	T=0.803	G=0.197	0.259125	7.88E-07
rs146803692	rs12191988	chr6:93756336	0.16876678	1	EPHA7	ENSG00000135333.13	Skin - Sun Exposed (Lower leg)	G=0.898	A=0.102	0.223508	7.94E-07
rs146803692	rs5878286	chr6:93823490	0.01244493	1	EPHA7	ENSG00000135333.13	Breast - Mammary Tissue	-=0.393	T=0.607	0.261656	8.30E-07
rs146803692	rs5878287	chr6:93823492	0.01244493	1	EPHA7	ENSG00000135333.13	Breast - Mammary Tissue	A=0.393	-=0.607	0.261656	8.30E-07
rs146803692	rs2476878	chr6:93735281	0.00471727	1	EPHA7	ENSG00000135333.13	Adipose - Subcutaneous	A=0.803	G=0.197	0.25835	8.34E-07
rs146803692	rs2506917	chr6:93736247	0.00471727	1	EPHA7	ENSG00000135333.13	Adipose - Subcutaneous	C=0.803	T=0.197	0.25835	8.34E-07
rs146803692	rs146471836	chr6:93817042	0.00063245	1	EPHA7	ENSG00000135333.13	Adipose - Subcutaneous	C=0.968	T=0.032	-0.617165	8.35E-07
rs146803692	rs10755499	chr6:93774438	0.19124708	1	EPHA7	ENSG00000135333.13	Skin - Sun Exposed (Lower leg)	C=0.909	T=0.091	0.225093	9.89E-07
rs146803692	rs10944643	chr6:93778739	0.19124708	1	EPHA7	ENSG00000135333.13	Skin - Sun Exposed (Lower leg)	C=0.909	T=0.091	0.225093	9.89E-07
rs146803692	rs11963855	chr6:93779831	0.19124708	1	EPHA7	ENSG00000135333.13	Skin - Sun Exposed (Lower leg)	G=0.909	T=0.091	0.225093	9.89E-07
rs146803692	rs16870674	chr6:93780798	0.19124708	1	EPHA7	ENSG00000135333.13	Skin - Sun Exposed (Lower leg)	C=0.909	T=0.091	0.225093	9.89E-07
rs146803692	rs6900435	chr6:93782921	0.19124708	1	EPHA7	ENSG00000135333.13	Skin - Sun Exposed (Lower leg)	G=0.909	A=0.091	0.225093	9.89E-07
rs146803692	rs9294560	chr6:93823182	0.01244493	1	EPHA7	ENSG00000135333.13	Nerve - Tibial	G=0.393	A=0.607	0.215745	9.90E-07
rs146803692	rs13213928	chr6:93778130	0.19124708	1	EPHA7	ENSG00000135333.13	Skin - Sun Exposed (Lower leg)	C=0.909	T=0.091	0.222817	1.03E-06
rs146803692	rs10944640	chr6:93751478	0.16876678	1	EPHA7	ENSG00000135333.13	Skin - Sun Exposed (Lower leg)	C=0.898	G=0.102	0.220789	1.05E-06
rs146803692	rs12189926	chr6:93755282	0.16876678	1	EPHA7	ENSG00000135333.13	Skin - Sun Exposed (Lower leg)	G=0.898	A=0.102	0.220789	1.05E-06
rs146803692	rs12190002	chr6:93755421	0.16876678	1	EPHA7	ENSG00000135333.13	Skin - Sun Exposed (Lower leg)	G=0.898	T=0.102	0.220789	1.05E-06
rs146803692	rs12190060	chr6:93755507	0.16876678	1	EPHA7	ENSG00000135333.13	Skin - Sun Exposed (Lower leg)	G=0.898	A=0.102	0.220789	1.05E-06
rs146803692	rs12198368	chr6:93761490	0.16876678	1	EPHA7	ENSG00000135333.13	Skin - Sun Exposed (Lower leg)	T=0.898	C=0.102	0.220789	1.05E-06
rs146803692	rs9445060	chr6:93754781	0.01813537	1	EPHA7	ENSG00000135333.13	Lung	A=0.485	G=0.515	0.224721	1.17E-06
rs146803692	rs9452216	chr6:93753484	0.00603141	1	EPHA7	ENSG00000135333.13	Adipose - Subcutaneous	T=0.761	A=0.239	0.248192	1.18E-06
rs146803692	rs9445061	chr6:93755285	0.00599845	1	EPHA7	ENSG00000135333.13	Adipose - Subcutaneous	T=0.762	C=0.238	0.248192	1.18E-06
rs146803692	rs9445063	chr6:93755610	0.00599845	1	EPHA7	ENSG00000135333.13	Adipose - Subcutaneous	T=0.762	C=0.238	0.248192	1.18E-06
rs146803692	rs75887019	chr6:93776052	0.19124708	1	EPHA7	ENSG00000135333.13	Skin - Sun Exposed (Lower leg)	T=0.909	C=0.091	0.224265	1.19E-06

rs146803692	rs11970484	chr6:93779586	0.19124708	1	EPHA7	ENSG00000135333.13	Skin - Sun Exposed (Lower leg)	G=0.909	A=0.091	0.224265	1.19E-06
rs146803692	rs13213427	chr6:93781422	0.19124708	1	EPHA7	ENSG00000135333.13	Skin - Sun Exposed (Lower leg)	A=0.909	G=0.091	0.224265	1.19E-06
rs146803692	rs35249597	chr6:93788631	0.19124708	1	EPHA7	ENSG00000135333.13	Skin - Sun Exposed (Lower leg)	A=0.909	G=0.091	0.224265	1.19E-06
rs146803692	rs16870689	chr6:93791845	0.19124708	1	EPHA7	ENSG00000135333.13	Skin - Sun Exposed (Lower leg)	C=0.909	G=0.091	0.224265	1.19E-06
rs146803692	rs553697	chr6:93730393	0.00372217	1	EPHA7	ENSG00000135333.13	Nerve - Tibial	T=0.162	C=0.838	0.287097	1.21E-06
rs146803692	rs1986173	chr6:93756502	0.03639847	1	EPHA7	ENSG00000135333.13	Skin - Sun Exposed (Lower leg)	A=0.654	G=0.346	0.144424	1.21E-06
rs146803692	rs12192013	chr6:93756561	0.16876678	1	EPHA7	ENSG00000135333.13	Skin - Sun Exposed (Lower leg)	C=0.898	T=0.102	0.217218	1.26E-06
rs146803692	rs5878286	chr6:93823490	0.01244493	1	EPHA7	ENSG00000135333.13	Nerve - Tibial	-=0.393	T=0.607	0.213244	1.33E-06
rs146803692	rs5878287	chr6:93823492	0.01244493	1	EPHA7	ENSG00000135333.13	Nerve - Tibial	A=0.393	-=0.607	0.213244	1.33E-06
rs146803692	rs9294560	chr6:93823182	0.01244493	1	EPHA7	ENSG00000135333.13	Breast - Mammary Tissue	G=0.393	A=0.607	0.25659	1.36E-06
rs146803692	rs2181806	chr6:93752150	0.16876678	1	EPHA7	ENSG00000135333.13	Skin - Sun Exposed (Lower leg)	C=0.898	A=0.102	0.216342	1.36E-06
rs146803692	rs9452223	chr6:93756793	0.16695893	1	EPHA7	ENSG00000135333.13	Skin - Sun Exposed (Lower leg)	A=0.897	G=0.103	0.210167	1.52E-06
rs146803692	rs490474	chr6:93728105	0.01402424	1	EPHA7	ENSG00000135333.13	Adipose - Subcutaneous	C=0.421	T=0.579	0.219192	1.52E-06
rs146803692	rs9452228	chr6:93770995	0.18898367	1	EPHA7	ENSG00000135333.13	Skin - Sun Exposed (Lower leg)	A=0.908	G=0.092	0.219991	1.53E-06
rs146803692	rs1324114	chr6:93793076	0.18898367	1	EPHA7	ENSG00000135333.13	Skin - Sun Exposed (Lower leg)	C=0.908	T=0.092	0.219991	1.53E-06
rs146803692	rs1324113	chr6:93793350	0.18898367	1	EPHA7	ENSG00000135333.13	Skin - Sun Exposed (Lower leg)	A=0.908	T=0.092	0.219991	1.53E-06
rs146803692	rs782702	chr6:93924511	0.00906225	1	EPHA7	ENSG00000135333.13	Adipose - Subcutaneous	G=0.32	T=0.68	0.227213	1.58E-06
rs146803692	rs5878306	chr6:93943683	0.00914528	1	EPHA7	ENSG00000135333.13	Adipose - Subcutaneous	T=0.322	-=0.678	0.227213	1.58E-06
rs146803692	rs62414137	chr6:93766182	0.16695893	1	EPHA7	ENSG00000135333.13	Skin - Sun Exposed (Lower leg)	A=0.897	G=0.103	0.209936	1.60E-06
rs146803692	rs13196582	chr6:93774046	0.01514718	1	EPHA7	ENSG00000135333.13	Lung	C=0.56	T=0.44	0.220459	1.65E-06
rs146803692	rs141174921	chr6:93733454	0.00063245	1	EPHA7	ENSG00000135333.13	Adipose - Subcutaneous	A=0.968	G=0.032	-0.598481	1.67E-06
rs146803692	rs9452217	chr6:93755699	0.16695893	1	EPHA7	ENSG00000135333.13	Skin - Sun Exposed (Lower leg)	T=0.897	C=0.103	0.212714	1.69E-06
rs146803692	rs9445064	chr6:93756123	0.16695893	1	EPHA7	ENSG00000135333.13	Skin - Sun Exposed (Lower leg)	T=0.897	C=0.103	0.212714	1.69E-06
rs146803692	rs9363051	chr6:93945160	0.00910371	1	EPHA7	ENSG00000135333.13	Adipose - Subcutaneous	T=0.321	C=0.679	0.225403	1.76E-06
rs146803692	rs11967054	chr6:93769206	0.18898367	1	EPHA7	ENSG00000135333.13	Skin - Sun Exposed (Lower leg)	C=0.908	T=0.092	0.218174	1.80E-06
rs146803692	rs12211721	chr6:93805409	0.19124708	1	EPHA7	ENSG00000135333.13	Skin - Sun Exposed (Lower leg)	A=0.909	T=0.091	0.219942	1.91E-06
rs146803692	rs12198413	chr6:93805574	0.19124708	1	EPHA7	ENSG00000135333.13	Skin - Sun Exposed (Lower leg)	C=0.909	T=0.091	0.21909	1.98E-06
rs146803692	rs1359241	chr6:93779417	0.01514718	1	EPHA7	ENSG00000135333.13	Lung	A=0.56	C=0.44	0.218391	2.02E-06
rs146803692	rs9452238	chr6:93788831	0.01551771	1	EPHA7	ENSG00000135333.13	Lung	A=0.554	G=0.446	0.218391	2.02E-06
rs146803692	rs10485370	chr6:93749598	0.20081514	1	EPHA7	ENSG00000135333.13	Skin - Sun Exposed (Lower leg)	G=0.913	C=0.087	0.23839	2.19E-06
rs146803692	rs62414098	chr6:93734692	0.20081514	1	EPHA7	ENSG00000135333.13	Skin - Sun Exposed (Lower leg)	C=0.913	T=0.087	0.246385	2.19E-06
rs146803692	rs370852231	chr6:93747736	0.04164835	1	EPHA7	ENSG00000135333.13	Skin - Sun Exposed (Lower leg)	A=0.684	-=0.316	0.145604	2.44E-06
rs146803692	rs1486756	chr6:93942185	0.00910371	1	EPHA7	ENSG00000135333.13	Adipose - Subcutaneous	A=0.321	G=0.679	0.223122	2.44E-06
rs146803692	rs145341859	chr6:93739275	0.20081514	1	EPHA7	ENSG00000135333.13	Skin - Sun Exposed (Lower leg)	C=0.913	-=0.087	0.243951	2.47E-06
rs146803692	rs144702817	chr6:93744190	0.20081514	1	EPHA7	ENSG00000135333.13	Skin - Sun Exposed (Lower leg)	C=0.913	T=0.087	0.243951	2.47E-06
rs146803692	rs12193996	chr6:93748696	0.20081514	1	EPHA7	ENSG00000135333.13	Skin - Sun Exposed (Lower leg)	T=0.913	G=0.087	0.243951	2.47E-06
rs146803692	rs1408296	chr6:93749165	0.20081514	1	EPHA7	ENSG00000135333.13	Skin - Sun Exposed (Lower leg)	G=0.913	A=0.087	0.243951	2.47E-06
rs146803692	rs117376030	chr6:94203683	0.00051072	1	EPHA7	ENSG00000135333.13	Esophagus - Gastroesophageal Junction	T=0.974	A=0.026	-0.384156	2.51E-06
rs146803692	rs11969246	chr6:93823113	0.01551771	1	EPHA7	ENSG00000135333.13	Lung	G=0.554	T=0.446	0.216285	2.55E-06
rs146803692	rs9445062	chr6:93755554	0.03639847	1	EPHA7	ENSG00000135333.13	Skin - Sun Exposed (Lower leg)	G=0.654	A=0.346	0.139172	2.98E-06
rs146803692	rs1014573	chr6:93756934	0.03655884	1	EPHA7	ENSG00000135333.13	Skin - Sun Exposed (Lower leg)	C=0.655	T=0.345	0.139172	2.98E-06
rs146803692	rs78837643	chr6:93824537	0.00061206	1	EPHA7	ENSG00000135333.13	Adipose - Subcutaneous	A=0.969	G=0.031	-0.592603	3.00E-06
rs146803692	rs9452240	chr6:93806820	0.01551771	1	EPHA7	ENSG00000135333.13	Lung	G=0.554	T=0.446	0.214812	3.00E-06
rs146803692	rs7739743	chr6:93813381	0.01551771	1	EPHA7	ENSG00000135333.13	Lung	G=0.554	A=0.446	0.214812	3.00E-06
rs146803692	rs9445075	chr6:93822458	0.01551771	1	EPHA7	ENSG00000135333.13	Lung	T=0.554	A=0.446	0.214812	3.00E-06
rs146803692	rs6921387	chr6:93804582	0.18898367	1	EPHA7	ENSG00000135333.13	Skin - Sun Exposed (Lower leg)	A=0.908	G=0.092	0.214152	3.01E-06
rs146803692	rs12209396	chr6:93807569	0.18898367	1	EPHA7	ENSG00000135333.13	Skin - Sun Exposed (Lower leg)	T=0.908	C=0.092	0.214152	3.01E-06
rs146803692	rs16871170	chr6:94041109	0.00130787	1	EPHA7	ENSG00000135333.13	Heart - Left Ventricle	C=0.936	T=0.064	-0.297607	3.05E-06
rs146803692	rs12190474	chr6:93773303	0.19124708	1	EPHA7	ENSG00000135333.13	Skin - Sun Exposed (Lower leg)	A=0.909	G=0.091	0.227595	3.17E-06
rs146803692	rs12190476	chr6:93773315	0.19124708	1	EPHA7	ENSG00000135333.13	Skin - Sun Exposed (Lower leg)	A=0.909	T=0.091	0.227595	3.17E-06
rs146803692	rs67916544	chr6:93783688	0.19356024	1	EPHA7	ENSG00000135333.13	Skin - Sun Exposed (Lower leg)	G=0.91	-=0.09	0.227595	3.17E-06

rs146803692	rs6454938	chr6:93804296	0.19124708	1	EPHA7	ENSG00000135333.13	Skin - Sun Exposed (Lower leg)	T=0.909	C=0.091	0.227595	3.17E-06
rs146803692	rs551175	chr6:93765182	0.06643893	1	EPHA7	ENSG00000135333.13	Skin - Sun Exposed (Lower leg)	G=0.775	A=0.225	0.157974	3.52E-06
rs146803692	rs6454936	chr6:93768526	0.01508619	1	EPHA7	ENSG00000135333.13	Lung	C=0.561	T=0.439	0.212947	3.53E-06
rs146803692	rs727998	chr6:93745434	0.03873105	1	EPHA7	ENSG00000135333.13	Skin - Sun Exposed (Lower leg)	T=0.668	A=0.332	0.140912	3.99E-06
rs146803692	rs1590388	chr6:93747226	0.03873105	1	EPHA7	ENSG00000135333.13	Skin - Sun Exposed (Lower leg)	T=0.668	C=0.332	0.140912	3.99E-06
rs146803692	rs1590392	chr6:93735074	0.03873105	1	EPHA7	ENSG00000135333.13	Skin - Sun Exposed (Lower leg)	T=0.668	G=0.332	0.140806	4.02E-06
rs146803692	rs9452215	chr6:93753233	0.15841722	1	EPHA7	ENSG00000135333.13	Skin - Sun Exposed (Lower leg)	A=0.892	G=0.108	0.20076	4.11E-06
rs146803692	rs2476891	chr6:93739608	0.03873105	1	EPHA7	ENSG00000135333.13	Skin - Sun Exposed (Lower leg)	G=0.668	A=0.332	0.140828	4.46E-06
rs146803692	rs2506923	chr6:93742094	0.03873105	1	EPHA7	ENSG00000135333.13	Skin - Sun Exposed (Lower leg)	A=0.668	G=0.332	0.140828	4.46E-06
rs146803692	rs3123791	chr6:93742835	0.04846218	1	EPHA7	ENSG00000135333.13	Skin - Sun Exposed (Lower leg)	C=0.716	T=0.284	0.145207	4.48E-06
rs146803692	rs377586702	chr6:93736162	0.03925656	1	EPHA7	ENSG00000135333.13	Skin - Sun Exposed (Lower leg)	-=0.671	AAG=0.329	0.14071	4.49E-06
rs146803692	rs72928597	chr6:93839843	0.0016857	1	EPHA7	ENSG00000135333.13	Lung	A=0.919	G=0.081	0.42955	4.55E-06
rs146803692	rs16870853	chr6:93841320	0.0016857	1	EPHA7	ENSG00000135333.13	Lung	T=0.919	C=0.081	0.42955	4.55E-06
rs146803692	rs1930933	chr6:93842544	0.0082189	1	EPHA7	ENSG00000135333.13	Minor Salivary Gland	C=0.299	T=0.701	0.246092	4.56E-06
rs146803692	rs1590390	chr6:93734963	0.04846218	1	EPHA7	ENSG00000135333.13	Skin - Sun Exposed (Lower leg)	G=0.716	A=0.284	0.144763	4.59E-06
rs146803692	rs142723671	chr6:94076414	0.00043039	1	EPHA7	ENSG00000135333.13	Heart - Left Ventricle	G=0.978	C=0.022	-0.495017	4.73E-06
rs146803692	rs4626392	chr6:93944519	0.00910371	1	EPHA7	ENSG00000135333.13	Adipose - Subcutaneous	G=0.321	A=0.679	0.215581	5.04E-06
rs146803692	rs5878286	chr6:93823490	0.01244493	1	EPHA7	ENSG00000135333.13	Skin - Not Sun Exposed (Suprapubic)	-=0.393	T=0.607	0.136533	5.41E-06
rs146803692	rs5878287	chr6:93823492	0.01244493	1	EPHA7	ENSG00000135333.13	Skin - Not Sun Exposed (Suprapubic)	A=0.393	-=0.607	0.136533	5.41E-06
rs146803692	rs2476862	chr6:93747972	0.04846218	1	EPHA7	ENSG00000135333.13	Skin - Sun Exposed (Lower leg)	T=0.716	C=0.284	0.142297	5.72E-06
rs146803692	rs72926509	chr6:93713733	0.00122095	1	EPHA7	ENSG00000135333.13	Skin - Sun Exposed (Lower leg)	G=0.94	A=0.06	-0.286935	5.75E-06
rs146803692	rs9452225	chr6:93766609	0.01402424	1	EPHA7	ENSG00000135333.13	Lung	C=0.579	A=0.421	0.208547	6.08E-06
rs146803692	rs149183077	chr6:93760024	0.00061206	1	EPHA7	ENSG00000135333.13	Adipose - Subcutaneous	T=0.969	G=0.031	-0.577593	6.18E-06
rs146803692	rs2506910	chr6:93730374	0.04846218	1	EPHA7	ENSG00000135333.13	Skin - Sun Exposed (Lower leg)	A=0.716	G=0.284	0.142422	6.19E-06
rs146803692	rs2485806	chr6:93731257	0.04846218	1	EPHA7	ENSG00000135333.13	Skin - Sun Exposed (Lower leg)	G=0.716	A=0.284	0.142422	6.19E-06
rs146803692	rs2506911	chr6:93731302	0.04846218	1	EPHA7	ENSG00000135333.13	Skin - Sun Exposed (Lower leg)	A=0.716	G=0.284	0.142422	6.19E-06
rs146803692	rs1324138	chr6:93732110	0.04846218	1	EPHA7	ENSG00000135333.13	Skin - Sun Exposed (Lower leg)	A=0.716	T=0.284	0.142422	6.19E-06
rs146803692	rs1324139	chr6:93732300	0.04846218	1	EPHA7	ENSG00000135333.13	Skin - Sun Exposed (Lower leg)	C=0.716	A=0.284	0.142422	6.19E-06
rs146803692	rs1324140	chr6:93732437	0.04846218	1	EPHA7	ENSG00000135333.13	Skin - Sun Exposed (Lower leg)	T=0.716	C=0.284	0.142422	6.19E-06
rs146803692	rs2476873	chr6:93732800	0.04846218	1	EPHA7	ENSG00000135333.13	Skin - Sun Exposed (Lower leg)	T=0.716	G=0.284	0.142422	6.19E-06
rs146803692	rs2485807	chr6:93732805	0.04846218	1	EPHA7	ENSG00000135333.13	Skin - Sun Exposed (Lower leg)	T=0.716	A=0.284	0.142422	6.19E-06
rs146803692	rs2485808	chr6:93733384	0.04846218	1	EPHA7	ENSG00000135333.13	Skin - Sun Exposed (Lower leg)	T=0.716	C=0.284	0.142422	6.19E-06
rs146803692	rs2506913	chr6:93733402	0.04846218	1	EPHA7	ENSG00000135333.13	Skin - Sun Exposed (Lower leg)	C=0.716	G=0.284	0.142422	6.19E-06
rs146803692	rs2506914	chr6:93733665	0.04846218	1	EPHA7	ENSG00000135333.13	Skin - Sun Exposed (Lower leg)	C=0.716	T=0.284	0.142422	6.19E-06
rs146803692	rs2506915	chr6:93733815	0.04846218	1	EPHA7	ENSG00000135333.13	Skin - Sun Exposed (Lower leg)	C=0.716	T=0.284	0.142422	6.19E-06
rs146803692	rs2506916	chr6:93733967	0.04846218	1	EPHA7	ENSG00000135333.13	Skin - Sun Exposed (Lower leg)	A=0.716	G=0.284	0.142422	6.19E-06
rs146803692	rs2476874	chr6:93734085	0.04869976	1	EPHA7	ENSG00000135333.13	Skin - Sun Exposed (Lower leg)	G=0.717	T=0.283	0.142422	6.19E-06
rs146803692	rs1590391	chr6:93735034	0.04846218	1	EPHA7	ENSG00000135333.13	Skin - Sun Exposed (Lower leg)	T=0.716	C=0.284	0.142422	6.19E-06
rs146803692	rs2476879	chr6:93735793	0.04846218	1	EPHA7	ENSG00000135333.13	Skin - Sun Exposed (Lower leg)	C=0.716	G=0.284	0.142422	6.19E-06
rs146803692	rs2476880	chr6:93735832	0.04846218	1	EPHA7	ENSG00000135333.13	Skin - Sun Exposed (Lower leg)	T=0.716	C=0.284	0.142422	6.19E-06
rs146803692	rs2476881	chr6:93736107	0.04846218	1	EPHA7	ENSG00000135333.13	Skin - Sun Exposed (Lower leg)	A=0.716	G=0.284	0.142422	6.19E-06
rs146803692	rs78019787	chr6:93736390	0.04846218	1	EPHA7	ENSG00000135333.13	Skin - Sun Exposed (Lower leg)	G=0.716	C=0.284	0.142422	6.19E-06
rs146803692	rs2485809	chr6:93736977	0.04822624	1	EPHA7	ENSG00000135333.13	Skin - Sun Exposed (Lower leg)	T=0.715	C=0.285	0.142422	6.19E-06
rs146803692	rs6454927	chr6:93737184	0.04846218	1	EPHA7	ENSG00000135333.13	Skin - Sun Exposed (Lower leg)	T=0.716	C=0.284	0.142422	6.19E-06
rs146803692	rs6454928	chr6:93737185	0.04846218	1	EPHA7	ENSG00000135333.13	Skin - Sun Exposed (Lower leg)	G=0.716	C=0.284	0.142422	6.19E-06
rs146803692	rs7757592	chr6:93737361	0.04846218	1	EPHA7	ENSG00000135333.13	Skin - Sun Exposed (Lower leg)	T=0.716	G=0.284	0.142422	6.19E-06
rs146803692	rs7775409	chr6:93737401	0.04846218	1	EPHA7	ENSG00000135333.13	Skin - Sun Exposed (Lower leg)	A=0.716	G=0.284	0.142422	6.19E-06
rs146803692	rs2476885	chr6:93737694	0.04846218	1	EPHA7	ENSG00000135333.13	Skin - Sun Exposed (Lower leg)	T=0.716	C=0.284	0.142422	6.19E-06
rs146803692	rs2506919	chr6:93737780	0.04846218	1	EPHA7	ENSG00000135333.13	Skin - Sun Exposed (Lower leg)	C=0.716	T=0.284	0.142422	6.19E-06
rs146803692	rs2506920	chr6:93738058	0.04846218	1	EPHA7	ENSG00000135333.13	Skin - Sun Exposed (Lower leg)	C=0.716	T=0.284	0.14236	6.23E-06
rs146803692	rs2476887	chr6:93738084	0.04846218	1	EPHA7	ENSG00000135333.13	Skin - Sun Exposed (Lower leg)	A=0.716	G=0.284	0.14236	6.23E-06

rs146803692	rs4053544	chr6:93738700	0.04846218	1	EPHA7	ENSG00000135333.13	Skin - Sun Exposed (Lower leg)	TG=0.716	-=0.284	0.14236	6.23E-06
rs146803692	rs2066011	chr6:93738725	0.04846218	1	EPHA7	ENSG00000135333.13	Skin - Sun Exposed (Lower leg)	A=0.716	G=0.284	0.14236	6.23E-06
rs146803692	rs2476889	chr6:93739308	0.04846218	1	EPHA7	ENSG00000135333.13	Skin - Sun Exposed (Lower leg)	C=0.716	T=0.284	0.14236	6.23E-06
rs146803692	rs2476890	chr6:93739465	0.04846218	1	EPHA7	ENSG00000135333.13	Skin - Sun Exposed (Lower leg)	A=0.716	G=0.284	0.14236	6.23E-06
rs146803692	rs2476892	chr6:93739722	0.04846218	1	EPHA7	ENSG00000135333.13	Skin - Sun Exposed (Lower leg)	T=0.716	A=0.284	0.14236	6.23E-06
rs146803692	rs1324142	chr6:93741039	0.04846218	1	EPHA7	ENSG00000135333.13	Skin - Sun Exposed (Lower leg)	T=0.716	G=0.284	0.14236	6.23E-06
rs146803692	rs1324143	chr6:93741213	0.04846218	1	EPHA7	ENSG00000135333.13	Skin - Sun Exposed (Lower leg)	T=0.716	A=0.284	0.14236	6.23E-06
rs146803692	rs2476893	chr6:93741420	0.04846218	1	EPHA7	ENSG00000135333.13	Skin - Sun Exposed (Lower leg)	G=0.716	A=0.284	0.14236	6.23E-06
rs146803692	rs2506921	chr6:93741899	0.04846218	1	EPHA7	ENSG00000135333.13	Skin - Sun Exposed (Lower leg)	A=0.716	G=0.284	0.14236	6.23E-06
rs146803692	rs2485810	chr6:93741903	0.04846218	1	EPHA7	ENSG00000135333.13	Skin - Sun Exposed (Lower leg)	G=0.716	A=0.284	0.14236	6.23E-06
rs146803692	rs2506922	chr6:93741970	0.04846218	1	EPHA7	ENSG00000135333.13	Skin - Sun Exposed (Lower leg)	G=0.716	T=0.284	0.14236	6.23E-06
rs146803692	rs2476850	chr6:93742041	0.04846218	1	EPHA7	ENSG00000135333.13	Skin - Sun Exposed (Lower leg)	G=0.716	A=0.284	0.14236	6.23E-06
rs146803692	rs2506924	chr6:93742108	0.04846218	1	EPHA7	ENSG00000135333.13	Skin - Sun Exposed (Lower leg)	C=0.716	T=0.284	0.14236	6.23E-06
rs146803692	rs2476851	chr6:93742248	0.04822624	1	EPHA7	ENSG00000135333.13	Skin - Sun Exposed (Lower leg)	C=0.715	A=0.285	0.14236	6.23E-06
rs146803692	rs2476852	chr6:93742310	0.04822624	1	EPHA7	ENSG00000135333.13	Skin - Sun Exposed (Lower leg)	G=0.715	A=0.285	0.14236	6.23E-06
rs146803692	rs9445050	chr6:93742909	0.04846218	1	EPHA7	ENSG00000135333.13	Skin - Sun Exposed (Lower leg)	C=0.716	A=0.284	0.14236	6.23E-06
rs146803692	rs9452196	chr6:93743149	0.04846218	1	EPHA7	ENSG00000135333.13	Skin - Sun Exposed (Lower leg)	T=0.716	G=0.284	0.14236	6.23E-06
rs146803692	rs9452197	chr6:93743169	0.04846218	1	EPHA7	ENSG00000135333.13	Skin - Sun Exposed (Lower leg)	A=0.716	G=0.284	0.14236	6.23E-06
rs146803692	rs6454929	chr6:93743651	0.04846218	1	EPHA7	ENSG00000135333.13	Skin - Sun Exposed (Lower leg)	C=0.716	G=0.284	0.14236	6.23E-06
rs146803692	rs6908179	chr6:93744034	0.04846218	1	EPHA7	ENSG00000135333.13	Skin - Sun Exposed (Lower leg)	C=0.716	T=0.284	0.14236	6.23E-06
rs146803692	rs9452204	chr6:93744272	0.04846218	1	EPHA7	ENSG00000135333.13	Skin - Sun Exposed (Lower leg)	A=0.716	G=0.284	0.14236	6.23E-06
rs146803692	rs9452205	chr6:93744365	0.04846218	1	EPHA7	ENSG00000135333.13	Skin - Sun Exposed (Lower leg)	A=0.716	C=0.284	0.14236	6.23E-06
rs146803692	rs1577970	chr6:93745608	0.04846218	1	EPHA7	ENSG00000135333.13	Skin - Sun Exposed (Lower leg)	T=0.716	A=0.284	0.14236	6.23E-06
rs146803692	rs1924472	chr6:93745615	0.04846218	1	EPHA7	ENSG00000135333.13	Skin - Sun Exposed (Lower leg)	C=0.716	T=0.284	0.14236	6.23E-06
rs146803692	rs1853600	chr6:93746420	0.04846218	1	EPHA7	ENSG00000135333.13	Skin - Sun Exposed (Lower leg)	T=0.716	G=0.284	0.14236	6.23E-06
rs146803692	rs1575542	chr6:93746652	0.04846218	1	EPHA7	ENSG00000135333.13	Skin - Sun Exposed (Lower leg)	G=0.716	A=0.284	0.14236	6.23E-06
rs146803692	rs1590386	chr6:93746885	0.04846218	1	EPHA7	ENSG00000135333.13	Skin - Sun Exposed (Lower leg)	G=0.716	T=0.284	0.14236	6.23E-06
rs146803692	rs1590387	chr6:93747142	0.04846218	1	EPHA7	ENSG00000135333.13	Skin - Sun Exposed (Lower leg)	C=0.716	T=0.284	0.14236	6.23E-06
rs146803692	rs1590389	chr6:93747266	0.04846218	1	EPHA7	ENSG00000135333.13	Skin - Sun Exposed (Lower leg)	G=0.716	A=0.284	0.14236	6.23E-06
rs146803692	rs2476859	chr6:93747271	0.04846218	1	EPHA7	ENSG00000135333.13	Skin - Sun Exposed (Lower leg)	G=0.716	A=0.284	0.14236	6.23E-06
rs146803692	rs1547632	chr6:93747562	0.04846218	1	EPHA7	ENSG00000135333.13	Skin - Sun Exposed (Lower leg)	G=0.716	A=0.284	0.14236	6.23E-06
rs146803692	rs1547633	chr6:93747735	0.04846218	1	EPHA7	ENSG00000135333.13	Skin - Sun Exposed (Lower leg)	G=0.716	T=0.284	0.14236	6.23E-06
rs146803692	rs1547634	chr6:93747743	0.04869976	1	EPHA7	ENSG00000135333.13	Skin - Sun Exposed (Lower leg)	A=0.717	T=0.283	0.14236	6.23E-06
rs146803692	rs5878282	chr6:93747748	0.04869976	1	EPHA7	ENSG00000135333.13	Skin - Sun Exposed (Lower leg)	T=0.717	-=0.283	0.14236	6.23E-06
rs146803692	rs2476861	chr6:93747876	0.04846218	1	EPHA7	ENSG00000135333.13	Skin - Sun Exposed (Lower leg)	G=0.716	A=0.284	0.14236	6.23E-06
rs146803692	rs1547739	chr6:93748079	0.04846218	1	EPHA7	ENSG00000135333.13	Skin - Sun Exposed (Lower leg)	C=0.716	T=0.284	0.14236	6.23E-06
rs146803692	rs1338244	chr6:93748547	0.04846218	1	EPHA7	ENSG00000135333.13	Skin - Sun Exposed (Lower leg)	T=0.716	C=0.284	0.14236	6.23E-06
rs146803692	rs1338245	chr6:93748601	0.04846218	1	EPHA7	ENSG00000135333.13	Skin - Sun Exposed (Lower leg)	A=0.716	G=0.284	0.14236	6.23E-06
rs146803692	rs1338246	chr6:93748605	0.04846218	1	EPHA7	ENSG00000135333.13	Skin - Sun Exposed (Lower leg)	C=0.716	A=0.284	0.14236	6.23E-06
rs146803692	rs1338247	chr6:93748638	0.04846218	1	EPHA7	ENSG00000135333.13	Skin - Sun Exposed (Lower leg)	C=0.716	T=0.284	0.14236	6.23E-06
rs146803692	rs2476886	chr6:93737850	0.04846218	1	EPHA7	ENSG00000135333.13	Skin - Sun Exposed (Lower leg)	G=0.716	A=0.284	0.141896	6.25E-06
rs146803692	rs147330775	chr6:93915683	0.0001155	1	EPHA7	ENSG00000135333.13	Brain - Anterior cingulate cortex (BA24)	C=0.994	T=0.006	0.808887	6.34E-06
rs146803692	rs12199651	chr6:93811308	0.19124708	1	EPHA7	ENSG00000135333.13	Skin - Sun Exposed (Lower leg)	C=0.909	T=0.091	0.220844	6.40E-06
rs146803692	rs28477223	chr6:93817230	0.19124708	1	EPHA7	ENSG00000135333.13	Skin - Sun Exposed (Lower leg)	A=0.909	G=0.091	0.220844	6.40E-06
rs146803692	rs12214823	chr6:93819205	0.19124708	1	EPHA7	ENSG00000135333.13	Skin - Sun Exposed (Lower leg)	C=0.909	T=0.091	0.220844	6.40E-06
rs146803692	rs2485812	chr6:93747334	0.04846218	1	EPHA7	ENSG00000135333.13	Skin - Sun Exposed (Lower leg)	A=0.716	G=0.284	0.141234	6.46E-06
rs146803692	rs16870793	chr6:93828599	0.00164053	1	EPHA7	ENSG00000135333.13	Lung	G=0.921	A=0.079	0.419095	6.62E-06
rs146803692	rs72928592	chr6:93837959	0.00166309	1	EPHA7	ENSG00000135333.13	Lung	T=0.92	C=0.08	0.419095	6.62E-06
rs146803692	rs9294560	chr6:93823182	0.01244493	1	EPHA7	ENSG00000135333.13	Skin - Not Sun Exposed (Suprapubic)	G=0.393	A=0.607	0.135178	6.85E-06
rs146803692	rs9452208	chr6:93745044	0.04846218	1	EPHA7	ENSG00000135333.13	Skin - Sun Exposed (Lower leg)	T=0.716	C=0.284	0.141348	7.36E-06
rs146803692	rs141173439	chr6:93700379	0.00255803	1	EPHA7	ENSG00000135333.13	Skin - Sun Exposed (Lower leg)	G=0.883	T=0.117	-0.222079	7.46E-06

rs146803692	rs12211507	chr6:93717031	0.00255803	1	EPHA7	ENSG00000135333.13	Skin - Sun Exposed (Lower leg)	C=0.883	A=0.117	-0.222079	7.46E-06
rs146803692	rs2476884	chr6:93737618	0.04846218	1	EPHA7	ENSG00000135333.13	Skin - Sun Exposed (Lower leg)	T=0.716	A=0.284	0.141305	7.66E-06
rs146803692	rs1575540	chr6:93836577	0.00117776	1	EPHA7	ENSG00000135333.13	Esophagus - Muscularis	G=0.942	A=0.058	0.272463	7.68E-06
rs146803692	rs544639807	chr6:93883657	0.00015431	1	EPHA7	ENSG00000135333.13	Minor Salivary Gland	G=0.992	A=0.008	-1.34812	8.25E-06
rs146803692	rs36114673	chr6:93847056	0.00126432	1	EPHA7	ENSG00000135333.13	Esophagus - Muscularis	A=0.938	T=0.062	0.268662	8.26E-06
rs146803692	rs7776099	chr6:93913423	0.00380422	1	EPHA7	ENSG00000135333.13	Testis	C=0.835	A=0.165	0.3716	8.26E-06
rs146803692	rs7758242	chr6:93913435	0.00380422	1	EPHA7	ENSG00000135333.13	Testis	T=0.835	A=0.165	0.3716	8.26E-06
rs146803692	rs66765303	chr6:93913692	0.00380422	1	EPHA7	ENSG00000135333.13	Testis	A=0.835	G=0.165	0.3716	8.26E-06
rs146803692	rs66522431	chr6:93913825	0.00380422	1	EPHA7	ENSG00000135333.13	Testis	G=0.835	A=0.165	0.3716	8.26E-06
rs146803692	rs62414181	chr6:93913909	0.00380422	1	EPHA7	ENSG00000135333.13	Testis	A=0.835	G=0.165	0.3716	8.26E-06
rs146803692	rs62414182	chr6:93914022	0.00380422	1	EPHA7	ENSG00000135333.13	Testis	A=0.835	G=0.165	0.3716	8.26E-06
rs146803692	rs113888577	chr6:93698093	0.0002324	1	EPHA7	ENSG00000135333.13	Brain - Anterior cingulate cortex (BA24)	C=0.988	T=0.012	0.66365	8.36E-06
rs146803692	rs12214457	chr6:93824364	0.19124708	1	EPHA7	ENSG00000135333.13	Skin - Sun Exposed (Lower leg)	G=0.909	A=0.091	0.216825	9.31E-06
rs146803692	rs141173439	chr6:93700379	0.00255803	1	EPHA7	ENSG00000135333.13	Adipose - Subcutaneous	G=0.883	T=0.117	-0.326407	9.52E-06
rs146803692	rs12211507	chr6:93717031	0.00255803	1	EPHA7	ENSG00000135333.13	Adipose - Subcutaneous	C=0.883	A=0.117	-0.326407	9.52E-06
rs146803692	rs16870780	chr6:93826505	0.00166309	1	EPHA7	ENSG00000135333.13	Lung	T=0.92	A=0.08	0.385133	9.62E-06
rs146803692	rs9445060	chr6:93754781	0.01813537	1	EPHA7	ENSG00000135333.13	Breast - Mammary Tissue	A=0.485	G=0.515	0.236493	1.00E-05
rs146803692	rs1590384	chr6:93837434	0.0082189	1	EPHA7	ENSG00000135333.13	Minor Salivary Gland	C=0.701	G=0.299	-0.24131	1.00E-05
rs146803692	rs1924474	chr6:93837991	0.0082189	1	EPHA7	ENSG00000135333.13	Minor Salivary Gland	C=0.701	T=0.299	-0.24131	1.00E-05
rs146803692	rs10944652	chr6:93838348	0.0082189	1	EPHA7	ENSG00000135333.13	Minor Salivary Gland	C=0.701	G=0.299	-0.24131	1.00E-05
rs146803692	rs12204186	chr6:93838665	0.0082189	1	EPHA7	ENSG00000135333.13	Minor Salivary Gland	C=0.701	T=0.299	-0.24131	1.00E-05
rs146803692	rs633279	chr6:93840705	0.0082189	1	EPHA7	ENSG00000135333.13	Minor Salivary Gland	A=0.299	T=0.701	0.24131	1.00E-05
rs146803692	rs634060	chr6:93840849	0.0082189	1	EPHA7	ENSG00000135333.13	Minor Salivary Gland	A=0.299	G=0.701	0.24131	1.00E-05
rs146803692	rs568957	chr6:93840855	0.0082189	1	EPHA7	ENSG00000135333.13	Minor Salivary Gland	G=0.299	T=0.701	0.24131	1.00E-05
rs146803692	rs34544163	chr6:93841565	0.0082189	1	EPHA7	ENSG00000135333.13	Minor Salivary Gland	-=0.299	A=0.701	0.24131	1.00E-05
rs146803692	rs562379	chr6:93841592	0.0082189	1	EPHA7	ENSG00000135333.13	Minor Salivary Gland	G=0.299	A=0.701	0.24131	1.00E-05
rs146803692	rs560731	chr6:93841734	0.0082189	1	EPHA7	ENSG00000135333.13	Minor Salivary Gland	A=0.299	G=0.701	0.24131	1.00E-05
rs146803692	rs650642	chr6:93842232	0.0082189	1	EPHA7	ENSG00000135333.13	Minor Salivary Gland	G=0.299	T=0.701	0.24131	1.00E-05
rs146803692	rs650747	chr6:93842298	0.0082189	1	EPHA7	ENSG00000135333.13	Minor Salivary Gland	T=0.299	C=0.701	0.24131	1.00E-05
rs146803692	rs1930934	chr6:93842533	0.0082189	1	EPHA7	ENSG00000135333.13	Minor Salivary Gland	A=0.299	G=0.701	0.24131	1.00E-05
rs146803692	rs9353987	chr6:93843588	0.0082189	1	EPHA7	ENSG00000135333.13	Minor Salivary Gland	A=0.299	G=0.701	0.24131	1.00E-05
rs146803692	rs9363034	chr6:93844136	0.0082189	1	EPHA7	ENSG00000135333.13	Minor Salivary Gland	G=0.299	A=0.701	0.24131	1.00E-05
rs146803692	rs9353989	chr6:93844383	0.0082189	1	EPHA7	ENSG00000135333.13	Minor Salivary Gland	G=0.299	T=0.701	0.24131	1.00E-05
rs146803692	rs2476882	chr6:93736881	0.04846218	1	EPHA7	ENSG00000135333.13	Skin - Sun Exposed (Lower leg)	A=0.716	G=0.284	0.139213	1.05E-05
rs146803692	rs74822095	chr6:93741512	0.00096452	1	EPHA7	ENSG00000135333.13	Adipose - Subcutaneous	C=0.952	T=0.048	-0.476757	1.06E-05
rs146803692	rs10944641	chr6:93761523	0.1056901	1	EPHA7	ENSG00000135333.13	Skin - Sun Exposed (Lower leg)	T=0.846	C=0.154	0.160221	1.09E-05
rs146803692	rs76282783	chr6:93576050	0.0008387	1	EPHA7	ENSG00000135333.13	Lung	C=0.958	A=0.042	0.702179	1.18E-05
rs146803692	rs147704337	chr6:93743425	0.05264186	0.93069716	EPHA7	ENSG00000135333.13	Skin - Sun Exposed (Lower leg)	-=0.759	T=0.241	0.137649	1.18E-05
rs146803692	rs9452183	chr6:93727698	0.01340701	1	EPHA7	ENSG00000135333.13	Adipose - Subcutaneous	T=0.589	A=0.411	-0.192679	1.20E-05
rs146803692	rs12216260	chr6:93760118	0.1065014	1	EPHA7	ENSG00000135333.13	Skin - Sun Exposed (Lower leg)	G=0.847	A=0.153	0.162414	1.26E-05
rs146803692	rs72926533	chr6:93740212	0.00017377	1	EPHA7	ENSG00000135333.13	Minor Salivary Gland	C=0.991	T=0.009	-1.15096	1.32E-05
rs146803692	rs188317446	chr6:93743394	0.00017377	1	EPHA7	ENSG00000135333.13	Minor Salivary Gland	G=0.991	C=0.009	-1.15096	1.32E-05
rs146803692	rs72926535	chr6:93747452	0.00017377	1	EPHA7	ENSG00000135333.13	Minor Salivary Gland	C=0.991	T=0.009	-1.15096	1.32E-05
rs146803692	rs145106301	chr6:93728868	0.04775928	1	EPHA7	ENSG00000135333.13	Skin - Sun Exposed (Lower leg)	AG=0.713	-=0.287	0.136543	1.33E-05
rs146803692	rs9445066	chr6:93763778	0.1048892	1	EPHA7	ENSG00000135333.13	Skin - Sun Exposed (Lower leg)	G=0.845	A=0.155	0.158197	1.35E-05
rs146803692	rs6922766	chr6:93764348	0.1048892	1	EPHA7	ENSG00000135333.13	Skin - Sun Exposed (Lower leg)	C=0.845	T=0.155	0.158197	1.35E-05
rs146803692	rs4645386	chr6:93802238	0.1845998	1	EPHA7	ENSG00000135333.13	Testis	G=0.906	C=0.094	-0.434973	1.36E-05
rs146803692	rs117415227	chr6:93735745	0.00096452	1	EPHA7	ENSG00000135333.13	Adipose - Subcutaneous	T=0.952	G=0.048	-0.47253	1.37E-05
rs146803692	rs3799812	chr6:93980794	0.01036099	1	EPHA7	ENSG00000135333.13	Adipose - Subcutaneous	A=0.35	T=0.65	0.206174	1.40E-05
rs146803692	rs199888991	chr6:93784078	9.62E-05	1	EPHA7	ENSG00000135333.13	Adipose - Subcutaneous	-=0.995	T=0.005	-0.796053	1.44E-05
rs146803692	rs10485371	chr6:93758592	0.1056901	1	EPHA7	ENSG00000135333.13	Skin - Sun Exposed (Lower leg)	A=0.846	C=0.154	0.158817	1.45E-05

rs146803692	rs6927733	chr6:93764882	0.1048892	1	EPHA7	ENSG00000135333.13	Skin - Sun Exposed (Lower leg)	A=0.845	G=0.155	0.15767	1.54E-05
rs146803692	rs111429740	chr6:93838453	0.0016857	1	EPHA7	ENSG00000135333.13	Lung	G=0.919	A=0.081	0.383525	1.55E-05
rs146803692	rs7770838	chr6:93757813	0.1065014	1	EPHA7	ENSG00000135333.13	Skin - Sun Exposed (Lower leg)	T=0.847	C=0.153	0.157751	1.56E-05
rs146803692	rs9452213	chr6:93750860	0.15841722	1	EPHA7	ENSG00000135333.13	Skin - Sun Exposed (Lower leg)	A=0.892	G=0.108	0.184296	1.66E-05
rs146803692	rs9445058	chr6:93750909	0.15841722	1	EPHA7	ENSG00000135333.13	Skin - Sun Exposed (Lower leg)	G=0.892	A=0.108	0.184296	1.66E-05
rs146803692	rs9294554	chr6:93751124	0.15841722	1	EPHA7	ENSG00000135333.13	Skin - Sun Exposed (Lower leg)	G=0.892	A=0.108	0.184296	1.66E-05
rs146803692	rs36063881	chr6:93805286	0.18247636	1	EPHA7	ENSG00000135333.13	Testis	A=0.905	C=0.095	-0.433339	1.66E-05
rs146803692	rs9294558	chr6:93812815	0.18247636	1	EPHA7	ENSG00000135333.13	Testis	G=0.905	A=0.095	-0.433339	1.66E-05
rs146803692	rs9452242	chr6:93812912	0.18247636	1	EPHA7	ENSG00000135333.13	Testis	C=0.905	T=0.095	-0.433339	1.66E-05
rs146803692	rs34459031	chr6:93813039	0.18247636	1	EPHA7	ENSG00000135333.13	Testis	G=0.905	A=0.095	-0.433339	1.66E-05
rs146803692	rs7760335	chr6:93752014	0.15841722	1	EPHA7	ENSG00000135333.13	Skin - Sun Exposed (Lower leg)	A=0.892	G=0.108	0.183015	1.78E-05
rs146803692	rs7765777	chr6:93752927	0.15680206	1	EPHA7	ENSG00000135333.13	Skin - Sun Exposed (Lower leg)	C=0.891	T=0.109	0.183015	1.78E-05
rs146803692	rs16870789	chr6:93827963	0.00166309	1	EPHA7	ENSG00000135333.13	Lung	C=0.92	T=0.08	0.374368	1.81E-05
rs146803692	rs145810085	chr6:93833819	0.00015431	1	EPHA7	ENSG00000135333.13	Adipose - Subcutaneous	G=0.992	A=0.008	-0.735109	1.88E-05
rs146803692	rs7774823	chr6:93830545	0.0081412	1	EPHA7	ENSG00000135333.13	Minor Salivary Gland	G=0.703	A=0.297	-0.232136	1.89E-05
rs146803692	rs1951907	chr6:93831192	0.0081412	1	EPHA7	ENSG00000135333.13	Minor Salivary Gland	T=0.703	C=0.297	-0.232136	1.89E-05
rs146803692	rs35306488	chr6:93832227	0.00802546	1	EPHA7	ENSG00000135333.13	Minor Salivary Gland	-=0.706	T=0.294	-0.232136	1.89E-05
rs146803692	rs10944648	chr6:93832280	0.0081412	1	EPHA7	ENSG00000135333.13	Minor Salivary Gland	A=0.703	G=0.297	-0.232136	1.89E-05
rs146803692	rs7751375	chr6:93832923	0.00817999	1	EPHA7	ENSG00000135333.13	Minor Salivary Gland	C=0.702	A=0.298	-0.232136	1.89E-05
rs146803692	rs7757292	chr6:93834165	0.0081412	1	EPHA7	ENSG00000135333.13	Minor Salivary Gland	C=0.703	A=0.297	-0.232136	1.89E-05
rs146803692	rs16870850	chr6:93841079	0.00177662	1	EPHA7	ENSG00000135333.13	Lung	T=0.916	C=0.084	0.361979	2.02E-05
rs146803692	rs12528846	chr6:93828842	0.00166309	1	EPHA7	ENSG00000135333.13	Lung	A=0.92	G=0.08	0.377147	2.03E-05
rs146803692	rs12527296	chr6:93829143	0.00166309	1	EPHA7	ENSG00000135333.13	Lung	T=0.92	C=0.08	0.377147	2.03E-05
rs146803692	rs72928575	chr6:93829399	0.0016857	1	EPHA7	ENSG00000135333.13	Lung	G=0.919	T=0.081	0.377147	2.03E-05
rs146803692	rs72928579	chr6:93829658	0.00166309	1	EPHA7	ENSG00000135333.13	Lung	G=0.92	A=0.08	0.377147	2.03E-05
rs146803692	rs77007574	chr6:93829960	0.00166309	1	EPHA7	ENSG00000135333.13	Lung	T=0.92	C=0.08	0.377147	2.03E-05
rs146803692	rs72928582	chr6:93830015	0.00166309	1	EPHA7	ENSG00000135333.13	Lung	G=0.92	A=0.08	0.377147	2.03E-05
rs146803692	rs16870801	chr6:93830951	0.00166309	1	EPHA7	ENSG00000135333.13	Lung	T=0.92	C=0.08	0.377147	2.03E-05
rs146803692	rs16870805	chr6:93834075	0.00166309	1	EPHA7	ENSG00000135333.13	Lung	G=0.92	T=0.08	0.377147	2.03E-05
rs146803692	rs150304721	chr6:93835801	0.00170836	1	EPHA7	ENSG00000135333.13	Lung	A=0.918	-=0.082	0.361316	2.04E-05
rs146803692	rs117128147	chr6:94125224	0.00017377	1	EPHA7	ENSG00000135333.13	Brain - Frontal Cortex (BA9)	T=0.991	C=0.009	1.05416	2.14E-05
rs146803692	rs16870855	chr6:93841440	0.0016857	1	EPHA7	ENSG00000135333.13	Lung	C=0.919	T=0.081	0.4288	2.14E-05
rs146803692	rs16880183	chr6:93889701	0.0037768	1	EPHA7	ENSG00000135333.13	Testis	A=0.836	G=0.164	0.353058	2.24E-05
rs146803692	rs7772178	chr6:93849608	0.00255803	1	EPHA7	ENSG00000135333.13	Testis	C=0.883	T=0.117	0.425527	2.47E-05
rs146803692	rs6923008	chr6:93887045	0.0037768	1	EPHA7	ENSG00000135333.13	Testis	G=0.836	T=0.164	0.351036	2.54E-05
rs146803692	rs60592958	chr6:93887933	0.0037768	1	EPHA7	ENSG00000135333.13	Testis	C=0.836	T=0.164	0.351036	2.54E-05
rs146803692	rs147445521	chr6:93825169	0.00139554	1	EPHA7	ENSG00000135333.13	Lung	TGT=0.932	-=0.068	0.417524	2.57E-05
rs146803692	rs118177782	chr6:93767900	0.00051072	1	EPHA7	ENSG00000135333.13	Brain - Anterior cingulate cortex (BA24)	C=0.974	T=0.026	0.590137	2.71E-05
rs146803692	rs73758259	chr6:93828724	0.00173106	1	EPHA7	ENSG00000135333.13	Lung	G=0.917	T=0.083	0.354112	2.72E-05
rs146803692	rs3799812	chr6:93980794	0.01036099	1	EPHA7	ENSG00000135333.13	Heart - Left Ventricle	A=0.35	T=0.65	0.157039	2.74E-05
rs146803692	rs150162846	chr6:93814656	0.10331782	1	EPHA7	ENSG00000135333.13	Skin - Sun Exposed (Lower leg)	T=0.843	-=0.157	0.157667	2.75E-05
rs146803692	rs112417593	chr6:93765399	0.10254695	1	EPHA7	ENSG00000135333.13	Skin - Sun Exposed (Lower leg)	-=0.842	T=0.158	0.151487	2.75E-05
rs146803692	rs16870796	chr6:93828781	0.00166309	1	EPHA7	ENSG00000135333.13	Lung	T=0.92	G=0.08	0.369069	2.76E-05
rs146803692	rs72928577	chr6:93829400	0.0016857	1	EPHA7	ENSG00000135333.13	Lung	C=0.919	T=0.081	0.369069	2.76E-05
rs146803692	rs117717826	chr6:94036945	0.00049058	1	EPHA7	ENSG00000135333.13	Heart - Left Ventricle	T=0.975	A=0.025	-0.389622	2.96E-05
rs146803692	rs9445074	chr6:93819929	0.00442426	1	EPHA7	ENSG00000135333.13	Adipose - Subcutaneous	T=0.813	C=0.187	0.233649	2.98E-05
rs146803692	rs56378615	chr6:93900091	0.00332056	1	EPHA7	ENSG00000135333.13	Testis	C=0.853	T=0.147	0.358985	3.02E-05
rs146803692	rs67438908	chr6:93814666	0.10331782	1	EPHA7	ENSG00000135333.13	Skin - Sun Exposed (Lower leg)	T=0.843	-=0.157	0.157172	3.13E-05
rs146803692	rs10944646	chr6:93815415	0.10331782	1	EPHA7	ENSG00000135333.13	Skin - Sun Exposed (Lower leg)	T=0.843	A=0.157	0.157172	3.13E-05
rs146803692	rs71558449	chr6:93828589	0.00065289	1	EPHA7	ENSG00000135333.13	Esophagus - Muscularis	G=0.967	A=0.033	0.272458	3.14E-05
rs146803692	rs4645386	chr6:93802238	0.1845998	1	EPHA7	ENSG00000135333.13	Skin - Sun Exposed (Lower leg)	G=0.906	C=0.094	0.184149	3.19E-05

rs146803692	rs559992	chr6:93759522	0.04942264	1	EPHA7	ENSG00000135333.13	Skin - Sun Exposed (Lower leg)	T=0.72	C=0.28	0.132011	3.25E-05
rs146803692	rs6922405	chr6:93842847	0.0016857	1	EPHA7	ENSG00000135333.13	Lung	T=0.919	C=0.081	0.370721	3.25E-05
rs146803692	rs72930416	chr6:93844150	0.0016857	1	EPHA7	ENSG00000135333.13	Lung	T=0.919	C=0.081	0.370721	3.25E-05
rs146803692	rs13220164	chr6:93816028	0.00448229	1	EPHA7	ENSG00000135333.13	Adipose - Subcutaneous	T=0.811	C=0.189	0.235113	3.37E-05
rs146803692	rs16870846	chr6:93840909	0.0016857	1	EPHA7	ENSG00000135333.13	Lung	C=0.919	G=0.081	0.371767	3.40E-05
rs146803692	rs1324115	chr6:93790248	0.05066222	1	EPHA7	ENSG00000135333.13	Adipose - Subcutaneous	G=0.275	T=0.725	-0.19168	3.51E-05
rs146803692	rs1319460	chr6:93846637	0.00603141	1	EPHA7	ENSG00000135333.13	Minor Salivary Gland	C=0.239	T=0.761	0.230876	3.54E-05
rs146803692	rs538751	chr6:93848400	0.00603141	1	EPHA7	ENSG00000135333.13	Minor Salivary Gland	A=0.239	C=0.761	0.230876	3.54E-05
rs146803692	rs11422146	chr6:93849652	0.00599845	1	EPHA7	ENSG00000135333.13	Minor Salivary Gland	-=0.238	T=0.762	0.230876	3.54E-05
rs146803692	rs473900	chr6:93931037	0.0056735	1	EPHA7	ENSG00000135333.13	Adipose - Subcutaneous	T=0.228	C=0.772	0.223628	3.56E-05
rs146803692	rs369196	chr6:93932634	0.0056735	1	EPHA7	ENSG00000135333.13	Adipose - Subcutaneous	C=0.228	T=0.772	0.223628	3.56E-05
rs146803692	rs1319460	chr6:93846637	0.00603141	1	EPHA7	ENSG00000135333.13	Testis	C=0.239	T=0.761	-0.330556	3.66E-05
rs146803692	rs538751	chr6:93848400	0.00603141	1	EPHA7	ENSG00000135333.13	Testis	A=0.239	C=0.761	-0.330556	3.66E-05
rs146803692	rs11422146	chr6:93849652	0.00599845	1	EPHA7	ENSG00000135333.13	Testis	-=0.238	T=0.762	-0.330556	3.66E-05
rs146803692	rs118077652	chr6:93972449	0.00051072	1	EPHA7	ENSG00000135333.13	Esophagus - Gastroesophageal Junction	A=0.974	G=0.026	-0.349896	3.99E-05
rs146803692	rs117415227	chr6:93735745	0.00096452	1	EPHA7	ENSG00000135333.13	Lung	T=0.952	G=0.048	-0.50856	4.07E-05
rs146803692	rs1324115	chr6:93790248	0.05066222	1	EPHA7	ENSG00000135333.13	Skin - Sun Exposed (Lower leg)	G=0.275	T=0.725	-0.125437	4.07E-05
rs146803692	rs553697	chr6:93730393	0.00372217	1	EPHA7	ENSG00000135333.13	Breast - Mammary Tissue	T=0.162	C=0.838	0.303124	4.12E-05
rs146803692	rs6935219	chr6:93889277	0.0037768	1	EPHA7	ENSG00000135333.13	Testis	A=0.836	G=0.164	0.33974	4.29E-05
rs146803692	rs140182812	chr6:93804212	0.00015431	1	EPHA7	ENSG00000135333.13	Adipose - Subcutaneous	G=0.992	A=0.008	-0.729088	4.39E-05
rs146803692	rs117238762	chr6:93927805	0.00051072	1	EPHA7	ENSG00000135333.13	Esophagus - Gastroesophageal Junction	G=0.974	T=0.026	-0.390831	4.53E-05
rs146803692	rs117022700	chr6:93948952	0.00049058	1	EPHA7	ENSG00000135333.13	Esophagus - Gastroesophageal Junction	A=0.975	C=0.025	-0.390831	4.53E-05
rs146803692	rs2057541	chr6:93781036	0.05066222	1	EPHA7	ENSG00000135333.13	Adipose - Subcutaneous	A=0.275	G=0.725	-0.188926	4.60E-05
rs146803692	rs7769684	chr6:93793869	0.05066222	1	EPHA7	ENSG00000135333.13	Adipose - Subcutaneous	C=0.275	T=0.725	-0.188926	4.60E-05
rs146803692	rs10686381	chr6:93887552	0.0037768	1	EPHA7	ENSG00000135333.13	Testis	-=0.836	AAGT=0.164	0.337644	4.84E-05
rs146803692	rs142985966	chr6:93961341	0.00749803	1	EPHA7	ENSG00000135333.13	Heart - Atrial Appendage	-=0.28	AAAT=0.72	0.0998058	4.85E-05
rs146803692	rs41273625	chr6:93952851	0.00128607	1	EPHA7	ENSG00000135333.13	Esophagus - Muscularis	C=0.937	G=0.063	0.224035	5.17E-05
rs146803692	rs9452212	chr6:93750416	0.1210813	1	EPHA7	ENSG00000135333.13	Skin - Sun Exposed (Lower leg)	A=0.863	G=0.137	0.160554	5.27E-05
rs146803692	rs12215791	chr6:94213910	0.00122095	1	EPHA7	ENSG00000135333.13	Colon - Sigmoid	G=0.94	A=0.06	-0.272256	5.28E-05
rs146803692	rs72624587	chr6:94221190	0.00122095	1	EPHA7	ENSG00000135333.13	Colon - Sigmoid	T=0.94	G=0.06	-0.272256	5.28E-05
rs146803692	rs7755786	chr6:94222857	0.00119933	1	EPHA7	ENSG00000135333.13	Colon - Sigmoid	A=0.941	T=0.059	-0.272256	5.28E-05
rs146803692	rs12201863	chr6:94226358	0.00119933	1	EPHA7	ENSG00000135333.13	Colon - Sigmoid	G=0.941	C=0.059	-0.272256	5.28E-05
rs146803692	rs7759544	chr6:94226888	0.00119933	1	EPHA7	ENSG00000135333.13	Colon - Sigmoid	T=0.941	A=0.059	-0.272256	5.28E-05
rs146803692	rs10806477	chr6:94228336	0.00119933	1	EPHA7	ENSG00000135333.13	Colon - Sigmoid	G=0.941	A=0.059	-0.272256	5.28E-05
rs146803692	rs12199277	chr6:94230292	0.00119933	1	EPHA7	ENSG00000135333.13	Colon - Sigmoid	C=0.941	A=0.059	-0.272256	5.28E-05
rs146803692	rs78355320	chr6:93821638	0.00096452	1	EPHA7	ENSG00000135333.13	Adipose - Subcutaneous	T=0.952	A=0.048	-0.431835	5.33E-05
rs146803692	rs12530331	chr6:93830460	0.0016857	1	EPHA7	ENSG00000135333.13	Lung	G=0.919	C=0.081	0.35653	5.33E-05
rs146803692	rs508511	chr6:93759983	0.0081412	1	EPHA7	ENSG00000135333.13	Esophagus - Mucosa	C=0.297	T=0.703	0.218061	5.36E-05
rs146803692	rs72920784	chr6:93673551	0.00039047	1	EPHA7	ENSG00000135333.13	Adipose - Subcutaneous	A=0.98	G=0.02	-0.579253	5.40E-05
rs146803692	rs72920785	chr6:93675496	0.00039047	1	EPHA7	ENSG00000135333.13	Adipose - Subcutaneous	G=0.98	C=0.02	-0.579253	5.40E-05
rs146803692	rs143162849	chr6:93732124	0.00041041	1	EPHA7	ENSG00000135333.13	Skin - Sun Exposed (Lower leg)	G=0.979	A=0.021	-0.424949	5.53E-05
rs146803692	rs9445060	chr6:93754781	0.01813537	1	EPHA7	ENSG00000135333.13	Nerve - Tibial	A=0.485	G=0.515	0.1737	5.83E-05
rs146803692	rs380782	chr6:93996635	0.0072057	1	EPHA7	ENSG00000135333.13	Heart - Atrial Appendage	A=0.272	G=0.728	0.096935	5.93E-05
rs146803692	rs74468395	chr6:94017659	0.00065289	1	EPHA7	ENSG00000135333.13	Heart - Left Ventricle	C=0.967	T=0.033	-0.353	6.19E-05
rs146803692	rs117256127	chr6:93852714	0.00047047	1	EPHA7	ENSG00000135333.13	Adipose - Subcutaneous	T=0.976	C=0.024	-0.674139	6.42E-05
rs146803692	rs117425371	chr6:93852717	0.00047047	1	EPHA7	ENSG00000135333.13	Adipose - Subcutaneous	A=0.976	G=0.024	-0.674139	6.42E-05
rs146803692	rs145519743	chr6:93884032	0.00049058	1	EPHA7	ENSG00000135333.13	Adipose - Subcutaneous	G=0.975	A=0.025	-0.674139	6.42E-05
rs146803692	rs2780662	chr6:93920660	0.00768404	1	EPHA7	ENSG00000135333.13	Testis	T=0.285	C=0.715	-0.29987	6.44E-05
rs146803692	rs79987536	chr6:94097796	0.00059171	1	EPHA7	ENSG00000135333.13	Esophagus - Gastroesophageal Junction	A=0.97	C=0.03	-0.290018	6.44E-05
rs146803692	rs117075973	chr6:94099681	0.00053091	1	EPHA7	ENSG00000135333.13	Esophagus - Gastroesophageal Junction	C=0.973	G=0.027	-0.290018	6.44E-05
rs146803692	rs147330775	chr6:93915683	0.0001155	1	EPHA7	ENSG00000135333.13	Skin - Sun Exposed (Lower leg)	C=0.994	T=0.006	-0.47159	6.56E-05

rs146803692	rs9452184	chr6:93728200	0.0179201	1	EPHA7	ENSG00000135333.13	Breast - Mammary Tissue	T=0.482	G=0.518	0.223443	6.62E-05
rs146803692	rs1570632	chr6:93811908	0.05091553	1	EPHA7	ENSG00000135333.13	Adipose - Subcutaneous	A=0.274	T=0.726	-0.183765	6.82E-05
rs146803692	rs2057541	chr6:93781036	0.05066222	1	EPHA7	ENSG00000135333.13	Skin - Sun Exposed (Lower leg)	A=0.275	G=0.725	-0.121874	6.87E-05
rs146803692	rs7769684	chr6:93793869	0.05066222	1	EPHA7	ENSG00000135333.13	Skin - Sun Exposed (Lower leg)	C=0.275	T=0.725	-0.121874	6.87E-05
rs146803692	rs9452232	chr6:93773302	0.09955806	1	EPHA7	ENSG00000135333.13	Skin - Sun Exposed (Lower leg)	C=0.838	T=0.162	0.142532	6.91E-05
rs146803692	rs9445070	chr6:93773341	0.09955806	1	EPHA7	ENSG00000135333.13	Skin - Sun Exposed (Lower leg)	T=0.838	A=0.162	0.142532	6.91E-05
rs146803692	rs10944642	chr6:93776755	0.09955806	1	EPHA7	ENSG00000135333.13	Skin - Sun Exposed (Lower leg)	C=0.838	T=0.162	0.142532	6.91E-05
rs146803692	rs6900522	chr6:93783200	0.09955806	1	EPHA7	ENSG00000135333.13	Skin - Sun Exposed (Lower leg)	A=0.838	G=0.162	0.142532	6.91E-05
rs146803692	rs12205294	chr6:93784097	0.09955806	1	EPHA7	ENSG00000135333.13	Skin - Sun Exposed (Lower leg)	C=0.838	G=0.162	0.142532	6.91E-05
rs146803692	rs117109139	chr6:93861398	0.00075569	1	EPHA7	ENSG00000135333.13	Esophagus - Muscularis	C=0.962	G=0.038	0.257424	7.15E-05
rs146803692	rs36063881	chr6:93805286	0.18247636	1	EPHA7	ENSG00000135333.13	Skin - Sun Exposed (Lower leg)	A=0.905	C=0.095	0.17563	7.35E-05
rs146803692	rs9294558	chr6:93812815	0.18247636	1	EPHA7	ENSG00000135333.13	Skin - Sun Exposed (Lower leg)	G=0.905	A=0.095	0.17563	7.35E-05
rs146803692	rs9452242	chr6:93812912	0.18247636	1	EPHA7	ENSG00000135333.13	Skin - Sun Exposed (Lower leg)	C=0.905	T=0.095	0.17563	7.35E-05
rs146803692	rs34459031	chr6:93813039	0.18247636	1	EPHA7	ENSG00000135333.13	Skin - Sun Exposed (Lower leg)	G=0.905	A=0.095	0.17563	7.35E-05
rs146803692	rs1386274	chr6:93961571	0.00746113	1	EPHA7	ENSG00000135333.13	Heart - Atrial Appendage	C=0.279	T=0.721	0.0953278	7.38E-05
rs146803692	rs2631560	chr6:93962517	0.00746113	1	EPHA7	ENSG00000135333.13	Heart - Atrial Appendage	C=0.279	T=0.721	0.0953278	7.38E-05
rs146803692	rs2631561	chr6:93962704	0.00746113	1	EPHA7	ENSG00000135333.13	Heart - Atrial Appendage	A=0.279	T=0.721	0.0953278	7.38E-05
rs146803692	rs419641	chr6:93963135	0.00746113	1	EPHA7	ENSG00000135333.13	Heart - Atrial Appendage	A=0.279	C=0.721	0.0953278	7.38E-05
rs146803692	rs3799817	chr6:93963180	0.00746113	1	EPHA7	ENSG00000135333.13	Heart - Atrial Appendage	A=0.279	T=0.721	0.0953278	7.38E-05
rs146803692	rs345737	chr6:93964187	0.00746113	1	EPHA7	ENSG00000135333.13	Heart - Atrial Appendage	C=0.279	T=0.721	0.0953278	7.38E-05
rs146803692	rs117760278	chr6:93741763	0.00098565	1	EPHA7	ENSG00000135333.13	Skin - Sun Exposed (Lower leg)	G=0.951	A=0.049	0.280199	7.44E-05
rs146803692	rs143798120	chr6:93743175	0.00088047	1	EPHA7	ENSG00000135333.13	Skin - Sun Exposed (Lower leg)	A=0.956	G=0.044	0.280199	7.44E-05
rs146803692	rs345730	chr6:93967851	0.00735106	1	EPHA7	ENSG00000135333.13	Heart - Atrial Appendage	C=0.276	T=0.724	0.0941831	7.46E-05
rs146803692	rs117630977	chr6:94026117	0.00059171	1	EPHA7	ENSG00000135333.13	Esophagus - Gastroesophageal Junction	C=0.97	T=0.03	-0.323671	7.75E-05
rs146803692	rs141173439	chr6:93700379	0.00255803	1	EPHA7	ENSG00000135333.13	Nerve - Tibial	G=0.883	T=0.117	-0.270739	7.95E-05
rs146803692	rs12211507	chr6:93717031	0.00255803	1	EPHA7	ENSG00000135333.13	Nerve - Tibial	C=0.883	A=0.117	-0.270739	7.95E-05
rs146803692	rs62414183	chr6:93914220	0.00397008	1	EPHA7	ENSG00000135333.13	Testis	A=0.829	G=0.171	0.324951	8.22E-05
rs146803692	rs471850	chr6:93766281	0.00817999	1	EPHA7	ENSG00000135333.13	Esophagus - Mucosa	C=0.298	A=0.702	0.215381	8.30E-05
rs146803692	rs1570632	chr6:93811908	0.05091553	1	EPHA7	ENSG00000135333.13	Skin - Sun Exposed (Lower leg)	A=0.274	T=0.726	-0.120054	8.49E-05
rs146803692	rs1486755	chr6:93961392	0.00746113	1	EPHA7	ENSG00000135333.13	Heart - Atrial Appendage	G=0.279	T=0.721	0.0935778	8.52E-05
rs146803692	rs117775893	chr6:94024023	0.0005714	1	EPHA7	ENSG00000135333.13	Esophagus - Gastroesophageal Junction	A=0.971	T=0.029	-0.310661	8.61E-05
rs146803692	rs412388	chr6:93916878	0.00768404	1	EPHA7	ENSG00000135333.13	Testis	G=0.285	A=0.715	-0.296773	8.64E-05
rs146803692	rs431650	chr6:93953878	0.00657075	1	EPHA7	ENSG00000135333.13	Heart - Left Ventricle	T=0.254	C=0.746	0.159801	9.12E-05
rs146803692	rs393351	chr6:93958447	0.00742434	1	EPHA7	ENSG00000135333.13	Heart - Atrial Appendage	A=0.278	T=0.722	0.0934689	9.24E-05
rs146803692	rs378261	chr6:93958449	0.00742434	1	EPHA7	ENSG00000135333.13	Heart - Atrial Appendage	T=0.278	C=0.722	0.0934689	9.24E-05
rs146803692	rs413576	chr6:93958639	0.00742434	1	EPHA7	ENSG00000135333.13	Heart - Atrial Appendage	G=0.278	A=0.722	0.0934689	9.24E-05
rs146803692	rs422834	chr6:93958659	0.00742434	1	EPHA7	ENSG00000135333.13	Heart - Atrial Appendage	T=0.278	C=0.722	0.0934689	9.24E-05
rs146803692	rs477307	chr6:93958852	0.00738765	1	EPHA7	ENSG00000135333.13	Heart - Atrial Appendage	T=0.277	A=0.723	0.0934689	9.24E-05
rs146803692	rs656435	chr6:93958900	0.00735106	1	EPHA7	ENSG00000135333.13	Heart - Atrial Appendage	C=0.276	T=0.724	0.0934689	9.24E-05
rs146803692	rs3799827	chr6:93958946	0.00735106	1	EPHA7	ENSG00000135333.13	Heart - Atrial Appendage	T=0.276	C=0.724	0.0934689	9.24E-05
rs146803692	rs2450422	chr6:93959022	0.00735106	1	EPHA7	ENSG00000135333.13	Heart - Atrial Appendage	G=0.276	A=0.724	0.0934689	9.24E-05
rs146803692	rs2631557	chr6:93959088	0.00738765	1	EPHA7	ENSG00000135333.13	Heart - Atrial Appendage	T=0.277	C=0.723	0.0934689	9.24E-05
rs146803692	rs2780660	chr6:93959146	0.00742434	1	EPHA7	ENSG00000135333.13	Heart - Atrial Appendage	A=0.278	G=0.722	0.0934689	9.24E-05
rs146803692	rs2631559	chr6:93959778	0.00742434	1	EPHA7	ENSG00000135333.13	Heart - Atrial Appendage	T=0.278	G=0.722	0.0934689	9.24E-05
rs146803692	rs423713	chr6:93960424	0.00746113	1	EPHA7	ENSG00000135333.13	Heart - Atrial Appendage	C=0.279	T=0.721	0.0934689	9.24E-05
rs146803692	rs392257	chr6:93960970	0.00746113	1	EPHA7	ENSG00000135333.13	Heart - Atrial Appendage	G=0.279	A=0.721	0.0934689	9.24E-05
rs146803692	rs440799	chr6:93961019	0.00746113	1	EPHA7	ENSG00000135333.13	Heart - Atrial Appendage	G=0.279	A=0.721	0.0934689	9.24E-05
rs146803692	rs66492350	chr6:93961239	0.00746113	1	EPHA7	ENSG00000135333.13	Heart - Atrial Appendage	-=0.279	T=0.721	0.0934689	9.24E-05
rs146803692	rs1386275	chr6:93961622	0.00746113	1	EPHA7	ENSG00000135333.13	Heart - Atrial Appendage	C=0.279	G=0.721	0.0934689	9.24E-05
rs146803692	rs3839561	chr6:93961845	0.00746113	1	EPHA7	ENSG00000135333.13	Heart - Atrial Appendage	-=0.279	CTT=0.721	0.0934689	9.24E-05
rs146803692	rs404201	chr6:93961892	0.00746113	1	EPHA7	ENSG00000135333.13	Heart - Atrial Appendage	T=0.279	C=0.721	0.0934689	9.24E-05

rs146803692	rs2274940	chr6:93963799	0.00746113	1	EPHA7	ENSG00000135333.13	Heart - Atrial Appendage	A=0.279	G=0.721	0.0934689	9.24E-05
rs146803692	rs7764643	chr6:93978372	0.0072057	1	EPHA7	ENSG00000135333.13	Heart - Atrial Appendage	G=0.272	T=0.728	0.0936658	9.43E-05
rs146803692	rs345717	chr6:93985029	0.00643377	1	EPHA7	ENSG00000135333.13	Heart - Left Ventricle	A=0.25	G=0.75	0.160928	9.48E-05
rs146803692	rs11332211	chr6:93985322	0.00735106	1	EPHA7	ENSG00000135333.13	Heart - Atrial Appendage	A=0.276	-=0.724	0.0945191	9.50E-05
rs146803692	rs345718	chr6:93987100	0.00735106	1	EPHA7	ENSG00000135333.13	Heart - Atrial Appendage	C=0.276	T=0.724	0.0945191	9.50E-05
rs146803692	rs12211644	chr6:93768334	0.09883362	1	EPHA7	ENSG00000135333.13	Skin - Sun Exposed (Lower leg)	T=0.837	C=0.163	0.14026	9.60E-05
rs146803692	rs1924475	chr6:93768807	0.09883362	1	EPHA7	ENSG00000135333.13	Skin - Sun Exposed (Lower leg)	G=0.837	A=0.163	0.14026	9.60E-05
rs146803692	rs9445068	chr6:93768956	0.09883362	1	EPHA7	ENSG00000135333.13	Skin - Sun Exposed (Lower leg)	A=0.837	G=0.163	0.14026	9.60E-05
rs146803692	rs11962127	chr6:93769208	0.09883362	1	EPHA7	ENSG00000135333.13	Skin - Sun Exposed (Lower leg)	A=0.837	G=0.163	0.14026	9.60E-05
rs146803692	rs6924214	chr6:93772909	0.09955806	1	EPHA7	ENSG00000135333.13	Skin - Sun Exposed (Lower leg)	T=0.838	C=0.162	0.14026	9.60E-05
rs146803692	rs9452235	chr6:93778531	0.09955806	1	EPHA7	ENSG00000135333.13	Skin - Sun Exposed (Lower leg)	T=0.838	C=0.162	0.14026	9.60E-05
rs146803692	rs9452237	chr6:93779733	0.09955806	1	EPHA7	ENSG00000135333.13	Skin - Sun Exposed (Lower leg)	C=0.838	A=0.162	0.14026	9.60E-05
rs146803692	rs113218522	chr6:93784667	0.09955806	1	EPHA7	ENSG00000135333.13	Skin - Sun Exposed (Lower leg)	-=0.838	CT=0.162	0.14026	9.60E-05
rs146803692	rs10944644	chr6:93785405	0.09955806	1	EPHA7	ENSG00000135333.13	Skin - Sun Exposed (Lower leg)	G=0.838	A=0.162	0.14026	9.60E-05
rs146803692	rs6918068	chr6:93785871	0.09955806	1	EPHA7	ENSG00000135333.13	Skin - Sun Exposed (Lower leg)	A=0.838	G=0.162	0.14026	9.60E-05
rs146803692	rs16870683	chr6:93788145	0.09955806	1	EPHA7	ENSG00000135333.13	Skin - Sun Exposed (Lower leg)	T=0.838	C=0.162	0.14026	9.60E-05
rs146803692	rs9452239	chr6:93789496	0.09955806	1	EPHA7	ENSG00000135333.13	Skin - Sun Exposed (Lower leg)	C=0.838	T=0.162	0.14026	9.60E-05
rs146803692	rs7753080	chr6:93797974	0.09955806	1	EPHA7	ENSG00000135333.13	Skin - Sun Exposed (Lower leg)	A=0.838	G=0.162	0.14026	9.60E-05
rs146803692	rs553697	chr6:93730393	0.00372217	1	EPHA7	ENSG00000135333.13	Skin - Not Sun Exposed (Suprapubic)	T=0.162	C=0.838	0.15591	9.94E-05
rs146803692	rs12211644	chr6:93768334	0.09883362	1	EPHA7	ENSG00000135333.13	Adipose - Subcutaneous	T=0.837	C=0.163	0.211846	0.00010172
rs146803692	rs1924475	chr6:93768807	0.09883362	1	EPHA7	ENSG00000135333.13	Adipose - Subcutaneous	G=0.837	A=0.163	0.211846	0.00010172
rs146803692	rs9445068	chr6:93768956	0.09883362	1	EPHA7	ENSG00000135333.13	Adipose - Subcutaneous	A=0.837	G=0.163	0.211846	0.00010172
rs146803692	rs11962127	chr6:93769208	0.09883362	1	EPHA7	ENSG00000135333.13	Adipose - Subcutaneous	A=0.837	G=0.163	0.211846	0.00010172
rs146803692	rs6924214	chr6:93772909	0.09955806	1	EPHA7	ENSG00000135333.13	Adipose - Subcutaneous	T=0.838	C=0.162	0.211846	0.00010172
rs146803692	rs9452232	chr6:93773302	0.09955806	1	EPHA7	ENSG00000135333.13	Adipose - Subcutaneous	C=0.838	T=0.162	0.211846	0.00010172
rs146803692	rs9445070	chr6:93773341	0.09955806	1	EPHA7	ENSG00000135333.13	Adipose - Subcutaneous	T=0.838	A=0.162	0.211846	0.00010172
rs146803692	rs10944642	chr6:93776755	0.09955806	1	EPHA7	ENSG00000135333.13	Adipose - Subcutaneous	C=0.838	T=0.162	0.211846	0.00010172
rs146803692	rs9452235	chr6:93778531	0.09955806	1	EPHA7	ENSG00000135333.13	Adipose - Subcutaneous	T=0.838	C=0.162	0.211846	0.00010172
rs146803692	rs9452237	chr6:93779733	0.09955806	1	EPHA7	ENSG00000135333.13	Adipose - Subcutaneous	C=0.838	A=0.162	0.211846	0.00010172
rs146803692	rs6900522	chr6:93783200	0.09955806	1	EPHA7	ENSG00000135333.13	Adipose - Subcutaneous	A=0.838	G=0.162	0.211846	0.00010172
rs146803692	rs12205294	chr6:93784097	0.09955806	1	EPHA7	ENSG00000135333.13	Adipose - Subcutaneous	C=0.838	G=0.162	0.211846	0.00010172
rs146803692	rs113218522	chr6:93784667	0.09955806	1	EPHA7	ENSG00000135333.13	Adipose - Subcutaneous	-=0.838	CT=0.162	0.211846	0.00010172
rs146803692	rs10944644	chr6:93785405	0.09955806	1	EPHA7	ENSG00000135333.13	Adipose - Subcutaneous	G=0.838	A=0.162	0.211846	0.00010172
rs146803692	rs6918068	chr6:93785871	0.09955806	1	EPHA7	ENSG00000135333.13	Adipose - Subcutaneous	A=0.838	G=0.162	0.211846	0.00010172
rs146803692	rs16870683	chr6:93788145	0.09955806	1	EPHA7	ENSG00000135333.13	Adipose - Subcutaneous	T=0.838	C=0.162	0.211846	0.00010172
rs146803692	rs9452239	chr6:93789496	0.09955806	1	EPHA7	ENSG00000135333.13	Adipose - Subcutaneous	C=0.838	T=0.162	0.211846	0.00010172
rs146803692	rs7753080	chr6:93797974	0.09955806	1	EPHA7	ENSG00000135333.13	Adipose - Subcutaneous	A=0.838	G=0.162	0.211846	0.00010172
rs146803692	rs41273625	chr6:93952851	0.00128607	1	EPHA7	ENSG00000135333.13	Esophagus - Gastroesophageal Junction	C=0.937	G=0.063	0.249826	0.00010545
rs146803692	rs10755499	chr6:93774438	0.19124708	1	EPHA7	ENSG00000135333.13	Testis	C=0.909	T=0.091	-0.407257	0.00010938
rs146803692	rs75887019	chr6:93776052	0.19124708	1	EPHA7	ENSG00000135333.13	Testis	T=0.909	C=0.091	-0.407257	0.00010938
rs146803692	rs13213928	chr6:93778130	0.19124708	1	EPHA7	ENSG00000135333.13	Testis	C=0.909	T=0.091	-0.407257	0.00010938
rs146803692	rs10944643	chr6:93778739	0.19124708	1	EPHA7	ENSG00000135333.13	Testis	C=0.909	T=0.091	-0.407257	0.00010938
rs146803692	rs11970484	chr6:93779586	0.19124708	1	EPHA7	ENSG00000135333.13	Testis	G=0.909	A=0.091	-0.407257	0.00010938
rs146803692	rs11963855	chr6:93779831	0.19124708	1	EPHA7	ENSG00000135333.13	Testis	G=0.909	T=0.091	-0.407257	0.00010938
rs146803692	rs16870674	chr6:93780798	0.19124708	1	EPHA7	ENSG00000135333.13	Testis	C=0.909	T=0.091	-0.407257	0.00010938
rs146803692	rs13213427	chr6:93781422	0.19124708	1	EPHA7	ENSG00000135333.13	Testis	A=0.909	G=0.091	-0.407257	0.00010938
rs146803692	rs6900435	chr6:93782921	0.19124708	1	EPHA7	ENSG00000135333.13	Testis	G=0.909	A=0.091	-0.407257	0.00010938
rs146803692	rs138022068	chr6:94069111	0.00096452	1	EPHA7	ENSG00000135333.13	Heart - Left Ventricle	C=0.952	-=0.048	-0.300501	0.00011172
rs146803692	rs503319	chr6:93750010	0.00551414	1	EPHA7	ENSG00000135333.13	Lung	A=0.777	T=0.223	0.220393	0.00011194
rs146803692	rs477356	chr6:93958870	0.00735106	1	EPHA7	ENSG00000135333.13	Heart - Atrial Appendage	A=0.276	G=0.724	0.0921109	0.00011272
rs146803692	rs424565	chr6:93961071	0.00746113	1	EPHA7	ENSG00000135333.13	Heart - Atrial Appendage	G=0.279	A=0.721	0.0922427	0.00011317

rs146803692	rs9294559	chr6:93821257	0.09955806	1	EPHA7	ENSG00000135333.13	Skin - Sun Exposed (Lower leg)	G=0.838	A=0.162	0.13894	0.00011465
rs146803692	rs345736	chr6:93964319	0.00657075	1	EPHA7	ENSG00000135333.13	Heart - Left Ventricle	C=0.254	T=0.746	0.159311	0.00011562
rs146803692	rs168290	chr6:93968162	0.00657075	1	EPHA7	ENSG00000135333.13	Heart - Left Ventricle	C=0.254	T=0.746	0.159311	0.00011562
rs146803692	rs538373	chr6:93732427	0.00746113	1	EPHA7	ENSG00000135333.13	Esophagus - Mucosa	G=0.279	A=0.721	0.20999	0.00011687
rs146803692	rs72926509	chr6:93713733	0.00122095	1	EPHA7	ENSG00000135333.13	Adipose - Subcutaneous	G=0.94	A=0.06	-0.385865	0.00011836
rs146803692	rs9452216	chr6:93753484	0.00603141	1	EPHA7	ENSG00000135333.13	Lung	T=0.761	A=0.239	0.224197	0.00012311
rs146803692	rs9445061	chr6:93755285	0.00599845	1	EPHA7	ENSG00000135333.13	Lung	T=0.762	C=0.238	0.224197	0.00012311
rs146803692	rs9445063	chr6:93755610	0.00599845	1	EPHA7	ENSG00000135333.13	Lung	T=0.762	C=0.238	0.224197	0.00012311
rs146803692	rs960857	chr6:93810834	0.09955806	1	EPHA7	ENSG00000135333.13	Skin - Sun Exposed (Lower leg)	T=0.838	G=0.162	0.138031	0.00012383
rs146803692	rs12189929	chr6:93813254	0.09955806	1	EPHA7	ENSG00000135333.13	Skin - Sun Exposed (Lower leg)	A=0.838	G=0.162	0.138031	0.00012383
rs146803692	rs6454939	chr6:93813414	0.09955806	1	EPHA7	ENSG00000135333.13	Skin - Sun Exposed (Lower leg)	C=0.838	T=0.162	0.138031	0.00012383
rs146803692	rs2224854	chr6:93814357	0.09955806	1	EPHA7	ENSG00000135333.13	Skin - Sun Exposed (Lower leg)	G=0.838	A=0.162	0.138031	0.00012383
rs146803692	rs33936852	chr6:93818131	0.09955806	1	EPHA7	ENSG00000135333.13	Skin - Sun Exposed (Lower leg)	-=0.838	TCT=0.162	0.138031	0.00012383
rs146803692	rs1408290	chr6:93818188	0.09955806	1	EPHA7	ENSG00000135333.13	Skin - Sun Exposed (Lower leg)	A=0.838	T=0.162	0.138031	0.00012383
rs146803692	rs7771872	chr6:93819372	0.09955806	1	EPHA7	ENSG00000135333.13	Skin - Sun Exposed (Lower leg)	A=0.838	G=0.162	0.138031	0.00012383
rs146803692	rs6454940	chr6:93819760	0.09955806	1	EPHA7	ENSG00000135333.13	Skin - Sun Exposed (Lower leg)	G=0.838	A=0.162	0.138031	0.00012383
rs146803692	rs75224735	chr6:93820279	0.09955806	1	EPHA7	ENSG00000135333.13	Skin - Sun Exposed (Lower leg)	-=0.838	CAA=0.162	0.138031	0.00012383
rs146803692	rs9345321	chr6:93789199	0.00764663	1	EPHA7	ENSG00000135333.13	Esophagus - Mucosa	T=0.716	G=0.284	-0.209017	0.00012891
rs146803692	rs9345322	chr6:93790565	0.00764663	1	EPHA7	ENSG00000135333.13	Esophagus - Mucosa	A=0.716	G=0.284	-0.209017	0.00012891
rs146803692	rs9353982	chr6:93802038	0.00764663	1	EPHA7	ENSG00000135333.13	Esophagus - Mucosa	G=0.716	A=0.284	-0.209017	0.00012891
rs146803692	rs7769656	chr6:93757068	0.00599845	1	EPHA7	ENSG00000135333.13	Lung	T=0.762	A=0.238	0.21828	0.00012978
rs146803692	rs16880179	chr6:93889506	0.00326807	1	EPHA7	ENSG00000135333.13	Testis	G=0.855	A=0.145	0.335079	0.00013631
rs146803692	rs59810007	chr6:93891733	0.00326807	1	EPHA7	ENSG00000135333.13	Testis	T=0.855	C=0.145	0.335079	0.00013631
rs146803692	rs6901961	chr6:93892700	0.00326807	1	EPHA7	ENSG00000135333.13	Testis	T=0.855	A=0.145	0.335079	0.00013631
rs146803692	rs60804851	chr6:93894513	0.00326807	1	EPHA7	ENSG00000135333.13	Testis	C=0.855	G=0.145	0.335079	0.00013631
rs146803692	rs6901870	chr6:93895903	0.00326807	1	EPHA7	ENSG00000135333.13	Testis	A=0.855	C=0.145	0.335079	0.00013631
rs146803692	rs9452228	chr6:93770995	0.18898367	1	EPHA7	ENSG00000135333.13	Testis	A=0.908	G=0.092	-0.403164	0.00013767
rs146803692	rs12528177	chr6:93815656	0.10331782	1	EPHA7	ENSG00000135333.13	Skin - Sun Exposed (Lower leg)	C=0.843	T=0.157	0.140911	0.00013946
rs146803692	rs11969676	chr6:93778241	0.19124708	1	EPHA7	ENSG00000135333.13	Adipose - Subcutaneous	G=0.909	A=0.091	0.279245	0.00014178
rs146803692	rs13219509	chr6:93798655	0.00173106	1	EPHA7	ENSG00000135333.13	Adipose - Subcutaneous	T=0.917	C=0.083	0.325674	0.00014298
rs146803692	rs74822095	chr6:93741512	0.00096452	1	EPHA7	ENSG00000135333.13	Lung	C=0.952	T=0.048	-0.471073	0.00014377
rs146803692	rs2057541	chr6:93781036	0.05066222	1	EPHA7	ENSG00000135333.13	Skin - Not Sun Exposed (Suprapubic)	A=0.275	G=0.725	-0.113669	0.00014881
rs146803692	rs7769684	chr6:93793869	0.05066222	1	EPHA7	ENSG00000135333.13	Skin - Not Sun Exposed (Suprapubic)	C=0.275	T=0.725	-0.113669	0.00014881
rs146803692	rs2631562	chr6:93920850	0.00764663	1	EPHA7	ENSG00000135333.13	Testis	T=0.284	C=0.716	-0.28302	0.00014886
rs146803692	rs143801031	chr6:93750441	0.00033089	1	EPHA7	ENSG00000135333.13	Adipose - Subcutaneous	T=0.983	C=0.017	-0.536087	0.00015072
rs146803692	rs58205228	chr6:93778613	0.00764663	1	EPHA7	ENSG00000135333.13	Esophagus - Mucosa	A=0.716	G=0.284	-0.206708	0.00015126
rs146803692	rs9342350	chr6:93798264	0.00764663	1	EPHA7	ENSG00000135333.13	Esophagus - Mucosa	A=0.716	G=0.284	-0.206708	0.00015126
rs146803692	rs9363029	chr6:93798520	0.00764663	1	EPHA7	ENSG00000135333.13	Esophagus - Mucosa	T=0.716	G=0.284	-0.206708	0.00015126
rs146803692	rs9345324	chr6:93803037	0.00764663	1	EPHA7	ENSG00000135333.13	Esophagus - Mucosa	C=0.716	A=0.284	-0.206708	0.00015126
rs146803692	rs6922792	chr6:93887222	0.00326807	1	EPHA7	ENSG00000135333.13	Testis	A=0.855	G=0.145	0.333048	0.00015245
rs146803692	rs10485372	chr6:93821238	0.10254695	1	EPHA7	ENSG00000135333.13	Skin - Sun Exposed (Lower leg)	T=0.842	C=0.158	0.141002	0.00015553
rs146803692	rs117365486	chr6:93820610	0.00063245	1	EPHA7	ENSG00000135333.13	Nerve - Tibial	C=0.968	T=0.032	-0.404506	0.000162
rs146803692	rs148086058	chr6:93820887	0.00063245	1	EPHA7	ENSG00000135333.13	Nerve - Tibial	C=0.968	A=0.032	-0.404506	0.000162
rs146803692	rs9452228	chr6:93770995	0.18898367	1	EPHA7	ENSG00000135333.13	Adipose - Subcutaneous	A=0.908	G=0.092	0.270625	0.00016882
rs146803692	rs76016336	chr6:93919257	0.00043039	1	EPHA7	ENSG00000135333.13	Adipose - Subcutaneous	A=0.978	G=0.022	-0.639741	0.00017071
rs146803692	rs1886252	chr6:93806402	0.09955806	1	EPHA7	ENSG00000135333.13	Skin - Sun Exposed (Lower leg)	C=0.838	T=0.162	0.134315	0.00017117
rs146803692	rs9345323	chr6:93797607	0.00764663	1	EPHA7	ENSG00000135333.13	Esophagus - Mucosa	C=0.716	T=0.284	-0.204207	0.00017144
rs146803692	rs11969676	chr6:93778241	0.19124708	1	EPHA7	ENSG00000135333.13	Testis	G=0.909	A=0.091	-0.401095	0.00017159
rs146803692	rs1570632	chr6:93811908	0.05091553	1	EPHA7	ENSG00000135333.13	Skin - Not Sun Exposed (Suprapubic)	A=0.274	T=0.726	-0.112357	0.00017435
rs146803692	rs1535833	chr6:93769380	0.00742434	1	EPHA7	ENSG00000135333.13	Esophagus - Mucosa	C=0.722	T=0.278	-0.205304	0.00017505
rs146803692	rs77898219	chr6:93824234	0.00742434	1	EPHA7	ENSG00000135333.13	Esophagus - Mucosa	C=0.722	-=0.278	-0.207398	0.00017867

rs146803692	rs9294558	chr6:93812815	0.18247636	1	EPHA7	ENSG00000135333.13	Adipose - Subcutaneous	G=0.905	A=0.095	0.256619	0.00018735
rs146803692	rs9452242	chr6:93812912	0.18247636	1	EPHA7	ENSG00000135333.13	Adipose - Subcutaneous	C=0.905	T=0.095	0.256619	0.00018735
rs146803692	rs34459031	chr6:93813039	0.18247636	1	EPHA7	ENSG00000135333.13	Adipose - Subcutaneous	G=0.905	A=0.095	0.256619	0.00018735
rs146803692	rs9363030	chr6:93806040	0.00746113	1	EPHA7	ENSG00000135333.13	Esophagus - Mucosa	G=0.721	A=0.279	-0.206095	0.00019318
rs146803692	rs4645386	chr6:93802238	0.1845998	1	EPHA7	ENSG00000135333.13	Adipose - Subcutaneous	G=0.906	C=0.094	0.255173	0.00019663
rs146803692	rs13209950	chr6:93805132	0.09955806	1	EPHA7	ENSG00000135333.13	Skin - Sun Exposed (Lower leg)	A=0.838	G=0.162	0.13281	0.00020548
rs146803692	rs1324114	chr6:93793076	0.18898367	1	EPHA7	ENSG00000135333.13	Adipose - Subcutaneous	C=0.908	T=0.092	0.265701	0.00020886
rs146803692	rs1324113	chr6:93793350	0.18898367	1	EPHA7	ENSG00000135333.13	Adipose - Subcutaneous	A=0.908	T=0.092	0.265701	0.00020886
rs146803692	rs182357880	chr6:93879803	0.00013489	1	EPHA7	ENSG00000135333.13	Skin - Sun Exposed (Lower leg)	T=0.993	C=0.007	-0.395624	0.00022421
rs146803692	rs9452240	chr6:93806820	0.01551771	1	EPHA7	ENSG00000135333.13	Nerve - Tibial	G=0.554	T=0.446	0.164067	0.00024188
rs146803692	rs7739743	chr6:93813381	0.01551771	1	EPHA7	ENSG00000135333.13	Nerve - Tibial	G=0.554	A=0.446	0.164067	0.00024188
rs146803692	rs9445075	chr6:93822458	0.01551771	1	EPHA7	ENSG00000135333.13	Nerve - Tibial	T=0.554	A=0.446	0.164067	0.00024188
rs146803692	rs11969246	chr6:93823113	0.01551771	1	EPHA7	ENSG00000135333.13	Nerve - Tibial	G=0.554	T=0.446	0.164067	0.00024188
rs146803692	rs13196582	chr6:93774046	0.01514718	1	EPHA7	ENSG00000135333.13	Nerve - Tibial	C=0.56	T=0.44	0.162798	0.00026347
rs146803692	rs6454936	chr6:93768526	0.01508619	1	EPHA7	ENSG00000135333.13	Nerve - Tibial	C=0.561	T=0.439	0.161655	0.00027184
rs1575539	rs117365486	chr6:93820610	0.0062598	1	EPHA7	ENSG00000135333.13	Adipose - Subcutaneous	C=0.968	T=0.032	-0.679264	8.33E-10
rs1575539	rs148086058	chr6:93820887	0.0062598	1	EPHA7	ENSG00000135333.13	Adipose - Subcutaneous	C=0.968	A=0.032	-0.679264	8.33E-10
rs1575539	rs189136709	chr6:93783798	0.00095171	1	EPHA7	ENSG00000135333.13	Brain - Anterior cingulate cortex (BA24)	G=0.995	A=0.005	1.35876	1.60E-08
rs1575539	rs1386276	chr6:94082749	0.00463452	0.82147294	EPHA7	ENSG00000135333.13	Heart - Left Ventricle	A=0.965	G=0.035	-0.463277	1.18E-07
rs1575539	rs79718749	chr6:94098832	0.00463452	0.82147294	EPHA7	ENSG00000135333.13	Heart - Left Ventricle	T=0.965	C=0.035	-0.463277	1.18E-07
rs1575539	rs12661215	chr6:94099119	0.00463452	0.82147294	EPHA7	ENSG00000135333.13	Heart - Left Ventricle	C=0.965	T=0.035	-0.463277	1.18E-07
rs1575539	rs16871305	chr6:94111173	0.00463452	0.82147294	EPHA7	ENSG00000135333.13	Heart - Left Ventricle	A=0.965	T=0.035	-0.463277	1.18E-07
rs1575539	rs80300952	chr6:94115209	0.00463452	0.82147294	EPHA7	ENSG00000135333.13	Heart - Left Ventricle	T=0.965	C=0.035	-0.463277	1.18E-07
rs1575539	rs146471836	chr6:93817042	0.00405384	0.80473602	EPHA7	ENSG00000135333.13	Adipose - Subcutaneous	C=0.968	T=0.032	-0.617165	8.35E-07
rs1575539	rs141174921	chr6:93733454	0.00405384	0.80473602	EPHA7	ENSG00000135333.13	Adipose - Subcutaneous	A=0.968	G=0.032	-0.598481	1.67E-06
rs1575539	rs117376030	chr6:94203683	0.00505495	1	EPHA7	ENSG00000135333.13	Esophagus - Gastroesophageal Junction	T=0.974	A=0.026	-0.384156	2.51E-06
rs1575539	rs72928597	chr6:93839843	0.01668447	1	EPHA7	ENSG00000135333.13	Lung	A=0.919	G=0.081	0.42955	4.55E-06
rs1575539	rs16870853	chr6:93841320	0.01668447	1	EPHA7	ENSG00000135333.13	Lung	T=0.919	C=0.081	0.42955	4.55E-06
rs1575539	rs1930933	chr6:93842544	0.08134794	1	EPHA7	ENSG00000135333.13	Minor Salivary Gland	C=0.299	T=0.701	0.246092	4.56E-06
rs1575539	rs142723671	chr6:94076414	0.00425987	1	EPHA7	ENSG00000135333.13	Heart - Left Ventricle	G=0.978	C=0.022	-0.495017	4.73E-06
rs1575539	rs147330775	chr6:93915683	0.0011432	1	EPHA7	ENSG00000135333.13	Brain - Anterior cingulate cortex (BA24)	C=0.994	T=0.006	0.808887	6.34E-06
rs1575539	rs16870793	chr6:93828599	0.0162374	1	EPHA7	ENSG00000135333.13	Lung	G=0.921	A=0.079	0.419095	6.62E-06
rs1575539	rs72928592	chr6:93837959	0.01646069	1	EPHA7	ENSG00000135333.13	Lung	T=0.92	C=0.08	0.419095	6.62E-06
rs1575539	rs1575540	chr6:93836577	0.01165705	1	EPHA7	ENSG00000135333.13	Esophagus - Muscularis	G=0.942	A=0.058	0.272463	7.68E-06
rs1575539	rs544639807	chr6:93883657	0.00152731	1	EPHA7	ENSG00000135333.13	Minor Salivary Gland	G=0.992	A=0.008	-1.34812	8.25E-06
rs1575539	rs36114673	chr6:93847056	0.01251379	1	EPHA7	ENSG00000135333.13	Esophagus - Muscularis	A=0.938	T=0.062	0.268662	8.26E-06
rs1575539	rs113888577	chr6:93698093	0.00230019	1	EPHA7	ENSG00000135333.13	Brain - Anterior cingulate cortex (BA24)	C=0.988	T=0.012	0.66365	8.36E-06
rs1575539	rs16870780	chr6:93826505	0.01646069	1	EPHA7	ENSG00000135333.13	Lung	T=0.92	A=0.08	0.385133	9.62E-06
rs1575539	rs1590384	chr6:93837434	0.08134794	1	EPHA7	ENSG00000135333.13	Minor Salivary Gland	C=0.701	G=0.299	-0.24131	1.00E-05
rs1575539	rs1924474	chr6:93837991	0.08134794	1	EPHA7	ENSG00000135333.13	Minor Salivary Gland	C=0.701	T=0.299	-0.24131	1.00E-05
rs1575539	rs10944652	chr6:93838348	0.08134794	1	EPHA7	ENSG00000135333.13	Minor Salivary Gland	C=0.701	G=0.299	-0.24131	1.00E-05
rs1575539	rs12204186	chr6:93838665	0.08134794	1	EPHA7	ENSG00000135333.13	Minor Salivary Gland	C=0.701	T=0.299	-0.24131	1.00E-05
rs1575539	rs633279	chr6:93840705	0.08134794	1	EPHA7	ENSG00000135333.13	Minor Salivary Gland	A=0.299	T=0.701	0.24131	1.00E-05
rs1575539	rs634060	chr6:93840849	0.08134794	1	EPHA7	ENSG00000135333.13	Minor Salivary Gland	A=0.299	G=0.701	0.24131	1.00E-05
rs1575539	rs568957	chr6:93840855	0.08134794	1	EPHA7	ENSG00000135333.13	Minor Salivary Gland	G=0.299	T=0.701	0.24131	1.00E-05
rs1575539	rs34544163	chr6:93841565	0.08134794	1	EPHA7	ENSG00000135333.13	Minor Salivary Gland	-=0.299	A=0.701	0.24131	1.00E-05
rs1575539	rs562379	chr6:93841592	0.08134794	1	EPHA7	ENSG00000135333.13	Minor Salivary Gland	G=0.299	A=0.701	0.24131	1.00E-05
rs1575539	rs560731	chr6:93841734	0.08134794	1	EPHA7	ENSG00000135333.13	Minor Salivary Gland	A=0.299	G=0.701	0.24131	1.00E-05
rs1575539	rs650642	chr6:93842232	0.08134794	1	EPHA7	ENSG00000135333.13	Minor Salivary Gland	G=0.299	T=0.701	0.24131	1.00E-05
rs1575539	rs650747	chr6:93842298	0.08134794	1	EPHA7	ENSG00000135333.13	Minor Salivary Gland	T=0.299	C=0.701	0.24131	1.00E-05
rs1575539	rs1930934	chr6:93842533	0.08134794	1	EPHA7	ENSG00000135333.13	Minor Salivary Gland	A=0.299	G=0.701	0.24131	1.00E-05

rs1575539	rs9353987	chr6:93843588	0.08134794	1	EPHA7	ENSG00000135333.13	Minor Salivary Gland	A=0.299	G=0.701	0.24131	1.00E-05
rs1575539	rs9363034	chr6:93844136	0.08134794	1	EPHA7	ENSG00000135333.13	Minor Salivary Gland	G=0.299	A=0.701	0.24131	1.00E-05
rs1575539	rs9353989	chr6:93844383	0.08134794	1	EPHA7	ENSG00000135333.13	Minor Salivary Gland	G=0.299	T=0.701	0.24131	1.00E-05
rs1575539	rs76282783	chr6:93576050	0.00601496	0.85122745	EPHA7	ENSG00000135333.13	Lung	C=0.958	A=0.042	0.702179	1.18E-05
rs1575539	rs199888991	chr6:93784078	0.00095171	1	EPHA7	ENSG00000135333.13	Adipose - Subcutaneous	-=0.995	T=0.005	-0.796053	1.44E-05
rs1575539	rs111429740	chr6:93838453	0.01668447	1	EPHA7	ENSG00000135333.13	Lung	G=0.919	A=0.081	0.383525	1.55E-05
rs1575539	rs16870789	chr6:93827963	0.01646069	1	EPHA7	ENSG00000135333.13	Lung	C=0.92	T=0.08	0.374368	1.81E-05
rs1575539	rs145810085	chr6:93833819	0.00152731	1	EPHA7	ENSG00000135333.13	Adipose - Subcutaneous	G=0.992	A=0.008	-0.735109	1.88E-05
rs1575539	rs7774823	chr6:93830545	0.08057883	1	EPHA7	ENSG00000135333.13	Minor Salivary Gland	G=0.703	A=0.297	-0.232136	1.89E-05
rs1575539	rs1951907	chr6:93831192	0.08057883	1	EPHA7	ENSG00000135333.13	Minor Salivary Gland	T=0.703	C=0.297	-0.232136	1.89E-05
rs1575539	rs35306488	chr6:93832227	0.07943329	1	EPHA7	ENSG00000135333.13	Minor Salivary Gland	-=0.706	T=0.294	-0.232136	1.89E-05
rs1575539	rs10944648	chr6:93832280	0.08057883	1	EPHA7	ENSG00000135333.13	Minor Salivary Gland	A=0.703	G=0.297	-0.232136	1.89E-05
rs1575539	rs7751375	chr6:93832923	0.08096284	1	EPHA7	ENSG00000135333.13	Minor Salivary Gland	C=0.702	A=0.298	-0.232136	1.89E-05
rs1575539	rs7757292	chr6:93834165	0.08057883	1	EPHA7	ENSG00000135333.13	Minor Salivary Gland	C=0.703	A=0.297	-0.232136	1.89E-05
rs1575539	rs16870850	chr6:93841079	0.01758444	1	EPHA7	ENSG00000135333.13	Lung	T=0.916	C=0.084	0.361979	2.02E-05
rs1575539	rs12528846	chr6:93828842	0.01646069	1	EPHA7	ENSG00000135333.13	Lung	A=0.92	G=0.08	0.377147	2.03E-05
rs1575539	rs12527296	chr6:93829143	0.01646069	1	EPHA7	ENSG00000135333.13	Lung	T=0.92	C=0.08	0.377147	2.03E-05
rs1575539	rs72928575	chr6:93829399	0.01668447	1	EPHA7	ENSG00000135333.13	Lung	G=0.919	T=0.081	0.377147	2.03E-05
rs1575539	rs72928579	chr6:93829658	0.01646069	1	EPHA7	ENSG00000135333.13	Lung	G=0.92	A=0.08	0.377147	2.03E-05
rs1575539	rs77007574	chr6:93829960	0.01646069	1	EPHA7	ENSG00000135333.13	Lung	T=0.92	C=0.08	0.377147	2.03E-05
rs1575539	rs72928582	chr6:93830015	0.01646069	1	EPHA7	ENSG00000135333.13	Lung	G=0.92	A=0.08	0.377147	2.03E-05
rs1575539	rs16870801	chr6:93830951	0.01646069	1	EPHA7	ENSG00000135333.13	Lung	T=0.92	C=0.08	0.377147	2.03E-05
rs1575539	rs16870805	chr6:93834075	0.01646069	1	EPHA7	ENSG00000135333.13	Lung	G=0.92	T=0.08	0.377147	2.03E-05
rs1575539	rs150304721	chr6:93835801	0.01690873	1	EPHA7	ENSG00000135333.13	Lung	A=0.918	-=0.082	0.361316	2.04E-05
rs1575539	rs16870855	chr6:93841440	0.01668447	1	EPHA7	ENSG00000135333.13	Lung	C=0.919	T=0.081	0.4288	2.14E-05
rs1575539	rs7772178	chr6:93849608	0.02531851	1	EPHA7	ENSG00000135333.13	Testis	C=0.883	T=0.117	0.425527	2.47E-05
rs1575539	rs73758259	chr6:93828724	0.01713348	1	EPHA7	ENSG00000135333.13	Lung	G=0.917	T=0.083	0.354112	2.72E-05
rs1575539	rs16870796	chr6:93828781	0.01646069	1	EPHA7	ENSG00000135333.13	Lung	T=0.92	G=0.08	0.369069	2.76E-05
rs1575539	rs72928577	chr6:93829400	0.01668447	1	EPHA7	ENSG00000135333.13	Lung	C=0.919	T=0.081	0.369069	2.76E-05
rs1575539	rs117717826	chr6:94036945	0.00485557	1	EPHA7	ENSG00000135333.13	Heart - Left Ventricle	T=0.975	A=0.025	-0.389622	2.96E-05
rs1575539	rs71558449	chr6:93828589	0.00646205	1	EPHA7	ENSG00000135333.13	Esophagus - Muscularis	G=0.967	A=0.033	0.272458	3.14E-05
rs1575539	rs6922405	chr6:93842847	0.01668447	1	EPHA7	ENSG00000135333.13	Lung	T=0.919	C=0.081	0.370721	3.25E-05
rs1575539	rs72930416	chr6:93844150	0.01668447	1	EPHA7	ENSG00000135333.13	Lung	T=0.919	C=0.081	0.370721	3.25E-05
rs1575539	rs16870846	chr6:93840909	0.01668447	1	EPHA7	ENSG00000135333.13	Lung	C=0.919	G=0.081	0.371767	3.40E-05
rs1575539	rs1319460	chr6:93846637	0.05662891	0.9739648	EPHA7	ENSG00000135333.13	Minor Salivary Gland	C=0.239	T=0.761	0.230876	3.54E-05
rs1575539	rs538751	chr6:93848400	0.05662891	0.9739648	EPHA7	ENSG00000135333.13	Minor Salivary Gland	A=0.239	C=0.761	0.230876	3.54E-05
rs1575539	rs11422146	chr6:93849652	0.05630683	0.97385587	EPHA7	ENSG00000135333.13	Minor Salivary Gland	-=0.238	T=0.762	0.230876	3.54E-05
rs1575539	rs1319460	chr6:93846637	0.05662891	0.9739648	EPHA7	ENSG00000135333.13	Testis	C=0.239	T=0.761	-0.330556	3.66E-05
rs1575539	rs538751	chr6:93848400	0.05662891	0.9739648	EPHA7	ENSG00000135333.13	Testis	A=0.239	C=0.761	-0.330556	3.66E-05
rs1575539	rs11422146	chr6:93849652	0.05630683	0.97385587	EPHA7	ENSG00000135333.13	Testis	-=0.238	T=0.762	-0.330556	3.66E-05
rs1575539	rs140182812	chr6:93804212	0.00152731	1	EPHA7	ENSG00000135333.13	Adipose - Subcutaneous	G=0.992	A=0.008	-0.729088	4.39E-05
rs1575539	rs41273625	chr6:93952851	0.01032934	0.9008183	EPHA7	ENSG00000135333.13	Esophagus - Muscularis	C=0.937	G=0.063	0.224035	5.17E-05
rs1575539	rs12530331	chr6:93830460	0.01668447	1	EPHA7	ENSG00000135333.13	Lung	G=0.919	C=0.081	0.35653	5.33E-05
rs1575539	rs74468395	chr6:94017659	0.00646205	1	EPHA7	ENSG00000135333.13	Heart - Left Ventricle	C=0.967	T=0.033	-0.353	6.19E-05
rs1575539	rs117256127	chr6:93852714	0.0046566	1	EPHA7	ENSG00000135333.13	Adipose - Subcutaneous	T=0.976	C=0.024	-0.674139	6.42E-05
rs1575539	rs117425371	chr6:93852717	0.0046566	1	EPHA7	ENSG00000135333.13	Adipose - Subcutaneous	A=0.976	G=0.024	-0.674139	6.42E-05
rs1575539	rs145519743	chr6:93884032	0.00485557	1	EPHA7	ENSG00000135333.13	Adipose - Subcutaneous	G=0.975	A=0.025	-0.674139	6.42E-05
rs1575539	rs9351349	chr6:93832104	0.04152041	1	EPHA7	ENSG00000135333.13	Adipose - Subcutaneous	G=0.179	A=0.821	0.228214	6.48E-05
rs1575539	rs147330775	chr6:93915683	0.0011432	1	EPHA7	ENSG00000135333.13	Skin - Sun Exposed (Lower leg)	C=0.994	T=0.006	-0.47159	6.56E-05
rs1575539	rs117109139	chr6:93861398	0.00747958	1	EPHA7	ENSG00000135333.13	Esophagus - Muscularis	C=0.962	G=0.038	0.257424	7.15E-05
rs1575539	rs650711	chr6:93842280	0.04152041	1	EPHA7	ENSG00000135333.13	Adipose - Subcutaneous	T=0.821	G=0.179	-0.226702	7.44E-05

rs1575539	rs768382	chr6:93826168	0.04123981	1	EPHA7	ENSG00000135333.13	Adipose - Subcutaneous	T=0.178	C=0.822	0.223191	7.87E-05
rs1575539	rs41273625	chr6:93952851	0.01032934	0.9008183	EPHA7	ENSG00000135333.13	Esophagus - Gastroesophageal Junction	C=0.937	G=0.063	0.249826	0.00010545
rs1575539	rs138022068	chr6:94069111	0.00722283	0.86982402	EPHA7	ENSG00000135333.13	Heart - Left Ventricle	C=0.952	-=0.048	-0.300501	0.00011172
rs1575539	rs192604147	chr6:94240629	0.00171995	1	EPHA7	ENSG00000135333.13	Heart - Left Ventricle	A=0.991	G=0.009	-0.514265	0.00011598
rs1575539	rs143801031	chr6:93750441	0.00327508	1	EPHA7	ENSG00000135333.13	Adipose - Subcutaneous	T=0.983	C=0.017	-0.536087	0.00015072
rs1575539	rs117365486	chr6:93820610	0.0062598	1	EPHA7	ENSG00000135333.13	Nerve - Tibial	C=0.968	T=0.032	-0.404506	0.000162
rs1575539	rs148086058	chr6:93820887	0.0062598	1	EPHA7	ENSG00000135333.13	Nerve - Tibial	C=0.968	A=0.032	-0.404506	0.000162
rs1575539	rs182357880	chr6:93879803	0.00133506	1	EPHA7	ENSG00000135333.13	Skin - Sun Exposed (Lower leg)	T=0.993	C=0.007	-0.395624	0.00022421
rs16870893	rs117365486	chr6:93820610	0.0062598	1	EPHA7	ENSG00000135333.13	Adipose - Subcutaneous	C=0.968	T=0.032	-0.679264	8.33E-10
rs16870893	rs148086058	chr6:93820887	0.0062598	1	EPHA7	ENSG00000135333.13	Adipose - Subcutaneous	C=0.968	A=0.032	-0.679264	8.33E-10
rs16870893	rs189136709	chr6:93783798	0.00095171	1	EPHA7	ENSG00000135333.13	Brain - Anterior cingulate cortex (BA24)	G=0.995	A=0.005	1.35876	1.60E-08
rs16870893	rs1386276	chr6:94082749	0.00463452	0.82147294	EPHA7	ENSG00000135333.13	Heart - Left Ventricle	A=0.965	G=0.035	-0.463277	1.18E-07
rs16870893	rs79718749	chr6:94098832	0.00463452	0.82147294	EPHA7	ENSG00000135333.13	Heart - Left Ventricle	T=0.965	C=0.035	-0.463277	1.18E-07
rs16870893	rs12661215	chr6:94099119	0.00463452	0.82147294	EPHA7	ENSG00000135333.13	Heart - Left Ventricle	C=0.965	T=0.035	-0.463277	1.18E-07
rs16870893	rs16871305	chr6:94111173	0.00463452	0.82147294	EPHA7	ENSG00000135333.13	Heart - Left Ventricle	A=0.965	T=0.035	-0.463277	1.18E-07
rs16870893	rs80300952	chr6:94115209	0.00463452	0.82147294	EPHA7	ENSG00000135333.13	Heart - Left Ventricle	T=0.965	C=0.035	-0.463277	1.18E-07
rs16870893	rs146471836	chr6:93817042	0.00405384	0.80473602	EPHA7	ENSG00000135333.13	Adipose - Subcutaneous	C=0.968	T=0.032	-0.617165	8.35E-07
rs16870893	rs141174921	chr6:93733454	0.00405384	0.80473602	EPHA7	ENSG00000135333.13	Adipose - Subcutaneous	A=0.968	G=0.032	-0.598481	1.67E-06
rs16870893	rs117376030	chr6:94203683	0.00505495	1	EPHA7	ENSG00000135333.13	Esophagus - Gastroesophageal Junction	T=0.974	A=0.026	-0.384156	2.51E-06
rs16870893	rs72928597	chr6:93839843	0.01668447	1	EPHA7	ENSG00000135333.13	Lung	A=0.919	G=0.081	0.42955	4.55E-06
rs16870893	rs16870853	chr6:93841320	0.01668447	1	EPHA7	ENSG00000135333.13	Lung	T=0.919	C=0.081	0.42955	4.55E-06
rs16870893	rs1930933	chr6:93842544	0.08134794	1	EPHA7	ENSG00000135333.13	Minor Salivary Gland	C=0.299	T=0.701	0.246092	4.56E-06
rs16870893	rs142723671	chr6:94076414	0.00425987	1	EPHA7	ENSG00000135333.13	Heart - Left Ventricle	G=0.978	C=0.022	-0.495017	4.73E-06
rs16870893	rs147330775	chr6:93915683	0.0011432	1	EPHA7	ENSG00000135333.13	Brain - Anterior cingulate cortex (BA24)	C=0.994	T=0.006	0.808887	6.34E-06
rs16870893	rs16870793	chr6:93828599	0.0162374	1	EPHA7	ENSG00000135333.13	Lung	G=0.921	A=0.079	0.419095	6.62E-06
rs16870893	rs72928592	chr6:93837959	0.01646069	1	EPHA7	ENSG00000135333.13	Lung	T=0.92	C=0.08	0.419095	6.62E-06
rs16870893	rs1575540	chr6:93836577	0.01165705	1	EPHA7	ENSG00000135333.13	Esophagus - Muscularis	G=0.942	A=0.058	0.272463	7.68E-06
rs16870893	rs544639807	chr6:93883657	0.00152731	1	EPHA7	ENSG00000135333.13	Minor Salivary Gland	G=0.992	A=0.008	-1.34812	8.25E-06
rs16870893	rs36114673	chr6:93847056	0.01251379	1	EPHA7	ENSG00000135333.13	Esophagus - Muscularis	A=0.938	T=0.062	0.268662	8.26E-06
rs16870893	rs113888577	chr6:93698093	0.00230019	1	EPHA7	ENSG00000135333.13	Brain - Anterior cingulate cortex (BA24)	C=0.988	T=0.012	0.66365	8.36E-06
rs16870893	rs16870780	chr6:93826505	0.01646069	1	EPHA7	ENSG00000135333.13	Lung	T=0.92	A=0.08	0.385133	9.62E-06
rs16870893	rs1590384	chr6:93837434	0.08134794	1	EPHA7	ENSG00000135333.13	Minor Salivary Gland	C=0.701	G=0.299	-0.24131	1.00E-05
rs16870893	rs1924474	chr6:93837991	0.08134794	1	EPHA7	ENSG00000135333.13	Minor Salivary Gland	C=0.701	T=0.299	-0.24131	1.00E-05
rs16870893	rs10944652	chr6:93838348	0.08134794	1	EPHA7	ENSG00000135333.13	Minor Salivary Gland	C=0.701	G=0.299	-0.24131	1.00E-05
rs16870893	rs12204186	chr6:93838665	0.08134794	1	EPHA7	ENSG00000135333.13	Minor Salivary Gland	C=0.701	T=0.299	-0.24131	1.00E-05
rs16870893	rs633279	chr6:93840705	0.08134794	1	EPHA7	ENSG00000135333.13	Minor Salivary Gland	A=0.299	T=0.701	0.24131	1.00E-05
rs16870893	rs634060	chr6:93840849	0.08134794	1	EPHA7	ENSG00000135333.13	Minor Salivary Gland	A=0.299	G=0.701	0.24131	1.00E-05
rs16870893	rs568957	chr6:93840855	0.08134794	1	EPHA7	ENSG00000135333.13	Minor Salivary Gland	G=0.299	T=0.701	0.24131	1.00E-05
rs16870893	rs34544163	chr6:93841565	0.08134794	1	EPHA7	ENSG00000135333.13	Minor Salivary Gland	-=0.299	A=0.701	0.24131	1.00E-05
rs16870893	rs562379	chr6:93841592	0.08134794	1	EPHA7	ENSG00000135333.13	Minor Salivary Gland	G=0.299	A=0.701	0.24131	1.00E-05
rs16870893	rs560731	chr6:93841734	0.08134794	1	EPHA7	ENSG00000135333.13	Minor Salivary Gland	A=0.299	G=0.701	0.24131	1.00E-05
rs16870893	rs650642	chr6:93842232	0.08134794	1	EPHA7	ENSG00000135333.13	Minor Salivary Gland	G=0.299	T=0.701	0.24131	1.00E-05
rs16870893	rs650747	chr6:93842298	0.08134794	1	EPHA7	ENSG00000135333.13	Minor Salivary Gland	T=0.299	C=0.701	0.24131	1.00E-05
rs16870893	rs1930934	chr6:93842533	0.08134794	1	EPHA7	ENSG00000135333.13	Minor Salivary Gland	A=0.299	G=0.701	0.24131	1.00E-05
rs16870893	rs9353987	chr6:93843588	0.08134794	1	EPHA7	ENSG00000135333.13	Minor Salivary Gland	A=0.299	G=0.701	0.24131	1.00E-05
rs16870893	rs9363034	chr6:93844136	0.08134794	1	EPHA7	ENSG00000135333.13	Minor Salivary Gland	G=0.299	A=0.701	0.24131	1.00E-05
rs16870893	rs9353989	chr6:93844383	0.08134794	1	EPHA7	ENSG00000135333.13	Minor Salivary Gland	G=0.299	T=0.701	0.24131	1.00E-05
rs16870893	rs76282783	chr6:93576050	0.00601496	0.85122745	EPHA7	ENSG00000135333.13	Lung	C=0.958	A=0.042	0.702179	1.18E-05
rs16870893	rs199888991	chr6:93784078	0.00095171	1	EPHA7	ENSG00000135333.13	Adipose - Subcutaneous	-=0.995	T=0.005	-0.796053	1.44E-05
rs16870893	rs111429740	chr6:93838453	0.01668447	1	EPHA7	ENSG00000135333.13	Lung	G=0.919	A=0.081	0.383525	1.55E-05
rs16870893	rs16870789	chr6:93827963	0.01646069	1	EPHA7	ENSG00000135333.13	Lung	C=0.92	T=0.08	0.374368	1.81E-05

rs16870893	rs145810085	chr6:93833819	0.00152731	1	EPHA7	ENSG00000135333.13	Adipose - Subcutaneous	G=0.992	A=0.008	-0.735109	1.88E-05
rs16870893	rs7774823	chr6:93830545	0.08057883	1	EPHA7	ENSG00000135333.13	Minor Salivary Gland	G=0.703	A=0.297	-0.232136	1.89E-05
rs16870893	rs1951907	chr6:93831192	0.08057883	1	EPHA7	ENSG00000135333.13	Minor Salivary Gland	T=0.703	C=0.297	-0.232136	1.89E-05
rs16870893	rs35306488	chr6:93832227	0.07943329	1	EPHA7	ENSG00000135333.13	Minor Salivary Gland	-=0.706	T=0.294	-0.232136	1.89E-05
rs16870893	rs10944648	chr6:93832280	0.08057883	1	EPHA7	ENSG00000135333.13	Minor Salivary Gland	A=0.703	G=0.297	-0.232136	1.89E-05
rs16870893	rs7751375	chr6:93832923	0.08096284	1	EPHA7	ENSG00000135333.13	Minor Salivary Gland	C=0.702	A=0.298	-0.232136	1.89E-05
rs16870893	rs7757292	chr6:93834165	0.08057883	1	EPHA7	ENSG00000135333.13	Minor Salivary Gland	C=0.703	A=0.297	-0.232136	1.89E-05
rs16870893	rs16870850	chr6:93841079	0.01758444	1	EPHA7	ENSG00000135333.13	Lung	T=0.916	C=0.084	0.361979	2.02E-05
rs16870893	rs12528846	chr6:93828842	0.01646069	1	EPHA7	ENSG00000135333.13	Lung	A=0.92	G=0.08	0.377147	2.03E-05
rs16870893	rs12527296	chr6:93829143	0.01646069	1	EPHA7	ENSG00000135333.13	Lung	T=0.92	C=0.08	0.377147	2.03E-05
rs16870893	rs72928575	chr6:93829399	0.01668447	1	EPHA7	ENSG00000135333.13	Lung	G=0.919	T=0.081	0.377147	2.03E-05
rs16870893	rs72928579	chr6:93829658	0.01646069	1	EPHA7	ENSG00000135333.13	Lung	G=0.92	A=0.08	0.377147	2.03E-05
rs16870893	rs77007574	chr6:93829960	0.01646069	1	EPHA7	ENSG00000135333.13	Lung	T=0.92	C=0.08	0.377147	2.03E-05
rs16870893	rs72928582	chr6:93830015	0.01646069	1	EPHA7	ENSG00000135333.13	Lung	G=0.92	A=0.08	0.377147	2.03E-05
rs16870893	rs16870801	chr6:93830951	0.01646069	1	EPHA7	ENSG00000135333.13	Lung	T=0.92	C=0.08	0.377147	2.03E-05
rs16870893	rs16870805	chr6:93834075	0.01646069	1	EPHA7	ENSG00000135333.13	Lung	G=0.92	T=0.08	0.377147	2.03E-05
rs16870893	rs150304721	chr6:93835801	0.01690873	1	EPHA7	ENSG00000135333.13	Lung	A=0.918	-=0.082	0.361316	2.04E-05
rs16870893	rs16870855	chr6:93841440	0.01668447	1	EPHA7	ENSG00000135333.13	Lung	C=0.919	T=0.081	0.4288	2.14E-05
rs16870893	rs7772178	chr6:93849608	0.02531851	1	EPHA7	ENSG00000135333.13	Testis	C=0.883	T=0.117	0.425527	2.47E-05
rs16870893	rs73758259	chr6:93828724	0.01713348	1	EPHA7	ENSG00000135333.13	Lung	G=0.917	T=0.083	0.354112	2.72E-05
rs16870893	rs16870796	chr6:93828781	0.01646069	1	EPHA7	ENSG00000135333.13	Lung	T=0.92	G=0.08	0.369069	2.76E-05
rs16870893	rs72928577	chr6:93829400	0.01668447	1	EPHA7	ENSG00000135333.13	Lung	C=0.919	T=0.081	0.369069	2.76E-05
rs16870893	rs117717826	chr6:94036945	0.00485557	1	EPHA7	ENSG00000135333.13	Heart - Left Ventricle	T=0.975	A=0.025	-0.389622	2.96E-05
rs16870893	rs71558449	chr6:93828589	0.00646205	1	EPHA7	ENSG00000135333.13	Esophagus - Muscularis	G=0.967	A=0.033	0.272458	3.14E-05
rs16870893	rs6922405	chr6:93842847	0.01668447	1	EPHA7	ENSG00000135333.13	Lung	T=0.919	C=0.081	0.370721	3.25E-05
rs16870893	rs72930416	chr6:93844150	0.01668447	1	EPHA7	ENSG00000135333.13	Lung	T=0.919	C=0.081	0.370721	3.25E-05
rs16870893	rs16870846	chr6:93840909	0.01668447	1	EPHA7	ENSG00000135333.13	Lung	C=0.919	G=0.081	0.371767	3.40E-05
rs16870893	rs1319460	chr6:93846637	0.05969688	1	EPHA7	ENSG00000135333.13	Minor Salivary Gland	C=0.239	T=0.761	0.230876	3.54E-05
rs16870893	rs538751	chr6:93848400	0.05969688	1	EPHA7	ENSG00000135333.13	Minor Salivary Gland	A=0.239	C=0.761	0.230876	3.54E-05
rs16870893	rs11422146	chr6:93849652	0.05937064	1	EPHA7	ENSG00000135333.13	Minor Salivary Gland	-=0.238	T=0.762	0.230876	3.54E-05
rs16870893	rs1319460	chr6:93846637	0.05969688	1	EPHA7	ENSG00000135333.13	Testis	C=0.239	T=0.761	-0.330556	3.66E-05
rs16870893	rs538751	chr6:93848400	0.05969688	1	EPHA7	ENSG00000135333.13	Testis	A=0.239	C=0.761	-0.330556	3.66E-05
rs16870893	rs11422146	chr6:93849652	0.05937064	1	EPHA7	ENSG00000135333.13	Testis	-=0.238	T=0.762	-0.330556	3.66E-05
rs16870893	rs140182812	chr6:93804212	0.00152731	1	EPHA7	ENSG00000135333.13	Adipose - Subcutaneous	G=0.992	A=0.008	-0.729088	4.39E-05
rs16870893	rs41273625	chr6:93952851	0.01032934	0.9008183	EPHA7	ENSG00000135333.13	Esophagus - Muscularis	C=0.937	G=0.063	0.224035	5.17E-05
rs16870893	rs12530331	chr6:93830460	0.01668447	1	EPHA7	ENSG00000135333.13	Lung	G=0.919	C=0.081	0.35653	5.33E-05
rs16870893	rs74468395	chr6:94017659	0.00646205	1	EPHA7	ENSG00000135333.13	Heart - Left Ventricle	C=0.967	T=0.033	-0.353	6.19E-05
rs16870893	rs117256127	chr6:93852714	0.0046566	1	EPHA7	ENSG00000135333.13	Adipose - Subcutaneous	T=0.976	C=0.024	-0.674139	6.42E-05
rs16870893	rs117425371	chr6:93852717	0.0046566	1	EPHA7	ENSG00000135333.13	Adipose - Subcutaneous	A=0.976	G=0.024	-0.674139	6.42E-05
rs16870893	rs145519743	chr6:93884032	0.00485557	1	EPHA7	ENSG00000135333.13	Adipose - Subcutaneous	G=0.975	A=0.025	-0.674139	6.42E-05
rs16870893	rs9351349	chr6:93832104	0.0386878	0.9652864	EPHA7	ENSG00000135333.13	Adipose - Subcutaneous	G=0.179	A=0.821	0.228214	6.48E-05
rs16870893	rs147330775	chr6:93915683	0.0011432	1	EPHA7	ENSG00000135333.13	Skin - Sun Exposed (Lower leg)	C=0.994	T=0.006	-0.47159	6.56E-05
rs16870893	rs117109139	chr6:93861398	0.00747958	1	EPHA7	ENSG00000135333.13	Esophagus - Muscularis	C=0.962	G=0.038	0.257424	7.15E-05
rs16870893	rs650711	chr6:93842280	0.0386878	0.9652864	EPHA7	ENSG00000135333.13	Adipose - Subcutaneous	T=0.821	G=0.179	-0.226702	7.44E-05
rs16870893	rs768382	chr6:93826168	0.03841091	0.96509247	EPHA7	ENSG00000135333.13	Adipose - Subcutaneous	T=0.178	C=0.822	0.223191	7.87E-05
rs16870893	rs1324110	chr6:93913200	0.15736501	0.80128613	EPHA7	ENSG00000135333.13	Heart - Atrial Appendage	G=0.563	C=0.437	0.0819979	9.78E-05
rs16870893	rs41273625	chr6:93952851	0.01032934	0.9008183	EPHA7	ENSG00000135333.13	Esophagus - Gastroesophageal Junction	C=0.937	G=0.063	0.249826	0.00010545
rs16870893	rs138022068	chr6:94069111	0.00722283	0.86982402	EPHA7	ENSG00000135333.13	Heart - Left Ventricle	C=0.952	-=0.048	-0.300501	0.00011172
rs16870893	rs192604147	chr6:94240629	0.00171995	1	EPHA7	ENSG00000135333.13	Heart - Left Ventricle	A=0.991	G=0.009	-0.514265	0.00011598
rs16870893	rs143801031	chr6:93750441	0.00327508	1	EPHA7	ENSG00000135333.13	Adipose - Subcutaneous	T=0.983	C=0.017	-0.536087	0.00015072
rs16870893	rs117365486	chr6:93820610	0.0062598	1	EPHA7	ENSG00000135333.13	Nerve - Tibial	C=0.968	T=0.032	-0.404506	0.000162

rs16870893	rs148086058	chr6:93820887	0.0062598	1	EPHA7	ENSG00000135333.13	Nerve - Tibial	C=0.968	A=0.032	-0.404506	0.000162
rs16870893	rs182357880	chr6:93879803	0.00133506	1	EPHA7	ENSG00000135333.13	Skin - Sun Exposed (Lower leg)	T=0.993	C=0.007	-0.395624	0.00022421
rs16870897	rs1535833	chr6:93769380	0.04367934	0.80709492	EPHA7	ENSG00000135333.13	Adipose - Subcutaneous	C=0.722	T=0.278	-0.770455	1.90E-63
rs16870897	rs58205228	chr6:93778613	0.0454394	0.81114188	EPHA7	ENSG00000135333.13	Adipose - Subcutaneous	A=0.716	G=0.284	-0.767837	2.77E-63
rs16870897	rs9345321	chr6:93789199	0.0454394	0.81114188	EPHA7	ENSG00000135333.13	Adipose - Subcutaneous	T=0.716	G=0.284	-0.767837	2.77E-63
rs16870897	rs9345322	chr6:93790565	0.0454394	0.81114188	EPHA7	ENSG00000135333.13	Adipose - Subcutaneous	A=0.716	G=0.284	-0.767837	2.77E-63
rs16870897	rs9345323	chr6:93797607	0.0454394	0.81114188	EPHA7	ENSG00000135333.13	Adipose - Subcutaneous	C=0.716	T=0.284	-0.767837	2.77E-63
rs16870897	rs9342350	chr6:93798264	0.0454394	0.81114188	EPHA7	ENSG00000135333.13	Adipose - Subcutaneous	A=0.716	G=0.284	-0.767837	2.77E-63
rs16870897	rs9363029	chr6:93798520	0.0454394	0.81114188	EPHA7	ENSG00000135333.13	Adipose - Subcutaneous	T=0.716	G=0.284	-0.767837	2.77E-63
rs16870897	rs9353982	chr6:93802038	0.0454394	0.81114188	EPHA7	ENSG00000135333.13	Adipose - Subcutaneous	G=0.716	A=0.284	-0.767837	2.77E-63
rs16870897	rs9345324	chr6:93803037	0.0454394	0.81114188	EPHA7	ENSG00000135333.13	Adipose - Subcutaneous	C=0.716	A=0.284	-0.767837	2.77E-63
rs16870897	rs1570631	chr6:93811876	0.04455544	0.80913985	EPHA7	ENSG00000135333.13	Adipose - Subcutaneous	T=0.719	C=0.281	-0.759574	1.45E-59
rs16870897	rs77898219	chr6:93824234	0.04367934	0.80709492	EPHA7	ENSG00000135333.13	Adipose - Subcutaneous	C=0.722	-=0.278	-0.752617	1.58E-59
rs16870897	rs9363030	chr6:93806040	0.04397051	0.80778141	EPHA7	ENSG00000135333.13	Adipose - Subcutaneous	G=0.721	A=0.279	-0.757344	1.59E-59
rs16870897	rs9363030	chr6:93806040	0.04397051	0.80778141	EPHA7	ENSG00000135333.13	Lung	G=0.721	A=0.279	-0.467048	1.04E-18
rs16870897	rs1570631	chr6:93811876	0.04455544	0.80913985	EPHA7	ENSG00000135333.13	Lung	T=0.719	C=0.281	-0.467048	1.04E-18
rs16870897	rs58205228	chr6:93778613	0.0454394	0.81114188	EPHA7	ENSG00000135333.13	Lung	A=0.716	G=0.284	-0.459589	1.54E-18
rs16870897	rs9345321	chr6:93789199	0.0454394	0.81114188	EPHA7	ENSG00000135333.13	Lung	T=0.716	G=0.284	-0.459589	1.54E-18
rs16870897	rs9345322	chr6:93790565	0.0454394	0.81114188	EPHA7	ENSG00000135333.13	Lung	A=0.716	G=0.284	-0.459589	1.54E-18
rs16870897	rs9345323	chr6:93797607	0.0454394	0.81114188	EPHA7	ENSG00000135333.13	Lung	C=0.716	T=0.284	-0.459589	1.54E-18
rs16870897	rs9342350	chr6:93798264	0.0454394	0.81114188	EPHA7	ENSG00000135333.13	Lung	A=0.716	G=0.284	-0.459589	1.54E-18
rs16870897	rs9363029	chr6:93798520	0.0454394	0.81114188	EPHA7	ENSG00000135333.13	Lung	T=0.716	G=0.284	-0.459589	1.54E-18
rs16870897	rs9353982	chr6:93802038	0.0454394	0.81114188	EPHA7	ENSG00000135333.13	Lung	G=0.716	A=0.284	-0.459589	1.54E-18
rs16870897	rs9345324	chr6:93803037	0.0454394	0.81114188	EPHA7	ENSG00000135333.13	Lung	C=0.716	A=0.284	-0.459589	1.54E-18
rs16870897	rs77898219	chr6:93824234	0.04367934	0.80709492	EPHA7	ENSG00000135333.13	Lung	C=0.722	-=0.278	-0.456134	5.88E-18
rs16870897	rs1535833	chr6:93769380	0.04367934	0.80709492	EPHA7	ENSG00000135333.13	Lung	C=0.722	T=0.278	-0.44499	2.25E-17
rs16870897	rs9363030	chr6:93806040	0.04397051	0.80778141	EPHA7	ENSG00000135333.13	Skin - Sun Exposed (Lower leg)	G=0.721	A=0.279	-0.281476	2.71E-17
rs16870897	rs9353982	chr6:93802038	0.0454394	0.81114188	EPHA7	ENSG00000135333.13	Skin - Sun Exposed (Lower leg)	G=0.716	A=0.284	-0.276454	4.84E-17
rs16870897	rs1570631	chr6:93811876	0.04455544	0.80913985	EPHA7	ENSG00000135333.13	Skin - Sun Exposed (Lower leg)	T=0.719	C=0.281	-0.279518	5.05E-17
rs16870897	rs58205228	chr6:93778613	0.0454394	0.81114188	EPHA7	ENSG00000135333.13	Skin - Sun Exposed (Lower leg)	A=0.716	G=0.284	-0.274662	6.63E-17
rs16870897	rs9345323	chr6:93797607	0.0454394	0.81114188	EPHA7	ENSG00000135333.13	Skin - Sun Exposed (Lower leg)	C=0.716	T=0.284	-0.274662	6.63E-17
rs16870897	rs9342350	chr6:93798264	0.0454394	0.81114188	EPHA7	ENSG00000135333.13	Skin - Sun Exposed (Lower leg)	A=0.716	G=0.284	-0.274662	6.63E-17
rs16870897	rs9363029	chr6:93798520	0.0454394	0.81114188	EPHA7	ENSG00000135333.13	Skin - Sun Exposed (Lower leg)	T=0.716	G=0.284	-0.274662	6.63E-17
rs16870897	rs9345324	chr6:93803037	0.0454394	0.81114188	EPHA7	ENSG00000135333.13	Skin - Sun Exposed (Lower leg)	C=0.716	A=0.284	-0.274662	6.63E-17
rs16870897	rs9345321	chr6:93789199	0.0454394	0.81114188	EPHA7	ENSG00000135333.13	Skin - Sun Exposed (Lower leg)	T=0.716	G=0.284	-0.273298	8.03E-17
rs16870897	rs9345322	chr6:93790565	0.0454394	0.81114188	EPHA7	ENSG00000135333.13	Skin - Sun Exposed (Lower leg)	A=0.716	G=0.284	-0.273298	8.03E-17
rs16870897	rs1535833	chr6:93769380	0.04367934	0.80709492	EPHA7	ENSG00000135333.13	Skin - Sun Exposed (Lower leg)	C=0.722	T=0.278	-0.274816	9.27E-17
rs16870897	rs77898219	chr6:93824234	0.04367934	0.80709492	EPHA7	ENSG00000135333.13	Skin - Sun Exposed (Lower leg)	C=0.722	-=0.278	-0.274384	1.06E-16
rs16870897	rs9363030	chr6:93806040	0.04397051	0.80778141	EPHA7	ENSG00000135333.13	Skin - Not Sun Exposed (Suprapubic)	G=0.721	A=0.279	-0.26209	8.68E-15
rs16870897	rs77898219	chr6:93824234	0.04367934	0.80709492	EPHA7	ENSG00000135333.13	Skin - Not Sun Exposed (Suprapubic)	C=0.722	-=0.278	-0.258658	1.10E-14
rs16870897	rs1570631	chr6:93811876	0.04455544	0.80913985	EPHA7	ENSG00000135333.13	Skin - Not Sun Exposed (Suprapubic)	T=0.719	C=0.281	-0.259435	1.84E-14
rs16870897	rs1535833	chr6:93769380	0.04367934	0.80709492	EPHA7	ENSG00000135333.13	Skin - Not Sun Exposed (Suprapubic)	C=0.722	T=0.278	-0.258257	2.03E-14
rs16870897	rs9342350	chr6:93798264	0.0454394	0.81114188	EPHA7	ENSG00000135333.13	Skin - Not Sun Exposed (Suprapubic)	A=0.716	G=0.284	-0.256364	2.36E-14
rs16870897	rs9363029	chr6:93798520	0.0454394	0.81114188	EPHA7	ENSG00000135333.13	Skin - Not Sun Exposed (Suprapubic)	T=0.716	G=0.284	-0.256364	2.36E-14
rs16870897	rs9353982	chr6:93802038	0.0454394	0.81114188	EPHA7	ENSG00000135333.13	Skin - Not Sun Exposed (Suprapubic)	G=0.716	A=0.284	-0.256364	2.36E-14
rs16870897	rs9345324	chr6:93803037	0.0454394	0.81114188	EPHA7	ENSG00000135333.13	Skin - Not Sun Exposed (Suprapubic)	C=0.716	A=0.284	-0.256364	2.36E-14
rs16870897	rs9345323	chr6:93797607	0.0454394	0.81114188	EPHA7	ENSG00000135333.13	Skin - Not Sun Exposed (Suprapubic)	C=0.716	T=0.284	-0.253851	3.29E-14
rs16870897	rs58205228	chr6:93778613	0.0454394	0.81114188	EPHA7	ENSG00000135333.13	Skin - Not Sun Exposed (Suprapubic)	A=0.716	G=0.284	-0.252522	5.58E-14
rs16870897	rs9345321	chr6:93789199	0.0454394	0.81114188	EPHA7	ENSG00000135333.13	Skin - Not Sun Exposed (Suprapubic)	T=0.716	G=0.284	-0.252522	5.58E-14
rs16870897	rs9345322	chr6:93790565	0.0454394	0.81114188	EPHA7	ENSG00000135333.13	Skin - Not Sun Exposed (Suprapubic)	A=0.716	G=0.284	-0.252522	5.58E-14
rs16870897	rs9353982	chr6:93802038	0.0454394	0.81114188	EPHA7	ENSG00000135333.13	Breast - Mammary Tissue	G=0.716	A=0.284	-0.435156	5.93E-13

rs16870897	rs58205228	chr6:93778613	0.0454394	0.81114188	EPHA7	ENSG00000135333.13	Breast - Mammary Tissue	A=0.716	G=0.284	-0.431238	6.79E-13
rs16870897	rs9345323	chr6:93797607	0.0454394	0.81114188	EPHA7	ENSG00000135333.13	Breast - Mammary Tissue	C=0.716	T=0.284	-0.431238	6.79E-13
rs16870897	rs9342350	chr6:93798264	0.0454394	0.81114188	EPHA7	ENSG00000135333.13	Breast - Mammary Tissue	A=0.716	G=0.284	-0.431238	6.79E-13
rs16870897	rs9363029	chr6:93798520	0.0454394	0.81114188	EPHA7	ENSG00000135333.13	Breast - Mammary Tissue	T=0.716	G=0.284	-0.431238	6.79E-13
rs16870897	rs9345324	chr6:93803037	0.0454394	0.81114188	EPHA7	ENSG00000135333.13	Breast - Mammary Tissue	C=0.716	A=0.284	-0.431238	6.79E-13
rs16870897	rs9363030	chr6:93806040	0.04397051	0.80778141	EPHA7	ENSG00000135333.13	Breast - Mammary Tissue	G=0.721	A=0.279	-0.42573	1.72E-12
rs16870897	rs1570631	chr6:93811876	0.04455544	0.80913985	EPHA7	ENSG00000135333.13	Breast - Mammary Tissue	T=0.719	C=0.281	-0.42573	1.72E-12
rs16870897	rs1535833	chr6:93769380	0.04367934	0.80709492	EPHA7	ENSG00000135333.13	Breast - Mammary Tissue	C=0.722	T=0.278	-0.427079	1.88E-12
rs16870897	rs9345321	chr6:93789199	0.0454394	0.81114188	EPHA7	ENSG00000135333.13	Breast - Mammary Tissue	T=0.716	G=0.284	-0.422298	1.97E-12
rs16870897	rs9345322	chr6:93790565	0.0454394	0.81114188	EPHA7	ENSG00000135333.13	Breast - Mammary Tissue	A=0.716	G=0.284	-0.422298	1.97E-12
rs16870897	rs9363030	chr6:93806040	0.04397051	0.80778141	EPHA7	ENSG00000135333.13	Nerve - Tibial	G=0.721	A=0.279	-0.360092	2.81E-12
rs16870897	rs9363029	chr6:93798520	0.0454394	0.81114188	EPHA7	ENSG00000135333.13	Nerve - Tibial	T=0.716	G=0.284	-0.358549	2.86E-12
rs16870897	rs9342350	chr6:93798264	0.0454394	0.81114188	EPHA7	ENSG00000135333.13	Nerve - Tibial	A=0.716	G=0.284	-0.358336	3.08E-12
rs16870897	rs9345324	chr6:93803037	0.0454394	0.81114188	EPHA7	ENSG00000135333.13	Nerve - Tibial	C=0.716	A=0.284	-0.358336	3.08E-12
rs16870897	rs9345323	chr6:93797607	0.0454394	0.81114188	EPHA7	ENSG00000135333.13	Nerve - Tibial	C=0.716	T=0.284	-0.352327	5.27E-12
rs16870897	rs77898219	chr6:93824234	0.04367934	0.80709492	EPHA7	ENSG00000135333.13	Breast - Mammary Tissue	C=0.722	-=0.278	-0.410352	5.75E-12
rs16870897	rs58205228	chr6:93778613	0.0454394	0.81114188	EPHA7	ENSG00000135333.13	Nerve - Tibial	A=0.716	G=0.284	-0.353209	6.01E-12
rs16870897	rs9353982	chr6:93802038	0.0454394	0.81114188	EPHA7	ENSG00000135333.13	Nerve - Tibial	G=0.716	A=0.284	-0.352973	7.65E-12
rs16870897	rs1570631	chr6:93811876	0.04455544	0.80913985	EPHA7	ENSG00000135333.13	Nerve - Tibial	T=0.719	C=0.281	-0.354962	8.15E-12
rs16870897	rs77898219	chr6:93824234	0.04367934	0.80709492	EPHA7	ENSG00000135333.13	Nerve - Tibial	C=0.722	-=0.278	-0.348397	9.13E-12
rs16870897	rs1535833	chr6:93769380	0.04367934	0.80709492	EPHA7	ENSG00000135333.13	Nerve - Tibial	C=0.722	T=0.278	-0.34822	1.38E-11
rs16870897	rs9345321	chr6:93789199	0.0454394	0.81114188	EPHA7	ENSG00000135333.13	Nerve - Tibial	T=0.716	G=0.284	-0.347757	1.48E-11
rs16870897	rs9345322	chr6:93790565	0.0454394	0.81114188	EPHA7	ENSG00000135333.13	Nerve - Tibial	A=0.716	G=0.284	-0.347757	1.48E-11
rs16870897	rs117365486	chr6:93820610	0.00571211	1	EPHA7	ENSG00000135333.13	Adipose - Subcutaneous	C=0.968	T=0.032	-0.679264	8.33E-10
rs16870897	rs148086058	chr6:93820887	0.00571211	1	EPHA7	ENSG00000135333.13	Adipose - Subcutaneous	C=0.968	A=0.032	-0.679264	8.33E-10
rs16870897	rs189136709	chr6:93783798	0.00086844	1	EPHA7	ENSG00000135333.13	Brain - Anterior cingulate cortex (BA24)	G=0.995	A=0.005	1.35876	1.60E-08
rs16870897	rs1386276	chr6:94082749	0.00408229	0.80709492	EPHA7	ENSG00000135333.13	Heart - Left Ventricle	A=0.965	G=0.035	-0.463277	1.18E-07
rs16870897	rs79718749	chr6:94098832	0.00408229	0.80709492	EPHA7	ENSG00000135333.13	Heart - Left Ventricle	T=0.965	C=0.035	-0.463277	1.18E-07
rs16870897	rs12661215	chr6:94099119	0.00408229	0.80709492	EPHA7	ENSG00000135333.13	Heart - Left Ventricle	C=0.965	T=0.035	-0.463277	1.18E-07
rs16870897	rs16871305	chr6:94111173	0.00408229	0.80709492	EPHA7	ENSG00000135333.13	Heart - Left Ventricle	A=0.965	T=0.035	-0.463277	1.18E-07
rs16870897	rs80300952	chr6:94115209	0.00408229	0.80709492	EPHA7	ENSG00000135333.13	Heart - Left Ventricle	T=0.965	C=0.035	-0.463277	1.18E-07
rs16870897	rs117376030	chr6:94203683	0.00461267	1	EPHA7	ENSG00000135333.13	Esophagus - Gastroesophageal Junction	T=0.974	A=0.026	-0.384156	2.51E-06
rs16870897	rs72928597	chr6:93839843	0.0152247	1	EPHA7	ENSG00000135333.13	Lung	A=0.919	G=0.081	0.42955	4.55E-06
rs16870897	rs16870853	chr6:93841320	0.0152247	1	EPHA7	ENSG00000135333.13	Lung	T=0.919	C=0.081	0.42955	4.55E-06
rs16870897	rs1930933	chr6:93842544	0.07093782	0.97756918	EPHA7	ENSG00000135333.13	Minor Salivary Gland	C=0.299	T=0.701	0.246092	4.56E-06
rs16870897	rs142723671	chr6:94076414	0.00388717	1	EPHA7	ENSG00000135333.13	Heart - Left Ventricle	G=0.978	C=0.022	-0.495017	4.73E-06
rs16870897	rs147330775	chr6:93915683	0.00104317	1	EPHA7	ENSG00000135333.13	Brain - Anterior cingulate cortex (BA24)	C=0.994	T=0.006	0.808887	6.34E-06
rs16870897	rs16870793	chr6:93828599	0.01481674	1	EPHA7	ENSG00000135333.13	Lung	G=0.921	A=0.079	0.419095	6.62E-06
rs16870897	rs72928592	chr6:93837959	0.0150205	1	EPHA7	ENSG00000135333.13	Lung	T=0.92	C=0.08	0.419095	6.62E-06
rs16870897	rs1575540	chr6:93836577	0.01063715	1	EPHA7	ENSG00000135333.13	Esophagus - Muscularis	G=0.942	A=0.058	0.272463	7.68E-06
rs16870897	rs544639807	chr6:93883657	0.00139369	1	EPHA7	ENSG00000135333.13	Minor Salivary Gland	G=0.992	A=0.008	-1.34812	8.25E-06
rs16870897	rs36114673	chr6:93847056	0.01141892	1	EPHA7	ENSG00000135333.13	Esophagus - Muscularis	A=0.938	T=0.062	0.268662	8.26E-06
rs16870897	rs7776099	chr6:93913423	0.02648532	0.87798173	EPHA7	ENSG00000135333.13	Testis	C=0.835	A=0.165	0.3716	8.26E-06
rs16870897	rs7758242	chr6:93913435	0.02648532	0.87798173	EPHA7	ENSG00000135333.13	Testis	T=0.835	A=0.165	0.3716	8.26E-06
rs16870897	rs66765303	chr6:93913692	0.02648532	0.87798173	EPHA7	ENSG00000135333.13	Testis	A=0.835	G=0.165	0.3716	8.26E-06
rs16870897	rs66522431	chr6:93913825	0.02648532	0.87798173	EPHA7	ENSG00000135333.13	Testis	G=0.835	A=0.165	0.3716	8.26E-06
rs16870897	rs62414181	chr6:93913909	0.02648532	0.87798173	EPHA7	ENSG00000135333.13	Testis	A=0.835	G=0.165	0.3716	8.26E-06
rs16870897	rs62414182	chr6:93914022	0.02648532	0.87798173	EPHA7	ENSG00000135333.13	Testis	A=0.835	G=0.165	0.3716	8.26E-06
rs16870897	rs113888577	chr6:93698093	0.00209894	1	EPHA7	ENSG00000135333.13	Brain - Anterior cingulate cortex (BA24)	C=0.988	T=0.012	0.66365	8.36E-06
rs16870897	rs16870780	chr6:93826505	0.0150205	1	EPHA7	ENSG00000135333.13	Lung	T=0.92	A=0.08	0.385133	9.62E-06
rs16870897	rs1590384	chr6:93837434	0.07093782	0.97756918	EPHA7	ENSG00000135333.13	Minor Salivary Gland	C=0.701	G=0.299	-0.24131	1.00E-05

rs16870897	rs1924474	chr6:93837991	0.07093782	0.97756918	EPHA7	ENSG00000135333.13	Minor Salivary Gland	C=0.701	T=0.299	-0.24131	1.00E-05
rs16870897	rs10944652	chr6:93838348	0.07093782	0.97756918	EPHA7	ENSG00000135333.13	Minor Salivary Gland	C=0.701	G=0.299	-0.24131	1.00E-05
rs16870897	rs12204186	chr6:93838665	0.07093782	0.97756918	EPHA7	ENSG00000135333.13	Minor Salivary Gland	C=0.701	T=0.299	-0.24131	1.00E-05
rs16870897	rs633279	chr6:93840705	0.07093782	0.97756918	EPHA7	ENSG00000135333.13	Minor Salivary Gland	A=0.299	T=0.701	0.24131	1.00E-05
rs16870897	rs634060	chr6:93840849	0.07093782	0.97756918	EPHA7	ENSG00000135333.13	Minor Salivary Gland	A=0.299	G=0.701	0.24131	1.00E-05
rs16870897	rs568957	chr6:93840855	0.07093782	0.97756918	EPHA7	ENSG00000135333.13	Minor Salivary Gland	G=0.299	T=0.701	0.24131	1.00E-05
rs16870897	rs34544163	chr6:93841565	0.07093782	0.97756918	EPHA7	ENSG00000135333.13	Minor Salivary Gland	-=0.299	A=0.701	0.24131	1.00E-05
rs16870897	rs562379	chr6:93841592	0.07093782	0.97756918	EPHA7	ENSG00000135333.13	Minor Salivary Gland	G=0.299	A=0.701	0.24131	1.00E-05
rs16870897	rs560731	chr6:93841734	0.07093782	0.97756918	EPHA7	ENSG00000135333.13	Minor Salivary Gland	A=0.299	G=0.701	0.24131	1.00E-05
rs16870897	rs650642	chr6:93842232	0.07093782	0.97756918	EPHA7	ENSG00000135333.13	Minor Salivary Gland	G=0.299	T=0.701	0.24131	1.00E-05
rs16870897	rs650747	chr6:93842298	0.07093782	0.97756918	EPHA7	ENSG00000135333.13	Minor Salivary Gland	T=0.299	C=0.701	0.24131	1.00E-05
rs16870897	rs1930934	chr6:93842533	0.07093782	0.97756918	EPHA7	ENSG00000135333.13	Minor Salivary Gland	A=0.299	G=0.701	0.24131	1.00E-05
rs16870897	rs9353987	chr6:93843588	0.07093782	0.97756918	EPHA7	ENSG00000135333.13	Minor Salivary Gland	A=0.299	G=0.701	0.24131	1.00E-05
rs16870897	rs9363034	chr6:93844136	0.07093782	0.97756918	EPHA7	ENSG00000135333.13	Minor Salivary Gland	G=0.299	A=0.701	0.24131	1.00E-05
rs16870897	rs9353989	chr6:93844383	0.07093782	0.97756918	EPHA7	ENSG00000135333.13	Minor Salivary Gland	G=0.299	T=0.701	0.24131	1.00E-05
rs16870897	rs76282783	chr6:93576050	0.00533527	0.83924577	EPHA7	ENSG00000135333.13	Lung	C=0.958	A=0.042	0.702179	1.18E-05
rs16870897	rs199888991	chr6:93784078	0.00086844	1	EPHA7	ENSG00000135333.13	Adipose - Subcutaneous	-=0.995	T=0.005	-0.796053	1.44E-05
rs16870897	rs111429740	chr6:93838453	0.0152247	1	EPHA7	ENSG00000135333.13	Lung	G=0.919	A=0.081	0.383525	1.55E-05
rs16870897	rs16870789	chr6:93827963	0.0150205	1	EPHA7	ENSG00000135333.13	Lung	C=0.92	T=0.08	0.374368	1.81E-05
rs16870897	rs145810085	chr6:93833819	0.00139369	1	EPHA7	ENSG00000135333.13	Adipose - Subcutaneous	G=0.992	A=0.008	-0.735109	1.88E-05
rs16870897	rs7774823	chr6:93830545	0.07024556	0.97741914	EPHA7	ENSG00000135333.13	Minor Salivary Gland	G=0.703	A=0.297	-0.232136	1.89E-05
rs16870897	rs1951907	chr6:93831192	0.07024556	0.97741914	EPHA7	ENSG00000135333.13	Minor Salivary Gland	T=0.703	C=0.297	-0.232136	1.89E-05
rs16870897	rs35306488	chr6:93832227	0.0692145	0.97719028	EPHA7	ENSG00000135333.13	Minor Salivary Gland	-=0.706	T=0.294	-0.232136	1.89E-05
rs16870897	rs10944648	chr6:93832280	0.07024556	0.97741914	EPHA7	ENSG00000135333.13	Minor Salivary Gland	A=0.703	G=0.297	-0.232136	1.89E-05
rs16870897	rs7751375	chr6:93832923	0.0705912	0.97749441	EPHA7	ENSG00000135333.13	Minor Salivary Gland	C=0.702	A=0.298	-0.232136	1.89E-05
rs16870897	rs7757292	chr6:93834165	0.07024556	0.97741914	EPHA7	ENSG00000135333.13	Minor Salivary Gland	C=0.703	A=0.297	-0.232136	1.89E-05
rs16870897	rs16870850	chr6:93841079	0.01604592	1	EPHA7	ENSG00000135333.13	Lung	T=0.916	C=0.084	0.361979	2.02E-05
rs16870897	rs12528846	chr6:93828842	0.0150205	1	EPHA7	ENSG00000135333.13	Lung	A=0.92	G=0.08	0.377147	2.03E-05
rs16870897	rs12527296	chr6:93829143	0.0150205	1	EPHA7	ENSG00000135333.13	Lung	T=0.92	C=0.08	0.377147	2.03E-05
rs16870897	rs72928575	chr6:93829399	0.0152247	1	EPHA7	ENSG00000135333.13	Lung	G=0.919	T=0.081	0.377147	2.03E-05
rs16870897	rs72928579	chr6:93829658	0.0150205	1	EPHA7	ENSG00000135333.13	Lung	G=0.92	A=0.08	0.377147	2.03E-05
rs16870897	rs77007574	chr6:93829960	0.0150205	1	EPHA7	ENSG00000135333.13	Lung	T=0.92	C=0.08	0.377147	2.03E-05
rs16870897	rs72928582	chr6:93830015	0.0150205	1	EPHA7	ENSG00000135333.13	Lung	G=0.92	A=0.08	0.377147	2.03E-05
rs16870897	rs16870801	chr6:93830951	0.0150205	1	EPHA7	ENSG00000135333.13	Lung	T=0.92	C=0.08	0.377147	2.03E-05
rs16870897	rs16870805	chr6:93834075	0.0150205	1	EPHA7	ENSG00000135333.13	Lung	G=0.92	T=0.08	0.377147	2.03E-05
rs16870897	rs150304721	chr6:93835801	0.01542934	1	EPHA7	ENSG00000135333.13	Lung	A=0.918	-=0.082	0.361316	2.04E-05
rs16870897	rs16870855	chr6:93841440	0.0152247	1	EPHA7	ENSG00000135333.13	Lung	C=0.919	T=0.081	0.4288	2.14E-05
rs16870897	rs16880183	chr6:93889701	0.03137644	0.95908074	EPHA7	ENSG00000135333.13	Testis	A=0.836	G=0.164	0.353058	2.24E-05
rs16870897	rs7772178	chr6:93849608	0.02310333	1	EPHA7	ENSG00000135333.13	Testis	C=0.883	T=0.117	0.425527	2.47E-05
rs16870897	rs6923008	chr6:93887045	0.03137644	0.95908074	EPHA7	ENSG00000135333.13	Testis	G=0.836	T=0.164	0.351036	2.54E-05
rs16870897	rs60592958	chr6:93887933	0.03137644	0.95908074	EPHA7	ENSG00000135333.13	Testis	C=0.836	T=0.164	0.351036	2.54E-05
rs16870897	rs73758259	chr6:93828724	0.01563442	1	EPHA7	ENSG00000135333.13	Lung	G=0.917	T=0.083	0.354112	2.72E-05
rs16870897	rs16870796	chr6:93828781	0.0150205	1	EPHA7	ENSG00000135333.13	Lung	T=0.92	G=0.08	0.369069	2.76E-05
rs16870897	rs72928577	chr6:93829400	0.0152247	1	EPHA7	ENSG00000135333.13	Lung	C=0.919	T=0.081	0.369069	2.76E-05
rs16870897	rs117717826	chr6:94036945	0.00443074	1	EPHA7	ENSG00000135333.13	Heart - Left Ventricle	T=0.975	A=0.025	-0.389622	2.96E-05
rs16870897	rs56378615	chr6:93900091	0.02476734	0.90876111	EPHA7	ENSG00000135333.13	Testis	C=0.853	T=0.147	0.358985	3.02E-05
rs16870897	rs71558449	chr6:93828589	0.00589667	1	EPHA7	ENSG00000135333.13	Esophagus - Muscularis	G=0.967	A=0.033	0.272458	3.14E-05
rs16870897	rs6922405	chr6:93842847	0.0152247	1	EPHA7	ENSG00000135333.13	Lung	T=0.919	C=0.081	0.370721	3.25E-05
rs16870897	rs72930416	chr6:93844150	0.0152247	1	EPHA7	ENSG00000135333.13	Lung	T=0.919	C=0.081	0.370721	3.25E-05
rs16870897	rs16870846	chr6:93840909	0.0152247	1	EPHA7	ENSG00000135333.13	Lung	C=0.919	G=0.081	0.371767	3.40E-05
rs16870897	rs1319460	chr6:93846637	0.05447383	1	EPHA7	ENSG00000135333.13	Minor Salivary Gland	C=0.239	T=0.761	0.230876	3.54E-05

rs16870897	rs538751	chr6:93848400	0.05447383	1	EPHA7	ENSG00000135333.13	Minor Salivary Gland	A=0.239	C=0.761	0.230876	3.54E-05
rs16870897	rs11422146	chr6:93849652	0.05417613	1	EPHA7	ENSG00000135333.13	Minor Salivary Gland	-=0.238	T=0.762	0.230876	3.54E-05
rs16870897	rs473900	chr6:93931037	0.03724721	0.85258345	EPHA7	ENSG00000135333.13	Adipose - Subcutaneous	T=0.228	C=0.772	0.223628	3.56E-05
rs16870897	rs369196	chr6:93932634	0.03724721	0.85258345	EPHA7	ENSG00000135333.13	Adipose - Subcutaneous	C=0.228	T=0.772	0.223628	3.56E-05
rs16870897	rs1319460	chr6:93846637	0.05447383	1	EPHA7	ENSG00000135333.13	Testis	C=0.239	T=0.761	-0.330556	3.66E-05
rs16870897	rs538751	chr6:93848400	0.05447383	1	EPHA7	ENSG00000135333.13	Testis	A=0.239	C=0.761	-0.330556	3.66E-05
rs16870897	rs11422146	chr6:93849652	0.05417613	1	EPHA7	ENSG00000135333.13	Testis	-=0.238	T=0.762	-0.330556	3.66E-05
rs16870897	rs6935219	chr6:93889277	0.03137644	0.95908074	EPHA7	ENSG00000135333.13	Testis	A=0.836	G=0.164	0.33974	4.29E-05
rs16870897	rs140182812	chr6:93804212	0.00139369	1	EPHA7	ENSG00000135333.13	Adipose - Subcutaneous	G=0.992	A=0.008	-0.729088	4.39E-05
rs16870897	rs10686381	chr6:93887552	0.03137644	0.95908074	EPHA7	ENSG00000135333.13	Testis	-=0.836	AAGT=0.164	0.337644	4.84E-05
rs16870897	rs12530331	chr6:93830460	0.0152247	1	EPHA7	ENSG00000135333.13	Lung	G=0.919	C=0.081	0.35653	5.33E-05
rs16870897	rs74468395	chr6:94017659	0.00589667	1	EPHA7	ENSG00000135333.13	Heart - Left Ventricle	C=0.967	T=0.033	-0.353	6.19E-05
rs16870897	rs117256127	chr6:93852714	0.00424918	1	EPHA7	ENSG00000135333.13	Adipose - Subcutaneous	T=0.976	C=0.024	-0.674139	6.42E-05
rs16870897	rs117425371	chr6:93852717	0.00424918	1	EPHA7	ENSG00000135333.13	Adipose - Subcutaneous	A=0.976	G=0.024	-0.674139	6.42E-05
rs16870897	rs145519743	chr6:93884032	0.00443074	1	EPHA7	ENSG00000135333.13	Adipose - Subcutaneous	G=0.975	A=0.025	-0.674139	6.42E-05
rs16870897	rs2780662	chr6:93920660	0.05119093	0.85884994	EPHA7	ENSG00000135333.13	Testis	T=0.285	C=0.715	-0.29987	6.44E-05
rs16870897	rs9351349	chr6:93832104	0.02984058	0.88747204	EPHA7	ENSG00000135333.13	Adipose - Subcutaneous	G=0.179	A=0.821	0.228214	6.48E-05
rs16870897	rs147330775	chr6:93915683	0.00104317	1	EPHA7	ENSG00000135333.13	Skin - Sun Exposed (Lower leg)	C=0.994	T=0.006	-0.47159	6.56E-05
rs16870897	rs117109139	chr6:93861398	0.00682517	1	EPHA7	ENSG00000135333.13	Esophagus - Muscularis	C=0.962	G=0.038	0.257424	7.15E-05
rs16870897	rs650711	chr6:93842280	0.02984058	0.88747204	EPHA7	ENSG00000135333.13	Adipose - Subcutaneous	T=0.821	G=0.179	-0.226702	7.44E-05
rs16870897	rs768382	chr6:93826168	0.02959694	0.88684339	EPHA7	ENSG00000135333.13	Adipose - Subcutaneous	T=0.178	C=0.822	0.223191	7.87E-05
rs16870897	rs412388	chr6:93916878	0.05119093	0.85884994	EPHA7	ENSG00000135333.13	Testis	G=0.285	A=0.715	-0.296773	8.64E-05
rs16870897	rs1324110	chr6:93913200	0.1551874	0.83299737	EPHA7	ENSG00000135333.13	Heart - Atrial Appendage	G=0.563	C=0.437	0.0819979	9.78E-05
rs16870897	rs138022068	chr6:94069111	0.00643297	0.85934004	EPHA7	ENSG00000135333.13	Heart - Left Ventricle	C=0.952	-=0.048	-0.300501	0.00011172
rs16870897	rs11970583	chr6:93895756	0.04367934	0.80709492	EPHA7	ENSG00000135333.13	Esophagus - Muscularis	C=0.722	T=0.278	0.0990772	0.00011475
rs16870897	rs2065582	chr6:93896309	0.04367934	0.80709492	EPHA7	ENSG00000135333.13	Esophagus - Muscularis	G=0.722	T=0.278	0.0990772	0.00011475
rs16870897	rs2147225	chr6:93898127	0.04367934	0.80709492	EPHA7	ENSG00000135333.13	Esophagus - Muscularis	T=0.722	A=0.278	0.0990772	0.00011475
rs16870897	rs13205265	chr6:93898896	0.04367934	0.80709492	EPHA7	ENSG00000135333.13	Esophagus - Muscularis	C=0.722	T=0.278	0.0990772	0.00011475
rs16870897	rs35480101	chr6:93899371	0.04338904	0.8064035	EPHA7	ENSG00000135333.13	Esophagus - Muscularis	G=0.723	T=0.277	0.0990772	0.00011475
rs16870897	rs145046790	chr6:93899520	0.04367934	0.80709492	EPHA7	ENSG00000135333.13	Esophagus - Muscularis	AT=0.722	-=0.278	0.0990772	0.00011475
rs16870897	rs192604147	chr6:94240629	0.00156947	1	EPHA7	ENSG00000135333.13	Heart - Left Ventricle	A=0.991	G=0.009	-0.514265	0.00011598
rs16870897	rs9345321	chr6:93789199	0.0454394	0.81114188	EPHA7	ENSG00000135333.13	Esophagus - Mucosa	T=0.716	G=0.284	-0.209017	0.00012891
rs16870897	rs9345322	chr6:93790565	0.0454394	0.81114188	EPHA7	ENSG00000135333.13	Esophagus - Mucosa	A=0.716	G=0.284	-0.209017	0.00012891
rs16870897	rs9353982	chr6:93802038	0.0454394	0.81114188	EPHA7	ENSG00000135333.13	Esophagus - Mucosa	G=0.716	A=0.284	-0.209017	0.00012891
rs16870897	rs16880179	chr6:93889506	0.02684937	0.95375563	EPHA7	ENSG00000135333.13	Testis	G=0.855	A=0.145	0.335079	0.00013631
rs16870897	rs59810007	chr6:93891733	0.02684937	0.95375563	EPHA7	ENSG00000135333.13	Testis	T=0.855	C=0.145	0.335079	0.00013631
rs16870897	rs6901961	chr6:93892700	0.02684937	0.95375563	EPHA7	ENSG00000135333.13	Testis	T=0.855	A=0.145	0.335079	0.00013631
rs16870897	rs60804851	chr6:93894513	0.02684937	0.95375563	EPHA7	ENSG00000135333.13	Testis	C=0.855	G=0.145	0.335079	0.00013631
rs16870897	rs6901870	chr6:93895903	0.02684937	0.95375563	EPHA7	ENSG00000135333.13	Testis	A=0.855	C=0.145	0.335079	0.00013631
rs16870897	rs6901827	chr6:93895828	0.04367934	0.80709492	EPHA7	ENSG00000135333.13	Esophagus - Muscularis	A=0.722	G=0.278	0.0976714	0.00014821
rs16870897	rs2181805	chr6:93898026	0.04367934	0.80709492	EPHA7	ENSG00000135333.13	Esophagus - Muscularis	T=0.722	C=0.278	0.0976714	0.00014821
rs16870897	rs2631562	chr6:93920850	0.05088319	0.85835641	EPHA7	ENSG00000135333.13	Testis	T=0.284	C=0.716	-0.28302	0.00014886
rs16870897	rs143801031	chr6:93750441	0.00298853	1	EPHA7	ENSG00000135333.13	Adipose - Subcutaneous	T=0.983	C=0.017	-0.536087	0.00015072
rs16870897	rs58205228	chr6:93778613	0.0454394	0.81114188	EPHA7	ENSG00000135333.13	Esophagus - Mucosa	A=0.716	G=0.284	-0.206708	0.00015126
rs16870897	rs9342350	chr6:93798264	0.0454394	0.81114188	EPHA7	ENSG00000135333.13	Esophagus - Mucosa	A=0.716	G=0.284	-0.206708	0.00015126
rs16870897	rs9363029	chr6:93798520	0.0454394	0.81114188	EPHA7	ENSG00000135333.13	Esophagus - Mucosa	T=0.716	G=0.284	-0.206708	0.00015126
rs16870897	rs9345324	chr6:93803037	0.0454394	0.81114188	EPHA7	ENSG00000135333.13	Esophagus - Mucosa	C=0.716	A=0.284	-0.206708	0.00015126
rs16870897	rs6922792	chr6:93887222	0.02684937	0.95375563	EPHA7	ENSG00000135333.13	Testis	A=0.855	G=0.145	0.333048	0.00015245
rs16870897	rs117365486	chr6:93820610	0.00571211	1	EPHA7	ENSG00000135333.13	Nerve - Tibial	C=0.968	T=0.032	-0.404506	0.000162
rs16870897	rs148086058	chr6:93820887	0.00571211	1	EPHA7	ENSG00000135333.13	Nerve - Tibial	C=0.968	A=0.032	-0.404506	0.000162
rs16870897	rs11966965	chr6:93890049	0.04338904	0.8064035	EPHA7	ENSG00000135333.13	Esophagus - Muscularis	G=0.723	T=0.277	0.096593	0.00016937

rs16870897	rs35373649	chr6:93891073	0.04338904	0.8064035	EPHA7	ENSG00000135333.13	Esophagus - Muscularis	A=0.723	G=0.277	0.096593	0.00016937
rs16870897	rs6921600	chr6:93892299	0.04367934	0.80709492	EPHA7	ENSG00000135333.13	Esophagus - Muscularis	G=0.722	T=0.278	0.096593	0.00016937
rs16870897	rs6901416	chr6:93892412	0.04367934	0.80709492	EPHA7	ENSG00000135333.13	Esophagus - Muscularis	T=0.722	G=0.278	0.096593	0.00016937
rs16870897	rs9345323	chr6:93797607	0.0454394	0.81114188	EPHA7	ENSG00000135333.13	Esophagus - Mucosa	C=0.716	T=0.284	-0.204207	0.00017144
rs16870897	rs1535833	chr6:93769380	0.04367934	0.80709492	EPHA7	ENSG00000135333.13	Esophagus - Mucosa	C=0.722	T=0.278	-0.205304	0.00017505
rs16870897	rs77898219	chr6:93824234	0.04367934	0.80709492	EPHA7	ENSG00000135333.13	Esophagus - Mucosa	C=0.722	-=0.278	-0.207398	0.00017867
rs16870897	rs9363030	chr6:93806040	0.04397051	0.80778141	EPHA7	ENSG00000135333.13	Esophagus - Mucosa	G=0.721	A=0.279	-0.206095	0.00019318
rs16870897	rs1953145	chr6:93890592	0.04338904	0.8064035	EPHA7	ENSG00000135333.13	Esophagus - Muscularis	G=0.723	A=0.277	0.0952131	0.00021698
rs16870897	rs12110542	chr6:93894263	0.04367934	0.80709492	EPHA7	ENSG00000135333.13	Esophagus - Muscularis	T=0.722	G=0.278	0.0952131	0.00021698
rs16870897	rs1953146	chr6:93897891	0.04367934	0.80709492	EPHA7	ENSG00000135333.13	Esophagus - Muscularis	C=0.722	G=0.278	0.0952131	0.00021698
rs16870897	rs182357880	chr6:93879803	0.00121825	1	EPHA7	ENSG00000135333.13	Skin - Sun Exposed (Lower leg)	T=0.993	C=0.007	-0.395624	0.00022421
rs16870897	rs144384339	chr6:93897863	0.00370671	1	EPHA7	ENSG00000135333.13	Skin - Sun Exposed (Lower leg)	G=0.979	A=0.021	-0.418007	0.00029351
rs16870964	rs117365486	chr6:93820610	0.00253266	1	EPHA7	ENSG00000135333.13	Adipose - Subcutaneous	C=0.968	T=0.032	-0.679264	8.33E-10
rs16870964	rs148086058	chr6:93820887	0.00253266	1	EPHA7	ENSG00000135333.13	Adipose - Subcutaneous	C=0.968	A=0.032	-0.679264	8.33E-10
rs16870964	rs11962709	chr6:93891195	0.00985203	1	EPHA7	ENSG00000135333.13	Esophagus - Muscularis	A=0.887	G=0.113	0.219885	7.45E-09
rs16870964	rs71540101	chr6:93892043	0.00985203	1	EPHA7	ENSG00000135333.13	Esophagus - Muscularis	AGGTATATC=0.887	-=0.113	0.219885	7.45E-09
rs16870964	rs35962225	chr6:93915766	0.01014541	1	EPHA7	ENSG00000135333.13	Esophagus - Muscularis	G=0.884	C=0.116	0.229888	1.18E-08
rs16870964	rs35762480	chr6:93906245	0.0100474	1	EPHA7	ENSG00000135333.13	Esophagus - Muscularis	G=0.885	C=0.115	0.216734	1.27E-08
rs16870964	rs189136709	chr6:93783798	0.00038505	1	EPHA7	ENSG00000135333.13	Brain - Anterior cingulate cortex (BA24)	G=0.995	A=0.005	1.35876	1.60E-08
rs16870964	rs11962003	chr6:93890071	0.01174419	1	EPHA7	ENSG00000135333.13	Esophagus - Muscularis	A=0.868	G=0.132	0.206691	1.76E-08
rs16870964	rs11966984	chr6:93890176	0.01174419	1	EPHA7	ENSG00000135333.13	Esophagus - Muscularis	G=0.868	A=0.132	0.206691	1.76E-08
rs16870964	rs34948845	chr6:93894820	0.01174419	1	EPHA7	ENSG00000135333.13	Esophagus - Muscularis	G=0.868	A=0.132	0.206691	1.76E-08
rs16870964	rs11966926	chr6:93897338	0.00985203	1	EPHA7	ENSG00000135333.13	Esophagus - Muscularis	A=0.887	G=0.113	0.215362	2.61E-08
rs16870964	rs72919018	chr6:93924471	0.0136905	0.80594136	EPHA7	ENSG00000135333.13	Adipose - Subcutaneous	G=0.785	C=0.215	0.32156	2.76E-08
rs16870964	rs12189899	chr6:93931092	0.0136905	0.80594136	EPHA7	ENSG00000135333.13	Adipose - Subcutaneous	C=0.785	T=0.215	0.32156	2.76E-08
rs16870964	rs12199960	chr6:93923853	0.01357942	0.80503876	EPHA7	ENSG00000135333.13	Adipose - Subcutaneous	A=0.786	C=0.214	0.32248	4.27E-08
rs16870964	rs11967483	chr6:93903691	0.0100474	1	EPHA7	ENSG00000135333.13	Esophagus - Muscularis	A=0.885	T=0.115	0.206895	4.64E-08
rs16870964	rs34832688	chr6:93891054	0.00985203	1	EPHA7	ENSG00000135333.13	Esophagus - Muscularis	A=0.887	C=0.113	0.205584	4.66E-08
rs16870964	rs72919020	chr6:93925349	0.0136905	0.80594136	EPHA7	ENSG00000135333.13	Adipose - Subcutaneous	G=0.785	C=0.215	0.32041	4.76E-08
rs16870964	rs12190544	chr6:93925959	0.0136905	0.80594136	EPHA7	ENSG00000135333.13	Adipose - Subcutaneous	G=0.785	A=0.215	0.32041	4.76E-08
rs16870964	rs12206216	chr6:93927822	0.01380189	0.80683564	EPHA7	ENSG00000135333.13	Adipose - Subcutaneous	A=0.784	G=0.216	0.32041	4.76E-08
rs16870964	rs12196073	chr6:93928926	0.0136905	0.80594136	EPHA7	ENSG00000135333.13	Adipose - Subcutaneous	C=0.785	G=0.215	0.32041	4.76E-08
rs16870964	rs12196420	chr6:93929507	0.0136905	0.80594136	EPHA7	ENSG00000135333.13	Adipose - Subcutaneous	C=0.785	T=0.215	0.32041	4.76E-08
rs16870964	rs12201744	chr6:93930642	0.01357942	0.80503876	EPHA7	ENSG00000135333.13	Adipose - Subcutaneous	A=0.786	T=0.214	0.32041	4.76E-08
rs16870964	rs12195545	chr6:93930755	0.01357942	0.80503876	EPHA7	ENSG00000135333.13	Adipose - Subcutaneous	T=0.786	G=0.214	0.32041	4.76E-08
rs16870964	rs143708097	chr6:93931051	0.01357942	0.80503876	EPHA7	ENSG00000135333.13	Adipose - Subcutaneous	-=0.786	CCTA=0.214	0.32041	4.76E-08
rs16870964	rs72919042	chr6:93935578	0.0136905	0.80594136	EPHA7	ENSG00000135333.13	Adipose - Subcutaneous	C=0.785	T=0.215	0.32041	4.76E-08
rs16870964	rs13212701	chr6:93902787	0.0100474	1	EPHA7	ENSG00000135333.13	Esophagus - Muscularis	T=0.885	C=0.115	0.206435	4.99E-08
rs16870964	rs6936693	chr6:93903686	0.0100474	1	EPHA7	ENSG00000135333.13	Esophagus - Muscularis	T=0.885	C=0.115	0.202265	5.80E-08
rs16870964	rs12374628	chr6:93934768	0.0136905	0.80594136	EPHA7	ENSG00000135333.13	Adipose - Subcutaneous	T=0.785	C=0.215	0.313163	6.59E-08
rs16870964	rs388461	chr6:93918959	0.01014541	1	EPHA7	ENSG00000135333.13	Esophagus - Muscularis	A=0.884	G=0.116	0.191263	7.48E-08
rs16870964	rs575590	chr6:93920650	0.0100474	1	EPHA7	ENSG00000135333.13	Esophagus - Muscularis	G=0.885	A=0.115	0.191263	7.48E-08
rs16870964	rs72919047	chr6:93941158	0.0136905	0.80594136	EPHA7	ENSG00000135333.13	Adipose - Subcutaneous	T=0.785	C=0.215	0.313468	9.08E-08
rs16870964	rs12209980	chr6:93943432	0.0136905	0.80594136	EPHA7	ENSG00000135333.13	Adipose - Subcutaneous	T=0.785	G=0.215	0.313468	9.08E-08
rs16870964	rs12200456	chr6:93924735	0.01380189	0.80683564	EPHA7	ENSG00000135333.13	Adipose - Subcutaneous	A=0.784	G=0.216	0.313602	9.70E-08
rs16870964	rs72919045	chr6:93940859	0.0136905	0.80594136	EPHA7	ENSG00000135333.13	Adipose - Subcutaneous	T=0.785	A=0.215	0.315063	9.80E-08
rs16870964	rs6931234	chr6:93927181	0.01380189	0.80683564	EPHA7	ENSG00000135333.13	Adipose - Subcutaneous	T=0.784	C=0.216	0.299008	1.06E-07
rs16870964	rs13202762	chr6:93937080	0.00785232	1	EPHA7	ENSG00000135333.13	Esophagus - Muscularis	T=0.908	G=0.092	0.222914	1.15E-07
rs16870964	rs10693258	chr6:93902052	0.01024365	1	EPHA7	ENSG00000135333.13	Esophagus - Muscularis	-=0.883	TTG=0.117	0.174304	5.19E-07
rs16870964	rs13212701	chr6:93902787	0.0100474	1	EPHA7	ENSG00000135333.13	Esophagus - Mucosa	T=0.885	C=0.115	0.405746	1.63E-06
rs16870964	rs35762480	chr6:93906245	0.0100474	1	EPHA7	ENSG00000135333.13	Esophagus - Mucosa	G=0.885	C=0.115	0.407424	1.79E-06

rs16870964	rs117376030	chr6:94203683	0.00204519	1	EPHA7	ENSG00000135333.13	Esophagus - Gastroesophageal Junction	T=0.974	A=0.026	-0.384156	2.51E-06
rs16870964	rs72928597	chr6:93839843	0.00675039	1	EPHA7	ENSG00000135333.13	Lung	A=0.919	G=0.081	0.42955	4.55E-06
rs16870964	rs16870853	chr6:93841320	0.00675039	1	EPHA7	ENSG00000135333.13	Lung	T=0.919	C=0.081	0.42955	4.55E-06
rs16870964	rs1930933	chr6:93842544	0.02992801	0.95358066	EPHA7	ENSG00000135333.13	Minor Salivary Gland	C=0.299	T=0.701	0.246092	4.56E-06
rs16870964	rs142723671	chr6:94076414	0.00172351	1	EPHA7	ENSG00000135333.13	Heart - Left Ventricle	G=0.978	C=0.022	-0.495017	4.73E-06
rs16870964	rs11962709	chr6:93891195	0.00985203	1	EPHA7	ENSG00000135333.13	Esophagus - Mucosa	A=0.887	G=0.113	0.385284	6.17E-06
rs16870964	rs71540101	chr6:93892043	0.00985203	1	EPHA7	ENSG00000135333.13	Esophagus - Mucosa	AGGTATATC=0.887	-=0.113	0.385284	6.17E-06
rs16870964	rs147330775	chr6:93915683	0.00046253	1	EPHA7	ENSG00000135333.13	Brain - Anterior cingulate cortex (BA24)	C=0.994	T=0.006	0.808887	6.34E-06
rs16870964	rs6928892	chr6:93888220	0.09285406	0.90654032	EPHA7	ENSG00000135333.13	Esophagus - Muscularis	A=0.594	G=0.406	0.107385	6.55E-06
rs16870964	rs16870793	chr6:93828599	0.00656951	1	EPHA7	ENSG00000135333.13	Lung	G=0.921	A=0.079	0.419095	6.62E-06
rs16870964	rs72928592	chr6:93837959	0.00665985	1	EPHA7	ENSG00000135333.13	Lung	T=0.92	C=0.08	0.419095	6.62E-06
rs16870964	rs10693258	chr6:93902052	0.01024365	1	EPHA7	ENSG00000135333.13	Esophagus - Mucosa	-=0.883	TTG=0.117	0.343667	6.63E-06
rs16870964	rs34832688	chr6:93891054	0.00985203	1	EPHA7	ENSG00000135333.13	Esophagus - Mucosa	A=0.887	C=0.113	0.378517	6.87E-06
rs16870964	rs1575540	chr6:93836577	0.00471634	1	EPHA7	ENSG00000135333.13	Esophagus - Muscularis	G=0.942	A=0.058	0.272463	7.68E-06
rs16870964	rs544639807	chr6:93883657	0.00061794	1	EPHA7	ENSG00000135333.13	Minor Salivary Gland	G=0.992	A=0.008	-1.34812	8.25E-06
rs16870964	rs36114673	chr6:93847056	0.00506297	1	EPHA7	ENSG00000135333.13	Esophagus - Muscularis	A=0.938	T=0.062	0.268662	8.26E-06
rs16870964	rs7776099	chr6:93913423	0.01053673	0.83165997	EPHA7	ENSG00000135333.13	Testis	C=0.835	A=0.165	0.3716	8.26E-06
rs16870964	rs7758242	chr6:93913435	0.01053673	0.83165997	EPHA7	ENSG00000135333.13	Testis	T=0.835	A=0.165	0.3716	8.26E-06
rs16870964	rs66765303	chr6:93913692	0.01053673	0.83165997	EPHA7	ENSG00000135333.13	Testis	A=0.835	G=0.165	0.3716	8.26E-06
rs16870964	rs66522431	chr6:93913825	0.01053673	0.83165997	EPHA7	ENSG00000135333.13	Testis	G=0.835	A=0.165	0.3716	8.26E-06
rs16870964	rs62414181	chr6:93913909	0.01053673	0.83165997	EPHA7	ENSG00000135333.13	Testis	A=0.835	G=0.165	0.3716	8.26E-06
rs16870964	rs62414182	chr6:93914022	0.01053673	0.83165997	EPHA7	ENSG00000135333.13	Testis	A=0.835	G=0.165	0.3716	8.26E-06
rs16870964	rs113888577	chr6:93698093	0.00093064	1	EPHA7	ENSG00000135333.13	Brain - Anterior cingulate cortex (BA24)	C=0.988	T=0.012	0.66365	8.36E-06
rs16870964	rs16870780	chr6:93826505	0.00665985	1	EPHA7	ENSG00000135333.13	Lung	T=0.92	A=0.08	0.385133	9.62E-06
rs16870964	rs1408285	chr6:93893666	0.0924402	0.90638377	EPHA7	ENSG00000135333.13	Esophagus - Muscularis	A=0.593	C=0.407	0.105615	9.88E-06
rs16870964	rs7761955	chr6:93896624	0.0924402	0.90638377	EPHA7	ENSG00000135333.13	Esophagus - Muscularis	T=0.593	C=0.407	0.105615	9.88E-06
rs16870964	rs7772899	chr6:93898633	0.0924402	0.90638377	EPHA7	ENSG00000135333.13	Esophagus - Muscularis	T=0.593	C=0.407	0.105615	9.88E-06
rs16870964	rs1590384	chr6:93837434	0.02992801	0.95358066	EPHA7	ENSG00000135333.13	Minor Salivary Gland	C=0.701	G=0.299	-0.24131	1.00E-05
rs16870964	rs1924474	chr6:93837991	0.02992801	0.95358066	EPHA7	ENSG00000135333.13	Minor Salivary Gland	C=0.701	T=0.299	-0.24131	1.00E-05
rs16870964	rs10944652	chr6:93838348	0.02992801	0.95358066	EPHA7	ENSG00000135333.13	Minor Salivary Gland	C=0.701	G=0.299	-0.24131	1.00E-05
rs16870964	rs12204186	chr6:93838665	0.02992801	0.95358066	EPHA7	ENSG00000135333.13	Minor Salivary Gland	C=0.701	T=0.299	-0.24131	1.00E-05
rs16870964	rs633279	chr6:93840705	0.02992801	0.95358066	EPHA7	ENSG00000135333.13	Minor Salivary Gland	A=0.299	T=0.701	0.24131	1.00E-05
rs16870964	rs634060	chr6:93840849	0.02992801	0.95358066	EPHA7	ENSG00000135333.13	Minor Salivary Gland	A=0.299	G=0.701	0.24131	1.00E-05
rs16870964	rs568957	chr6:93840855	0.02992801	0.95358066	EPHA7	ENSG00000135333.13	Minor Salivary Gland	G=0.299	T=0.701	0.24131	1.00E-05
rs16870964	rs34544163	chr6:93841565	0.02992801	0.95358066	EPHA7	ENSG00000135333.13	Minor Salivary Gland	-=0.299	A=0.701	0.24131	1.00E-05
rs16870964	rs562379	chr6:93841592	0.02992801	0.95358066	EPHA7	ENSG00000135333.13	Minor Salivary Gland	G=0.299	A=0.701	0.24131	1.00E-05
rs16870964	rs560731	chr6:93841734	0.02992801	0.95358066	EPHA7	ENSG00000135333.13	Minor Salivary Gland	A=0.299	G=0.701	0.24131	1.00E-05
rs16870964	rs650642	chr6:93842232	0.02992801	0.95358066	EPHA7	ENSG00000135333.13	Minor Salivary Gland	G=0.299	T=0.701	0.24131	1.00E-05
rs16870964	rs650747	chr6:93842298	0.02992801	0.95358066	EPHA7	ENSG00000135333.13	Minor Salivary Gland	T=0.299	C=0.701	0.24131	1.00E-05
rs16870964	rs1930934	chr6:93842533	0.02992801	0.95358066	EPHA7	ENSG00000135333.13	Minor Salivary Gland	A=0.299	G=0.701	0.24131	1.00E-05
rs16870964	rs9353987	chr6:93843588	0.02992801	0.95358066	EPHA7	ENSG00000135333.13	Minor Salivary Gland	A=0.299	G=0.701	0.24131	1.00E-05
rs16870964	rs9363034	chr6:93844136	0.02992801	0.95358066	EPHA7	ENSG00000135333.13	Minor Salivary Gland	G=0.299	A=0.701	0.24131	1.00E-05
rs16870964	rs9353989	chr6:93844383	0.02992801	0.95358066	EPHA7	ENSG00000135333.13	Minor Salivary Gland	G=0.299	T=0.701	0.24131	1.00E-05
rs16870964	rs74822095	chr6:93741512	0.00386244	1	EPHA7	ENSG00000135333.13	Adipose - Subcutaneous	C=0.952	T=0.048	-0.476757	1.06E-05
rs16870964	rs76282783	chr6:93576050	0.0033586	1	EPHA7	ENSG00000135333.13	Lung	C=0.958	A=0.042	0.702179	1.18E-05
rs16870964	rs10693258	chr6:93902052	0.01024365	1	EPHA7	ENSG00000135333.13	Esophagus - Gastroesophageal Junction	-=0.883	TTG=0.117	0.189047	1.21E-05
rs16870964	rs6936693	chr6:93903686	0.0100474	1	EPHA7	ENSG00000135333.13	Esophagus - Mucosa	T=0.885	C=0.115	0.361801	1.30E-05
rs16870964	rs11967483	chr6:93903691	0.0100474	1	EPHA7	ENSG00000135333.13	Esophagus - Mucosa	A=0.885	T=0.115	0.36623	1.31E-05
rs16870964	rs72926533	chr6:93740212	0.00069588	1	EPHA7	ENSG00000135333.13	Minor Salivary Gland	C=0.991	T=0.009	-1.15096	1.32E-05
rs16870964	rs188317446	chr6:93743394	0.00069588	1	EPHA7	ENSG00000135333.13	Minor Salivary Gland	G=0.991	C=0.009	-1.15096	1.32E-05
rs16870964	rs72926535	chr6:93747452	0.00069588	1	EPHA7	ENSG00000135333.13	Minor Salivary Gland	C=0.991	T=0.009	-1.15096	1.32E-05

rs16870964	rs117415227	chr6:93735745	0.00386244	1	EPHA7	ENSG00000135333.13	Adipose - Subcutaneous	T=0.952	G=0.048	-0.47253	1.37E-05
rs16870964	rs11966926	chr6:93897338	0.00985203	1	EPHA7	ENSG00000135333.13	Esophagus - Mucosa	A=0.887	G=0.113	0.374015	1.38E-05
rs16870964	rs199888991	chr6:93784078	0.00038505	1	EPHA7	ENSG00000135333.13	Adipose - Subcutaneous	-=0.995	T=0.005	-0.796053	1.44E-05
rs16870964	rs111429740	chr6:93838453	0.00675039	1	EPHA7	ENSG00000135333.13	Lung	G=0.919	A=0.081	0.383525	1.55E-05
rs16870964	rs72919045	chr6:93940859	0.0136905	0.80594136	EPHA7	ENSG00000135333.13	Breast - Mammary Tissue	T=0.785	A=0.215	0.313719	1.71E-05
rs16870964	rs16870789	chr6:93827963	0.00665985	1	EPHA7	ENSG00000135333.13	Lung	C=0.92	T=0.08	0.374368	1.81E-05
rs16870964	rs145810085	chr6:93833819	0.00061794	1	EPHA7	ENSG00000135333.13	Adipose - Subcutaneous	G=0.992	A=0.008	-0.735109	1.88E-05
rs16870964	rs7774823	chr6:93830545	0.02962575	0.95327016	EPHA7	ENSG00000135333.13	Minor Salivary Gland	G=0.703	A=0.297	-0.232136	1.89E-05
rs16870964	rs1951907	chr6:93831192	0.02962575	0.95327016	EPHA7	ENSG00000135333.13	Minor Salivary Gland	T=0.703	C=0.297	-0.232136	1.89E-05
rs16870964	rs35306488	chr6:93832227	0.02917557	0.95279655	EPHA7	ENSG00000135333.13	Minor Salivary Gland	-=0.706	T=0.294	-0.232136	1.89E-05
rs16870964	rs10944648	chr6:93832280	0.02962575	0.95327016	EPHA7	ENSG00000135333.13	Minor Salivary Gland	A=0.703	G=0.297	-0.232136	1.89E-05
rs16870964	rs7751375	chr6:93832923	0.02977667	0.95342593	EPHA7	ENSG00000135333.13	Minor Salivary Gland	C=0.702	A=0.298	-0.232136	1.89E-05
rs16870964	rs7757292	chr6:93834165	0.02962575	0.95327016	EPHA7	ENSG00000135333.13	Minor Salivary Gland	C=0.703	A=0.297	-0.232136	1.89E-05
rs16870964	rs11962709	chr6:93891195	0.00985203	1	EPHA7	ENSG00000135333.13	Esophagus - Gastroesophageal Junction	A=0.887	G=0.113	0.198094	1.91E-05
rs16870964	rs71540101	chr6:93892043	0.00985203	1	EPHA7	ENSG00000135333.13	Esophagus - Gastroesophageal Junction	AGGTATATC=0.887	-=0.113	0.198094	1.91E-05
rs16870964	rs35762480	chr6:93906245	0.0100474	1	EPHA7	ENSG00000135333.13	Esophagus - Gastroesophageal Junction	G=0.885	C=0.115	0.19742	1.95E-05
rs16870964	rs16870850	chr6:93841079	0.00711451	1	EPHA7	ENSG00000135333.13	Lung	T=0.916	C=0.084	0.361979	2.02E-05
rs16870964	rs12528846	chr6:93828842	0.00665985	1	EPHA7	ENSG00000135333.13	Lung	A=0.92	G=0.08	0.377147	2.03E-05
rs16870964	rs12527296	chr6:93829143	0.00665985	1	EPHA7	ENSG00000135333.13	Lung	T=0.92	C=0.08	0.377147	2.03E-05
rs16870964	rs72928575	chr6:93829399	0.00675039	1	EPHA7	ENSG00000135333.13	Lung	G=0.919	T=0.081	0.377147	2.03E-05
rs16870964	rs72928579	chr6:93829658	0.00665985	1	EPHA7	ENSG00000135333.13	Lung	G=0.92	A=0.08	0.377147	2.03E-05
rs16870964	rs77007574	chr6:93829960	0.00665985	1	EPHA7	ENSG00000135333.13	Lung	T=0.92	C=0.08	0.377147	2.03E-05
rs16870964	rs72928582	chr6:93830015	0.00665985	1	EPHA7	ENSG00000135333.13	Lung	G=0.92	A=0.08	0.377147	2.03E-05
rs16870964	rs16870801	chr6:93830951	0.00665985	1	EPHA7	ENSG00000135333.13	Lung	T=0.92	C=0.08	0.377147	2.03E-05
rs16870964	rs16870805	chr6:93834075	0.00665985	1	EPHA7	ENSG00000135333.13	Lung	G=0.92	T=0.08	0.377147	2.03E-05
rs16870964	rs150304721	chr6:93835801	0.00684112	1	EPHA7	ENSG00000135333.13	Lung	A=0.918	-=0.082	0.361316	2.04E-05
rs16870964	rs11967483	chr6:93903691	0.0100474	1	EPHA7	ENSG00000135333.13	Esophagus - Gastroesophageal Junction	A=0.885	T=0.115	0.196042	2.11E-05
rs16870964	rs16870855	chr6:93841440	0.00675039	1	EPHA7	ENSG00000135333.13	Lung	C=0.919	T=0.081	0.4288	2.14E-05
rs16870964	rs13212701	chr6:93902787	0.0100474	1	EPHA7	ENSG00000135333.13	Esophagus - Gastroesophageal Junction	T=0.885	C=0.115	0.195748	2.16E-05
rs16870964	rs16880183	chr6:93889701	0.01512424	1	EPHA7	ENSG00000135333.13	Testis	A=0.836	G=0.164	0.353058	2.24E-05
rs16870964	rs12199960	chr6:93923853	0.01357942	0.80503876	EPHA7	ENSG00000135333.13	Breast - Mammary Tissue	A=0.786	C=0.214	0.305305	2.45E-05
rs16870964	rs12200456	chr6:93924735	0.01380189	0.80683564	EPHA7	ENSG00000135333.13	Breast - Mammary Tissue	A=0.784	G=0.216	0.305305	2.45E-05
rs16870964	rs72919020	chr6:93925349	0.0136905	0.80594136	EPHA7	ENSG00000135333.13	Breast - Mammary Tissue	G=0.785	C=0.215	0.305305	2.45E-05
rs16870964	rs12190544	chr6:93925959	0.0136905	0.80594136	EPHA7	ENSG00000135333.13	Breast - Mammary Tissue	G=0.785	A=0.215	0.305305	2.45E-05
rs16870964	rs12206216	chr6:93927822	0.01380189	0.80683564	EPHA7	ENSG00000135333.13	Breast - Mammary Tissue	A=0.784	G=0.216	0.305305	2.45E-05
rs16870964	rs12196073	chr6:93928926	0.0136905	0.80594136	EPHA7	ENSG00000135333.13	Breast - Mammary Tissue	C=0.785	G=0.215	0.305305	2.45E-05
rs16870964	rs12196420	chr6:93929507	0.0136905	0.80594136	EPHA7	ENSG00000135333.13	Breast - Mammary Tissue	C=0.785	T=0.215	0.305305	2.45E-05
rs16870964	rs12201744	chr6:93930642	0.01357942	0.80503876	EPHA7	ENSG00000135333.13	Breast - Mammary Tissue	A=0.786	T=0.214	0.305305	2.45E-05
rs16870964	rs12195545	chr6:93930755	0.01357942	0.80503876	EPHA7	ENSG00000135333.13	Breast - Mammary Tissue	T=0.786	G=0.214	0.305305	2.45E-05
rs16870964	rs143708097	chr6:93931051	0.01357942	0.80503876	EPHA7	ENSG00000135333.13	Breast - Mammary Tissue	-=0.786	CCTA=0.214	0.305305	2.45E-05
rs16870964	rs72919042	chr6:93935578	0.0136905	0.80594136	EPHA7	ENSG00000135333.13	Breast - Mammary Tissue	C=0.785	T=0.215	0.305305	2.45E-05
rs16870964	rs7772178	chr6:93849608	0.01024365	1	EPHA7	ENSG00000135333.13	Testis	C=0.883	T=0.117	0.425527	2.47E-05
rs16870964	rs72919047	chr6:93941158	0.0136905	0.80594136	EPHA7	ENSG00000135333.13	Breast - Mammary Tissue	T=0.785	C=0.215	0.304408	2.54E-05
rs16870964	rs12209980	chr6:93943432	0.0136905	0.80594136	EPHA7	ENSG00000135333.13	Breast - Mammary Tissue	T=0.785	G=0.215	0.304408	2.54E-05
rs16870964	rs6923008	chr6:93887045	0.01512424	1	EPHA7	ENSG00000135333.13	Testis	G=0.836	T=0.164	0.351036	2.54E-05
rs16870964	rs60592958	chr6:93887933	0.01512424	1	EPHA7	ENSG00000135333.13	Testis	C=0.836	T=0.164	0.351036	2.54E-05
rs16870964	rs147445521	chr6:93825169	0.00558845	1	EPHA7	ENSG00000135333.13	Lung	TGT=0.932	-=0.068	0.417524	2.57E-05
rs16870964	rs118177782	chr6:93767900	0.00204519	1	EPHA7	ENSG00000135333.13	Brain - Anterior cingulate cortex (BA24)	C=0.974	T=0.026	0.590137	2.71E-05
rs16870964	rs73758259	chr6:93828724	0.00693206	1	EPHA7	ENSG00000135333.13	Lung	G=0.917	T=0.083	0.354112	2.72E-05
rs16870964	rs16870796	chr6:93828781	0.00665985	1	EPHA7	ENSG00000135333.13	Lung	T=0.92	G=0.08	0.369069	2.76E-05
rs16870964	rs72928577	chr6:93829400	0.00675039	1	EPHA7	ENSG00000135333.13	Lung	C=0.919	T=0.081	0.369069	2.76E-05

rs16870964	rs11966926	chr6:93897338	0.00985203	1	EPHA7	ENSG00000135333.13	Esophagus - Gastroesophageal Junction	A=0.887	G=0.113	0.196345	2.79E-05
rs16870964	rs117717826	chr6:94036945	0.00196452	1	EPHA7	ENSG00000135333.13	Heart - Left Ventricle	T=0.975	A=0.025	-0.389622	2.96E-05
rs16870964	rs56378615	chr6:93900091	0.01090501	0.90559309	EPHA7	ENSG00000135333.13	Testis	C=0.853	T=0.147	0.358985	3.02E-05
rs16870964	rs6936693	chr6:93903686	0.0100474	1	EPHA7	ENSG00000135333.13	Esophagus - Gastroesophageal Junction	T=0.885	C=0.115	0.189133	3.05E-05
rs16870964	rs71558449	chr6:93828589	0.00261449	1	EPHA7	ENSG00000135333.13	Esophagus - Muscularis	G=0.967	A=0.033	0.272458	3.14E-05
rs16870964	rs6922405	chr6:93842847	0.00675039	1	EPHA7	ENSG00000135333.13	Lung	T=0.919	C=0.081	0.370721	3.25E-05
rs16870964	rs72930416	chr6:93844150	0.00675039	1	EPHA7	ENSG00000135333.13	Lung	T=0.919	C=0.081	0.370721	3.25E-05
rs16870964	rs34948845	chr6:93894820	0.01174419	1	EPHA7	ENSG00000135333.13	Esophagus - Gastroesophageal Junction	G=0.868	A=0.132	0.186343	3.27E-05
rs16870964	rs16870846	chr6:93840909	0.00675039	1	EPHA7	ENSG00000135333.13	Lung	C=0.919	G=0.081	0.371767	3.40E-05
rs16870964	rs1319460	chr6:93846637	0.02415283	1	EPHA7	ENSG00000135333.13	Minor Salivary Gland	C=0.239	T=0.761	0.230876	3.54E-05
rs16870964	rs538751	chr6:93848400	0.02415283	1	EPHA7	ENSG00000135333.13	Minor Salivary Gland	A=0.239	C=0.761	0.230876	3.54E-05
rs16870964	rs11422146	chr6:93849652	0.02402084	1	EPHA7	ENSG00000135333.13	Minor Salivary Gland	-=0.238	T=0.762	0.230876	3.54E-05
rs16870964	rs34832688	chr6:93891054	0.00985203	1	EPHA7	ENSG00000135333.13	Esophagus - Gastroesophageal Junction	A=0.887	C=0.113	0.187735	3.54E-05
rs16870964	rs1319460	chr6:93846637	0.02415283	1	EPHA7	ENSG00000135333.13	Testis	C=0.239	T=0.761	-0.330556	3.66E-05
rs16870964	rs538751	chr6:93848400	0.02415283	1	EPHA7	ENSG00000135333.13	Testis	A=0.239	C=0.761	-0.330556	3.66E-05
rs16870964	rs11422146	chr6:93849652	0.02402084	1	EPHA7	ENSG00000135333.13	Testis	-=0.238	T=0.762	-0.330556	3.66E-05
rs16870964	rs6931234	chr6:93927181	0.01380189	0.80683564	EPHA7	ENSG00000135333.13	Breast - Mammary Tissue	T=0.784	C=0.216	0.288667	3.69E-05
rs16870964	rs117415227	chr6:93735745	0.00386244	1	EPHA7	ENSG00000135333.13	Lung	T=0.952	G=0.048	-0.50856	4.07E-05
rs16870964	rs72919018	chr6:93924471	0.0136905	0.80594136	EPHA7	ENSG00000135333.13	Breast - Mammary Tissue	G=0.785	C=0.215	0.291651	4.19E-05
rs16870964	rs12189899	chr6:93931092	0.0136905	0.80594136	EPHA7	ENSG00000135333.13	Breast - Mammary Tissue	C=0.785	T=0.215	0.291651	4.19E-05
rs16870964	rs6935219	chr6:93889277	0.01512424	1	EPHA7	ENSG00000135333.13	Testis	A=0.836	G=0.164	0.33974	4.29E-05
rs16870964	rs12374628	chr6:93934768	0.0136905	0.80594136	EPHA7	ENSG00000135333.13	Breast - Mammary Tissue	T=0.785	C=0.215	0.290954	4.31E-05
rs16870964	rs11962003	chr6:93890071	0.01174419	1	EPHA7	ENSG00000135333.13	Esophagus - Mucosa	A=0.868	G=0.132	0.333682	4.39E-05
rs16870964	rs11966984	chr6:93890176	0.01174419	1	EPHA7	ENSG00000135333.13	Esophagus - Mucosa	G=0.868	A=0.132	0.333682	4.39E-05
rs16870964	rs140182812	chr6:93804212	0.00061794	1	EPHA7	ENSG00000135333.13	Adipose - Subcutaneous	G=0.992	A=0.008	-0.729088	4.39E-05
rs16870964	rs11962003	chr6:93890071	0.01174419	1	EPHA7	ENSG00000135333.13	Esophagus - Gastroesophageal Junction	A=0.868	G=0.132	0.183007	4.40E-05
rs16870964	rs11966984	chr6:93890176	0.01174419	1	EPHA7	ENSG00000135333.13	Esophagus - Gastroesophageal Junction	G=0.868	A=0.132	0.183007	4.40E-05
rs16870964	rs34948845	chr6:93894820	0.01174419	1	EPHA7	ENSG00000135333.13	Esophagus - Mucosa	G=0.868	A=0.132	0.334329	4.44E-05
rs16870964	rs10686381	chr6:93887552	0.01512424	1	EPHA7	ENSG00000135333.13	Testis	-=0.836	AAGT=0.164	0.337644	4.84E-05
rs16870964	rs2224853	chr6:93890865	0.04687736	0.93250134	EPHA7	ENSG00000135333.13	Esophagus - Muscularis	T=0.588	C=0.412	-0.0928827	5.11E-05
rs16870964	rs41273625	chr6:93952851	0.00515009	1	EPHA7	ENSG00000135333.13	Esophagus - Muscularis	C=0.937	G=0.063	0.224035	5.17E-05
rs16870964	rs78355320	chr6:93821638	0.00386244	1	EPHA7	ENSG00000135333.13	Adipose - Subcutaneous	T=0.952	A=0.048	-0.431835	5.33E-05
rs16870964	rs12530331	chr6:93830460	0.00675039	1	EPHA7	ENSG00000135333.13	Lung	G=0.919	C=0.081	0.35653	5.33E-05
rs16870964	rs72920784	chr6:93673551	0.00156365	1	EPHA7	ENSG00000135333.13	Adipose - Subcutaneous	A=0.98	G=0.02	-0.579253	5.40E-05
rs16870964	rs72920785	chr6:93675496	0.00156365	1	EPHA7	ENSG00000135333.13	Adipose - Subcutaneous	G=0.98	C=0.02	-0.579253	5.40E-05
rs16870964	rs74468395	chr6:94017659	0.00261449	1	EPHA7	ENSG00000135333.13	Heart - Left Ventricle	C=0.967	T=0.033	-0.353	6.19E-05
rs16870964	rs117256127	chr6:93852714	0.00188402	1	EPHA7	ENSG00000135333.13	Adipose - Subcutaneous	T=0.976	C=0.024	-0.674139	6.42E-05
rs16870964	rs117425371	chr6:93852717	0.00188402	1	EPHA7	ENSG00000135333.13	Adipose - Subcutaneous	A=0.976	G=0.024	-0.674139	6.42E-05
rs16870964	rs145519743	chr6:93884032	0.00196452	1	EPHA7	ENSG00000135333.13	Adipose - Subcutaneous	G=0.975	A=0.025	-0.674139	6.42E-05
rs16870964	rs9351349	chr6:93832104	0.01429205	0.92237654	EPHA7	ENSG00000135333.13	Adipose - Subcutaneous	G=0.179	A=0.821	0.228214	6.48E-05
rs16870964	rs147330775	chr6:93915683	0.00046253	1	EPHA7	ENSG00000135333.13	Skin - Sun Exposed (Lower leg)	C=0.994	T=0.006	-0.47159	6.56E-05
rs16870964	rs117109139	chr6:93861398	0.00302617	1	EPHA7	ENSG00000135333.13	Esophagus - Muscularis	C=0.962	G=0.038	0.257424	7.15E-05
rs16870964	rs650711	chr6:93842280	0.01429205	0.92237654	EPHA7	ENSG00000135333.13	Adipose - Subcutaneous	T=0.821	G=0.179	-0.226702	7.44E-05
rs16870964	rs768382	chr6:93826168	0.01418212	0.92194289	EPHA7	ENSG00000135333.13	Adipose - Subcutaneous	T=0.178	C=0.822	0.223191	7.87E-05
rs16870964	rs1324110	chr6:93913200	0.08054639	0.90125638	EPHA7	ENSG00000135333.13	Heart - Atrial Appendage	G=0.563	C=0.437	0.0819979	9.78E-05
rs16870964	rs41273625	chr6:93952851	0.00515009	1	EPHA7	ENSG00000135333.13	Esophagus - Gastroesophageal Junction	C=0.937	G=0.063	0.249826	0.00010545
rs16870964	rs11970583	chr6:93895756	0.02973083	1	EPHA7	ENSG00000135333.13	Esophagus - Muscularis	C=0.722	T=0.278	0.0990772	0.00011475
rs16870964	rs2065582	chr6:93896309	0.02973083	1	EPHA7	ENSG00000135333.13	Esophagus - Muscularis	G=0.722	T=0.278	0.0990772	0.00011475
rs16870964	rs2147225	chr6:93898127	0.02973083	1	EPHA7	ENSG00000135333.13	Esophagus - Muscularis	T=0.722	A=0.278	0.0990772	0.00011475
rs16870964	rs13205265	chr6:93898896	0.02973083	1	EPHA7	ENSG00000135333.13	Esophagus - Muscularis	C=0.722	T=0.278	0.0990772	0.00011475
rs16870964	rs35480101	chr6:93899371	0.0295839	1	EPHA7	ENSG00000135333.13	Esophagus - Muscularis	G=0.723	T=0.277	0.0990772	0.00011475

rs16870964	rs145046790	chr6:93899520	0.02973083	1	EPHA7	ENSG00000135333.13	Esophagus - Muscularis	AT=0.722	-=0.278	0.0990772	0.00011475
rs16870964	rs192604147	chr6:94240629	0.00069588	1	EPHA7	ENSG00000135333.13	Heart - Left Ventricle	A=0.991	G=0.009	-0.514265	0.00011598
rs16870964	rs1324103	chr6:93901016	0.04436088	0.89972089	EPHA7	ENSG00000135333.13	Esophagus - Muscularis	A=0.584	G=0.416	-0.0876121	0.00012747
rs16870964	rs1324104	chr6:93901538	0.04436088	0.89972089	EPHA7	ENSG00000135333.13	Esophagus - Muscularis	T=0.584	G=0.416	-0.0876121	0.00012747
rs16870964	rs16880179	chr6:93889506	0.013087	1	EPHA7	ENSG00000135333.13	Testis	G=0.855	A=0.145	0.335079	0.00013631
rs16870964	rs59810007	chr6:93891733	0.013087	1	EPHA7	ENSG00000135333.13	Testis	T=0.855	C=0.145	0.335079	0.00013631
rs16870964	rs6901961	chr6:93892700	0.013087	1	EPHA7	ENSG00000135333.13	Testis	T=0.855	A=0.145	0.335079	0.00013631
rs16870964	rs60804851	chr6:93894513	0.013087	1	EPHA7	ENSG00000135333.13	Testis	C=0.855	G=0.145	0.335079	0.00013631
rs16870964	rs6901870	chr6:93895903	0.013087	1	EPHA7	ENSG00000135333.13	Testis	A=0.855	C=0.145	0.335079	0.00013631
rs16870964	rs74822095	chr6:93741512	0.00386244	1	EPHA7	ENSG00000135333.13	Lung	C=0.952	T=0.048	-0.471073	0.00014377
rs16870964	rs6901827	chr6:93895828	0.02973083	1	EPHA7	ENSG00000135333.13	Esophagus - Muscularis	A=0.722	G=0.278	0.0976714	0.00014821
rs16870964	rs2181805	chr6:93898026	0.02973083	1	EPHA7	ENSG00000135333.13	Esophagus - Muscularis	T=0.722	C=0.278	0.0976714	0.00014821
rs16870964	rs71298717	chr6:93898609	0.02617495	1	EPHA7	ENSG00000135333.13	Esophagus - Muscularis	-=0.747	ATTTTAGGAATA=0.25:	0.0976714	0.00014821
rs16870964	rs143801031	chr6:93750441	0.00132507	1	EPHA7	ENSG00000135333.13	Adipose - Subcutaneous	T=0.983	C=0.017	-0.536087	0.00015072
rs16870964	rs6922792	chr6:93887222	0.013087	1	EPHA7	ENSG00000135333.13	Testis	A=0.855	G=0.145	0.333048	0.00015245
rs16870964	rs117365486	chr6:93820610	0.00253266	1	EPHA7	ENSG00000135333.13	Nerve - Tibial	C=0.968	T=0.032	-0.404506	0.000162
rs16870964	rs148086058	chr6:93820887	0.00253266	1	EPHA7	ENSG00000135333.13	Nerve - Tibial	C=0.968	A=0.032	-0.404506	0.000162
rs16870964	rs11966965	chr6:93890049	0.0295839	1	EPHA7	ENSG00000135333.13	Esophagus - Muscularis	G=0.723	T=0.277	0.096593	0.00016937
rs16870964	rs35373649	chr6:93891073	0.0295839	1	EPHA7	ENSG00000135333.13	Esophagus - Muscularis	A=0.723	G=0.277	0.096593	0.00016937
rs16870964	rs6921600	chr6:93892299	0.02973083	1	EPHA7	ENSG00000135333.13	Esophagus - Muscularis	G=0.722	T=0.278	0.096593	0.00016937
rs16870964	rs6901416	chr6:93892412	0.02973083	1	EPHA7	ENSG00000135333.13	Esophagus - Muscularis	T=0.722	G=0.278	0.096593	0.00016937
rs16870964	rs6454946	chr6:93902683	0.04477275	0.90019841	EPHA7	ENSG00000135333.13	Esophagus - Muscularis	A=0.583	G=0.417	-0.0835518	0.00021296
rs16870964	rs1324106	chr6:93902867	0.04477275	0.90019841	EPHA7	ENSG00000135333.13	Esophagus - Muscularis	T=0.583	A=0.417	-0.0835518	0.00021296
rs16870964	rs1324107	chr6:93903050	0.04477275	0.90019841	EPHA7	ENSG00000135333.13	Esophagus - Muscularis	C=0.583	G=0.417	-0.0835518	0.00021296
rs16870964	rs658231	chr6:93903544	0.04477275	0.90019841	EPHA7	ENSG00000135333.13	Esophagus - Muscularis	C=0.583	T=0.417	-0.0835518	0.00021296
rs16870964	rs1953145	chr6:93890592	0.0295839	1	EPHA7	ENSG00000135333.13	Esophagus - Muscularis	G=0.723	A=0.277	0.0952131	0.00021698
rs16870964	rs12110542	chr6:93894263	0.02973083	1	EPHA7	ENSG00000135333.13	Esophagus - Muscularis	T=0.722	G=0.278	0.0952131	0.00021698
rs16870964	rs1953146	chr6:93897891	0.02973083	1	EPHA7	ENSG00000135333.13	Esophagus - Muscularis	C=0.722	G=0.278	0.0952131	0.00021698
rs16870964	rs182357880	chr6:93879803	0.00054015	1	EPHA7	ENSG00000135333.13	Skin - Sun Exposed (Lower leg)	T=0.993	C=0.007	-0.395624	0.00022421
rs16870964	rs144384339	chr6:93897863	0.0016435	1	EPHA7	ENSG00000135333.13	Skin - Sun Exposed (Lower leg)	G=0.979	A=0.021	-0.418007	0.00029351
rs16880170	rs117365486	chr6:93820610	0.0062598	1	EPHA7	ENSG00000135333.13	Adipose - Subcutaneous	C=0.968	T=0.032	-0.679264	8.33E-10
rs16880170	rs148086058	chr6:93820887	0.0062598	1	EPHA7	ENSG00000135333.13	Adipose - Subcutaneous	C=0.968	A=0.032	-0.679264	8.33E-10
rs16880170	rs189136709	chr6:93783798	0.00095171	1	EPHA7	ENSG00000135333.13	Brain - Anterior cingulate cortex (BA24)	G=0.995	A=0.005	1.35876	1.60E-08
rs16880170	rs1386276	chr6:94082749	0.00463452	0.82147294	EPHA7	ENSG00000135333.13	Heart - Left Ventricle	A=0.965	G=0.035	-0.463277	1.18E-07
rs16880170	rs79718749	chr6:94098832	0.00463452	0.82147294	EPHA7	ENSG00000135333.13	Heart - Left Ventricle	T=0.965	C=0.035	-0.463277	1.18E-07
rs16880170	rs12661215	chr6:94099119	0.00463452	0.82147294	EPHA7	ENSG00000135333.13	Heart - Left Ventricle	C=0.965	T=0.035	-0.463277	1.18E-07
rs16880170	rs16871305	chr6:94111173	0.00463452	0.82147294	EPHA7	ENSG00000135333.13	Heart - Left Ventricle	A=0.965	T=0.035	-0.463277	1.18E-07
rs16880170	rs80300952	chr6:94115209	0.00463452	0.82147294	EPHA7	ENSG00000135333.13	Heart - Left Ventricle	T=0.965	C=0.035	-0.463277	1.18E-07
rs16880170	rs146471836	chr6:93817042	0.00405384	0.80473602	EPHA7	ENSG00000135333.13	Adipose - Subcutaneous	C=0.968	T=0.032	-0.617165	8.35E-07
rs16880170	rs141174921	chr6:93733454	0.00405384	0.80473602	EPHA7	ENSG00000135333.13	Adipose - Subcutaneous	A=0.968	G=0.032	-0.598481	1.67E-06
rs16880170	rs117376030	chr6:94203683	0.00505495	1	EPHA7	ENSG00000135333.13	Esophagus - Gastroesophageal Junction	T=0.974	A=0.026	-0.384156	2.51E-06
rs16880170	rs72928597	chr6:93839843	0.01668447	1	EPHA7	ENSG00000135333.13	Lung	A=0.919	G=0.081	0.42955	4.55E-06
rs16880170	rs16870853	chr6:93841320	0.01668447	1	EPHA7	ENSG00000135333.13	Lung	T=0.919	C=0.081	0.42955	4.55E-06
rs16880170	rs1930933	chr6:93842544	0.08134794	1	EPHA7	ENSG00000135333.13	Minor Salivary Gland	C=0.299	T=0.701	0.246092	4.56E-06
rs16880170	rs142723671	chr6:94076414	0.00425987	1	EPHA7	ENSG00000135333.13	Heart - Left Ventricle	G=0.978	C=0.022	-0.495017	4.73E-06
rs16880170	rs147330775	chr6:93915683	0.0011432	1	EPHA7	ENSG00000135333.13	Brain - Anterior cingulate cortex (BA24)	C=0.994	T=0.006	0.808887	6.34E-06
rs16880170	rs16870793	chr6:93828599	0.0162374	1	EPHA7	ENSG00000135333.13	Lung	G=0.921	A=0.079	0.419095	6.62E-06
rs16880170	rs72928592	chr6:93837959	0.01646069	1	EPHA7	ENSG00000135333.13	Lung	T=0.92	C=0.08	0.419095	6.62E-06
rs16880170	rs1575540	chr6:93836577	0.01165705	1	EPHA7	ENSG00000135333.13	Esophagus - Muscularis	G=0.942	A=0.058	0.272463	7.68E-06
rs16880170	rs544639807	chr6:93883657	0.00152731	1	EPHA7	ENSG00000135333.13	Minor Salivary Gland	G=0.992	A=0.008	-1.34812	8.25E-06
rs16880170	rs36114673	chr6:93847056	0.01251379	1	EPHA7	ENSG00000135333.13	Esophagus - Muscularis	A=0.938	T=0.062	0.268662	8.26E-06

rs16880170	rs113888577	chr6:93698093	0.00230019	1	EPHA7	ENSG00000135333.13	Brain - Anterior cingulate cortex (BA24)	C=0.988	T=0.012	0.66365	8.36E-06
rs16880170	rs16870780	chr6:93826505	0.01646069	1	EPHA7	ENSG00000135333.13	Lung	T=0.92	A=0.08	0.385133	9.62E-06
rs16880170	rs1590384	chr6:93837434	0.08134794	1	EPHA7	ENSG00000135333.13	Minor Salivary Gland	C=0.701	G=0.299	-0.24131	1.00E-05
rs16880170	rs1924474	chr6:93837991	0.08134794	1	EPHA7	ENSG00000135333.13	Minor Salivary Gland	C=0.701	T=0.299	-0.24131	1.00E-05
rs16880170	rs10944652	chr6:93838348	0.08134794	1	EPHA7	ENSG00000135333.13	Minor Salivary Gland	C=0.701	G=0.299	-0.24131	1.00E-05
rs16880170	rs12204186	chr6:93838665	0.08134794	1	EPHA7	ENSG00000135333.13	Minor Salivary Gland	C=0.701	T=0.299	-0.24131	1.00E-05
rs16880170	rs633279	chr6:93840705	0.08134794	1	EPHA7	ENSG00000135333.13	Minor Salivary Gland	A=0.299	T=0.701	0.24131	1.00E-05
rs16880170	rs634060	chr6:93840849	0.08134794	1	EPHA7	ENSG00000135333.13	Minor Salivary Gland	A=0.299	G=0.701	0.24131	1.00E-05
rs16880170	rs568957	chr6:93840855	0.08134794	1	EPHA7	ENSG00000135333.13	Minor Salivary Gland	G=0.299	T=0.701	0.24131	1.00E-05
rs16880170	rs34544163	chr6:93841565	0.08134794	1	EPHA7	ENSG00000135333.13	Minor Salivary Gland	-=0.299	A=0.701	0.24131	1.00E-05
rs16880170	rs562379	chr6:93841592	0.08134794	1	EPHA7	ENSG00000135333.13	Minor Salivary Gland	G=0.299	A=0.701	0.24131	1.00E-05
rs16880170	rs560731	chr6:93841734	0.08134794	1	EPHA7	ENSG00000135333.13	Minor Salivary Gland	A=0.299	G=0.701	0.24131	1.00E-05
rs16880170	rs650642	chr6:93842232	0.08134794	1	EPHA7	ENSG00000135333.13	Minor Salivary Gland	G=0.299	T=0.701	0.24131	1.00E-05
rs16880170	rs650747	chr6:93842298	0.08134794	1	EPHA7	ENSG00000135333.13	Minor Salivary Gland	T=0.299	C=0.701	0.24131	1.00E-05
rs16880170	rs1930934	chr6:93842533	0.08134794	1	EPHA7	ENSG00000135333.13	Minor Salivary Gland	A=0.299	G=0.701	0.24131	1.00E-05
rs16880170	rs9353987	chr6:93843588	0.08134794	1	EPHA7	ENSG00000135333.13	Minor Salivary Gland	A=0.299	G=0.701	0.24131	1.00E-05
rs16880170	rs9363034	chr6:93844136	0.08134794	1	EPHA7	ENSG00000135333.13	Minor Salivary Gland	G=0.299	A=0.701	0.24131	1.00E-05
rs16880170	rs9353989	chr6:93844383	0.08134794	1	EPHA7	ENSG00000135333.13	Minor Salivary Gland	G=0.299	T=0.701	0.24131	1.00E-05
rs16880170	rs76282783	chr6:93576050	0.00601496	0.85122745	EPHA7	ENSG00000135333.13	Lung	C=0.958	A=0.042	0.702179	1.18E-05
rs16880170	rs199888991	chr6:93784078	0.00095171	1	EPHA7	ENSG00000135333.13	Adipose - Subcutaneous	-=0.995	T=0.005	-0.796053	1.44E-05
rs16880170	rs111429740	chr6:93838453	0.01668447	1	EPHA7	ENSG00000135333.13	Lung	G=0.919	A=0.081	0.383525	1.55E-05
rs16880170	rs16870789	chr6:93827963	0.01646069	1	EPHA7	ENSG00000135333.13	Lung	C=0.92	T=0.08	0.374368	1.81E-05
rs16880170	rs145810085	chr6:93833819	0.00152731	1	EPHA7	ENSG00000135333.13	Adipose - Subcutaneous	G=0.992	A=0.008	-0.735109	1.88E-05
rs16880170	rs7774823	chr6:93830545	0.08057883	1	EPHA7	ENSG00000135333.13	Minor Salivary Gland	G=0.703	A=0.297	-0.232136	1.89E-05
rs16880170	rs1951907	chr6:93831192	0.08057883	1	EPHA7	ENSG00000135333.13	Minor Salivary Gland	T=0.703	C=0.297	-0.232136	1.89E-05
rs16880170	rs35306488	chr6:93832227	0.07943329	1	EPHA7	ENSG00000135333.13	Minor Salivary Gland	-=0.706	T=0.294	-0.232136	1.89E-05
rs16880170	rs10944648	chr6:93832280	0.08057883	1	EPHA7	ENSG00000135333.13	Minor Salivary Gland	A=0.703	G=0.297	-0.232136	1.89E-05
rs16880170	rs7751375	chr6:93832923	0.08096284	1	EPHA7	ENSG00000135333.13	Minor Salivary Gland	C=0.702	A=0.298	-0.232136	1.89E-05
rs16880170	rs7757292	chr6:93834165	0.08057883	1	EPHA7	ENSG00000135333.13	Minor Salivary Gland	C=0.703	A=0.297	-0.232136	1.89E-05
rs16880170	rs16870850	chr6:93841079	0.01758444	1	EPHA7	ENSG00000135333.13	Lung	T=0.916	C=0.084	0.361979	2.02E-05
rs16880170	rs12528846	chr6:93828842	0.01646069	1	EPHA7	ENSG00000135333.13	Lung	A=0.92	G=0.08	0.377147	2.03E-05
rs16880170	rs12527296	chr6:93829143	0.01646069	1	EPHA7	ENSG00000135333.13	Lung	T=0.92	C=0.08	0.377147	2.03E-05
rs16880170	rs72928575	chr6:93829399	0.01668447	1	EPHA7	ENSG00000135333.13	Lung	G=0.919	T=0.081	0.377147	2.03E-05
rs16880170	rs72928579	chr6:93829658	0.01646069	1	EPHA7	ENSG00000135333.13	Lung	G=0.92	A=0.08	0.377147	2.03E-05
rs16880170	rs77007574	chr6:93829960	0.01646069	1	EPHA7	ENSG00000135333.13	Lung	T=0.92	C=0.08	0.377147	2.03E-05
rs16880170	rs72928582	chr6:93830015	0.01646069	1	EPHA7	ENSG00000135333.13	Lung	G=0.92	A=0.08	0.377147	2.03E-05
rs16880170	rs16870801	chr6:93830951	0.01646069	1	EPHA7	ENSG00000135333.13	Lung	T=0.92	C=0.08	0.377147	2.03E-05
rs16880170	rs16870805	chr6:93834075	0.01646069	1	EPHA7	ENSG00000135333.13	Lung	G=0.92	T=0.08	0.377147	2.03E-05
rs16880170	rs150304721	chr6:93835801	0.01690873	1	EPHA7	ENSG00000135333.13	Lung	A=0.918	-=0.082	0.361316	2.04E-05
rs16880170	rs16870855	chr6:93841440	0.01668447	1	EPHA7	ENSG00000135333.13	Lung	C=0.919	T=0.081	0.4288	2.14E-05
rs16880170	rs7772178	chr6:93849608	0.02531851	1	EPHA7	ENSG00000135333.13	Testis	C=0.883	T=0.117	0.425527	2.47E-05
rs16880170	rs73758259	chr6:93828724	0.01713348	1	EPHA7	ENSG00000135333.13	Lung	G=0.917	T=0.083	0.354112	2.72E-05
rs16880170	rs16870796	chr6:93828781	0.01646069	1	EPHA7	ENSG00000135333.13	Lung	T=0.92	G=0.08	0.369069	2.76E-05
rs16880170	rs72928577	chr6:93829400	0.01668447	1	EPHA7	ENSG00000135333.13	Lung	C=0.919	T=0.081	0.369069	2.76E-05
rs16880170	rs117717826	chr6:94036945	0.00485557	1	EPHA7	ENSG00000135333.13	Heart - Left Ventricle	T=0.975	A=0.025	-0.389622	2.96E-05
rs16880170	rs71558449	chr6:93828589	0.00646205	1	EPHA7	ENSG00000135333.13	Esophagus - Muscularis	G=0.967	A=0.033	0.272458	3.14E-05
rs16880170	rs6922405	chr6:93842847	0.01668447	1	EPHA7	ENSG00000135333.13	Lung	T=0.919	C=0.081	0.370721	3.25E-05
rs16880170	rs72930416	chr6:93844150	0.01668447	1	EPHA7	ENSG00000135333.13	Lung	T=0.919	C=0.081	0.370721	3.25E-05
rs16880170	rs16870846	chr6:93840909	0.01668447	1	EPHA7	ENSG00000135333.13	Lung	C=0.919	G=0.081	0.371767	3.40E-05
rs16880170	rs1319460	chr6:93846637	0.05969688	1	EPHA7	ENSG00000135333.13	Minor Salivary Gland	C=0.239	T=0.761	0.230876	3.54E-05
rs16880170	rs538751	chr6:93848400	0.05969688	1	EPHA7	ENSG00000135333.13	Minor Salivary Gland	A=0.239	C=0.761	0.230876	3.54E-05

rs16880170	rs11422146	chr6:93849652	0.05937064	1	EPHA7	ENSG00000135333.13	Minor Salivary Gland	-=0.238	T=0.762	0.230876	3.54E-05
rs16880170	rs1319460	chr6:93846637	0.05969688	1	EPHA7	ENSG00000135333.13	Testis	C=0.239	T=0.761	-0.330556	3.66E-05
rs16880170	rs538751	chr6:93848400	0.05969688	1	EPHA7	ENSG00000135333.13	Testis	A=0.239	C=0.761	-0.330556	3.66E-05
rs16880170	rs11422146	chr6:93849652	0.05937064	1	EPHA7	ENSG00000135333.13	Testis	-=0.238	T=0.762	-0.330556	3.66E-05
rs16880170	rs140182812	chr6:93804212	0.00152731	1	EPHA7	ENSG00000135333.13	Adipose - Subcutaneous	G=0.992	A=0.008	-0.729088	4.39E-05
rs16880170	rs41273625	chr6:93952851	0.01032934	0.9008183	EPHA7	ENSG00000135333.13	Esophagus - Muscularis	C=0.937	G=0.063	0.224035	5.17E-05
rs16880170	rs12530331	chr6:93830460	0.01668447	1	EPHA7	ENSG00000135333.13	Lung	G=0.919	C=0.081	0.35653	5.33E-05
rs16880170	rs74468395	chr6:94017659	0.00646205	1	EPHA7	ENSG00000135333.13	Heart - Left Ventricle	C=0.967	T=0.033	-0.353	6.19E-05
rs16880170	rs117256127	chr6:93852714	0.0046566	1	EPHA7	ENSG00000135333.13	Adipose - Subcutaneous	T=0.976	C=0.024	-0.674139	6.42E-05
rs16880170	rs117425371	chr6:93852717	0.0046566	1	EPHA7	ENSG00000135333.13	Adipose - Subcutaneous	A=0.976	G=0.024	-0.674139	6.42E-05
rs16880170	rs145519743	chr6:93884032	0.00485557	1	EPHA7	ENSG00000135333.13	Adipose - Subcutaneous	G=0.975	A=0.025	-0.674139	6.42E-05
rs16880170	rs9351349	chr6:93832104	0.0386878	0.9652864	EPHA7	ENSG00000135333.13	Adipose - Subcutaneous	G=0.179	A=0.821	0.228214	6.48E-05
rs16880170	rs147330775	chr6:93915683	0.0011432	1	EPHA7	ENSG00000135333.13	Skin - Sun Exposed (Lower leg)	C=0.994	T=0.006	-0.47159	6.56E-05
rs16880170	rs117109139	chr6:93861398	0.00747958	1	EPHA7	ENSG00000135333.13	Esophagus - Muscularis	C=0.962	G=0.038	0.257424	7.15E-05
rs16880170	rs650711	chr6:93842280	0.0386878	0.9652864	EPHA7	ENSG00000135333.13	Adipose - Subcutaneous	T=0.821	G=0.179	-0.226702	7.44E-05
rs16880170	rs768382	chr6:93826168	0.03841091	0.96509247	EPHA7	ENSG00000135333.13	Adipose - Subcutaneous	T=0.178	C=0.822	0.223191	7.87E-05
rs16880170	rs1324110	chr6:93913200	0.15736501	0.80128613	EPHA7	ENSG00000135333.13	Heart - Atrial Appendage	G=0.563	C=0.437	0.0819979	9.78E-05
rs16880170	rs41273625	chr6:93952851	0.01032934	0.9008183	EPHA7	ENSG00000135333.13	Esophagus - Gastroesophageal Junction	C=0.937	G=0.063	0.249826	0.00010545
rs16880170	rs138022068	chr6:94069111	0.00722283	0.86982402	EPHA7	ENSG00000135333.13	Heart - Left Ventricle	C=0.952	-=0.048	-0.300501	0.00011172
rs16880170	rs192604147	chr6:94240629	0.00171995	1	EPHA7	ENSG00000135333.13	Heart - Left Ventricle	A=0.991	G=0.009	-0.514265	0.00011598
rs16880170	rs143801031	chr6:93750441	0.00327508	1	EPHA7	ENSG00000135333.13	Adipose - Subcutaneous	T=0.983	C=0.017	-0.536087	0.00015072
rs16880170	rs117365486	chr6:93820610	0.0062598	1	EPHA7	ENSG00000135333.13	Nerve - Tibial	C=0.968	T=0.032	-0.404506	0.000162
rs16880170	rs148086058	chr6:93820887	0.0062598	1	EPHA7	ENSG00000135333.13	Nerve - Tibial	C=0.968	A=0.032	-0.404506	0.000162
rs16880170	rs182357880	chr6:93879803	0.00133506	1	EPHA7	ENSG00000135333.13	Skin - Sun Exposed (Lower leg)	T=0.993	C=0.007	-0.395624	0.00022421
rs17540607	rs117365486	chr6:93820610	0.00616744	1	EPHA7	ENSG00000135333.13	Adipose - Subcutaneous	C=0.968	T=0.032	-0.679264	8.33E-10
rs17540607	rs148086058	chr6:93820887	0.00616744	1	EPHA7	ENSG00000135333.13	Adipose - Subcutaneous	C=0.968	A=0.032	-0.679264	8.33E-10
rs17540607	rs189136709	chr6:93783798	0.00093767	1	EPHA7	ENSG00000135333.13	Brain - Anterior cingulate cortex (BA24)	G=0.995	A=0.005	1.35876	1.60E-08
rs17540607	rs1386276	chr6:94082749	0.00454121	0.81922731	EPHA7	ENSG00000135333.13	Heart - Left Ventricle	A=0.965	G=0.035	-0.463277	1.18E-07
rs17540607	rs79718749	chr6:94098832	0.00454121	0.81922731	EPHA7	ENSG00000135333.13	Heart - Left Ventricle	T=0.965	C=0.035	-0.463277	1.18E-07
rs17540607	rs12661215	chr6:94099119	0.00454121	0.81922731	EPHA7	ENSG00000135333.13	Heart - Left Ventricle	C=0.965	T=0.035	-0.463277	1.18E-07
rs17540607	rs16871305	chr6:94111173	0.00454121	0.81922731	EPHA7	ENSG00000135333.13	Heart - Left Ventricle	A=0.965	T=0.035	-0.463277	1.18E-07
rs17540607	rs80300952	chr6:94115209	0.00454121	0.81922731	EPHA7	ENSG00000135333.13	Heart - Left Ventricle	T=0.965	C=0.035	-0.463277	1.18E-07
rs17540607	rs146471836	chr6:93817042	0.00396969	0.80227987	EPHA7	ENSG00000135333.13	Adipose - Subcutaneous	C=0.968	T=0.032	-0.617165	8.35E-07
rs17540607	rs141174921	chr6:93733454	0.00396969	0.80227987	EPHA7	ENSG00000135333.13	Adipose - Subcutaneous	A=0.968	G=0.032	-0.598481	1.67E-06
rs17540607	rs117376030	chr6:94203683	0.00498036	1	EPHA7	ENSG00000135333.13	Esophagus - Gastroesophageal Junction	T=0.974	A=0.026	-0.384156	2.51E-06
rs17540607	rs72928597	chr6:93839843	0.0164383	1	EPHA7	ENSG00000135333.13	Lung	A=0.919	G=0.081	0.42955	4.55E-06
rs17540607	rs16870853	chr6:93841320	0.0164383	1	EPHA7	ENSG00000135333.13	Lung	T=0.919	C=0.081	0.42955	4.55E-06
rs17540607	rs1930933	chr6:93842544	0.08014771	1	EPHA7	ENSG00000135333.13	Minor Salivary Gland	C=0.299	T=0.701	0.246092	4.56E-06
rs17540607	rs142723671	chr6:94076414	0.00419702	1	EPHA7	ENSG00000135333.13	Heart - Left Ventricle	G=0.978	C=0.022	-0.495017	4.73E-06
rs17540607	rs147330775	chr6:93915683	0.00112633	1	EPHA7	ENSG00000135333.13	Brain - Anterior cingulate cortex (BA24)	C=0.994	T=0.006	0.808887	6.34E-06
rs17540607	rs16870793	chr6:93828599	0.01599783	1	EPHA7	ENSG00000135333.13	Lung	G=0.921	A=0.079	0.419095	6.62E-06
rs17540607	rs72928592	chr6:93837959	0.01621783	1	EPHA7	ENSG00000135333.13	Lung	T=0.92	C=0.08	0.419095	6.62E-06
rs17540607	rs1575540	chr6:93836577	0.01148506	1	EPHA7	ENSG00000135333.13	Esophagus - Muscularis	G=0.942	A=0.058	0.272463	7.68E-06
rs17540607	rs544639807	chr6:93883657	0.00150478	1	EPHA7	ENSG00000135333.13	Minor Salivary Gland	G=0.992	A=0.008	-1.34812	8.25E-06
rs17540607	rs36114673	chr6:93847056	0.01232916	1	EPHA7	ENSG00000135333.13	Esophagus - Muscularis	A=0.938	T=0.062	0.268662	8.26E-06
rs17540607	rs113888577	chr6:93698093	0.00226625	1	EPHA7	ENSG00000135333.13	Brain - Anterior cingulate cortex (BA24)	C=0.988	T=0.012	0.66365	8.36E-06
rs17540607	rs16870780	chr6:93826505	0.01621783	1	EPHA7	ENSG00000135333.13	Lung	T=0.92	A=0.08	0.385133	9.62E-06
rs17540607	rs1590384	chr6:93837434	0.08014771	1	EPHA7	ENSG00000135333.13	Minor Salivary Gland	C=0.701	G=0.299	-0.24131	1.00E-05
rs17540607	rs1924474	chr6:93837991	0.08014771	1	EPHA7	ENSG00000135333.13	Minor Salivary Gland	C=0.701	T=0.299	-0.24131	1.00E-05
rs17540607	rs10944652	chr6:93838348	0.08014771	1	EPHA7	ENSG00000135333.13	Minor Salivary Gland	C=0.701	G=0.299	-0.24131	1.00E-05
rs17540607	rs12204186	chr6:93838665	0.08014771	1	EPHA7	ENSG00000135333.13	Minor Salivary Gland	C=0.701	T=0.299	-0.24131	1.00E-05

rs17540607	rs633279	chr6:93840705	0.08014771	1	EPHA7	ENSG00000135333.13	Minor Salivary Gland	A=0.299	T=0.701	0.24131	1.00E-05
rs17540607	rs634060	chr6:93840849	0.08014771	1	EPHA7	ENSG00000135333.13	Minor Salivary Gland	A=0.299	G=0.701	0.24131	1.00E-05
rs17540607	rs568957	chr6:93840855	0.08014771	1	EPHA7	ENSG00000135333.13	Minor Salivary Gland	G=0.299	T=0.701	0.24131	1.00E-05
rs17540607	rs34544163	chr6:93841565	0.08014771	1	EPHA7	ENSG00000135333.13	Minor Salivary Gland	-=0.299	A=0.701	0.24131	1.00E-05
rs17540607	rs562379	chr6:93841592	0.08014771	1	EPHA7	ENSG00000135333.13	Minor Salivary Gland	G=0.299	A=0.701	0.24131	1.00E-05
rs17540607	rs560731	chr6:93841734	0.08014771	1	EPHA7	ENSG00000135333.13	Minor Salivary Gland	A=0.299	G=0.701	0.24131	1.00E-05
rs17540607	rs650642	chr6:93842232	0.08014771	1	EPHA7	ENSG00000135333.13	Minor Salivary Gland	G=0.299	T=0.701	0.24131	1.00E-05
rs17540607	rs650747	chr6:93842298	0.08014771	1	EPHA7	ENSG00000135333.13	Minor Salivary Gland	T=0.299	C=0.701	0.24131	1.00E-05
rs17540607	rs1930934	chr6:93842533	0.08014771	1	EPHA7	ENSG00000135333.13	Minor Salivary Gland	A=0.299	G=0.701	0.24131	1.00E-05
rs17540607	rs9353987	chr6:93843588	0.08014771	1	EPHA7	ENSG00000135333.13	Minor Salivary Gland	A=0.299	G=0.701	0.24131	1.00E-05
rs17540607	rs9363034	chr6:93844136	0.08014771	1	EPHA7	ENSG00000135333.13	Minor Salivary Gland	G=0.299	A=0.701	0.24131	1.00E-05
rs17540607	rs9353989	chr6:93844383	0.08014771	1	EPHA7	ENSG00000135333.13	Minor Salivary Gland	G=0.299	T=0.701	0.24131	1.00E-05
rs17540607	rs76282783	chr6:93576050	0.00590018	0.84935609	EPHA7	ENSG00000135333.13	Lung	C=0.958	A=0.042	0.702179	1.18E-05
rs17540607	rs199888991	chr6:93784078	0.00093767	1	EPHA7	ENSG00000135333.13	Adipose - Subcutaneous	-=0.995	T=0.005	-0.796053	1.44E-05
rs17540607	rs111429740	chr6:93838453	0.0164383	1	EPHA7	ENSG00000135333.13	Lung	G=0.919	A=0.081	0.383525	1.55E-05
rs17540607	rs16870789	chr6:93827963	0.01621783	1	EPHA7	ENSG00000135333.13	Lung	C=0.92	T=0.08	0.374368	1.81E-05
rs17540607	rs145810085	chr6:93833819	0.00150478	1	EPHA7	ENSG00000135333.13	Adipose - Subcutaneous	G=0.992	A=0.008	-0.735109	1.88E-05
rs17540607	rs7774823	chr6:93830545	0.07938994	1	EPHA7	ENSG00000135333.13	Minor Salivary Gland	G=0.703	A=0.297	-0.232136	1.89E-05
rs17540607	rs1951907	chr6:93831192	0.07938994	1	EPHA7	ENSG00000135333.13	Minor Salivary Gland	T=0.703	C=0.297	-0.232136	1.89E-05
rs17540607	rs35306488	chr6:93832227	0.0782613	1	EPHA7	ENSG00000135333.13	Minor Salivary Gland	-=0.706	T=0.294	-0.232136	1.89E-05
rs17540607	rs10944648	chr6:93832280	0.07938994	1	EPHA7	ENSG00000135333.13	Minor Salivary Gland	A=0.703	G=0.297	-0.232136	1.89E-05
rs17540607	rs7751375	chr6:93832923	0.07976829	1	EPHA7	ENSG00000135333.13	Minor Salivary Gland	C=0.702	A=0.298	-0.232136	1.89E-05
rs17540607	rs7757292	chr6:93834165	0.07938994	1	EPHA7	ENSG00000135333.13	Minor Salivary Gland	C=0.703	A=0.297	-0.232136	1.89E-05
rs17540607	rs16870850	chr6:93841079	0.01732499	1	EPHA7	ENSG00000135333.13	Lung	T=0.916	C=0.084	0.361979	2.02E-05
rs17540607	rs12528846	chr6:93828842	0.01621783	1	EPHA7	ENSG00000135333.13	Lung	A=0.92	G=0.08	0.377147	2.03E-05
rs17540607	rs12527296	chr6:93829143	0.01621783	1	EPHA7	ENSG00000135333.13	Lung	T=0.92	C=0.08	0.377147	2.03E-05
rs17540607	rs72928575	chr6:93829399	0.0164383	1	EPHA7	ENSG00000135333.13	Lung	G=0.919	T=0.081	0.377147	2.03E-05
rs17540607	rs72928579	chr6:93829658	0.01621783	1	EPHA7	ENSG00000135333.13	Lung	G=0.92	A=0.08	0.377147	2.03E-05
rs17540607	rs77007574	chr6:93829960	0.01621783	1	EPHA7	ENSG00000135333.13	Lung	T=0.92	C=0.08	0.377147	2.03E-05
rs17540607	rs72928582	chr6:93830015	0.01621783	1	EPHA7	ENSG00000135333.13	Lung	G=0.92	A=0.08	0.377147	2.03E-05
rs17540607	rs16870801	chr6:93830951	0.01621783	1	EPHA7	ENSG00000135333.13	Lung	T=0.92	C=0.08	0.377147	2.03E-05
rs17540607	rs16870805	chr6:93834075	0.01621783	1	EPHA7	ENSG00000135333.13	Lung	G=0.92	T=0.08	0.377147	2.03E-05
rs17540607	rs150304721	chr6:93835801	0.01665926	1	EPHA7	ENSG00000135333.13	Lung	A=0.918	-=0.082	0.361316	2.04E-05
rs17540607	rs16870855	chr6:93841440	0.0164383	1	EPHA7	ENSG00000135333.13	Lung	C=0.919	T=0.081	0.4288	2.14E-05
rs17540607	rs7772178	chr6:93849608	0.02494496	1	EPHA7	ENSG00000135333.13	Testis	C=0.883	T=0.117	0.425527	2.47E-05
rs17540607	rs73758259	chr6:93828724	0.01688069	1	EPHA7	ENSG00000135333.13	Lung	G=0.917	T=0.083	0.354112	2.72E-05
rs17540607	rs16870796	chr6:93828781	0.01621783	1	EPHA7	ENSG00000135333.13	Lung	T=0.92	G=0.08	0.369069	2.76E-05
rs17540607	rs72928577	chr6:93829400	0.0164383	1	EPHA7	ENSG00000135333.13	Lung	C=0.919	T=0.081	0.369069	2.76E-05
rs17540607	rs117717826	chr6:94036945	0.00478393	1	EPHA7	ENSG00000135333.13	Heart - Left Ventricle	T=0.975	A=0.025	-0.389622	2.96E-05
rs17540607	rs71558449	chr6:93828589	0.00636671	1	EPHA7	ENSG00000135333.13	Esophagus - Muscularis	G=0.967	A=0.033	0.272458	3.14E-05
rs17540607	rs6922405	chr6:93842847	0.0164383	1	EPHA7	ENSG00000135333.13	Lung	T=0.919	C=0.081	0.370721	3.25E-05
rs17540607	rs72930416	chr6:93844150	0.0164383	1	EPHA7	ENSG00000135333.13	Lung	T=0.919	C=0.081	0.370721	3.25E-05
rs17540607	rs16870846	chr6:93840909	0.0164383	1	EPHA7	ENSG00000135333.13	Lung	C=0.919	G=0.081	0.371767	3.40E-05
rs17540607	rs1319460	chr6:93846637	0.05881609	1	EPHA7	ENSG00000135333.13	Minor Salivary Gland	C=0.239	T=0.761	0.230876	3.54E-05
rs17540607	rs538751	chr6:93848400	0.05881609	1	EPHA7	ENSG00000135333.13	Minor Salivary Gland	A=0.239	C=0.761	0.230876	3.54E-05
rs17540607	rs11422146	chr6:93849652	0.05849466	1	EPHA7	ENSG00000135333.13	Minor Salivary Gland	-=0.238	T=0.762	0.230876	3.54E-05
rs17540607	rs1319460	chr6:93846637	0.05881609	1	EPHA7	ENSG00000135333.13	Testis	C=0.239	T=0.761	-0.330556	3.66E-05
rs17540607	rs538751	chr6:93848400	0.05881609	1	EPHA7	ENSG00000135333.13	Testis	A=0.239	C=0.761	-0.330556	3.66E-05
rs17540607	rs11422146	chr6:93849652	0.05849466	1	EPHA7	ENSG00000135333.13	Testis	-=0.238	T=0.762	-0.330556	3.66E-05
rs17540607	rs140182812	chr6:93804212	0.00150478	1	EPHA7	ENSG00000135333.13	Adipose - Subcutaneous	G=0.992	A=0.008	-0.729088	4.39E-05
rs17540607	rs41273625	chr6:93952851	0.01014877	0.89957073	EPHA7	ENSG00000135333.13	Esophagus - Muscularis	C=0.937	G=0.063	0.224035	5.17E-05

rs17540607	rs12530331	chr6:93830460	0.0164383	1	EPHA7	ENSG00000135333.13	Lung	G=0.919	C=0.081	0.35653	5.33E-05
rs17540607	rs74468395	chr6:94017659	0.00636671	1	EPHA7	ENSG00000135333.13	Heart - Left Ventricle	C=0.967	T=0.033	-0.353	6.19E-05
rs17540607	rs117256127	chr6:93852714	0.00458789	1	EPHA7	ENSG00000135333.13	Adipose - Subcutaneous	T=0.976	C=0.024	-0.674139	6.42E-05
rs17540607	rs117425371	chr6:93852717	0.00458789	1	EPHA7	ENSG00000135333.13	Adipose - Subcutaneous	A=0.976	G=0.024	-0.674139	6.42E-05
rs17540607	rs145519743	chr6:93884032	0.00478393	1	EPHA7	ENSG00000135333.13	Adipose - Subcutaneous	G=0.975	A=0.025	-0.674139	6.42E-05
rs17540607	rs9351349	chr6:93832104	0.0409078	1	EPHA7	ENSG00000135333.13	Adipose - Subcutaneous	G=0.179	A=0.821	0.228214	6.48E-05
rs17540607	rs147330775	chr6:93915683	0.00112633	1	EPHA7	ENSG00000135333.13	Skin - Sun Exposed (Lower leg)	C=0.994	T=0.006	-0.47159	6.56E-05
rs17540607	rs117109139	chr6:93861398	0.00736923	1	EPHA7	ENSG00000135333.13	Esophagus - Muscularis	C=0.962	G=0.038	0.257424	7.15E-05
rs17540607	rs650711	chr6:93842280	0.0409078	1	EPHA7	ENSG00000135333.13	Adipose - Subcutaneous	T=0.821	G=0.179	-0.226702	7.44E-05
rs17540607	rs768382	chr6:93826168	0.04063135	1	EPHA7	ENSG00000135333.13	Adipose - Subcutaneous	T=0.178	C=0.822	0.223191	7.87E-05
rs17540607	rs1324110	chr6:93913200	0.15842003	0.80996511	EPHA7	ENSG00000135333.13	Heart - Atrial Appendage	G=0.563	C=0.437	0.0819979	9.78E-05
rs17540607	rs41273625	chr6:93952851	0.01014877	0.89957073	EPHA7	ENSG00000135333.13	Esophagus - Gastroesophageal Junction	C=0.937	G=0.063	0.249826	0.00010545
rs17540607	rs138022068	chr6:94069111	0.0070895	0.86818658	EPHA7	ENSG00000135333.13	Heart - Left Ventricle	C=0.952	-=0.048	-0.300501	0.00011172
rs17540607	rs192604147	chr6:94240629	0.00169458	1	EPHA7	ENSG00000135333.13	Heart - Left Ventricle	A=0.991	G=0.009	-0.514265	0.00011598
rs17540607	rs143801031	chr6:93750441	0.00322676	1	EPHA7	ENSG00000135333.13	Adipose - Subcutaneous	T=0.983	C=0.017	-0.536087	0.00015072
rs17540607	rs117365486	chr6:93820610	0.00616744	1	EPHA7	ENSG00000135333.13	Nerve - Tibial	C=0.968	T=0.032	-0.404506	0.000162
rs17540607	rs148086058	chr6:93820887	0.00616744	1	EPHA7	ENSG00000135333.13	Nerve - Tibial	C=0.968	A=0.032	-0.404506	0.000162
rs17540607	rs182357880	chr6:93879803	0.00131536	1	EPHA7	ENSG00000135333.13	Skin - Sun Exposed (Lower leg)	T=0.993	C=0.007	-0.395624	0.00022421
rs6927004	rs1535833	chr6:93769380	0.04367934	0.80709492	EPHA7	ENSG00000135333.13	Adipose - Subcutaneous	C=0.722	T=0.278	-0.770455	1.90E-63
rs6927004	rs58205228	chr6:93778613	0.0454394	0.81114188	EPHA7	ENSG00000135333.13	Adipose - Subcutaneous	A=0.716	G=0.284	-0.767837	2.77E-63
rs6927004	rs9345321	chr6:93789199	0.0454394	0.81114188	EPHA7	ENSG00000135333.13	Adipose - Subcutaneous	T=0.716	G=0.284	-0.767837	2.77E-63
rs6927004	rs9345322	chr6:93790565	0.0454394	0.81114188	EPHA7	ENSG00000135333.13	Adipose - Subcutaneous	A=0.716	G=0.284	-0.767837	2.77E-63
rs6927004	rs9345323	chr6:93797607	0.0454394	0.81114188	EPHA7	ENSG00000135333.13	Adipose - Subcutaneous	C=0.716	T=0.284	-0.767837	2.77E-63
rs6927004	rs9342350	chr6:93798264	0.0454394	0.81114188	EPHA7	ENSG00000135333.13	Adipose - Subcutaneous	A=0.716	G=0.284	-0.767837	2.77E-63
rs6927004	rs9363029	chr6:93798520	0.0454394	0.81114188	EPHA7	ENSG00000135333.13	Adipose - Subcutaneous	T=0.716	G=0.284	-0.767837	2.77E-63
rs6927004	rs9353982	chr6:93802038	0.0454394	0.81114188	EPHA7	ENSG00000135333.13	Adipose - Subcutaneous	G=0.716	A=0.284	-0.767837	2.77E-63
rs6927004	rs9345324	chr6:93803037	0.0454394	0.81114188	EPHA7	ENSG00000135333.13	Adipose - Subcutaneous	C=0.716	A=0.284	-0.767837	2.77E-63
rs6927004	rs1570631	chr6:93811876	0.04455544	0.80913985	EPHA7	ENSG00000135333.13	Adipose - Subcutaneous	T=0.719	C=0.281	-0.759574	1.45E-59
rs6927004	rs77898219	chr6:93824234	0.04367934	0.80709492	EPHA7	ENSG00000135333.13	Adipose - Subcutaneous	C=0.722	-=0.278	-0.752617	1.58E-59
rs6927004	rs9363030	chr6:93806040	0.04397051	0.80778141	EPHA7	ENSG00000135333.13	Adipose - Subcutaneous	G=0.721	A=0.279	-0.757344	1.59E-59
rs6927004	rs9363030	chr6:93806040	0.04397051	0.80778141	EPHA7	ENSG00000135333.13	Lung	G=0.721	A=0.279	-0.467048	1.04E-18
rs6927004	rs1570631	chr6:93811876	0.04455544	0.80913985	EPHA7	ENSG00000135333.13	Lung	T=0.719	C=0.281	-0.467048	1.04E-18
rs6927004	rs58205228	chr6:93778613	0.0454394	0.81114188	EPHA7	ENSG00000135333.13	Lung	A=0.716	G=0.284	-0.459589	1.54E-18
rs6927004	rs9345321	chr6:93789199	0.0454394	0.81114188	EPHA7	ENSG00000135333.13	Lung	T=0.716	G=0.284	-0.459589	1.54E-18
rs6927004	rs9345322	chr6:93790565	0.0454394	0.81114188	EPHA7	ENSG00000135333.13	Lung	A=0.716	G=0.284	-0.459589	1.54E-18
rs6927004	rs9345323	chr6:93797607	0.0454394	0.81114188	EPHA7	ENSG00000135333.13	Lung	C=0.716	T=0.284	-0.459589	1.54E-18
rs6927004	rs9342350	chr6:93798264	0.0454394	0.81114188	EPHA7	ENSG00000135333.13	Lung	A=0.716	G=0.284	-0.459589	1.54E-18
rs6927004	rs9363029	chr6:93798520	0.0454394	0.81114188	EPHA7	ENSG00000135333.13	Lung	T=0.716	G=0.284	-0.459589	1.54E-18
rs6927004	rs9353982	chr6:93802038	0.0454394	0.81114188	EPHA7	ENSG00000135333.13	Lung	G=0.716	A=0.284	-0.459589	1.54E-18
rs6927004	rs9345324	chr6:93803037	0.0454394	0.81114188	EPHA7	ENSG00000135333.13	Lung	C=0.716	A=0.284	-0.459589	1.54E-18
rs6927004	rs77898219	chr6:93824234	0.04367934	0.80709492	EPHA7	ENSG00000135333.13	Lung	C=0.722	-=0.278	-0.456134	5.88E-18
rs6927004	rs1535833	chr6:93769380	0.04367934	0.80709492	EPHA7	ENSG00000135333.13	Lung	C=0.722	T=0.278	-0.44499	2.25E-17
rs6927004	rs9363030	chr6:93806040	0.04397051	0.80778141	EPHA7	ENSG00000135333.13	Skin - Sun Exposed (Lower leg)	G=0.721	A=0.279	-0.281476	2.71E-17
rs6927004	rs9353982	chr6:93802038	0.0454394	0.81114188	EPHA7	ENSG00000135333.13	Skin - Sun Exposed (Lower leg)	G=0.716	A=0.284	-0.276454	4.84E-17
rs6927004	rs1570631	chr6:93811876	0.04455544	0.80913985	EPHA7	ENSG00000135333.13	Skin - Sun Exposed (Lower leg)	T=0.719	C=0.281	-0.279518	5.05E-17
rs6927004	rs58205228	chr6:93778613	0.0454394	0.81114188	EPHA7	ENSG00000135333.13	Skin - Sun Exposed (Lower leg)	A=0.716	G=0.284	-0.274662	6.63E-17
rs6927004	rs9345323	chr6:93797607	0.0454394	0.81114188	EPHA7	ENSG00000135333.13	Skin - Sun Exposed (Lower leg)	C=0.716	T=0.284	-0.274662	6.63E-17
rs6927004	rs9342350	chr6:93798264	0.0454394	0.81114188	EPHA7	ENSG00000135333.13	Skin - Sun Exposed (Lower leg)	A=0.716	G=0.284	-0.274662	6.63E-17
rs6927004	rs9363029	chr6:93798520	0.0454394	0.81114188	EPHA7	ENSG00000135333.13	Skin - Sun Exposed (Lower leg)	T=0.716	G=0.284	-0.274662	6.63E-17
rs6927004	rs9345324	chr6:93803037	0.0454394	0.81114188	EPHA7	ENSG00000135333.13	Skin - Sun Exposed (Lower leg)	C=0.716	A=0.284	-0.274662	6.63E-17
rs6927004	rs9345321	chr6:93789199	0.0454394	0.81114188	EPHA7	ENSG00000135333.13	Skin - Sun Exposed (Lower leg)	T=0.716	G=0.284	-0.273298	8.03E-17

rs6927004	rs9345322	chr6:93790565	0.0454394	0.81114188	EPHA7	ENSG00000135333.13	Skin - Sun Exposed (Lower leg)	A=0.716	G=0.284	-0.273298	8.03E-17
rs6927004	rs1535833	chr6:93769380	0.04367934	0.80709492	EPHA7	ENSG00000135333.13	Skin - Sun Exposed (Lower leg)	C=0.722	T=0.278	-0.274816	9.27E-17
rs6927004	rs77898219	chr6:93824234	0.04367934	0.80709492	EPHA7	ENSG00000135333.13	Skin - Sun Exposed (Lower leg)	C=0.722	-=0.278	-0.274384	1.06E-16
rs6927004	rs9363030	chr6:93806040	0.04397051	0.80778141	EPHA7	ENSG00000135333.13	Skin - Not Sun Exposed (Suprapubic)	G=0.721	A=0.279	-0.26209	8.68E-15
rs6927004	rs77898219	chr6:93824234	0.04367934	0.80709492	EPHA7	ENSG00000135333.13	Skin - Not Sun Exposed (Suprapubic)	C=0.722	-=0.278	-0.258658	1.10E-14
rs6927004	rs1570631	chr6:93811876	0.04455544	0.80913985	EPHA7	ENSG00000135333.13	Skin - Not Sun Exposed (Suprapubic)	T=0.719	C=0.281	-0.259435	1.84E-14
rs6927004	rs1535833	chr6:93769380	0.04367934	0.80709492	EPHA7	ENSG00000135333.13	Skin - Not Sun Exposed (Suprapubic)	C=0.722	T=0.278	-0.258257	2.03E-14
rs6927004	rs9342350	chr6:93798264	0.0454394	0.81114188	EPHA7	ENSG00000135333.13	Skin - Not Sun Exposed (Suprapubic)	A=0.716	G=0.284	-0.256364	2.36E-14
rs6927004	rs9363029	chr6:93798520	0.0454394	0.81114188	EPHA7	ENSG00000135333.13	Skin - Not Sun Exposed (Suprapubic)	T=0.716	G=0.284	-0.256364	2.36E-14
rs6927004	rs9353982	chr6:93802038	0.0454394	0.81114188	EPHA7	ENSG00000135333.13	Skin - Not Sun Exposed (Suprapubic)	G=0.716	A=0.284	-0.256364	2.36E-14
rs6927004	rs9345324	chr6:93803037	0.0454394	0.81114188	EPHA7	ENSG00000135333.13	Skin - Not Sun Exposed (Suprapubic)	C=0.716	A=0.284	-0.256364	2.36E-14
rs6927004	rs9345323	chr6:93797607	0.0454394	0.81114188	EPHA7	ENSG00000135333.13	Skin - Not Sun Exposed (Suprapubic)	C=0.716	T=0.284	-0.253851	3.29E-14
rs6927004	rs58205228	chr6:93778613	0.0454394	0.81114188	EPHA7	ENSG00000135333.13	Skin - Not Sun Exposed (Suprapubic)	A=0.716	G=0.284	-0.252522	5.58E-14
rs6927004	rs9345321	chr6:93789199	0.0454394	0.81114188	EPHA7	ENSG00000135333.13	Skin - Not Sun Exposed (Suprapubic)	T=0.716	G=0.284	-0.252522	5.58E-14
rs6927004	rs9345322	chr6:93790565	0.0454394	0.81114188	EPHA7	ENSG00000135333.13	Skin - Not Sun Exposed (Suprapubic)	A=0.716	G=0.284	-0.252522	5.58E-14
rs6927004	rs9353982	chr6:93802038	0.0454394	0.81114188	EPHA7	ENSG00000135333.13	Breast - Mammary Tissue	G=0.716	A=0.284	-0.435156	5.93E-13
rs6927004	rs58205228	chr6:93778613	0.0454394	0.81114188	EPHA7	ENSG00000135333.13	Breast - Mammary Tissue	A=0.716	G=0.284	-0.431238	6.79E-13
rs6927004	rs9345323	chr6:93797607	0.0454394	0.81114188	EPHA7	ENSG00000135333.13	Breast - Mammary Tissue	C=0.716	T=0.284	-0.431238	6.79E-13
rs6927004	rs9342350	chr6:93798264	0.0454394	0.81114188	EPHA7	ENSG00000135333.13	Breast - Mammary Tissue	A=0.716	G=0.284	-0.431238	6.79E-13
rs6927004	rs9363029	chr6:93798520	0.0454394	0.81114188	EPHA7	ENSG00000135333.13	Breast - Mammary Tissue	T=0.716	G=0.284	-0.431238	6.79E-13
rs6927004	rs9345324	chr6:93803037	0.0454394	0.81114188	EPHA7	ENSG00000135333.13	Breast - Mammary Tissue	C=0.716	A=0.284	-0.431238	6.79E-13
rs6927004	rs9363030	chr6:93806040	0.04397051	0.80778141	EPHA7	ENSG00000135333.13	Breast - Mammary Tissue	G=0.721	A=0.279	-0.42573	1.72E-12
rs6927004	rs1570631	chr6:93811876	0.04455544	0.80913985	EPHA7	ENSG00000135333.13	Breast - Mammary Tissue	T=0.719	C=0.281	-0.42573	1.72E-12
rs6927004	rs1535833	chr6:93769380	0.04367934	0.80709492	EPHA7	ENSG00000135333.13	Breast - Mammary Tissue	C=0.722	T=0.278	-0.427079	1.88E-12
rs6927004	rs9345321	chr6:93789199	0.0454394	0.81114188	EPHA7	ENSG00000135333.13	Breast - Mammary Tissue	T=0.716	G=0.284	-0.422298	1.97E-12
rs6927004	rs9345322	chr6:93790565	0.0454394	0.81114188	EPHA7	ENSG00000135333.13	Breast - Mammary Tissue	A=0.716	G=0.284	-0.422298	1.97E-12
rs6927004	rs9363030	chr6:93806040	0.04397051	0.80778141	EPHA7	ENSG00000135333.13	Nerve - Tibial	G=0.721	A=0.279	-0.360092	2.81E-12
rs6927004	rs9363029	chr6:93798520	0.0454394	0.81114188	EPHA7	ENSG00000135333.13	Nerve - Tibial	T=0.716	G=0.284	-0.358549	2.86E-12
rs6927004	rs9342350	chr6:93798264	0.0454394	0.81114188	EPHA7	ENSG00000135333.13	Nerve - Tibial	A=0.716	G=0.284	-0.358336	3.08E-12
rs6927004	rs9345324	chr6:93803037	0.0454394	0.81114188	EPHA7	ENSG00000135333.13	Nerve - Tibial	C=0.716	A=0.284	-0.358336	3.08E-12
rs6927004	rs9345323	chr6:93797607	0.0454394	0.81114188	EPHA7	ENSG00000135333.13	Nerve - Tibial	C=0.716	T=0.284	-0.352327	5.27E-12
rs6927004	rs77898219	chr6:93824234	0.04367934	0.80709492	EPHA7	ENSG00000135333.13	Breast - Mammary Tissue	C=0.722	-=0.278	-0.410352	5.75E-12
rs6927004	rs58205228	chr6:93778613	0.0454394	0.81114188	EPHA7	ENSG00000135333.13	Nerve - Tibial	A=0.716	G=0.284	-0.353209	6.01E-12
rs6927004	rs9353982	chr6:93802038	0.0454394	0.81114188	EPHA7	ENSG00000135333.13	Nerve - Tibial	G=0.716	A=0.284	-0.352973	7.65E-12
rs6927004	rs1570631	chr6:93811876	0.04455544	0.80913985	EPHA7	ENSG00000135333.13	Nerve - Tibial	T=0.719	C=0.281	-0.354962	8.15E-12
rs6927004	rs77898219	chr6:93824234	0.04367934	0.80709492	EPHA7	ENSG00000135333.13	Nerve - Tibial	C=0.722	-=0.278	-0.348397	9.13E-12
rs6927004	rs1535833	chr6:93769380	0.04367934	0.80709492	EPHA7	ENSG00000135333.13	Nerve - Tibial	C=0.722	T=0.278	-0.34822	1.38E-11
rs6927004	rs9345321	chr6:93789199	0.0454394	0.81114188	EPHA7	ENSG00000135333.13	Nerve - Tibial	T=0.716	G=0.284	-0.347757	1.48E-11
rs6927004	rs9345322	chr6:93790565	0.0454394	0.81114188	EPHA7	ENSG00000135333.13	Nerve - Tibial	A=0.716	G=0.284	-0.347757	1.48E-11
rs6927004	rs117365486	chr6:93820610	0.00571211	1	EPHA7	ENSG00000135333.13	Adipose - Subcutaneous	C=0.968	T=0.032	-0.679264	8.33E-10
rs6927004	rs148086058	chr6:93820887	0.00571211	1	EPHA7	ENSG00000135333.13	Adipose - Subcutaneous	C=0.968	A=0.032	-0.679264	8.33E-10
rs6927004	rs189136709	chr6:93783798	0.00086844	1	EPHA7	ENSG00000135333.13	Brain - Anterior cingulate cortex (BA24)	G=0.995	A=0.005	1.35876	1.60E-08
rs6927004	rs1386276	chr6:94082749	0.00408229	0.80709492	EPHA7	ENSG00000135333.13	Heart - Left Ventricle	A=0.965	G=0.035	-0.463277	1.18E-07
rs6927004	rs79718749	chr6:94098832	0.00408229	0.80709492	EPHA7	ENSG00000135333.13	Heart - Left Ventricle	T=0.965	C=0.035	-0.463277	1.18E-07
rs6927004	rs12661215	chr6:94099119	0.00408229	0.80709492	EPHA7	ENSG00000135333.13	Heart - Left Ventricle	C=0.965	T=0.035	-0.463277	1.18E-07
rs6927004	rs16871305	chr6:94111173	0.00408229	0.80709492	EPHA7	ENSG00000135333.13	Heart - Left Ventricle	A=0.965	T=0.035	-0.463277	1.18E-07
rs6927004	rs80300952	chr6:94115209	0.00408229	0.80709492	EPHA7	ENSG00000135333.13	Heart - Left Ventricle	T=0.965	C=0.035	-0.463277	1.18E-07
rs6927004	rs117376030	chr6:94203683	0.00461267	1	EPHA7	ENSG00000135333.13	Esophagus - Gastroesophageal Junction	T=0.974	A=0.026	-0.384156	2.51E-06
rs6927004	rs72928597	chr6:93839843	0.0152247	1	EPHA7	ENSG00000135333.13	Lung	A=0.919	G=0.081	0.42955	4.55E-06
rs6927004	rs16870853	chr6:93841320	0.0152247	1	EPHA7	ENSG00000135333.13	Lung	T=0.919	C=0.081	0.42955	4.55E-06
rs6927004	rs1930933	chr6:93842544	0.07093782	0.97756918	EPHA7	ENSG00000135333.13	Minor Salivary Gland	C=0.299	T=0.701	0.246092	4.56E-06

rs6927004	rs142723671	chr6:94076414	0.00388717	1	EPHA7	ENSG00000135333.13	Heart - Left Ventricle	G=0.978	C=0.022	-0.495017	4.73E-06
rs6927004	rs147330775	chr6:93915683	0.00104317	1	EPHA7	ENSG00000135333.13	Brain - Anterior cingulate cortex (BA24)	C=0.994	T=0.006	0.808887	6.34E-06
rs6927004	rs16870793	chr6:93828599	0.01481674	1	EPHA7	ENSG00000135333.13	Lung	G=0.921	A=0.079	0.419095	6.62E-06
rs6927004	rs72928592	chr6:93837959	0.0150205	1	EPHA7	ENSG00000135333.13	Lung	T=0.92	C=0.08	0.419095	6.62E-06
rs6927004	rs1575540	chr6:93836577	0.01063715	1	EPHA7	ENSG00000135333.13	Esophagus - Muscularis	G=0.942	A=0.058	0.272463	7.68E-06
rs6927004	rs544639807	chr6:93883657	0.00139369	1	EPHA7	ENSG00000135333.13	Minor Salivary Gland	G=0.992	A=0.008	-1.34812	8.25E-06
rs6927004	rs36114673	chr6:93847056	0.01141892	1	EPHA7	ENSG00000135333.13	Esophagus - Muscularis	A=0.938	T=0.062	0.268662	8.26E-06
rs6927004	rs7776099	chr6:93913423	0.02648532	0.87798173	EPHA7	ENSG00000135333.13	Testis	C=0.835	A=0.165	0.3716	8.26E-06
rs6927004	rs7758242	chr6:93913435	0.02648532	0.87798173	EPHA7	ENSG00000135333.13	Testis	T=0.835	A=0.165	0.3716	8.26E-06
rs6927004	rs66765303	chr6:93913692	0.02648532	0.87798173	EPHA7	ENSG00000135333.13	Testis	A=0.835	G=0.165	0.3716	8.26E-06
rs6927004	rs66522431	chr6:93913825	0.02648532	0.87798173	EPHA7	ENSG00000135333.13	Testis	G=0.835	A=0.165	0.3716	8.26E-06
rs6927004	rs62414181	chr6:93913909	0.02648532	0.87798173	EPHA7	ENSG00000135333.13	Testis	A=0.835	G=0.165	0.3716	8.26E-06
rs6927004	rs62414182	chr6:93914022	0.02648532	0.87798173	EPHA7	ENSG00000135333.13	Testis	A=0.835	G=0.165	0.3716	8.26E-06
rs6927004	rs113888577	chr6:93698093	0.00209894	1	EPHA7	ENSG00000135333.13	Brain - Anterior cingulate cortex (BA24)	C=0.988	T=0.012	0.66365	8.36E-06
rs6927004	rs16870780	chr6:93826505	0.0150205	1	EPHA7	ENSG00000135333.13	Lung	T=0.92	A=0.08	0.385133	9.62E-06
rs6927004	rs1590384	chr6:93837434	0.07093782	0.97756918	EPHA7	ENSG00000135333.13	Minor Salivary Gland	C=0.701	G=0.299	-0.24131	1.00E-05
rs6927004	rs1924474	chr6:93837991	0.07093782	0.97756918	EPHA7	ENSG00000135333.13	Minor Salivary Gland	C=0.701	T=0.299	-0.24131	1.00E-05
rs6927004	rs10944652	chr6:93838348	0.07093782	0.97756918	EPHA7	ENSG00000135333.13	Minor Salivary Gland	C=0.701	G=0.299	-0.24131	1.00E-05
rs6927004	rs12204186	chr6:93838665	0.07093782	0.97756918	EPHA7	ENSG00000135333.13	Minor Salivary Gland	C=0.701	T=0.299	-0.24131	1.00E-05
rs6927004	rs633279	chr6:93840705	0.07093782	0.97756918	EPHA7	ENSG00000135333.13	Minor Salivary Gland	A=0.299	T=0.701	0.24131	1.00E-05
rs6927004	rs634060	chr6:93840849	0.07093782	0.97756918	EPHA7	ENSG00000135333.13	Minor Salivary Gland	A=0.299	G=0.701	0.24131	1.00E-05
rs6927004	rs568957	chr6:93840855	0.07093782	0.97756918	EPHA7	ENSG00000135333.13	Minor Salivary Gland	G=0.299	T=0.701	0.24131	1.00E-05
rs6927004	rs34544163	chr6:93841565	0.07093782	0.97756918	EPHA7	ENSG00000135333.13	Minor Salivary Gland	-=0.299	A=0.701	0.24131	1.00E-05
rs6927004	rs562379	chr6:93841592	0.07093782	0.97756918	EPHA7	ENSG00000135333.13	Minor Salivary Gland	G=0.299	A=0.701	0.24131	1.00E-05
rs6927004	rs560731	chr6:93841734	0.07093782	0.97756918	EPHA7	ENSG00000135333.13	Minor Salivary Gland	A=0.299	G=0.701	0.24131	1.00E-05
rs6927004	rs650642	chr6:93842232	0.07093782	0.97756918	EPHA7	ENSG00000135333.13	Minor Salivary Gland	G=0.299	T=0.701	0.24131	1.00E-05
rs6927004	rs650747	chr6:93842298	0.07093782	0.97756918	EPHA7	ENSG00000135333.13	Minor Salivary Gland	T=0.299	C=0.701	0.24131	1.00E-05
rs6927004	rs1930934	chr6:93842533	0.07093782	0.97756918	EPHA7	ENSG00000135333.13	Minor Salivary Gland	A=0.299	G=0.701	0.24131	1.00E-05
rs6927004	rs9353987	chr6:93843588	0.07093782	0.97756918	EPHA7	ENSG00000135333.13	Minor Salivary Gland	A=0.299	G=0.701	0.24131	1.00E-05
rs6927004	rs9363034	chr6:93844136	0.07093782	0.97756918	EPHA7	ENSG00000135333.13	Minor Salivary Gland	G=0.299	A=0.701	0.24131	1.00E-05
rs6927004	rs9353989	chr6:93844383	0.07093782	0.97756918	EPHA7	ENSG00000135333.13	Minor Salivary Gland	G=0.299	T=0.701	0.24131	1.00E-05
rs6927004	rs76282783	chr6:93576050	0.00533527	0.83924577	EPHA7	ENSG00000135333.13	Lung	C=0.958	A=0.042	0.702179	1.18E-05
rs6927004	rs199888991	chr6:93784078	0.00086844	1	EPHA7	ENSG00000135333.13	Adipose - Subcutaneous	-=0.995	T=0.005	-0.796053	1.44E-05
rs6927004	rs111429740	chr6:93838453	0.0152247	1	EPHA7	ENSG00000135333.13	Lung	G=0.919	A=0.081	0.383525	1.55E-05
rs6927004	rs16870789	chr6:93827963	0.0150205	1	EPHA7	ENSG00000135333.13	Lung	C=0.92	T=0.08	0.374368	1.81E-05
rs6927004	rs145810085	chr6:93833819	0.00139369	1	EPHA7	ENSG00000135333.13	Adipose - Subcutaneous	G=0.992	A=0.008	-0.735109	1.88E-05
rs6927004	rs7774823	chr6:93830545	0.07024556	0.97741914	EPHA7	ENSG00000135333.13	Minor Salivary Gland	G=0.703	A=0.297	-0.232136	1.89E-05
rs6927004	rs1951907	chr6:93831192	0.07024556	0.97741914	EPHA7	ENSG00000135333.13	Minor Salivary Gland	T=0.703	C=0.297	-0.232136	1.89E-05
rs6927004	rs35306488	chr6:93832227	0.0692145	0.97719028	EPHA7	ENSG00000135333.13	Minor Salivary Gland	-=0.706	T=0.294	-0.232136	1.89E-05
rs6927004	rs10944648	chr6:93832280	0.07024556	0.97741914	EPHA7	ENSG00000135333.13	Minor Salivary Gland	A=0.703	G=0.297	-0.232136	1.89E-05
rs6927004	rs7751375	chr6:93832923	0.0705912	0.97749441	EPHA7	ENSG00000135333.13	Minor Salivary Gland	C=0.702	A=0.298	-0.232136	1.89E-05
rs6927004	rs7757292	chr6:93834165	0.07024556	0.97741914	EPHA7	ENSG00000135333.13	Minor Salivary Gland	C=0.703	A=0.297	-0.232136	1.89E-05
rs6927004	rs16870850	chr6:93841079	0.01604592	1	EPHA7	ENSG00000135333.13	Lung	T=0.916	C=0.084	0.361979	2.02E-05
rs6927004	rs12528846	chr6:93828842	0.0150205	1	EPHA7	ENSG00000135333.13	Lung	A=0.92	G=0.08	0.377147	2.03E-05
rs6927004	rs12527296	chr6:93829143	0.0150205	1	EPHA7	ENSG00000135333.13	Lung	T=0.92	C=0.08	0.377147	2.03E-05
rs6927004	rs72928575	chr6:93829399	0.0152247	1	EPHA7	ENSG00000135333.13	Lung	G=0.919	T=0.081	0.377147	2.03E-05
rs6927004	rs72928579	chr6:93829658	0.0150205	1	EPHA7	ENSG00000135333.13	Lung	G=0.92	A=0.08	0.377147	2.03E-05
rs6927004	rs77007574	chr6:93829960	0.0150205	1	EPHA7	ENSG00000135333.13	Lung	T=0.92	C=0.08	0.377147	2.03E-05
rs6927004	rs72928582	chr6:93830015	0.0150205	1	EPHA7	ENSG00000135333.13	Lung	G=0.92	A=0.08	0.377147	2.03E-05
rs6927004	rs16870801	chr6:93830951	0.0150205	1	EPHA7	ENSG00000135333.13	Lung	T=0.92	C=0.08	0.377147	2.03E-05
rs6927004	rs16870805	chr6:93834075	0.0150205	1	EPHA7	ENSG00000135333.13	Lung	G=0.92	T=0.08	0.377147	2.03E-05

rs6927004	rs150304721	chr6:93835801	0.01542934	1	EPHA7	ENSG00000135333.13	Lung	A=0.918	-=0.082	0.361316	2.04E-05
rs6927004	rs16870855	chr6:93841440	0.0152247	1	EPHA7	ENSG00000135333.13	Lung	C=0.919	T=0.081	0.4288	2.14E-05
rs6927004	rs16880183	chr6:93889701	0.03137644	0.95908074	EPHA7	ENSG00000135333.13	Testis	A=0.836	G=0.164	0.353058	2.24E-05
rs6927004	rs7772178	chr6:93849608	0.02310333	1	EPHA7	ENSG00000135333.13	Testis	C=0.883	T=0.117	0.425527	2.47E-05
rs6927004	rs6923008	chr6:93887045	0.03137644	0.95908074	EPHA7	ENSG00000135333.13	Testis	G=0.836	T=0.164	0.351036	2.54E-05
rs6927004	rs60592958	chr6:93887933	0.03137644	0.95908074	EPHA7	ENSG00000135333.13	Testis	C=0.836	T=0.164	0.351036	2.54E-05
rs6927004	rs73758259	chr6:93828724	0.01563442	1	EPHA7	ENSG00000135333.13	Lung	G=0.917	T=0.083	0.354112	2.72E-05
rs6927004	rs16870796	chr6:93828781	0.0150205	1	EPHA7	ENSG00000135333.13	Lung	T=0.92	G=0.08	0.369069	2.76E-05
rs6927004	rs72928577	chr6:93829400	0.0152247	1	EPHA7	ENSG00000135333.13	Lung	C=0.919	T=0.081	0.369069	2.76E-05
rs6927004	rs117717826	chr6:94036945	0.00443074	1	EPHA7	ENSG00000135333.13	Heart - Left Ventricle	T=0.975	A=0.025	-0.389622	2.96E-05
rs6927004	rs56378615	chr6:93900091	0.02476734	0.90876111	EPHA7	ENSG00000135333.13	Testis	C=0.853	T=0.147	0.358985	3.02E-05
rs6927004	rs71558449	chr6:93828589	0.00589667	1	EPHA7	ENSG00000135333.13	Esophagus - Muscularis	G=0.967	A=0.033	0.272458	3.14E-05
rs6927004	rs6922405	chr6:93842847	0.0152247	1	EPHA7	ENSG00000135333.13	Lung	T=0.919	C=0.081	0.370721	3.25E-05
rs6927004	rs72930416	chr6:93844150	0.0152247	1	EPHA7	ENSG00000135333.13	Lung	T=0.919	C=0.081	0.370721	3.25E-05
rs6927004	rs16870846	chr6:93840909	0.0152247	1	EPHA7	ENSG00000135333.13	Lung	C=0.919	G=0.081	0.371767	3.40E-05
rs6927004	rs1319460	chr6:93846637	0.05447383	1	EPHA7	ENSG00000135333.13	Minor Salivary Gland	C=0.239	T=0.761	0.230876	3.54E-05
rs6927004	rs538751	chr6:93848400	0.05447383	1	EPHA7	ENSG00000135333.13	Minor Salivary Gland	A=0.239	C=0.761	0.230876	3.54E-05
rs6927004	rs11422146	chr6:93849652	0.05417613	1	EPHA7	ENSG00000135333.13	Minor Salivary Gland	-=0.238	T=0.762	0.230876	3.54E-05
rs6927004	rs473900	chr6:93931037	0.03724721	0.85258345	EPHA7	ENSG00000135333.13	Adipose - Subcutaneous	T=0.228	C=0.772	0.223628	3.56E-05
rs6927004	rs369196	chr6:93932634	0.03724721	0.85258345	EPHA7	ENSG00000135333.13	Adipose - Subcutaneous	C=0.228	T=0.772	0.223628	3.56E-05
rs6927004	rs1319460	chr6:93846637	0.05447383	1	EPHA7	ENSG00000135333.13	Testis	C=0.239	T=0.761	-0.330556	3.66E-05
rs6927004	rs538751	chr6:93848400	0.05447383	1	EPHA7	ENSG00000135333.13	Testis	A=0.239	C=0.761	-0.330556	3.66E-05
rs6927004	rs11422146	chr6:93849652	0.05417613	1	EPHA7	ENSG00000135333.13	Testis	-=0.238	T=0.762	-0.330556	3.66E-05
rs6927004	rs6935219	chr6:93889277	0.03137644	0.95908074	EPHA7	ENSG00000135333.13	Testis	A=0.836	G=0.164	0.33974	4.29E-05
rs6927004	rs140182812	chr6:93804212	0.00139369	1	EPHA7	ENSG00000135333.13	Adipose - Subcutaneous	G=0.992	A=0.008	-0.729088	4.39E-05
rs6927004	rs10686381	chr6:93887552	0.03137644	0.95908074	EPHA7	ENSG00000135333.13	Testis	-=0.836	AAGT=0.164	0.337644	4.84E-05
rs6927004	rs12530331	chr6:93830460	0.0152247	1	EPHA7	ENSG00000135333.13	Lung	G=0.919	C=0.081	0.35653	5.33E-05
rs6927004	rs74468395	chr6:94017659	0.00589667	1	EPHA7	ENSG00000135333.13	Heart - Left Ventricle	C=0.967	T=0.033	-0.353	6.19E-05
rs6927004	rs117256127	chr6:93852714	0.00424918	1	EPHA7	ENSG00000135333.13	Adipose - Subcutaneous	T=0.976	C=0.024	-0.674139	6.42E-05
rs6927004	rs117425371	chr6:93852717	0.00424918	1	EPHA7	ENSG00000135333.13	Adipose - Subcutaneous	A=0.976	G=0.024	-0.674139	6.42E-05
rs6927004	rs145519743	chr6:93884032	0.00443074	1	EPHA7	ENSG00000135333.13	Adipose - Subcutaneous	G=0.975	A=0.025	-0.674139	6.42E-05
rs6927004	rs2780662	chr6:93920660	0.05119093	0.85884994	EPHA7	ENSG00000135333.13	Testis	T=0.285	C=0.715	-0.29987	6.44E-05
rs6927004	rs9351349	chr6:93832104	0.02984058	0.88747204	EPHA7	ENSG00000135333.13	Adipose - Subcutaneous	G=0.179	A=0.821	0.228214	6.48E-05
rs6927004	rs147330775	chr6:93915683	0.00104317	1	EPHA7	ENSG00000135333.13	Skin - Sun Exposed (Lower leg)	C=0.994	T=0.006	-0.47159	6.56E-05
rs6927004	rs117109139	chr6:93861398	0.00682517	1	EPHA7	ENSG00000135333.13	Esophagus - Muscularis	C=0.962	G=0.038	0.257424	7.15E-05
rs6927004	rs650711	chr6:93842280	0.02984058	0.88747204	EPHA7	ENSG00000135333.13	Adipose - Subcutaneous	T=0.821	G=0.179	-0.226702	7.44E-05
rs6927004	rs768382	chr6:93826168	0.02959694	0.88684339	EPHA7	ENSG00000135333.13	Adipose - Subcutaneous	T=0.178	C=0.822	0.223191	7.87E-05
rs6927004	rs412388	chr6:93916878	0.05119093	0.85884994	EPHA7	ENSG00000135333.13	Testis	G=0.285	A=0.715	-0.296773	8.64E-05
rs6927004	rs1324110	chr6:93913200	0.1551874	0.83299737	EPHA7	ENSG00000135333.13	Heart - Atrial Appendage	G=0.563	C=0.437	0.0819979	9.78E-05
rs6927004	rs138022068	chr6:94069111	0.00643297	0.85934004	EPHA7	ENSG00000135333.13	Heart - Left Ventricle	C=0.952	-=0.048	-0.300501	0.00011172
rs6927004	rs11970583	chr6:93895756	0.04367934	0.80709492	EPHA7	ENSG00000135333.13	Esophagus - Muscularis	C=0.722	T=0.278	0.0990772	0.00011475
rs6927004	rs2065582	chr6:93896309	0.04367934	0.80709492	EPHA7	ENSG00000135333.13	Esophagus - Muscularis	G=0.722	T=0.278	0.0990772	0.00011475
rs6927004	rs2147225	chr6:93898127	0.04367934	0.80709492	EPHA7	ENSG00000135333.13	Esophagus - Muscularis	T=0.722	A=0.278	0.0990772	0.00011475
rs6927004	rs13205265	chr6:93898896	0.04367934	0.80709492	EPHA7	ENSG00000135333.13	Esophagus - Muscularis	C=0.722	T=0.278	0.0990772	0.00011475
rs6927004	rs35480101	chr6:93899371	0.04338904	0.8064035	EPHA7	ENSG00000135333.13	Esophagus - Muscularis	G=0.723	T=0.277	0.0990772	0.00011475
rs6927004	rs145046790	chr6:93899520	0.04367934	0.80709492	EPHA7	ENSG00000135333.13	Esophagus - Muscularis	AT=0.722	-=0.278	0.0990772	0.00011475
rs6927004	rs192604147	chr6:94240629	0.00156947	1	EPHA7	ENSG00000135333.13	Heart - Left Ventricle	A=0.991	G=0.009	-0.514265	0.00011598
rs6927004	rs9345321	chr6:93789199	0.0454394	0.81114188	EPHA7	ENSG00000135333.13	Esophagus - Mucosa	T=0.716	G=0.284	-0.209017	0.00012891
rs6927004	rs9345322	chr6:93790565	0.0454394	0.81114188	EPHA7	ENSG00000135333.13	Esophagus - Mucosa	A=0.716	G=0.284	-0.209017	0.00012891
rs6927004	rs9353982	chr6:93802038	0.0454394	0.81114188	EPHA7	ENSG00000135333.13	Esophagus - Mucosa	G=0.716	A=0.284	-0.209017	0.00012891
rs6927004	rs16880179	chr6:93889506	0.02684937	0.95375563	EPHA7	ENSG00000135333.13	Testis	G=0.855	A=0.145	0.335079	0.00013631

rs6927004	rs59810007	chr6:93891733	0.02684937	0.95375563	EPHA7	ENSG00000135333.13	Testis	T=0.855	C=0.145	0.335079	0.00013631
rs6927004	rs6901961	chr6:93892700	0.02684937	0.95375563	EPHA7	ENSG00000135333.13	Testis	T=0.855	A=0.145	0.335079	0.00013631
rs6927004	rs60804851	chr6:93894513	0.02684937	0.95375563	EPHA7	ENSG00000135333.13	Testis	C=0.855	G=0.145	0.335079	0.00013631
rs6927004	rs6901870	chr6:93895903	0.02684937	0.95375563	EPHA7	ENSG00000135333.13	Testis	A=0.855	C=0.145	0.335079	0.00013631
rs6927004	rs6901827	chr6:93895828	0.04367934	0.80709492	EPHA7	ENSG00000135333.13	Esophagus - Muscularis	A=0.722	G=0.278	0.0976714	0.00014821
rs6927004	rs2181805	chr6:93898026	0.04367934	0.80709492	EPHA7	ENSG00000135333.13	Esophagus - Muscularis	T=0.722	C=0.278	0.0976714	0.00014821
rs6927004	rs2631562	chr6:93920850	0.05088319	0.85835641	EPHA7	ENSG00000135333.13	Testis	T=0.284	C=0.716	-0.28302	0.00014886
rs6927004	rs143801031	chr6:93750441	0.00298853	1	EPHA7	ENSG00000135333.13	Adipose - Subcutaneous	T=0.983	C=0.017	-0.536087	0.00015072
rs6927004	rs58205228	chr6:93778613	0.0454394	0.81114188	EPHA7	ENSG00000135333.13	Esophagus - Mucosa	A=0.716	G=0.284	-0.206708	0.00015126
rs6927004	rs9342350	chr6:93798264	0.0454394	0.81114188	EPHA7	ENSG00000135333.13	Esophagus - Mucosa	A=0.716	G=0.284	-0.206708	0.00015126
rs6927004	rs9363029	chr6:93798520	0.0454394	0.81114188	EPHA7	ENSG00000135333.13	Esophagus - Mucosa	T=0.716	G=0.284	-0.206708	0.00015126
rs6927004	rs9345324	chr6:93803037	0.0454394	0.81114188	EPHA7	ENSG00000135333.13	Esophagus - Mucosa	C=0.716	A=0.284	-0.206708	0.00015126
rs6927004	rs6922792	chr6:93887222	0.02684937	0.95375563	EPHA7	ENSG00000135333.13	Testis	A=0.855	G=0.145	0.333048	0.00015245
rs6927004	rs117365486	chr6:93820610	0.00571211	1	EPHA7	ENSG00000135333.13	Nerve - Tibial	C=0.968	T=0.032	-0.404506	0.000162
rs6927004	rs148086058	chr6:93820887	0.00571211	1	EPHA7	ENSG00000135333.13	Nerve - Tibial	C=0.968	A=0.032	-0.404506	0.000162
rs6927004	rs11966965	chr6:93890049	0.04338904	0.8064035	EPHA7	ENSG00000135333.13	Esophagus - Muscularis	G=0.723	T=0.277	0.096593	0.00016937
rs6927004	rs35373649	chr6:93891073	0.04338904	0.8064035	EPHA7	ENSG00000135333.13	Esophagus - Muscularis	A=0.723	G=0.277	0.096593	0.00016937
rs6927004	rs6921600	chr6:93892299	0.04367934	0.80709492	EPHA7	ENSG00000135333.13	Esophagus - Muscularis	G=0.722	T=0.278	0.096593	0.00016937
rs6927004	rs6901416	chr6:93892412	0.04367934	0.80709492	EPHA7	ENSG00000135333.13	Esophagus - Muscularis	T=0.722	G=0.278	0.096593	0.00016937
rs6927004	rs9345323	chr6:93797607	0.0454394	0.81114188	EPHA7	ENSG00000135333.13	Esophagus - Mucosa	C=0.716	T=0.284	-0.204207	0.00017144
rs6927004	rs1535833	chr6:93769380	0.04367934	0.80709492	EPHA7	ENSG00000135333.13	Esophagus - Mucosa	C=0.722	T=0.278	-0.205304	0.00017505
rs6927004	rs77898219	chr6:93824234	0.04367934	0.80709492	EPHA7	ENSG00000135333.13	Esophagus - Mucosa	C=0.722	-=0.278	-0.207398	0.00017867
rs6927004	rs9363030	chr6:93806040	0.04397051	0.80778141	EPHA7	ENSG00000135333.13	Esophagus - Mucosa	G=0.721	A=0.279	-0.206095	0.00019318
rs6927004	rs1953145	chr6:93890592	0.04338904	0.8064035	EPHA7	ENSG00000135333.13	Esophagus - Muscularis	G=0.723	A=0.277	0.0952131	0.00021698
rs6927004	rs12110542	chr6:93894263	0.04367934	0.80709492	EPHA7	ENSG00000135333.13	Esophagus - Muscularis	T=0.722	G=0.278	0.0952131	0.00021698
rs6927004	rs1953146	chr6:93897891	0.04367934	0.80709492	EPHA7	ENSG00000135333.13	Esophagus - Muscularis	C=0.722	G=0.278	0.0952131	0.00021698
rs6927004	rs182357880	chr6:93879803	0.00121825	1	EPHA7	ENSG00000135333.13	Skin - Sun Exposed (Lower leg)	T=0.993	C=0.007	-0.395624	0.00022421
rs6927004	rs144384339	chr6:93897863	0.00370671	1	EPHA7	ENSG00000135333.13	Skin - Sun Exposed (Lower leg)	G=0.979	A=0.021	-0.418007	0.00029351
rs72928570	rs117365486	chr6:93820610	0.0062598	1	EPHA7	ENSG00000135333.13	Adipose - Subcutaneous	C=0.968	T=0.032	-0.679264	8.33E-10
rs72928570	rs148086058	chr6:93820887	0.0062598	1	EPHA7	ENSG00000135333.13	Adipose - Subcutaneous	C=0.968	A=0.032	-0.679264	8.33E-10
rs72928570	rs189136709	chr6:93783798	0.00095171	1	EPHA7	ENSG00000135333.13	Brain - Anterior cingulate cortex (BA24)	G=0.995	A=0.005	1.35876	1.60E-08
rs72928570	rs1386276	chr6:94082749	0.00463452	0.82147294	EPHA7	ENSG00000135333.13	Heart - Left Ventricle	A=0.965	G=0.035	-0.463277	1.18E-07
rs72928570	rs79718749	chr6:94098832	0.00463452	0.82147294	EPHA7	ENSG00000135333.13	Heart - Left Ventricle	T=0.965	C=0.035	-0.463277	1.18E-07
rs72928570	rs12661215	chr6:94099119	0.00463452	0.82147294	EPHA7	ENSG00000135333.13	Heart - Left Ventricle	C=0.965	T=0.035	-0.463277	1.18E-07
rs72928570	rs16871305	chr6:94111173	0.00463452	0.82147294	EPHA7	ENSG00000135333.13	Heart - Left Ventricle	A=0.965	T=0.035	-0.463277	1.18E-07
rs72928570	rs80300952	chr6:94115209	0.00463452	0.82147294	EPHA7	ENSG00000135333.13	Heart - Left Ventricle	T=0.965	C=0.035	-0.463277	1.18E-07
rs72928570	rs146471836	chr6:93817042	0.00405384	0.80473602	EPHA7	ENSG00000135333.13	Adipose - Subcutaneous	C=0.968	T=0.032	-0.617165	8.35E-07
rs72928570	rs141174921	chr6:93733454	0.00405384	0.80473602	EPHA7	ENSG00000135333.13	Adipose - Subcutaneous	A=0.968	G=0.032	-0.598481	1.67E-06
rs72928570	rs117376030	chr6:94203683	0.00505495	1	EPHA7	ENSG00000135333.13	Esophagus - Gastroesophageal Junction	T=0.974	A=0.026	-0.384156	2.51E-06
rs72928570	rs72928597	chr6:93839843	0.01668447	1	EPHA7	ENSG00000135333.13	Lung	A=0.919	G=0.081	0.42955	4.55E-06
rs72928570	rs16870853	chr6:93841320	0.01668447	1	EPHA7	ENSG00000135333.13	Lung	T=0.919	C=0.081	0.42955	4.55E-06
rs72928570	rs1930933	chr6:93842544	0.08134794	1	EPHA7	ENSG00000135333.13	Minor Salivary Gland	C=0.299	T=0.701	0.246092	4.56E-06
rs72928570	rs142723671	chr6:94076414	0.00425987	1	EPHA7	ENSG00000135333.13	Heart - Left Ventricle	G=0.978	C=0.022	-0.495017	4.73E-06
rs72928570	rs147330775	chr6:93915683	0.0011432	1	EPHA7	ENSG00000135333.13	Brain - Anterior cingulate cortex (BA24)	C=0.994	T=0.006	0.808887	6.34E-06
rs72928570	rs16870793	chr6:93828599	0.0162374	1	EPHA7	ENSG00000135333.13	Lung	G=0.921	A=0.079	0.419095	6.62E-06
rs72928570	rs72928592	chr6:93837959	0.01646069	1	EPHA7	ENSG00000135333.13	Lung	T=0.92	C=0.08	0.419095	6.62E-06
rs72928570	rs1575540	chr6:93836577	0.01165705	1	EPHA7	ENSG00000135333.13	Esophagus - Muscularis	G=0.942	A=0.058	0.272463	7.68E-06
rs72928570	rs544639807	chr6:93883657	0.00152731	1	EPHA7	ENSG00000135333.13	Minor Salivary Gland	G=0.992	A=0.008	-1.34812	8.25E-06
rs72928570	rs36114673	chr6:93847056	0.01251379	1	EPHA7	ENSG00000135333.13	Esophagus - Muscularis	A=0.938	T=0.062	0.268662	8.26E-06
rs72928570	rs113888577	chr6:93698093	0.00230019	1	EPHA7	ENSG00000135333.13	Brain - Anterior cingulate cortex (BA24)	C=0.988	T=0.012	0.66365	8.36E-06
rs72928570	rs16870780	chr6:93826505	0.01646069	1	EPHA7	ENSG00000135333.13	Lung	T=0.92	A=0.08	0.385133	9.62E-06

rs72928570	rs1590384	chr6:93837434	0.08134794	1	EPHA7	ENSG00000135333.13	Minor Salivary Gland	C=0.701	G=0.299	-0.24131	1.00E-05
rs72928570	rs1924474	chr6:93837991	0.08134794	1	EPHA7	ENSG00000135333.13	Minor Salivary Gland	C=0.701	T=0.299	-0.24131	1.00E-05
rs72928570	rs10944652	chr6:93838348	0.08134794	1	EPHA7	ENSG00000135333.13	Minor Salivary Gland	C=0.701	G=0.299	-0.24131	1.00E-05
rs72928570	rs12204186	chr6:93838665	0.08134794	1	EPHA7	ENSG00000135333.13	Minor Salivary Gland	C=0.701	T=0.299	-0.24131	1.00E-05
rs72928570	rs633279	chr6:93840705	0.08134794	1	EPHA7	ENSG00000135333.13	Minor Salivary Gland	A=0.299	T=0.701	0.24131	1.00E-05
rs72928570	rs634060	chr6:93840849	0.08134794	1	EPHA7	ENSG00000135333.13	Minor Salivary Gland	A=0.299	G=0.701	0.24131	1.00E-05
rs72928570	rs568957	chr6:93840855	0.08134794	1	EPHA7	ENSG00000135333.13	Minor Salivary Gland	G=0.299	T=0.701	0.24131	1.00E-05
rs72928570	rs34544163	chr6:93841565	0.08134794	1	EPHA7	ENSG00000135333.13	Minor Salivary Gland	-=0.299	A=0.701	0.24131	1.00E-05
rs72928570	rs562379	chr6:93841592	0.08134794	1	EPHA7	ENSG00000135333.13	Minor Salivary Gland	G=0.299	A=0.701	0.24131	1.00E-05
rs72928570	rs560731	chr6:93841734	0.08134794	1	EPHA7	ENSG00000135333.13	Minor Salivary Gland	A=0.299	G=0.701	0.24131	1.00E-05
rs72928570	rs650642	chr6:93842232	0.08134794	1	EPHA7	ENSG00000135333.13	Minor Salivary Gland	G=0.299	T=0.701	0.24131	1.00E-05
rs72928570	rs650747	chr6:93842298	0.08134794	1	EPHA7	ENSG00000135333.13	Minor Salivary Gland	T=0.299	C=0.701	0.24131	1.00E-05
rs72928570	rs1930934	chr6:93842533	0.08134794	1	EPHA7	ENSG00000135333.13	Minor Salivary Gland	A=0.299	G=0.701	0.24131	1.00E-05
rs72928570	rs9353987	chr6:93843588	0.08134794	1	EPHA7	ENSG00000135333.13	Minor Salivary Gland	A=0.299	G=0.701	0.24131	1.00E-05
rs72928570	rs9363034	chr6:93844136	0.08134794	1	EPHA7	ENSG00000135333.13	Minor Salivary Gland	G=0.299	A=0.701	0.24131	1.00E-05
rs72928570	rs9353989	chr6:93844383	0.08134794	1	EPHA7	ENSG00000135333.13	Minor Salivary Gland	G=0.299	T=0.701	0.24131	1.00E-05
rs72928570	rs76282783	chr6:93576050	0.00601496	0.85122745	EPHA7	ENSG00000135333.13	Lung	C=0.958	A=0.042	0.702179	1.18E-05
rs72928570	rs199888991	chr6:93784078	0.00095171	1	EPHA7	ENSG00000135333.13	Adipose - Subcutaneous	-=0.995	T=0.005	-0.796053	1.44E-05
rs72928570	rs111429740	chr6:93838453	0.01668447	1	EPHA7	ENSG00000135333.13	Lung	G=0.919	A=0.081	0.383525	1.55E-05
rs72928570	rs16870789	chr6:93827963	0.01646069	1	EPHA7	ENSG00000135333.13	Lung	C=0.92	T=0.08	0.374368	1.81E-05
rs72928570	rs145810085	chr6:93833819	0.00152731	1	EPHA7	ENSG00000135333.13	Adipose - Subcutaneous	G=0.992	A=0.008	-0.735109	1.88E-05
rs72928570	rs7774823	chr6:93830545	0.08057883	1	EPHA7	ENSG00000135333.13	Minor Salivary Gland	G=0.703	A=0.297	-0.232136	1.89E-05
rs72928570	rs1951907	chr6:93831192	0.08057883	1	EPHA7	ENSG00000135333.13	Minor Salivary Gland	T=0.703	C=0.297	-0.232136	1.89E-05
rs72928570	rs35306488	chr6:93832227	0.07943329	1	EPHA7	ENSG00000135333.13	Minor Salivary Gland	-=0.706	T=0.294	-0.232136	1.89E-05
rs72928570	rs10944648	chr6:93832280	0.08057883	1	EPHA7	ENSG00000135333.13	Minor Salivary Gland	A=0.703	G=0.297	-0.232136	1.89E-05
rs72928570	rs7751375	chr6:93832923	0.08096284	1	EPHA7	ENSG00000135333.13	Minor Salivary Gland	C=0.702	A=0.298	-0.232136	1.89E-05
rs72928570	rs7757292	chr6:93834165	0.08057883	1	EPHA7	ENSG00000135333.13	Minor Salivary Gland	C=0.703	A=0.297	-0.232136	1.89E-05
rs72928570	rs16870850	chr6:93841079	0.01758444	1	EPHA7	ENSG00000135333.13	Lung	T=0.916	C=0.084	0.361979	2.02E-05
rs72928570	rs12528846	chr6:93828842	0.01646069	1	EPHA7	ENSG00000135333.13	Lung	A=0.92	G=0.08	0.377147	2.03E-05
rs72928570	rs12527296	chr6:93829143	0.01646069	1	EPHA7	ENSG00000135333.13	Lung	T=0.92	C=0.08	0.377147	2.03E-05
rs72928570	rs72928575	chr6:93829399	0.01668447	1	EPHA7	ENSG00000135333.13	Lung	G=0.919	T=0.081	0.377147	2.03E-05
rs72928570	rs72928579	chr6:93829658	0.01646069	1	EPHA7	ENSG00000135333.13	Lung	G=0.92	A=0.08	0.377147	2.03E-05
rs72928570	rs77007574	chr6:93829960	0.01646069	1	EPHA7	ENSG00000135333.13	Lung	T=0.92	C=0.08	0.377147	2.03E-05
rs72928570	rs72928582	chr6:93830015	0.01646069	1	EPHA7	ENSG00000135333.13	Lung	G=0.92	A=0.08	0.377147	2.03E-05
rs72928570	rs16870801	chr6:93830951	0.01646069	1	EPHA7	ENSG00000135333.13	Lung	T=0.92	C=0.08	0.377147	2.03E-05
rs72928570	rs16870805	chr6:93834075	0.01646069	1	EPHA7	ENSG00000135333.13	Lung	G=0.92	T=0.08	0.377147	2.03E-05
rs72928570	rs150304721	chr6:93835801	0.01690873	1	EPHA7	ENSG00000135333.13	Lung	A=0.918	-=0.082	0.361316	2.04E-05
rs72928570	rs16870855	chr6:93841440	0.01668447	1	EPHA7	ENSG00000135333.13	Lung	C=0.919	T=0.081	0.4288	2.14E-05
rs72928570	rs7772178	chr6:93849608	0.02531851	1	EPHA7	ENSG00000135333.13	Testis	C=0.883	T=0.117	0.425527	2.47E-05
rs72928570	rs73758259	chr6:93828724	0.01713348	1	EPHA7	ENSG00000135333.13	Lung	G=0.917	T=0.083	0.354112	2.72E-05
rs72928570	rs16870796	chr6:93828781	0.01646069	1	EPHA7	ENSG00000135333.13	Lung	T=0.92	G=0.08	0.369069	2.76E-05
rs72928570	rs72928577	chr6:93829400	0.01668447	1	EPHA7	ENSG00000135333.13	Lung	C=0.919	T=0.081	0.369069	2.76E-05
rs72928570	rs117717826	chr6:94036945	0.00485557	1	EPHA7	ENSG00000135333.13	Heart - Left Ventricle	T=0.975	A=0.025	-0.389622	2.96E-05
rs72928570	rs71558449	chr6:93828589	0.00646205	1	EPHA7	ENSG00000135333.13	Esophagus - Muscularis	G=0.967	A=0.033	0.272458	3.14E-05
rs72928570	rs6922405	chr6:93842847	0.01668447	1	EPHA7	ENSG00000135333.13	Lung	T=0.919	C=0.081	0.370721	3.25E-05
rs72928570	rs72930416	chr6:93844150	0.01668447	1	EPHA7	ENSG00000135333.13	Lung	T=0.919	C=0.081	0.370721	3.25E-05
rs72928570	rs16870846	chr6:93840909	0.01668447	1	EPHA7	ENSG00000135333.13	Lung	C=0.919	G=0.081	0.371767	3.40E-05
rs72928570	rs1319460	chr6:93846637	0.05662891	0.9739648	EPHA7	ENSG00000135333.13	Minor Salivary Gland	C=0.239	T=0.761	0.230876	3.54E-05
rs72928570	rs538751	chr6:93848400	0.05662891	0.9739648	EPHA7	ENSG00000135333.13	Minor Salivary Gland	A=0.239	C=0.761	0.230876	3.54E-05
rs72928570	rs11422146	chr6:93849652	0.05630683	0.97385587	EPHA7	ENSG00000135333.13	Minor Salivary Gland	-=0.238	T=0.762	0.230876	3.54E-05
rs72928570	rs1319460	chr6:93846637	0.05662891	0.9739648	EPHA7	ENSG00000135333.13	Testis	C=0.239	T=0.761	-0.330556	3.66E-05

rs72928570	rs538751	chr6:93848400	0.05662891	0.9739648	EPHA7	ENSG00000135333.13	Testis	A=0.239	C=0.761	-0.330556	3.66E-05
rs72928570	rs11422146	chr6:93849652	0.05630683	0.97385587	EPHA7	ENSG00000135333.13	Testis	-=0.238	T=0.762	-0.330556	3.66E-05
rs72928570	rs140182812	chr6:93804212	0.00152731	1	EPHA7	ENSG00000135333.13	Adipose - Subcutaneous	G=0.992	A=0.008	-0.729088	4.39E-05
rs72928570	rs41273625	chr6:93952851	0.01032934	0.9008183	EPHA7	ENSG00000135333.13	Esophagus - Muscularis	C=0.937	G=0.063	0.224035	5.17E-05
rs72928570	rs12530331	chr6:93830460	0.01668447	1	EPHA7	ENSG00000135333.13	Lung	G=0.919	C=0.081	0.35653	5.33E-05
rs72928570	rs74468395	chr6:94017659	0.00646205	1	EPHA7	ENSG00000135333.13	Heart - Left Ventricle	C=0.967	T=0.033	-0.353	6.19E-05
rs72928570	rs117256127	chr6:93852714	0.0046566	1	EPHA7	ENSG00000135333.13	Adipose - Subcutaneous	T=0.976	C=0.024	-0.674139	6.42E-05
rs72928570	rs117425371	chr6:93852717	0.0046566	1	EPHA7	ENSG00000135333.13	Adipose - Subcutaneous	A=0.976	G=0.024	-0.674139	6.42E-05
rs72928570	rs145519743	chr6:93884032	0.00485557	1	EPHA7	ENSG00000135333.13	Adipose - Subcutaneous	G=0.975	A=0.025	-0.674139	6.42E-05
rs72928570	rs9351349	chr6:93832104	0.04152041	1	EPHA7	ENSG00000135333.13	Adipose - Subcutaneous	G=0.179	A=0.821	0.228214	6.48E-05
rs72928570	rs147330775	chr6:93915683	0.0011432	1	EPHA7	ENSG00000135333.13	Skin - Sun Exposed (Lower leg)	C=0.994	T=0.006	-0.47159	6.56E-05
rs72928570	rs117109139	chr6:93861398	0.00747958	1	EPHA7	ENSG00000135333.13	Esophagus - Muscularis	C=0.962	G=0.038	0.257424	7.15E-05
rs72928570	rs650711	chr6:93842280	0.04152041	1	EPHA7	ENSG00000135333.13	Adipose - Subcutaneous	T=0.821	G=0.179	-0.226702	7.44E-05
rs72928570	rs768382	chr6:93826168	0.04123981	1	EPHA7	ENSG00000135333.13	Adipose - Subcutaneous	T=0.178	C=0.822	0.223191	7.87E-05
rs72928570	rs41273625	chr6:93952851	0.01032934	0.9008183	EPHA7	ENSG00000135333.13	Esophagus - Gastroesophageal Junction	C=0.937	G=0.063	0.249826	0.00010545
rs72928570	rs138022068	chr6:94069111	0.00722283	0.86982402	EPHA7	ENSG00000135333.13	Heart - Left Ventricle	C=0.952	-=0.048	-0.300501	0.00011172
rs72928570	rs192604147	chr6:94240629	0.00171995	1	EPHA7	ENSG00000135333.13	Heart - Left Ventricle	A=0.991	G=0.009	-0.514265	0.00011598
rs72928570	rs143801031	chr6:93750441	0.00327508	1	EPHA7	ENSG00000135333.13	Adipose - Subcutaneous	T=0.983	C=0.017	-0.536087	0.00015072
rs72928570	rs117365486	chr6:93820610	0.0062598	1	EPHA7	ENSG00000135333.13	Nerve - Tibial	C=0.968	T=0.032	-0.404506	0.000162
rs72928570	rs148086058	chr6:93820887	0.0062598	1	EPHA7	ENSG00000135333.13	Nerve - Tibial	C=0.968	A=0.032	-0.404506	0.000162
rs72928570	rs182357880	chr6:93879803	0.00133506	1	EPHA7	ENSG00000135333.13	Skin - Sun Exposed (Lower leg)	T=0.993	C=0.007	-0.395624	0.00022421
rs75841618	rs1535833	chr6:93769380	0.04367934	0.80709492	EPHA7	ENSG00000135333.13	Adipose - Subcutaneous	C=0.722	T=0.278	-0.770455	1.90E-63
rs75841618	rs58205228	chr6:93778613	0.0454394	0.81114188	EPHA7	ENSG00000135333.13	Adipose - Subcutaneous	A=0.716	G=0.284	-0.767837	2.77E-63
rs75841618	rs9345321	chr6:93789199	0.0454394	0.81114188	EPHA7	ENSG00000135333.13	Adipose - Subcutaneous	T=0.716	G=0.284	-0.767837	2.77E-63
rs75841618	rs9345322	chr6:93790565	0.0454394	0.81114188	EPHA7	ENSG00000135333.13	Adipose - Subcutaneous	A=0.716	G=0.284	-0.767837	2.77E-63
rs75841618	rs9345323	chr6:93797607	0.0454394	0.81114188	EPHA7	ENSG00000135333.13	Adipose - Subcutaneous	C=0.716	T=0.284	-0.767837	2.77E-63
rs75841618	rs9342350	chr6:93798264	0.0454394	0.81114188	EPHA7	ENSG00000135333.13	Adipose - Subcutaneous	A=0.716	G=0.284	-0.767837	2.77E-63
rs75841618	rs9363029	chr6:93798520	0.0454394	0.81114188	EPHA7	ENSG00000135333.13	Adipose - Subcutaneous	T=0.716	G=0.284	-0.767837	2.77E-63
rs75841618	rs9353982	chr6:93802038	0.0454394	0.81114188	EPHA7	ENSG00000135333.13	Adipose - Subcutaneous	G=0.716	A=0.284	-0.767837	2.77E-63
rs75841618	rs9345324	chr6:93803037	0.0454394	0.81114188	EPHA7	ENSG00000135333.13	Adipose - Subcutaneous	C=0.716	A=0.284	-0.767837	2.77E-63
rs75841618	rs1570631	chr6:93811876	0.04455544	0.80913985	EPHA7	ENSG00000135333.13	Adipose - Subcutaneous	T=0.719	C=0.281	-0.759574	1.45E-59
rs75841618	rs77898219	chr6:93824234	0.04367934	0.80709492	EPHA7	ENSG00000135333.13	Adipose - Subcutaneous	C=0.722	-=0.278	-0.752617	1.58E-59
rs75841618	rs9363030	chr6:93806040	0.04397051	0.80778141	EPHA7	ENSG00000135333.13	Adipose - Subcutaneous	G=0.721	A=0.279	-0.757344	1.59E-59
rs75841618	rs9363030	chr6:93806040	0.04397051	0.80778141	EPHA7	ENSG00000135333.13	Lung	G=0.721	A=0.279	-0.467048	1.04E-18
rs75841618	rs1570631	chr6:93811876	0.04455544	0.80913985	EPHA7	ENSG00000135333.13	Lung	T=0.719	C=0.281	-0.467048	1.04E-18
rs75841618	rs58205228	chr6:93778613	0.0454394	0.81114188	EPHA7	ENSG00000135333.13	Lung	A=0.716	G=0.284	-0.459589	1.54E-18
rs75841618	rs9345321	chr6:93789199	0.0454394	0.81114188	EPHA7	ENSG00000135333.13	Lung	T=0.716	G=0.284	-0.459589	1.54E-18
rs75841618	rs9345322	chr6:93790565	0.0454394	0.81114188	EPHA7	ENSG00000135333.13	Lung	A=0.716	G=0.284	-0.459589	1.54E-18
rs75841618	rs9345323	chr6:93797607	0.0454394	0.81114188	EPHA7	ENSG00000135333.13	Lung	C=0.716	T=0.284	-0.459589	1.54E-18
rs75841618	rs9342350	chr6:93798264	0.0454394	0.81114188	EPHA7	ENSG00000135333.13	Lung	A=0.716	G=0.284	-0.459589	1.54E-18
rs75841618	rs9363029	chr6:93798520	0.0454394	0.81114188	EPHA7	ENSG00000135333.13	Lung	T=0.716	G=0.284	-0.459589	1.54E-18
rs75841618	rs9353982	chr6:93802038	0.0454394	0.81114188	EPHA7	ENSG00000135333.13	Lung	G=0.716	A=0.284	-0.459589	1.54E-18
rs75841618	rs9345324	chr6:93803037	0.0454394	0.81114188	EPHA7	ENSG00000135333.13	Lung	C=0.716	A=0.284	-0.459589	1.54E-18
rs75841618	rs77898219	chr6:93824234	0.04367934	0.80709492	EPHA7	ENSG00000135333.13	Lung	C=0.722	-=0.278	-0.456134	5.88E-18
rs75841618	rs1535833	chr6:93769380	0.04367934	0.80709492	EPHA7	ENSG00000135333.13	Lung	C=0.722	T=0.278	-0.44499	2.25E-17
rs75841618	rs9363030	chr6:93806040	0.04397051	0.80778141	EPHA7	ENSG00000135333.13	Skin - Sun Exposed (Lower leg)	G=0.721	A=0.279	-0.281476	2.71E-17
rs75841618	rs9353982	chr6:93802038	0.0454394	0.81114188	EPHA7	ENSG00000135333.13	Skin - Sun Exposed (Lower leg)	G=0.716	A=0.284	-0.276454	4.84E-17
rs75841618	rs1570631	chr6:93811876	0.04455544	0.80913985	EPHA7	ENSG00000135333.13	Skin - Sun Exposed (Lower leg)	T=0.719	C=0.281	-0.279518	5.05E-17
rs75841618	rs58205228	chr6:93778613	0.0454394	0.81114188	EPHA7	ENSG00000135333.13	Skin - Sun Exposed (Lower leg)	A=0.716	G=0.284	-0.274662	6.63E-17
rs75841618	rs9345323	chr6:93797607	0.0454394	0.81114188	EPHA7	ENSG00000135333.13	Skin - Sun Exposed (Lower leg)	C=0.716	T=0.284	-0.274662	6.63E-17
rs75841618	rs9342350	chr6:93798264	0.0454394	0.81114188	EPHA7	ENSG00000135333.13	Skin - Sun Exposed (Lower leg)	A=0.716	G=0.284	-0.274662	6.63E-17

rs75841618	rs9363029	chr6:93798520	0.0454394	0.81114188	EPHA7	ENSG00000135333.13	Skin - Sun Exposed (Lower leg)	T=0.716	G=0.284	-0.274662	6.63E-17
rs75841618	rs9345324	chr6:93803037	0.0454394	0.81114188	EPHA7	ENSG00000135333.13	Skin - Sun Exposed (Lower leg)	C=0.716	A=0.284	-0.274662	6.63E-17
rs75841618	rs9345321	chr6:93789199	0.0454394	0.81114188	EPHA7	ENSG00000135333.13	Skin - Sun Exposed (Lower leg)	T=0.716	G=0.284	-0.273298	8.03E-17
rs75841618	rs9345322	chr6:93790565	0.0454394	0.81114188	EPHA7	ENSG00000135333.13	Skin - Sun Exposed (Lower leg)	A=0.716	G=0.284	-0.273298	8.03E-17
rs75841618	rs1535833	chr6:93769380	0.04367934	0.80709492	EPHA7	ENSG00000135333.13	Skin - Sun Exposed (Lower leg)	C=0.722	T=0.278	-0.274816	9.27E-17
rs75841618	rs77898219	chr6:93824234	0.04367934	0.80709492	EPHA7	ENSG00000135333.13	Skin - Sun Exposed (Lower leg)	C=0.722	-=0.278	-0.274384	1.06E-16
rs75841618	rs9363030	chr6:93806040	0.04397051	0.80778141	EPHA7	ENSG00000135333.13	Skin - Not Sun Exposed (Suprapubic)	G=0.721	A=0.279	-0.26209	8.68E-15
rs75841618	rs77898219	chr6:93824234	0.04367934	0.80709492	EPHA7	ENSG00000135333.13	Skin - Not Sun Exposed (Suprapubic)	C=0.722	-=0.278	-0.258658	1.10E-14
rs75841618	rs1570631	chr6:93811876	0.04455544	0.80913985	EPHA7	ENSG00000135333.13	Skin - Not Sun Exposed (Suprapubic)	T=0.719	C=0.281	-0.259435	1.84E-14
rs75841618	rs1535833	chr6:93769380	0.04367934	0.80709492	EPHA7	ENSG00000135333.13	Skin - Not Sun Exposed (Suprapubic)	C=0.722	T=0.278	-0.258257	2.03E-14
rs75841618	rs9342350	chr6:93798264	0.0454394	0.81114188	EPHA7	ENSG00000135333.13	Skin - Not Sun Exposed (Suprapubic)	A=0.716	G=0.284	-0.256364	2.36E-14
rs75841618	rs9363029	chr6:93798520	0.0454394	0.81114188	EPHA7	ENSG00000135333.13	Skin - Not Sun Exposed (Suprapubic)	T=0.716	G=0.284	-0.256364	2.36E-14
rs75841618	rs9353982	chr6:93802038	0.0454394	0.81114188	EPHA7	ENSG00000135333.13	Skin - Not Sun Exposed (Suprapubic)	G=0.716	A=0.284	-0.256364	2.36E-14
rs75841618	rs9345324	chr6:93803037	0.0454394	0.81114188	EPHA7	ENSG00000135333.13	Skin - Not Sun Exposed (Suprapubic)	C=0.716	A=0.284	-0.256364	2.36E-14
rs75841618	rs9345323	chr6:93797607	0.0454394	0.81114188	EPHA7	ENSG00000135333.13	Skin - Not Sun Exposed (Suprapubic)	C=0.716	T=0.284	-0.253851	3.29E-14
rs75841618	rs58205228	chr6:93778613	0.0454394	0.81114188	EPHA7	ENSG00000135333.13	Skin - Not Sun Exposed (Suprapubic)	A=0.716	G=0.284	-0.252522	5.58E-14
rs75841618	rs9345321	chr6:93789199	0.0454394	0.81114188	EPHA7	ENSG00000135333.13	Skin - Not Sun Exposed (Suprapubic)	T=0.716	G=0.284	-0.252522	5.58E-14
rs75841618	rs9345322	chr6:93790565	0.0454394	0.81114188	EPHA7	ENSG00000135333.13	Skin - Not Sun Exposed (Suprapubic)	A=0.716	G=0.284	-0.252522	5.58E-14
rs75841618	rs9353982	chr6:93802038	0.0454394	0.81114188	EPHA7	ENSG00000135333.13	Breast - Mammary Tissue	G=0.716	A=0.284	-0.435156	5.93E-13
rs75841618	rs58205228	chr6:93778613	0.0454394	0.81114188	EPHA7	ENSG00000135333.13	Breast - Mammary Tissue	A=0.716	G=0.284	-0.431238	6.79E-13
rs75841618	rs9345323	chr6:93797607	0.0454394	0.81114188	EPHA7	ENSG00000135333.13	Breast - Mammary Tissue	C=0.716	T=0.284	-0.431238	6.79E-13
rs75841618	rs9342350	chr6:93798264	0.0454394	0.81114188	EPHA7	ENSG00000135333.13	Breast - Mammary Tissue	A=0.716	G=0.284	-0.431238	6.79E-13
rs75841618	rs9363029	chr6:93798520	0.0454394	0.81114188	EPHA7	ENSG00000135333.13	Breast - Mammary Tissue	T=0.716	G=0.284	-0.431238	6.79E-13
rs75841618	rs9345324	chr6:93803037	0.0454394	0.81114188	EPHA7	ENSG00000135333.13	Breast - Mammary Tissue	C=0.716	A=0.284	-0.431238	6.79E-13
rs75841618	rs9363030	chr6:93806040	0.04397051	0.80778141	EPHA7	ENSG00000135333.13	Breast - Mammary Tissue	G=0.721	A=0.279	-0.42573	1.72E-12
rs75841618	rs1570631	chr6:93811876	0.04455544	0.80913985	EPHA7	ENSG00000135333.13	Breast - Mammary Tissue	T=0.719	C=0.281	-0.42573	1.72E-12
rs75841618	rs1535833	chr6:93769380	0.04367934	0.80709492	EPHA7	ENSG00000135333.13	Breast - Mammary Tissue	C=0.722	T=0.278	-0.427079	1.88E-12
rs75841618	rs9345321	chr6:93789199	0.0454394	0.81114188	EPHA7	ENSG00000135333.13	Breast - Mammary Tissue	T=0.716	G=0.284	-0.422298	1.97E-12
rs75841618	rs9345322	chr6:93790565	0.0454394	0.81114188	EPHA7	ENSG00000135333.13	Breast - Mammary Tissue	A=0.716	G=0.284	-0.422298	1.97E-12
rs75841618	rs9363030	chr6:93806040	0.04397051	0.80778141	EPHA7	ENSG00000135333.13	Nerve - Tibial	G=0.721	A=0.279	-0.360092	2.81E-12
rs75841618	rs9363029	chr6:93798520	0.0454394	0.81114188	EPHA7	ENSG00000135333.13	Nerve - Tibial	T=0.716	G=0.284	-0.358549	2.86E-12
rs75841618	rs9342350	chr6:93798264	0.0454394	0.81114188	EPHA7	ENSG00000135333.13	Nerve - Tibial	A=0.716	G=0.284	-0.358336	3.08E-12
rs75841618	rs9345324	chr6:93803037	0.0454394	0.81114188	EPHA7	ENSG00000135333.13	Nerve - Tibial	C=0.716	A=0.284	-0.358336	3.08E-12
rs75841618	rs9345323	chr6:93797607	0.0454394	0.81114188	EPHA7	ENSG00000135333.13	Nerve - Tibial	C=0.716	T=0.284	-0.352327	5.27E-12
rs75841618	rs77898219	chr6:93824234	0.04367934	0.80709492	EPHA7	ENSG00000135333.13	Breast - Mammary Tissue	C=0.722	-=0.278	-0.410352	5.75E-12
rs75841618	rs58205228	chr6:93778613	0.0454394	0.81114188	EPHA7	ENSG00000135333.13	Nerve - Tibial	A=0.716	G=0.284	-0.353209	6.01E-12
rs75841618	rs9353982	chr6:93802038	0.0454394	0.81114188	EPHA7	ENSG00000135333.13	Nerve - Tibial	G=0.716	A=0.284	-0.352973	7.65E-12
rs75841618	rs1570631	chr6:93811876	0.04455544	0.80913985	EPHA7	ENSG00000135333.13	Nerve - Tibial	T=0.719	C=0.281	-0.354962	8.15E-12
rs75841618	rs77898219	chr6:93824234	0.04367934	0.80709492	EPHA7	ENSG00000135333.13	Nerve - Tibial	C=0.722	-=0.278	-0.348397	9.13E-12
rs75841618	rs1535833	chr6:93769380	0.04367934	0.80709492	EPHA7	ENSG00000135333.13	Nerve - Tibial	C=0.722	T=0.278	-0.34822	1.38E-11
rs75841618	rs9345321	chr6:93789199	0.0454394	0.81114188	EPHA7	ENSG00000135333.13	Nerve - Tibial	T=0.716	G=0.284	-0.347757	1.48E-11
rs75841618	rs9345322	chr6:93790565	0.0454394	0.81114188	EPHA7	ENSG00000135333.13	Nerve - Tibial	A=0.716	G=0.284	-0.347757	1.48E-11
rs75841618	rs117365486	chr6:93820610	0.00571211	1	EPHA7	ENSG00000135333.13	Adipose - Subcutaneous	C=0.968	T=0.032	-0.679264	8.33E-10
rs75841618	rs148086058	chr6:93820887	0.00571211	1	EPHA7	ENSG00000135333.13	Adipose - Subcutaneous	C=0.968	A=0.032	-0.679264	8.33E-10
rs75841618	rs189136709	chr6:93783798	0.00086844	1	EPHA7	ENSG00000135333.13	Brain - Anterior cingulate cortex (BA24)	G=0.995	A=0.005	1.35876	1.60E-08
rs75841618	rs1386276	chr6:94082749	0.00408229	0.80709492	EPHA7	ENSG00000135333.13	Heart - Left Ventricle	A=0.965	G=0.035	-0.463277	1.18E-07
rs75841618	rs79718749	chr6:94098832	0.00408229	0.80709492	EPHA7	ENSG00000135333.13	Heart - Left Ventricle	T=0.965	C=0.035	-0.463277	1.18E-07
rs75841618	rs12661215	chr6:94099119	0.00408229	0.80709492	EPHA7	ENSG00000135333.13	Heart - Left Ventricle	C=0.965	T=0.035	-0.463277	1.18E-07
rs75841618	rs16871305	chr6:94111173	0.00408229	0.80709492	EPHA7	ENSG00000135333.13	Heart - Left Ventricle	A=0.965	T=0.035	-0.463277	1.18E-07
rs75841618	rs80300952	chr6:94115209	0.00408229	0.80709492	EPHA7	ENSG00000135333.13	Heart - Left Ventricle	T=0.965	C=0.035	-0.463277	1.18E-07
rs75841618	rs117376030	chr6:94203683	0.00461267	1	EPHA7	ENSG00000135333.13	Esophagus - Gastroesophageal Junction	T=0.974	A=0.026	-0.384156	2.51E-06

rs75841618	rs72928597	chr6:93839843	0.0152247	1	EPHA7	ENSG00000135333.13	Lung	A=0.919	G=0.081	0.42955	4.55E-06
rs75841618	rs16870853	chr6:93841320	0.0152247	1	EPHA7	ENSG00000135333.13	Lung	T=0.919	C=0.081	0.42955	4.55E-06
rs75841618	rs1930933	chr6:93842544	0.07093782	0.97756918	EPHA7	ENSG00000135333.13	Minor Salivary Gland	C=0.299	T=0.701	0.246092	4.56E-06
rs75841618	rs142723671	chr6:94076414	0.00388717	1	EPHA7	ENSG00000135333.13	Heart - Left Ventricle	G=0.978	C=0.022	-0.495017	4.73E-06
rs75841618	rs147330775	chr6:93915683	0.00104317	1	EPHA7	ENSG00000135333.13	Brain - Anterior cingulate cortex (BA24)	C=0.994	T=0.006	0.808887	6.34E-06
rs75841618	rs16870793	chr6:93828599	0.01481674	1	EPHA7	ENSG00000135333.13	Lung	G=0.921	A=0.079	0.419095	6.62E-06
rs75841618	rs72928592	chr6:93837959	0.0150205	1	EPHA7	ENSG00000135333.13	Lung	T=0.92	C=0.08	0.419095	6.62E-06
rs75841618	rs1575540	chr6:93836577	0.01063715	1	EPHA7	ENSG00000135333.13	Esophagus - Muscularis	G=0.942	A=0.058	0.272463	7.68E-06
rs75841618	rs544639807	chr6:93883657	0.00139369	1	EPHA7	ENSG00000135333.13	Minor Salivary Gland	G=0.992	A=0.008	-1.34812	8.25E-06
rs75841618	rs36114673	chr6:93847056	0.01141892	1	EPHA7	ENSG00000135333.13	Esophagus - Muscularis	A=0.938	T=0.062	0.268662	8.26E-06
rs75841618	rs7776099	chr6:93913423	0.02648532	0.87798173	EPHA7	ENSG00000135333.13	Testis	C=0.835	A=0.165	0.3716	8.26E-06
rs75841618	rs7758242	chr6:93913435	0.02648532	0.87798173	EPHA7	ENSG00000135333.13	Testis	T=0.835	A=0.165	0.3716	8.26E-06
rs75841618	rs66765303	chr6:93913692	0.02648532	0.87798173	EPHA7	ENSG00000135333.13	Testis	A=0.835	G=0.165	0.3716	8.26E-06
rs75841618	rs66522431	chr6:93913825	0.02648532	0.87798173	EPHA7	ENSG00000135333.13	Testis	G=0.835	A=0.165	0.3716	8.26E-06
rs75841618	rs62414181	chr6:93913909	0.02648532	0.87798173	EPHA7	ENSG00000135333.13	Testis	A=0.835	G=0.165	0.3716	8.26E-06
rs75841618	rs62414182	chr6:93914022	0.02648532	0.87798173	EPHA7	ENSG00000135333.13	Testis	A=0.835	G=0.165	0.3716	8.26E-06
rs75841618	rs113888577	chr6:93698093	0.00209894	1	EPHA7	ENSG00000135333.13	Brain - Anterior cingulate cortex (BA24)	C=0.988	T=0.012	0.66365	8.36E-06
rs75841618	rs16870780	chr6:93826505	0.0150205	1	EPHA7	ENSG00000135333.13	Lung	T=0.92	A=0.08	0.385133	9.62E-06
rs75841618	rs1590384	chr6:93837434	0.07093782	0.97756918	EPHA7	ENSG00000135333.13	Minor Salivary Gland	C=0.701	G=0.299	-0.24131	1.00E-05
rs75841618	rs1924474	chr6:93837991	0.07093782	0.97756918	EPHA7	ENSG00000135333.13	Minor Salivary Gland	C=0.701	T=0.299	-0.24131	1.00E-05
rs75841618	rs10944652	chr6:93838348	0.07093782	0.97756918	EPHA7	ENSG00000135333.13	Minor Salivary Gland	C=0.701	G=0.299	-0.24131	1.00E-05
rs75841618	rs12204186	chr6:93838665	0.07093782	0.97756918	EPHA7	ENSG00000135333.13	Minor Salivary Gland	C=0.701	T=0.299	-0.24131	1.00E-05
rs75841618	rs633279	chr6:93840705	0.07093782	0.97756918	EPHA7	ENSG00000135333.13	Minor Salivary Gland	A=0.299	T=0.701	0.24131	1.00E-05
rs75841618	rs634060	chr6:93840849	0.07093782	0.97756918	EPHA7	ENSG00000135333.13	Minor Salivary Gland	A=0.299	G=0.701	0.24131	1.00E-05
rs75841618	rs568957	chr6:93840855	0.07093782	0.97756918	EPHA7	ENSG00000135333.13	Minor Salivary Gland	G=0.299	T=0.701	0.24131	1.00E-05
rs75841618	rs34544163	chr6:93841565	0.07093782	0.97756918	EPHA7	ENSG00000135333.13	Minor Salivary Gland	-=0.299	A=0.701	0.24131	1.00E-05
rs75841618	rs562379	chr6:93841592	0.07093782	0.97756918	EPHA7	ENSG00000135333.13	Minor Salivary Gland	G=0.299	A=0.701	0.24131	1.00E-05
rs75841618	rs560731	chr6:93841734	0.07093782	0.97756918	EPHA7	ENSG00000135333.13	Minor Salivary Gland	A=0.299	G=0.701	0.24131	1.00E-05
rs75841618	rs650642	chr6:93842232	0.07093782	0.97756918	EPHA7	ENSG00000135333.13	Minor Salivary Gland	G=0.299	T=0.701	0.24131	1.00E-05
rs75841618	rs650747	chr6:93842298	0.07093782	0.97756918	EPHA7	ENSG00000135333.13	Minor Salivary Gland	T=0.299	C=0.701	0.24131	1.00E-05
rs75841618	rs1930934	chr6:93842533	0.07093782	0.97756918	EPHA7	ENSG00000135333.13	Minor Salivary Gland	A=0.299	G=0.701	0.24131	1.00E-05
rs75841618	rs9353987	chr6:93843588	0.07093782	0.97756918	EPHA7	ENSG00000135333.13	Minor Salivary Gland	A=0.299	G=0.701	0.24131	1.00E-05
rs75841618	rs9363034	chr6:93844136	0.07093782	0.97756918	EPHA7	ENSG00000135333.13	Minor Salivary Gland	G=0.299	A=0.701	0.24131	1.00E-05
rs75841618	rs9353989	chr6:93844383	0.07093782	0.97756918	EPHA7	ENSG00000135333.13	Minor Salivary Gland	G=0.299	T=0.701	0.24131	1.00E-05
rs75841618	rs76282783	chr6:93576050	0.00533527	0.83924577	EPHA7	ENSG00000135333.13	Lung	C=0.958	A=0.042	0.702179	1.18E-05
rs75841618	rs199888991	chr6:93784078	0.00086844	1	EPHA7	ENSG00000135333.13	Adipose - Subcutaneous	-=0.995	T=0.005	-0.796053	1.44E-05
rs75841618	rs111429740	chr6:93838453	0.0152247	1	EPHA7	ENSG00000135333.13	Lung	G=0.919	A=0.081	0.383525	1.55E-05
rs75841618	rs16870789	chr6:93827963	0.0150205	1	EPHA7	ENSG00000135333.13	Lung	C=0.92	T=0.08	0.374368	1.81E-05
rs75841618	rs145810085	chr6:93833819	0.00139369	1	EPHA7	ENSG00000135333.13	Adipose - Subcutaneous	G=0.992	A=0.008	-0.735109	1.88E-05
rs75841618	rs7774823	chr6:93830545	0.07024556	0.97741914	EPHA7	ENSG00000135333.13	Minor Salivary Gland	G=0.703	A=0.297	-0.232136	1.89E-05
rs75841618	rs1951907	chr6:93831192	0.07024556	0.97741914	EPHA7	ENSG00000135333.13	Minor Salivary Gland	T=0.703	C=0.297	-0.232136	1.89E-05
rs75841618	rs35306488	chr6:93832227	0.0692145	0.97719028	EPHA7	ENSG00000135333.13	Minor Salivary Gland	-=0.706	T=0.294	-0.232136	1.89E-05
rs75841618	rs10944648	chr6:93832280	0.07024556	0.97741914	EPHA7	ENSG00000135333.13	Minor Salivary Gland	A=0.703	G=0.297	-0.232136	1.89E-05
rs75841618	rs7751375	chr6:93832923	0.0705912	0.97749441	EPHA7	ENSG00000135333.13	Minor Salivary Gland	C=0.702	A=0.298	-0.232136	1.89E-05
rs75841618	rs7757292	chr6:93834165	0.07024556	0.97741914	EPHA7	ENSG00000135333.13	Minor Salivary Gland	C=0.703	A=0.297	-0.232136	1.89E-05
rs75841618	rs16870850	chr6:93841079	0.01604592	1	EPHA7	ENSG00000135333.13	Lung	T=0.916	C=0.084	0.361979	2.02E-05
rs75841618	rs12528846	chr6:93828842	0.0150205	1	EPHA7	ENSG00000135333.13	Lung	A=0.92	G=0.08	0.377147	2.03E-05
rs75841618	rs12527296	chr6:93829143	0.0150205	1	EPHA7	ENSG00000135333.13	Lung	T=0.92	C=0.08	0.377147	2.03E-05
rs75841618	rs72928575	chr6:93829399	0.0152247	1	EPHA7	ENSG00000135333.13	Lung	G=0.919	T=0.081	0.377147	2.03E-05
rs75841618	rs72928579	chr6:93829658	0.0150205	1	EPHA7	ENSG00000135333.13	Lung	G=0.92	A=0.08	0.377147	2.03E-05
rs75841618	rs77007574	chr6:93829960	0.0150205	1	EPHA7	ENSG00000135333.13	Lung	T=0.92	C=0.08	0.377147	2.03E-05

rs75841618	rs72928582	chr6:93830015	0.0150205	1	EPHA7	ENSG00000135333.13	Lung	G=0.92	A=0.08	0.377147	2.03E-05
rs75841618	rs16870801	chr6:93830951	0.0150205	1	EPHA7	ENSG00000135333.13	Lung	T=0.92	C=0.08	0.377147	2.03E-05
rs75841618	rs16870805	chr6:93834075	0.0150205	1	EPHA7	ENSG00000135333.13	Lung	G=0.92	T=0.08	0.377147	2.03E-05
rs75841618	rs150304721	chr6:93835801	0.01542934	1	EPHA7	ENSG00000135333.13	Lung	A=0.918	-=0.082	0.361316	2.04E-05
rs75841618	rs16870855	chr6:93841440	0.0152247	1	EPHA7	ENSG00000135333.13	Lung	C=0.919	T=0.081	0.4288	2.14E-05
rs75841618	rs16880183	chr6:93889701	0.03137644	0.95908074	EPHA7	ENSG00000135333.13	Testis	A=0.836	G=0.164	0.353058	2.24E-05
rs75841618	rs7772178	chr6:93849608	0.02310333	1	EPHA7	ENSG00000135333.13	Testis	C=0.883	T=0.117	0.425527	2.47E-05
rs75841618	rs6923008	chr6:93887045	0.03137644	0.95908074	EPHA7	ENSG00000135333.13	Testis	G=0.836	T=0.164	0.351036	2.54E-05
rs75841618	rs60592958	chr6:93887933	0.03137644	0.95908074	EPHA7	ENSG00000135333.13	Testis	C=0.836	T=0.164	0.351036	2.54E-05
rs75841618	rs73758259	chr6:93828724	0.01563442	1	EPHA7	ENSG00000135333.13	Lung	G=0.917	T=0.083	0.354112	2.72E-05
rs75841618	rs16870796	chr6:93828781	0.0150205	1	EPHA7	ENSG00000135333.13	Lung	T=0.92	G=0.08	0.369069	2.76E-05
rs75841618	rs72928577	chr6:93829400	0.0152247	1	EPHA7	ENSG00000135333.13	Lung	C=0.919	T=0.081	0.369069	2.76E-05
rs75841618	rs117717826	chr6:94036945	0.00443074	1	EPHA7	ENSG00000135333.13	Heart - Left Ventricle	T=0.975	A=0.025	-0.389622	2.96E-05
rs75841618	rs56378615	chr6:93900091	0.02476734	0.90876111	EPHA7	ENSG00000135333.13	Testis	C=0.853	T=0.147	0.358985	3.02E-05
rs75841618	rs71558449	chr6:93828589	0.00589667	1	EPHA7	ENSG00000135333.13	Esophagus - Muscularis	G=0.967	A=0.033	0.272458	3.14E-05
rs75841618	rs6922405	chr6:93842847	0.0152247	1	EPHA7	ENSG00000135333.13	Lung	T=0.919	C=0.081	0.370721	3.25E-05
rs75841618	rs72930416	chr6:93844150	0.0152247	1	EPHA7	ENSG00000135333.13	Lung	T=0.919	C=0.081	0.370721	3.25E-05
rs75841618	rs16870846	chr6:93840909	0.0152247	1	EPHA7	ENSG00000135333.13	Lung	C=0.919	G=0.081	0.371767	3.40E-05
rs75841618	rs1319460	chr6:93846637	0.05447383	1	EPHA7	ENSG00000135333.13	Minor Salivary Gland	C=0.239	T=0.761	0.230876	3.54E-05
rs75841618	rs538751	chr6:93848400	0.05447383	1	EPHA7	ENSG00000135333.13	Minor Salivary Gland	A=0.239	C=0.761	0.230876	3.54E-05
rs75841618	rs11422146	chr6:93849652	0.05417613	1	EPHA7	ENSG00000135333.13	Minor Salivary Gland	-=0.238	T=0.762	0.230876	3.54E-05
rs75841618	rs473900	chr6:93931037	0.03724721	0.85258345	EPHA7	ENSG00000135333.13	Adipose - Subcutaneous	T=0.228	C=0.772	0.223628	3.56E-05
rs75841618	rs369196	chr6:93932634	0.03724721	0.85258345	EPHA7	ENSG00000135333.13	Adipose - Subcutaneous	C=0.228	T=0.772	0.223628	3.56E-05
rs75841618	rs1319460	chr6:93846637	0.05447383	1	EPHA7	ENSG00000135333.13	Testis	C=0.239	T=0.761	-0.330556	3.66E-05
rs75841618	rs538751	chr6:93848400	0.05447383	1	EPHA7	ENSG00000135333.13	Testis	A=0.239	C=0.761	-0.330556	3.66E-05
rs75841618	rs11422146	chr6:93849652	0.05417613	1	EPHA7	ENSG00000135333.13	Testis	-=0.238	T=0.762	-0.330556	3.66E-05
rs75841618	rs6935219	chr6:93889277	0.03137644	0.95908074	EPHA7	ENSG00000135333.13	Testis	A=0.836	G=0.164	0.33974	4.29E-05
rs75841618	rs140182812	chr6:93804212	0.00139369	1	EPHA7	ENSG00000135333.13	Adipose - Subcutaneous	G=0.992	A=0.008	-0.729088	4.39E-05
rs75841618	rs10686381	chr6:93887552	0.03137644	0.95908074	EPHA7	ENSG00000135333.13	Testis	-=0.836	AAGT=0.164	0.337644	4.84E-05
rs75841618	rs12530331	chr6:93830460	0.0152247	1	EPHA7	ENSG00000135333.13	Lung	G=0.919	C=0.081	0.35653	5.33E-05
rs75841618	rs74468395	chr6:94017659	0.00589667	1	EPHA7	ENSG00000135333.13	Heart - Left Ventricle	C=0.967	T=0.033	-0.353	6.19E-05
rs75841618	rs117256127	chr6:93852714	0.00424918	1	EPHA7	ENSG00000135333.13	Adipose - Subcutaneous	T=0.976	C=0.024	-0.674139	6.42E-05
rs75841618	rs117425371	chr6:93852717	0.00424918	1	EPHA7	ENSG00000135333.13	Adipose - Subcutaneous	A=0.976	G=0.024	-0.674139	6.42E-05
rs75841618	rs145519743	chr6:93884032	0.00443074	1	EPHA7	ENSG00000135333.13	Adipose - Subcutaneous	G=0.975	A=0.025	-0.674139	6.42E-05
rs75841618	rs2780662	chr6:93920660	0.05119093	0.85884994	EPHA7	ENSG00000135333.13	Testis	T=0.285	C=0.715	-0.29987	6.44E-05
rs75841618	rs9351349	chr6:93832104	0.02984058	0.88747204	EPHA7	ENSG00000135333.13	Adipose - Subcutaneous	G=0.179	A=0.821	0.228214	6.48E-05
rs75841618	rs147330775	chr6:93915683	0.00104317	1	EPHA7	ENSG00000135333.13	Skin - Sun Exposed (Lower leg)	C=0.994	T=0.006	-0.47159	6.56E-05
rs75841618	rs117109139	chr6:93861398	0.00682517	1	EPHA7	ENSG00000135333.13	Esophagus - Muscularis	C=0.962	G=0.038	0.257424	7.15E-05
rs75841618	rs650711	chr6:93842280	0.02984058	0.88747204	EPHA7	ENSG00000135333.13	Adipose - Subcutaneous	T=0.821	G=0.179	-0.226702	7.44E-05
rs75841618	rs768382	chr6:93826168	0.02959694	0.88684339	EPHA7	ENSG00000135333.13	Adipose - Subcutaneous	T=0.178	C=0.822	0.223191	7.87E-05
rs75841618	rs412388	chr6:93916878	0.05119093	0.85884994	EPHA7	ENSG00000135333.13	Testis	G=0.285	A=0.715	-0.296773	8.64E-05
rs75841618	rs1324110	chr6:93913200	0.1551874	0.83299737	EPHA7	ENSG00000135333.13	Heart - Atrial Appendage	G=0.563	C=0.437	0.0819979	9.78E-05
rs75841618	rs138022068	chr6:94069111	0.00643297	0.85934004	EPHA7	ENSG00000135333.13	Heart - Left Ventricle	C=0.952	-=0.048	-0.300501	0.00011172
rs75841618	rs11970583	chr6:93895756	0.04367934	0.80709492	EPHA7	ENSG00000135333.13	Esophagus - Muscularis	C=0.722	T=0.278	0.0990772	0.00011475
rs75841618	rs2065582	chr6:93896309	0.04367934	0.80709492	EPHA7	ENSG00000135333.13	Esophagus - Muscularis	G=0.722	T=0.278	0.0990772	0.00011475
rs75841618	rs2147225	chr6:93898127	0.04367934	0.80709492	EPHA7	ENSG00000135333.13	Esophagus - Muscularis	T=0.722	A=0.278	0.0990772	0.00011475
rs75841618	rs13205265	chr6:93898896	0.04367934	0.80709492	EPHA7	ENSG00000135333.13	Esophagus - Muscularis	C=0.722	T=0.278	0.0990772	0.00011475
rs75841618	rs35480101	chr6:93899371	0.04338904	0.8064035	EPHA7	ENSG00000135333.13	Esophagus - Muscularis	G=0.723	T=0.277	0.0990772	0.00011475
rs75841618	rs145046790	chr6:93899520	0.04367934	0.80709492	EPHA7	ENSG00000135333.13	Esophagus - Muscularis	AT=0.722	-=0.278	0.0990772	0.00011475
rs75841618	rs192604147	chr6:94240629	0.00156947	1	EPHA7	ENSG00000135333.13	Heart - Left Ventricle	A=0.991	G=0.009	-0.514265	0.00011598
rs75841618	rs9345321	chr6:93789199	0.0454394	0.81114188	EPHA7	ENSG00000135333.13	Esophagus - Mucosa	T=0.716	G=0.284	-0.209017	0.00012891

rs75841618	rs9345322	chr6:93790565	0.0454394	0.81114188	EPHA7	ENSG00000135333.13	Esophagus - Mucosa	A=0.716	G=0.284	-0.209017	0.00012891
rs75841618	rs9353982	chr6:93802038	0.0454394	0.81114188	EPHA7	ENSG00000135333.13	Esophagus - Mucosa	G=0.716	A=0.284	-0.209017	0.00012891
rs75841618	rs16880179	chr6:93889506	0.02684937	0.95375563	EPHA7	ENSG00000135333.13	Testis	G=0.855	A=0.145	0.335079	0.00013631
rs75841618	rs59810007	chr6:93891733	0.02684937	0.95375563	EPHA7	ENSG00000135333.13	Testis	T=0.855	C=0.145	0.335079	0.00013631
rs75841618	rs6901961	chr6:93892700	0.02684937	0.95375563	EPHA7	ENSG00000135333.13	Testis	T=0.855	A=0.145	0.335079	0.00013631
rs75841618	rs60804851	chr6:93894513	0.02684937	0.95375563	EPHA7	ENSG00000135333.13	Testis	C=0.855	G=0.145	0.335079	0.00013631
rs75841618	rs6901870	chr6:93895903	0.02684937	0.95375563	EPHA7	ENSG00000135333.13	Testis	A=0.855	C=0.145	0.335079	0.00013631
rs75841618	rs6901827	chr6:93895828	0.04367934	0.80709492	EPHA7	ENSG00000135333.13	Esophagus - Muscularis	A=0.722	G=0.278	0.0976714	0.00014821
rs75841618	rs2181805	chr6:93898026	0.04367934	0.80709492	EPHA7	ENSG00000135333.13	Esophagus - Muscularis	T=0.722	C=0.278	0.0976714	0.00014821
rs75841618	rs2631562	chr6:93920850	0.05088319	0.85835641	EPHA7	ENSG00000135333.13	Testis	T=0.284	C=0.716	-0.28302	0.00014886
rs75841618	rs143801031	chr6:93750441	0.00298853	1	EPHA7	ENSG00000135333.13	Adipose - Subcutaneous	T=0.983	C=0.017	-0.536087	0.00015072
rs75841618	rs58205228	chr6:93778613	0.0454394	0.81114188	EPHA7	ENSG00000135333.13	Esophagus - Mucosa	A=0.716	G=0.284	-0.206708	0.00015126
rs75841618	rs9342350	chr6:93798264	0.0454394	0.81114188	EPHA7	ENSG00000135333.13	Esophagus - Mucosa	A=0.716	G=0.284	-0.206708	0.00015126
rs75841618	rs9363029	chr6:93798520	0.0454394	0.81114188	EPHA7	ENSG00000135333.13	Esophagus - Mucosa	T=0.716	G=0.284	-0.206708	0.00015126
rs75841618	rs9345324	chr6:93803037	0.0454394	0.81114188	EPHA7	ENSG00000135333.13	Esophagus - Mucosa	C=0.716	A=0.284	-0.206708	0.00015126
rs75841618	rs6922792	chr6:93887222	0.02684937	0.95375563	EPHA7	ENSG00000135333.13	Testis	A=0.855	G=0.145	0.333048	0.00015245
rs75841618	rs117365486	chr6:93820610	0.00571211	1	EPHA7	ENSG00000135333.13	Nerve - Tibial	C=0.968	T=0.032	-0.404506	0.000162
rs75841618	rs148086058	chr6:93820887	0.00571211	1	EPHA7	ENSG00000135333.13	Nerve - Tibial	C=0.968	A=0.032	-0.404506	0.000162
rs75841618	rs11966965	chr6:93890049	0.04338904	0.8064035	EPHA7	ENSG00000135333.13	Esophagus - Muscularis	G=0.723	T=0.277	0.096593	0.00016937
rs75841618	rs35373649	chr6:93891073	0.04338904	0.8064035	EPHA7	ENSG00000135333.13	Esophagus - Muscularis	A=0.723	G=0.277	0.096593	0.00016937
rs75841618	rs6921600	chr6:93892299	0.04367934	0.80709492	EPHA7	ENSG00000135333.13	Esophagus - Muscularis	G=0.722	T=0.278	0.096593	0.00016937
rs75841618	rs6901416	chr6:93892412	0.04367934	0.80709492	EPHA7	ENSG00000135333.13	Esophagus - Muscularis	T=0.722	G=0.278	0.096593	0.00016937
rs75841618	rs9345323	chr6:93797607	0.0454394	0.81114188	EPHA7	ENSG00000135333.13	Esophagus - Mucosa	C=0.716	T=0.284	-0.204207	0.00017144
rs75841618	rs1535833	chr6:93769380	0.04367934	0.80709492	EPHA7	ENSG00000135333.13	Esophagus - Mucosa	C=0.722	T=0.278	-0.205304	0.00017505
rs75841618	rs77898219	chr6:93824234	0.04367934	0.80709492	EPHA7	ENSG00000135333.13	Esophagus - Mucosa	C=0.722	-=0.278	-0.207398	0.00017867
rs75841618	rs9363030	chr6:93806040	0.04397051	0.80778141	EPHA7	ENSG00000135333.13	Esophagus - Mucosa	G=0.721	A=0.279	-0.206095	0.00019318
rs75841618	rs1953145	chr6:93890592	0.04338904	0.8064035	EPHA7	ENSG00000135333.13	Esophagus - Muscularis	G=0.723	A=0.277	0.0952131	0.00021698
rs75841618	rs12110542	chr6:93894263	0.04367934	0.80709492	EPHA7	ENSG00000135333.13	Esophagus - Muscularis	T=0.722	G=0.278	0.0952131	0.00021698
rs75841618	rs1953146	chr6:93897891	0.04367934	0.80709492	EPHA7	ENSG00000135333.13	Esophagus - Muscularis	C=0.722	G=0.278	0.0952131	0.00021698
rs75841618	rs182357880	chr6:93879803	0.00121825	1	EPHA7	ENSG00000135333.13	Skin - Sun Exposed (Lower leg)	T=0.993	C=0.007	-0.395624	0.00022421
rs75841618	rs144384339	chr6:93897863	0.00370671	1	EPHA7	ENSG00000135333.13	Skin - Sun Exposed (Lower leg)	G=0.979	A=0.021	-0.418007	0.00029351
rs76037606	rs117365486	chr6:93820610	0.00253266	1	EPHA7	ENSG00000135333.13	Adipose - Subcutaneous	C=0.968	T=0.032	-0.679264	8.33E-10
rs76037606	rs148086058	chr6:93820887	0.00253266	1	EPHA7	ENSG00000135333.13	Adipose - Subcutaneous	C=0.968	A=0.032	-0.679264	8.33E-10
rs76037606	rs11962709	chr6:93891195	0.00985203	1	EPHA7	ENSG00000135333.13	Esophagus - Muscularis	A=0.887	G=0.113	0.219885	7.45E-09
rs76037606	rs71540101	chr6:93892043	0.00985203	1	EPHA7	ENSG00000135333.13	Esophagus - Muscularis	AGGTATATC=0.887	-=0.113	0.219885	7.45E-09
rs76037606	rs35962225	chr6:93915766	0.01014541	1	EPHA7	ENSG00000135333.13	Esophagus - Muscularis	G=0.884	C=0.116	0.229888	1.18E-08
rs76037606	rs35762480	chr6:93906245	0.0100474	1	EPHA7	ENSG00000135333.13	Esophagus - Muscularis	G=0.885	C=0.115	0.216734	1.27E-08
rs76037606	rs189136709	chr6:93783798	0.00038505	1	EPHA7	ENSG00000135333.13	Brain - Anterior cingulate cortex (BA24)	G=0.995	A=0.005	1.35876	1.60E-08
rs76037606	rs11962003	chr6:93890071	0.01174419	1	EPHA7	ENSG00000135333.13	Esophagus - Muscularis	A=0.868	G=0.132	0.206691	1.76E-08
rs76037606	rs11966984	chr6:93890176	0.01174419	1	EPHA7	ENSG00000135333.13	Esophagus - Muscularis	G=0.868	A=0.132	0.206691	1.76E-08
rs76037606	rs34948845	chr6:93894820	0.01174419	1	EPHA7	ENSG00000135333.13	Esophagus - Muscularis	G=0.868	A=0.132	0.206691	1.76E-08
rs76037606	rs11966926	chr6:93897338	0.00985203	1	EPHA7	ENSG00000135333.13	Esophagus - Muscularis	A=0.887	G=0.113	0.215362	2.61E-08
rs76037606	rs72919018	chr6:93924471	0.0136905	0.80594136	EPHA7	ENSG00000135333.13	Adipose - Subcutaneous	G=0.785	C=0.215	0.32156	2.76E-08
rs76037606	rs12189899	chr6:93931092	0.0136905	0.80594136	EPHA7	ENSG00000135333.13	Adipose - Subcutaneous	C=0.785	T=0.215	0.32156	2.76E-08
rs76037606	rs12199960	chr6:93923853	0.01357942	0.80503876	EPHA7	ENSG00000135333.13	Adipose - Subcutaneous	A=0.786	C=0.214	0.32248	4.27E-08
rs76037606	rs11967483	chr6:93903691	0.0100474	1	EPHA7	ENSG00000135333.13	Esophagus - Muscularis	A=0.885	T=0.115	0.206895	4.64E-08
rs76037606	rs34832688	chr6:93891054	0.00985203	1	EPHA7	ENSG00000135333.13	Esophagus - Muscularis	A=0.887	C=0.113	0.205584	4.66E-08
rs76037606	rs72919020	chr6:93925349	0.0136905	0.80594136	EPHA7	ENSG00000135333.13	Adipose - Subcutaneous	G=0.785	C=0.215	0.32041	4.76E-08
rs76037606	rs12190544	chr6:93925959	0.0136905	0.80594136	EPHA7	ENSG00000135333.13	Adipose - Subcutaneous	G=0.785	A=0.215	0.32041	4.76E-08
rs76037606	rs12206216	chr6:93927822	0.01380189	0.80683564	EPHA7	ENSG00000135333.13	Adipose - Subcutaneous	A=0.784	G=0.216	0.32041	4.76E-08
rs76037606	rs12196073	chr6:93928926	0.0136905	0.80594136	EPHA7	ENSG00000135333.13	Adipose - Subcutaneous	C=0.785	G=0.215	0.32041	4.76E-08

rs76037606	rs12196420	chr6:93929507	0.0136905	0.80594136	EPHA7	ENSG00000135333.13	Adipose - Subcutaneous	C=0.785	T=0.215	0.32041	4.76E-08
rs76037606	rs12201744	chr6:93930642	0.01357942	0.80503876	EPHA7	ENSG00000135333.13	Adipose - Subcutaneous	A=0.786	T=0.214	0.32041	4.76E-08
rs76037606	rs12195545	chr6:93930755	0.01357942	0.80503876	EPHA7	ENSG00000135333.13	Adipose - Subcutaneous	T=0.786	G=0.214	0.32041	4.76E-08
rs76037606	rs143708097	chr6:93931051	0.01357942	0.80503876	EPHA7	ENSG00000135333.13	Adipose - Subcutaneous	-=0.786	CCTA=0.214	0.32041	4.76E-08
rs76037606	rs72919042	chr6:93935578	0.0136905	0.80594136	EPHA7	ENSG00000135333.13	Adipose - Subcutaneous	C=0.785	T=0.215	0.32041	4.76E-08
rs76037606	rs13212701	chr6:93902787	0.0100474	1	EPHA7	ENSG00000135333.13	Esophagus - Muscularis	T=0.885	C=0.115	0.206435	4.99E-08
rs76037606	rs6936693	chr6:93903686	0.0100474	1	EPHA7	ENSG00000135333.13	Esophagus - Muscularis	T=0.885	C=0.115	0.202265	5.80E-08
rs76037606	rs12374628	chr6:93934768	0.0136905	0.80594136	EPHA7	ENSG00000135333.13	Adipose - Subcutaneous	T=0.785	C=0.215	0.313163	6.59E-08
rs76037606	rs388461	chr6:93918959	0.01014541	1	EPHA7	ENSG00000135333.13	Esophagus - Muscularis	A=0.884	G=0.116	0.191263	7.48E-08
rs76037606	rs575590	chr6:93920650	0.0100474	1	EPHA7	ENSG00000135333.13	Esophagus - Muscularis	G=0.885	A=0.115	0.191263	7.48E-08
rs76037606	rs72919047	chr6:93941158	0.0136905	0.80594136	EPHA7	ENSG00000135333.13	Adipose - Subcutaneous	T=0.785	C=0.215	0.313468	9.08E-08
rs76037606	rs12209980	chr6:93943432	0.0136905	0.80594136	EPHA7	ENSG00000135333.13	Adipose - Subcutaneous	T=0.785	G=0.215	0.313468	9.08E-08
rs76037606	rs12200456	chr6:93924735	0.01380189	0.80683564	EPHA7	ENSG00000135333.13	Adipose - Subcutaneous	A=0.784	G=0.216	0.313602	9.70E-08
rs76037606	rs72919045	chr6:93940859	0.0136905	0.80594136	EPHA7	ENSG00000135333.13	Adipose - Subcutaneous	T=0.785	A=0.215	0.315063	9.80E-08
rs76037606	rs6931234	chr6:93927181	0.01380189	0.80683564	EPHA7	ENSG00000135333.13	Adipose - Subcutaneous	T=0.784	C=0.216	0.299008	1.06E-07
rs76037606	rs13202762	chr6:93937080	0.00785232	1	EPHA7	ENSG00000135333.13	Esophagus - Muscularis	T=0.908	G=0.092	0.222914	1.15E-07
rs76037606	rs10693258	chr6:93902052	0.01024365	1	EPHA7	ENSG00000135333.13	Esophagus - Muscularis	-=0.883	TTG=0.117	0.174304	5.19E-07
rs76037606	rs13212701	chr6:93902787	0.0100474	1	EPHA7	ENSG00000135333.13	Esophagus - Mucosa	T=0.885	C=0.115	0.405746	1.63E-06
rs76037606	rs35762480	chr6:93906245	0.0100474	1	EPHA7	ENSG00000135333.13	Esophagus - Mucosa	G=0.885	C=0.115	0.407424	1.79E-06
rs76037606	rs117376030	chr6:94203683	0.00204519	1	EPHA7	ENSG00000135333.13	Esophagus - Gastroesophageal Junction	T=0.974	A=0.026	-0.384156	2.51E-06
rs76037606	rs72928597	chr6:93839843	0.00675039	1	EPHA7	ENSG00000135333.13	Lung	A=0.919	G=0.081	0.42955	4.55E-06
rs76037606	rs16870853	chr6:93841320	0.00675039	1	EPHA7	ENSG00000135333.13	Lung	T=0.919	C=0.081	0.42955	4.55E-06
rs76037606	rs1930933	chr6:93842544	0.02992801	0.95358066	EPHA7	ENSG00000135333.13	Minor Salivary Gland	C=0.299	T=0.701	0.246092	4.56E-06
rs76037606	rs142723671	chr6:94076414	0.00172351	1	EPHA7	ENSG00000135333.13	Heart - Left Ventricle	G=0.978	C=0.022	-0.495017	4.73E-06
rs76037606	rs11962709	chr6:93891195	0.00985203	1	EPHA7	ENSG00000135333.13	Esophagus - Mucosa	A=0.887	G=0.113	0.385284	6.17E-06
rs76037606	rs71540101	chr6:93892043	0.00985203	1	EPHA7	ENSG00000135333.13	Esophagus - Mucosa	AGGTATATC=0.887	-=0.113	0.385284	6.17E-06
rs76037606	rs147330775	chr6:93915683	0.00046253	1	EPHA7	ENSG00000135333.13	Brain - Anterior cingulate cortex (BA24)	C=0.994	T=0.006	0.808887	6.34E-06
rs76037606	rs6928892	chr6:93888220	0.09285406	0.90654032	EPHA7	ENSG00000135333.13	Esophagus - Muscularis	A=0.594	G=0.406	0.107385	6.55E-06
rs76037606	rs16870793	chr6:93828599	0.00656951	1	EPHA7	ENSG00000135333.13	Lung	G=0.921	A=0.079	0.419095	6.62E-06
rs76037606	rs72928592	chr6:93837959	0.00665985	1	EPHA7	ENSG00000135333.13	Lung	T=0.92	C=0.08	0.419095	6.62E-06
rs76037606	rs10693258	chr6:93902052	0.01024365	1	EPHA7	ENSG00000135333.13	Esophagus - Mucosa	-=0.883	TTG=0.117	0.343667	6.63E-06
rs76037606	rs34832688	chr6:93891054	0.00985203	1	EPHA7	ENSG00000135333.13	Esophagus - Mucosa	A=0.887	C=0.113	0.378517	6.87E-06
rs76037606	rs1575540	chr6:93836577	0.00471634	1	EPHA7	ENSG00000135333.13	Esophagus - Muscularis	G=0.942	A=0.058	0.272463	7.68E-06
rs76037606	rs544639807	chr6:93883657	0.00061794	1	EPHA7	ENSG00000135333.13	Minor Salivary Gland	G=0.992	A=0.008	-1.34812	8.25E-06
rs76037606	rs36114673	chr6:93847056	0.00506297	1	EPHA7	ENSG00000135333.13	Esophagus - Muscularis	A=0.938	T=0.062	0.268662	8.26E-06
rs76037606	rs7776099	chr6:93913423	0.01053673	0.83165997	EPHA7	ENSG00000135333.13	Testis	C=0.835	A=0.165	0.3716	8.26E-06
rs76037606	rs7758242	chr6:93913435	0.01053673	0.83165997	EPHA7	ENSG00000135333.13	Testis	T=0.835	A=0.165	0.3716	8.26E-06
rs76037606	rs66765303	chr6:93913692	0.01053673	0.83165997	EPHA7	ENSG00000135333.13	Testis	A=0.835	G=0.165	0.3716	8.26E-06
rs76037606	rs66522431	chr6:93913825	0.01053673	0.83165997	EPHA7	ENSG00000135333.13	Testis	G=0.835	A=0.165	0.3716	8.26E-06
rs76037606	rs62414181	chr6:93913909	0.01053673	0.83165997	EPHA7	ENSG00000135333.13	Testis	A=0.835	G=0.165	0.3716	8.26E-06
rs76037606	rs62414182	chr6:93914022	0.01053673	0.83165997	EPHA7	ENSG00000135333.13	Testis	A=0.835	G=0.165	0.3716	8.26E-06
rs76037606	rs113888577	chr6:93698093	0.00093064	1	EPHA7	ENSG00000135333.13	Brain - Anterior cingulate cortex (BA24)	C=0.988	T=0.012	0.66365	8.36E-06
rs76037606	rs16870780	chr6:93826505	0.00665985	1	EPHA7	ENSG00000135333.13	Lung	T=0.92	A=0.08	0.385133	9.62E-06
rs76037606	rs1408285	chr6:93893666	0.0924402	0.90638377	EPHA7	ENSG00000135333.13	Esophagus - Muscularis	A=0.593	C=0.407	0.105615	9.88E-06
rs76037606	rs7761955	chr6:93896624	0.0924402	0.90638377	EPHA7	ENSG00000135333.13	Esophagus - Muscularis	T=0.593	C=0.407	0.105615	9.88E-06
rs76037606	rs7772899	chr6:93898633	0.0924402	0.90638377	EPHA7	ENSG00000135333.13	Esophagus - Muscularis	T=0.593	C=0.407	0.105615	9.88E-06
rs76037606	rs1590384	chr6:93837434	0.02992801	0.95358066	EPHA7	ENSG00000135333.13	Minor Salivary Gland	C=0.701	G=0.299	-0.24131	1.00E-05
rs76037606	rs1924474	chr6:93837991	0.02992801	0.95358066	EPHA7	ENSG00000135333.13	Minor Salivary Gland	C=0.701	T=0.299	-0.24131	1.00E-05
rs76037606	rs10944652	chr6:93838348	0.02992801	0.95358066	EPHA7	ENSG00000135333.13	Minor Salivary Gland	C=0.701	G=0.299	-0.24131	1.00E-05
rs76037606	rs12204186	chr6:93838665	0.02992801	0.95358066	EPHA7	ENSG00000135333.13	Minor Salivary Gland	C=0.701	T=0.299	-0.24131	1.00E-05
rs76037606	rs633279	chr6:93840705	0.02992801	0.95358066	EPHA7	ENSG00000135333.13	Minor Salivary Gland	A=0.299	T=0.701	0.24131	1.00E-05

rs76037606	rs634060	chr6:93840849	0.02992801	0.95358066	EPHA7	ENSG00000135333.13	Minor Salivary Gland	A=0.299	G=0.701	0.24131	1.00E-05
rs76037606	rs568957	chr6:93840855	0.02992801	0.95358066	EPHA7	ENSG00000135333.13	Minor Salivary Gland	G=0.299	T=0.701	0.24131	1.00E-05
rs76037606	rs34544163	chr6:93841565	0.02992801	0.95358066	EPHA7	ENSG00000135333.13	Minor Salivary Gland	-=0.299	A=0.701	0.24131	1.00E-05
rs76037606	rs562379	chr6:93841592	0.02992801	0.95358066	EPHA7	ENSG00000135333.13	Minor Salivary Gland	G=0.299	A=0.701	0.24131	1.00E-05
rs76037606	rs560731	chr6:93841734	0.02992801	0.95358066	EPHA7	ENSG00000135333.13	Minor Salivary Gland	A=0.299	G=0.701	0.24131	1.00E-05
rs76037606	rs650642	chr6:93842232	0.02992801	0.95358066	EPHA7	ENSG00000135333.13	Minor Salivary Gland	G=0.299	T=0.701	0.24131	1.00E-05
rs76037606	rs650747	chr6:93842298	0.02992801	0.95358066	EPHA7	ENSG00000135333.13	Minor Salivary Gland	T=0.299	C=0.701	0.24131	1.00E-05
rs76037606	rs1930934	chr6:93842533	0.02992801	0.95358066	EPHA7	ENSG00000135333.13	Minor Salivary Gland	A=0.299	G=0.701	0.24131	1.00E-05
rs76037606	rs9353987	chr6:93843588	0.02992801	0.95358066	EPHA7	ENSG00000135333.13	Minor Salivary Gland	A=0.299	G=0.701	0.24131	1.00E-05
rs76037606	rs9363034	chr6:93844136	0.02992801	0.95358066	EPHA7	ENSG00000135333.13	Minor Salivary Gland	G=0.299	A=0.701	0.24131	1.00E-05
rs76037606	rs9353989	chr6:93844383	0.02992801	0.95358066	EPHA7	ENSG00000135333.13	Minor Salivary Gland	G=0.299	T=0.701	0.24131	1.00E-05
rs76037606	rs74822095	chr6:93741512	0.00386244	1	EPHA7	ENSG00000135333.13	Adipose - Subcutaneous	C=0.952	T=0.048	-0.476757	1.06E-05
rs76037606	rs76282783	chr6:93576050	0.0033586	1	EPHA7	ENSG00000135333.13	Lung	C=0.958	A=0.042	0.702179	1.18E-05
rs76037606	rs10693258	chr6:93902052	0.01024365	1	EPHA7	ENSG00000135333.13	Esophagus - Gastroesophageal Junction	-=0.883	TTG=0.117	0.189047	1.21E-05
rs76037606	rs6936693	chr6:93903686	0.0100474	1	EPHA7	ENSG00000135333.13	Esophagus - Mucosa	T=0.885	C=0.115	0.361801	1.30E-05
rs76037606	rs11967483	chr6:93903691	0.0100474	1	EPHA7	ENSG00000135333.13	Esophagus - Mucosa	A=0.885	T=0.115	0.36623	1.31E-05
rs76037606	rs72926533	chr6:93740212	0.00069588	1	EPHA7	ENSG00000135333.13	Minor Salivary Gland	C=0.991	T=0.009	-1.15096	1.32E-05
rs76037606	rs188317446	chr6:93743394	0.00069588	1	EPHA7	ENSG00000135333.13	Minor Salivary Gland	G=0.991	C=0.009	-1.15096	1.32E-05
rs76037606	rs72926535	chr6:93747452	0.00069588	1	EPHA7	ENSG00000135333.13	Minor Salivary Gland	C=0.991	T=0.009	-1.15096	1.32E-05
rs76037606	rs117415227	chr6:93735745	0.00386244	1	EPHA7	ENSG00000135333.13	Adipose - Subcutaneous	T=0.952	G=0.048	-0.47253	1.37E-05
rs76037606	rs11966926	chr6:93897338	0.00985203	1	EPHA7	ENSG00000135333.13	Esophagus - Mucosa	A=0.887	G=0.113	0.374015	1.38E-05
rs76037606	rs199888991	chr6:93784078	0.00038505	1	EPHA7	ENSG00000135333.13	Adipose - Subcutaneous	-=0.995	T=0.005	-0.796053	1.44E-05
rs76037606	rs111429740	chr6:93838453	0.00675039	1	EPHA7	ENSG00000135333.13	Lung	G=0.919	A=0.081	0.383525	1.55E-05
rs76037606	rs72919045	chr6:93940859	0.0136905	0.80594136	EPHA7	ENSG00000135333.13	Breast - Mammary Tissue	T=0.785	A=0.215	0.313719	1.71E-05
rs76037606	rs16870789	chr6:93827963	0.00665985	1	EPHA7	ENSG00000135333.13	Lung	C=0.92	T=0.08	0.374368	1.81E-05
rs76037606	rs145810085	chr6:93833819	0.00061794	1	EPHA7	ENSG00000135333.13	Adipose - Subcutaneous	G=0.992	A=0.008	-0.735109	1.88E-05
rs76037606	rs7774823	chr6:93830545	0.02962575	0.95327016	EPHA7	ENSG00000135333.13	Minor Salivary Gland	G=0.703	A=0.297	-0.232136	1.89E-05
rs76037606	rs1951907	chr6:93831192	0.02962575	0.95327016	EPHA7	ENSG00000135333.13	Minor Salivary Gland	T=0.703	C=0.297	-0.232136	1.89E-05
rs76037606	rs35306488	chr6:93832227	0.02917557	0.95279655	EPHA7	ENSG00000135333.13	Minor Salivary Gland	-=0.706	T=0.294	-0.232136	1.89E-05
rs76037606	rs10944648	chr6:93832280	0.02962575	0.95327016	EPHA7	ENSG00000135333.13	Minor Salivary Gland	A=0.703	G=0.297	-0.232136	1.89E-05
rs76037606	rs7751375	chr6:93832923	0.02977667	0.95342593	EPHA7	ENSG00000135333.13	Minor Salivary Gland	C=0.702	A=0.298	-0.232136	1.89E-05
rs76037606	rs7757292	chr6:93834165	0.02962575	0.95327016	EPHA7	ENSG00000135333.13	Minor Salivary Gland	C=0.703	A=0.297	-0.232136	1.89E-05
rs76037606	rs11962709	chr6:93891195	0.00985203	1	EPHA7	ENSG00000135333.13	Esophagus - Gastroesophageal Junction	A=0.887	G=0.113	0.198094	1.91E-05
rs76037606	rs71540101	chr6:93892043	0.00985203	1	EPHA7	ENSG00000135333.13	Esophagus - Gastroesophageal Junction	AGGTATATC=0.887	-=0.113	0.198094	1.91E-05
rs76037606	rs35762480	chr6:93906245	0.0100474	1	EPHA7	ENSG00000135333.13	Esophagus - Gastroesophageal Junction	G=0.885	C=0.115	0.19742	1.95E-05
rs76037606	rs16870850	chr6:93841079	0.00711451	1	EPHA7	ENSG00000135333.13	Lung	T=0.916	C=0.084	0.361979	2.02E-05
rs76037606	rs12528846	chr6:93828842	0.00665985	1	EPHA7	ENSG00000135333.13	Lung	A=0.92	G=0.08	0.377147	2.03E-05
rs76037606	rs12527296	chr6:93829143	0.00665985	1	EPHA7	ENSG00000135333.13	Lung	T=0.92	C=0.08	0.377147	2.03E-05
rs76037606	rs72928575	chr6:93829399	0.00675039	1	EPHA7	ENSG00000135333.13	Lung	G=0.919	T=0.081	0.377147	2.03E-05
rs76037606	rs72928579	chr6:93829658	0.00665985	1	EPHA7	ENSG00000135333.13	Lung	G=0.92	A=0.08	0.377147	2.03E-05
rs76037606	rs77007574	chr6:93829960	0.00665985	1	EPHA7	ENSG00000135333.13	Lung	T=0.92	C=0.08	0.377147	2.03E-05
rs76037606	rs72928582	chr6:93830015	0.00665985	1	EPHA7	ENSG00000135333.13	Lung	G=0.92	A=0.08	0.377147	2.03E-05
rs76037606	rs16870801	chr6:93830951	0.00665985	1	EPHA7	ENSG00000135333.13	Lung	T=0.92	C=0.08	0.377147	2.03E-05
rs76037606	rs16870805	chr6:93834075	0.00665985	1	EPHA7	ENSG00000135333.13	Lung	G=0.92	T=0.08	0.377147	2.03E-05
rs76037606	rs150304721	chr6:93835801	0.00684112	1	EPHA7	ENSG00000135333.13	Lung	A=0.918	-=0.082	0.361316	2.04E-05
rs76037606	rs11967483	chr6:93903691	0.0100474	1	EPHA7	ENSG00000135333.13	Esophagus - Gastroesophageal Junction	A=0.885	T=0.115	0.196042	2.11E-05
rs76037606	rs16870855	chr6:93841440	0.00675039	1	EPHA7	ENSG00000135333.13	Lung	C=0.919	T=0.081	0.4288	2.14E-05
rs76037606	rs13212701	chr6:93902787	0.0100474	1	EPHA7	ENSG00000135333.13	Esophagus - Gastroesophageal Junction	T=0.885	C=0.115	0.195748	2.16E-05
rs76037606	rs16880183	chr6:93889701	0.01512424	1	EPHA7	ENSG00000135333.13	Testis	A=0.836	G=0.164	0.353058	2.24E-05
rs76037606	rs12199960	chr6:93923853	0.01357942	0.80503876	EPHA7	ENSG00000135333.13	Breast - Mammary Tissue	A=0.786	C=0.214	0.305305	2.45E-05
rs76037606	rs12200456	chr6:93924735	0.01380189	0.80683564	EPHA7	ENSG00000135333.13	Breast - Mammary Tissue	A=0.784	G=0.216	0.305305	2.45E-05

rs76037606	rs72919020	chr6:93925349	0.0136905	0.80594136 EPHA7	ENSG00000135333.13	Breast - Mammary Tissue	G=0.785	C=0.215	0.305305	2.45E-05
rs76037606	rs12190544	chr6:93925959	0.0136905	0.80594136 EPHA7	ENSG00000135333.13	Breast - Mammary Tissue	G=0.785	A=0.215	0.305305	2.45E-05
rs76037606	rs12206216	chr6:93927822	0.01380189	0.80683564 EPHA7	ENSG00000135333.13	Breast - Mammary Tissue	A=0.784	G=0.216	0.305305	2.45E-05
rs76037606	rs12196073	chr6:93928926	0.0136905	0.80594136 EPHA7	ENSG00000135333.13	Breast - Mammary Tissue	C=0.785	G=0.215	0.305305	2.45E-05
rs76037606	rs12196420	chr6:93929507	0.0136905	0.80594136 EPHA7	ENSG00000135333.13	Breast - Mammary Tissue	C=0.785	T=0.215	0.305305	2.45E-05
rs76037606	rs12201744	chr6:93930642	0.01357942	0.80503876 EPHA7	ENSG00000135333.13	Breast - Mammary Tissue	A=0.786	T=0.214	0.305305	2.45E-05
rs76037606	rs12195545	chr6:93930755	0.01357942	0.80503876 EPHA7	ENSG00000135333.13	Breast - Mammary Tissue	T=0.786	G=0.214	0.305305	2.45E-05
rs76037606	rs143708097	chr6:93931051	0.01357942	0.80503876 EPHA7	ENSG00000135333.13	Breast - Mammary Tissue	-=0.786	CCTA=0.214	0.305305	2.45E-05
rs76037606	rs72919042	chr6:93935578	0.0136905	0.80594136 EPHA7	ENSG00000135333.13	Breast - Mammary Tissue	C=0.785	T=0.215	0.305305	2.45E-05
rs76037606	rs7772178	chr6:93849608	0.01024365	1 EPHA7	ENSG00000135333.13	Testis	C=0.883	T=0.117	0.425527	2.47E-05
rs76037606	rs72919047	chr6:93941158	0.0136905	0.80594136 EPHA7	ENSG00000135333.13	Breast - Mammary Tissue	T=0.785	C=0.215	0.304408	2.54E-05
rs76037606	rs12209980	chr6:93943432	0.0136905	0.80594136 EPHA7	ENSG00000135333.13	Breast - Mammary Tissue	T=0.785	G=0.215	0.304408	2.54E-05
rs76037606	rs6923008	chr6:93887045	0.01512424	1 EPHA7	ENSG00000135333.13	Testis	G=0.836	T=0.164	0.351036	2.54E-05
rs76037606	rs60592958	chr6:93887933	0.01512424	1 EPHA7	ENSG00000135333.13	Testis	C=0.836	T=0.164	0.351036	2.54E-05
rs76037606	rs147445521	chr6:93825169	0.00558845	1 EPHA7	ENSG00000135333.13	Lung	TGT=0.932	-=0.068	0.417524	2.57E-05
rs76037606	rs118177782	chr6:93767900	0.00204519	1 EPHA7	ENSG00000135333.13	Brain - Anterior cingulate cortex (BA24)	C=0.974	T=0.026	0.590137	2.71E-05
rs76037606	rs73758259	chr6:93828724	0.00693206	1 EPHA7	ENSG00000135333.13	Lung	G=0.917	T=0.083	0.354112	2.72E-05
rs76037606	rs16870796	chr6:93828781	0.00665985	1 EPHA7	ENSG00000135333.13	Lung	T=0.92	G=0.08	0.369069	2.76E-05
rs76037606	rs72928577	chr6:93829400	0.00675039	1 EPHA7	ENSG00000135333.13	Lung	C=0.919	T=0.081	0.369069	2.76E-05
rs76037606	rs11966926	chr6:93897338	0.00985203	1 EPHA7	ENSG00000135333.13	Esophagus - Gastroesophageal Junction	A=0.887	G=0.113	0.196345	2.79E-05
rs76037606	rs117717826	chr6:94036945	0.00196452	1 EPHA7	ENSG00000135333.13	Heart - Left Ventricle	T=0.975	A=0.025	-0.389622	2.96E-05
rs76037606	rs56378615	chr6:93900091	0.01090501	0.90559309 EPHA7	ENSG00000135333.13	Testis	C=0.853	T=0.147	0.358985	3.02E-05
rs76037606	rs6936693	chr6:93903686	0.0100474	1 EPHA7	ENSG00000135333.13	Esophagus - Gastroesophageal Junction	T=0.885	C=0.115	0.189133	3.05E-05
rs76037606	rs71558449	chr6:93828589	0.00261449	1 EPHA7	ENSG00000135333.13	Esophagus - Muscularis	G=0.967	A=0.033	0.272458	3.14E-05
rs76037606	rs6922405	chr6:93842847	0.00675039	1 EPHA7	ENSG00000135333.13	Lung	T=0.919	C=0.081	0.370721	3.25E-05
rs76037606	rs72930416	chr6:93844150	0.00675039	1 EPHA7	ENSG00000135333.13	Lung	T=0.919	C=0.081	0.370721	3.25E-05
rs76037606	rs34948845	chr6:93894820	0.01174419	1 EPHA7	ENSG00000135333.13	Esophagus - Gastroesophageal Junction	G=0.868	A=0.132	0.186343	3.27E-05
rs76037606	rs16870846	chr6:93840909	0.00675039	1 EPHA7	ENSG00000135333.13	Lung	C=0.919	G=0.081	0.371767	3.40E-05
rs76037606	rs1319460	chr6:93846637	0.02415283	1 EPHA7	ENSG00000135333.13	Minor Salivary Gland	C=0.239	T=0.761	0.230876	3.54E-05
rs76037606	rs538751	chr6:93848400	0.02415283	1 EPHA7	ENSG00000135333.13	Minor Salivary Gland	A=0.239	C=0.761	0.230876	3.54E-05
rs76037606	rs11422146	chr6:93849652	0.02402084	1 EPHA7	ENSG00000135333.13	Minor Salivary Gland	-=0.238	T=0.762	0.230876	3.54E-05
rs76037606	rs34832688	chr6:93891054	0.00985203	1 EPHA7	ENSG00000135333.13	Esophagus - Gastroesophageal Junction	A=0.887	C=0.113	0.187735	3.54E-05
rs76037606	rs1319460	chr6:93846637	0.02415283	1 EPHA7	ENSG00000135333.13	Testis	C=0.239	T=0.761	-0.330556	3.66E-05
rs76037606	rs538751	chr6:93848400	0.02415283	1 EPHA7	ENSG00000135333.13	Testis	A=0.239	C=0.761	-0.330556	3.66E-05
rs76037606	rs11422146	chr6:93849652	0.02402084	1 EPHA7	ENSG00000135333.13	Testis	-=0.238	T=0.762	-0.330556	3.66E-05
rs76037606	rs6931234	chr6:93927181	0.01380189	0.80683564 EPHA7	ENSG00000135333.13	Breast - Mammary Tissue	T=0.784	C=0.216	0.288667	3.69E-05
rs76037606	rs117415227	chr6:93735745	0.00386244	1 EPHA7	ENSG00000135333.13	Lung	T=0.952	G=0.048	-0.50856	4.07E-05
rs76037606	rs72919018	chr6:93924471	0.0136905	0.80594136 EPHA7	ENSG00000135333.13	Breast - Mammary Tissue	G=0.785	C=0.215	0.291651	4.19E-05
rs76037606	rs12189899	chr6:93931092	0.0136905	0.80594136 EPHA7	ENSG00000135333.13	Breast - Mammary Tissue	C=0.785	T=0.215	0.291651	4.19E-05
rs76037606	rs6935219	chr6:93889277	0.01512424	1 EPHA7	ENSG00000135333.13	Testis	A=0.836	G=0.164	0.33974	4.29E-05
rs76037606	rs12374628	chr6:93934768	0.0136905	0.80594136 EPHA7	ENSG00000135333.13	Breast - Mammary Tissue	T=0.785	C=0.215	0.290954	4.31E-05
rs76037606	rs11962003	chr6:93890071	0.01174419	1 EPHA7	ENSG00000135333.13	Esophagus - Mucosa	A=0.868	G=0.132	0.333682	4.39E-05
rs76037606	rs11966984	chr6:93890176	0.01174419	1 EPHA7	ENSG00000135333.13	Esophagus - Mucosa	G=0.868	A=0.132	0.333682	4.39E-05
rs76037606	rs140182812	chr6:93804212	0.00061794	1 EPHA7	ENSG00000135333.13	Adipose - Subcutaneous	G=0.992	A=0.008	-0.729088	4.39E-05
rs76037606	rs11962003	chr6:93890071	0.01174419	1 EPHA7	ENSG00000135333.13	Esophagus - Gastroesophageal Junction	A=0.868	G=0.132	0.183007	4.40E-05
rs76037606	rs11966984	chr6:93890176	0.01174419	1 EPHA7	ENSG00000135333.13	Esophagus - Gastroesophageal Junction	G=0.868	A=0.132	0.183007	4.40E-05
rs76037606	rs34948845	chr6:93894820	0.01174419	1 EPHA7	ENSG00000135333.13	Esophagus - Mucosa	G=0.868	A=0.132	0.334329	4.44E-05
rs76037606	rs10686381	chr6:93887552	0.01512424	1 EPHA7	ENSG00000135333.13	Testis	-=0.836	AAGT=0.164	0.337644	4.84E-05
rs76037606	rs2224853	chr6:93890865	0.04687736	0.93250134 EPHA7	ENSG00000135333.13	Esophagus - Muscularis	T=0.588	C=0.412	-0.0928827	5.11E-05
rs76037606	rs41273625	chr6:93952851	0.00515009	1 EPHA7	ENSG00000135333.13	Esophagus - Muscularis	C=0.937	G=0.063	0.224035	5.17E-05
rs76037606	rs78355320	chr6:93821638	0.00386244	1 EPHA7	ENSG00000135333.13	Adipose - Subcutaneous	T=0.952	A=0.048	-0.431835	5.33E-05

rs76037606	rs12530331	chr6:93830460	0.00675039	1	EPHA7	ENSG00000135333.13	Lung	G=0.919	C=0.081	0.35653	5.33E-05
rs76037606	rs72920784	chr6:93673551	0.00156365	1	EPHA7	ENSG00000135333.13	Adipose - Subcutaneous	A=0.98	G=0.02	-0.579253	5.40E-05
rs76037606	rs72920785	chr6:93675496	0.00156365	1	EPHA7	ENSG00000135333.13	Adipose - Subcutaneous	G=0.98	C=0.02	-0.579253	5.40E-05
rs76037606	rs74468395	chr6:94017659	0.00261449	1	EPHA7	ENSG00000135333.13	Heart - Left Ventricle	C=0.967	T=0.033	-0.353	6.19E-05
rs76037606	rs117256127	chr6:93852714	0.00188402	1	EPHA7	ENSG00000135333.13	Adipose - Subcutaneous	T=0.976	C=0.024	-0.674139	6.42E-05
rs76037606	rs117425371	chr6:93852717	0.00188402	1	EPHA7	ENSG00000135333.13	Adipose - Subcutaneous	A=0.976	G=0.024	-0.674139	6.42E-05
rs76037606	rs145519743	chr6:93884032	0.00196452	1	EPHA7	ENSG00000135333.13	Adipose - Subcutaneous	G=0.975	A=0.025	-0.674139	6.42E-05
rs76037606	rs9351349	chr6:93832104	0.01429205	0.92237654	EPHA7	ENSG00000135333.13	Adipose - Subcutaneous	G=0.179	A=0.821	0.228214	6.48E-05
rs76037606	rs147330775	chr6:93915683	0.00046253	1	EPHA7	ENSG00000135333.13	Skin - Sun Exposed (Lower leg)	C=0.994	T=0.006	-0.47159	6.56E-05
rs76037606	rs117109139	chr6:93861398	0.00302617	1	EPHA7	ENSG00000135333.13	Esophagus - Muscularis	C=0.962	G=0.038	0.257424	7.15E-05
rs76037606	rs650711	chr6:93842280	0.01429205	0.92237654	EPHA7	ENSG00000135333.13	Adipose - Subcutaneous	T=0.821	G=0.179	-0.226702	7.44E-05
rs76037606	rs768382	chr6:93826168	0.01418212	0.92194289	EPHA7	ENSG00000135333.13	Adipose - Subcutaneous	T=0.178	C=0.822	0.223191	7.87E-05
rs76037606	rs1324110	chr6:93913200	0.08054639	0.90125638	EPHA7	ENSG00000135333.13	Heart - Atrial Appendage	G=0.563	C=0.437	0.0819979	9.78E-05
rs76037606	rs41273625	chr6:93952851	0.00515009	1	EPHA7	ENSG00000135333.13	Esophagus - Gastroesophageal Junction	C=0.937	G=0.063	0.249826	0.00010545
rs76037606	rs11970583	chr6:93895756	0.02973083	1	EPHA7	ENSG00000135333.13	Esophagus - Muscularis	C=0.722	T=0.278	0.0990772	0.00011475
rs76037606	rs2065582	chr6:93896309	0.02973083	1	EPHA7	ENSG00000135333.13	Esophagus - Muscularis	G=0.722	T=0.278	0.0990772	0.00011475
rs76037606	rs2147225	chr6:93898127	0.02973083	1	EPHA7	ENSG00000135333.13	Esophagus - Muscularis	T=0.722	A=0.278	0.0990772	0.00011475
rs76037606	rs13205265	chr6:93898896	0.02973083	1	EPHA7	ENSG00000135333.13	Esophagus - Muscularis	C=0.722	T=0.278	0.0990772	0.00011475
rs76037606	rs35480101	chr6:93899371	0.0295839	1	EPHA7	ENSG00000135333.13	Esophagus - Muscularis	G=0.723	T=0.277	0.0990772	0.00011475
rs76037606	rs145046790	chr6:93899520	0.02973083	1	EPHA7	ENSG00000135333.13	Esophagus - Muscularis	AT=0.722	-=0.278	0.0990772	0.00011475
rs76037606	rs192604147	chr6:94240629	0.00069588	1	EPHA7	ENSG00000135333.13	Heart - Left Ventricle	A=0.991	G=0.009	-0.514265	0.00011598
rs76037606	rs1324103	chr6:93901016	0.04436088	0.89972089	EPHA7	ENSG00000135333.13	Esophagus - Muscularis	A=0.584	G=0.416	-0.0876121	0.00012747
rs76037606	rs1324104	chr6:93901538	0.04436088	0.89972089	EPHA7	ENSG00000135333.13	Esophagus - Muscularis	T=0.584	G=0.416	-0.0876121	0.00012747
rs76037606	rs16880179	chr6:93889506	0.013087	1	EPHA7	ENSG00000135333.13	Testis	G=0.855	A=0.145	0.335079	0.00013631
rs76037606	rs59810007	chr6:93891733	0.013087	1	EPHA7	ENSG00000135333.13	Testis	T=0.855	C=0.145	0.335079	0.00013631
rs76037606	rs6901961	chr6:93892700	0.013087	1	EPHA7	ENSG00000135333.13	Testis	T=0.855	A=0.145	0.335079	0.00013631
rs76037606	rs60804851	chr6:93894513	0.013087	1	EPHA7	ENSG00000135333.13	Testis	C=0.855	G=0.145	0.335079	0.00013631
rs76037606	rs6901870	chr6:93895903	0.013087	1	EPHA7	ENSG00000135333.13	Testis	A=0.855	C=0.145	0.335079	0.00013631
rs76037606	rs74822095	chr6:93741512	0.00386244	1	EPHA7	ENSG00000135333.13	Lung	C=0.952	T=0.048	-0.471073	0.00014377
rs76037606	rs6901827	chr6:93895828	0.02973083	1	EPHA7	ENSG00000135333.13	Esophagus - Muscularis	A=0.722	G=0.278	0.0976714	0.00014821
rs76037606	rs2181805	chr6:93898026	0.02973083	1	EPHA7	ENSG00000135333.13	Esophagus - Muscularis	T=0.722	C=0.278	0.0976714	0.00014821
rs76037606	rs71298717	chr6:93898609	0.02617495	1	EPHA7	ENSG00000135333.13	Esophagus - Muscularis	-=0.747	ATTTTAGGAATA=0.25	0.0976714	0.00014821
rs76037606	rs143801031	chr6:93750441	0.00132507	1	EPHA7	ENSG00000135333.13	Adipose - Subcutaneous	T=0.983	C=0.017	-0.536087	0.00015072
rs76037606	rs6922792	chr6:93887222	0.013087	1	EPHA7	ENSG00000135333.13	Testis	A=0.855	G=0.145	0.333048	0.00015245
rs76037606	rs117365486	chr6:93820610	0.00253266	1	EPHA7	ENSG00000135333.13	Nerve - Tibial	C=0.968	T=0.032	-0.404506	0.000162
rs76037606	rs148086058	chr6:93820887	0.00253266	1	EPHA7	ENSG00000135333.13	Nerve - Tibial	C=0.968	A=0.032	-0.404506	0.000162
rs76037606	rs11966965	chr6:93890049	0.0295839	1	EPHA7	ENSG00000135333.13	Esophagus - Muscularis	G=0.723	T=0.277	0.096593	0.00016937
rs76037606	rs35373649	chr6:93891073	0.0295839	1	EPHA7	ENSG00000135333.13	Esophagus - Muscularis	A=0.723	G=0.277	0.096593	0.00016937
rs76037606	rs6921600	chr6:93892299	0.02973083	1	EPHA7	ENSG00000135333.13	Esophagus - Muscularis	G=0.722	T=0.278	0.096593	0.00016937
rs76037606	rs6901416	chr6:93892412	0.02973083	1	EPHA7	ENSG00000135333.13	Esophagus - Muscularis	T=0.722	G=0.278	0.096593	0.00016937
rs76037606	rs6454946	chr6:93902683	0.04477275	0.90019841	EPHA7	ENSG00000135333.13	Esophagus - Muscularis	A=0.583	G=0.417	-0.0835518	0.00021296
rs76037606	rs1324106	chr6:93902867	0.04477275	0.90019841	EPHA7	ENSG00000135333.13	Esophagus - Muscularis	T=0.583	A=0.417	-0.0835518	0.00021296
rs76037606	rs1324107	chr6:93903050	0.04477275	0.90019841	EPHA7	ENSG00000135333.13	Esophagus - Muscularis	C=0.583	G=0.417	-0.0835518	0.00021296
rs76037606	rs658231	chr6:93903544	0.04477275	0.90019841	EPHA7	ENSG00000135333.13	Esophagus - Muscularis	C=0.583	T=0.417	-0.0835518	0.00021296
rs76037606	rs1953145	chr6:93890592	0.0295839	1	EPHA7	ENSG00000135333.13	Esophagus - Muscularis	G=0.723	A=0.277	0.0952131	0.00021698
rs76037606	rs12110542	chr6:93894263	0.02973083	1	EPHA7	ENSG00000135333.13	Esophagus - Muscularis	T=0.722	G=0.278	0.0952131	0.00021698
rs76037606	rs1953146	chr6:93897891	0.02973083	1	EPHA7	ENSG00000135333.13	Esophagus - Muscularis	C=0.722	G=0.278	0.0952131	0.00021698
rs76037606	rs182357880	chr6:93879803	0.00054015	1	EPHA7	ENSG00000135333.13	Skin - Sun Exposed (Lower leg)	T=0.993	C=0.007	-0.395624	0.00022421
rs76037606	rs144384339	chr6:93897863	0.0016435	1	EPHA7	ENSG00000135333.13	Skin - Sun Exposed (Lower leg)	G=0.979	A=0.021	-0.418007	0.00029351
rs76462658	rs117365486	chr6:93820610	0.00253266	1	EPHA7	ENSG00000135333.13	Adipose - Subcutaneous	C=0.968	T=0.032	-0.679264	8.33E-10
rs76462658	rs148086058	chr6:93820887	0.00253266	1	EPHA7	ENSG00000135333.13	Adipose - Subcutaneous	C=0.968	A=0.032	-0.679264	8.33E-10

rs76462658	rs11962709	chr6:93891195	0.00985203	1	EPHA7	ENSG00000135333.13	Esophagus - Muscularis	A=0.887	G=0.113	0.219885	7.45E-09
rs76462658	rs71540101	chr6:93892043	0.00985203	1	EPHA7	ENSG00000135333.13	Esophagus - Muscularis	AGGTATATC=0.887	-=0.113	0.219885	7.45E-09
rs76462658	rs35962225	chr6:93915766	0.01014541	1	EPHA7	ENSG00000135333.13	Esophagus - Muscularis	G=0.884	C=0.116	0.229888	1.18E-08
rs76462658	rs35762480	chr6:93906245	0.0100474	1	EPHA7	ENSG00000135333.13	Esophagus - Muscularis	G=0.885	C=0.115	0.216734	1.27E-08
rs76462658	rs189136709	chr6:93783798	0.00038505	1	EPHA7	ENSG00000135333.13	Brain - Anterior cingulate cortex (BA24)	G=0.995	A=0.005	1.35876	1.60E-08
rs76462658	rs11962003	chr6:93890071	0.01174419	1	EPHA7	ENSG00000135333.13	Esophagus - Muscularis	A=0.868	G=0.132	0.206691	1.76E-08
rs76462658	rs11966984	chr6:93890176	0.01174419	1	EPHA7	ENSG00000135333.13	Esophagus - Muscularis	G=0.868	A=0.132	0.206691	1.76E-08
rs76462658	rs34948845	chr6:93894820	0.01174419	1	EPHA7	ENSG00000135333.13	Esophagus - Muscularis	G=0.868	A=0.132	0.206691	1.76E-08
rs76462658	rs11966926	chr6:93897338	0.00985203	1	EPHA7	ENSG00000135333.13	Esophagus - Muscularis	A=0.887	G=0.113	0.215362	2.61E-08
rs76462658	rs72919018	chr6:93924471	0.0136905	0.80594136	EPHA7	ENSG00000135333.13	Adipose - Subcutaneous	G=0.785	C=0.215	0.32156	2.76E-08
rs76462658	rs12189899	chr6:93931092	0.0136905	0.80594136	EPHA7	ENSG00000135333.13	Adipose - Subcutaneous	C=0.785	T=0.215	0.32156	2.76E-08
rs76462658	rs12199960	chr6:93923853	0.01357942	0.80503876	EPHA7	ENSG00000135333.13	Adipose - Subcutaneous	A=0.786	C=0.214	0.32248	4.27E-08
rs76462658	rs11967483	chr6:93903691	0.0100474	1	EPHA7	ENSG00000135333.13	Esophagus - Muscularis	A=0.885	T=0.115	0.206895	4.64E-08
rs76462658	rs34832688	chr6:93891054	0.00985203	1	EPHA7	ENSG00000135333.13	Esophagus - Muscularis	A=0.887	C=0.113	0.205584	4.66E-08
rs76462658	rs72919020	chr6:93925349	0.0136905	0.80594136	EPHA7	ENSG00000135333.13	Adipose - Subcutaneous	G=0.785	C=0.215	0.32041	4.76E-08
rs76462658	rs12190544	chr6:93925959	0.0136905	0.80594136	EPHA7	ENSG00000135333.13	Adipose - Subcutaneous	G=0.785	A=0.215	0.32041	4.76E-08
rs76462658	rs12206216	chr6:93927822	0.01380189	0.80683564	EPHA7	ENSG00000135333.13	Adipose - Subcutaneous	A=0.784	G=0.216	0.32041	4.76E-08
rs76462658	rs12196073	chr6:93928926	0.0136905	0.80594136	EPHA7	ENSG00000135333.13	Adipose - Subcutaneous	C=0.785	G=0.215	0.32041	4.76E-08
rs76462658	rs12196420	chr6:93929507	0.0136905	0.80594136	EPHA7	ENSG00000135333.13	Adipose - Subcutaneous	C=0.785	T=0.215	0.32041	4.76E-08
rs76462658	rs12201744	chr6:93930642	0.01357942	0.80503876	EPHA7	ENSG00000135333.13	Adipose - Subcutaneous	A=0.786	T=0.214	0.32041	4.76E-08
rs76462658	rs12195545	chr6:93930755	0.01357942	0.80503876	EPHA7	ENSG00000135333.13	Adipose - Subcutaneous	T=0.786	G=0.214	0.32041	4.76E-08
rs76462658	rs143708097	chr6:93931051	0.01357942	0.80503876	EPHA7	ENSG00000135333.13	Adipose - Subcutaneous	-=0.786	CCTA=0.214	0.32041	4.76E-08
rs76462658	rs72919042	chr6:93935578	0.0136905	0.80594136	EPHA7	ENSG00000135333.13	Adipose - Subcutaneous	C=0.785	T=0.215	0.32041	4.76E-08
rs76462658	rs13212701	chr6:93902787	0.0100474	1	EPHA7	ENSG00000135333.13	Esophagus - Muscularis	T=0.885	C=0.115	0.206435	4.99E-08
rs76462658	rs6936693	chr6:93903686	0.0100474	1	EPHA7	ENSG00000135333.13	Esophagus - Muscularis	T=0.885	C=0.115	0.202265	5.80E-08
rs76462658	rs12374628	chr6:93934768	0.0136905	0.80594136	EPHA7	ENSG00000135333.13	Adipose - Subcutaneous	T=0.785	C=0.215	0.313163	6.59E-08
rs76462658	rs388461	chr6:93918959	0.01014541	1	EPHA7	ENSG00000135333.13	Esophagus - Muscularis	A=0.884	G=0.116	0.191263	7.48E-08
rs76462658	rs575590	chr6:93920650	0.0100474	1	EPHA7	ENSG00000135333.13	Esophagus - Muscularis	G=0.885	A=0.115	0.191263	7.48E-08
rs76462658	rs72919047	chr6:93941158	0.0136905	0.80594136	EPHA7	ENSG00000135333.13	Adipose - Subcutaneous	T=0.785	C=0.215	0.313468	9.08E-08
rs76462658	rs12209980	chr6:93943432	0.0136905	0.80594136	EPHA7	ENSG00000135333.13	Adipose - Subcutaneous	T=0.785	G=0.215	0.313468	9.08E-08
rs76462658	rs12200456	chr6:93924735	0.01380189	0.80683564	EPHA7	ENSG00000135333.13	Adipose - Subcutaneous	A=0.784	G=0.216	0.313602	9.70E-08
rs76462658	rs72919045	chr6:93940859	0.0136905	0.80594136	EPHA7	ENSG00000135333.13	Adipose - Subcutaneous	T=0.785	A=0.215	0.315063	9.80E-08
rs76462658	rs6931234	chr6:93927181	0.01380189	0.80683564	EPHA7	ENSG00000135333.13	Adipose - Subcutaneous	T=0.784	C=0.216	0.299008	1.06E-07
rs76462658	rs13202762	chr6:93937080	0.00785232	1	EPHA7	ENSG00000135333.13	Esophagus - Muscularis	T=0.908	G=0.092	0.222914	1.15E-07
rs76462658	rs10693258	chr6:93902052	0.01024365	1	EPHA7	ENSG00000135333.13	Esophagus - Muscularis	-=0.883	TTG=0.117	0.174304	5.19E-07
rs76462658	rs13212701	chr6:93902787	0.0100474	1	EPHA7	ENSG00000135333.13	Esophagus - Mucosa	T=0.885	C=0.115	0.405746	1.63E-06
rs76462658	rs35762480	chr6:93906245	0.0100474	1	EPHA7	ENSG00000135333.13	Esophagus - Mucosa	G=0.885	C=0.115	0.407424	1.79E-06
rs76462658	rs117376030	chr6:94203683	0.00204519	1	EPHA7	ENSG00000135333.13	Esophagus - Gastroesophageal Junction	T=0.974	A=0.026	-0.384156	2.51E-06
rs76462658	rs72928597	chr6:93839843	0.00675039	1	EPHA7	ENSG00000135333.13	Lung	A=0.919	G=0.081	0.42955	4.55E-06
rs76462658	rs16870853	chr6:93841320	0.00675039	1	EPHA7	ENSG00000135333.13	Lung	T=0.919	C=0.081	0.42955	4.55E-06
rs76462658	rs1930933	chr6:93842544	0.02992801	0.95358066	EPHA7	ENSG00000135333.13	Minor Salivary Gland	C=0.299	T=0.701	0.246092	4.56E-06
rs76462658	rs142723671	chr6:94076414	0.00172351	1	EPHA7	ENSG00000135333.13	Heart - Left Ventricle	G=0.978	C=0.022	-0.495017	4.73E-06
rs76462658	rs11962709	chr6:93891195	0.00985203	1	EPHA7	ENSG00000135333.13	Esophagus - Mucosa	A=0.887	G=0.113	0.385284	6.17E-06
rs76462658	rs71540101	chr6:93892043	0.00985203	1	EPHA7	ENSG00000135333.13	Esophagus - Mucosa	AGGTATATC=0.887	-=0.113	0.385284	6.17E-06
rs76462658	rs147330775	chr6:93915683	0.00046253	1	EPHA7	ENSG00000135333.13	Brain - Anterior cingulate cortex (BA24)	C=0.994	T=0.006	0.808887	6.34E-06
rs76462658	rs6928892	chr6:93888220	0.09285406	0.90654032	EPHA7	ENSG00000135333.13	Esophagus - Muscularis	A=0.594	G=0.406	0.107385	6.55E-06
rs76462658	rs16870793	chr6:93828599	0.00656951	1	EPHA7	ENSG00000135333.13	Lung	G=0.921	A=0.079	0.419095	6.62E-06
rs76462658	rs72928592	chr6:93837959	0.00665985	1	EPHA7	ENSG00000135333.13	Lung	T=0.92	C=0.08	0.419095	6.62E-06
rs76462658	rs10693258	chr6:93902052	0.01024365	1	EPHA7	ENSG00000135333.13	Esophagus - Mucosa	-=0.883	TTG=0.117	0.343667	6.63E-06
rs76462658	rs34832688	chr6:93891054	0.00985203	1	EPHA7	ENSG00000135333.13	Esophagus - Mucosa	A=0.887	C=0.113	0.378517	6.87E-06
rs76462658	rs1575540	chr6:93836577	0.00471634	1	EPHA7	ENSG00000135333.13	Esophagus - Muscularis	G=0.942	A=0.058	0.272463	7.68E-06

rs76462658	rs544639807	chr6:93883657	0.00061794	1	EPHA7	ENSG00000135333.13	Minor Salivary Gland	G=0.992	A=0.008	-1.34812	8.25E-06
rs76462658	rs36114673	chr6:93847056	0.00506297	1	EPHA7	ENSG00000135333.13	Esophagus - Muscularis	A=0.938	T=0.062	0.268662	8.26E-06
rs76462658	rs7776099	chr6:93913423	0.01053673	0.83165997	EPHA7	ENSG00000135333.13	Testis	C=0.835	A=0.165	0.3716	8.26E-06
rs76462658	rs7758242	chr6:93913435	0.01053673	0.83165997	EPHA7	ENSG00000135333.13	Testis	T=0.835	A=0.165	0.3716	8.26E-06
rs76462658	rs66765303	chr6:93913692	0.01053673	0.83165997	EPHA7	ENSG00000135333.13	Testis	A=0.835	G=0.165	0.3716	8.26E-06
rs76462658	rs66522431	chr6:93913825	0.01053673	0.83165997	EPHA7	ENSG00000135333.13	Testis	G=0.835	A=0.165	0.3716	8.26E-06
rs76462658	rs62414181	chr6:93913909	0.01053673	0.83165997	EPHA7	ENSG00000135333.13	Testis	A=0.835	G=0.165	0.3716	8.26E-06
rs76462658	rs62414182	chr6:93914022	0.01053673	0.83165997	EPHA7	ENSG00000135333.13	Testis	A=0.835	G=0.165	0.3716	8.26E-06
rs76462658	rs113888577	chr6:93698093	0.00093064	1	EPHA7	ENSG00000135333.13	Brain - Anterior cingulate cortex (BA24)	C=0.988	T=0.012	0.66365	8.36E-06
rs76462658	rs16870780	chr6:93826505	0.00665985	1	EPHA7	ENSG00000135333.13	Lung	T=0.92	A=0.08	0.385133	9.62E-06
rs76462658	rs1408285	chr6:93893666	0.0924402	0.90638377	EPHA7	ENSG00000135333.13	Esophagus - Muscularis	A=0.593	C=0.407	0.105615	9.88E-06
rs76462658	rs7761955	chr6:93896624	0.0924402	0.90638377	EPHA7	ENSG00000135333.13	Esophagus - Muscularis	T=0.593	C=0.407	0.105615	9.88E-06
rs76462658	rs7772899	chr6:93898633	0.0924402	0.90638377	EPHA7	ENSG00000135333.13	Esophagus - Muscularis	T=0.593	C=0.407	0.105615	9.88E-06
rs76462658	rs1590384	chr6:93837434	0.02992801	0.95358066	EPHA7	ENSG00000135333.13	Minor Salivary Gland	C=0.701	G=0.299	-0.24131	1.00E-05
rs76462658	rs1924474	chr6:93837991	0.02992801	0.95358066	EPHA7	ENSG00000135333.13	Minor Salivary Gland	C=0.701	T=0.299	-0.24131	1.00E-05
rs76462658	rs10944652	chr6:93838348	0.02992801	0.95358066	EPHA7	ENSG00000135333.13	Minor Salivary Gland	C=0.701	G=0.299	-0.24131	1.00E-05
rs76462658	rs12204186	chr6:93838665	0.02992801	0.95358066	EPHA7	ENSG00000135333.13	Minor Salivary Gland	C=0.701	T=0.299	-0.24131	1.00E-05
rs76462658	rs633279	chr6:93840705	0.02992801	0.95358066	EPHA7	ENSG00000135333.13	Minor Salivary Gland	A=0.299	T=0.701	0.24131	1.00E-05
rs76462658	rs634060	chr6:93840849	0.02992801	0.95358066	EPHA7	ENSG00000135333.13	Minor Salivary Gland	A=0.299	G=0.701	0.24131	1.00E-05
rs76462658	rs568957	chr6:93840855	0.02992801	0.95358066	EPHA7	ENSG00000135333.13	Minor Salivary Gland	G=0.299	T=0.701	0.24131	1.00E-05
rs76462658	rs34544163	chr6:93841565	0.02992801	0.95358066	EPHA7	ENSG00000135333.13	Minor Salivary Gland	-=0.299	A=0.701	0.24131	1.00E-05
rs76462658	rs562379	chr6:93841592	0.02992801	0.95358066	EPHA7	ENSG00000135333.13	Minor Salivary Gland	G=0.299	A=0.701	0.24131	1.00E-05
rs76462658	rs560731	chr6:93841734	0.02992801	0.95358066	EPHA7	ENSG00000135333.13	Minor Salivary Gland	A=0.299	G=0.701	0.24131	1.00E-05
rs76462658	rs650642	chr6:93842232	0.02992801	0.95358066	EPHA7	ENSG00000135333.13	Minor Salivary Gland	G=0.299	T=0.701	0.24131	1.00E-05
rs76462658	rs650747	chr6:93842298	0.02992801	0.95358066	EPHA7	ENSG00000135333.13	Minor Salivary Gland	T=0.299	C=0.701	0.24131	1.00E-05
rs76462658	rs1930934	chr6:93842533	0.02992801	0.95358066	EPHA7	ENSG00000135333.13	Minor Salivary Gland	A=0.299	G=0.701	0.24131	1.00E-05
rs76462658	rs9353987	chr6:93843588	0.02992801	0.95358066	EPHA7	ENSG00000135333.13	Minor Salivary Gland	A=0.299	G=0.701	0.24131	1.00E-05
rs76462658	rs9363034	chr6:93844136	0.02992801	0.95358066	EPHA7	ENSG00000135333.13	Minor Salivary Gland	G=0.299	A=0.701	0.24131	1.00E-05
rs76462658	rs9353989	chr6:93844383	0.02992801	0.95358066	EPHA7	ENSG00000135333.13	Minor Salivary Gland	G=0.299	T=0.701	0.24131	1.00E-05
rs76462658	rs74822095	chr6:93741512	0.00386244	1	EPHA7	ENSG00000135333.13	Adipose - Subcutaneous	C=0.952	T=0.048	-0.476757	1.06E-05
rs76462658	rs76282783	chr6:93576050	0.0033586	1	EPHA7	ENSG00000135333.13	Lung	C=0.958	A=0.042	0.702179	1.18E-05
rs76462658	rs10693258	chr6:93902052	0.01024365	1	EPHA7	ENSG00000135333.13	Esophagus - Gastroesophageal Junction	-=0.883	TTG=0.117	0.189047	1.21E-05
rs76462658	rs6936693	chr6:93903686	0.0100474	1	EPHA7	ENSG00000135333.13	Esophagus - Mucosa	T=0.885	C=0.115	0.361801	1.30E-05
rs76462658	rs11967483	chr6:93903691	0.0100474	1	EPHA7	ENSG00000135333.13	Esophagus - Mucosa	A=0.885	T=0.115	0.36623	1.31E-05
rs76462658	rs72926533	chr6:93740212	0.00069588	1	EPHA7	ENSG00000135333.13	Minor Salivary Gland	C=0.991	T=0.009	-1.15096	1.32E-05
rs76462658	rs188317446	chr6:93743394	0.00069588	1	EPHA7	ENSG00000135333.13	Minor Salivary Gland	G=0.991	C=0.009	-1.15096	1.32E-05
rs76462658	rs72926535	chr6:93747452	0.00069588	1	EPHA7	ENSG00000135333.13	Minor Salivary Gland	C=0.991	T=0.009	-1.15096	1.32E-05
rs76462658	rs117415227	chr6:93735745	0.00386244	1	EPHA7	ENSG00000135333.13	Adipose - Subcutaneous	T=0.952	G=0.048	-0.47253	1.37E-05
rs76462658	rs11966926	chr6:93897338	0.00985203	1	EPHA7	ENSG00000135333.13	Esophagus - Mucosa	A=0.887	G=0.113	0.374015	1.38E-05
rs76462658	rs199888991	chr6:93784078	0.00038505	1	EPHA7	ENSG00000135333.13	Adipose - Subcutaneous	-=0.995	T=0.005	-0.796053	1.44E-05
rs76462658	rs111429740	chr6:93838453	0.00675039	1	EPHA7	ENSG00000135333.13	Lung	G=0.919	A=0.081	0.383525	1.55E-05
rs76462658	rs72919045	chr6:93940859	0.0136905	0.80594136	EPHA7	ENSG00000135333.13	Breast - Mammary Tissue	T=0.785	A=0.215	0.313719	1.71E-05
rs76462658	rs16870789	chr6:93827963	0.00665985	1	EPHA7	ENSG00000135333.13	Lung	C=0.92	T=0.08	0.374368	1.81E-05
rs76462658	rs145810085	chr6:93833819	0.00061794	1	EPHA7	ENSG00000135333.13	Adipose - Subcutaneous	G=0.992	A=0.008	-0.735109	1.88E-05
rs76462658	rs7774823	chr6:93830545	0.02962575	0.95327016	EPHA7	ENSG00000135333.13	Minor Salivary Gland	G=0.703	A=0.297	-0.232136	1.89E-05
rs76462658	rs1951907	chr6:93831192	0.02962575	0.95327016	EPHA7	ENSG00000135333.13	Minor Salivary Gland	T=0.703	C=0.297	-0.232136	1.89E-05
rs76462658	rs35306488	chr6:93832227	0.02917557	0.95279655	EPHA7	ENSG00000135333.13	Minor Salivary Gland	-=0.706	T=0.294	-0.232136	1.89E-05
rs76462658	rs10944648	chr6:93832280	0.02962575	0.95327016	EPHA7	ENSG00000135333.13	Minor Salivary Gland	A=0.703	G=0.297	-0.232136	1.89E-05
rs76462658	rs7751375	chr6:93832923	0.02977667	0.95342593	EPHA7	ENSG00000135333.13	Minor Salivary Gland	C=0.702	A=0.298	-0.232136	1.89E-05
rs76462658	rs7757292	chr6:93834165	0.02962575	0.95327016	EPHA7	ENSG00000135333.13	Minor Salivary Gland	C=0.703	A=0.297	-0.232136	1.89E-05
rs76462658	rs11962709	chr6:93891195	0.00985203	1	EPHA7	ENSG00000135333.13	Esophagus - Gastroesophageal Junction	A=0.887	G=0.113	0.198094	1.91E-05

rs76462658	rs71540101	chr6:93892043	0.00985203	1	EPHA7	ENSG00000135333.13	Esophagus - Gastroesophageal Junction	AGGTATATC=0.887	-=0.113	0.198094	1.91E-05
rs76462658	rs35762480	chr6:93906245	0.0100474	1	EPHA7	ENSG00000135333.13	Esophagus - Gastroesophageal Junction	G=0.885	C=0.115	0.19742	1.95E-05
rs76462658	rs16870850	chr6:93841079	0.00711451	1	EPHA7	ENSG00000135333.13	Lung	T=0.916	C=0.084	0.361979	2.02E-05
rs76462658	rs12528846	chr6:93828842	0.00665985	1	EPHA7	ENSG00000135333.13	Lung	A=0.92	G=0.08	0.377147	2.03E-05
rs76462658	rs12527296	chr6:93829143	0.00665985	1	EPHA7	ENSG00000135333.13	Lung	T=0.92	C=0.08	0.377147	2.03E-05
rs76462658	rs72928575	chr6:93829399	0.00675039	1	EPHA7	ENSG00000135333.13	Lung	G=0.919	T=0.081	0.377147	2.03E-05
rs76462658	rs72928579	chr6:93829658	0.00665985	1	EPHA7	ENSG00000135333.13	Lung	G=0.92	A=0.08	0.377147	2.03E-05
rs76462658	rs77007574	chr6:93829960	0.00665985	1	EPHA7	ENSG00000135333.13	Lung	T=0.92	C=0.08	0.377147	2.03E-05
rs76462658	rs72928582	chr6:93830015	0.00665985	1	EPHA7	ENSG00000135333.13	Lung	G=0.92	A=0.08	0.377147	2.03E-05
rs76462658	rs16870801	chr6:93830951	0.00665985	1	EPHA7	ENSG00000135333.13	Lung	T=0.92	C=0.08	0.377147	2.03E-05
rs76462658	rs16870805	chr6:93834075	0.00665985	1	EPHA7	ENSG00000135333.13	Lung	G=0.92	T=0.08	0.377147	2.03E-05
rs76462658	rs150304721	chr6:93835801	0.00684112	1	EPHA7	ENSG00000135333.13	Lung	A=0.918	-=0.082	0.361316	2.04E-05
rs76462658	rs11967483	chr6:93903691	0.0100474	1	EPHA7	ENSG00000135333.13	Esophagus - Gastroesophageal Junction	A=0.885	T=0.115	0.196042	2.11E-05
rs76462658	rs16870855	chr6:93841440	0.00675039	1	EPHA7	ENSG00000135333.13	Lung	C=0.919	T=0.081	0.4288	2.14E-05
rs76462658	rs13212701	chr6:93902787	0.0100474	1	EPHA7	ENSG00000135333.13	Esophagus - Gastroesophageal Junction	T=0.885	C=0.115	0.195748	2.16E-05
rs76462658	rs16880183	chr6:93889701	0.01512424	1	EPHA7	ENSG00000135333.13	Testis	A=0.836	G=0.164	0.353058	2.24E-05
rs76462658	rs12199960	chr6:93923853	0.01357942	0.80503876	EPHA7	ENSG00000135333.13	Breast - Mammary Tissue	A=0.786	C=0.214	0.305305	2.45E-05
rs76462658	rs12200456	chr6:93924735	0.01380189	0.80683564	EPHA7	ENSG00000135333.13	Breast - Mammary Tissue	A=0.784	G=0.216	0.305305	2.45E-05
rs76462658	rs72919020	chr6:93925349	0.0136905	0.80594136	EPHA7	ENSG00000135333.13	Breast - Mammary Tissue	G=0.785	C=0.215	0.305305	2.45E-05
rs76462658	rs12190544	chr6:93925959	0.0136905	0.80594136	EPHA7	ENSG00000135333.13	Breast - Mammary Tissue	G=0.785	A=0.215	0.305305	2.45E-05
rs76462658	rs12206216	chr6:93927822	0.01380189	0.80683564	EPHA7	ENSG00000135333.13	Breast - Mammary Tissue	A=0.784	G=0.216	0.305305	2.45E-05
rs76462658	rs12196073	chr6:93928926	0.0136905	0.80594136	EPHA7	ENSG00000135333.13	Breast - Mammary Tissue	C=0.785	G=0.215	0.305305	2.45E-05
rs76462658	rs12196420	chr6:93929507	0.0136905	0.80594136	EPHA7	ENSG00000135333.13	Breast - Mammary Tissue	C=0.785	T=0.215	0.305305	2.45E-05
rs76462658	rs12201744	chr6:93930642	0.01357942	0.80503876	EPHA7	ENSG00000135333.13	Breast - Mammary Tissue	A=0.786	T=0.214	0.305305	2.45E-05
rs76462658	rs12195545	chr6:93930755	0.01357942	0.80503876	EPHA7	ENSG00000135333.13	Breast - Mammary Tissue	T=0.786	G=0.214	0.305305	2.45E-05
rs76462658	rs143708097	chr6:93931051	0.01357942	0.80503876	EPHA7	ENSG00000135333.13	Breast - Mammary Tissue	-=0.786	CCTA=0.214	0.305305	2.45E-05
rs76462658	rs72919042	chr6:93935578	0.0136905	0.80594136	EPHA7	ENSG00000135333.13	Breast - Mammary Tissue	C=0.785	T=0.215	0.305305	2.45E-05
rs76462658	rs7772178	chr6:93849608	0.01024365	1	EPHA7	ENSG00000135333.13	Testis	C=0.883	T=0.117	0.425527	2.47E-05
rs76462658	rs72919047	chr6:93941158	0.0136905	0.80594136	EPHA7	ENSG00000135333.13	Breast - Mammary Tissue	T=0.785	C=0.215	0.304408	2.54E-05
rs76462658	rs12209980	chr6:93943432	0.0136905	0.80594136	EPHA7	ENSG00000135333.13	Breast - Mammary Tissue	T=0.785	G=0.215	0.304408	2.54E-05
rs76462658	rs6923008	chr6:93887045	0.01512424	1	EPHA7	ENSG00000135333.13	Testis	G=0.836	T=0.164	0.351036	2.54E-05
rs76462658	rs60592958	chr6:93887933	0.01512424	1	EPHA7	ENSG00000135333.13	Testis	C=0.836	T=0.164	0.351036	2.54E-05
rs76462658	rs147445521	chr6:93825169	0.00558845	1	EPHA7	ENSG00000135333.13	Lung	TGT=0.932	-=0.068	0.417524	2.57E-05
rs76462658	rs118177782	chr6:93767900	0.00204519	1	EPHA7	ENSG00000135333.13	Brain - Anterior cingulate cortex (BA24)	C=0.974	T=0.026	0.590137	2.71E-05
rs76462658	rs73758259	chr6:93828724	0.00693206	1	EPHA7	ENSG00000135333.13	Lung	G=0.917	T=0.083	0.354112	2.72E-05
rs76462658	rs16870796	chr6:93828781	0.00665985	1	EPHA7	ENSG00000135333.13	Lung	T=0.92	G=0.08	0.369069	2.76E-05
rs76462658	rs72928577	chr6:93829400	0.00675039	1	EPHA7	ENSG00000135333.13	Lung	C=0.919	T=0.081	0.369069	2.76E-05
rs76462658	rs11966926	chr6:93897338	0.00985203	1	EPHA7	ENSG00000135333.13	Esophagus - Gastroesophageal Junction	A=0.887	G=0.113	0.196345	2.79E-05
rs76462658	rs117717826	chr6:94036945	0.00196452	1	EPHA7	ENSG00000135333.13	Heart - Left Ventricle	T=0.975	A=0.025	-0.389622	2.96E-05
rs76462658	rs56378615	chr6:93900091	0.01090501	0.90559309	EPHA7	ENSG00000135333.13	Testis	C=0.853	T=0.147	0.358985	3.02E-05
rs76462658	rs6936693	chr6:93903686	0.0100474	1	EPHA7	ENSG00000135333.13	Esophagus - Gastroesophageal Junction	T=0.885	C=0.115	0.189133	3.05E-05
rs76462658	rs71558449	chr6:93828589	0.00261449	1	EPHA7	ENSG00000135333.13	Esophagus - Muscularis	G=0.967	A=0.033	0.272458	3.14E-05
rs76462658	rs6922405	chr6:93842847	0.00675039	1	EPHA7	ENSG00000135333.13	Lung	T=0.919	C=0.081	0.370721	3.25E-05
rs76462658	rs72930416	chr6:93844150	0.00675039	1	EPHA7	ENSG00000135333.13	Lung	T=0.919	C=0.081	0.370721	3.25E-05
rs76462658	rs34948845	chr6:93894820	0.01174419	1	EPHA7	ENSG00000135333.13	Esophagus - Gastroesophageal Junction	G=0.868	A=0.132	0.186343	3.27E-05
rs76462658	rs16870846	chr6:93840909	0.00675039	1	EPHA7	ENSG00000135333.13	Lung	C=0.919	G=0.081	0.371767	3.40E-05
rs76462658	rs1319460	chr6:93846637	0.02415283	1	EPHA7	ENSG00000135333.13	Minor Salivary Gland	C=0.239	T=0.761	0.230876	3.54E-05
rs76462658	rs538751	chr6:93848400	0.02415283	1	EPHA7	ENSG00000135333.13	Minor Salivary Gland	A=0.239	C=0.761	0.230876	3.54E-05
rs76462658	rs11422146	chr6:93849652	0.02402084	1	EPHA7	ENSG00000135333.13	Minor Salivary Gland	-=0.238	T=0.762	0.230876	3.54E-05
rs76462658	rs34832688	chr6:93891054	0.00985203	1	EPHA7	ENSG00000135333.13	Esophagus - Gastroesophageal Junction	A=0.887	C=0.113	0.187735	3.54E-05
rs76462658	rs1319460	chr6:93846637	0.02415283	1	EPHA7	ENSG00000135333.13	Testis	C=0.239	T=0.761	-0.330556	3.66E-05

rs76462658	rs538751	chr6:93848400	0.02415283	1	EPHA7	ENSG00000135333.13	Testis	A=0.239	C=0.761	-0.330556	3.66E-05
rs76462658	rs11422146	chr6:93849652	0.02402084	1	EPHA7	ENSG00000135333.13	Testis	-=0.238	T=0.762	-0.330556	3.66E-05
rs76462658	rs6931234	chr6:93927181	0.01380189	0.80683564	EPHA7	ENSG00000135333.13	Breast - Mammary Tissue	T=0.784	C=0.216	0.288667	3.69E-05
rs76462658	rs117415227	chr6:93735745	0.00386244	1	EPHA7	ENSG00000135333.13	Lung	T=0.952	G=0.048	-0.50856	4.07E-05
rs76462658	rs72919018	chr6:93924471	0.0136905	0.80594136	EPHA7	ENSG00000135333.13	Breast - Mammary Tissue	G=0.785	C=0.215	0.291651	4.19E-05
rs76462658	rs12189899	chr6:93931092	0.0136905	0.80594136	EPHA7	ENSG00000135333.13	Breast - Mammary Tissue	C=0.785	T=0.215	0.291651	4.19E-05
rs76462658	rs6935219	chr6:93889277	0.01512424	1	EPHA7	ENSG00000135333.13	Testis	A=0.836	G=0.164	0.33974	4.29E-05
rs76462658	rs12374628	chr6:93934768	0.0136905	0.80594136	EPHA7	ENSG00000135333.13	Breast - Mammary Tissue	T=0.785	C=0.215	0.290954	4.31E-05
rs76462658	rs11962003	chr6:93890071	0.01174419	1	EPHA7	ENSG00000135333.13	Esophagus - Mucosa	A=0.868	G=0.132	0.333682	4.39E-05
rs76462658	rs11966984	chr6:93890176	0.01174419	1	EPHA7	ENSG00000135333.13	Esophagus - Mucosa	G=0.868	A=0.132	0.333682	4.39E-05
rs76462658	rs140182812	chr6:93804212	0.00061794	1	EPHA7	ENSG00000135333.13	Adipose - Subcutaneous	G=0.992	A=0.008	-0.729088	4.39E-05
rs76462658	rs11962003	chr6:93890071	0.01174419	1	EPHA7	ENSG00000135333.13	Esophagus - Gastroesophageal Junction	A=0.868	G=0.132	0.183007	4.40E-05
rs76462658	rs11966984	chr6:93890176	0.01174419	1	EPHA7	ENSG00000135333.13	Esophagus - Gastroesophageal Junction	G=0.868	A=0.132	0.183007	4.40E-05
rs76462658	rs34948845	chr6:93894820	0.01174419	1	EPHA7	ENSG00000135333.13	Esophagus - Mucosa	G=0.868	A=0.132	0.334329	4.44E-05
rs76462658	rs10686381	chr6:93887552	0.01512424	1	EPHA7	ENSG00000135333.13	Testis	-=0.836	AAGT=0.164	0.337644	4.84E-05
rs76462658	rs2224853	chr6:93890865	0.04687736	0.93250134	EPHA7	ENSG00000135333.13	Esophagus - Muscularis	T=0.588	C=0.412	-0.0928827	5.11E-05
rs76462658	rs41273625	chr6:93952851	0.00515009	1	EPHA7	ENSG00000135333.13	Esophagus - Muscularis	C=0.937	G=0.063	0.224035	5.17E-05
rs76462658	rs78355320	chr6:93821638	0.00386244	1	EPHA7	ENSG00000135333.13	Adipose - Subcutaneous	T=0.952	A=0.048	-0.431835	5.33E-05
rs76462658	rs12530331	chr6:93830460	0.00675039	1	EPHA7	ENSG00000135333.13	Lung	G=0.919	C=0.081	0.35653	5.33E-05
rs76462658	rs72920784	chr6:93673551	0.00156365	1	EPHA7	ENSG00000135333.13	Adipose - Subcutaneous	A=0.98	G=0.02	-0.579253	5.40E-05
rs76462658	rs72920785	chr6:93675496	0.00156365	1	EPHA7	ENSG00000135333.13	Adipose - Subcutaneous	G=0.98	C=0.02	-0.579253	5.40E-05
rs76462658	rs74468395	chr6:94017659	0.00261449	1	EPHA7	ENSG00000135333.13	Heart - Left Ventricle	C=0.967	T=0.033	-0.353	6.19E-05
rs76462658	rs117256127	chr6:93852714	0.00188402	1	EPHA7	ENSG00000135333.13	Adipose - Subcutaneous	T=0.976	C=0.024	-0.674139	6.42E-05
rs76462658	rs117425371	chr6:93852717	0.00188402	1	EPHA7	ENSG00000135333.13	Adipose - Subcutaneous	A=0.976	G=0.024	-0.674139	6.42E-05
rs76462658	rs145519743	chr6:93884032	0.00196452	1	EPHA7	ENSG00000135333.13	Adipose - Subcutaneous	G=0.975	A=0.025	-0.674139	6.42E-05
rs76462658	rs9351349	chr6:93832104	0.01429205	0.92237654	EPHA7	ENSG00000135333.13	Adipose - Subcutaneous	G=0.179	A=0.821	0.228214	6.48E-05
rs76462658	rs147330775	chr6:93915683	0.00046253	1	EPHA7	ENSG00000135333.13	Skin - Sun Exposed (Lower leg)	C=0.994	T=0.006	-0.47159	6.56E-05
rs76462658	rs117109139	chr6:93861398	0.00302617	1	EPHA7	ENSG00000135333.13	Esophagus - Muscularis	C=0.962	G=0.038	0.257424	7.15E-05
rs76462658	rs650711	chr6:93842280	0.01429205	0.92237654	EPHA7	ENSG00000135333.13	Adipose - Subcutaneous	T=0.821	G=0.179	-0.226702	7.44E-05
rs76462658	rs768382	chr6:93826168	0.01418212	0.92194289	EPHA7	ENSG00000135333.13	Adipose - Subcutaneous	T=0.178	C=0.822	0.223191	7.87E-05
rs76462658	rs1324110	chr6:93913200	0.08054639	0.90125638	EPHA7	ENSG00000135333.13	Heart - Atrial Appendage	G=0.563	C=0.437	0.0819979	9.78E-05
rs76462658	rs41273625	chr6:93952851	0.00515009	1	EPHA7	ENSG00000135333.13	Esophagus - Gastroesophageal Junction	C=0.937	G=0.063	0.249826	0.00010545
rs76462658	rs11970583	chr6:93895756	0.02973083	1	EPHA7	ENSG00000135333.13	Esophagus - Muscularis	C=0.722	T=0.278	0.0990772	0.00011475
rs76462658	rs2065582	chr6:93896309	0.02973083	1	EPHA7	ENSG00000135333.13	Esophagus - Muscularis	G=0.722	T=0.278	0.0990772	0.00011475
rs76462658	rs2147225	chr6:93898127	0.02973083	1	EPHA7	ENSG00000135333.13	Esophagus - Muscularis	T=0.722	A=0.278	0.0990772	0.00011475
rs76462658	rs13205265	chr6:93898896	0.02973083	1	EPHA7	ENSG00000135333.13	Esophagus - Muscularis	C=0.722	T=0.278	0.0990772	0.00011475
rs76462658	rs35480101	chr6:93899371	0.0295839	1	EPHA7	ENSG00000135333.13	Esophagus - Muscularis	G=0.723	T=0.277	0.0990772	0.00011475
rs76462658	rs145046790	chr6:93899520	0.02973083	1	EPHA7	ENSG00000135333.13	Esophagus - Muscularis	AT=0.722	-=0.278	0.0990772	0.00011475
rs76462658	rs192604147	chr6:94240629	0.00069588	1	EPHA7	ENSG00000135333.13	Heart - Left Ventricle	A=0.991	G=0.009	-0.514265	0.00011598
rs76462658	rs1324103	chr6:93901016	0.04436088	0.89972089	EPHA7	ENSG00000135333.13	Esophagus - Muscularis	A=0.584	G=0.416	-0.0876121	0.00012747
rs76462658	rs1324104	chr6:93901538	0.04436088	0.89972089	EPHA7	ENSG00000135333.13	Esophagus - Muscularis	T=0.584	G=0.416	-0.0876121	0.00012747
rs76462658	rs16880179	chr6:93889506	0.013087	1	EPHA7	ENSG00000135333.13	Testis	G=0.855	A=0.145	0.335079	0.00013631
rs76462658	rs59810007	chr6:93891733	0.013087	1	EPHA7	ENSG00000135333.13	Testis	T=0.855	C=0.145	0.335079	0.00013631
rs76462658	rs6901961	chr6:93892700	0.013087	1	EPHA7	ENSG00000135333.13	Testis	T=0.855	A=0.145	0.335079	0.00013631
rs76462658	rs60804851	chr6:93894513	0.013087	1	EPHA7	ENSG00000135333.13	Testis	C=0.855	G=0.145	0.335079	0.00013631
rs76462658	rs6901870	chr6:93895903	0.013087	1	EPHA7	ENSG00000135333.13	Testis	A=0.855	C=0.145	0.335079	0.00013631
rs76462658	rs74822095	chr6:93741512	0.00386244	1	EPHA7	ENSG00000135333.13	Lung	C=0.952	T=0.048	-0.471073	0.00014377
rs76462658	rs6901827	chr6:93895828	0.02973083	1	EPHA7	ENSG00000135333.13	Esophagus - Muscularis	A=0.722	G=0.278	0.0976714	0.00014821
rs76462658	rs2181805	chr6:93898026	0.02973083	1	EPHA7	ENSG00000135333.13	Esophagus - Muscularis	T=0.722	C=0.278	0.0976714	0.00014821
rs76462658	rs71298717	chr6:93898609	0.02617495	1	EPHA7	ENSG00000135333.13	Esophagus - Muscularis	-=0.747	ATTTTAGGAATA=0.25:	0.0976714	0.00014821
rs76462658	rs143801031	chr6:93750441	0.00132507	1	EPHA7	ENSG00000135333.13	Adipose - Subcutaneous	T=0.983	C=0.017	-0.536087	0.00015072

rs76462658	rs6922792	chr6:93887222	0.013087	1	EPHA7	ENSG00000135333.13	Testis	A=0.855	G=0.145	0.333048	0.00015245
rs76462658	rs117365486	chr6:93820610	0.00253266	1	EPHA7	ENSG00000135333.13	Nerve - Tibial	C=0.968	T=0.032	-0.404506	0.000162
rs76462658	rs148086058	chr6:93820887	0.00253266	1	EPHA7	ENSG00000135333.13	Nerve - Tibial	C=0.968	A=0.032	-0.404506	0.000162
rs76462658	rs11966965	chr6:93890049	0.0295839	1	EPHA7	ENSG00000135333.13	Esophagus - Muscularis	G=0.723	T=0.277	0.096593	0.00016937
rs76462658	rs35373649	chr6:93891073	0.0295839	1	EPHA7	ENSG00000135333.13	Esophagus - Muscularis	A=0.723	G=0.277	0.096593	0.00016937
rs76462658	rs6921600	chr6:93892299	0.02973083	1	EPHA7	ENSG00000135333.13	Esophagus - Muscularis	G=0.722	T=0.278	0.096593	0.00016937
rs76462658	rs6901416	chr6:93892412	0.02973083	1	EPHA7	ENSG00000135333.13	Esophagus - Muscularis	T=0.722	G=0.278	0.096593	0.00016937
rs76462658	rs6454946	chr6:93902683	0.04477275	0.90019841	EPHA7	ENSG00000135333.13	Esophagus - Muscularis	A=0.583	G=0.417	-0.0835518	0.00021296
rs76462658	rs1324106	chr6:93902867	0.04477275	0.90019841	EPHA7	ENSG00000135333.13	Esophagus - Muscularis	T=0.583	A=0.417	-0.0835518	0.00021296
rs76462658	rs1324107	chr6:93903050	0.04477275	0.90019841	EPHA7	ENSG00000135333.13	Esophagus - Muscularis	C=0.583	G=0.417	-0.0835518	0.00021296
rs76462658	rs658231	chr6:93903544	0.04477275	0.90019841	EPHA7	ENSG00000135333.13	Esophagus - Muscularis	C=0.583	T=0.417	-0.0835518	0.00021296
rs76462658	rs1953145	chr6:93890592	0.0295839	1	EPHA7	ENSG00000135333.13	Esophagus - Muscularis	G=0.723	A=0.277	0.0952131	0.00021698
rs76462658	rs12110542	chr6:93894263	0.02973083	1	EPHA7	ENSG00000135333.13	Esophagus - Muscularis	T=0.722	G=0.278	0.0952131	0.00021698
rs76462658	rs1953146	chr6:93897891	0.02973083	1	EPHA7	ENSG00000135333.13	Esophagus - Muscularis	C=0.722	G=0.278	0.0952131	0.00021698
rs76462658	rs182357880	chr6:93879803	0.00054015	1	EPHA7	ENSG00000135333.13	Skin - Sun Exposed (Lower leg)	T=0.993	C=0.007	-0.395624	0.00022421
rs76462658	rs144384339	chr6:93897863	0.0016435	1	EPHA7	ENSG00000135333.13	Skin - Sun Exposed (Lower leg)	G=0.979	A=0.021	-0.418007	0.00029351

Supplementary Table S5b. *PSD3* eQTL SNPs in LD with SNPs on chromosome 8, associated with COVID-19 survival

Query	RS ID	Position	R2	D'	Gene Symbol	Gencode ID	Tissue	Non-effect Allele Freq	Effect Allele Freq	Effect Size	P-value
rs111241636	rs11778625	chr8:18668681	0.014069014	0.81660742	PSD3	ENSG00000156011.16	Brain - Spinal cord (cervical c-1)	T=0.526	G=0.474	0.51445	3.05E-09
rs111241636	rs11775742	chr8:18665710	0.014848306	0.820741269	PSD3	ENSG00000156011.16	Brain - Spinal cord (cervical c-1)	T=0.515	C=0.485	0.50233	8.42E-09
rs111241636	rs144493102	chr8:18882143	0.000306315	1	PSD3	ENSG00000156011.16	Esophagus - Muscularis	G=0.987	C=0.013	0.888397	1.05E-08
rs111241636	rs11774752	chr8:18664654	0.015586679	0.824340842	PSD3	ENSG00000156011.16	Brain - Spinal cord (cervical c-1)	T=0.505	C=0.495	0.487492	2.02E-08
rs111241636	rs11775676	chr8:18665492	0.014561042	0.819259792	PSD3	ENSG00000156011.16	Brain - Spinal cord (cervical c-1)	T=0.519	C=0.481	0.487492	2.02E-08
rs111241636	rs11780950	chr8:18668483	0.013862201	0.815446707	PSD3	ENSG00000156011.16	Brain - Spinal cord (cervical c-1)	G=0.529	C=0.471	0.48504	2.14E-08
rs111241636	rs2069245	chr8:18657093	0.015066746	0.821836536	PSD3	ENSG00000156011.16	Brain - Spinal cord (cervical c-1)	G=0.512	C=0.488	0.482474	3.30E-08
rs111241636	rs11774165	chr8:18658950	0.015814006	0.825392693	PSD3	ENSG00000156011.16	Brain - Spinal cord (cervical c-1)	T=0.502	G=0.498	0.482474	3.30E-08
rs111241636	rs11786921	chr8:18657985	0.015890395	0.825740516	PSD3	ENSG00000156011.16	Brain - Spinal cord (cervical c-1)	T=0.501	C=0.499	0.46989	4.88E-08
rs111241636	rs11786923	chr8:18658007	0.015814006	0.825392693	PSD3	ENSG00000156011.16	Brain - Spinal cord (cervical c-1)	T=0.502	G=0.498	0.46989	4.88E-08
rs111241636	rs13276530	chr8:18658346	0.016121424	0.826775721	PSD3	ENSG00000156011.16	Brain - Spinal cord (cervical c-1)	A=0.498	G=0.502	0.46989	4.88E-08
rs111241636	rs36085278	chr8:18659470	0.016355287	0.827798699	PSD3	ENSG00000156011.16	Brain - Spinal cord (cervical c-1)	A=0.495	G=0.505	0.46989	4.88E-08
rs111241636	rs11784567	chr8:18835526	0.000378146	1	PSD3	ENSG00000156011.16	Esophagus - Muscularis	C=0.984	T=0.016	0.807437	4.93E-08
rs111241636	rs11993401	chr8:18657427	0.01566215	0.824692864	PSD3	ENSG00000156011.16	Brain - Spinal cord (cervical c-1)	C=0.504	T=0.496	0.468976	8.04E-08
rs111241636	rs11993467	chr8:18657661	0.015586679	0.824340842	PSD3	ENSG00000156011.16	Brain - Spinal cord (cervical c-1)	C=0.505	T=0.495	0.468976	8.04E-08
rs111241636	rs11997879	chr8:18657450	0.015737925	0.825043478	PSD3	ENSG00000156011.16	Brain - Spinal cord (cervical c-1)	T=0.503	A=0.497	0.464045	8.13E-08
rs111241636	rs11986109	chr8:18657632	0.015737925	0.825043478	PSD3	ENSG00000156011.16	Brain - Spinal cord (cervical c-1)	G=0.503	C=0.497	0.464045	8.13E-08
rs111241636	rs34059664	chr8:18657698	0.01566215	0.824692864	PSD3	ENSG00000156011.16	Brain - Spinal cord (cervical c-1)	A=0.504	G=0.496	0.464045	8.13E-08
rs111241636	rs151026391	chr8:18820154	0.000645291	1	PSD3	ENSG00000156011.16	Esophagus - Muscularis	G=0.973	T=0.027	0.610937	1.19E-07
rs111241636	rs7009615	chr8:18572835	0.002668848	1	PSD3	ENSG00000156011.16	Small Intestine - Terminal Ileum	T=0.898	C=0.102	0.37749	1.38E-07
rs111241636	rs138983771	chr8:18919610	0.00033021	1	PSD3	ENSG00000156011.16	Esophagus - Muscularis	G=0.986	A=0.014	0.754799	1.43E-07
rs111241636	rs2069245	chr8:18657093	0.015066746	0.821836536	PSD3	ENSG00000156011.16	Brain - Substantia nigra	G=0.512	C=0.488	0.289388	3.50E-07
rs111241636	rs11780950	chr8:18668483	0.013862201	0.815446707	PSD3	ENSG00000156011.16	Brain - Substantia nigra	G=0.529	C=0.471	0.294511	3.71E-07
rs111241636	rs11775676	chr8:18665492	0.014561042	0.819259792	PSD3	ENSG00000156011.16	Brain - Substantia nigra	T=0.519	C=0.481	0.278113	5.86E-07
rs111241636	rs11778625	chr8:18668681	0.014069014	0.81660742	PSD3	ENSG00000156011.16	Brain - Substantia nigra	T=0.526	G=0.474	0.286342	6.80E-07
rs111241636	rs10503636	chr8:18654794	0.017155955	0.831123049	PSD3	ENSG00000156011.16	Brain - Spinal cord (cervical c-1)	T=0.485	C=0.515	0.423645	8.47E-07
rs111241636	rs11774752	chr8:18664654	0.015586679	0.824340842	PSD3	ENSG00000156011.16	Brain - Substantia nigra	T=0.505	C=0.495	0.272367	9.41E-07
rs111241636	rs11786921	chr8:18657985	0.015890395	0.825740516	PSD3	ENSG00000156011.16	Brain - Substantia nigra	T=0.501	C=0.499	0.268879	9.98E-07
rs111241636	rs11786923	chr8:18658007	0.015814006	0.825392693	PSD3	ENSG00000156011.16	Brain - Substantia nigra	T=0.502	G=0.498	0.268879	9.98E-07
rs111241636	rs13276530	chr8:18658346	0.016121424	0.826775721	PSD3	ENSG00000156011.16	Brain - Substantia nigra	A=0.498	G=0.502	0.268879	9.98E-07
rs111241636	rs11774165	chr8:18658950	0.015814006	0.825392693	PSD3	ENSG00000156011.16	Brain - Substantia nigra	T=0.502	G=0.498	0.268879	9.98E-07
rs111241636	rs36085278	chr8:18659470	0.016355287	0.827798699	PSD3	ENSG00000156011.16	Brain - Substantia nigra	A=0.495	G=0.505	0.268879	9.98E-07
rs111241636	rs11775742	chr8:18665710	0.014848306	0.820741269	PSD3	ENSG00000156011.16	Brain - Substantia nigra	T=0.515	C=0.485	0.27395	1.42E-06
rs111241636	rs11986109	chr8:18657632	0.015737925	0.825043478	PSD3	ENSG00000156011.16	Brain - Substantia nigra	G=0.503	C=0.497	0.267572	1.94E-06
rs111241636	rs60854172	chr8:18867056	0.002813985	1	PSD3	ENSG00000156011.16	Esophagus - Mucosa	-=0.893	A=0.107	0.215939	2.93E-06
rs111241636	rs111916415	chr8:18656221	0.023807682	0.920329453	PSD3	ENSG00000156011.16	Brain - Spinal cord (cervical c-1)	A=0.454	T=0.546	0.411994	2.93E-06
rs111241636	rs11997879	chr8:18657450	0.015737925	0.825043478	PSD3	ENSG00000156011.16	Brain - Substantia nigra	T=0.503	A=0.497	0.261377	3.09E-06
rs111241636	rs34059664	chr8:18657698	0.01566215	0.824692864	PSD3	ENSG00000156011.16	Brain - Substantia nigra	A=0.504	G=0.496	0.261377	3.09E-06
rs111241636	rs11993401	chr8:18657427	0.01566215	0.824692864	PSD3	ENSG00000156011.16	Brain - Substantia nigra	C=0.504	T=0.496	0.264322	3.14E-06
rs111241636	rs11993467	chr8:18657661	0.015586679	0.824340842	PSD3	ENSG00000156011.16	Brain - Substantia nigra	C=0.505	T=0.495	0.264322	3.14E-06
rs111241636	rs17696125	chr8:18574874	0.001070168	1	PSD3	ENSG00000156011.16	Small Intestine - Terminal Ileum	T=0.956	C=0.044	0.410081	3.38E-06
rs111241636	rs7841096	chr8:18632333	0.037581897	1	PSD3	ENSG00000156011.16	Brain - Spinal cord (cervical c-1)	A=0.384	T=0.616	0.378279	3.44E-06
rs111241636	rs59321198	chr8:18866979	0.002784828	1	PSD3	ENSG00000156011.16	Esophagus - Mucosa	C=0.894	G=0.106	0.217409	3.55E-06
rs111241636	rs78656518	chr8:18844514	0.000140387	1	PSD3	ENSG00000156011.16	Esophagus - Muscularis	T=0.994	C=0.006	0.494999	4.95E-06
rs111241636	rs111699729	chr8:18865650	0.002784828	1	PSD3	ENSG00000156011.16	Esophagus - Mucosa	G=0.894	A=0.106	0.213434	5.55E-06
rs111241636	rs7010324	chr8:18866227	0.002697746	1	PSD3	ENSG00000156011.16	Esophagus - Mucosa	C=0.897	T=0.103	0.213434	5.55E-06
rs111241636	rs6996112	chr8:18866326	0.002813985	1	PSD3	ENSG00000156011.16	Esophagus - Mucosa	T=0.893	C=0.107	0.213434	5.55E-06
rs111241636	rs7010496	chr8:18866368	0.002784828	1	PSD3	ENSG00000156011.16	Esophagus - Mucosa	C=0.894	T=0.106	0.213434	5.55E-06
rs111241636	rs117966479	chr8:18893665	0.00127535	1	PSD3	ENSG00000156011.16	Small Intestine - Terminal Ileum	T=0.948	C=0.052	0.630419	5.82E-06

rs111241636	rs77386793	chr8:18869663	0.002784828	1	PSD3	ENSG00000156011.16	Esophagus - Mucosa	C=0.894	A=0.106	0.225491	6.32E-06	
rs111241636	rs34426360	chr8:18575404	0.00104476	1	PSD3	ENSG00000156011.16	Small Intestine - Terminal Ileum	-=0.957	AGA=0.043	0.457749	7.58E-06	
rs111241636	rs1872890	chr8:18575581	0.001019405	1	PSD3	ENSG00000156011.16	Small Intestine - Terminal Ileum	A=0.958	G=0.042	0.457749	7.58E-06	
rs111241636	rs1038611	chr8:18624327	0.041090316	1	PSD3	ENSG00000156011.16	Brain - Spinal cord (cervical c-1)	C=0.363	T=0.637	0.378354	7.96E-06	
rs111241636	rs1038610	chr8:18624424	0.041090316	1	PSD3	ENSG00000156011.16	Brain - Spinal cord (cervical c-1)	C=0.363	T=0.637	0.378354	7.96E-06	
rs111241636	rs146517899	chr8:18867154	0.002813985	1	PSD3	ENSG00000156011.16	Esophagus - Mucosa	C=0.893	T=0.107	0.210362	8.66E-06	
rs111241636	rs56990136	chr8:18872866	0.002843206	1	PSD3	ENSG00000156011.16	Esophagus - Mucosa	C=0.892	T=0.108	0.21699	9.52E-06	
rs111241636	rs73594648	chr8:18876592	0.002843206	1	PSD3	ENSG00000156011.16	Esophagus - Mucosa	A=0.892	T=0.108	0.208395	1.24E-05	
rs111241636	rs141114042	chr8:18631690	0.038382155	1	PSD3	ENSG00000156011.16	Brain - Spinal cord (cervical c-1)	TTAA=0.379	-=0.621	0.367347	1.35E-05	
rs111241636	rs116324874	chr8:18876557	0.002843206	1	PSD3	ENSG00000156011.16	Esophagus - Mucosa	G=0.892	A=0.108	0.219157	1.52E-05	
rs111241636	rs80027157	chr8:18866078	0.002843206	1	PSD3	ENSG00000156011.16	Esophagus - Mucosa	A=0.892	G=0.108	0.208302	1.57E-05	
rs111241636	rs7009185	chr8:18866220	0.002784828	1	PSD3	ENSG00000156011.16	Esophagus - Mucosa	G=0.894	A=0.106	0.208302	1.57E-05	
rs111241636	rs7010821	chr8:18866541	0.002784828	1	PSD3	ENSG00000156011.16	Esophagus - Mucosa	C=0.894	T=0.106	0.208302	1.57E-05	
rs111241636	rs375703979	chr8:18866779	0.002784828	1	PSD3	ENSG00000156011.16	Esophagus - Mucosa	AG=0.894	-=0.106	0.208302	1.57E-05	
rs111241636	rs35675493	chr8:18653677	0.022485569	1	PSD3	ENSG00000156011.16	Brain - Spinal cord (cervical c-1)	CCC=0.51	-=0.49	0.381151	1.59E-05	
rs111241636	rs9644614	chr8:18630435	0.041985984	1	PSD3	ENSG00000156011.16	Brain - Spinal cord (cervical c-1)	C=0.358	T=0.642	0.374175	1.59E-05	
rs111241636	rs9644615	chr8:18630532	0.041804866	1	PSD3	ENSG00000156011.16	Brain - Spinal cord (cervical c-1)	C=0.359	A=0.641	0.374175	1.59E-05	
rs111241636	rs189718418	chr8:18857486	0.000116872	1	PSD3	ENSG00000156011.16	Esophagus - Muscularis	A=0.995	G=0.005	0.496246	1.84E-05	
rs111241636	rs2410598	chr8:18872462	0.002843206	1	PSD3	ENSG00000156011.16	Esophagus - Mucosa	C=0.892	T=0.108	0.204344	1.89E-05	
rs111241636	rs57855654	chr8:18872943	0.002843206	1	PSD3	ENSG00000156011.16	Esophagus - Mucosa	G=0.892	A=0.108	0.204344	1.89E-05	
rs111241636	rs76653031	chr8:18574691	0.000426275	1	PSD3	ENSG00000156011.16	Small Intestine - Terminal Ileum	C=0.982	A=0.018	0.614718	2.15E-05	
rs111241636	rs35592282	chr8:18576601	0.000426275	1	PSD3	ENSG00000156011.16	Small Intestine - Terminal Ileum	A=0.982	-=0.018	0.614718	2.15E-05	
rs111241636	rs143185883	chr8:18577856	0.000426275	1	PSD3	ENSG00000156011.16	Small Intestine - Terminal Ileum	C=0.982	-=0.018	0.614718	2.15E-05	
rs111241636	rs79697401	chr8:18578261	0.000426275	1	PSD3	ENSG00000156011.16	Small Intestine - Terminal Ileum	T=0.982	C=0.018	0.614718	2.15E-05	
rs111241636	rs17127092	chr8:18579361	0.000426275	1	PSD3	ENSG00000156011.16	Small Intestine - Terminal Ileum	A=0.982	G=0.018	0.614718	2.15E-05	
rs111241636	rs12056418	chr8:18580835	0.000426275	1	PSD3	ENSG00000156011.16	Small Intestine - Terminal Ileum	C=0.982	T=0.018	0.614718	2.15E-05	
rs111241636	rs76973373	chr8:18583218	0.000426275	1	PSD3	ENSG00000156011.16	Small Intestine - Terminal Ileum	C=0.982	T=0.018	0.614718	2.15E-05	
rs111241636	rs79550828	chr8:18587562	0.000426275	1	PSD3	ENSG00000156011.16	Small Intestine - Terminal Ileum	G=0.982	C=0.018	0.614718	2.15E-05	
rs111241636	rs79052288	chr8:18589608	0.000426275	1	PSD3	ENSG00000156011.16	Small Intestine - Terminal Ileum	C=0.982	G=0.018	0.614718	2.15E-05	
rs111241636	rs57825650	chr8:18591403	0.000426275	1	PSD3	ENSG00000156011.16	Small Intestine - Terminal Ileum	A=0.982	G=0.018	0.614718	2.15E-05	
rs111241636	rs11784567	chr8:18835526	0.000378146	1	PSD3	ENSG00000156011.16	Esophagus - Gastroesophageal Junctio	C=0.984	T=0.016	0.526419	3.09E-05	
rs111241636	rs144493102	chr8:18882143	0.000306315	1	PSD3	ENSG00000156011.16	Esophagus - Gastroesophageal Junctio	G=0.987	C=0.013	0.526419	3.09E-05	
rs111241636	rs60549282	chr8:18877472	0.002697746	1	PSD3	ENSG00000156011.16	Esophagus - Mucosa	C=0.897	T=0.103	0.198321	3.37E-05	
rs111241636	rs4463468	chr8:18879717	0.002697746	1	PSD3	ENSG00000156011.16	Esophagus - Mucosa	C=0.897	T=0.103	0.198321	3.37E-05	
rs111241636	rs75123216	chr8:18821251	0.000426275	1	PSD3	ENSG00000156011.16	Esophagus - Muscularis	T=0.982	C=0.018	0.413555	3.38E-05	
rs111241636	rs116971711	chr8:18822209	0.000426275	1	PSD3	ENSG00000156011.16	Esophagus - Muscularis	G=0.982	A=0.018	0.413555	3.38E-05	
rs111241636	rs77537870	chr8:18877244	0.002843206	1	PSD3	ENSG00000156011.16	Esophagus - Mucosa	A=0.892	G=0.108	0.202602	3.49E-05	
rs111241636	rs61050926	chr8:18864611	0.002813985	1	PSD3	ENSG00000156011.16	Esophagus - Mucosa	G=0.893	A=0.107	0.185851	4.35E-05	
rs111241636	rs57965775	chr8:18865248	0.002813985	1	PSD3	ENSG00000156011.16	Esophagus - Mucosa	C=0.893	G=0.107	0.185851	4.35E-05	
rs111241636	rs73199994	chr8:18646365	0.002048885	1	PSD3	ENSG00000156011.16	Brain - Cerebellum	C=0.919	T=0.081	0.375659	4.63E-05	
rs111241636	rs76595146	chr8:18873146	0.002843206	1	PSD3	ENSG00000156011.16	Esophagus - Mucosa	G=0.892	T=0.108	0.198351	5.28E-05	
rs111241636	rs79513952	chr8:18875713	0.002843206	1	PSD3	ENSG00000156011.16	Esophagus - Mucosa	G=0.892	A=0.108	0.198351	5.28E-05	
rs111241636	rs115432345	chr8:18871863	0.002872493	1	PSD3	ENSG00000156011.16	Esophagus - Mucosa	G=0.891	T=0.109	0.194814	6.45E-05	
rs111241636	rs73594633	chr8:18871992	0.002843206	1	PSD3	ENSG00000156011.16	Esophagus - Mucosa	C=0.892	G=0.108	0.189659	6.52E-05	
rs111241636	rs145752806	chr8:18882886	0.002697746	1	PSD3	ENSG00000156011.16	Esophagus - Mucosa	ACTC=0.897	-=0.103	0.194717	7.13E-05	
rs111241636	rs78119471	chr8:18883327	0.002697746	1	PSD3	ENSG00000156011.16	Esophagus - Mucosa	A=0.897	G=0.103	0.194717	7.13E-05	
rs111241636	rs60074764	chr8:18863855	0.002843206	1	PSD3	ENSG00000156011.16	Esophagus - Mucosa	T=0.892	C=0.108	0.173434	8.93E-05	
rs111241636	rs60016927	chr8:18863989	0.002843206	1	PSD3	ENSG00000156011.16	Esophagus - Mucosa	T=0.892	C=0.108	0.173434	8.93E-05	
rs111241636	rs79233079	chr8:19029881	0.002901845	1	PSD3	ENSG00000156011.16	Cells - Cultured fibroblasts	C=0.89	T=0.11	-0.168053	0.000116145	
rs112738876	rs138983771	chr8:18919610	0.000523778	1	PSD3	ENSG00000156011.16	Esophagus - Muscularis	G=0.986	A=0.014	0.754799	1.43E-07	
rs112738876	rs111916415	chr8:18656221	0.032008063	0.847298118	PSD3	ENSG00000156011.16	Brain - Spinal cord (cervical c-1)	A=0.454	T=0.546	0.411994	2.93E-06	

rs112738876	rs78656518	chr8:18844514	0.00022268	1	PSD3	ENSG00000156011.16	Esophagus - Muscularis	T=0.994	C=0.006	0.494999	4.95E-06
rs112738876	rs117966479	chr8:18893665	0.002022953	1	PSD3	ENSG00000156011.16	Small Intestine - Terminal Ileum	T=0.948	C=0.052	0.630419	5.82E-06
rs112738876	rs141114042	chr8:18631690	0.060881565	1	PSD3	ENSG00000156011.16	Brain - Spinal cord (cervical c-1)	TTAA=0.379	-=0.621	0.367347	1.35E-05
rs112738876	rs35675493	chr8:18653677	0.031737752	0.943317557	PSD3	ENSG00000156011.16	Brain - Spinal cord (cervical c-1)	CCC=0.51	-=0.49	0.381151	1.59E-05
rs112738876	rs189718418	chr8:18857486	0.000185382	1	PSD3	ENSG00000156011.16	Esophagus - Muscularis	A=0.995	G=0.005	0.496246	1.84E-05
rs112738876	rs75123216	chr8:18821251	0.000676155	1	PSD3	ENSG00000156011.16	Esophagus - Muscularis	T=0.982	C=0.018	0.413555	3.38E-05
rs112738876	rs116971711	chr8:18822209	0.000676155	1	PSD3	ENSG00000156011.16	Esophagus - Muscularis	G=0.982	A=0.018	0.413555	3.38E-05
rs112738876	rs73199994	chr8:18646365	0.00324993	1	PSD3	ENSG00000156011.16	Brain - Cerebellum	C=0.919	T=0.081	0.375659	4.63E-05
rs12676642	rs547180436	chr8:18871342	0.000172581	1	PSD3	ENSG00000156011.16	Esophagus - Muscularis	-=0.99	A=0.01	0.41154	8.21E-19
rs12676642	rs2632845	chr8:18660720	0.015890538	0.800752624	PSD3	ENSG00000156011.16	Brain - Spinal cord (cervical c-1)	C=0.59	G=0.41	-0.519542	3.39E-10
rs12676642	rs2638625	chr8:18659269	0.015969366	0.801087494	PSD3	ENSG00000156011.16	Brain - Spinal cord (cervical c-1)	C=0.591	T=0.409	-0.506856	1.08E-09
rs12676642	rs547180436	chr8:18871342	0.000172581	1	PSD3	ENSG00000156011.16	Esophagus - Gastroesophageal Junctio	-=0.99	A=0.01	0.267169	5.99E-09
rs12676642	rs10088636	chr8:18355945	0.00213183	1	PSD3	ENSG00000156011.16	Brain - Cerebellar Hemisphere	G=0.89	T=0.11	-0.381687	2.71E-08
rs12676642	rs7009615	chr8:18572835	0.001960659	1	PSD3	ENSG00000156011.16	Small Intestine - Terminal Ileum	T=0.898	C=0.102	0.37749	1.38E-07
rs12676642	rs138983771	chr8:18919610	0.000242588	1	PSD3	ENSG00000156011.16	Esophagus - Muscularis	G=0.986	A=0.014	0.754799	1.43E-07
rs12676642	rs3214999	chr8:18656888	0.031741385	0.837650286	PSD3	ENSG00000156011.16	Brain - Substantia nigra	G=0.725	-=0.275	-0.269949	2.63E-07
rs12676642	rs10503636	chr8:18654794	0.014315101	0.885759709	PSD3	ENSG00000156011.16	Brain - Spinal cord (cervical c-1)	T=0.485	C=0.515	0.423645	8.47E-07
rs12676642	rs3214999	chr8:18656888	0.031741385	0.837650286	PSD3	ENSG00000156011.16	Brain - Spinal cord (cervical c-1)	G=0.725	-=0.275	-0.461094	2.06E-06
rs12676642	rs60854172	chr8:18867056	0.002067284	1	PSD3	ENSG00000156011.16	Esophagus - Mucosa	-=0.893	A=0.107	0.215939	2.93E-06
rs12676642	rs111916415	chr8:18656221	0.020649464	1	PSD3	ENSG00000156011.16	Brain - Spinal cord (cervical c-1)	A=0.454	T=0.546	0.411994	2.93E-06
rs12676642	rs2632845	chr8:18660720	0.015890538	0.800752624	PSD3	ENSG00000156011.16	Brain - Substantia nigra	C=0.59	G=0.41	-0.241026	3.37E-06
rs12676642	rs17696125	chr8:18574874	0.000786195	1	PSD3	ENSG00000156011.16	Small Intestine - Terminal Ileum	T=0.956	C=0.044	0.410081	3.38E-06
rs12676642	rs7841096	chr8:18632333	0.027609403	1	PSD3	ENSG00000156011.16	Brain - Spinal cord (cervical c-1)	A=0.384	T=0.616	0.378279	3.44E-06
rs12676642	rs59321198	chr8:18866979	0.002045864	1	PSD3	ENSG00000156011.16	Esophagus - Mucosa	C=0.894	G=0.106	0.217409	3.55E-06
rs12676642	rs2638625	chr8:18659269	0.015969366	0.801087494	PSD3	ENSG00000156011.16	Brain - Substantia nigra	C=0.591	T=0.409	-0.23662	4.89E-06
rs12676642	rs78656518	chr8:18844514	0.000103134	1	PSD3	ENSG00000156011.16	Esophagus - Muscularis	T=0.994	C=0.006	0.494999	4.95E-06
rs12676642	rs111699729	chr8:18865650	0.002045864	1	PSD3	ENSG00000156011.16	Esophagus - Mucosa	G=0.894	A=0.106	0.213434	5.55E-06
rs12676642	rs7010324	chr8:18866227	0.001981889	1	PSD3	ENSG00000156011.16	Esophagus - Mucosa	C=0.897	T=0.103	0.213434	5.55E-06
rs12676642	rs6996112	chr8:18866326	0.002067284	1	PSD3	ENSG00000156011.16	Esophagus - Mucosa	T=0.893	C=0.107	0.213434	5.55E-06
rs12676642	rs7010496	chr8:18866368	0.002045864	1	PSD3	ENSG00000156011.16	Esophagus - Mucosa	C=0.894	T=0.106	0.213434	5.55E-06
rs12676642	rs117966479	chr8:18893665	0.000936931	1	PSD3	ENSG00000156011.16	Small Intestine - Terminal Ileum	T=0.948	C=0.052	0.630419	5.82E-06
rs12676642	rs77386793	chr8:18869663	0.002045864	1	PSD3	ENSG00000156011.16	Esophagus - Mucosa	C=0.894	A=0.106	0.225491	6.32E-06
rs12676642	rs34426360	chr8:18575404	0.000767529	1	PSD3	ENSG00000156011.16	Small Intestine - Terminal Ileum	-=0.957	AGA=0.043	0.457749	7.58E-06
rs12676642	rs1872890	chr8:18575581	0.000748902	1	PSD3	ENSG00000156011.16	Small Intestine - Terminal Ileum	A=0.958	G=0.042	0.457749	7.58E-06
rs12676642	rs1038611	chr8:18624327	0.03018685	1	PSD3	ENSG00000156011.16	Brain - Spinal cord (cervical c-1)	C=0.363	T=0.637	0.378354	7.96E-06
rs12676642	rs1038610	chr8:18624424	0.03018685	1	PSD3	ENSG00000156011.16	Brain - Spinal cord (cervical c-1)	C=0.363	T=0.637	0.378354	7.96E-06
rs12676642	rs146517899	chr8:18867154	0.002067284	1	PSD3	ENSG00000156011.16	Esophagus - Mucosa	C=0.893	T=0.107	0.210362	8.66E-06
rs12676642	rs56990136	chr8:18872866	0.002088751	1	PSD3	ENSG00000156011.16	Esophagus - Mucosa	C=0.892	T=0.108	0.21699	9.52E-06
rs12676642	rs73594648	chr8:18876592	0.002088751	1	PSD3	ENSG00000156011.16	Esophagus - Mucosa	A=0.892	T=0.108	0.208395	1.24E-05
rs12676642	rs141114042	chr8:18631690	0.02819731	1	PSD3	ENSG00000156011.16	Brain - Spinal cord (cervical c-1)	TTAA=0.379	-=0.621	0.367347	1.35E-05
rs12676642	rs116324874	chr8:18876557	0.002088751	1	PSD3	ENSG00000156011.16	Esophagus - Mucosa	G=0.892	A=0.108	0.219157	1.52E-05
rs12676642	rs80027157	chr8:18866078	0.002088751	1	PSD3	ENSG00000156011.16	Esophagus - Mucosa	A=0.892	G=0.108	0.208302	1.57E-05
rs12676642	rs7009185	chr8:18866220	0.002045864	1	PSD3	ENSG00000156011.16	Esophagus - Mucosa	G=0.894	A=0.106	0.208302	1.57E-05
rs12676642	rs7010821	chr8:18866541	0.002045864	1	PSD3	ENSG00000156011.16	Esophagus - Mucosa	C=0.894	T=0.106	0.208302	1.57E-05
rs12676642	rs375703979	chr8:18866779	0.002045864	1	PSD3	ENSG00000156011.16	Esophagus - Mucosa	AG=0.894	-=0.106	0.208302	1.57E-05
rs12676642	rs35675493	chr8:18653677	0.01651894	1	PSD3	ENSG00000156011.16	Brain - Spinal cord (cervical c-1)	CCC=0.51	-=0.49	0.381151	1.59E-05
rs12676642	rs9644614	chr8:18630435	0.030844849	1	PSD3	ENSG00000156011.16	Brain - Spinal cord (cervical c-1)	C=0.358	T=0.642	0.374175	1.59E-05
rs12676642	rs9644615	chr8:18630532	0.030711791	1	PSD3	ENSG00000156011.16	Brain - Spinal cord (cervical c-1)	C=0.359	A=0.641	0.374175	1.59E-05
rs12676642	rs189718418	chr8:18857486	8.59E-05	1	PSD3	ENSG00000156011.16	Esophagus - Muscularis	A=0.995	G=0.005	0.496246	1.84E-05
rs12676642	rs2410598	chr8:18872462	0.002088751	1	PSD3	ENSG00000156011.16	Esophagus - Mucosa	C=0.892	T=0.108	0.204344	1.89E-05
rs12676642	rs57855654	chr8:18872943	0.002088751	1	PSD3	ENSG00000156011.16	Esophagus - Mucosa	G=0.892	A=0.108	0.204344	1.89E-05

rs12676642	rs335232	chr8:18636126	0.007656056	1	PSD3	ENSG00000156011.16	Brain - Cerebellum	G=0.692	A=0.308	0.211844	2.05E-05
rs12676642	rs10111009	chr8:18973729	0.003873301	1	PSD3	ENSG00000156011.16	Skin - Sun Exposed (Lower leg)	C=0.816	T=0.184	0.172705	2.10E-05
rs12676642	rs76653031	chr8:18574691	0.000313161	1	PSD3	ENSG00000156011.16	Small Intestine - Terminal Ileum	C=0.982	A=0.018	0.614718	2.15E-05
rs12676642	rs35592282	chr8:18576601	0.000313161	1	PSD3	ENSG00000156011.16	Small Intestine - Terminal Ileum	A=0.982	-=0.018	0.614718	2.15E-05
rs12676642	rs143185883	chr8:18577856	0.000313161	1	PSD3	ENSG00000156011.16	Small Intestine - Terminal Ileum	C=0.982	-=0.018	0.614718	2.15E-05
rs12676642	rs79697401	chr8:18578261	0.000313161	1	PSD3	ENSG00000156011.16	Small Intestine - Terminal Ileum	T=0.982	C=0.018	0.614718	2.15E-05
rs12676642	rs17127092	chr8:18579361	0.000313161	1	PSD3	ENSG00000156011.16	Small Intestine - Terminal Ileum	A=0.982	G=0.018	0.614718	2.15E-05
rs12676642	rs12056418	chr8:18580835	0.000313161	1	PSD3	ENSG00000156011.16	Small Intestine - Terminal Ileum	C=0.982	T=0.018	0.614718	2.15E-05
rs12676642	rs76973373	chr8:18583218	0.000313161	1	PSD3	ENSG00000156011.16	Small Intestine - Terminal Ileum	C=0.982	T=0.018	0.614718	2.15E-05
rs12676642	rs79550828	chr8:18587562	0.000313161	1	PSD3	ENSG00000156011.16	Small Intestine - Terminal Ileum	G=0.982	C=0.018	0.614718	2.15E-05
rs12676642	rs79052288	chr8:18589608	0.000313161	1	PSD3	ENSG00000156011.16	Small Intestine - Terminal Ileum	C=0.982	G=0.018	0.614718	2.15E-05
rs12676642	rs57825650	chr8:18591403	0.000313161	1	PSD3	ENSG00000156011.16	Small Intestine - Terminal Ileum	A=0.982	G=0.018	0.614718	2.15E-05
rs12676642	rs335234	chr8:18637444	0.007835832	1	PSD3	ENSG00000156011.16	Brain - Cerebellum	C=0.687	T=0.313	0.2134	2.22E-05
rs12676642	rs6995932	chr8:18362067	0.003372412	1	PSD3	ENSG00000156011.16	Brain - Cerebellar Hemisphere	G=0.836	A=0.164	-0.253274	2.38E-05
rs12676642	rs60549282	chr8:18877472	0.001981889	1	PSD3	ENSG00000156011.16	Esophagus - Mucosa	C=0.897	T=0.103	0.198321	3.37E-05
rs12676642	rs4463468	chr8:18879717	0.001981889	1	PSD3	ENSG00000156011.16	Esophagus - Mucosa	C=0.897	T=0.103	0.198321	3.37E-05
rs12676642	rs75123216	chr8:18821251	0.000313161	1	PSD3	ENSG00000156011.16	Esophagus - Muscularis	T=0.982	C=0.018	0.413555	3.38E-05
rs12676642	rs116971711	chr8:18822209	0.000313161	1	PSD3	ENSG00000156011.16	Esophagus - Muscularis	G=0.982	A=0.018	0.413555	3.38E-05
rs12676642	rs335248	chr8:18646733	0.007691804	1	PSD3	ENSG00000156011.16	Brain - Cerebellum	G=0.691	C=0.309	0.203295	3.48E-05
rs12676642	rs77537870	chr8:18877244	0.002088751	1	PSD3	ENSG00000156011.16	Esophagus - Mucosa	A=0.892	G=0.108	0.202602	3.49E-05
rs12676642	rs335237	chr8:18641291	0.009051003	1	PSD3	ENSG00000156011.16	Brain - Cerebellum	T=0.655	C=0.345	0.200677	3.52E-05
rs12676642	rs61050926	chr8:18864611	0.002067284	1	PSD3	ENSG00000156011.16	Esophagus - Mucosa	G=0.893	A=0.107	0.185851	4.35E-05
rs12676642	rs57965775	chr8:18865248	0.002067284	1	PSD3	ENSG00000156011.16	Esophagus - Mucosa	C=0.893	G=0.107	0.185851	4.35E-05
rs12676642	rs73199994	chr8:18646365	0.001505206	1	PSD3	ENSG00000156011.16	Brain - Cerebellum	C=0.919	T=0.081	0.375659	4.63E-05
rs12676642	rs6995932	chr8:18362067	0.003372412	1	PSD3	ENSG00000156011.16	Brain - Cerebellum	G=0.836	A=0.164	-0.26983	4.92E-05
rs12676642	rs76595146	chr8:18873146	0.002088751	1	PSD3	ENSG00000156011.16	Esophagus - Mucosa	G=0.892	T=0.108	0.198351	5.28E-05
rs12676642	rs79513952	chr8:18875713	0.002088751	1	PSD3	ENSG00000156011.16	Esophagus - Mucosa	G=0.892	A=0.108	0.198351	5.28E-05
rs12676642	rs115432345	chr8:18871863	0.002110267	1	PSD3	ENSG00000156011.16	Esophagus - Mucosa	G=0.891	T=0.109	0.194814	6.45E-05
rs12676642	rs73594633	chr8:18871992	0.002088751	1	PSD3	ENSG00000156011.16	Esophagus - Mucosa	C=0.892	G=0.108	0.189659	6.52E-05
rs12676642	rs145752806	chr8:18882886	0.001981889	1	PSD3	ENSG00000156011.16	Esophagus - Mucosa	ACTC=0.897	-=0.103	0.194717	7.13E-05
rs12676642	rs78119471	chr8:18883327	0.001981889	1	PSD3	ENSG00000156011.16	Esophagus - Mucosa	A=0.897	G=0.103	0.194717	7.13E-05
rs12676642	rs60074764	chr8:18863855	0.002088751	1	PSD3	ENSG00000156011.16	Esophagus - Mucosa	T=0.892	C=0.108	0.173434	8.93E-05
rs12676642	rs60016927	chr8:18863989	0.002088751	1	PSD3	ENSG00000156011.16	Esophagus - Mucosa	T=0.892	C=0.108	0.173434	8.93E-05
rs12676642	rs79233079	chr8:19029881	0.00213183	1	PSD3	ENSG00000156011.16	Cells - Cultured fibroblasts	C=0.89	T=0.11	-0.168053	0.000116145
rs12677280	rs547180436	chr8:18871342	0.000308611	1	PSD3	ENSG00000156011.16	Esophagus - Muscularis	-=0.99	A=0.01	0.41154	8.21E-19
rs12677280	rs547180436	chr8:18871342	0.000308611	1	PSD3	ENSG00000156011.16	Esophagus - Gastroesophageal Junctio	-=0.99	A=0.01	0.267169	5.99E-09
rs12677280	rs138983771	chr8:18919610	0.000433798	1	PSD3	ENSG00000156011.16	Esophagus - Muscularis	G=0.986	A=0.014	0.754799	1.43E-07
rs12677280	rs10503636	chr8:18654794	0.021184916	0.805791506	PSD3	ENSG00000156011.16	Brain - Spinal cord (cervical c-1)	T=0.485	C=0.515	0.423645	8.47E-07
rs12677280	rs111916415	chr8:18656221	0.028454884	0.877838494	PSD3	ENSG00000156011.16	Brain - Spinal cord (cervical c-1)	A=0.454	T=0.546	0.411994	2.93E-06
rs12677280	rs78656518	chr8:18844514	0.000184426	1	PSD3	ENSG00000156011.16	Esophagus - Muscularis	T=0.994	C=0.006	0.494999	4.95E-06
rs12677280	rs117966479	chr8:18893665	0.00167543	1	PSD3	ENSG00000156011.16	Small Intestine - Terminal Ileum	T=0.948	C=0.052	0.630419	5.82E-06
rs12677280	rs141114042	chr8:18631690	0.050422744	1	PSD3	ENSG00000156011.16	Brain - Spinal cord (cervical c-1)	TTAA=0.379	-=0.621	0.367347	1.35E-05
rs12677280	rs35675493	chr8:18653677	0.025657549	0.931981068	PSD3	ENSG00000156011.16	Brain - Spinal cord (cervical c-1)	CCC=0.51	-=0.49	0.381151	1.59E-05
rs12677280	rs189718418	chr8:18857486	0.000153535	1	PSD3	ENSG00000156011.16	Esophagus - Muscularis	A=0.995	G=0.005	0.496246	1.84E-05
rs12677280	rs335232	chr8:18636126	0.013690644	1	PSD3	ENSG00000156011.16	Brain - Cerebellum	G=0.692	A=0.308	0.211844	2.05E-05
rs12677280	rs10111009	chr8:18973729	0.006926279	1	PSD3	ENSG00000156011.16	Skin - Sun Exposed (Lower leg)	C=0.816	T=0.184	0.172705	2.10E-05
rs12677280	rs335234	chr8:18637444	0.014012123	1	PSD3	ENSG00000156011.16	Brain - Cerebellum	C=0.687	T=0.313	0.2134	2.22E-05
rs12677280	rs75123216	chr8:18821251	0.000559999	1	PSD3	ENSG00000156011.16	Esophagus - Muscularis	T=0.982	C=0.018	0.413555	3.38E-05
rs12677280	rs116971711	chr8:18822209	0.000559999	1	PSD3	ENSG00000156011.16	Esophagus - Muscularis	G=0.982	A=0.018	0.413555	3.38E-05
rs12677280	rs335248	chr8:18646733	0.01375457	1	PSD3	ENSG00000156011.16	Brain - Cerebellum	G=0.691	C=0.309	0.203295	3.48E-05
rs12677280	rs335237	chr8:18641291	0.016185104	1	PSD3	ENSG00000156011.16	Brain - Cerebellum	T=0.655	C=0.345	0.200677	3.52E-05

rs12677280	rs73199994	chr8:18646365	0.002691626	1	PSD3	ENSG00000156011.16	Brain - Cerebellum	C=0.919	T=0.081	0.375659	4.63E-05
rs13439022	rs547180436	chr8:18871342	0.000172581	1	PSD3	ENSG00000156011.16	Esophagus - Muscularis	-=0.99	A=0.01	0.41154	8.21E-19
rs13439022	rs2632845	chr8:18660720	0.015890538	0.800752624	PSD3	ENSG00000156011.16	Brain - Spinal cord (cervical c-1)	C=0.59	G=0.41	-0.519542	3.39E-10
rs13439022	rs2638625	chr8:18659269	0.015969366	0.801087494	PSD3	ENSG00000156011.16	Brain - Spinal cord (cervical c-1)	C=0.591	T=0.409	-0.506856	1.08E-09
rs13439022	rs547180436	chr8:18871342	0.000172581	1	PSD3	ENSG00000156011.16	Esophagus - Gastroesophageal Junctio	-=0.99	A=0.01	0.267169	5.99E-09
rs13439022	rs10088636	chr8:18355945	0.00213183	1	PSD3	ENSG00000156011.16	Brain - Cerebellar Hemisphere	G=0.89	T=0.11	-0.381687	2.71E-08
rs13439022	rs7009615	chr8:18572835	0.001960659	1	PSD3	ENSG00000156011.16	Small Intestine - Terminal Ileum	T=0.898	C=0.102	0.37749	1.38E-07
rs13439022	rs138983771	chr8:18919610	0.000242588	1	PSD3	ENSG00000156011.16	Esophagus - Muscularis	G=0.986	A=0.014	0.754799	1.43E-07
rs13439022	rs3214999	chr8:18656888	0.031741385	0.837650286	PSD3	ENSG00000156011.16	Brain - Substantia nigra	G=0.725	-=0.275	-0.269949	2.63E-07
rs13439022	rs10503636	chr8:18654794	0.014315101	0.885759709	PSD3	ENSG00000156011.16	Brain - Spinal cord (cervical c-1)	T=0.485	C=0.515	0.423645	8.47E-07
rs13439022	rs3214999	chr8:18656888	0.031741385	0.837650286	PSD3	ENSG00000156011.16	Brain - Spinal cord (cervical c-1)	G=0.725	-=0.275	-0.461094	2.06E-06
rs13439022	rs60854172	chr8:18867056	0.002067284	1	PSD3	ENSG00000156011.16	Esophagus - Mucosa	-=0.893	A=0.107	0.215939	2.93E-06
rs13439022	rs111916415	chr8:18656221	0.020649464	1	PSD3	ENSG00000156011.16	Brain - Spinal cord (cervical c-1)	A=0.454	T=0.546	0.411994	2.93E-06
rs13439022	rs2632845	chr8:18660720	0.015890538	0.800752624	PSD3	ENSG00000156011.16	Brain - Substantia nigra	C=0.59	G=0.41	-0.241026	3.37E-06
rs13439022	rs17696125	chr8:18574874	0.000786195	1	PSD3	ENSG00000156011.16	Small Intestine - Terminal Ileum	T=0.956	C=0.044	0.410081	3.38E-06
rs13439022	rs7841096	chr8:18632333	0.027609403	1	PSD3	ENSG00000156011.16	Brain - Spinal cord (cervical c-1)	A=0.384	T=0.616	0.378279	3.44E-06
rs13439022	rs59321198	chr8:18866979	0.002045864	1	PSD3	ENSG00000156011.16	Esophagus - Mucosa	C=0.894	G=0.106	0.217409	3.55E-06
rs13439022	rs2638625	chr8:18659269	0.015969366	0.801087494	PSD3	ENSG00000156011.16	Brain - Substantia nigra	C=0.591	T=0.409	-0.23662	4.89E-06
rs13439022	rs78656518	chr8:18844514	0.000103134	1	PSD3	ENSG00000156011.16	Esophagus - Muscularis	T=0.994	C=0.006	0.494999	4.95E-06
rs13439022	rs111699729	chr8:18865650	0.002045864	1	PSD3	ENSG00000156011.16	Esophagus - Mucosa	G=0.894	A=0.106	0.213434	5.55E-06
rs13439022	rs7010324	chr8:18866227	0.001981889	1	PSD3	ENSG00000156011.16	Esophagus - Mucosa	C=0.897	T=0.103	0.213434	5.55E-06
rs13439022	rs6996112	chr8:18866326	0.002067284	1	PSD3	ENSG00000156011.16	Esophagus - Mucosa	T=0.893	C=0.107	0.213434	5.55E-06
rs13439022	rs7010496	chr8:18866368	0.002045864	1	PSD3	ENSG00000156011.16	Esophagus - Mucosa	C=0.894	T=0.106	0.213434	5.55E-06
rs13439022	rs117966479	chr8:18893665	0.000936931	1	PSD3	ENSG00000156011.16	Small Intestine - Terminal Ileum	T=0.948	C=0.052	0.630419	5.82E-06
rs13439022	rs77386793	chr8:18869663	0.002045864	1	PSD3	ENSG00000156011.16	Esophagus - Mucosa	C=0.894	A=0.106	0.225491	6.32E-06
rs13439022	rs34426360	chr8:18575404	0.000767529	1	PSD3	ENSG00000156011.16	Small Intestine - Terminal Ileum	-=0.957	AGA=0.043	0.457749	7.58E-06
rs13439022	rs1872890	chr8:18575581	0.000748902	1	PSD3	ENSG00000156011.16	Small Intestine - Terminal Ileum	A=0.958	G=0.042	0.457749	7.58E-06
rs13439022	rs1038611	chr8:18624327	0.03018685	1	PSD3	ENSG00000156011.16	Brain - Spinal cord (cervical c-1)	C=0.363	T=0.637	0.378354	7.96E-06
rs13439022	rs1038610	chr8:18624424	0.03018685	1	PSD3	ENSG00000156011.16	Brain - Spinal cord (cervical c-1)	C=0.363	T=0.637	0.378354	7.96E-06
rs13439022	rs146517899	chr8:18867154	0.002067284	1	PSD3	ENSG00000156011.16	Esophagus - Mucosa	C=0.893	T=0.107	0.210362	8.66E-06
rs13439022	rs56990136	chr8:18872866	0.002088751	1	PSD3	ENSG00000156011.16	Esophagus - Mucosa	C=0.892	T=0.108	0.21699	9.52E-06
rs13439022	rs73594648	chr8:18876592	0.002088751	1	PSD3	ENSG00000156011.16	Esophagus - Mucosa	A=0.892	T=0.108	0.208395	1.24E-05
rs13439022	rs141114042	chr8:18631690	0.02819731	1	PSD3	ENSG00000156011.16	Brain - Spinal cord (cervical c-1)	TTAA=0.379	-=0.621	0.367347	1.35E-05
rs13439022	rs116324874	chr8:18876557	0.002088751	1	PSD3	ENSG00000156011.16	Esophagus - Mucosa	G=0.892	A=0.108	0.219157	1.52E-05
rs13439022	rs80027157	chr8:18866078	0.002088751	1	PSD3	ENSG00000156011.16	Esophagus - Mucosa	A=0.892	G=0.108	0.208302	1.57E-05
rs13439022	rs7009185	chr8:18866220	0.002045864	1	PSD3	ENSG00000156011.16	Esophagus - Mucosa	G=0.894	A=0.106	0.208302	1.57E-05
rs13439022	rs7010821	chr8:18866541	0.002045864	1	PSD3	ENSG00000156011.16	Esophagus - Mucosa	C=0.894	T=0.106	0.208302	1.57E-05
rs13439022	rs375703979	chr8:18866779	0.002045864	1	PSD3	ENSG00000156011.16	Esophagus - Mucosa	AG=0.894	-=0.106	0.208302	1.57E-05
rs13439022	rs35675493	chr8:18653677	0.01651894	1	PSD3	ENSG00000156011.16	Brain - Spinal cord (cervical c-1)	CCC=0.51	-=0.49	0.381151	1.59E-05
rs13439022	rs9644614	chr8:18630435	0.030844849	1	PSD3	ENSG00000156011.16	Brain - Spinal cord (cervical c-1)	C=0.358	T=0.642	0.374175	1.59E-05
rs13439022	rs9644615	chr8:18630532	0.030711791	1	PSD3	ENSG00000156011.16	Brain - Spinal cord (cervical c-1)	C=0.359	A=0.641	0.374175	1.59E-05
rs13439022	rs189718418	chr8:18857486	8.59E-05	1	PSD3	ENSG00000156011.16	Esophagus - Muscularis	A=0.995	G=0.005	0.496246	1.84E-05
rs13439022	rs2410598	chr8:18872462	0.002088751	1	PSD3	ENSG00000156011.16	Esophagus - Mucosa	C=0.892	T=0.108	0.204344	1.89E-05
rs13439022	rs57855654	chr8:18872943	0.002088751	1	PSD3	ENSG00000156011.16	Esophagus - Mucosa	G=0.892	A=0.108	0.204344	1.89E-05
rs13439022	rs335232	chr8:18636126	0.007656056	1	PSD3	ENSG00000156011.16	Brain - Cerebellum	G=0.692	A=0.308	0.211844	2.05E-05
rs13439022	rs10111009	chr8:18973729	0.003873301	1	PSD3	ENSG00000156011.16	Skin - Sun Exposed (Lower leg)	C=0.816	T=0.184	0.172705	2.10E-05
rs13439022	rs76653031	chr8:18574691	0.000313161	1	PSD3	ENSG00000156011.16	Small Intestine - Terminal Ileum	C=0.982	A=0.018	0.614718	2.15E-05
rs13439022	rs35592282	chr8:18576601	0.000313161	1	PSD3	ENSG00000156011.16	Small Intestine - Terminal Ileum	A=0.982	-=0.018	0.614718	2.15E-05
rs13439022	rs143185883	chr8:18577856	0.000313161	1	PSD3	ENSG00000156011.16	Small Intestine - Terminal Ileum	C=0.982	-=0.018	0.614718	2.15E-05
rs13439022	rs79697401	chr8:18578261	0.000313161	1	PSD3	ENSG00000156011.16	Small Intestine - Terminal Ileum	T=0.982	C=0.018	0.614718	2.15E-05
rs13439022	rs17127092	chr8:18579361	0.000313161	1	PSD3	ENSG00000156011.16	Small Intestine - Terminal Ileum	A=0.982	G=0.018	0.614718	2.15E-05

rs13439022	rs12056418	chr8:18580835	0.000313161	1	PSD3	ENSG00000156011.16	Small Intestine - Terminal Ileum	C=0.982	T=0.018	0.614718	2.15E-05
rs13439022	rs76973373	chr8:18583218	0.000313161	1	PSD3	ENSG00000156011.16	Small Intestine - Terminal Ileum	C=0.982	T=0.018	0.614718	2.15E-05
rs13439022	rs79550828	chr8:18587562	0.000313161	1	PSD3	ENSG00000156011.16	Small Intestine - Terminal Ileum	G=0.982	C=0.018	0.614718	2.15E-05
rs13439022	rs79052288	chr8:18589608	0.000313161	1	PSD3	ENSG00000156011.16	Small Intestine - Terminal Ileum	C=0.982	G=0.018	0.614718	2.15E-05
rs13439022	rs57825650	chr8:18591403	0.000313161	1	PSD3	ENSG00000156011.16	Small Intestine - Terminal Ileum	A=0.982	G=0.018	0.614718	2.15E-05
rs13439022	rs335234	chr8:18637444	0.007835832	1	PSD3	ENSG00000156011.16	Brain - Cerebellum	C=0.687	T=0.313	0.2134	2.22E-05
rs13439022	rs6995932	chr8:18362067	0.003372412	1	PSD3	ENSG00000156011.16	Brain - Cerebellar Hemisphere	G=0.836	A=0.164	-0.253274	2.38E-05
rs13439022	rs60549282	chr8:18877472	0.001981889	1	PSD3	ENSG00000156011.16	Esophagus - Mucosa	C=0.897	T=0.103	0.198321	3.37E-05
rs13439022	rs4463468	chr8:18879717	0.001981889	1	PSD3	ENSG00000156011.16	Esophagus - Mucosa	C=0.897	T=0.103	0.198321	3.37E-05
rs13439022	rs75123216	chr8:18821251	0.000313161	1	PSD3	ENSG00000156011.16	Esophagus - Muscularis	T=0.982	C=0.018	0.413555	3.38E-05
rs13439022	rs116971711	chr8:18822209	0.000313161	1	PSD3	ENSG00000156011.16	Esophagus - Muscularis	G=0.982	A=0.018	0.413555	3.38E-05
rs13439022	rs335248	chr8:18646733	0.007691804	1	PSD3	ENSG00000156011.16	Brain - Cerebellum	G=0.691	C=0.309	0.203295	3.48E-05
rs13439022	rs77537870	chr8:18877244	0.002088751	1	PSD3	ENSG00000156011.16	Esophagus - Mucosa	A=0.892	G=0.108	0.202602	3.49E-05
rs13439022	rs335237	chr8:18641291	0.009051003	1	PSD3	ENSG00000156011.16	Brain - Cerebellum	T=0.655	C=0.345	0.200677	3.52E-05
rs13439022	rs61050926	chr8:18864611	0.002067284	1	PSD3	ENSG00000156011.16	Esophagus - Mucosa	G=0.893	A=0.107	0.185851	4.35E-05
rs13439022	rs57965775	chr8:18865248	0.002067284	1	PSD3	ENSG00000156011.16	Esophagus - Mucosa	C=0.893	G=0.107	0.185851	4.35E-05
rs13439022	rs73199994	chr8:18646365	0.001505206	1	PSD3	ENSG00000156011.16	Brain - Cerebellum	C=0.919	T=0.081	0.375659	4.63E-05
rs13439022	rs6995932	chr8:18362067	0.003372412	1	PSD3	ENSG00000156011.16	Brain - Cerebellum	G=0.836	A=0.164	-0.26983	4.92E-05
rs13439022	rs76595146	chr8:18873146	0.002088751	1	PSD3	ENSG00000156011.16	Esophagus - Mucosa	G=0.892	T=0.108	0.198351	5.28E-05
rs13439022	rs79513952	chr8:18875713	0.002088751	1	PSD3	ENSG00000156011.16	Esophagus - Mucosa	G=0.892	A=0.108	0.198351	5.28E-05
rs13439022	rs115432345	chr8:18871863	0.002110267	1	PSD3	ENSG00000156011.16	Esophagus - Mucosa	G=0.891	T=0.109	0.194814	6.45E-05
rs13439022	rs73594633	chr8:18871992	0.002088751	1	PSD3	ENSG00000156011.16	Esophagus - Mucosa	C=0.892	G=0.108	0.189659	6.52E-05
rs13439022	rs145752806	chr8:18882886	0.001981889	1	PSD3	ENSG00000156011.16	Esophagus - Mucosa	ACTC=0.897	-=0.103	0.194717	7.13E-05
rs13439022	rs78119471	chr8:18883327	0.001981889	1	PSD3	ENSG00000156011.16	Esophagus - Mucosa	A=0.897	G=0.103	0.194717	7.13E-05
rs13439022	rs60074764	chr8:18863855	0.002088751	1	PSD3	ENSG00000156011.16	Esophagus - Mucosa	T=0.892	C=0.108	0.173434	8.93E-05
rs13439022	rs60016927	chr8:18863989	0.002088751	1	PSD3	ENSG00000156011.16	Esophagus - Mucosa	T=0.892	C=0.108	0.173434	8.93E-05
rs13439022	rs79233079	chr8:19029881	0.00213183	1	PSD3	ENSG00000156011.16	Cells - Cultured fibroblasts	C=0.89	T=0.11	-0.168053	0.000116145
rs4921958	rs547180436	chr8:18871342	0.000182918	1	PSD3	ENSG00000156011.16	Esophagus - Muscularis	-=0.99	A=0.01	0.41154	8.21E-19
rs4921958	rs2632845	chr8:18660720	0.017311167	0.811821923	PSD3	ENSG00000156011.16	Brain - Spinal cord (cervical c-1)	C=0.59	G=0.41	-0.519542	3.39E-10
rs4921958	rs2632848	chr8:18659780	0.017059012	0.810866704	PSD3	ENSG00000156011.16	Brain - Spinal cord (cervical c-1)	T=0.587	C=0.413	-0.516994	5.95E-10
rs4921958	rs1386692	chr8:18658750	0.017059012	0.810866704	PSD3	ENSG00000156011.16	Brain - Spinal cord (cervical c-1)	A=0.587	G=0.413	-0.506856	1.08E-09
rs4921958	rs2638625	chr8:18659269	0.017396047	0.812138189	PSD3	ENSG00000156011.16	Brain - Spinal cord (cervical c-1)	C=0.591	T=0.409	-0.506856	1.08E-09
rs4921958	rs547180436	chr8:18871342	0.000182918	1	PSD3	ENSG00000156011.16	Esophagus - Gastroesophageal Junctio	-=0.99	A=0.01	0.267169	5.99E-09
rs4921958	rs7009615	chr8:18572835	0.002078093	1	PSD3	ENSG00000156011.16	Small Intestine - Terminal Ileum	T=0.898	C=0.102	0.37749	1.38E-07
rs4921958	rs138983771	chr8:18919610	0.000257118	1	PSD3	ENSG00000156011.16	Esophagus - Muscularis	G=0.986	A=0.014	0.754799	1.43E-07
rs4921958	rs60854172	chr8:18867056	0.002191104	1	PSD3	ENSG00000156011.16	Esophagus - Mucosa	-=0.893	A=0.107	0.215939	2.93E-06
rs4921958	rs111916415	chr8:18656221	0.017656987	0.898198745	PSD3	ENSG00000156011.16	Brain - Spinal cord (cervical c-1)	A=0.454	T=0.546	0.411994	2.93E-06
rs4921958	rs2632845	chr8:18660720	0.017311167	0.811821923	PSD3	ENSG00000156011.16	Brain - Substantia nigra	C=0.59	G=0.41	-0.241026	3.37E-06
rs4921958	rs17696125	chr8:18574874	0.000833284	1	PSD3	ENSG00000156011.16	Small Intestine - Terminal Ileum	T=0.956	C=0.044	0.410081	3.38E-06
rs4921958	rs7841096	chr8:18632333	0.029263074	1	PSD3	ENSG00000156011.16	Brain - Spinal cord (cervical c-1)	A=0.384	T=0.616	0.378279	3.44E-06
rs4921958	rs59321198	chr8:18866979	0.002168401	1	PSD3	ENSG00000156011.16	Esophagus - Mucosa	C=0.894	G=0.106	0.217409	3.55E-06
rs4921958	rs1386692	chr8:18658750	0.017059012	0.810866704	PSD3	ENSG00000156011.16	Brain - Substantia nigra	A=0.587	G=0.413	-0.23662	4.89E-06
rs4921958	rs2638625	chr8:18659269	0.017396047	0.812138189	PSD3	ENSG00000156011.16	Brain - Substantia nigra	C=0.591	T=0.409	-0.23662	4.89E-06
rs4921958	rs78656518	chr8:18844514	0.000109312	1	PSD3	ENSG00000156011.16	Esophagus - Muscularis	T=0.994	C=0.006	0.494999	4.95E-06
rs4921958	rs111699729	chr8:18865650	0.002168401	1	PSD3	ENSG00000156011.16	Esophagus - Mucosa	G=0.894	A=0.106	0.213434	5.55E-06
rs4921958	rs7010324	chr8:18866227	0.002100595	1	PSD3	ENSG00000156011.16	Esophagus - Mucosa	C=0.897	T=0.103	0.213434	5.55E-06
rs4921958	rs6996112	chr8:18866326	0.002191104	1	PSD3	ENSG00000156011.16	Esophagus - Mucosa	T=0.893	C=0.107	0.213434	5.55E-06
rs4921958	rs7010496	chr8:18866368	0.002168401	1	PSD3	ENSG00000156011.16	Esophagus - Mucosa	C=0.894	T=0.106	0.213434	5.55E-06
rs4921958	rs117966479	chr8:18893665	0.000993049	1	PSD3	ENSG00000156011.16	Small Intestine - Terminal Ileum	T=0.948	C=0.052	0.630419	5.82E-06
rs4921958	rs77386793	chr8:18869663	0.002168401	1	PSD3	ENSG00000156011.16	Esophagus - Mucosa	C=0.894	A=0.106	0.225491	6.32E-06
rs4921958	rs34426360	chr8:18575404	0.0008135	1	PSD3	ENSG00000156011.16	Small Intestine - Terminal Ileum	-=0.957	AGA=0.043	0.457749	7.58E-06

rs4921958	rs1872890	chr8:18575581	0.000793757	1	PSD3	ENSG00000156011.16	Small Intestine - Terminal Ileum	A=0.958	G=0.042	0.457749	7.58E-06
rs4921958	rs1038611	chr8:18624327	0.031994898	1	PSD3	ENSG00000156011.16	Brain - Spinal cord (cervical c-1)	C=0.363	T=0.637	0.378354	7.96E-06
rs4921958	rs1038610	chr8:18624424	0.031994898	1	PSD3	ENSG00000156011.16	Brain - Spinal cord (cervical c-1)	C=0.363	T=0.637	0.378354	7.96E-06
rs4921958	rs146517899	chr8:18867154	0.002191104	1	PSD3	ENSG00000156011.16	Esophagus - Mucosa	C=0.893	T=0.107	0.210362	8.66E-06
rs4921958	rs56990136	chr8:18872866	0.002213857	1	PSD3	ENSG00000156011.16	Esophagus - Mucosa	C=0.892	T=0.108	0.21699	9.52E-06
rs4921958	rs73594648	chr8:18876592	0.002213857	1	PSD3	ENSG00000156011.16	Esophagus - Mucosa	A=0.892	T=0.108	0.208395	1.24E-05
rs4921958	rs141114042	chr8:18631690	0.029886193	1	PSD3	ENSG00000156011.16	Brain - Spinal cord (cervical c-1)	TTAA=0.379	-=0.621	0.367347	1.35E-05
rs4921958	rs116324874	chr8:18876557	0.002213857	1	PSD3	ENSG00000156011.16	Esophagus - Mucosa	G=0.892	A=0.108	0.219157	1.52E-05
rs4921958	rs80027157	chr8:18866078	0.002213857	1	PSD3	ENSG00000156011.16	Esophagus - Mucosa	A=0.892	G=0.108	0.208302	1.57E-05
rs4921958	rs7009185	chr8:18866220	0.002168401	1	PSD3	ENSG00000156011.16	Esophagus - Mucosa	G=0.894	A=0.106	0.208302	1.57E-05
rs4921958	rs7010821	chr8:18866541	0.002168401	1	PSD3	ENSG00000156011.16	Esophagus - Mucosa	C=0.894	T=0.106	0.208302	1.57E-05
rs4921958	rs375703979	chr8:18866779	0.002168401	1	PSD3	ENSG00000156011.16	Esophagus - Mucosa	AG=0.894	-=0.106	0.208302	1.57E-05
rs4921958	rs35675493	chr8:18653677	0.017508346	1	PSD3	ENSG00000156011.16	Brain - Spinal cord (cervical c-1)	CCC=0.51	-=0.49	0.381151	1.59E-05
rs4921958	rs9644614	chr8:18630435	0.032692308	1	PSD3	ENSG00000156011.16	Brain - Spinal cord (cervical c-1)	C=0.358	T=0.642	0.374175	1.59E-05
rs4921958	rs9644615	chr8:18630532	0.03255128	1	PSD3	ENSG00000156011.16	Brain - Spinal cord (cervical c-1)	C=0.359	A=0.641	0.374175	1.59E-05
rs4921958	rs189718418	chr8:18857486	9.10E-05	1	PSD3	ENSG00000156011.16	Esophagus - Muscularis	A=0.995	G=0.005	0.496246	1.84E-05
rs4921958	rs2410598	chr8:18872462	0.002213857	1	PSD3	ENSG00000156011.16	Esophagus - Mucosa	C=0.892	T=0.108	0.204344	1.89E-05
rs4921958	rs57855654	chr8:18872943	0.002213857	1	PSD3	ENSG00000156011.16	Esophagus - Mucosa	G=0.892	A=0.108	0.204344	1.89E-05
rs4921958	rs335232	chr8:18636126	0.008114617	1	PSD3	ENSG00000156011.16	Brain - Cerebellum	G=0.692	A=0.308	0.211844	2.05E-05
rs4921958	rs10111009	chr8:18973729	0.004105293	1	PSD3	ENSG00000156011.16	Skin - Sun Exposed (Lower leg)	C=0.816	T=0.184	0.172705	2.10E-05
rs4921958	rs76653031	chr8:18574691	0.000331918	1	PSD3	ENSG00000156011.16	Small Intestine - Terminal Ileum	C=0.982	A=0.018	0.614718	2.15E-05
rs4921958	rs35592282	chr8:18576601	0.000331918	1	PSD3	ENSG00000156011.16	Small Intestine - Terminal Ileum	A=0.982	-=0.018	0.614718	2.15E-05
rs4921958	rs143185883	chr8:18577856	0.000331918	1	PSD3	ENSG00000156011.16	Small Intestine - Terminal Ileum	C=0.982	-=0.018	0.614718	2.15E-05
rs4921958	rs79697401	chr8:18578261	0.000331918	1	PSD3	ENSG00000156011.16	Small Intestine - Terminal Ileum	T=0.982	C=0.018	0.614718	2.15E-05
rs4921958	rs17127092	chr8:18579361	0.000331918	1	PSD3	ENSG00000156011.16	Small Intestine - Terminal Ileum	A=0.982	G=0.018	0.614718	2.15E-05
rs4921958	rs12056418	chr8:18580835	0.000331918	1	PSD3	ENSG00000156011.16	Small Intestine - Terminal Ileum	C=0.982	T=0.018	0.614718	2.15E-05
rs4921958	rs76973373	chr8:18583218	0.000331918	1	PSD3	ENSG00000156011.16	Small Intestine - Terminal Ileum	C=0.982	T=0.018	0.614718	2.15E-05
rs4921958	rs79550828	chr8:18587562	0.000331918	1	PSD3	ENSG00000156011.16	Small Intestine - Terminal Ileum	G=0.982	C=0.018	0.614718	2.15E-05
rs4921958	rs79052288	chr8:18589608	0.000331918	1	PSD3	ENSG00000156011.16	Small Intestine - Terminal Ileum	C=0.982	G=0.018	0.614718	2.15E-05
rs4921958	rs57825650	chr8:18591403	0.000331918	1	PSD3	ENSG00000156011.16	Small Intestine - Terminal Ileum	A=0.982	G=0.018	0.614718	2.15E-05
rs4921958	rs335234	chr8:18637444	0.008305161	1	PSD3	ENSG00000156011.16	Brain - Cerebellum	C=0.687	T=0.313	0.2134	2.22E-05
rs4921958	rs60549282	chr8:18877472	0.002100595	1	PSD3	ENSG00000156011.16	Esophagus - Mucosa	C=0.897	T=0.103	0.198321	3.37E-05
rs4921958	rs4463468	chr8:18879717	0.002100595	1	PSD3	ENSG00000156011.16	Esophagus - Mucosa	C=0.897	T=0.103	0.198321	3.37E-05
rs4921958	rs75123216	chr8:18821251	0.000331918	1	PSD3	ENSG00000156011.16	Esophagus - Muscularis	T=0.982	C=0.018	0.413555	3.38E-05
rs4921958	rs116971711	chr8:18822209	0.000331918	1	PSD3	ENSG00000156011.16	Esophagus - Muscularis	G=0.982	A=0.018	0.413555	3.38E-05
rs4921958	rs335248	chr8:18646733	0.008152506	1	PSD3	ENSG00000156011.16	Brain - Cerebellum	G=0.691	C=0.309	0.203295	3.48E-05
rs4921958	rs77537870	chr8:18877244	0.002213857	1	PSD3	ENSG00000156011.16	Esophagus - Mucosa	A=0.892	G=0.108	0.202602	3.49E-05
rs4921958	rs335237	chr8:18641291	0.009593114	1	PSD3	ENSG00000156011.16	Brain - Cerebellum	T=0.655	C=0.345	0.200677	3.52E-05
rs4921958	rs61050926	chr8:18864611	0.002191104	1	PSD3	ENSG00000156011.16	Esophagus - Mucosa	G=0.893	A=0.107	0.185851	4.35E-05
rs4921958	rs57965775	chr8:18865248	0.002191104	1	PSD3	ENSG00000156011.16	Esophagus - Mucosa	C=0.893	G=0.107	0.185851	4.35E-05
rs4921958	rs73199994	chr8:18646365	0.001595361	1	PSD3	ENSG00000156011.16	Brain - Cerebellum	C=0.919	T=0.081	0.375659	4.63E-05
rs4921958	rs76595146	chr8:18873146	0.002213857	1	PSD3	ENSG00000156011.16	Esophagus - Mucosa	G=0.892	T=0.108	0.198351	5.28E-05
rs4921958	rs79513952	chr8:18875713	0.002213857	1	PSD3	ENSG00000156011.16	Esophagus - Mucosa	G=0.892	A=0.108	0.198351	5.28E-05
rs4921958	rs115432345	chr8:18871863	0.002236661	1	PSD3	ENSG00000156011.16	Esophagus - Mucosa	G=0.891	T=0.109	0.194814	6.45E-05
rs4921958	rs73594633	chr8:18871992	0.002213857	1	PSD3	ENSG00000156011.16	Esophagus - Mucosa	C=0.892	G=0.108	0.189659	6.52E-05
rs4921958	rs145752806	chr8:18882886	0.002100595	1	PSD3	ENSG00000156011.16	Esophagus - Mucosa	ACTC=0.897	-=0.103	0.194717	7.13E-05
rs4921958	rs78119471	chr8:18883327	0.002100595	1	PSD3	ENSG00000156011.16	Esophagus - Mucosa	A=0.897	G=0.103	0.194717	7.13E-05
rs4921958	rs60074764	chr8:18863855	0.002213857	1	PSD3	ENSG00000156011.16	Esophagus - Mucosa	T=0.892	C=0.108	0.173434	8.93E-05
rs4921958	rs60016927	chr8:18863989	0.002213857	1	PSD3	ENSG00000156011.16	Esophagus - Mucosa	T=0.892	C=0.108	0.173434	8.93E-05
rs4921958	rs79233079	chr8:19029881	0.002259516	1	PSD3	ENSG00000156011.16	Cells - Cultured fibroblasts	C=0.89	T=0.11	-0.168053	0.000116145
rs4921959	rs547180436	chr8:18871342	0.000319226	1	PSD3	ENSG00000156011.16	Esophagus - Muscularis	-=0.99	A=0.01	0.41154	8.21E-19

rs4921959	rs547180436	chr8:18871342	0.000319226	1	PSD3	ENSG00000156011.16	Esophagus - Gastroesophageal Junctio	-=0.99	A=0.01	0.267169	5.99E-09
rs4921959	rs138983771	chr8:18919610	0.000448718	1	PSD3	ENSG00000156011.16	Esophagus - Muscularis	G=0.986	A=0.014	0.754799	1.43E-07
rs4921959	rs111916415	chr8:18656221	0.025850161	0.822668782	PSD3	ENSG00000156011.16	Brain - Spinal cord (cervical c-1)	A=0.454	T=0.546	0.411994	2.93E-06
rs4921959	rs78656518	chr8:18844514	0.000190769	1	PSD3	ENSG00000156011.16	Esophagus - Muscularis	T=0.994	C=0.006	0.494999	4.95E-06
rs4921959	rs117966479	chr8:18893665	0.001733054	1	PSD3	ENSG00000156011.16	Small Intestine - Terminal Ileum	T=0.948	C=0.052	0.630419	5.82E-06
rs4921959	rs141114042	chr8:18631690	0.052156942	1	PSD3	ENSG00000156011.16	Brain - Spinal cord (cervical c-1)	TTAA=0.379	-=0.621	0.367347	1.35E-05
rs4921959	rs35675493	chr8:18653677	0.026665107	0.934175227	PSD3	ENSG00000156011.16	Brain - Spinal cord (cervical c-1)	CCC=0.51	-=0.49	0.381151	1.59E-05
rs4921959	rs189718418	chr8:18857486	0.000158816	1	PSD3	ENSG00000156011.16	Esophagus - Muscularis	A=0.995	G=0.005	0.496246	1.84E-05
rs4921959	rs335232	chr8:18636126	0.014161509	1	PSD3	ENSG00000156011.16	Brain - Cerebellum	G=0.692	A=0.308	0.211844	2.05E-05
rs4921959	rs10111009	chr8:18973729	0.007164496	1	PSD3	ENSG00000156011.16	Skin - Sun Exposed (Lower leg)	C=0.816	T=0.184	0.172705	2.10E-05
rs4921959	rs335234	chr8:18637444	0.014494044	1	PSD3	ENSG00000156011.16	Brain - Cerebellum	C=0.687	T=0.313	0.2134	2.22E-05
rs4921959	rs75123216	chr8:18821251	0.000579259	1	PSD3	ENSG00000156011.16	Esophagus - Muscularis	T=0.982	C=0.018	0.413555	3.38E-05
rs4921959	rs116971711	chr8:18822209	0.000579259	1	PSD3	ENSG00000156011.16	Esophagus - Muscularis	G=0.982	A=0.018	0.413555	3.38E-05
rs4921959	rs335248	chr8:18646733	0.014227633	1	PSD3	ENSG00000156011.16	Brain - Cerebellum	G=0.691	C=0.309	0.203295	3.48E-05
rs4921959	rs335237	chr8:18641291	0.016741761	1	PSD3	ENSG00000156011.16	Brain - Cerebellum	T=0.655	C=0.345	0.200677	3.52E-05
rs4921959	rs73199994	chr8:18646365	0.0027842	1	PSD3	ENSG00000156011.16	Brain - Cerebellum	C=0.919	T=0.081	0.375659	4.63E-05
rs4921959	rs6983992	chr8:18728635	0.004025557	0.803323558	PSD3	ENSG00000156011.16	Heart - Atrial Appendage	T=0.836	C=0.164	0.234918	5.40E-05
rs4921961	rs547180436	chr8:18871342	0.000224475	1	PSD3	ENSG00000156011.16	Esophagus - Muscularis	-=0.99	A=0.01	0.41154	8.21E-19
rs4921961	rs547180436	chr8:18871342	0.000224475	1	PSD3	ENSG00000156011.16	Esophagus - Gastroesophageal Junctio	-=0.99	A=0.01	0.267169	5.99E-09
rs4921961	rs138983771	chr8:18919610	0.000315532	1	PSD3	ENSG00000156011.16	Esophagus - Muscularis	G=0.986	A=0.014	0.754799	1.43E-07
rs4921961	rs111916415	chr8:18656221	0.018655527	0.833416128	PSD3	ENSG00000156011.16	Brain - Spinal cord (cervical c-1)	A=0.454	T=0.546	0.411994	2.93E-06
rs4921961	rs78656518	chr8:18844514	0.000134146	1	PSD3	ENSG00000156011.16	Esophagus - Muscularis	T=0.994	C=0.006	0.494999	4.95E-06
rs4921961	rs117966479	chr8:18893665	0.00121866	1	PSD3	ENSG00000156011.16	Small Intestine - Terminal Ileum	T=0.948	C=0.052	0.630419	5.82E-06
rs4921961	rs141114042	chr8:18631690	0.031505672	0.926836364	PSD3	ENSG00000156011.16	Brain - Spinal cord (cervical c-1)	TTAA=0.379	-=0.621	0.367347	1.35E-05
rs4921961	rs35675493	chr8:18653677	0.014253868	0.814493823	PSD3	ENSG00000156011.16	Brain - Spinal cord (cervical c-1)	CCC=0.51	-=0.49	0.381151	1.59E-05
rs4921961	rs189718418	chr8:18857486	0.000111677	1	PSD3	ENSG00000156011.16	Esophagus - Muscularis	A=0.995	G=0.005	0.496246	1.84E-05
rs4921961	rs335232	chr8:18636126	0.009958181	1	PSD3	ENSG00000156011.16	Brain - Cerebellum	G=0.692	A=0.308	0.211844	2.05E-05
rs4921961	rs10111009	chr8:18973729	0.005037977	1	PSD3	ENSG00000156011.16	Skin - Sun Exposed (Lower leg)	C=0.816	T=0.184	0.172705	2.10E-05
rs4921961	rs335234	chr8:18637444	0.010192016	1	PSD3	ENSG00000156011.16	Brain - Cerebellum	C=0.687	T=0.313	0.2134	2.22E-05
rs4921961	rs75123216	chr8:18821251	0.000407327	1	PSD3	ENSG00000156011.16	Esophagus - Muscularis	T=0.982	C=0.018	0.413555	3.38E-05
rs4921961	rs116971711	chr8:18822209	0.000407327	1	PSD3	ENSG00000156011.16	Esophagus - Muscularis	G=0.982	A=0.018	0.413555	3.38E-05
rs4921961	rs335248	chr8:18646733	0.010004679	1	PSD3	ENSG00000156011.16	Brain - Cerebellum	G=0.691	C=0.309	0.203295	3.48E-05
rs4921961	rs335237	chr8:18641291	0.01177258	1	PSD3	ENSG00000156011.16	Brain - Cerebellum	T=0.655	C=0.345	0.200677	3.52E-05
rs4921961	rs73199994	chr8:18646365	0.001957811	1	PSD3	ENSG00000156011.16	Brain - Cerebellum	C=0.919	T=0.081	0.375659	4.63E-05
rs56062960	rs547180436	chr8:18871342	0.000182918	1	PSD3	ENSG00000156011.16	Esophagus - Muscularis	-=0.99	A=0.01	0.41154	8.21E-19
rs56062960	rs2632845	chr8:18660720	0.017311167	0.811821923	PSD3	ENSG00000156011.16	Brain - Spinal cord (cervical c-1)	C=0.59	G=0.41	-0.519542	3.39E-10
rs56062960	rs2632848	chr8:18659780	0.017059012	0.810866704	PSD3	ENSG00000156011.16	Brain - Spinal cord (cervical c-1)	T=0.587	C=0.413	-0.516994	5.95E-10
rs56062960	rs1386692	chr8:18658750	0.017059012	0.810866704	PSD3	ENSG00000156011.16	Brain - Spinal cord (cervical c-1)	A=0.587	G=0.413	-0.506856	1.08E-09
rs56062960	rs2638625	chr8:18659269	0.017396047	0.812138189	PSD3	ENSG00000156011.16	Brain - Spinal cord (cervical c-1)	C=0.591	T=0.409	-0.506856	1.08E-09
rs56062960	rs547180436	chr8:18871342	0.000182918	1	PSD3	ENSG00000156011.16	Esophagus - Gastroesophageal Junctio	-=0.99	A=0.01	0.267169	5.99E-09
rs56062960	rs7009615	chr8:18572835	0.002078093	1	PSD3	ENSG00000156011.16	Small Intestine - Terminal Ileum	T=0.898	C=0.102	0.37749	1.38E-07
rs56062960	rs138983771	chr8:18919610	0.000257118	1	PSD3	ENSG00000156011.16	Esophagus - Muscularis	G=0.986	A=0.014	0.754799	1.43E-07
rs56062960	rs60854172	chr8:18867056	0.002191104	1	PSD3	ENSG00000156011.16	Esophagus - Mucosa	-=0.893	A=0.107	0.215939	2.93E-06
rs56062960	rs111916415	chr8:18656221	0.017656987	0.898198745	PSD3	ENSG00000156011.16	Brain - Spinal cord (cervical c-1)	A=0.454	T=0.546	0.411994	2.93E-06
rs56062960	rs2632845	chr8:18660720	0.017311167	0.811821923	PSD3	ENSG00000156011.16	Brain - Substantia nigra	C=0.59	G=0.41	-0.241026	3.37E-06
rs56062960	rs17696125	chr8:18574874	0.000833284	1	PSD3	ENSG00000156011.16	Small Intestine - Terminal Ileum	T=0.956	C=0.044	0.410081	3.38E-06
rs56062960	rs7841096	chr8:18632333	0.029263074	1	PSD3	ENSG00000156011.16	Brain - Spinal cord (cervical c-1)	A=0.384	T=0.616	0.378279	3.44E-06
rs56062960	rs59321198	chr8:18866979	0.002168401	1	PSD3	ENSG00000156011.16	Esophagus - Mucosa	C=0.894	G=0.106	0.217409	3.55E-06
rs56062960	rs1386692	chr8:18658750	0.017059012	0.810866704	PSD3	ENSG00000156011.16	Brain - Substantia nigra	A=0.587	G=0.413	-0.23662	4.89E-06
rs56062960	rs2638625	chr8:18659269	0.017396047	0.812138189	PSD3	ENSG00000156011.16	Brain - Substantia nigra	C=0.591	T=0.409	-0.23662	4.89E-06
rs56062960	rs78656518	chr8:18844514	0.000109312	1	PSD3	ENSG00000156011.16	Esophagus - Muscularis	T=0.994	C=0.006	0.494999	4.95E-06

rs56062960	rs111699729	chr8:18865650	0.002168401	1	PSD3	ENSG00000156011.16	Esophagus - Mucosa	G=0.894	A=0.106	0.213434	5.55E-06
rs56062960	rs7010324	chr8:18866227	0.002100595	1	PSD3	ENSG00000156011.16	Esophagus - Mucosa	C=0.897	T=0.103	0.213434	5.55E-06
rs56062960	rs6996112	chr8:18866326	0.002191104	1	PSD3	ENSG00000156011.16	Esophagus - Mucosa	T=0.893	C=0.107	0.213434	5.55E-06
rs56062960	rs7010496	chr8:18866368	0.002168401	1	PSD3	ENSG00000156011.16	Esophagus - Mucosa	C=0.894	T=0.106	0.213434	5.55E-06
rs56062960	rs117966479	chr8:18893665	0.000993049	1	PSD3	ENSG00000156011.16	Small Intestine - Terminal Ileum	T=0.948	C=0.052	0.630419	5.82E-06
rs56062960	rs77386793	chr8:18869663	0.002168401	1	PSD3	ENSG00000156011.16	Esophagus - Mucosa	C=0.894	A=0.106	0.225491	6.32E-06
rs56062960	rs34426360	chr8:18575404	0.0008135	1	PSD3	ENSG00000156011.16	Small Intestine - Terminal Ileum	-=0.957	AGA=0.043	0.457749	7.58E-06
rs56062960	rs1872890	chr8:18575581	0.000793757	1	PSD3	ENSG00000156011.16	Small Intestine - Terminal Ileum	A=0.958	G=0.042	0.457749	7.58E-06
rs56062960	rs1038611	chr8:18624327	0.031994898	1	PSD3	ENSG00000156011.16	Brain - Spinal cord (cervical c-1)	C=0.363	T=0.637	0.378354	7.96E-06
rs56062960	rs1038610	chr8:18624424	0.031994898	1	PSD3	ENSG00000156011.16	Brain - Spinal cord (cervical c-1)	C=0.363	T=0.637	0.378354	7.96E-06
rs56062960	rs146517899	chr8:18867154	0.002191104	1	PSD3	ENSG00000156011.16	Esophagus - Mucosa	C=0.893	T=0.107	0.210362	8.66E-06
rs56062960	rs56990136	chr8:18872866	0.002213857	1	PSD3	ENSG00000156011.16	Esophagus - Mucosa	C=0.892	T=0.108	0.21699	9.52E-06
rs56062960	rs73594648	chr8:18876592	0.002213857	1	PSD3	ENSG00000156011.16	Esophagus - Mucosa	A=0.892	T=0.108	0.208395	1.24E-05
rs56062960	rs141114042	chr8:18631690	0.029886193	1	PSD3	ENSG00000156011.16	Brain - Spinal cord (cervical c-1)	TTAA=0.379	-=0.621	0.367347	1.35E-05
rs56062960	rs116324874	chr8:18876557	0.002213857	1	PSD3	ENSG00000156011.16	Esophagus - Mucosa	G=0.892	A=0.108	0.219157	1.52E-05
rs56062960	rs80027157	chr8:18866078	0.002213857	1	PSD3	ENSG00000156011.16	Esophagus - Mucosa	A=0.892	G=0.108	0.208302	1.57E-05
rs56062960	rs7009185	chr8:18866220	0.002168401	1	PSD3	ENSG00000156011.16	Esophagus - Mucosa	G=0.894	A=0.106	0.208302	1.57E-05
rs56062960	rs7010821	chr8:18866541	0.002168401	1	PSD3	ENSG00000156011.16	Esophagus - Mucosa	C=0.894	T=0.106	0.208302	1.57E-05
rs56062960	rs375703979	chr8:18866779	0.002168401	1	PSD3	ENSG00000156011.16	Esophagus - Mucosa	AG=0.894	-=0.106	0.208302	1.57E-05
rs56062960	rs35675493	chr8:18653677	0.017508346	1	PSD3	ENSG00000156011.16	Brain - Spinal cord (cervical c-1)	CCC=0.51	-=0.49	0.381151	1.59E-05
rs56062960	rs9644614	chr8:18630435	0.032692308	1	PSD3	ENSG00000156011.16	Brain - Spinal cord (cervical c-1)	C=0.358	T=0.642	0.374175	1.59E-05
rs56062960	rs9644615	chr8:18630532	0.03255128	1	PSD3	ENSG00000156011.16	Brain - Spinal cord (cervical c-1)	C=0.359	A=0.641	0.374175	1.59E-05
rs56062960	rs189718418	chr8:18857486	9.10E-05	1	PSD3	ENSG00000156011.16	Esophagus - Muscularis	A=0.995	G=0.005	0.496246	1.84E-05
rs56062960	rs2410598	chr8:18872462	0.002213857	1	PSD3	ENSG00000156011.16	Esophagus - Mucosa	C=0.892	T=0.108	0.204344	1.89E-05
rs56062960	rs57855654	chr8:18872943	0.002213857	1	PSD3	ENSG00000156011.16	Esophagus - Mucosa	G=0.892	A=0.108	0.204344	1.89E-05
rs56062960	rs335232	chr8:18636126	0.008114617	1	PSD3	ENSG00000156011.16	Brain - Cerebellum	G=0.692	A=0.308	0.211844	2.05E-05
rs56062960	rs10111009	chr8:18973729	0.004105293	1	PSD3	ENSG00000156011.16	Skin - Sun Exposed (Lower leg)	C=0.816	T=0.184	0.172705	2.10E-05
rs56062960	rs76653031	chr8:18574691	0.000331918	1	PSD3	ENSG00000156011.16	Small Intestine - Terminal Ileum	C=0.982	A=0.018	0.614718	2.15E-05
rs56062960	rs35592282	chr8:18576601	0.000331918	1	PSD3	ENSG00000156011.16	Small Intestine - Terminal Ileum	A=0.982	-=0.018	0.614718	2.15E-05
rs56062960	rs143185883	chr8:18577856	0.000331918	1	PSD3	ENSG00000156011.16	Small Intestine - Terminal Ileum	C=0.982	-=0.018	0.614718	2.15E-05
rs56062960	rs79697401	chr8:18578261	0.000331918	1	PSD3	ENSG00000156011.16	Small Intestine - Terminal Ileum	T=0.982	C=0.018	0.614718	2.15E-05
rs56062960	rs17127092	chr8:18579361	0.000331918	1	PSD3	ENSG00000156011.16	Small Intestine - Terminal Ileum	A=0.982	G=0.018	0.614718	2.15E-05
rs56062960	rs12056418	chr8:18580835	0.000331918	1	PSD3	ENSG00000156011.16	Small Intestine - Terminal Ileum	C=0.982	T=0.018	0.614718	2.15E-05
rs56062960	rs76973373	chr8:18583218	0.000331918	1	PSD3	ENSG00000156011.16	Small Intestine - Terminal Ileum	C=0.982	T=0.018	0.614718	2.15E-05
rs56062960	rs79550828	chr8:18587562	0.000331918	1	PSD3	ENSG00000156011.16	Small Intestine - Terminal Ileum	G=0.982	C=0.018	0.614718	2.15E-05
rs56062960	rs79052288	chr8:18589608	0.000331918	1	PSD3	ENSG00000156011.16	Small Intestine - Terminal Ileum	C=0.982	G=0.018	0.614718	2.15E-05
rs56062960	rs57825650	chr8:18591403	0.000331918	1	PSD3	ENSG00000156011.16	Small Intestine - Terminal Ileum	A=0.982	G=0.018	0.614718	2.15E-05
rs56062960	rs335234	chr8:18637444	0.008305161	1	PSD3	ENSG00000156011.16	Brain - Cerebellum	C=0.687	T=0.313	0.2134	2.22E-05
rs56062960	rs60549282	chr8:18877472	0.002100595	1	PSD3	ENSG00000156011.16	Esophagus - Mucosa	C=0.897	T=0.103	0.198321	3.37E-05
rs56062960	rs4463468	chr8:18879717	0.002100595	1	PSD3	ENSG00000156011.16	Esophagus - Mucosa	C=0.897	T=0.103	0.198321	3.37E-05
rs56062960	rs75123216	chr8:18821251	0.000331918	1	PSD3	ENSG00000156011.16	Esophagus - Muscularis	T=0.982	C=0.018	0.413555	3.38E-05
rs56062960	rs116971711	chr8:18822209	0.000331918	1	PSD3	ENSG00000156011.16	Esophagus - Muscularis	G=0.982	A=0.018	0.413555	3.38E-05
rs56062960	rs335248	chr8:18646733	0.008152506	1	PSD3	ENSG00000156011.16	Brain - Cerebellum	G=0.691	C=0.309	0.203295	3.48E-05
rs56062960	rs77537870	chr8:18877244	0.002213857	1	PSD3	ENSG00000156011.16	Esophagus - Mucosa	A=0.892	G=0.108	0.202602	3.49E-05
rs56062960	rs335237	chr8:18641291	0.009593114	1	PSD3	ENSG00000156011.16	Brain - Cerebellum	T=0.655	C=0.345	0.200677	3.52E-05
rs56062960	rs61050926	chr8:18864611	0.002191104	1	PSD3	ENSG00000156011.16	Esophagus - Mucosa	G=0.893	A=0.107	0.185851	4.35E-05
rs56062960	rs57965775	chr8:18865248	0.002191104	1	PSD3	ENSG00000156011.16	Esophagus - Mucosa	C=0.893	G=0.107	0.185851	4.35E-05
rs56062960	rs73199994	chr8:18646365	0.001595361	1	PSD3	ENSG00000156011.16	Brain - Cerebellum	C=0.919	T=0.081	0.375659	4.63E-05
rs56062960	rs76595146	chr8:18873146	0.002213857	1	PSD3	ENSG00000156011.16	Esophagus - Mucosa	G=0.892	T=0.108	0.198351	5.28E-05
rs56062960	rs79513952	chr8:18875713	0.002213857	1	PSD3	ENSG00000156011.16	Esophagus - Mucosa	G=0.892	A=0.108	0.198351	5.28E-05
rs56062960	rs115432345	chr8:18871863	0.002236661	1	PSD3	ENSG00000156011.16	Esophagus - Mucosa	G=0.891	T=0.109	0.194814	6.45E-05

rs56062960	rs73594633	chr8:18871992	0.002213857	1	PSD3	ENSG00000156011.16	Esophagus - Mucosa	C=0.892	G=0.108	0.189659	6.52E-05
rs56062960	rs145752806	chr8:18882886	0.002100595	1	PSD3	ENSG00000156011.16	Esophagus - Mucosa	ACTC=0.897	-=0.103	0.194717	7.13E-05
rs56062960	rs78119471	chr8:18883327	0.002100595	1	PSD3	ENSG00000156011.16	Esophagus - Mucosa	A=0.897	G=0.103	0.194717	7.13E-05
rs56062960	rs60074764	chr8:18863855	0.002213857	1	PSD3	ENSG00000156011.16	Esophagus - Mucosa	T=0.892	C=0.108	0.173434	8.93E-05
rs56062960	rs60016927	chr8:18863989	0.002213857	1	PSD3	ENSG00000156011.16	Esophagus - Mucosa	T=0.892	C=0.108	0.173434	8.93E-05
rs56062960	rs79233079	chr8:19029881	0.002259516	1	PSD3	ENSG00000156011.16	Cells - Cultured fibroblasts	C=0.89	T=0.11	-0.168053	0.000116145
rs57557661	rs547180436	chr8:18871342	0.000182918	1	PSD3	ENSG00000156011.16	Esophagus - Muscularis	-=0.99	A=0.01	0.41154	8.21E-19
rs57557661	rs2632845	chr8:18660720	0.017311167	0.811821923	PSD3	ENSG00000156011.16	Brain - Spinal cord (cervical c-1)	C=0.59	G=0.41	-0.519542	3.39E-10
rs57557661	rs2632848	chr8:18659780	0.017059012	0.810866704	PSD3	ENSG00000156011.16	Brain - Spinal cord (cervical c-1)	T=0.587	C=0.413	-0.516994	5.95E-10
rs57557661	rs1386692	chr8:18658750	0.017059012	0.810866704	PSD3	ENSG00000156011.16	Brain - Spinal cord (cervical c-1)	A=0.587	G=0.413	-0.506856	1.08E-09
rs57557661	rs2638625	chr8:18659269	0.017396047	0.812138189	PSD3	ENSG00000156011.16	Brain - Spinal cord (cervical c-1)	C=0.591	T=0.409	-0.506856	1.08E-09
rs57557661	rs547180436	chr8:18871342	0.000182918	1	PSD3	ENSG00000156011.16	Esophagus - Gastroesophageal Junctio	-=0.99	A=0.01	0.267169	5.99E-09
rs57557661	rs7009615	chr8:18572835	0.002078093	1	PSD3	ENSG00000156011.16	Small Intestine - Terminal Ileum	T=0.898	C=0.102	0.37749	1.38E-07
rs57557661	rs138983771	chr8:18919610	0.000257118	1	PSD3	ENSG00000156011.16	Esophagus - Muscularis	G=0.986	A=0.014	0.754799	1.43E-07
rs57557661	rs60854172	chr8:18867056	0.002191104	1	PSD3	ENSG00000156011.16	Esophagus - Mucosa	-=0.893	A=0.107	0.215939	2.93E-06
rs57557661	rs111916415	chr8:18656221	0.017656987	0.898198745	PSD3	ENSG00000156011.16	Brain - Spinal cord (cervical c-1)	A=0.454	T=0.546	0.411994	2.93E-06
rs57557661	rs2632845	chr8:18660720	0.017311167	0.811821923	PSD3	ENSG00000156011.16	Brain - Substantia nigra	C=0.59	G=0.41	-0.241026	3.37E-06
rs57557661	rs17696125	chr8:18574874	0.000833284	1	PSD3	ENSG00000156011.16	Small Intestine - Terminal Ileum	T=0.956	C=0.044	0.410081	3.38E-06
rs57557661	rs7841096	chr8:18632333	0.029263074	1	PSD3	ENSG00000156011.16	Brain - Spinal cord (cervical c-1)	A=0.384	T=0.616	0.378279	3.44E-06
rs57557661	rs59321198	chr8:18866979	0.002168401	1	PSD3	ENSG00000156011.16	Esophagus - Mucosa	C=0.894	G=0.106	0.217409	3.55E-06
rs57557661	rs1386692	chr8:18658750	0.017059012	0.810866704	PSD3	ENSG00000156011.16	Brain - Substantia nigra	A=0.587	G=0.413	-0.23662	4.89E-06
rs57557661	rs2638625	chr8:18659269	0.017396047	0.812138189	PSD3	ENSG00000156011.16	Brain - Substantia nigra	C=0.591	T=0.409	-0.23662	4.89E-06
rs57557661	rs78656518	chr8:18844514	0.000109312	1	PSD3	ENSG00000156011.16	Esophagus - Muscularis	T=0.994	C=0.006	0.494999	4.95E-06
rs57557661	rs111699729	chr8:18865650	0.002168401	1	PSD3	ENSG00000156011.16	Esophagus - Mucosa	G=0.894	A=0.106	0.213434	5.55E-06
rs57557661	rs7010324	chr8:18866227	0.002100595	1	PSD3	ENSG00000156011.16	Esophagus - Mucosa	C=0.897	T=0.103	0.213434	5.55E-06
rs57557661	rs6996112	chr8:18866326	0.002191104	1	PSD3	ENSG00000156011.16	Esophagus - Mucosa	T=0.893	C=0.107	0.213434	5.55E-06
rs57557661	rs7010496	chr8:18866368	0.002168401	1	PSD3	ENSG00000156011.16	Esophagus - Mucosa	C=0.894	T=0.106	0.213434	5.55E-06
rs57557661	rs117966479	chr8:18893665	0.000993049	1	PSD3	ENSG00000156011.16	Small Intestine - Terminal Ileum	T=0.948	C=0.052	0.630419	5.82E-06
rs57557661	rs77386793	chr8:18869663	0.002168401	1	PSD3	ENSG00000156011.16	Esophagus - Mucosa	C=0.894	A=0.106	0.225491	6.32E-06
rs57557661	rs34426360	chr8:18575404	0.0008135	1	PSD3	ENSG00000156011.16	Small Intestine - Terminal Ileum	-=0.957	AGA=0.043	0.457749	7.58E-06
rs57557661	rs1872890	chr8:18575581	0.000793757	1	PSD3	ENSG00000156011.16	Small Intestine - Terminal Ileum	A=0.958	G=0.042	0.457749	7.58E-06
rs57557661	rs1038611	chr8:18624327	0.031994898	1	PSD3	ENSG00000156011.16	Brain - Spinal cord (cervical c-1)	C=0.363	T=0.637	0.378354	7.96E-06
rs57557661	rs1038610	chr8:18624424	0.031994898	1	PSD3	ENSG00000156011.16	Brain - Spinal cord (cervical c-1)	C=0.363	T=0.637	0.378354	7.96E-06
rs57557661	rs146517899	chr8:18867154	0.002191104	1	PSD3	ENSG00000156011.16	Esophagus - Mucosa	C=0.893	T=0.107	0.210362	8.66E-06
rs57557661	rs56990136	chr8:18872866	0.002213857	1	PSD3	ENSG00000156011.16	Esophagus - Mucosa	C=0.892	T=0.108	0.21699	9.52E-06
rs57557661	rs73594648	chr8:18876592	0.002213857	1	PSD3	ENSG00000156011.16	Esophagus - Mucosa	A=0.892	T=0.108	0.208395	1.24E-05
rs57557661	rs141114042	chr8:18631690	0.029886193	1	PSD3	ENSG00000156011.16	Brain - Spinal cord (cervical c-1)	TTAA=0.379	-=0.621	0.367347	1.35E-05
rs57557661	rs116324874	chr8:18876557	0.002213857	1	PSD3	ENSG00000156011.16	Esophagus - Mucosa	G=0.892	A=0.108	0.219157	1.52E-05
rs57557661	rs80027157	chr8:18866078	0.002213857	1	PSD3	ENSG00000156011.16	Esophagus - Mucosa	A=0.892	G=0.108	0.208302	1.57E-05
rs57557661	rs7009185	chr8:18866220	0.002168401	1	PSD3	ENSG00000156011.16	Esophagus - Mucosa	G=0.894	A=0.106	0.208302	1.57E-05
rs57557661	rs7010821	chr8:18866541	0.002168401	1	PSD3	ENSG00000156011.16	Esophagus - Mucosa	C=0.894	T=0.106	0.208302	1.57E-05
rs57557661	rs375703979	chr8:18866779	0.002168401	1	PSD3	ENSG00000156011.16	Esophagus - Mucosa	AG=0.894	-=0.106	0.208302	1.57E-05
rs57557661	rs35675493	chr8:18653677	0.017508346	1	PSD3	ENSG00000156011.16	Brain - Spinal cord (cervical c-1)	CCC=0.51	-=0.49	0.381151	1.59E-05
rs57557661	rs9644614	chr8:18630435	0.032692308	1	PSD3	ENSG00000156011.16	Brain - Spinal cord (cervical c-1)	C=0.358	T=0.642	0.374175	1.59E-05
rs57557661	rs9644615	chr8:18630532	0.03255128	1	PSD3	ENSG00000156011.16	Brain - Spinal cord (cervical c-1)	C=0.359	A=0.641	0.374175	1.59E-05
rs57557661	rs189718418	chr8:18857486	9.10E-05	1	PSD3	ENSG00000156011.16	Esophagus - Muscularis	A=0.995	G=0.005	0.496246	1.84E-05
rs57557661	rs2410598	chr8:18872462	0.002213857	1	PSD3	ENSG00000156011.16	Esophagus - Mucosa	C=0.892	T=0.108	0.204344	1.89E-05
rs57557661	rs57855654	chr8:18872943	0.002213857	1	PSD3	ENSG00000156011.16	Esophagus - Mucosa	G=0.892	A=0.108	0.204344	1.89E-05
rs57557661	rs335232	chr8:18636126	0.008114617	1	PSD3	ENSG00000156011.16	Brain - Cerebellum	G=0.692	A=0.308	0.211844	2.05E-05
rs57557661	rs10111009	chr8:18973729	0.004105293	1	PSD3	ENSG00000156011.16	Skin - Sun Exposed (Lower leg)	C=0.816	T=0.184	0.172705	2.10E-05
rs57557661	rs76653031	chr8:18574691	0.000331918	1	PSD3	ENSG00000156011.16	Small Intestine - Terminal Ileum	C=0.982	A=0.018	0.614718	2.15E-05

rs57557661	rs35592282	chr8:18576601	0.000331918	1	PSD3	ENSG00000156011.16	Small Intestine - Terminal Ileum	A=0.982	-=0.018	0.614718	2.15E-05
rs57557661	rs143185883	chr8:18577856	0.000331918	1	PSD3	ENSG00000156011.16	Small Intestine - Terminal Ileum	C=0.982	-=0.018	0.614718	2.15E-05
rs57557661	rs79697401	chr8:18578261	0.000331918	1	PSD3	ENSG00000156011.16	Small Intestine - Terminal Ileum	T=0.982	C=0.018	0.614718	2.15E-05
rs57557661	rs17127092	chr8:18579361	0.000331918	1	PSD3	ENSG00000156011.16	Small Intestine - Terminal Ileum	A=0.982	G=0.018	0.614718	2.15E-05
rs57557661	rs12056418	chr8:18580835	0.000331918	1	PSD3	ENSG00000156011.16	Small Intestine - Terminal Ileum	C=0.982	T=0.018	0.614718	2.15E-05
rs57557661	rs76973373	chr8:18583218	0.000331918	1	PSD3	ENSG00000156011.16	Small Intestine - Terminal Ileum	C=0.982	T=0.018	0.614718	2.15E-05
rs57557661	rs79550828	chr8:18587562	0.000331918	1	PSD3	ENSG00000156011.16	Small Intestine - Terminal Ileum	G=0.982	C=0.018	0.614718	2.15E-05
rs57557661	rs79052288	chr8:18589608	0.000331918	1	PSD3	ENSG00000156011.16	Small Intestine - Terminal Ileum	C=0.982	G=0.018	0.614718	2.15E-05
rs57557661	rs57825650	chr8:18591403	0.000331918	1	PSD3	ENSG00000156011.16	Small Intestine - Terminal Ileum	A=0.982	G=0.018	0.614718	2.15E-05
rs57557661	rs335234	chr8:18637444	0.008305161	1	PSD3	ENSG00000156011.16	Brain - Cerebellum	C=0.687	T=0.313	0.2134	2.22E-05
rs57557661	rs60549282	chr8:18877472	0.002100595	1	PSD3	ENSG00000156011.16	Esophagus - Mucosa	C=0.897	T=0.103	0.198321	3.37E-05
rs57557661	rs4463468	chr8:18879717	0.002100595	1	PSD3	ENSG00000156011.16	Esophagus - Mucosa	C=0.897	T=0.103	0.198321	3.37E-05
rs57557661	rs75123216	chr8:18821251	0.000331918	1	PSD3	ENSG00000156011.16	Esophagus - Muscularis	T=0.982	C=0.018	0.413555	3.38E-05
rs57557661	rs116971711	chr8:18822209	0.000331918	1	PSD3	ENSG00000156011.16	Esophagus - Muscularis	G=0.982	A=0.018	0.413555	3.38E-05
rs57557661	rs335248	chr8:18646733	0.008152506	1	PSD3	ENSG00000156011.16	Brain - Cerebellum	G=0.691	C=0.309	0.203295	3.48E-05
rs57557661	rs77537870	chr8:18877244	0.002213857	1	PSD3	ENSG00000156011.16	Esophagus - Mucosa	A=0.892	G=0.108	0.202602	3.49E-05
rs57557661	rs335237	chr8:18641291	0.009593114	1	PSD3	ENSG00000156011.16	Brain - Cerebellum	T=0.655	C=0.345	0.200677	3.52E-05
rs57557661	rs61050926	chr8:18864611	0.002191104	1	PSD3	ENSG00000156011.16	Esophagus - Mucosa	G=0.893	A=0.107	0.185851	4.35E-05
rs57557661	rs57965775	chr8:18865248	0.002191104	1	PSD3	ENSG00000156011.16	Esophagus - Mucosa	C=0.893	G=0.107	0.185851	4.35E-05
rs57557661	rs73199994	chr8:18646365	0.001595361	1	PSD3	ENSG00000156011.16	Brain - Cerebellum	C=0.919	T=0.081	0.375659	4.63E-05
rs57557661	rs76595146	chr8:18873146	0.002213857	1	PSD3	ENSG00000156011.16	Esophagus - Mucosa	G=0.892	T=0.108	0.198351	5.28E-05
rs57557661	rs79513952	chr8:18875713	0.002213857	1	PSD3	ENSG00000156011.16	Esophagus - Mucosa	G=0.892	A=0.108	0.198351	5.28E-05
rs57557661	rs115432345	chr8:18871863	0.002236661	1	PSD3	ENSG00000156011.16	Esophagus - Mucosa	G=0.891	T=0.109	0.194814	6.45E-05
rs57557661	rs73594633	chr8:18871992	0.002213857	1	PSD3	ENSG00000156011.16	Esophagus - Mucosa	C=0.892	G=0.108	0.189659	6.52E-05
rs57557661	rs145752806	chr8:18882886	0.002100595	1	PSD3	ENSG00000156011.16	Esophagus - Mucosa	ACTC=0.897	-=0.103	0.194717	7.13E-05
rs57557661	rs78119471	chr8:18883327	0.002100595	1	PSD3	ENSG00000156011.16	Esophagus - Mucosa	A=0.897	G=0.103	0.194717	7.13E-05
rs57557661	rs60074764	chr8:18863855	0.002213857	1	PSD3	ENSG00000156011.16	Esophagus - Mucosa	T=0.892	C=0.108	0.173434	8.93E-05
rs57557661	rs60016927	chr8:18863989	0.002213857	1	PSD3	ENSG00000156011.16	Esophagus - Mucosa	T=0.892	C=0.108	0.173434	8.93E-05
rs57557661	rs79233079	chr8:19029881	0.002259516	1	PSD3	ENSG00000156011.16	Cells - Cultured fibroblasts	C=0.89	T=0.11	-0.168053	0.000116145
rs73666715	rs547180436	chr8:18871342	0.000172581	1	PSD3	ENSG00000156011.16	Esophagus - Muscularis	-=0.99	A=0.01	0.41154	8.21E-19
rs73666715	rs2632845	chr8:18660720	0.015890538	0.800752624	PSD3	ENSG00000156011.16	Brain - Spinal cord (cervical c-1)	C=0.59	G=0.41	-0.519542	3.39E-10
rs73666715	rs2638625	chr8:18659269	0.015969366	0.801087494	PSD3	ENSG00000156011.16	Brain - Spinal cord (cervical c-1)	C=0.591	T=0.409	-0.506856	1.08E-09
rs73666715	rs547180436	chr8:18871342	0.000172581	1	PSD3	ENSG00000156011.16	Esophagus - Gastroesophageal Junctio	-=0.99	A=0.01	0.267169	5.99E-09
rs73666715	rs10088636	chr8:18355945	0.00213183	1	PSD3	ENSG00000156011.16	Brain - Cerebellar Hemisphere	G=0.89	T=0.11	-0.381687	2.71E-08
rs73666715	rs7009615	chr8:18572835	0.001960659	1	PSD3	ENSG00000156011.16	Small Intestine - Terminal Ileum	T=0.898	C=0.102	0.37749	1.38E-07
rs73666715	rs138983771	chr8:18919610	0.000242588	1	PSD3	ENSG00000156011.16	Esophagus - Muscularis	G=0.986	A=0.014	0.754799	1.43E-07
rs73666715	rs3214999	chr8:18656888	0.031741385	0.837650286	PSD3	ENSG00000156011.16	Brain - Substantia nigra	G=0.725	-=0.275	-0.269949	2.63E-07
rs73666715	rs10503636	chr8:18654794	0.014315101	0.885759709	PSD3	ENSG00000156011.16	Brain - Spinal cord (cervical c-1)	T=0.485	C=0.515	0.423645	8.47E-07
rs73666715	rs3214999	chr8:18656888	0.031741385	0.837650286	PSD3	ENSG00000156011.16	Brain - Spinal cord (cervical c-1)	G=0.725	-=0.275	-0.461094	2.06E-06
rs73666715	rs60854172	chr8:18867056	0.002067284	1	PSD3	ENSG00000156011.16	Esophagus - Mucosa	-=0.893	A=0.107	0.215939	2.93E-06
rs73666715	rs111916415	chr8:18656221	0.020649464	1	PSD3	ENSG00000156011.16	Brain - Spinal cord (cervical c-1)	A=0.454	T=0.546	0.411994	2.93E-06
rs73666715	rs2632845	chr8:18660720	0.015890538	0.800752624	PSD3	ENSG00000156011.16	Brain - Substantia nigra	C=0.59	G=0.41	-0.241026	3.37E-06
rs73666715	rs17696125	chr8:18574874	0.000786195	1	PSD3	ENSG00000156011.16	Small Intestine - Terminal Ileum	T=0.956	C=0.044	0.410081	3.38E-06
rs73666715	rs7841096	chr8:18632333	0.027609403	1	PSD3	ENSG00000156011.16	Brain - Spinal cord (cervical c-1)	A=0.384	T=0.616	0.378279	3.44E-06
rs73666715	rs59321198	chr8:18866979	0.002045864	1	PSD3	ENSG00000156011.16	Esophagus - Mucosa	C=0.894	G=0.106	0.217409	3.55E-06
rs73666715	rs2638625	chr8:18659269	0.015969366	0.801087494	PSD3	ENSG00000156011.16	Brain - Substantia nigra	C=0.591	T=0.409	-0.23662	4.89E-06
rs73666715	rs78656518	chr8:18844514	0.000103134	1	PSD3	ENSG00000156011.16	Esophagus - Muscularis	T=0.994	C=0.006	0.494999	4.95E-06
rs73666715	rs111699729	chr8:18865650	0.002045864	1	PSD3	ENSG00000156011.16	Esophagus - Mucosa	G=0.894	A=0.106	0.213434	5.55E-06
rs73666715	rs7010324	chr8:18866227	0.001981889	1	PSD3	ENSG00000156011.16	Esophagus - Mucosa	C=0.897	T=0.103	0.213434	5.55E-06
rs73666715	rs6996112	chr8:18866326	0.002067284	1	PSD3	ENSG00000156011.16	Esophagus - Mucosa	T=0.893	C=0.107	0.213434	5.55E-06
rs73666715	rs7010496	chr8:18866368	0.002045864	1	PSD3	ENSG00000156011.16	Esophagus - Mucosa	C=0.894	T=0.106	0.213434	5.55E-06

rs73666715	rs117966479	chr8:18893665	0.000936931	1	PSD3	ENSG00000156011.16	Small Intestine - Terminal Ileum	T=0.948	C=0.052	0.630419	5.82E-06
rs73666715	rs77386793	chr8:18869663	0.002045864	1	PSD3	ENSG00000156011.16	Esophagus - Mucosa	C=0.894	A=0.106	0.225491	6.32E-06
rs73666715	rs34426360	chr8:18575404	0.000767529	1	PSD3	ENSG00000156011.16	Small Intestine - Terminal Ileum	-=0.957	AGA=0.043	0.457749	7.58E-06
rs73666715	rs1872890	chr8:18575581	0.000748902	1	PSD3	ENSG00000156011.16	Small Intestine - Terminal Ileum	A=0.958	G=0.042	0.457749	7.58E-06
rs73666715	rs1038611	chr8:18624327	0.03018685	1	PSD3	ENSG00000156011.16	Brain - Spinal cord (cervical c-1)	C=0.363	T=0.637	0.378354	7.96E-06
rs73666715	rs1038610	chr8:18624424	0.03018685	1	PSD3	ENSG00000156011.16	Brain - Spinal cord (cervical c-1)	C=0.363	T=0.637	0.378354	7.96E-06
rs73666715	rs146517899	chr8:18867154	0.002067284	1	PSD3	ENSG00000156011.16	Esophagus - Mucosa	C=0.893	T=0.107	0.210362	8.66E-06
rs73666715	rs56990136	chr8:18872866	0.002088751	1	PSD3	ENSG00000156011.16	Esophagus - Mucosa	C=0.892	T=0.108	0.21699	9.52E-06
rs73666715	rs73594648	chr8:18876592	0.002088751	1	PSD3	ENSG00000156011.16	Esophagus - Mucosa	A=0.892	T=0.108	0.208395	1.24E-05
rs73666715	rs141114042	chr8:18631690	0.02819731	1	PSD3	ENSG00000156011.16	Brain - Spinal cord (cervical c-1)	TTAA=0.379	-=0.621	0.367347	1.35E-05
rs73666715	rs116324874	chr8:18876557	0.002088751	1	PSD3	ENSG00000156011.16	Esophagus - Mucosa	G=0.892	A=0.108	0.219157	1.52E-05
rs73666715	rs80027157	chr8:18866078	0.002088751	1	PSD3	ENSG00000156011.16	Esophagus - Mucosa	A=0.892	G=0.108	0.208302	1.57E-05
rs73666715	rs7009185	chr8:18866220	0.002045864	1	PSD3	ENSG00000156011.16	Esophagus - Mucosa	G=0.894	A=0.106	0.208302	1.57E-05
rs73666715	rs7010821	chr8:18866541	0.002045864	1	PSD3	ENSG00000156011.16	Esophagus - Mucosa	C=0.894	T=0.106	0.208302	1.57E-05
rs73666715	rs375703979	chr8:18866779	0.002045864	1	PSD3	ENSG00000156011.16	Esophagus - Mucosa	AG=0.894	-=0.106	0.208302	1.57E-05
rs73666715	rs35675493	chr8:18653677	0.01651894	1	PSD3	ENSG00000156011.16	Brain - Spinal cord (cervical c-1)	CCC=0.51	-=0.49	0.381151	1.59E-05
rs73666715	rs9644614	chr8:18630435	0.030844849	1	PSD3	ENSG00000156011.16	Brain - Spinal cord (cervical c-1)	C=0.358	T=0.642	0.374175	1.59E-05
rs73666715	rs9644615	chr8:18630532	0.030711791	1	PSD3	ENSG00000156011.16	Brain - Spinal cord (cervical c-1)	C=0.359	A=0.641	0.374175	1.59E-05
rs73666715	rs189718418	chr8:18857486	8.59E-05	1	PSD3	ENSG00000156011.16	Esophagus - Muscularis	A=0.995	G=0.005	0.496246	1.84E-05
rs73666715	rs2410598	chr8:18872462	0.002088751	1	PSD3	ENSG00000156011.16	Esophagus - Mucosa	C=0.892	T=0.108	0.204344	1.89E-05
rs73666715	rs57855654	chr8:18872943	0.002088751	1	PSD3	ENSG00000156011.16	Esophagus - Mucosa	G=0.892	A=0.108	0.204344	1.89E-05
rs73666715	rs335232	chr8:18636126	0.007656056	1	PSD3	ENSG00000156011.16	Brain - Cerebellum	G=0.692	A=0.308	0.211844	2.05E-05
rs73666715	rs10111009	chr8:18973729	0.003873301	1	PSD3	ENSG00000156011.16	Skin - Sun Exposed (Lower leg)	C=0.816	T=0.184	0.172705	2.10E-05
rs73666715	rs76653031	chr8:18574691	0.000313161	1	PSD3	ENSG00000156011.16	Small Intestine - Terminal Ileum	C=0.982	A=0.018	0.614718	2.15E-05
rs73666715	rs35592282	chr8:18576601	0.000313161	1	PSD3	ENSG00000156011.16	Small Intestine - Terminal Ileum	A=0.982	-=0.018	0.614718	2.15E-05
rs73666715	rs143185883	chr8:18577856	0.000313161	1	PSD3	ENSG00000156011.16	Small Intestine - Terminal Ileum	C=0.982	-=0.018	0.614718	2.15E-05
rs73666715	rs79697401	chr8:18578261	0.000313161	1	PSD3	ENSG00000156011.16	Small Intestine - Terminal Ileum	T=0.982	C=0.018	0.614718	2.15E-05
rs73666715	rs17127092	chr8:18579361	0.000313161	1	PSD3	ENSG00000156011.16	Small Intestine - Terminal Ileum	A=0.982	G=0.018	0.614718	2.15E-05
rs73666715	rs12056418	chr8:18580835	0.000313161	1	PSD3	ENSG00000156011.16	Small Intestine - Terminal Ileum	C=0.982	T=0.018	0.614718	2.15E-05
rs73666715	rs76973373	chr8:18583218	0.000313161	1	PSD3	ENSG00000156011.16	Small Intestine - Terminal Ileum	C=0.982	T=0.018	0.614718	2.15E-05
rs73666715	rs79550828	chr8:18587562	0.000313161	1	PSD3	ENSG00000156011.16	Small Intestine - Terminal Ileum	G=0.982	C=0.018	0.614718	2.15E-05
rs73666715	rs79052288	chr8:18589608	0.000313161	1	PSD3	ENSG00000156011.16	Small Intestine - Terminal Ileum	C=0.982	G=0.018	0.614718	2.15E-05
rs73666715	rs57825650	chr8:18591403	0.000313161	1	PSD3	ENSG00000156011.16	Small Intestine - Terminal Ileum	A=0.982	G=0.018	0.614718	2.15E-05
rs73666715	rs335234	chr8:18637444	0.007835832	1	PSD3	ENSG00000156011.16	Brain - Cerebellum	C=0.687	T=0.313	0.2134	2.22E-05
rs73666715	rs6995932	chr8:18362067	0.003372412	1	PSD3	ENSG00000156011.16	Brain - Cerebellar Hemisphere	G=0.836	A=0.164	-0.253274	2.38E-05
rs73666715	rs60549282	chr8:18877472	0.001981889	1	PSD3	ENSG00000156011.16	Esophagus - Mucosa	C=0.897	T=0.103	0.198321	3.37E-05
rs73666715	rs4463468	chr8:18879717	0.001981889	1	PSD3	ENSG00000156011.16	Esophagus - Mucosa	C=0.897	T=0.103	0.198321	3.37E-05
rs73666715	rs75123216	chr8:18821251	0.000313161	1	PSD3	ENSG00000156011.16	Esophagus - Muscularis	T=0.982	C=0.018	0.413555	3.38E-05
rs73666715	rs116971711	chr8:18822209	0.000313161	1	PSD3	ENSG00000156011.16	Esophagus - Muscularis	G=0.982	A=0.018	0.413555	3.38E-05
rs73666715	rs335248	chr8:18646733	0.007691804	1	PSD3	ENSG00000156011.16	Brain - Cerebellum	G=0.691	C=0.309	0.203295	3.48E-05
rs73666715	rs77537870	chr8:18877244	0.002088751	1	PSD3	ENSG00000156011.16	Esophagus - Mucosa	A=0.892	G=0.108	0.202602	3.49E-05
rs73666715	rs335237	chr8:18641291	0.009051003	1	PSD3	ENSG00000156011.16	Brain - Cerebellum	T=0.655	C=0.345	0.200677	3.52E-05
rs73666715	rs61050926	chr8:18864611	0.002067284	1	PSD3	ENSG00000156011.16	Esophagus - Mucosa	G=0.893	A=0.107	0.185851	4.35E-05
rs73666715	rs57965775	chr8:18865248	0.002067284	1	PSD3	ENSG00000156011.16	Esophagus - Mucosa	C=0.893	G=0.107	0.185851	4.35E-05
rs73666715	rs73199994	chr8:18646365	0.001505206	1	PSD3	ENSG00000156011.16	Brain - Cerebellum	C=0.919	T=0.081	0.375659	4.63E-05
rs73666715	rs6995932	chr8:18362067	0.003372412	1	PSD3	ENSG00000156011.16	Brain - Cerebellum	G=0.836	A=0.164	-0.26983	4.92E-05
rs73666715	rs76595146	chr8:18873146	0.002088751	1	PSD3	ENSG00000156011.16	Esophagus - Mucosa	G=0.892	T=0.108	0.198351	5.28E-05
rs73666715	rs79513952	chr8:18875713	0.002088751	1	PSD3	ENSG00000156011.16	Esophagus - Mucosa	G=0.892	A=0.108	0.198351	5.28E-05
rs73666715	rs115432345	chr8:18871863	0.002110267	1	PSD3	ENSG00000156011.16	Esophagus - Mucosa	G=0.891	T=0.109	0.194814	6.45E-05
rs73666715	rs73594633	chr8:18871992	0.002088751	1	PSD3	ENSG00000156011.16	Esophagus - Mucosa	C=0.892	G=0.108	0.189659	6.52E-05
rs73666715	rs145752806	chr8:18882886	0.001981889	1	PSD3	ENSG00000156011.16	Esophagus - Mucosa	ACTC=0.897	-=0.103	0.194717	7.13E-05

rs73666715	rs78119471	chr8:18883327	0.001981889	1	PSD3	ENSG00000156011.16	Esophagus - Mucosa	A=0.897	G=0.103	0.194717	7.13E-05
rs73666715	rs60074764	chr8:18863855	0.002088751	1	PSD3	ENSG00000156011.16	Esophagus - Mucosa	T=0.892	C=0.108	0.173434	8.93E-05
rs73666715	rs60016927	chr8:18863989	0.002088751	1	PSD3	ENSG00000156011.16	Esophagus - Mucosa	T=0.892	C=0.108	0.173434	8.93E-05
rs73666715	rs79233079	chr8:19029881	0.00213183	1	PSD3	ENSG00000156011.16	Cells - Cultured fibroblasts	C=0.89	T=0.11	-0.168053	0.000116145
rs7814472	rs138983771	chr8:18919610	0.000554019	1	PSD3	ENSG00000156011.16	Esophagus - Muscularis	G=0.986	A=0.014	0.754799	1.43E-07
rs7814472	rs111916415	chr8:18656221	0.034501417	0.855335059	PSD3	ENSG00000156011.16	Brain - Spinal cord (cervical c-1)	A=0.454	T=0.546	0.411994	2.93E-06
rs7814472	rs78656518	chr8:18844514	0.000235537	1	PSD3	ENSG00000156011.16	Esophagus - Muscularis	T=0.994	C=0.006	0.494999	4.95E-06
rs7814472	rs117966479	chr8:18893665	0.002139751	1	PSD3	ENSG00000156011.16	Small Intestine - Terminal Ileum	T=0.948	C=0.052	0.630419	5.82E-06
rs7814472	rs141114042	chr8:18631690	0.064396651	1	PSD3	ENSG00000156011.16	Brain - Spinal cord (cervical c-1)	TTAA=0.379	-=0.621	0.367347	1.35E-05
rs7814472	rs35675493	chr8:18653677	0.033782847	0.946300843	PSD3	ENSG00000156011.16	Brain - Spinal cord (cervical c-1)	CCC=0.51	-=0.49	0.381151	1.59E-05
rs7814472	rs189718418	chr8:18857486	0.000196085	1	PSD3	ENSG00000156011.16	Esophagus - Muscularis	A=0.995	G=0.005	0.496246	1.84E-05
rs7814472	rs75123216	chr8:18821251	0.000715194	1	PSD3	ENSG00000156011.16	Esophagus - Muscularis	T=0.982	C=0.018	0.413555	3.38E-05
rs7814472	rs116971711	chr8:18822209	0.000715194	1	PSD3	ENSG00000156011.16	Esophagus - Muscularis	G=0.982	A=0.018	0.413555	3.38E-05
rs7814472	rs73199994	chr8:18646365	0.00343757	1	PSD3	ENSG00000156011.16	Brain - Cerebellum	C=0.919	T=0.081	0.375659	4.63E-05
rs7830088	rs547180436	chr8:18871342	0.000298019	1	PSD3	ENSG00000156011.16	Esophagus - Muscularis	-=0.99	A=0.01	0.41154	8.21E-19
rs7830088	rs547180436	chr8:18871342	0.000298019	1	PSD3	ENSG00000156011.16	Esophagus - Gastroesophageal Junctio	-=0.99	A=0.01	0.267169	5.99E-09
rs7830088	rs138983771	chr8:18919610	0.000418909	1	PSD3	ENSG00000156011.16	Esophagus - Muscularis	G=0.986	A=0.014	0.754799	1.43E-07
rs7830088	rs10503636	chr8:18654794	0.023632651	0.866063107	PSD3	ENSG00000156011.16	Brain - Spinal cord (cervical c-1)	T=0.485	C=0.515	0.423645	8.47E-07
rs7830088	rs2131183	chr8:18664444	0.027608351	0.821494056	PSD3	ENSG00000156011.16	Brain - Spinal cord (cervical c-1)	G=0.42	C=0.58	0.422546	1.02E-06
rs7830088	rs111916415	chr8:18656221	0.031294312	0.936813014	PSD3	ENSG00000156011.16	Brain - Spinal cord (cervical c-1)	A=0.454	T=0.546	0.411994	2.93E-06
rs7830088	rs78656518	chr8:18844514	0.000178096	1	PSD3	ENSG00000156011.16	Esophagus - Muscularis	T=0.994	C=0.006	0.494999	4.95E-06
rs7830088	rs117966479	chr8:18893665	0.001617925	1	PSD3	ENSG00000156011.16	Small Intestine - Terminal Ileum	T=0.948	C=0.052	0.630419	5.82E-06
rs7830088	rs692014	chr8:19002618	0.007089184	0.858985142	PSD3	ENSG00000156011.16	Skin - Sun Exposed (Lower leg)	T=0.755	C=0.245	0.179487	8.72E-06
rs7830088	rs141114042	chr8:18631690	0.048692097	1	PSD3	ENSG00000156011.16	Brain - Spinal cord (cervical c-1)	TTAA=0.379	-=0.621	0.367347	1.35E-05
rs7830088	rs35675493	chr8:18653677	0.024652358	0.929635588	PSD3	ENSG00000156011.16	Brain - Spinal cord (cervical c-1)	CCC=0.51	-=0.49	0.381151	1.59E-05
rs7830088	rs189718418	chr8:18857486	0.000148265	1	PSD3	ENSG00000156011.16	Esophagus - Muscularis	A=0.995	G=0.005	0.496246	1.84E-05
rs7830088	rs335232	chr8:18636126	0.013220744	1	PSD3	ENSG00000156011.16	Brain - Cerebellum	G=0.692	A=0.308	0.211844	2.05E-05
rs7830088	rs10111009	chr8:18973729	0.00668855	1	PSD3	ENSG00000156011.16	Skin - Sun Exposed (Lower leg)	C=0.816	T=0.184	0.172705	2.10E-05
rs7830088	rs335234	chr8:18637444	0.013531188	1	PSD3	ENSG00000156011.16	Brain - Cerebellum	C=0.687	T=0.313	0.2134	2.22E-05
rs7830088	rs75123216	chr8:18821251	0.000540778	1	PSD3	ENSG00000156011.16	Esophagus - Muscularis	T=0.982	C=0.018	0.413555	3.38E-05
rs7830088	rs116971711	chr8:18822209	0.000540778	1	PSD3	ENSG00000156011.16	Esophagus - Muscularis	G=0.982	A=0.018	0.413555	3.38E-05
rs7830088	rs335248	chr8:18646733	0.013282475	1	PSD3	ENSG00000156011.16	Brain - Cerebellum	G=0.691	C=0.309	0.203295	3.48E-05
rs7830088	rs335237	chr8:18641291	0.015629587	1	PSD3	ENSG00000156011.16	Brain - Cerebellum	T=0.655	C=0.345	0.200677	3.52E-05
rs7830088	rs73199994	chr8:18646365	0.002599242	1	PSD3	ENSG00000156011.16	Brain - Cerebellum	C=0.919	T=0.081	0.375659	4.63E-05
rs7830088	rs333070	chr8:19006584	0.00926434	0.879966591	PSD3	ENSG00000156011.16	Skin - Sun Exposed (Lower leg)	T=0.713	A=0.287	0.149087	6.39E-05
rs7836892	rs547180436	chr8:18871342	0.000319226	1	PSD3	ENSG00000156011.16	Esophagus - Muscularis	-=0.99	A=0.01	0.41154	8.21E-19
rs7836892	rs547180436	chr8:18871342	0.000319226	1	PSD3	ENSG00000156011.16	Esophagus - Gastroesophageal Junctio	-=0.99	A=0.01	0.267169	5.99E-09
rs7836892	rs138983771	chr8:18919610	0.000448718	1	PSD3	ENSG00000156011.16	Esophagus - Muscularis	G=0.986	A=0.014	0.754799	1.43E-07
rs7836892	rs111916415	chr8:18656221	0.025850161	0.822668782	PSD3	ENSG00000156011.16	Brain - Spinal cord (cervical c-1)	A=0.454	T=0.546	0.411994	2.93E-06
rs7836892	rs78656518	chr8:18844514	0.000190769	1	PSD3	ENSG00000156011.16	Esophagus - Muscularis	T=0.994	C=0.006	0.494999	4.95E-06
rs7836892	rs117966479	chr8:18893665	0.001733054	1	PSD3	ENSG00000156011.16	Small Intestine - Terminal Ileum	T=0.948	C=0.052	0.630419	5.82E-06
rs7836892	rs141114042	chr8:18631690	0.052156942	1	PSD3	ENSG00000156011.16	Brain - Spinal cord (cervical c-1)	TTAA=0.379	-=0.621	0.367347	1.35E-05
rs7836892	rs35675493	chr8:18653677	0.026665107	0.934175227	PSD3	ENSG00000156011.16	Brain - Spinal cord (cervical c-1)	CCC=0.51	-=0.49	0.381151	1.59E-05
rs7836892	rs189718418	chr8:18857486	0.000158816	1	PSD3	ENSG00000156011.16	Esophagus - Muscularis	A=0.995	G=0.005	0.496246	1.84E-05
rs7836892	rs335232	chr8:18636126	0.014161509	1	PSD3	ENSG00000156011.16	Brain - Cerebellum	G=0.692	A=0.308	0.211844	2.05E-05
rs7836892	rs10111009	chr8:18973729	0.007164496	1	PSD3	ENSG00000156011.16	Skin - Sun Exposed (Lower leg)	C=0.816	T=0.184	0.172705	2.10E-05
rs7836892	rs335234	chr8:18637444	0.014494044	1	PSD3	ENSG00000156011.16	Brain - Cerebellum	C=0.687	T=0.313	0.2134	2.22E-05
rs7836892	rs75123216	chr8:18821251	0.000579259	1	PSD3	ENSG00000156011.16	Esophagus - Muscularis	T=0.982	C=0.018	0.413555	3.38E-05
rs7836892	rs116971711	chr8:18822209	0.000579259	1	PSD3	ENSG00000156011.16	Esophagus - Muscularis	G=0.982	A=0.018	0.413555	3.38E-05
rs7836892	rs335248	chr8:18646733	0.014227633	1	PSD3	ENSG00000156011.16	Brain - Cerebellum	G=0.691	C=0.309	0.203295	3.48E-05
rs7836892	rs335237	chr8:18641291	0.016741761	1	PSD3	ENSG00000156011.16	Brain - Cerebellum	T=0.655	C=0.345	0.200677	3.52E-05

rs7836892	rs73199994	chr8:18646365	0.0027842	1	PSD3	ENSG00000156011.16	Brain - Cerebellum	C=0.919	T=0.081	0.375659	4.63E-05
rs7836892	rs6983992	chr8:18728635	0.004025557	0.803323558	PSD3	ENSG00000156011.16	Heart - Atrial Appendage	T=0.836	C=0.164	0.234918	5.40E-05
rs7839578	rs547180436	chr8:18871342	0.000308611	1	PSD3	ENSG00000156011.16	Esophagus - Muscularis	-=0.99	A=0.01	0.41154	8.21E-19
rs7839578	rs547180436	chr8:18871342	0.000308611	1	PSD3	ENSG00000156011.16	Esophagus - Gastroesophageal Junctio	-=0.99	A=0.01	0.267169	5.99E-09
rs7839578	rs138983771	chr8:18919610	0.000433798	1	PSD3	ENSG00000156011.16	Esophagus - Muscularis	G=0.986	A=0.014	0.754799	1.43E-07
rs7839578	rs10503636	chr8:18654794	0.024725583	0.870527671	PSD3	ENSG00000156011.16	Brain - Spinal cord (cervical c-1)	T=0.485	C=0.515	0.423645	8.47E-07
rs7839578	rs2131183	chr8:18664444	0.029005283	0.827444254	PSD3	ENSG00000156011.16	Brain - Spinal cord (cervical c-1)	G=0.42	C=0.58	0.422546	1.02E-06
rs7839578	rs111916415	chr8:18656221	0.032552479	0.938919247	PSD3	ENSG00000156011.16	Brain - Spinal cord (cervical c-1)	A=0.454	T=0.546	0.411994	2.93E-06
rs7839578	rs78656518	chr8:18844514	0.000184426	1	PSD3	ENSG00000156011.16	Esophagus - Muscularis	T=0.994	C=0.006	0.494999	4.95E-06
rs7839578	rs117966479	chr8:18893665	0.00167543	1	PSD3	ENSG00000156011.16	Small Intestine - Terminal Ileum	T=0.948	C=0.052	0.630419	5.82E-06
rs7839578	rs692014	chr8:19002618	0.007421716	0.863685637	PSD3	ENSG00000156011.16	Skin - Sun Exposed (Lower leg)	T=0.755	C=0.245	0.179487	8.72E-06
rs7839578	rs141114042	chr8:18631690	0.050422744	1	PSD3	ENSG00000156011.16	Brain - Spinal cord (cervical c-1)	TTAA=0.379	-=0.621	0.367347	1.35E-05
rs7839578	rs35675493	chr8:18653677	0.025657549	0.931981068	PSD3	ENSG00000156011.16	Brain - Spinal cord (cervical c-1)	CCC=0.51	-=0.49	0.381151	1.59E-05
rs7839578	rs189718418	chr8:18857486	0.000153535	1	PSD3	ENSG00000156011.16	Esophagus - Muscularis	A=0.995	G=0.005	0.496246	1.84E-05
rs7839578	rs335232	chr8:18636126	0.013690644	1	PSD3	ENSG00000156011.16	Brain - Cerebellum	G=0.692	A=0.308	0.211844	2.05E-05
rs7839578	rs10111009	chr8:18973729	0.004642915	0.818738739	PSD3	ENSG00000156011.16	Skin - Sun Exposed (Lower leg)	C=0.816	T=0.184	0.172705	2.10E-05
rs7839578	rs335234	chr8:18637444	0.011187596	0.893544974	PSD3	ENSG00000156011.16	Brain - Cerebellum	C=0.687	T=0.313	0.2134	2.22E-05
rs7839578	rs75123216	chr8:18821251	0.000559999	1	PSD3	ENSG00000156011.16	Esophagus - Muscularis	T=0.982	C=0.018	0.413555	3.38E-05
rs7839578	rs116971711	chr8:18822209	0.000559999	1	PSD3	ENSG00000156011.16	Esophagus - Muscularis	G=0.982	A=0.018	0.413555	3.38E-05
rs7839578	rs335248	chr8:18646733	0.01375457	1	PSD3	ENSG00000156011.16	Brain - Cerebellum	G=0.691	C=0.309	0.203295	3.48E-05
rs7839578	rs335237	chr8:18641291	0.016185104	1	PSD3	ENSG00000156011.16	Brain - Cerebellum	T=0.655	C=0.345	0.200677	3.52E-05
rs7839578	rs73199994	chr8:18646365	0.002691626	1	PSD3	ENSG00000156011.16	Brain - Cerebellum	C=0.919	T=0.081	0.375659	4.63E-05
rs79441119	rs11778625	chr8:18668681	0.014971975	0.824248777	PSD3	ENSG00000156011.16	Brain - Spinal cord (cervical c-1)	T=0.526	G=0.474	0.51445	3.05E-09
rs79441119	rs11775742	chr8:18665710	0.015793237	0.828210383	PSD3	ENSG00000156011.16	Brain - Spinal cord (cervical c-1)	T=0.515	C=0.485	0.50233	8.42E-09
rs79441119	rs11774752	chr8:18664654	0.016571315	0.831659973	PSD3	ENSG00000156011.16	Brain - Spinal cord (cervical c-1)	T=0.505	C=0.495	0.487492	2.02E-08
rs79441119	rs11775676	chr8:18665492	0.01549051	0.826790634	PSD3	ENSG00000156011.16	Brain - Spinal cord (cervical c-1)	T=0.519	C=0.481	0.487492	2.02E-08
rs79441119	rs11780950	chr8:18668483	0.014754011	0.823136428	PSD3	ENSG00000156011.16	Brain - Spinal cord (cervical c-1)	G=0.529	C=0.471	0.48504	2.14E-08
rs79441119	rs2069245	chr8:18657093	0.016023429	0.829260014	PSD3	ENSG00000156011.16	Brain - Spinal cord (cervical c-1)	G=0.512	C=0.488	0.482474	3.30E-08
rs79441119	rs11774165	chr8:18658950	0.016810857	0.832667997	PSD3	ENSG00000156011.16	Brain - Spinal cord (cervical c-1)	T=0.502	G=0.498	0.482474	3.30E-08
rs79441119	rs11786921	chr8:18657985	0.016891348	0.833001328	PSD3	ENSG00000156011.16	Brain - Spinal cord (cervical c-1)	T=0.501	C=0.499	0.46989	4.88E-08
rs79441119	rs11786923	chr8:18658007	0.016810857	0.832667997	PSD3	ENSG00000156011.16	Brain - Spinal cord (cervical c-1)	T=0.502	G=0.498	0.46989	4.88E-08
rs79441119	rs13276530	chr8:18658346	0.017134784	0.833993399	PSD3	ENSG00000156011.16	Brain - Spinal cord (cervical c-1)	A=0.498	G=0.502	0.46989	4.88E-08
rs79441119	rs36085278	chr8:18659470	0.017381201	0.834973753	PSD3	ENSG00000156011.16	Brain - Spinal cord (cervical c-1)	A=0.495	G=0.505	0.46989	4.88E-08
rs79441119	rs11993401	chr8:18657427	0.016650842	0.831997328	PSD3	ENSG00000156011.16	Brain - Spinal cord (cervical c-1)	C=0.504	T=0.496	0.468976	8.04E-08
rs79441119	rs11993467	chr8:18657661	0.016571315	0.831659973	PSD3	ENSG00000156011.16	Brain - Spinal cord (cervical c-1)	C=0.505	T=0.495	0.468976	8.04E-08
rs79441119	rs11997879	chr8:18657450	0.016730689	0.832333333	PSD3	ENSG00000156011.16	Brain - Spinal cord (cervical c-1)	T=0.503	A=0.497	0.464045	8.13E-08
rs79441119	rs11986109	chr8:18657632	0.016730689	0.832333333	PSD3	ENSG00000156011.16	Brain - Spinal cord (cervical c-1)	G=0.503	C=0.497	0.464045	8.13E-08
rs79441119	rs34059664	chr8:18657698	0.016650842	0.831997328	PSD3	ENSG00000156011.16	Brain - Spinal cord (cervical c-1)	A=0.504	G=0.496	0.464045	8.13E-08
rs79441119	rs7009615	chr8:18572835	0.00278772	1	PSD3	ENSG00000156011.16	Small Intestine - Terminal Ileum	T=0.898	C=0.102	0.37749	1.38E-07
rs79441119	rs138983771	chr8:18919610	0.000344918	1	PSD3	ENSG00000156011.16	Esophagus - Muscularis	G=0.986	A=0.014	0.754799	1.43E-07
rs79441119	rs2069245	chr8:18657093	0.016023429	0.829260014	PSD3	ENSG00000156011.16	Brain - Substantia nigra	G=0.512	C=0.488	0.289388	3.50E-07
rs79441119	rs11780950	chr8:18668483	0.014754011	0.823136428	PSD3	ENSG00000156011.16	Brain - Substantia nigra	G=0.529	C=0.471	0.294511	3.71E-07
rs79441119	rs11775676	chr8:18665492	0.01549051	0.826790634	PSD3	ENSG00000156011.16	Brain - Substantia nigra	T=0.519	C=0.481	0.278113	5.86E-07
rs79441119	rs11778625	chr8:18668681	0.014971975	0.824248777	PSD3	ENSG00000156011.16	Brain - Substantia nigra	T=0.526	G=0.474	0.286342	6.80E-07
rs79441119	rs10503636	chr8:18654794	0.018224815	0.838159588	PSD3	ENSG00000156011.16	Brain - Spinal cord (cervical c-1)	T=0.485	C=0.515	0.423645	8.47E-07
rs79441119	rs11774752	chr8:18664654	0.016571315	0.831659973	PSD3	ENSG00000156011.16	Brain - Substantia nigra	T=0.505	C=0.495	0.272367	9.41E-07
rs79441119	rs11786921	chr8:18657985	0.016891348	0.833001328	PSD3	ENSG00000156011.16	Brain - Substantia nigra	T=0.501	C=0.499	0.268879	9.98E-07
rs79441119	rs11786923	chr8:18658007	0.016810857	0.832667997	PSD3	ENSG00000156011.16	Brain - Substantia nigra	T=0.502	G=0.498	0.268879	9.98E-07
rs79441119	rs13276530	chr8:18658346	0.017134784	0.833993399	PSD3	ENSG00000156011.16	Brain - Substantia nigra	A=0.498	G=0.502	0.268879	9.98E-07
rs79441119	rs11774165	chr8:18658950	0.016810857	0.832667997	PSD3	ENSG00000156011.16	Brain - Substantia nigra	T=0.502	G=0.498	0.268879	9.98E-07
rs79441119	rs36085278	chr8:18659470	0.017381201	0.834973753	PSD3	ENSG00000156011.16	Brain - Substantia nigra	A=0.495	G=0.505	0.268879	9.98E-07

rs79441119	rs11775742	chr8:18665710	0.015793237	0.828210383	PSD3	ENSG00000156011.16	Brain - Substantia nigra	T=0.515	C=0.485	0.27395	1.42E-06
rs79441119	rs11986109	chr8:18657632	0.016730689	0.832333333	PSD3	ENSG00000156011.16	Brain - Substantia nigra	G=0.503	C=0.497	0.267572	1.94E-06
rs79441119	rs60854172	chr8:18867056	0.002939322	1	PSD3	ENSG00000156011.16	Esophagus - Mucosa	-=0.893	A=0.107	0.215939	2.93E-06
rs79441119	rs111916415	chr8:18656221	0.025047817	0.923649059	PSD3	ENSG00000156011.16	Brain - Spinal cord (cervical c-1)	A=0.454	T=0.546	0.411994	2.93E-06
rs79441119	rs11997879	chr8:18657450	0.016730689	0.832333333	PSD3	ENSG00000156011.16	Brain - Substantia nigra	T=0.503	A=0.497	0.261377	3.09E-06
rs79441119	rs34059664	chr8:18657698	0.016650842	0.831997328	PSD3	ENSG00000156011.16	Brain - Substantia nigra	A=0.504	G=0.496	0.261377	3.09E-06
rs79441119	rs11993401	chr8:18657427	0.016650842	0.831997328	PSD3	ENSG00000156011.16	Brain - Substantia nigra	C=0.504	T=0.496	0.264322	3.14E-06
rs79441119	rs11993467	chr8:18657661	0.016571315	0.831659973	PSD3	ENSG00000156011.16	Brain - Substantia nigra	C=0.505	T=0.495	0.264322	3.14E-06
rs79441119	rs17696125	chr8:18574874	0.001117834	1	PSD3	ENSG00000156011.16	Small Intestine - Terminal Ileum	T=0.956	C=0.044	0.410081	3.38E-06
rs79441119	rs7841096	chr8:18632333	0.039255828	1	PSD3	ENSG00000156011.16	Brain - Spinal cord (cervical c-1)	A=0.384	T=0.616	0.378279	3.44E-06
rs79441119	rs59321198	chr8:18866979	0.002908867	1	PSD3	ENSG00000156011.16	Esophagus - Mucosa	C=0.894	G=0.106	0.217409	3.55E-06
rs79441119	rs78656518	chr8:18844514	0.00014664	1	PSD3	ENSG00000156011.16	Esophagus - Muscularis	T=0.994	C=0.006	0.494999	4.95E-06
rs79441119	rs111699729	chr8:18865650	0.002908867	1	PSD3	ENSG00000156011.16	Esophagus - Mucosa	G=0.894	A=0.106	0.213434	5.55E-06
rs79441119	rs7010324	chr8:18866227	0.002817906	1	PSD3	ENSG00000156011.16	Esophagus - Mucosa	C=0.897	T=0.103	0.213434	5.55E-06
rs79441119	rs6996112	chr8:18866326	0.002939322	1	PSD3	ENSG00000156011.16	Esophagus - Mucosa	T=0.893	C=0.107	0.213434	5.55E-06
rs79441119	rs7010496	chr8:18866368	0.002908867	1	PSD3	ENSG00000156011.16	Esophagus - Mucosa	C=0.894	T=0.106	0.213434	5.55E-06
rs79441119	rs117966479	chr8:18893665	0.001332155	1	PSD3	ENSG00000156011.16	Small Intestine - Terminal Ileum	T=0.948	C=0.052	0.630419	5.82E-06
rs79441119	rs77386793	chr8:18869663	0.002908867	1	PSD3	ENSG00000156011.16	Esophagus - Mucosa	C=0.894	A=0.106	0.225491	6.32E-06
rs79441119	rs34426360	chr8:18575404	0.001091294	1	PSD3	ENSG00000156011.16	Small Intestine - Terminal Ileum	-=0.957	AGA=0.043	0.457749	7.58E-06
rs79441119	rs1872890	chr8:18575581	0.00106481	1	PSD3	ENSG00000156011.16	Small Intestine - Terminal Ileum	A=0.958	G=0.042	0.457749	7.58E-06
rs79441119	rs1038611	chr8:18624327	0.042920514	1	PSD3	ENSG00000156011.16	Brain - Spinal cord (cervical c-1)	C=0.363	T=0.637	0.378354	7.96E-06
rs79441119	rs1038610	chr8:18624424	0.042920514	1	PSD3	ENSG00000156011.16	Brain - Spinal cord (cervical c-1)	C=0.363	T=0.637	0.378354	7.96E-06
rs79441119	rs146517899	chr8:18867154	0.002939322	1	PSD3	ENSG00000156011.16	Esophagus - Mucosa	C=0.893	T=0.107	0.210362	8.66E-06
rs79441119	rs56990136	chr8:18872866	0.002969845	1	PSD3	ENSG00000156011.16	Esophagus - Mucosa	C=0.892	T=0.108	0.21699	9.52E-06
rs79441119	rs73594648	chr8:18876592	0.002969845	1	PSD3	ENSG00000156011.16	Esophagus - Mucosa	A=0.892	T=0.108	0.208395	1.24E-05
rs79441119	rs141114042	chr8:18631690	0.04009173	1	PSD3	ENSG00000156011.16	Brain - Spinal cord (cervical c-1)	TTAA=0.379	-=0.621	0.367347	1.35E-05
rs79441119	rs116324874	chr8:18876557	0.002969845	1	PSD3	ENSG00000156011.16	Esophagus - Mucosa	G=0.892	A=0.108	0.219157	1.52E-05
rs79441119	rs80027157	chr8:18866078	0.002969845	1	PSD3	ENSG00000156011.16	Esophagus - Mucosa	A=0.892	G=0.108	0.208302	1.57E-05
rs79441119	rs7009185	chr8:18866220	0.002908867	1	PSD3	ENSG00000156011.16	Esophagus - Mucosa	G=0.894	A=0.106	0.208302	1.57E-05
rs79441119	rs7010821	chr8:18866541	0.002908867	1	PSD3	ENSG00000156011.16	Esophagus - Mucosa	C=0.894	T=0.106	0.208302	1.57E-05
rs79441119	rs375703979	chr8:18866779	0.002908867	1	PSD3	ENSG00000156011.16	Esophagus - Mucosa	AG=0.894	-=0.106	0.208302	1.57E-05
rs79441119	rs35675493	chr8:18653677	0.023487095	1	PSD3	ENSG00000156011.16	Brain - Spinal cord (cervical c-1)	CCC=0.51	-=0.49	0.381151	1.59E-05
rs79441119	rs9644614	chr8:18630435	0.043856076	1	PSD3	ENSG00000156011.16	Brain - Spinal cord (cervical c-1)	C=0.358	T=0.642	0.374175	1.59E-05
rs79441119	rs9644615	chr8:18630532	0.04366689	1	PSD3	ENSG00000156011.16	Brain - Spinal cord (cervical c-1)	C=0.359	A=0.641	0.374175	1.59E-05
rs79441119	rs189718418	chr8:18857486	0.000122078	1	PSD3	ENSG00000156011.16	Esophagus - Muscularis	A=0.995	G=0.005	0.496246	1.84E-05
rs79441119	rs2410598	chr8:18872462	0.002969845	1	PSD3	ENSG00000156011.16	Esophagus - Mucosa	C=0.892	T=0.108	0.204344	1.89E-05
rs79441119	rs57855654	chr8:18872943	0.002969845	1	PSD3	ENSG00000156011.16	Esophagus - Mucosa	G=0.892	A=0.108	0.204344	1.89E-05
rs79441119	rs76653031	chr8:18574691	0.000445262	1	PSD3	ENSG00000156011.16	Small Intestine - Terminal Ileum	C=0.982	A=0.018	0.614718	2.15E-05
rs79441119	rs35592282	chr8:18576601	0.000445262	1	PSD3	ENSG00000156011.16	Small Intestine - Terminal Ileum	A=0.982	-=0.018	0.614718	2.15E-05
rs79441119	rs143185883	chr8:18577856	0.000445262	1	PSD3	ENSG00000156011.16	Small Intestine - Terminal Ileum	C=0.982	-=0.018	0.614718	2.15E-05
rs79441119	rs79697401	chr8:18578261	0.000445262	1	PSD3	ENSG00000156011.16	Small Intestine - Terminal Ileum	T=0.982	C=0.018	0.614718	2.15E-05
rs79441119	rs17127092	chr8:18579361	0.000445262	1	PSD3	ENSG00000156011.16	Small Intestine - Terminal Ileum	A=0.982	G=0.018	0.614718	2.15E-05
rs79441119	rs12056418	chr8:18580835	0.000445262	1	PSD3	ENSG00000156011.16	Small Intestine - Terminal Ileum	C=0.982	T=0.018	0.614718	2.15E-05
rs79441119	rs76973373	chr8:18583218	0.000445262	1	PSD3	ENSG00000156011.16	Small Intestine - Terminal Ileum	C=0.982	T=0.018	0.614718	2.15E-05
rs79441119	rs79550828	chr8:18587562	0.000445262	1	PSD3	ENSG00000156011.16	Small Intestine - Terminal Ileum	G=0.982	C=0.018	0.614718	2.15E-05
rs79441119	rs79052288	chr8:18589608	0.000445262	1	PSD3	ENSG00000156011.16	Small Intestine - Terminal Ileum	C=0.982	G=0.018	0.614718	2.15E-05
rs79441119	rs57825650	chr8:18591403	0.000445262	1	PSD3	ENSG00000156011.16	Small Intestine - Terminal Ileum	A=0.982	G=0.018	0.614718	2.15E-05
rs79441119	rs60549282	chr8:18877472	0.002817906	1	PSD3	ENSG00000156011.16	Esophagus - Mucosa	C=0.897	T=0.103	0.198321	3.37E-05
rs79441119	rs4463468	chr8:18879717	0.002817906	1	PSD3	ENSG00000156011.16	Esophagus - Mucosa	C=0.897	T=0.103	0.198321	3.37E-05
rs79441119	rs75123216	chr8:18821251	0.000445262	1	PSD3	ENSG00000156011.16	Esophagus - Muscularis	T=0.982	C=0.018	0.413555	3.38E-05
rs79441119	rs116971711	chr8:18822209	0.000445262	1	PSD3	ENSG00000156011.16	Esophagus - Muscularis	G=0.982	A=0.018	0.413555	3.38E-05

rs79441119	rs77537870	chr8:18877244	0.002969845	1	PSD3	ENSG00000156011.16	Esophagus - Mucosa	A=0.892	G=0.108	0.202602	3.49E-05
rs79441119	rs61050926	chr8:18864611	0.002939322	1	PSD3	ENSG00000156011.16	Esophagus - Mucosa	G=0.893	A=0.107	0.185851	4.35E-05
rs79441119	rs57965775	chr8:18865248	0.002939322	1	PSD3	ENSG00000156011.16	Esophagus - Mucosa	C=0.893	G=0.107	0.185851	4.35E-05
rs79441119	rs73199994	chr8:18646365	0.002140144	1	PSD3	ENSG00000156011.16	Brain - Cerebellum	C=0.919	T=0.081	0.375659	4.63E-05
rs79441119	rs76595146	chr8:18873146	0.002969845	1	PSD3	ENSG00000156011.16	Esophagus - Mucosa	G=0.892	T=0.108	0.198351	5.28E-05
rs79441119	rs79513952	chr8:18875713	0.002969845	1	PSD3	ENSG00000156011.16	Esophagus - Mucosa	G=0.892	A=0.108	0.198351	5.28E-05
rs79441119	rs115432345	chr8:18871863	0.003000436	1	PSD3	ENSG00000156011.16	Esophagus - Mucosa	G=0.891	T=0.109	0.194814	6.45E-05
rs79441119	rs73594633	chr8:18871992	0.002969845	1	PSD3	ENSG00000156011.16	Esophagus - Mucosa	C=0.892	G=0.108	0.189659	6.52E-05
rs79441119	rs145752806	chr8:18882886	0.002817906	1	PSD3	ENSG00000156011.16	Esophagus - Mucosa	ACTC=0.897	-=0.103	0.194717	7.13E-05
rs79441119	rs78119471	chr8:18883327	0.002817906	1	PSD3	ENSG00000156011.16	Esophagus - Mucosa	A=0.897	G=0.103	0.194717	7.13E-05
rs79441119	rs60074764	chr8:18863855	0.002969845	1	PSD3	ENSG00000156011.16	Esophagus - Mucosa	T=0.892	C=0.108	0.173434	8.93E-05
rs79441119	rs60016927	chr8:18863989	0.002969845	1	PSD3	ENSG00000156011.16	Esophagus - Mucosa	T=0.892	C=0.108	0.173434	8.93E-05
rs79441119	rs79233079	chr8:19029881	0.003031096	1	PSD3	ENSG00000156011.16	Cells - Cultured fibroblasts	C=0.89	T=0.11	-0.168053	0.000116145
