# Supplementary material for: A genome-wide association study for survival from a multi-centre European study identified variants associated with COVID-19 risk of death
Source: Sci Rep. 2024 Feb 6;14:3000. doi: 10.1038/s41598-024-53310-x (PMC10847137; doi:10.1038/s41598-024-53310-x)
Supplement: Supplementary file 8 — Supplementary Table S6. [file 41598_2024_53310_MOESM8_ESM.pdf]

Supplementary Table S6. Genes mapping within 50 kbp of the top 113 SNPs

Gene symbol	Gene name	Chromosome	Gene start (bp)	Gene end (bp)
ACVR1C	activin A receptor type 1C	2	157,526,767	157,628,864
ARFGEF3	ARFGEF family member 3	6	138,161,939	138,344,663
B3GALT1	beta-1,3-galactosyltransferase 1	2	167,293,001	167,874,045
CD300A	CD300a molecule	17	74,466,399	74,484,794
CDH13	cadherin 13	16	82,626,965	83,800,640
CECR2	CECR2 histone acetyl-lysine reader	22	17,359,949	17,558,151
CSMD1	CUB and Sushi multiple domains 1	8	2,935,353	4,994,972
CTNND2	catenin delta 2	5	10,971,836	11,904,446
CYTIP	cytohesin 1 interacting protein	2	157,414,619	157,488,961
EPHB4	EPH receptor B4	7	100,802,565	100,827,523
EPO	erythropoietin	7	100,720,468	100,723,700
FAM162B	family with sequence similarity 162 member B	6	116,752,197	116,765,719
FGF19	fibroblast growth factor 19	11	69,698,238	69,704,022
FMN1	formin 1	15	32,765,544	33,194,714
GPR142	G protein-coupled receptor 142	17	74,367,458	74,372,600
GPRC5C	G protein-coupled receptor class C group 5 member C	17	74,424,851	74,451,653
GPRC6A	G protein-coupled receptor class C group 6 member A	6	116,792,085	116,829,083
GUCY1A2	guanylate cyclase 1 soluble subunit alpha 2	11	106,674,019	107,018,476
KLRC3	killer cell lectin like receptor C3	12	10,412,315	10,420,595
KLRC4	killer cell lectin like receptor C4	12	10,407,384	10,409,757
KLRC4-KLRK1	KLRC4-KLRK1 readthrough	12	10,372,353	10,410,146
KLRD1	killer cell lectin like receptor D1	12	10,226,058	10,329,608
KLRK1	killer cell lectin like receptor K1	12	10,372,353	10,391,874
KPNA5	karyopherin subunit alpha 5	6	116,681,187	116,741,867
LTO1	LTO1 maturation factor of ABCE1	11	69,653,076	69,675,416
MARCHF6	membrane associated ring-CH-type finger 6	5	10,353,695	10,440,388
MEIS2	Meis homeobox 2	15	36,889,204	37,101,299
NCL	nucleolin	2	231,453,531	231,483,641
NMUR1	neuromedin U receptor 1	2	231,523,187	231,530,445
PERP	p53 apoptosis effector related to PMP22	6	138,088,505	138,107,419
PSD3	pleckstrin and Sec7 domain containing 3	8	18,527,303	19,084,730
RFX6	regulatory factor X6	6	116,877,212	116,932,161
ROPN1L	rhophilin associated tail protein 1 like	5	10,441,524	10,472,029
SPPL2A	signal peptide peptidase like 2A	15	50,702,266	50,765,948
TMEM132E	transmembrane protein 132E	17	34,579,487	34,639,318
WWOX	WW domain containing oxidoreductase	16	78,099,400	79,212,667
ZAN	zonadhesin	7	100,733,595	100,797,797
ZNF474	zinc finger protein 474	5	122,129,546	122,182,658
