# Supplementary material for: A genome-wide association study for survival from a multi-centre European study identified variants associated with COVID-19 risk of death
Source: Sci Rep. 2024 Feb 6;14:3000. doi: 10.1038/s41598-024-53310-x (PMC10847137; doi:10.1038/s41598-024-53310-x)
Supplement: Supplementary file 9 — Supplementary Table S7. [file 41598_2024_53310_MOESM9_ESM.pdf]

Supplementary Table S7. Enriched functional terms identified with DAVID.

Category	Term	Count	%	P-value	Genes	List Total	Pop Hits	Pop Total	Fold Enrichment	Bonferroni	Benjamini	FDR
GOTERM_BP_DIRECT	GO:0045954~positive regulation of natural killer cell mediated cytotoxicity	5	13.16	1.51E-07	KLRK1, KLRC3, KLRC4, KLRC4-KLRK1, KLRD1	36	27	19414	99.87	3.97E-05	3.97E-05	**3.97E-05**
GOTERM_BP_DIRECT	GO:0002223~stimulatory C-type lectin receptor signaling pathway	5	13.16	1.37E-06	KLRK1, KLRC3, KLRC4, KLRC4-KLRK1, KLRD1	36	46	19414	58.62	3.60E-04	1.80E-04	**1.80E-04**
INTERPRO	IPR016186:C-type lectin-like	5	13.16	4.56E-05	KLRK1, KLRC3, KLRC4, KLRC4-KLRK1, KLRD1	37	106	19144	24.41	4.46E-03	2.97E-03	**2.97E-03**
INTERPRO	IPR016187:C-type lectin fold	5	13.16	6.06E-05	KLRK1, KLRC3, KLRC4, KLRC4-KLRK1, KLRD1	37	114	19144	22.69	5.93E-03	2.97E-03	**2.97E-03**
UP_SEQ_FEATURE	TOPO_DOM:Extracellular	16	42.11	5.89E-05	TMEM132E, KLRC3, CD300A, KLRC4, ZAN, GPRC6A, KLRK1, GPR142, ACVR1C, KLRC4-KLRK1, NMUR1, KLRD1, GPRC5C, CSMD1, EPHB4, MARCHF6	37	2941	20543	3.02	1.26E-02	9.59E-03	**9.59E-03**
UP_SEQ_FEATURE	TOPO_DOM:Cytoplasmic	18	47.37	8.92E-05	TMEM132E, KLRC3, CD300A, KLRC4, ZAN, SPPL2A, GPRC6A, KLRK1, GPR142, ACVR1C, B3GALT1, KLRC4-KLRK1, NMUR1, KLRD1, GPRC5C, CSMD1, EPHB4, MARCHF6	37	3837	20543	2.60	1.90E-02	9.59E-03	**9.59E-03**
BIOCARTA	h_nkcellsPathway:Ras-Independent pathway in NK cell-mediated cytotoxicity	3	7.89	2.10E-03	KLRC3, KLRC4, KLRD1	7	20	1623	34.78	2.08E-02	1.89E-02	**1.89E-02**
UP_KW_MOLECULAR_FUNCTION	KW-0675~Receptor	12	31.58	9.42E-04	GPRC6A, KLRK1, GPR142, ACVR1C, CD300A, KLRC3, KLRC4, KLRC4-KLRK1, KLRD1, NMUR1, GPRC5C, EPHB4	27	1815	11749	2.88	2.05E-02	2.07E-02	**2.07E-02**
INTERPRO	IPR001304:C-type lectin	4	10.53	6.41E-04	KLRK1, KLRC3, KLRC4-KLRK1, KLRD1	37	90	19144	23.00	6.09E-02	2.09E-02	**2.09E-02**
GOTERM_CC_DIRECT	GO:0043235~receptor complex	5	13.16	5.68E-04	ACVR1C, KLRC3, KLRD1, GPRC5C, EPHB4	37	220	20624	12.67	3.95E-02	2.27E-02	**2.24E-02**
GOTERM_CC_DIRECT	GO:0005887~integral component of plasma membrane	10	26.32	6.39E-04	GPRC6A, KLRK1, KLRC3, PERP, KLRC4, KLRC4-KLRK1, KLRD1, NMUR1, GPRC5C, EPHB4	37	1438	20624	3.88	4.44E-02	2.27E-02	**2.24E-02**
UP_KW_LIGAND	KW-0430~Lectin	4	10.53	2.24E-03	KLRK1, KLRC3, KLRC4-KLRK1, KLRD1	12	173	6858	13.21	2.22E-02	2.24E-02	**2.24E-02**
UP_KW_DOMAIN	KW-0735~Signal-anchor	6	15.79	3.78E-03	KLRK1, KLRC3, KLRC4, B3GALT1, KLRC4-KLRK1, KLRD1	34	466	14504	5.49	4.44E-02	2.61E-02	**2.61E-02**
UP_KW_DOMAIN	KW-1133~Transmembrane helix	22	57.89	5.67E-03	ZNF474, TMEM132E, KLRC3, CD300A, KLRC4, ZAN, SPPL2A, GPRC6A, KLRK1, GPR142, ACVR1C, FAM162B, PERP, B3GALT1, KLRC4-KLRK1, NMUR1, KLRD1, GPRC5C, CSMD1, EPHB4, ARFGEF3, MARCHF6	34	5844	14504	1.61	6.59E-02	2.61E-02	**2.61E-02**
UP_KW_DOMAIN	KW-0812~Transmembrane	22	57.89	6.53E-03	ZNF474, TMEM132E, KLRC3, CD300A, KLRC4, ZAN, SPPL2A, GPRC6A, KLRK1, GPR142, ACVR1C, FAM162B, PERP, B3GALT1, KLRC4-KLRK1, NMUR1, KLRD1, GPRC5C, CSMD1, EPHB4, ARFGEF3, MARCHF6	34	5905	14504	1.59	7.56E-02	2.61E-02	**2.61E-02**
GOTERM_CC_DIRECT	GO:0009897~external side of plasma membrane	6	15.79	1.35E-03	KLRK1, KLRC3, KLRC4, KLRC4-KLRK1, CDH13, KLRD1	37	477	20624	7.01	9.16E-02	3.20E-02	**3.15E-02**
UP_SEQ_FEATURE	DOMAIN:C-type lectin	4	10.53	4.90E-04	KLRK1, KLRC3, KLRC4-KLRK1, KLRD1	37	88	20543	25.24	9.99E-02	3.44E-02	**3.44E-02**
UP_SEQ_FEATURE	TRANSMEM:Helical; Signal-anchor for type II membrane protein	6	15.79	6.40E-04	KLRK1, KLRC3, KLRC4, B3GALT1, KLRC4-KLRK1, KLRD1	37	402	20543	8.29	1.29E-01	3.44E-02	**3.44E-02**
SMART	SM00034:CLECT	4	10.53	1.05E-03	KLRK1, KLRC3, KLRC4-KLRK1, KLRD1	25	88	10378	18.87	3.40E-02	3.46E-02	**3.46E-02**
UP_KW_PTM	KW-0325~Glycoprotein	17	44.74	5.45E-03	TMEM132E, EPO, KLRC3, CD300A, CTNND2, ZAN, SPPL2A, GPRC6A, KLRK1, B3GALT1, KLRC4-KLRK1, CDH13, NMUR1, KLRD1, GPRC5C, CSMD1, EPHB4	28	4740	14111	1.81	4.80E-02	5.45E-02	5.45E-02
GOTERM_CC_DIRECT	GO:0005886~plasma membrane	18	47.37	4.61E-03	WWOX, KLRC3, CD300A, CTNND2, ZAN, SPPL2A, FMN1, GPRC6A, KLRK1, GPR142, ACVR1C, PERP, KLRC4-KLRK1, CDH13, NMUR1, KLRD1, GPRC5C, EPHB4	37	5333	20624	1.88	2.80E-01	8.18E-02	8.07E-02
UP_KW_BIOLOGICAL_PROCESS	KW-0130~Cell adhesion	5	13.16	5.44E-03	CD300A, PERP, CTNND2, ZAN, CDH13	18	492	11262	6.36	8.85E-02	9.24E-02	9.24E-02
KEGG_PATHWAY	hsa04650:Natural killer cell mediated cytotoxicity	4	10.53	2.68E-03	KLRK1, KLRC3, KLRC4-KLRK1, KLRD1	20	127	8465	13.33	1.11E-01	1.18E-01	1.18E-01
UP_SEQ_FEATURE	MUTAGEN:R->A: Inhibits association with the HCST signaling dimer.	2	5.26	3.50E-03	KLRK1, KLRC4-KLRK1	37	2	20543	555.22	5.30E-01	1.51E-01	1.51E-01
UP_SEQ_FEATURE	CARBOHYD:N-linked (GlcNAc...) asparagine	16	42.11	4.60E-03	TMEM132E, EPO, KLRC3, CD300A, ZAN, SPPL2A, GPRC6A, KLRK1, B3GALT1, KLRC4-KLRK1, CDH13, NMUR1, KLRD1, GPRC5C, CSMD1, EPHB4	37	4375	20543	2.03	6.29E-01	1.61E-01	1.61E-01
UP_SEQ_FEATURE	TRANSMEM:Helical	18	47.37	5.24E-03	ZNF474, TMEM132E, CD300A, ZAN, SPPL2A, KLRK1, GPR142, ACVR1C, FAM162B, PERP, KLRC4-KLRK1, NMUR1, KLRD1, GPRC5C, CSMD1, EPHB4, ARFGEF3, MARCHF6	37	5374	20543	1.86	6.77E-01	1.61E-01	1.61E-01
UP_KW_PTM	KW-1015~Disulfide bond	13	34.21	3.87E-02	EPO, KLRC3, CD300A, KLRC4, ZAN, GPRC6A, KLRK1, FGF19, KLRC4-KLRK1, NMUR1, KLRD1, CSMD1, EPHB4	28	3816	14111	1.72	2.99E-01	1.94E-01	1.94E-01
GOTERM_MF_DIRECT	GO:0004888~transmembrane signaling receptor activity	4	10.53	4.22E-03	CD300A, KLRC3, KLRC4, KLRD1	34	188	18945	11.86	2.84E-01	2.03E-01	2.03E-01
GOTERM_MF_DIRECT	GO:0030246~carbohydrate binding	4	10.53	5.15E-03	KLRK1, KLRC3, KLRC4-KLRK1, KLRD1	34	202	18945	11.03	3.35E-01	2.03E-01	2.03E-01
GOTERM_MF_DIRECT	GO:0032394~MHC class Ib receptor activity	2	5.26	8.68E-03	KLRK1, KLRC4-KLRK1	34	5	18945	222.88	4.98E-01	2.29E-01	2.29E-01
KEGG_PATHWAY	hsa04612:Antigen processing and presentation	3	7.89	1.30E-02	KLRC3, KLRC4, KLRD1	20	78	8465	16.28	4.36E-01	2.85E-01	2.85E-01
UP_KW_CELLULAR_COMPONENT	KW-0472~Membrane	25	65.79	1.88E-02	ZNF474, ZAN, SPPL2A, FMN1, GPRC6A, KLRK1, ACVR1C, FAM162B, PERP, PSD3, B3GALT1, KLRC4-KLRK1, CSMD1, EPHB4, ARFGEF3, MARCHF6, TMEM132E, KLRC3, CD300A, KLRC4, GPR142, CDH13, NMUR1, KLRD1, GPRC5C	38	8234	17708	1.41	2.61E-01	3.00E-01	3.00E-01
UP_KW_CELLULAR_COMPONENT	KW-1003~Cell membrane	14	36.84	4.92E-02	CD300A, ZAN, FMN1, GPRC6A, KLRK1, GPR142, PERP, PSD3, KLRC4-KLRK1, CDH13, NMUR1, KLRD1, GPRC5C, EPHB4	38	3923	17708	1.66	5.54E-01	3.94E-01	3.94E-01

GOTERM_BP_DIRECT	GO:2000502~negative regulation of natural killer cell chemotaxis	2	5.26	5.40E-03	KLRK1, KLRC4-KLRK1	36	3	19414	359.52	7.59E-01	4.73E-01	4.73E-01
GOTERM_CC_DIRECT	GO:0005912~adherens junction	3	7.89	3.97E-02	CTNND2, CDH13, FMN1	37	181	20624	9.24	9.44E-01	5.01E-01	4.94E-01
GOTERM_CC_DIRECT	GO:0016020~membrane	12	31.58	4.23E-02	KLRK1, TMEM132E, FAM162B, CD300A, NCL, KLRC4, B3GALT1, SPPL2A, KLRC4-KLRK1, NMUR1, CSMD1, MARCHF6	37	3653	20624	1.83	9.54E-01	5.01E-01	4.94E-01
SMART	SM00222:Sec7	2	5.26	3.64E-02	PSD3, ARFGEF3	25	16	10378	51.89	7.06E-01	6.00E-01	6.00E-01
GOTERM_CC_DIRECT	GO:0016021~integral component of membrane	15	39.47	6.45E-02	TMEM132E, CD300A, ZAN, SPPL2A, KLRK1, GPR142, FAM162B, B3GALT1, KLRC4-KLRK1, NMUR1, KLRD1, GPRC5C, CSMD1, ARFGEF3, MARCHF6	37	5394	20624	1.55	9.91E-01	6.54E-01	6.45E-01
GOTERM_MF_DIRECT	GO:0042288~MHC class I protein binding	2	5.26	3.43E-02	KLRK1, KLRC4-KLRK1	34	20	18945	55.72	9.36E-01	6.77E-01	6.77E-01
GOTERM_BP_DIRECT	GO:0032814~regulation of natural killer cell activation	2	5.26	1.08E-02	KLRC3, KLRD1	36	6	19414	179.76	9.42E-01	7.08E-01	7.08E-01
UP_SEQ_FEATURE	DOMAIN:SEC7	2	5.26	2.94E-02	PSD3, ARFGEF3	37	17	20543	65.32	9.98E-01	7.43E-01	7.43E-01
UP_SEQ_FEATURE	REPEAT:ARM 9	2	5.26	3.11E-02	CTNND2, KPNA5	37	18	20543	61.69	9.99E-01	7.43E-01	7.43E-01
INTERPRO	IPR000904:SEC7-like	2	5.26	3.15E-02	PSD3, ARFGEF3	37	17	19144	60.87	9.57E-01	7.72E-01	7.72E-01
UP_SEQ_FEATURE	REPEAT:ARM 8	2	5.26	4.12E-02	CTNND2, KPNA5	37	24	20543	46.27	1.00E+00	8.21E-01	8.21E-01
UP_SEQ_FEATURE	REPEAT:ARM 7	2	5.26	4.46E-02	CTNND2, KPNA5	37	26	20543	42.71	1.00E+00	8.21E-01	8.21E-01
UP_SEQ_FEATURE	REPEAT:ARM	2	5.26	4.63E-02	CTNND2, KPNA5	37	27	20543	41.13	1.00E+00	8.21E-01	8.21E-01
UP_SEQ_FEATURE	REPEAT:ARM 6	2	5.26	4.96E-02	CTNND2, KPNA5	37	29	20543	38.29	1.00E+00	8.21E-01	8.21E-01
INTERPRO	IPR017978:GPCR, family 3, C-terminal	2	5.26	4.24E-02	GPRC6A, GPRC5C	37	23	19144	44.99	9.86E-01	8.31E-01	8.31E-01
UP_SEQ_FEATURE	REPEAT:ARM 5	2	5.26	5.63E-02	CTNND2, KPNA5	37	33	20543	33.65	1.00E+00	8.64E-01	8.64E-01
GOTERM_MF_DIRECT	GO:0038023~signaling receptor activity	3	7.89	5.83E-02	KLRK1, CD300A, KLRC4-KLRK1	34	225	18945	7.43	9.91E-01	9.21E-01	9.21E-01
UP_SEQ_FEATURE	REPEAT:ARM 4	2	5.26	6.45E-02	CTNND2, KPNA5	37	38	20543	29.22	1.00E+00	9.25E-01	9.25E-01
GOTERM_MF_DIRECT	GO:0030295~protein kinase activator activity	2	5.26	7.07E-02	EPO, GPRC5C	34	42	18945	26.53	9.97E-01	9.31E-01	9.31E-01
UP_SEQ_FEATURE	REPEAT:ARM 3	2	5.26	7.11E-02	CTNND2, KPNA5	37	42	20543	26.44	1.00E+00	9.40E-01	9.40E-01
UP_SEQ_FEATURE	REPEAT:ARM 2	2	5.26	7.43E-02	CTNND2, KPNA5	37	44	20543	25.24	1.00E+00	9.40E-01	9.40E-01
SMART	SM00185:ARM	2	5.26	9.07E-02	CTNND2, KPNA5	25	41	10378	20.25	9.57E-01	9.98E-01	9.98E-01
GOTERM_BP_DIRECT	GO:0032012~regulation of ARF protein signal transduction	2	5.26	3.55E-02	PSD3, ARFGEF3	36	20	19414	53.93	1.00E+00	1.00E+00	1.00E+00
GOTERM_BP_DIRECT	GO:0006809~nitric oxide biosynthetic process	2	5.26	4.07E-02	KLRK1, KLRC4-KLRK1	36	23	19414	46.89	1.00E+00	1.00E+00	1.00E+00
GOTERM_BP_DIRECT	GO:0030101~natural killer cell activation	2	5.26	4.59E-02	KLRK1, KLRC4-KLRK1	36	26	19414	41.48	1.00E+00	1.00E+00	1.00E+00
GOTERM_BP_DIRECT	GO:0072332~intrinsic apoptotic signaling pathway by p53 class mediator	2	5.26	4.76E-02	WWOX, PERP	36	27	19414	39.95	1.00E+00	1.00E+00	1.00E+00
GOTERM_BP_DIRECT	GO:0098609~cell-cell adhesion	3	7.89	4.81E-02	PERP, CTNND2, ZAN	36	195	19414	8.30	1.00E+00	1.00E+00	1.00E+00
GOTERM_BP_DIRECT	GO:0042267~natural killer cell mediated cytotoxicity	2	5.26	5.10E-02	KLRK1, KLRC4-KLRK1	36	29	19414	37.19	1.00E+00	1.00E+00	1.00E+00
GOTERM_BP_DIRECT	GO:0048705~skeletal system morphogenesis	2	5.26	5.96E-02	WWOX, FMN1	36	34	19414	31.72	1.00E+00	1.00E+00	1.00E+00
GOTERM_BP_DIRECT	GO:0034260~negative regulation of GTPase activity	2	5.26	6.30E-02	KLRK1, KLRC4-KLRK1	36	36	19414	29.96	1.00E+00	1.00E+00	1.00E+00
GOTERM_BP_DIRECT	GO:0031295~T cell costimulation	2	5.26	6.97E-02	KLRK1, KLRC4-KLRK1	36	40	19414	26.96	1.00E+00	1.00E+00	1.00E+00
GOTERM_BP_DIRECT	GO:0007043~cell-cell junction assembly	2	5.26	7.97E-02	CTNND2, CDH13	36	46	19414	23.45	1.00E+00	1.00E+00	1.00E+00
GOTERM_BP_DIRECT	GO:0045429~positive regulation of nitric oxide biosynthetic process	2	5.26	7.97E-02	KLRK1, KLRC4-KLRK1	36	46	19414	23.45	1.00E+00	1.00E+00	1.00E+00
INTERPRO	IPR000225:Armadillo	2	5.26	8.13E-02	CTNND2, KPNA5	37	45	19144	23.00	1.00E+00	1.00E+00	1.00E+00
