# Supplementary material for: A genome-wide association study for survival from a multi-centre European study identified variants associated with COVID-19 risk of death
Source: Sci Rep. 2024 Feb 6;14:3000. doi: 10.1038/s41598-024-53310-x (PMC10847137; doi:10.1038/s41598-024-53310-x)
Supplement: Supplementary file 10 — Supplementary Table S8. [file 41598_2024_53310_MOESM10_ESM.pdf]

Supplementary Table S8. Enriched functional gene sets identified by FUMA.

Category	GeneSet	N_genes	N_overlap	p	adjP	genes	link
GO_mf	GO MOLECULAR TRANSDUCER ACTIVITY	1529	11	1.62E-07	**2.31E-04**	KLRD1, KLRC4-KLRK1, KLRK1, NKG2-E, KLRC3, GPR142, GPRC5C, CD300A, ACVR1C, NMUR1, GPRC6A, EPHB4	http://www.gsea-msigdb.org/gsea/msigdb/cards/GO_MOLECULAR_TRANSDUCER_ACTIVITY
GO_mf	GO TRANSMEMBRANE SIGNALING RECEPTOR ACTIVITY	1263	10	2.81E-07	**2.31E-04**	KLRD1, KLRC4-KLRK1, KLRK1, NKG2-E, KLRC3, GPR142, GPRC5C, ACVR1C, NMUR1, GPRC6A, EPHB4	http://www.gsea-msigdb.org/gsea/msigdb/cards/GO_TRANSMEMBRANE_SIGNALING_RECEPTOR_ACTIVITY
BioCarta	BIOCARTA NKCELLS PATHWAY	20	3	1.02E-06	**2.95E-04**	KLRD1, KLRC4, NKG2-E, KLRC3	http://www.gsea-msigdb.org/gsea/msigdb/cards/BIOCARTA_NKCELLS_PATHWAY
GO_bp	GO BIOLOGICAL ADHESION	1404	11	6.86E-08	**5.04E-04**	KLRC4-KLRK1, KLRK1, FMN1, CDH13, CD300A, CYTIP, CTNND2, PERP, EPO, ZAN, EPHB4	http://www.gsea-msigdb.org/gsea/msigdb/cards/GO_BIOLOGICAL_ADHESION
Positional_gene_sets	chr6q22	106	4	3.81E-06	**1.14E-03**	KPNA5, FAM162B, GPRC6A, RFX6	http://www.gsea-msigdb.org/gsea/msigdb/cards/chr6q22
Positional_gene_sets	chr12p13	292	5	1.01E-05	**1.51E-03**	KLRD1, KLRC4-KLRK1, KLRK1, KLRC4, NKG2-E, KLRC3	http://www.gsea-msigdb.org/gsea/msigdb/cards/chr12p13
Cancer_gene_neighborhoods	GNF2 RAB7L1	33	3	4.84E-06	**1.59E-03**	KLRD1, NKG2-E, KLRC3, CYTIP	http://www.gsea-msigdb.org/gsea/msigdb/cards/GNF2_RAB7L1
Cancer_gene_neighborhoods	GNF2 CD7	38	3	7.45E-06	**1.59E-03**	KLRD1, KLRK1, NKG2-E, KLRC3	http://www.gsea-msigdb.org/gsea/msigdb/cards/GNF2_CD7
Canonical_Pathways	BIOCARTA NKCELLS PATHWAY	20	3	1.02E-06	**2.24E-03**	KLRD1, KLRC4, NKG2-E, KLRC3	http://www.gsea-msigdb.org/gsea/msigdb/cards/BIOCARTA_NKCELLS_PATHWAY
Cancer_gene_neighborhoods	GNF2 PTPN4	49	3	1.62E-05	**2.30E-03**	KLRD1, KLRK1, NKG2-E, KLRC3	http://www.gsea-msigdb.org/gsea/msigdb/cards/GNF2_PTPN4
Computational_gene_sets	GNF2 RAB7L1	33	3	4.84E-06	**3.20E-03**	KLRD1, NKG2-E, KLRC3, CYTIP	http://www.gsea-msigdb.org/gsea/msigdb/cards/GNF2_RAB7L1
Computational_gene_sets	GNF2 CD7	38	3	7.45E-06	**3.20E-03**	KLRD1, KLRK1, NKG2-E, KLRC3	http://www.gsea-msigdb.org/gsea/msigdb/cards/GNF2_CD7
GO_mf	GO MHC PROTEIN BINDING	37	3	6.87E-06	**3.77E-03**	KLRD1, KLRC4-KLRK1, KLRK1	http://www.gsea-msigdb.org/gsea/msigdb/cards/GO_MHC_PROTEIN_BINDING
GO_mf	GO MHC CLASS IB RECEPTOR ACTIVITY	5	2	9.62E-06	**3.96E-03**	KLRC4-KLRK1, KLRK1	http://www.gsea-msigdb.org/gsea/msigdb/cards/GO_MHC_CLASS_IB_RECEPTOR_ACTIVITY
GO_bp	GO CELL CELL ADHESION	819	8	1.13E-06	**4.17E-03**	KLRC4-KLRK1, KLRK1, CDH13, CD300A, CTNND2, PERP, EPO, ZAN	http://www.gsea-msigdb.org/gsea/msigdb/cards/GO_CELL_CELL_ADHESION
Computational_gene_sets	GNF2 PTPN4	49	3	1.62E-05	**4.62E-03**	KLRD1, KLRK1, NKG2-E, KLRC3	http://www.gsea-msigdb.org/gsea/msigdb/cards/GNF2_PTPN4
Curated_gene_sets	BIOCARTA NKCELLS PATHWAY	20	3	1.02E-06	**5.61E-03**	KLRD1, KLRC4, NKG2-E, KLRC3	http://www.gsea-msigdb.org/gsea/msigdb/cards/BIOCARTA_NKCELLS_PATHWAY
Immunologic_signatures	GSE19888 CTRL VS A3R ACT TREATED MAST CELL PRETREATED WITH A3R INH DN	198	5	1.53E-06	**7.44E-03**	KLRK1, SPPL2A, WWOX, TMEM132E, ZAN	http://www.gsea-msigdb.org/gsea/msigdb/cards/GSE19888_CTRL_VS_A3R_ACT_TREATED_MAST_CELL_PRETREATED_WITH_A3R_INH_DN
GO_bp	GO REGULATION OF CELL ADHESION	676	7	3.98E-06	**0.010**	KLRC4-KLRK1, KLRK1, FMN1, CDH13, CD300A, CYTIP, EPO	http://www.gsea-msigdb.org/gsea/msigdb/cards/GO_REGULATION_OF_CELL_ADHESION
TF_targets	YTAAYNGCT UNKNOWN	155	4	1.71E-05	**0.010**	MEIS2, CDH13, ACVR1C, CTNND2	http://www.gsea-msigdb.org/gsea/msigdb/cards/YTAAYNGCT_UNKNOWN
KEGG	KEGG ANTIGEN PROCESSING AND PRESENTATION	81	3	7.32E-05	**0.014**	KLRD1, KLRC4, NKG2-E, KLRC3	http://www.gsea-msigdb.org/gsea/msigdb/cards/KEGG_ANTIGEN_PROCESSING_AND_PRESENTATION
Positional_gene_sets	chr2q24	103	3	1.49E-04	**0.015**	CYTIP, ACVR1C, B3GALT1	http://www.gsea-msigdb.org/gsea/msigdb/cards/chr2q24
GO_bp	GO NEGATIVE REGULATION OF LEUKOCYTE MIGRATION	45	3	1.25E-05	**0.023**	KLRC4-KLRK1, KLRK1, CD300A	http://www.gsea-msigdb.org/gsea/msigdb/cards/GO_NEGATIVE_REGULATION_OF_LEUKOCYTE_MIGRATION
Cancer_gene_neighborhoods	GNF2 MATK	24	2	2.62E-04	**0.028**	KLRD1, KLRK1	http://www.gsea-msigdb.org/gsea/msigdb/cards/GNF2_MATK
KEGG	KEGG NATURAL KILLER CELL MEDIATED CYTOTOXICITY	132	3	3.11E-04	**0.029**	KLRD1, KLRK1, NKG2-E, KLRC3	http://www.gsea-msigdb.org/gsea/msigdb/cards/KEGG_NATURAL_KILLER_CELL_MEDIATED_CYTOTOXICITY
GO_bp	GO REGULATION OF MYELOID DENDRITIC CELL ACTIVATION	8	2	2.69E-05	**0.035**	KLRC4-KLRK1, KLRK1	http://www.gsea-msigdb.org/gsea/msigdb/cards/GO_REGULATION_OF_MYELOID_DENDRITIC_CELL_ACTIVATION
GO_bp	GO POSITIVE REGULATION OF NATURAL KILLER CELL MEDIATED IMMUNE RESPONSE TO TUMOR CELL	9	2	3.45E-05	**0.035**	KLRC4-KLRK1, KLRK1	http://www.gsea-msigdb.org/gsea/msigdb/cards/GO_POSITIVE_REGULATION_OF_NATURAL_KILLER_CELL_MEDIATED_IMMUNE_RESPONSE_TO_TUMOR_CELL
GO_bp	GO REGULATION OF NATURAL KILLER CELL CHEMOTAXIS	9	2	3.45E-05	**0.035**	KLRC4-KLRK1, KLRK1	http://www.gsea-msigdb.org/gsea/msigdb/cards/GO_REGULATION_OF_NATURAL_KILLER_CELL_CHEMOTAXIS
GO_bp	GO NATURAL KILLER CELL MEDIATED IMMUNITY	65	3	3.79E-05	**0.035**	KLRD1, KLRC4-KLRK1, KLRK1	http://www.gsea-msigdb.org/gsea/msigdb/cards/GO_NATURAL_KILLER_CELL_MEDIATED_IMMUNITY
GO_bp	GO POSITIVE REGULATION OF CELL ADHESION	397	5	4.41E-05	**0.035**	KLRC4-KLRK1, KLRK1, FMN1, CDH13, EPO	http://www.gsea-msigdb.org/gsea/msigdb/cards/GO_POSITIVE_REGULATION_OF_CELL_ADHESION
GO_bp	GO NATURAL KILLER CELL MEDIATED IMMUNE RESPONSE TO TUMOR CELL	11	2	5.27E-05	**0.035**	KLRC4-KLRK1, KLRK1	http://www.gsea-msigdb.org/gsea/msigdb/cards/GO_NATURAL_KILLER_CELL_MEDIATED_IMMUNE_RESPONSE_TO_TUMOR_CELL
GO_bp	GO NATURAL KILLER CELL CHEMOTAXIS	11	2	5.27E-05	**0.035**	KLRC4-KLRK1, KLRK1	http://www.gsea-msigdb.org/gsea/msigdb/cards/GO_NATURAL_KILLER_CELL_CHEMOTAXIS
GO_bp	GO POSITIVE REGULATION OF RESPONSE TO TUMOR CELL	12	2	6.32E-05	**0.039**	KLRC4-KLRK1, KLRK1	http://www.gsea-msigdb.org/gsea/msigdb/cards/GO_POSITIVE_REGULATION_OF_RESPONSE_TO_TUMOR_CELL
GO_mf	GO CARBOHYDRATE BINDING	270	4	1.48E-04	**0.049**	KLRD1, KLRC4-KLRK1, KLRK1, NKG2-E, KLRC3	http://www.gsea-msigdb.org/gsea/msigdb/cards/GO_CARBOHYDRATE_BINDING
GO_bp	GO NEGATIVE REGULATION OF LYMPHOCYTE MIGRATION	14	2	8.70E-05	**0.049**	KLRC4-KLRK1, KLRK1	http://www.gsea-msigdb.org/gsea/msigdb/cards/GO_NEGATIVE_REGULATION_OF_LYMPHOCYTE_MIGRATION
GO_mf	GO MHC CLASS I PROTEIN BINDING	20	2	1.81E-04	**0.050**	KLRC4-KLRK1, KLRK1	http://www.gsea-msigdb.org/gsea/msigdb/cards/GO_MHC_CLASS_I_PROTEIN_BINDING
